# Supplementary material for: Micro-osteoperforation for enhancement of orthodontic movement: A mechanical analysis using the finite element method
Source: PLoS One. 2024 Aug 19;19(8):e0308739. doi: 10.1371/journal.pone.0308739 (PMC11332926; doi:10.1371/journal.pone.0308739)
Supplement: S1 File — (PDF) [file pone.0308739.s001.pdf]

Study 1 without perforations

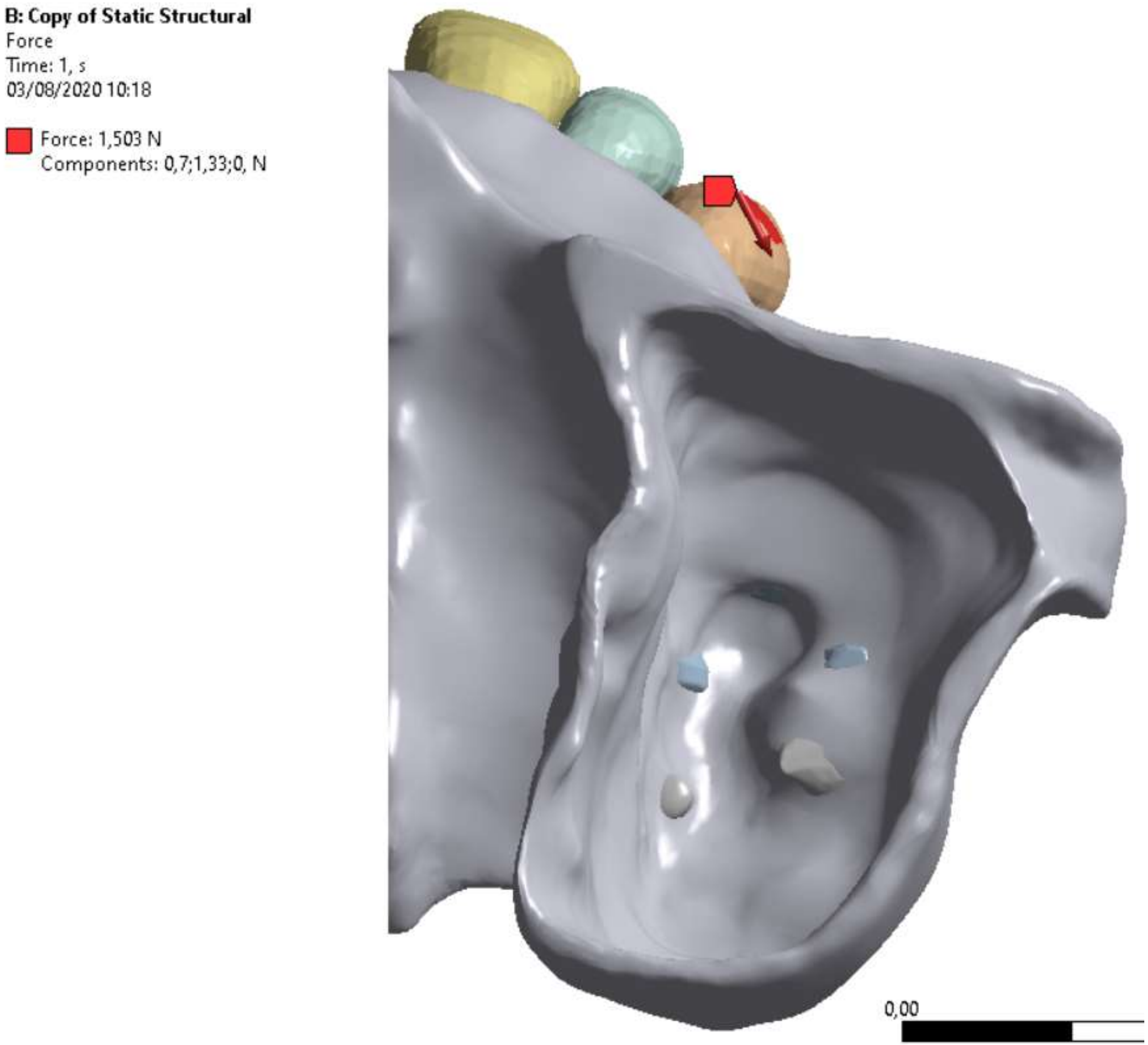
B: Copy of Static Structural
Force
Time: 1, s
03/08/2020 10:18
Force: 1,503 N
Components: 0,7;1,33;0, N
0,00

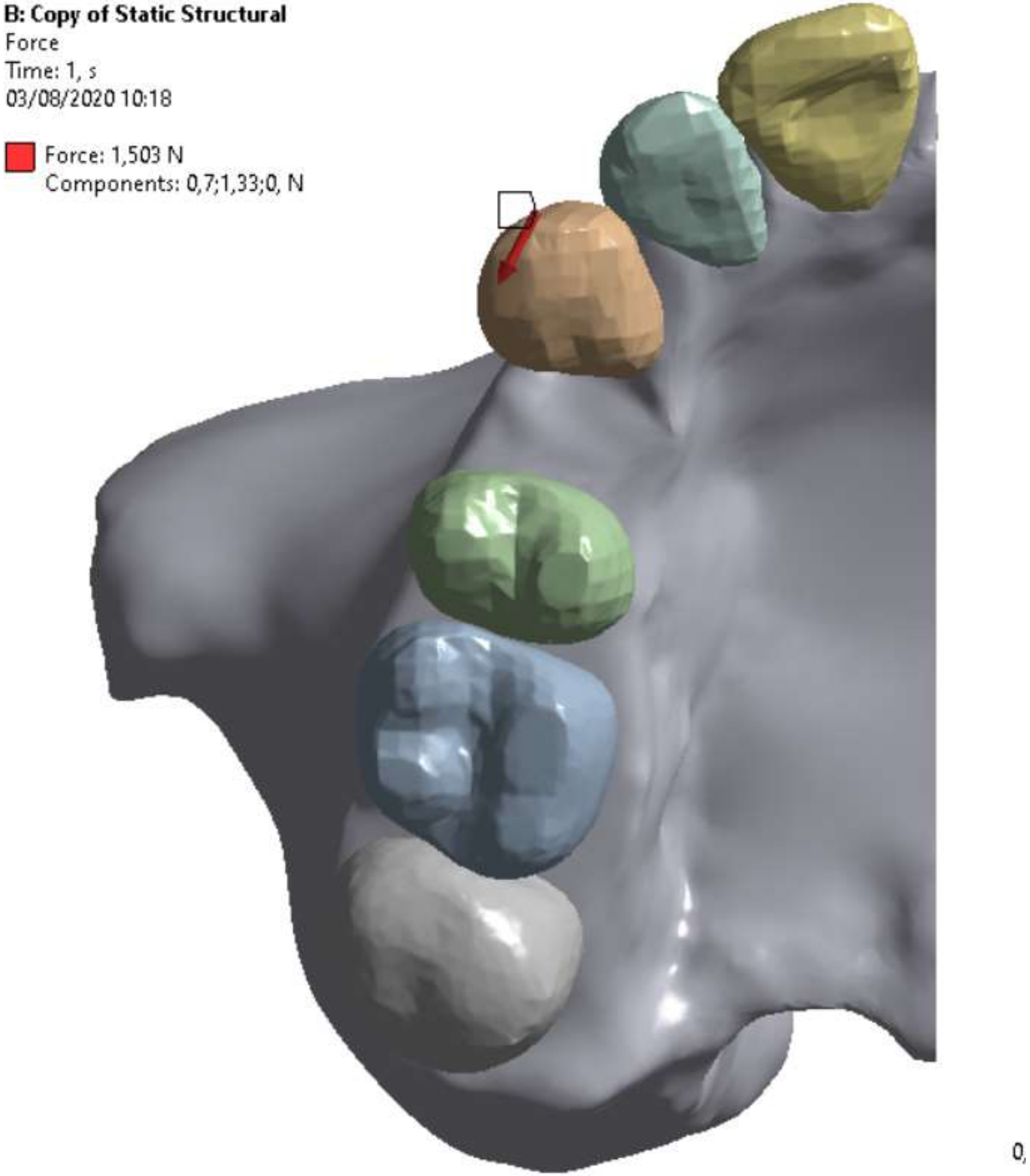
B: Copy of Static Structural
Force
Time: 1, s
03/08/2020 10:18
Force: 1,503 N
Components: 0,7;1,33;0, N
0,

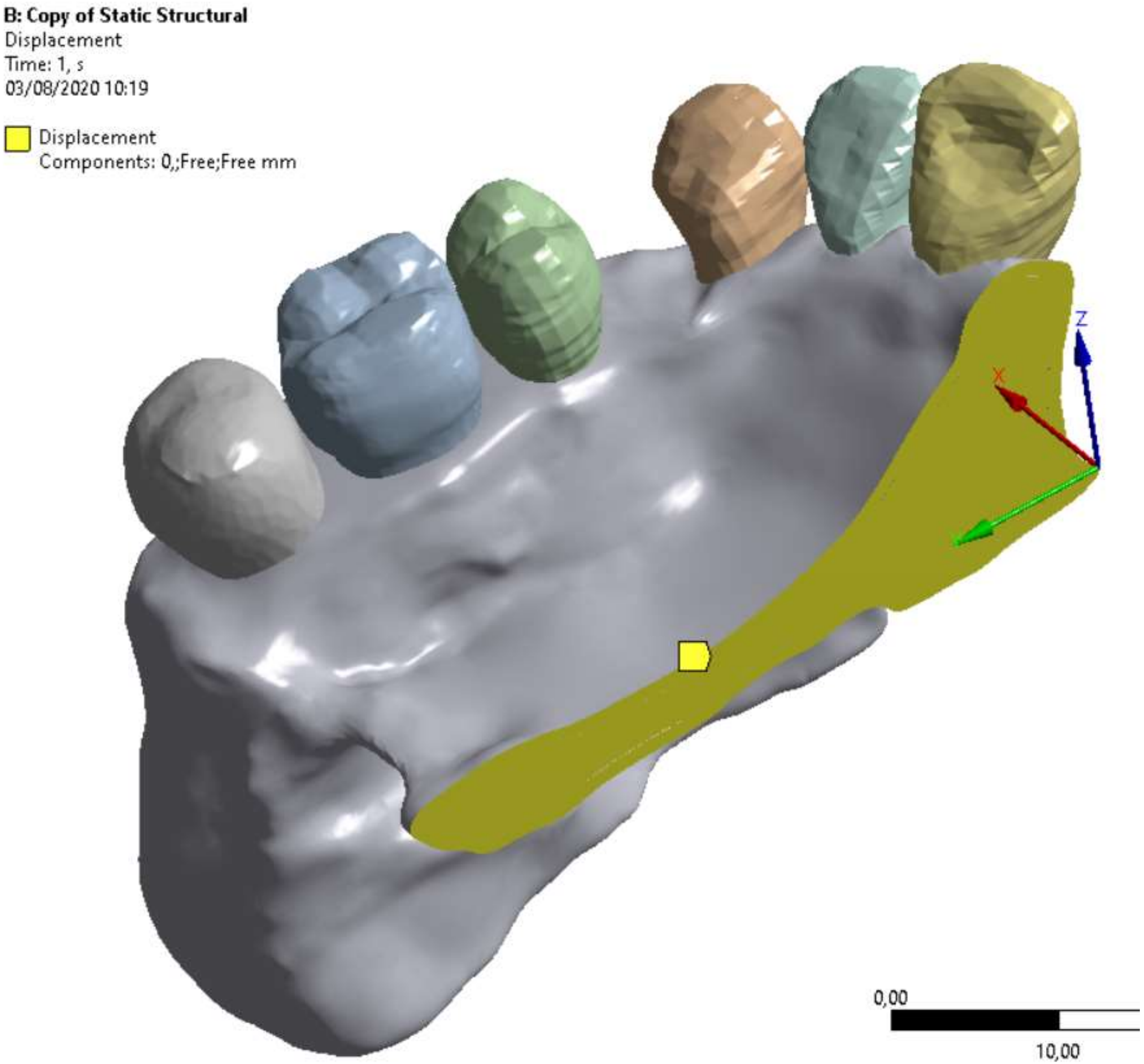
B: Copy of Static Structural
Displacement
Time: 1, s
03/08/2020 10:19
Displacement
Components: 0,;Free;Free mm
Z
X
0,00
10,00

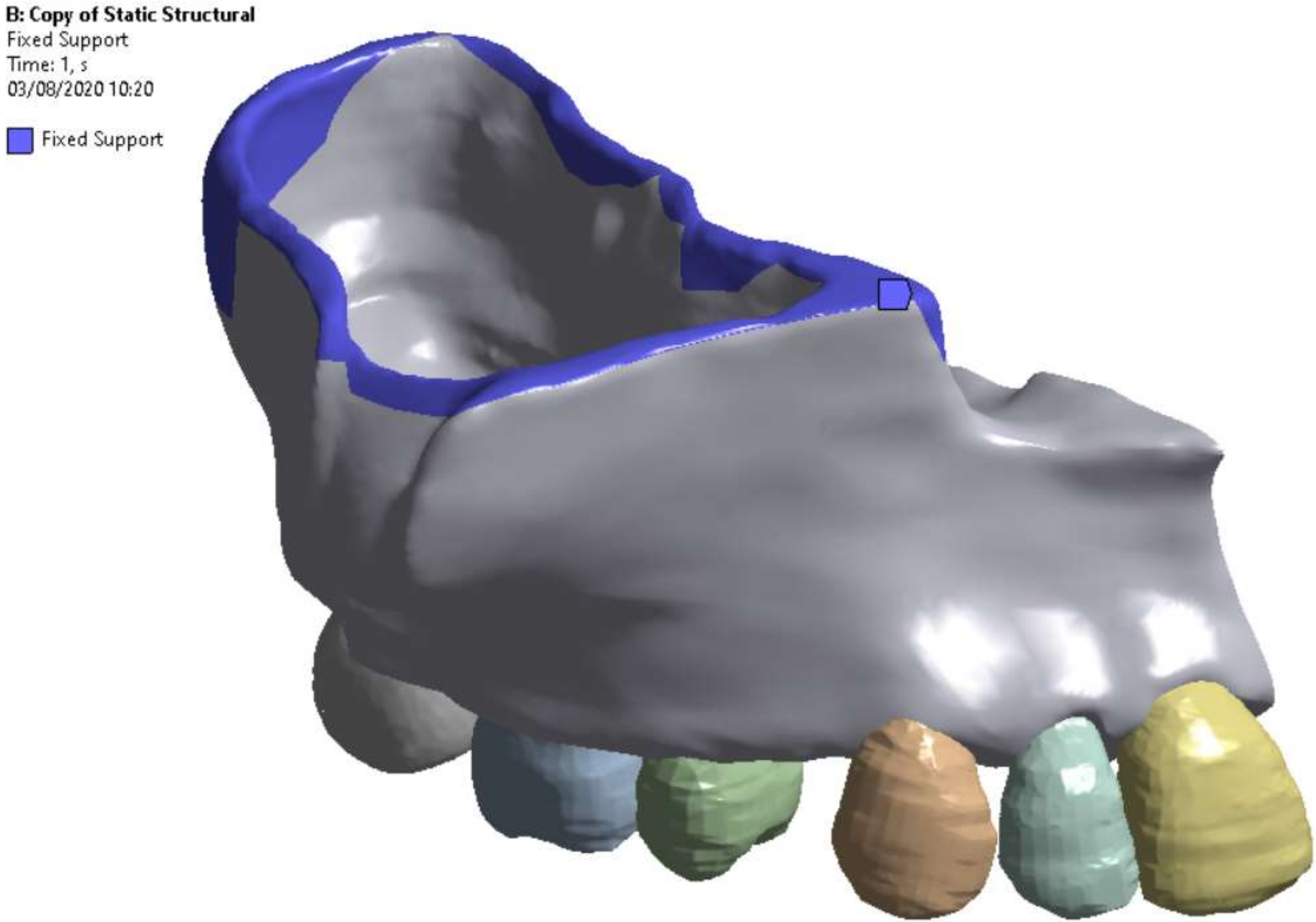
B: Copy of Static Structural
Fixed Support
Time: 1, s
03/08/2020 10:20
Fixed Support

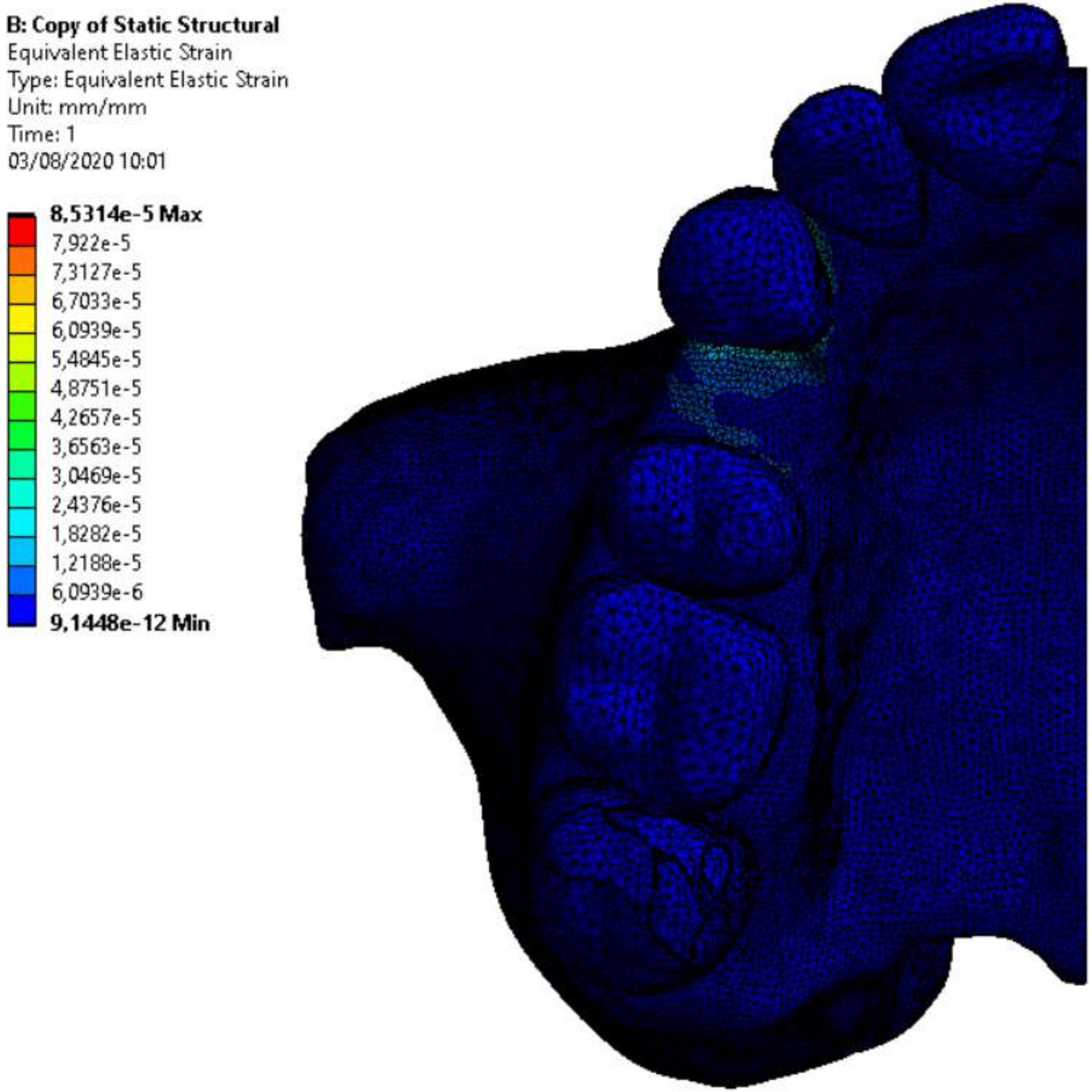
B: Copy of Static Structural
Equivalent Elastic Strain
Type: Equivalent Elastic Strain
Unit: mm/mm
Time: 1
03/08/2020 10:01
8,5314e-5 Max
7,922e-5
7,3127e-5
6,7033e-5
6,0939e-5
5,4845e-5
4,8751e-5
4,2657e-5
3,6563e-5
3,0469e-5
2,4376e-5
1,8282e-5
1,2188e-5
6,0939e-6
9,1448e-12 Min

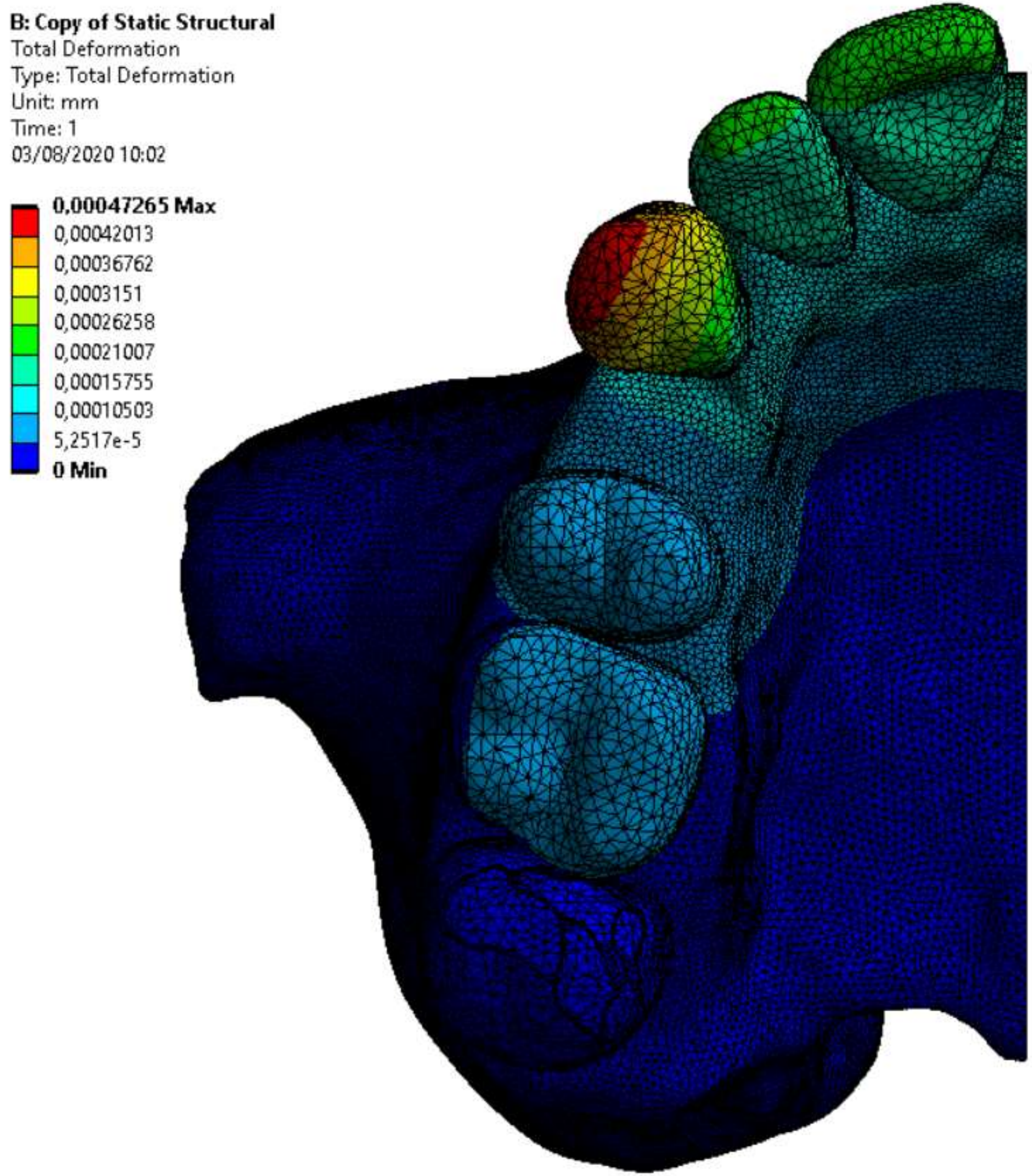
B: Copy of Static Structural
Total Deformation
Type: Total Deformation
Unit: mm
Time: 1
03/08/2020 10:02
0,00047265 Max
0,00042013
0,00036762
0,0003151
0,00026258
0,00021007
0,00015755
0,00010503
5,2517e-5
0 Min

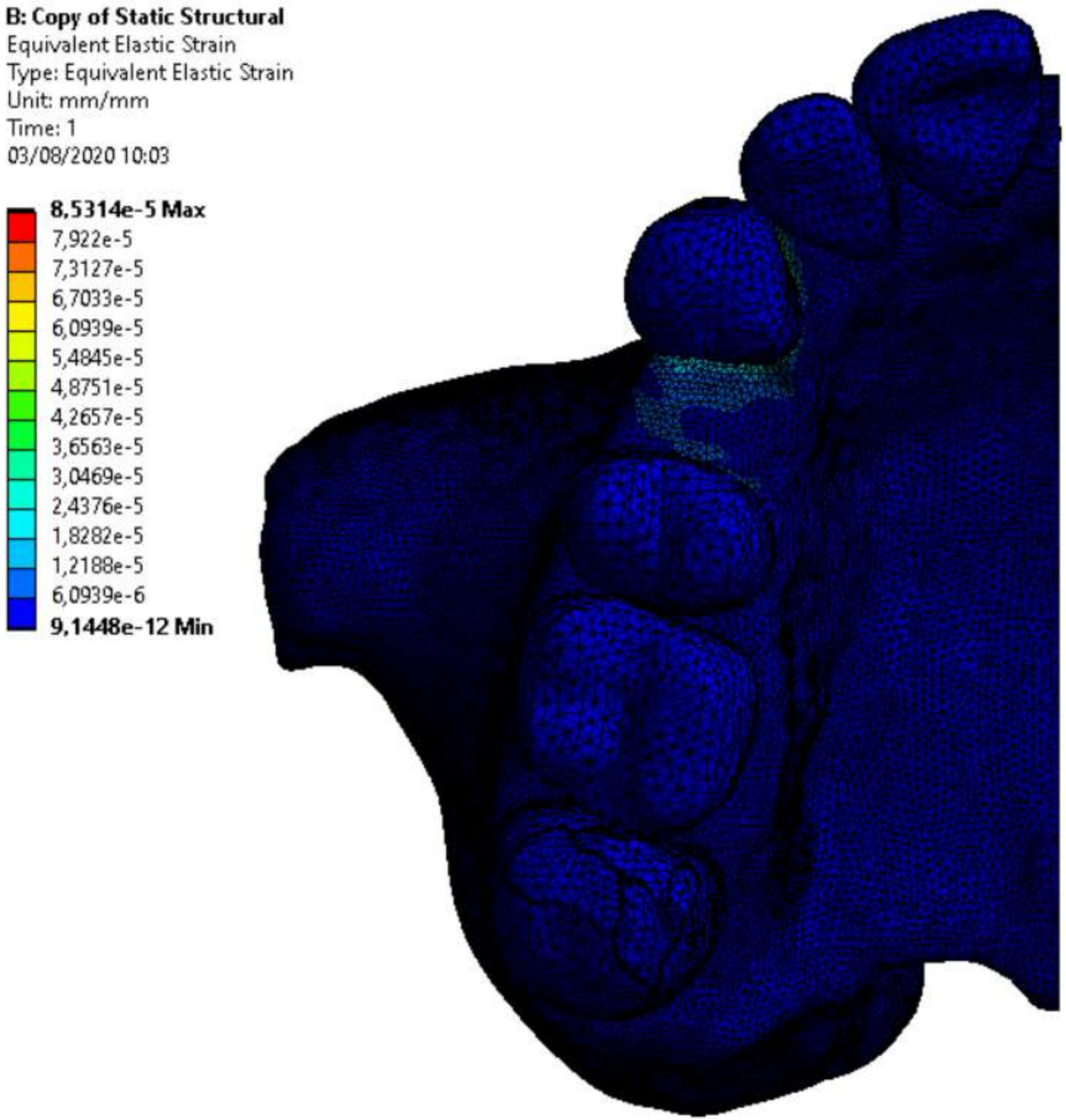
B: Copy of Static Structural
Equivalent Elastic Strain
Type: Equivalent Elastic Strain
Unit: mm/mm
Time: 1
03/08/2020 10:03
8,5314e-5 Max
7,922e-5
7,3127e-5
6,7033e-5
6,0939e-5
5,4845e-5
4,8751e-5
4,2657e-5
3,6563e-5
3,0469e-5
2,4376e-5
1,8282e-5
1,2188e-5
6,0939e-6
9,1448e-12 Min

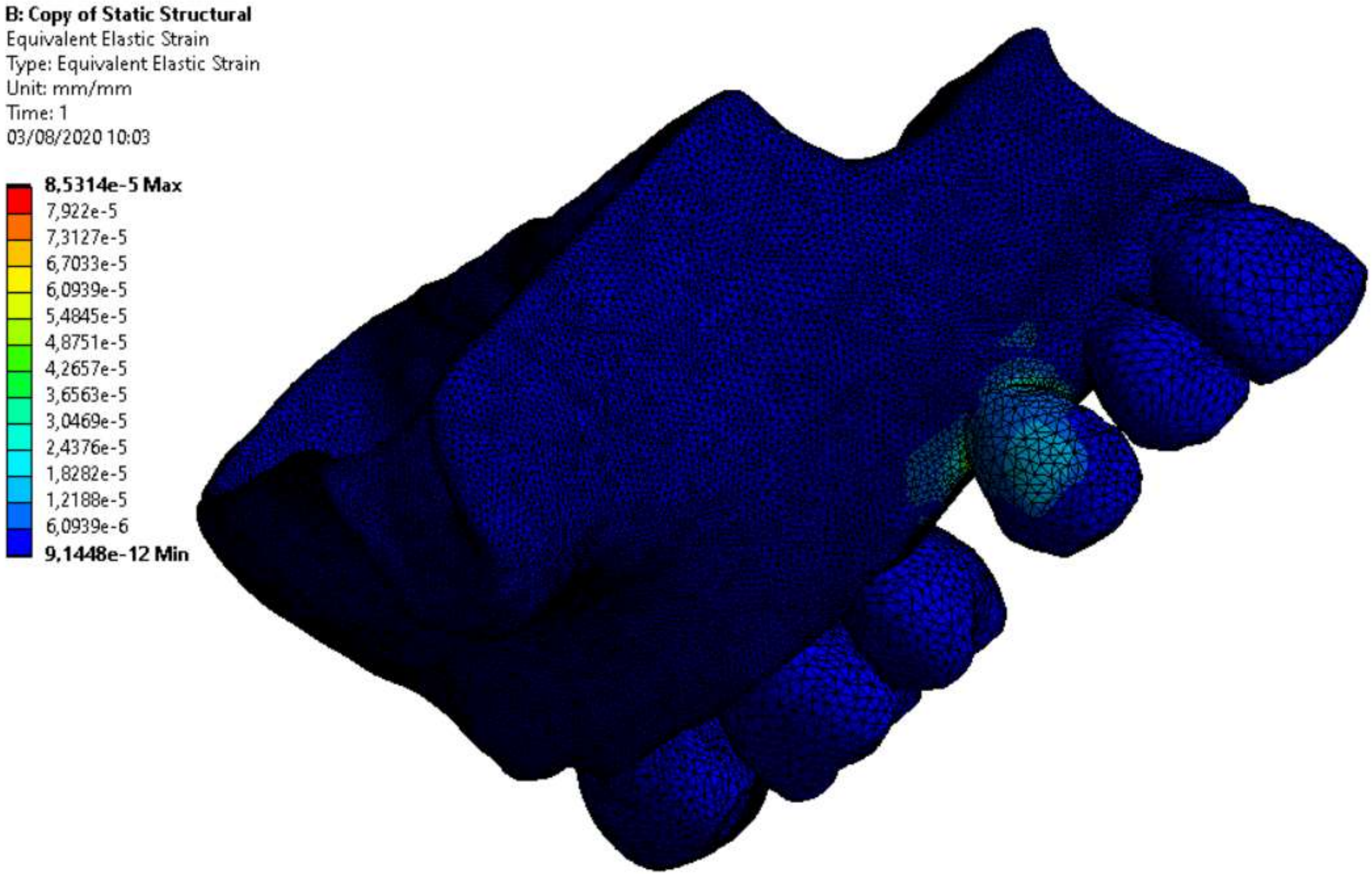
B: Copy of Static Structural
Equivalent Elastic Strain
Type: Equivalent Elastic Strain
Unit: mm/mm
Time: 1
03/08/2020 10:03
8,5314e-5 Max
7,922e-5
7,3127e-5
6,7033e-5
6,0939e-5
5,4845e-5
4,8751e-5
4,2657e-5
3,6563e-5
3,0469e-5
2,4376e-5
1,8282e-5
1,2188e-5
6,0939e-6
9,1448e-12 Min

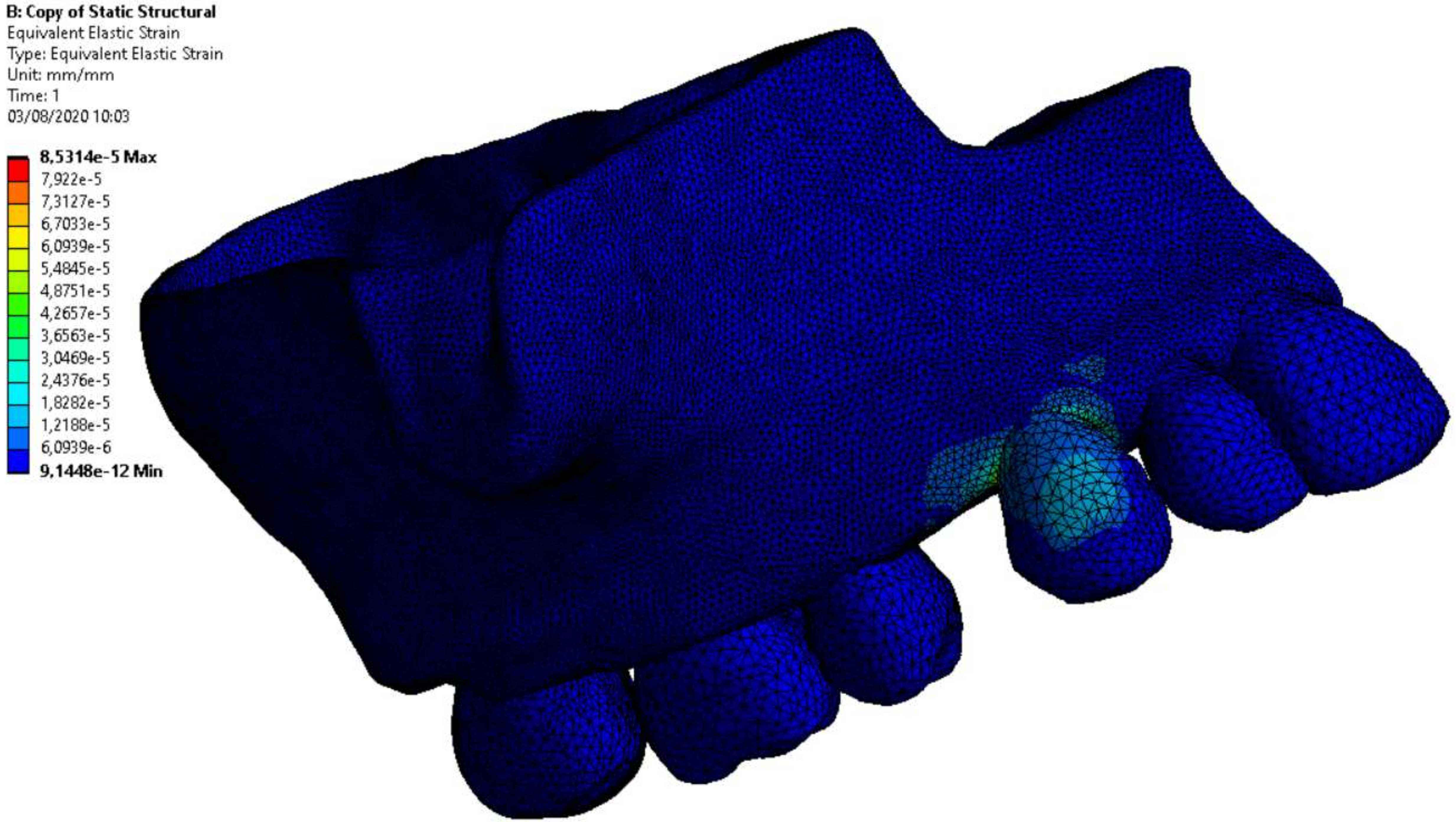

B: Copy of Static Structural
Equivalent Elastic Strain
Type: Equivalent Elastic Strain
Unit: mm/mm
Time: 1
03/08/2020 10:03
8,5314e-5 Max
7,922e-5
7,3127e-5
6,7033e-5
6,0939e-5
5,4845e-5
4,8751e-5
4,2657e-5
3,6563e-5
3,0469e-5
2,4376e-5
1,8282e-5
1,2188e-5
6,0939e-6
9,1448e-12 Min

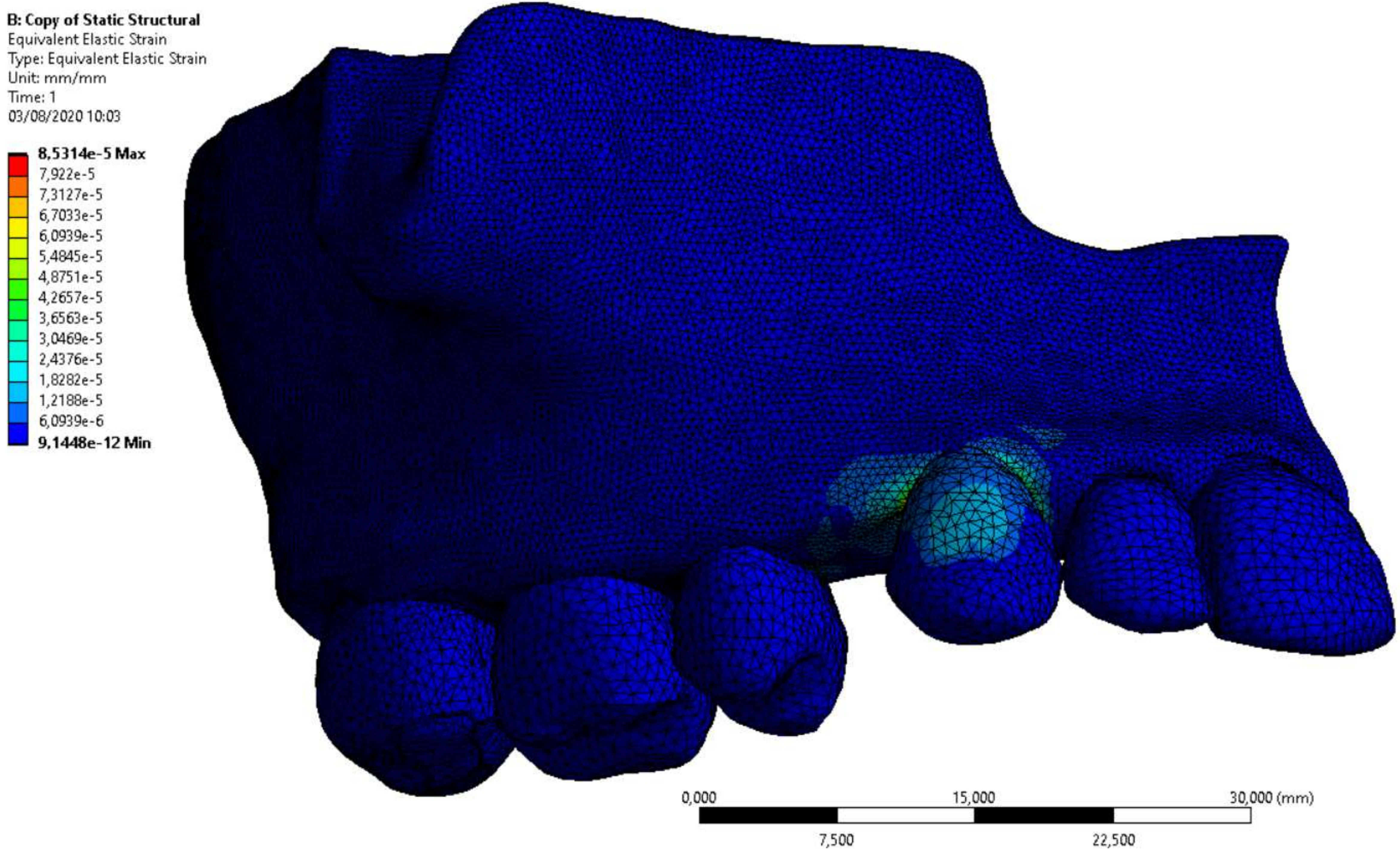
B: Copy of Static Structural
Equivalent Elastic Strain
Type: Equivalent Elastic Strain
Unit: mm/mm
Time: 1
03/08/2020 10:03
8,5314e-5 Max
7,922e-5
7,3127e-5
6,7033e-5
6,0939e-5
5,4845e-5
4,8751e-5
4,2657e-5
3,6563e-5
3,0469e-5
2,4376e-5
1,8282e-5
1,2188e-5
6,0939e-6
9,1448e-12 Min
0,000
15,000
30,000 (mm)
7,500
22,500

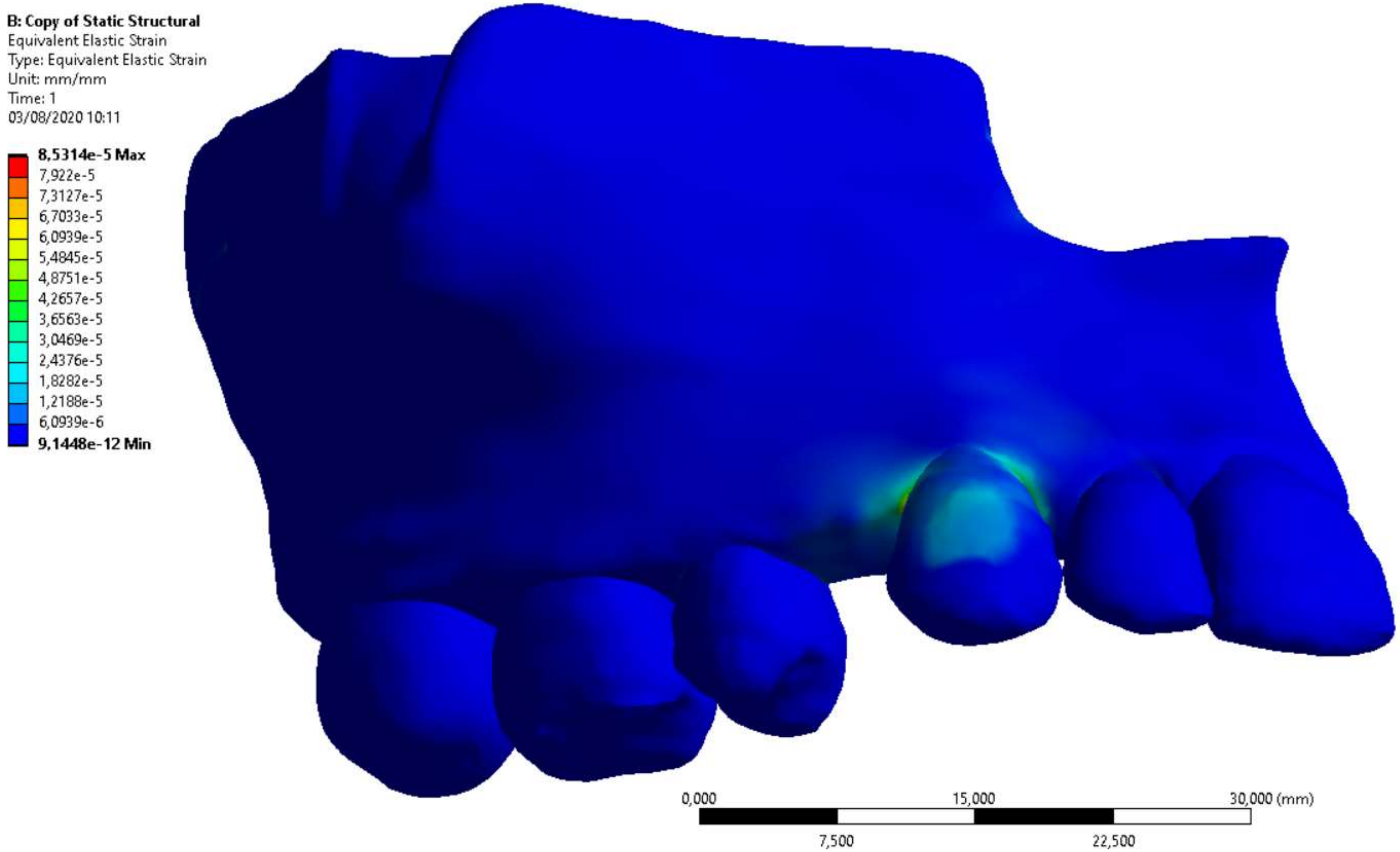
B: Copy of Static Structural
Equivalent Elastic Strain
Type: Equivalent Elastic Strain
Unit: mm/mm
Time: 1
03/08/2020 10:11
8,5314e-5 Max
7,922e-5
7,3127e-5
6,7033e-5
6,0939e-5
5,4845e-5
4,8751e-5
4,2657e-5
3,6563e-5
3,0469e-5
2,4376e-5
1,8282e-5
1,2188e-5
6,0939e-6
9,1448e-12 Min
0,000
15,000
30,000 (mm)
7,500
22,500

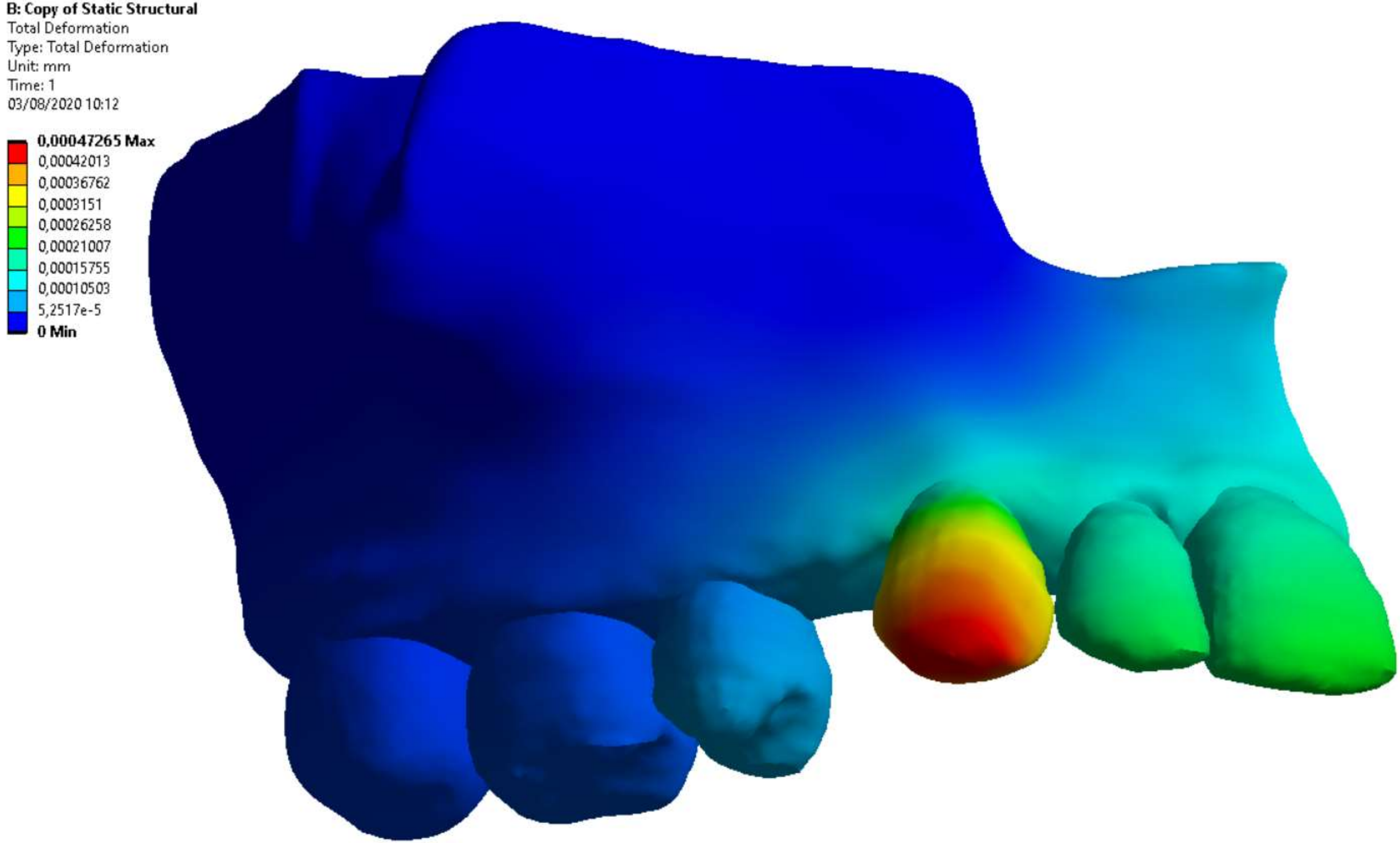
B: Copy of Static Structural
Total Deformation
Type: Total Deformation
Unit: mm
Time: 1
03/08/2020 10:12
0,00047265 Max
0,00042013
0,00036762
0,0003151
0,00026258
0,00021007
0,00015755
0,00010503
5,2517e-5
0 Min

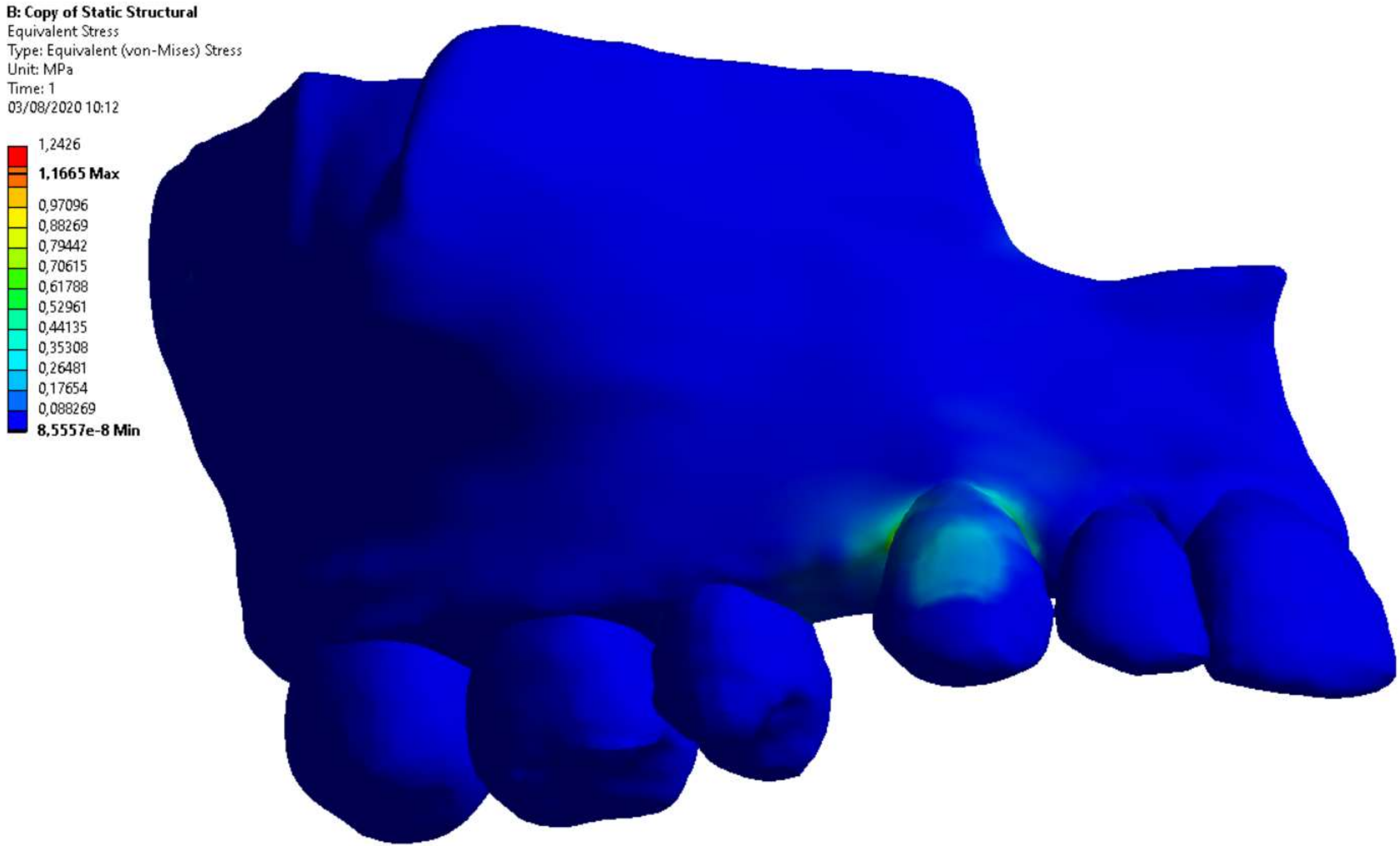
B: Copy of Static Structural
Equivalent Stress
Type: Equivalent (von-Mises) Stress
Unit: MPa
Time: 1
03/08/2020 10:12
1,2426
1,1665 Max
0,97096
0,88269
0,79442
0,70615
0,61788
0,52961
0,44135
0,35308
0,26481
0,17654
0,088269
8,5557e-8 Min

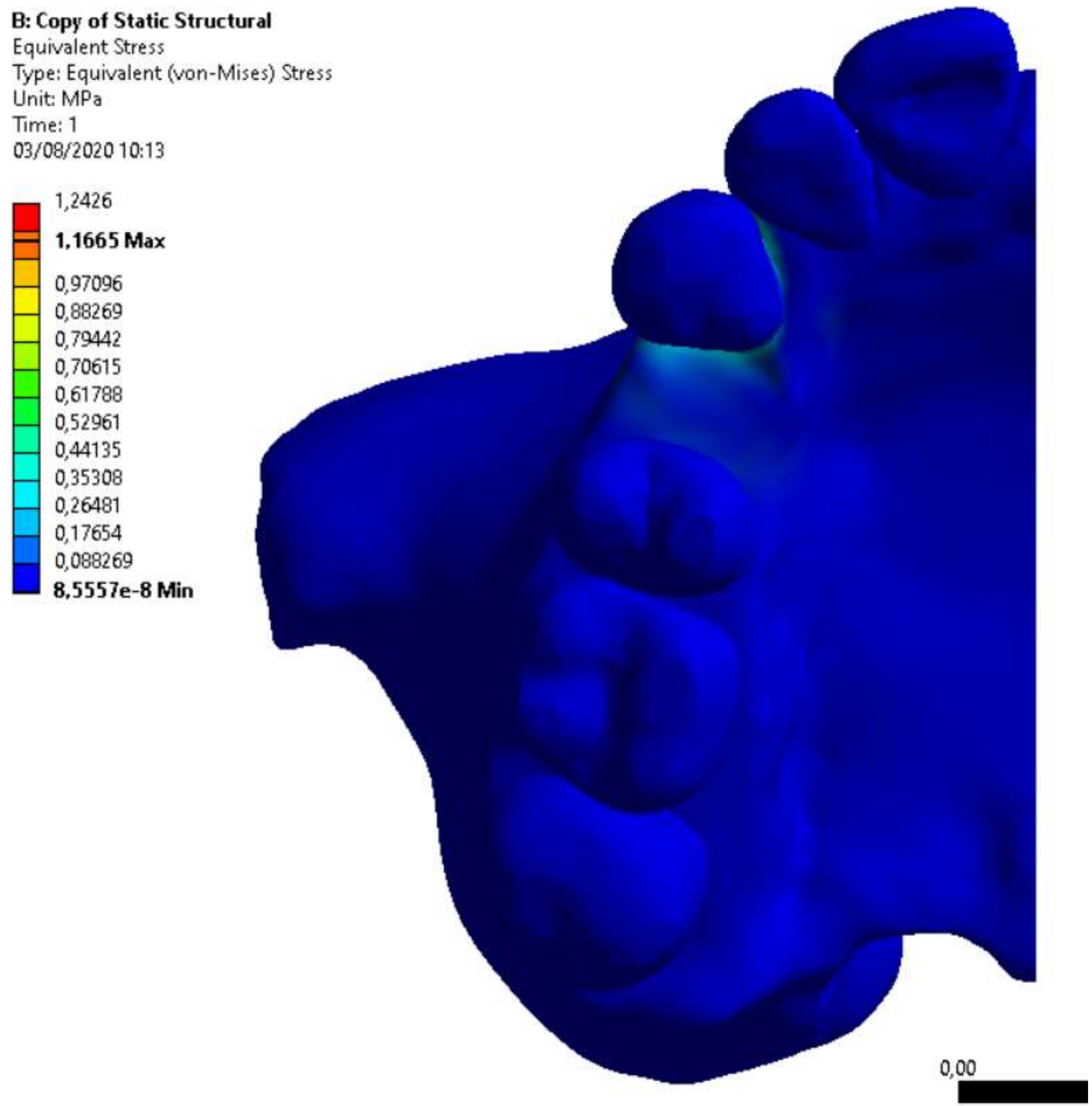
B: Copy of Static Structural
Equivalent Stress
Type: Equivalent (von-Mises) Stress
Unit: MPa
Time: 1
03/08/2020 10:13
1,2426
1,1665 Max
0,97096
0,88269
0,79442
0,70615
0,61788
0,52961
0,44135
0,35308
0,26481
0,17654
0,088269
8,5557e-8 Min
0,00

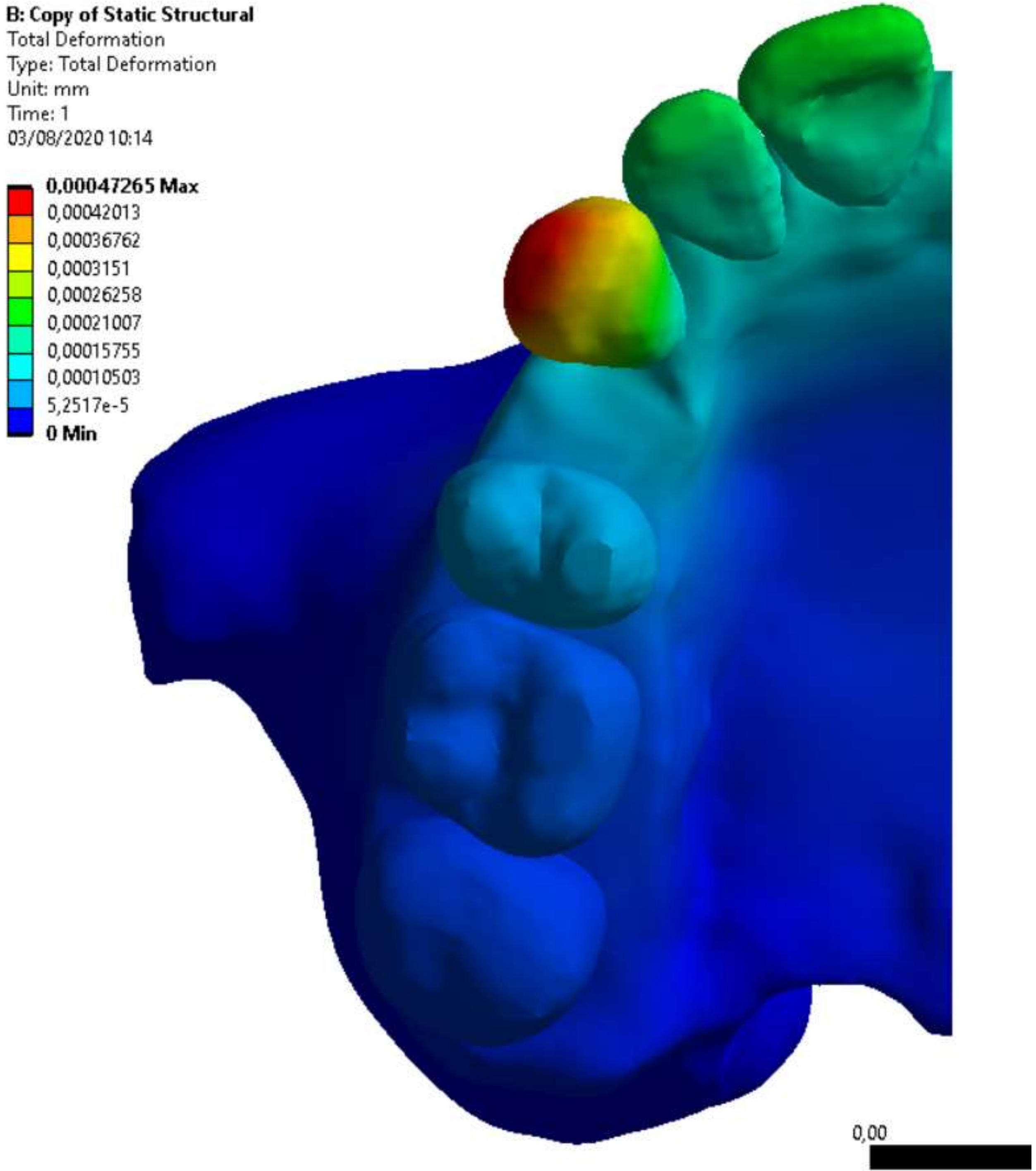
B: Copy of Static Structural
Total Deformation
Type: Total Deformation
Unit: mm
Time: 1
03/08/2020 10:14
0,00047265 Max
0,00042013
0,00036762
0,0003151
0,00026258
0,00021007
0,00015755
0,00010503
5,2517e-5
0 Min
0,00

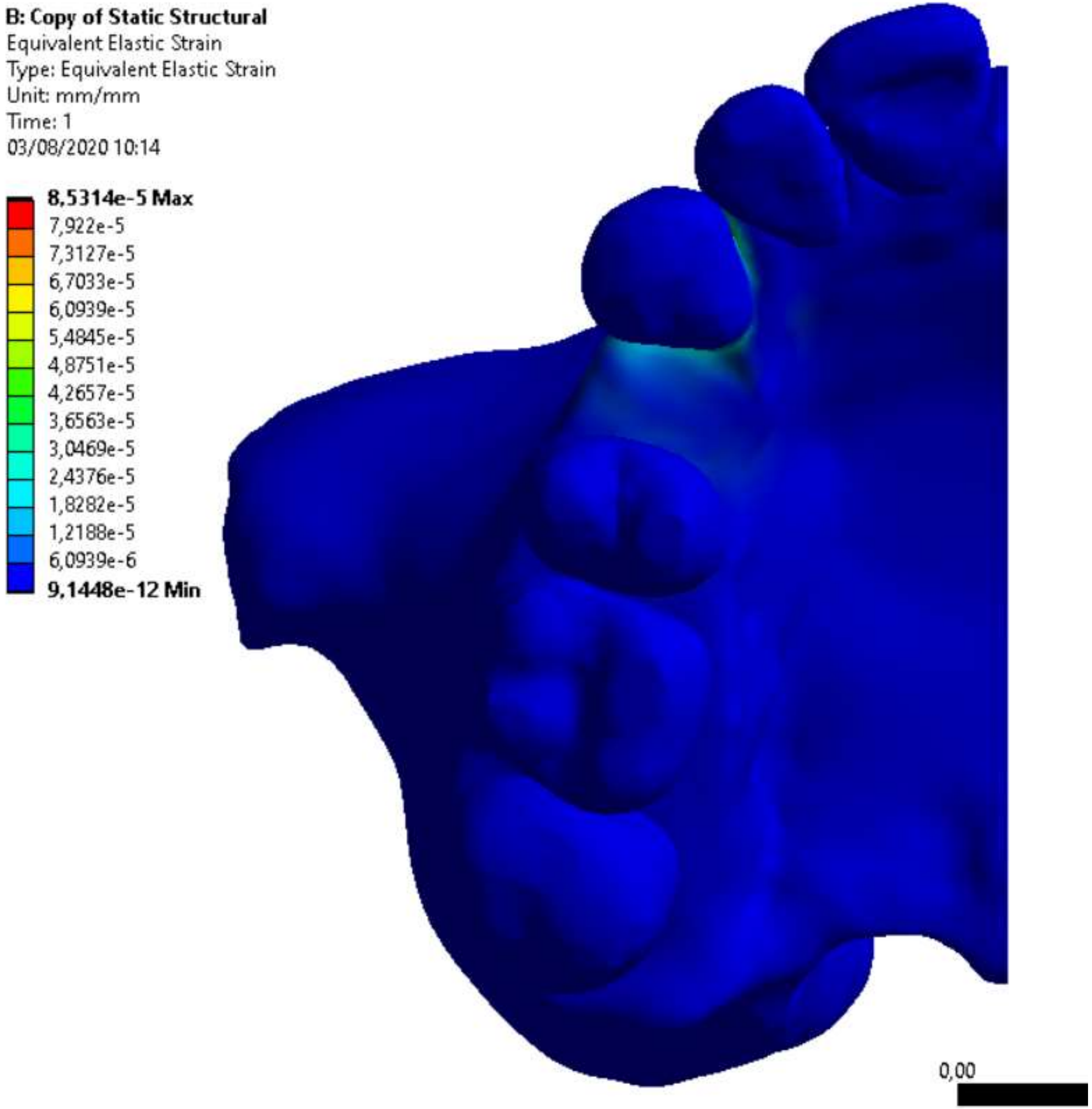
B: Copy of Static Structural
Equivalent Elastic Strain
Type: Equivalent Elastic Strain
Unit: mm/mm
Time: 1
03/08/2020 10:14
8,5314e-5 Max
7,922e-5
7,3127e-5
6,7033e-5
6,0939e-5
5,4845e-5
4,8751e-5
4,2657e-5
3,6563e-5
3,0469e-5
2,4376e-5
1,8282e-5
1,2188e-5
6,0939e-6
9,1448e-12 Min
0,00

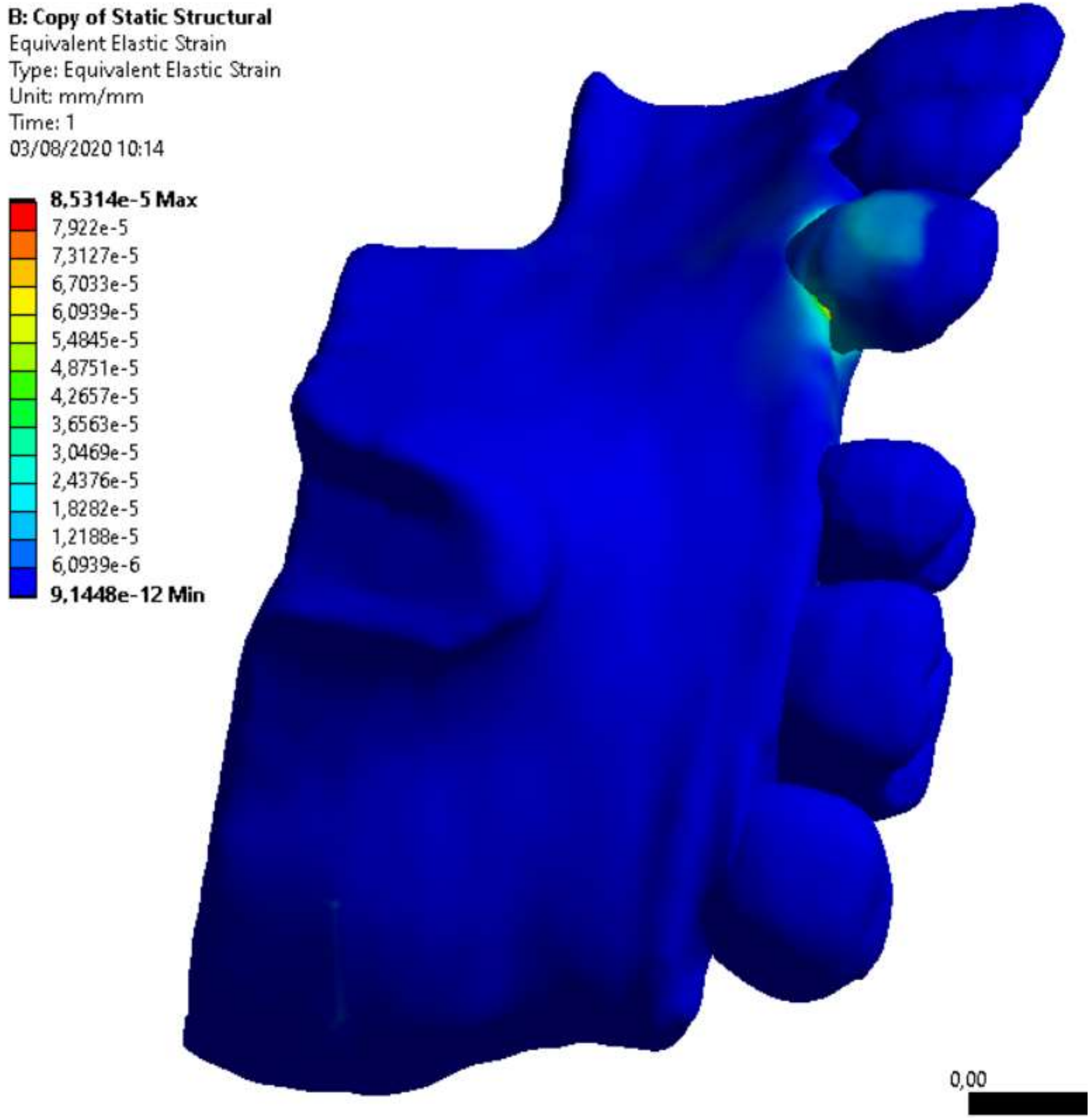
B: Copy of Static Structural
Equivalent Elastic Strain
Type: Equivalent Elastic Strain
Unit: mm/mm
Time: 1
03/08/2020 10:14
8,5314e-5 Max
7,922e-5
7,3127e-5
6,7033e-5
6,0939e-5
5,4845e-5
4,8751e-5
4,2657e-5
3,6563e-5
3,0469e-5
2,4376e-5
1,8282e-5
1,2188e-5
6,0939e-6
9,1448e-12 Min
0,00

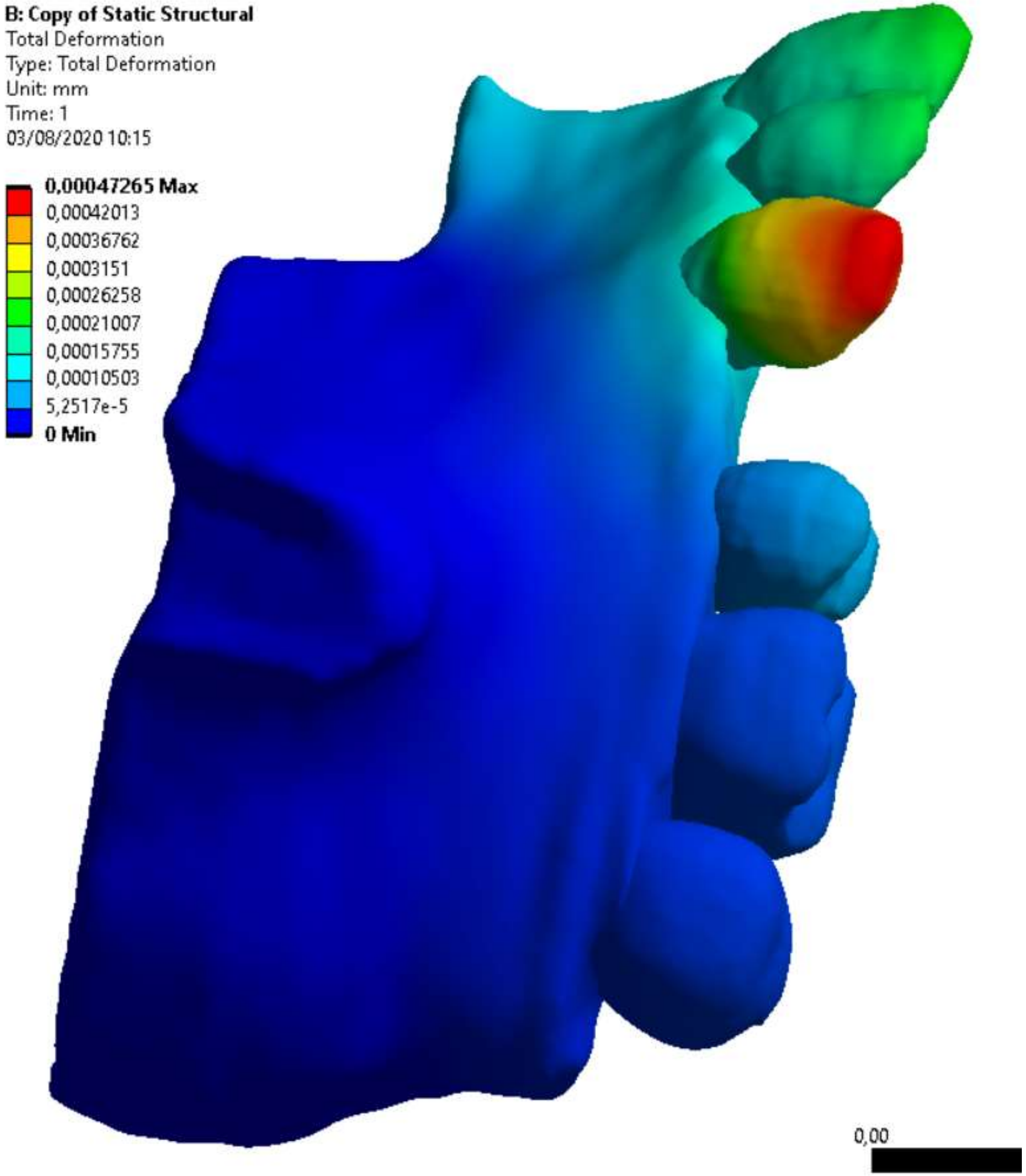
B: Copy of Static Structural
Total Deformation
Type: Total Deformation
Unit: mm
Time: 1
03/08/2020 10:15
0,00047265 Max
0,00042013
0,00036762
0,0003151
0,00026258
0,00021007
0,00015755
0,00010503
5,2517e-5
0 Min
0,00

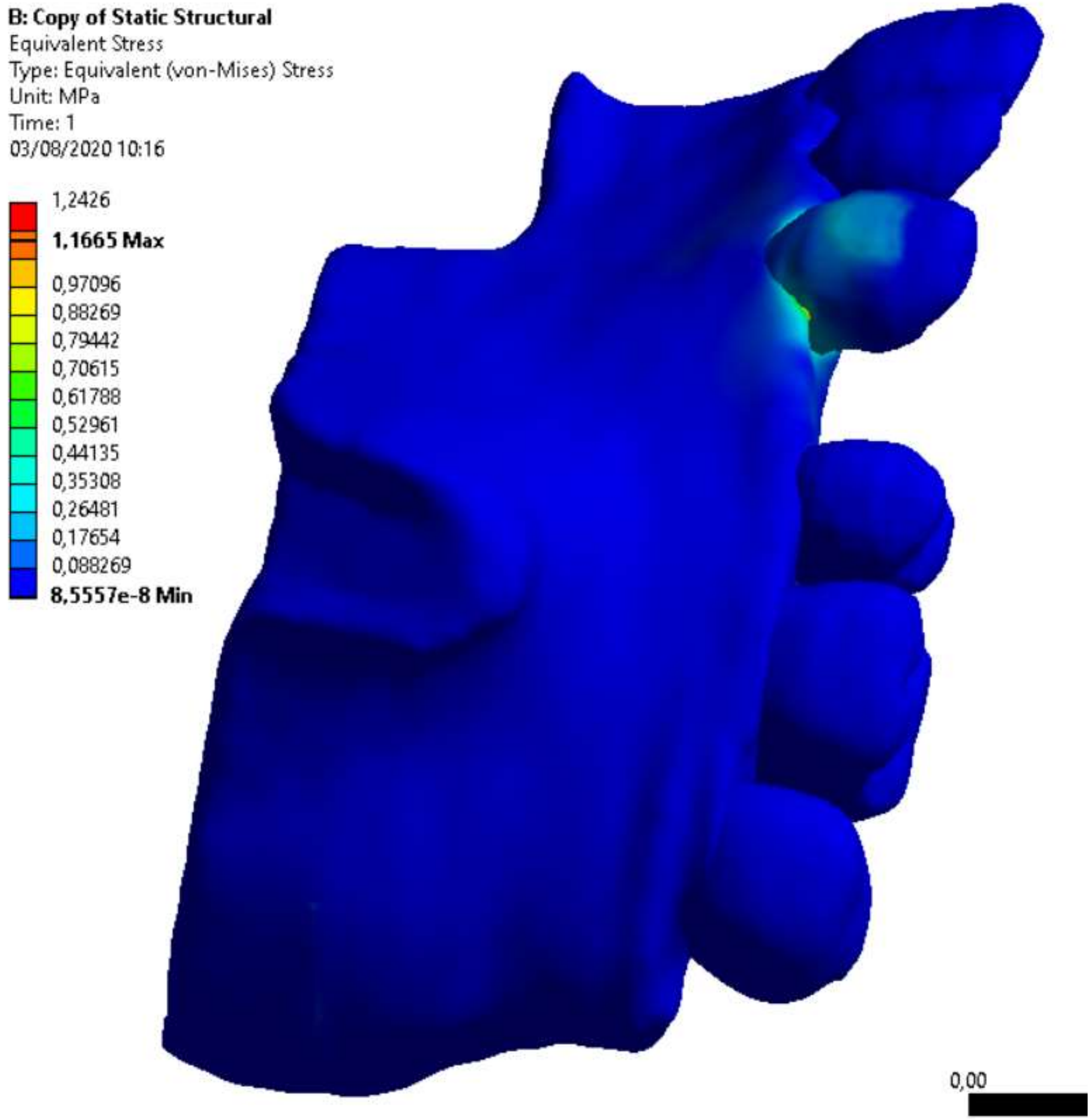
B: Copy of Static Structural
Equivalent Stress
Type: Equivalent (von-Mises) Stress
Unit: MPa
Time: 1
03/08/2020 10:16
1,2426
1,1665 Max
0,97096
0,88269
0,79442
0,70615
0,61788
0,52961
0,44135
0,35308
0,26481
0,17654
0,088269
8,5557e-8 Min
0,00
