# Supplementary material for: Micro-osteoperforation for enhancement of orthodontic movement: A mechanical analysis using the finite element method
Source: PLoS One. 2024 Aug 19;19(8):e0308739. doi: 10.1371/journal.pone.0308739 (PMC11332926; doi:10.1371/journal.pone.0308739)
Supplement: S2 File — (PDF) [file pone.0308739.s002.pdf]

Study 2 with perforations

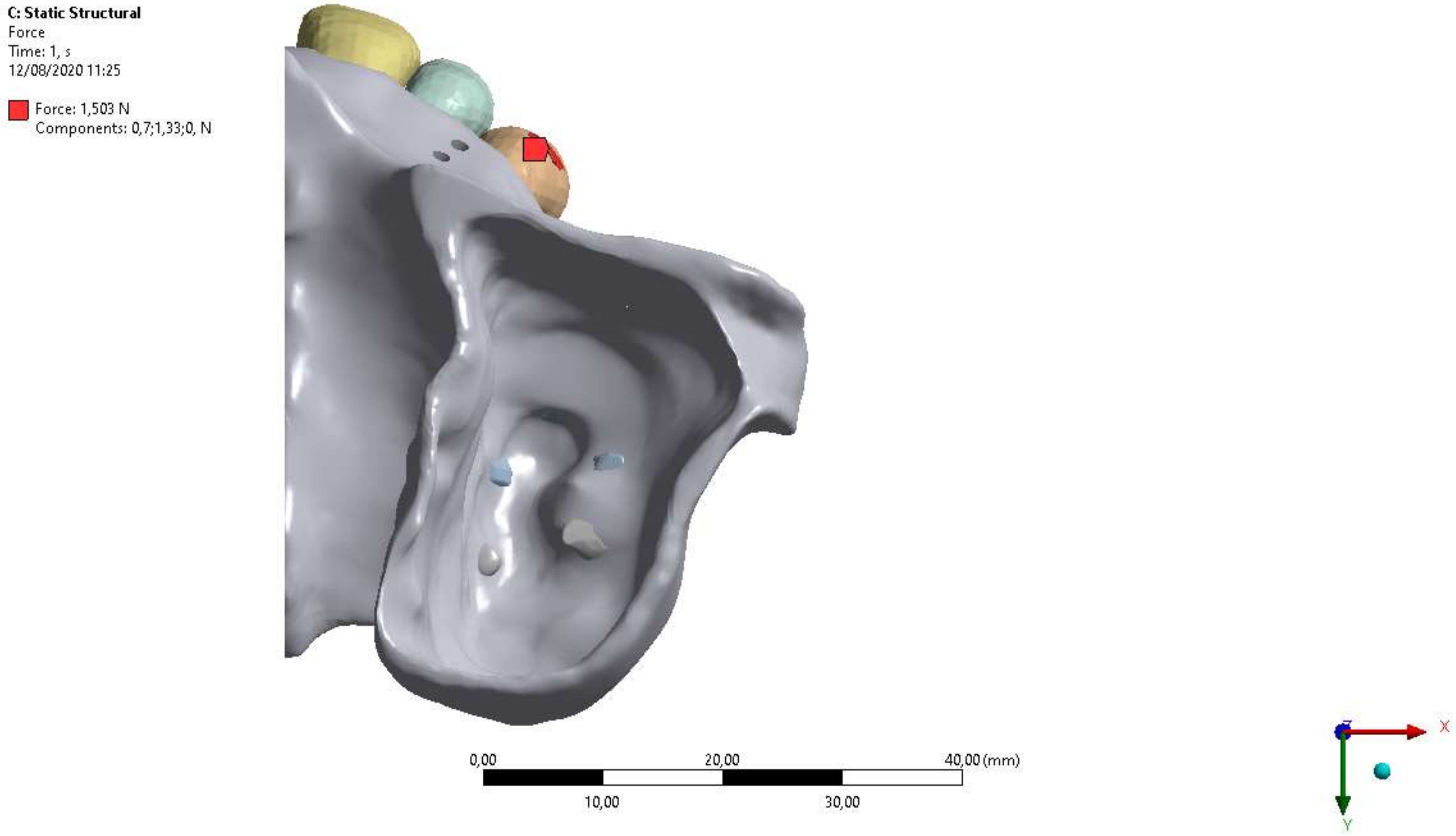
C: Static Structural
Force
Time: 1, s
12/08/2020 11:25
Force: 1,503 N
Components: 0,7;1,33;0, N
0,00
20,00
40,00 (mm)
10,00
30,00
X
Y

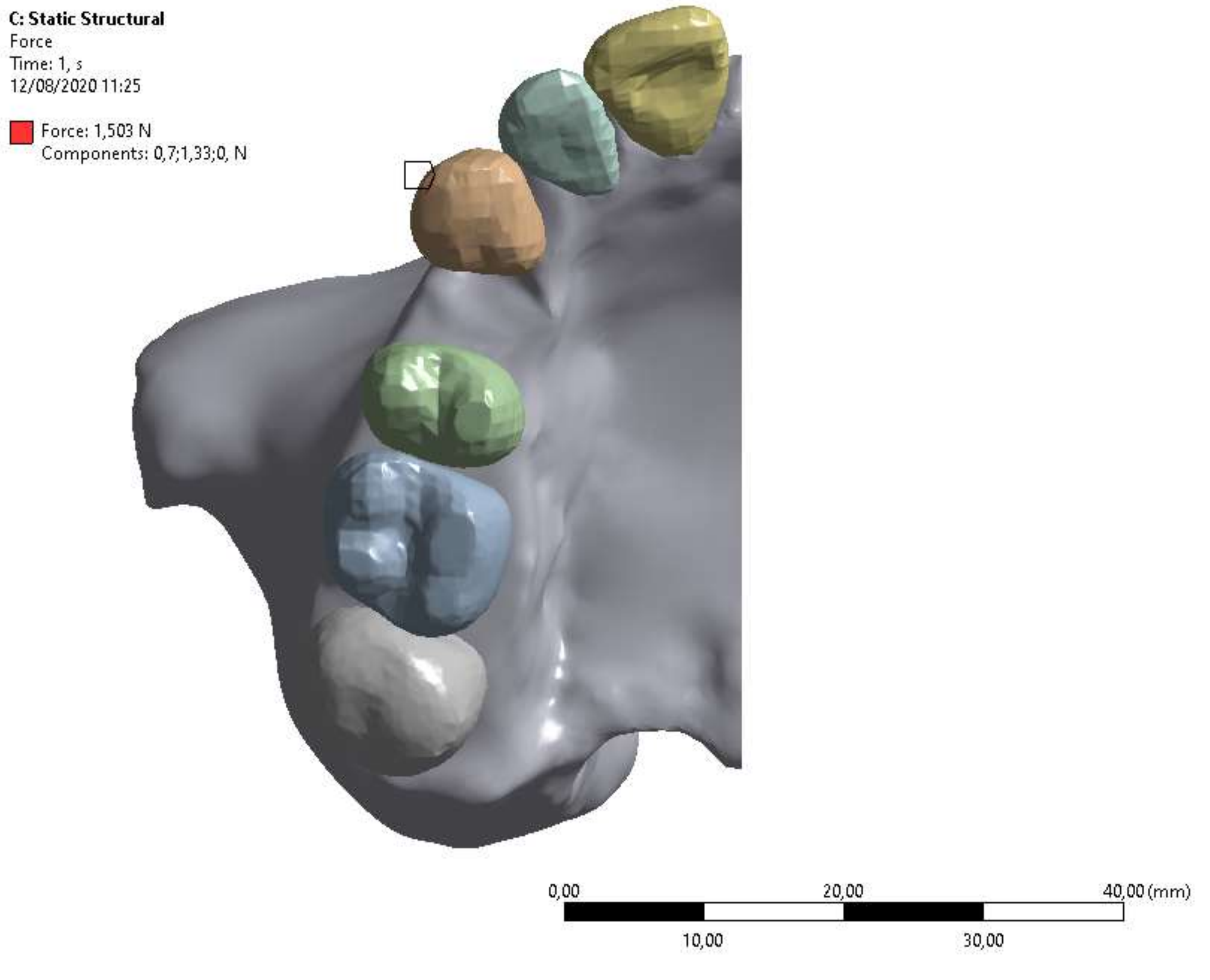

C: Static Structural
Force
Time: 1, s
12/08/2020 11:25
Force: 1,503 N
Components: 0,7;1,33;0, N
0,00
20,00
40,00 (mm)
10,00
30,00
X
Z
Y

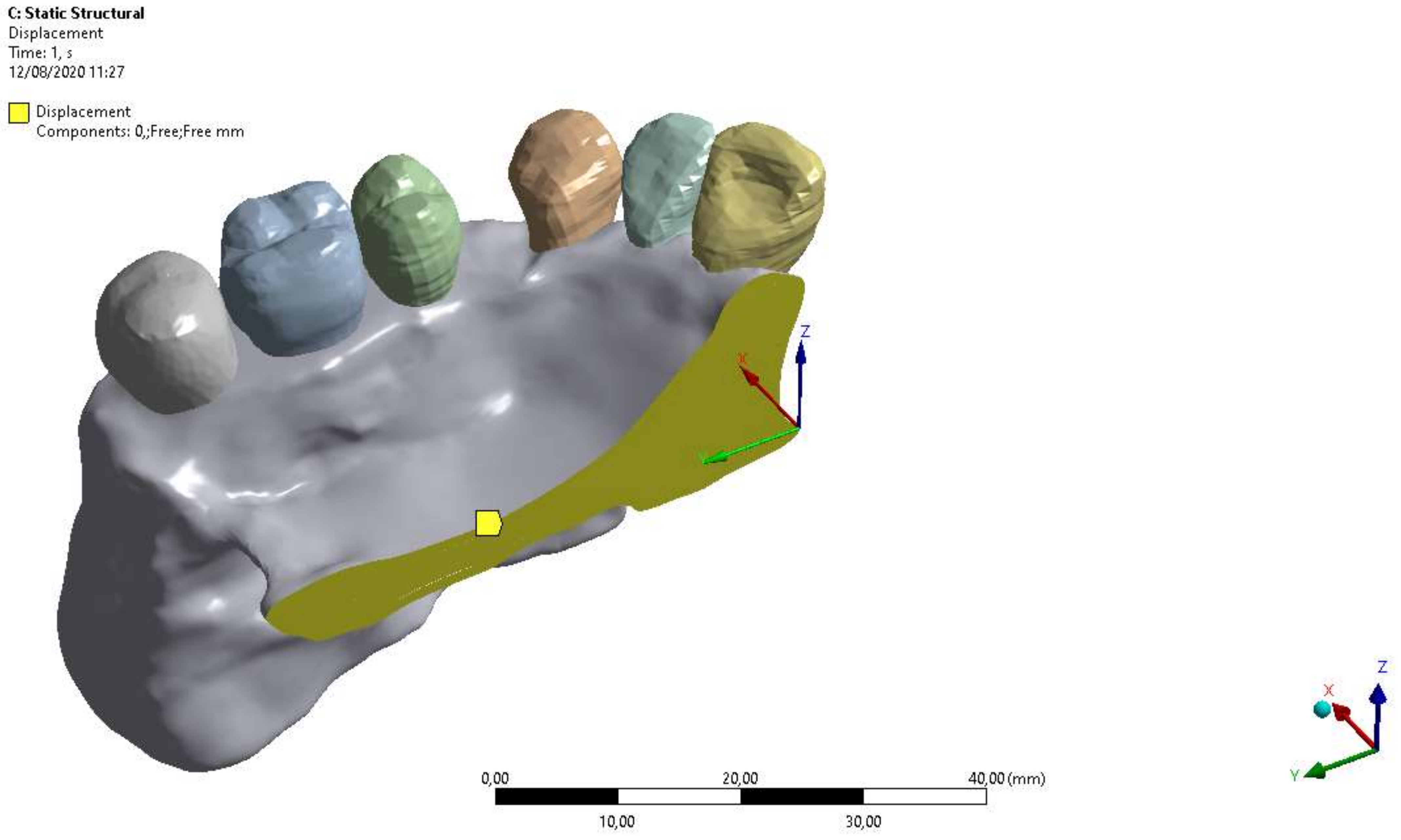
C: Static Structural
Displacement
Time: 1, s
12/08/2020 11:27
Displacement
Components: 0,;Free;Free mm
0,00
10,00
20,00
30,00
40,00 (mm)
X
Y
Z

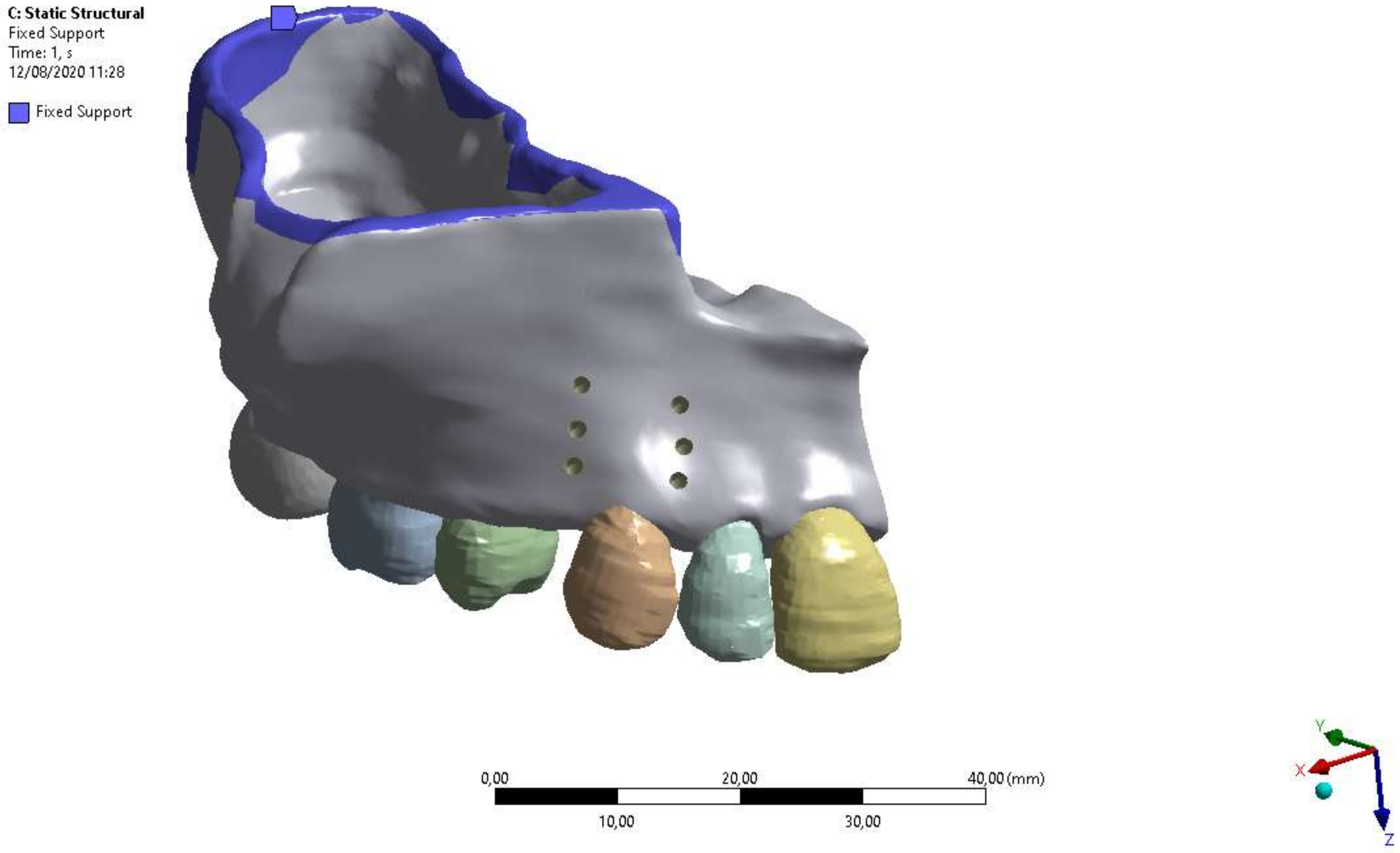
C: Static Structural
Fixed Support
Time: 1, s
12/08/2020 11:28
Fixed Support
0,00
20,00
40,00 (mm)
10,00
30,00
Y
X
Z

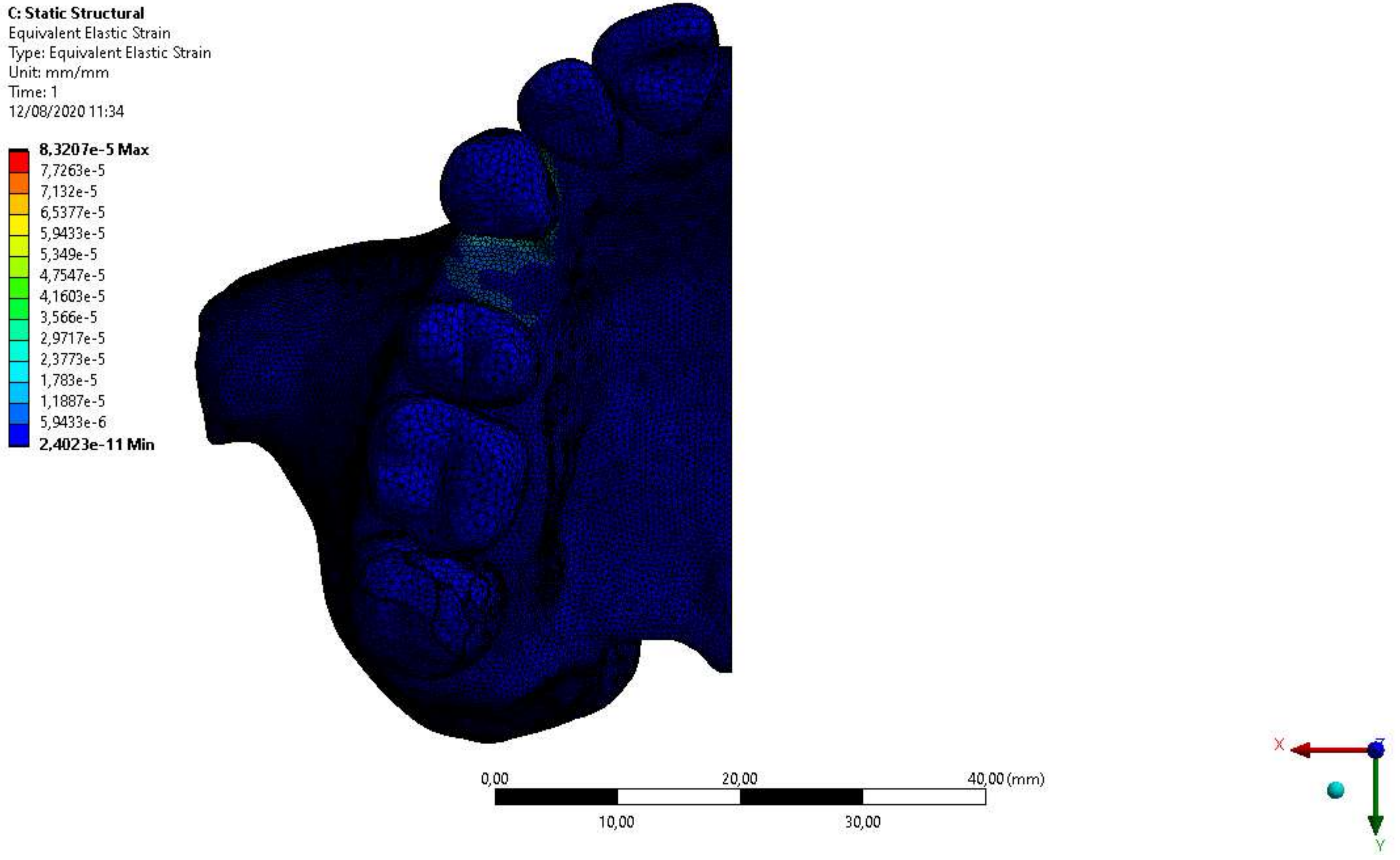
C: Static Structural
Equivalent Elastic Strain
Type: Equivalent Elastic Strain
Unit: mm/mm
Time: 1
12/08/2020 11:34
8,3207e-5 Max
7,7263e-5
7,132e-5
6,5377e-5
5,9433e-5
5,349e-5
4,7547e-5
4,1603e-5
3,566e-5
2,9717e-5
2,3773e-5
1,783e-5
1,1887e-5
5,9433e-6
2,4023e-11 Min
0,00
20,00
40,00 (mm)
10,00
30,00
X
Y
Z

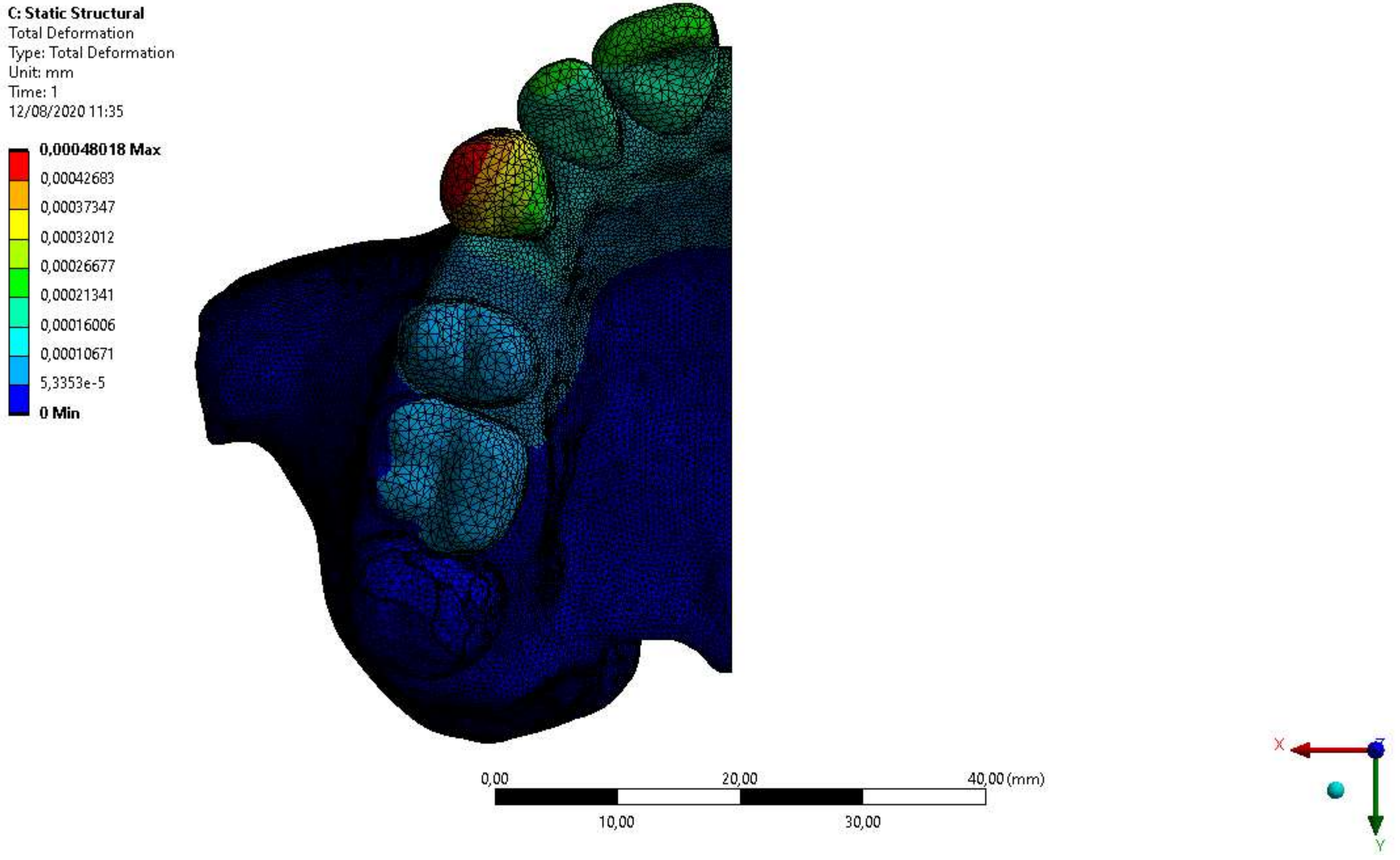
C: Static Structural
Total Deformation
Type: Total Deformation
Unit: mm
Time: 1
12/08/2020 11:35
0,00048018 Max
0,00042683
0,00037347
0,00032012
0,00026677
0,00021341
0,00016006
0,00010671
5,3353e-5
0 Min
0,00
20,00
40,00 (mm)
10,00
30,00
X
Y

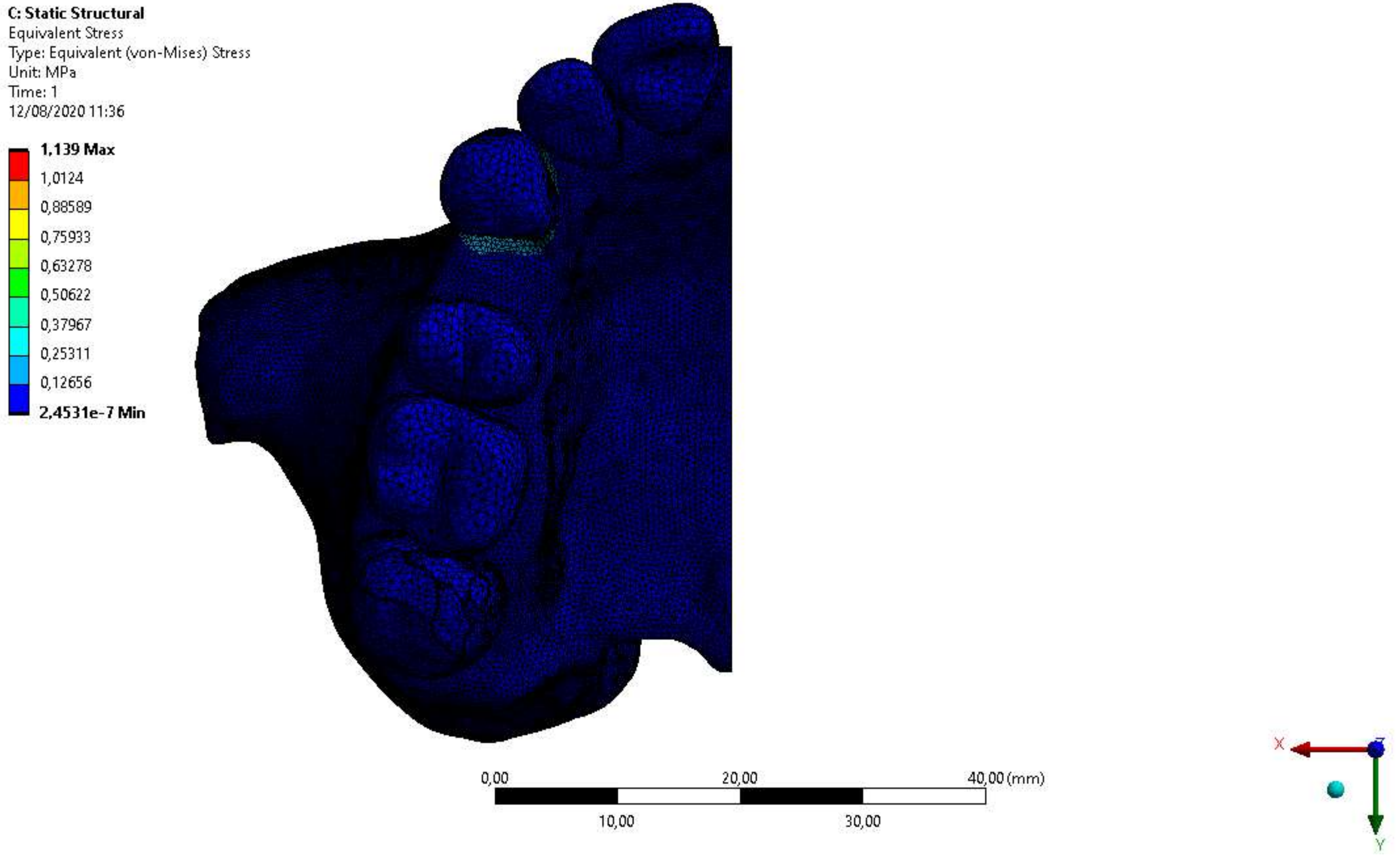
C: Static Structural
Equivalent Stress
Type: Equivalent (von-Mises) Stress
Unit: MPa
Time: 1
12/08/2020 11:36
1,139 Max
1,0124
0,88589
0,75933
0,63278
0,50622
0,37967
0,25311
0,12656
2,4531e-7 Min
0,00
20,00
40,00 (mm)
10,00
30,00
X
Z
Y

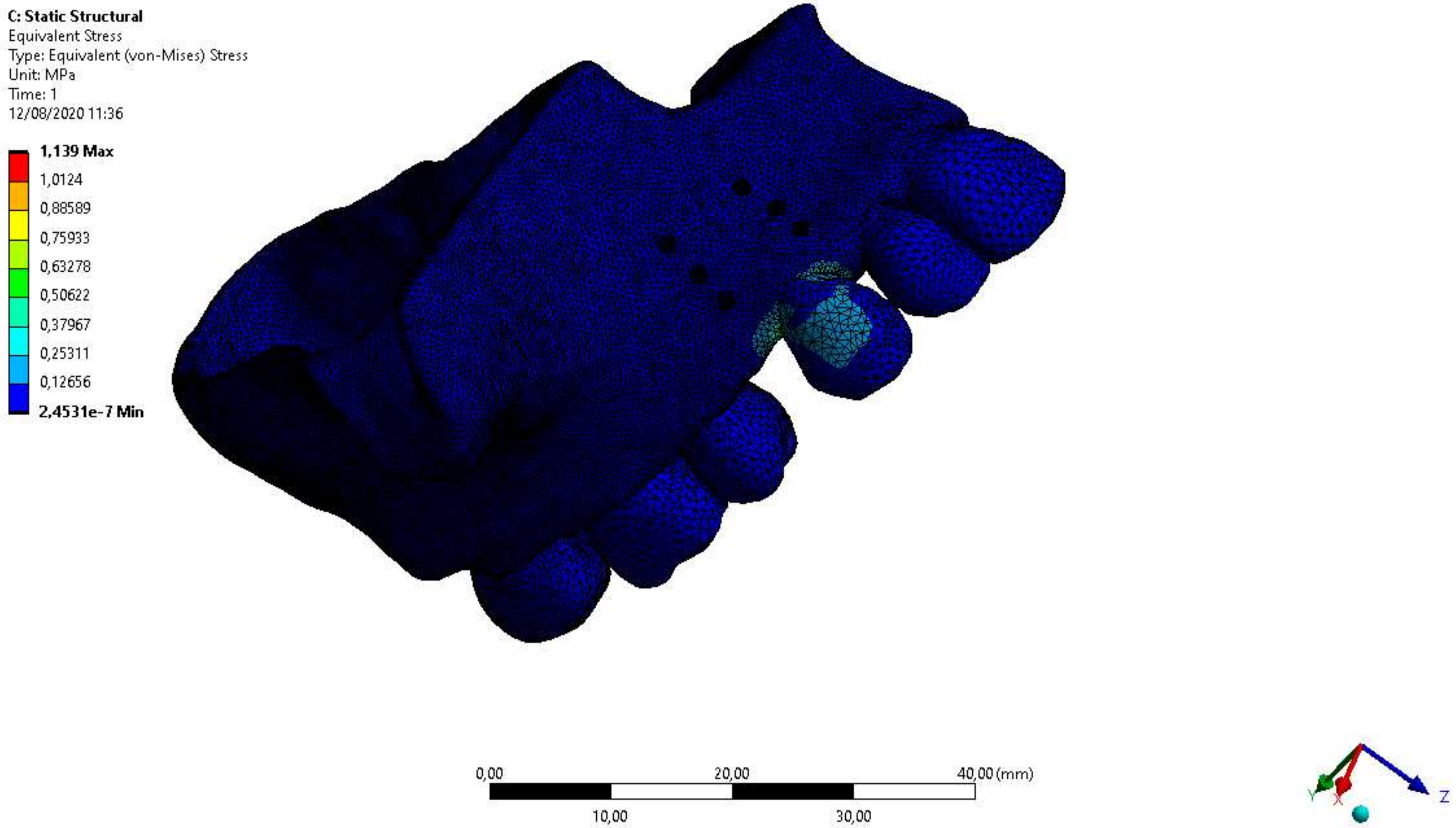
C: Static Structural
Equivalent Stress
Type: Equivalent (von-Mises) Stress
Unit: MPa
Time: 1
12/08/2020 11:36
1,139 Max
1,0124
0,88589
0,75933
0,63278
0,50622
0,37967
0,25311
0,12656
2,4531e-7 Min
0,00
20,00
40,00 (mm)
10,00
30,00
Z

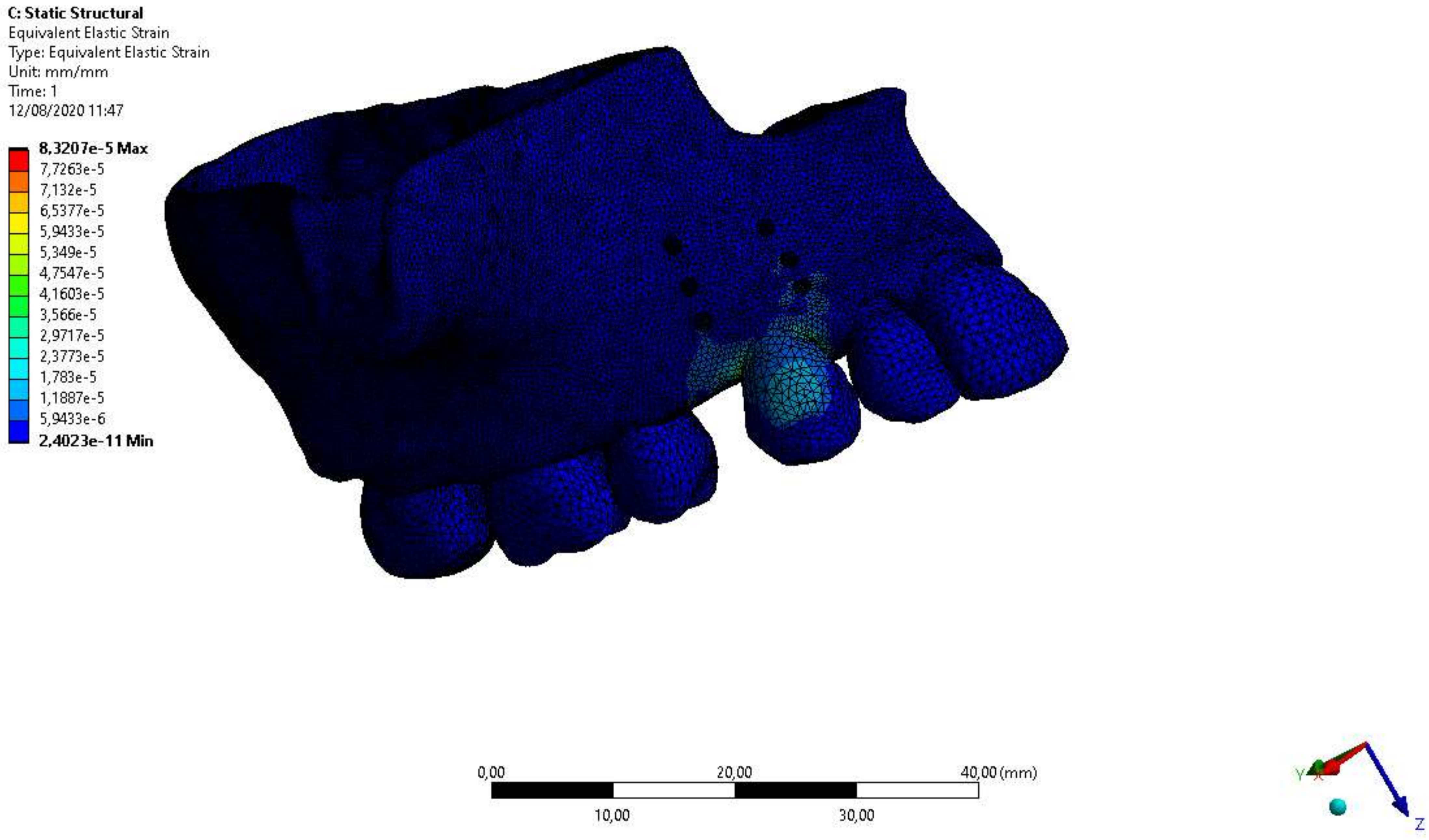
C: Static Structural
Equivalent Elastic Strain
Type: Equivalent Elastic Strain
Unit: mm/mm
Time: 1
12/08/2020 11:47
8,3207e-5 Max
7,7263e-5
7,132e-5
6,5377e-5
5,9433e-5
5,349e-5
4,7547e-5
4,1603e-5
3,566e-5
2,9717e-5
2,3773e-5
1,783e-5
1,1887e-5
5,9433e-6
2,4023e-11 Min
0,00
20,00
40,00 (mm)
10,00
30,00
Y
X
Z

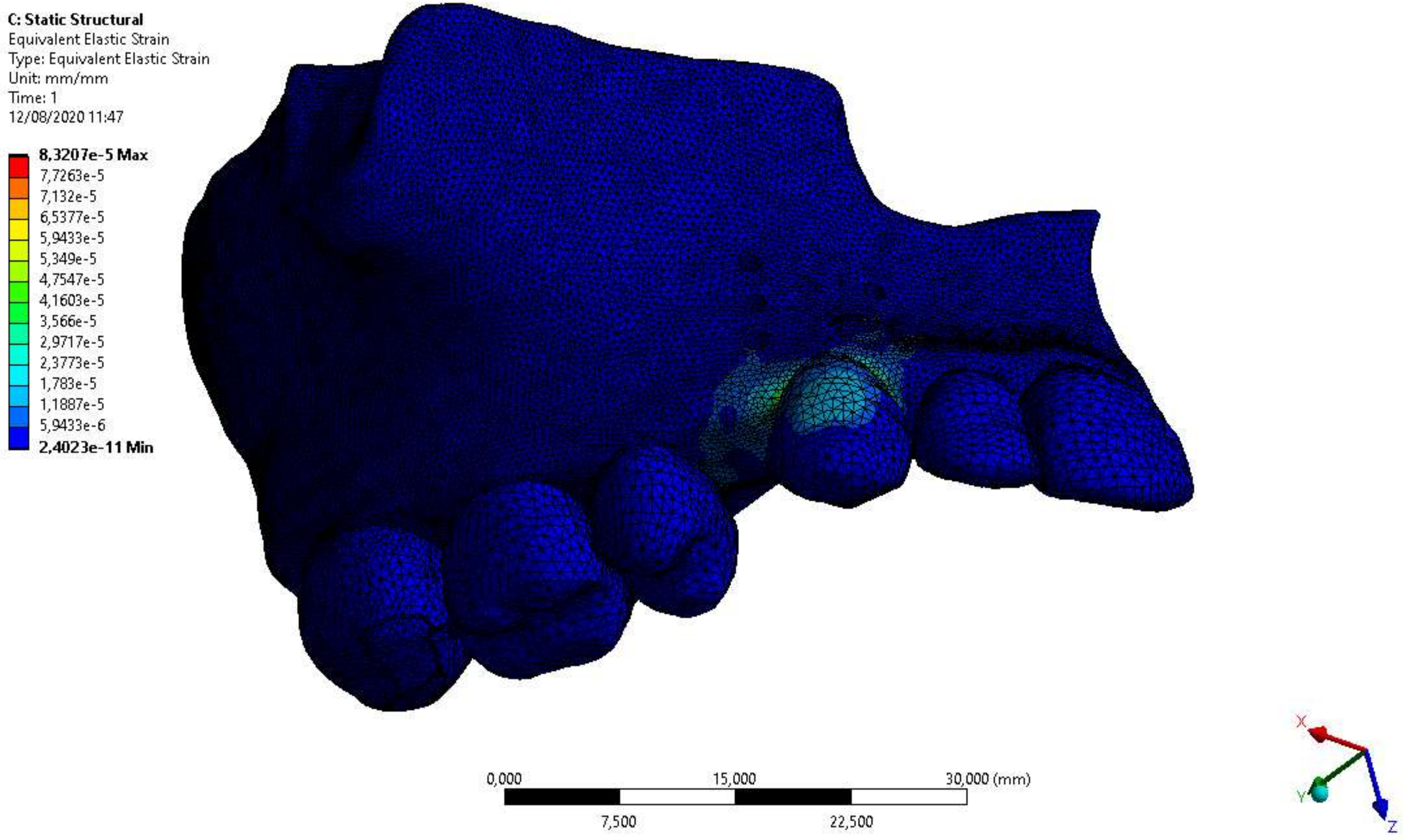
C: Static Structural
Equivalent Elastic Strain
Type: Equivalent Elastic Strain
Unit: mm/mm
Time: 1
12/08/2020 11:47
8,3207e-5 Max
7,7263e-5
7,132e-5
6,5377e-5
5,9433e-5
5,349e-5
4,7547e-5
4,1603e-5
3,566e-5
2,9717e-5
2,3773e-5
1,783e-5
1,1887e-5
5,9433e-6
2,4023e-11 Min
0,000
15,000
30,000 (mm)
7,500
22,500
X
Y
Z

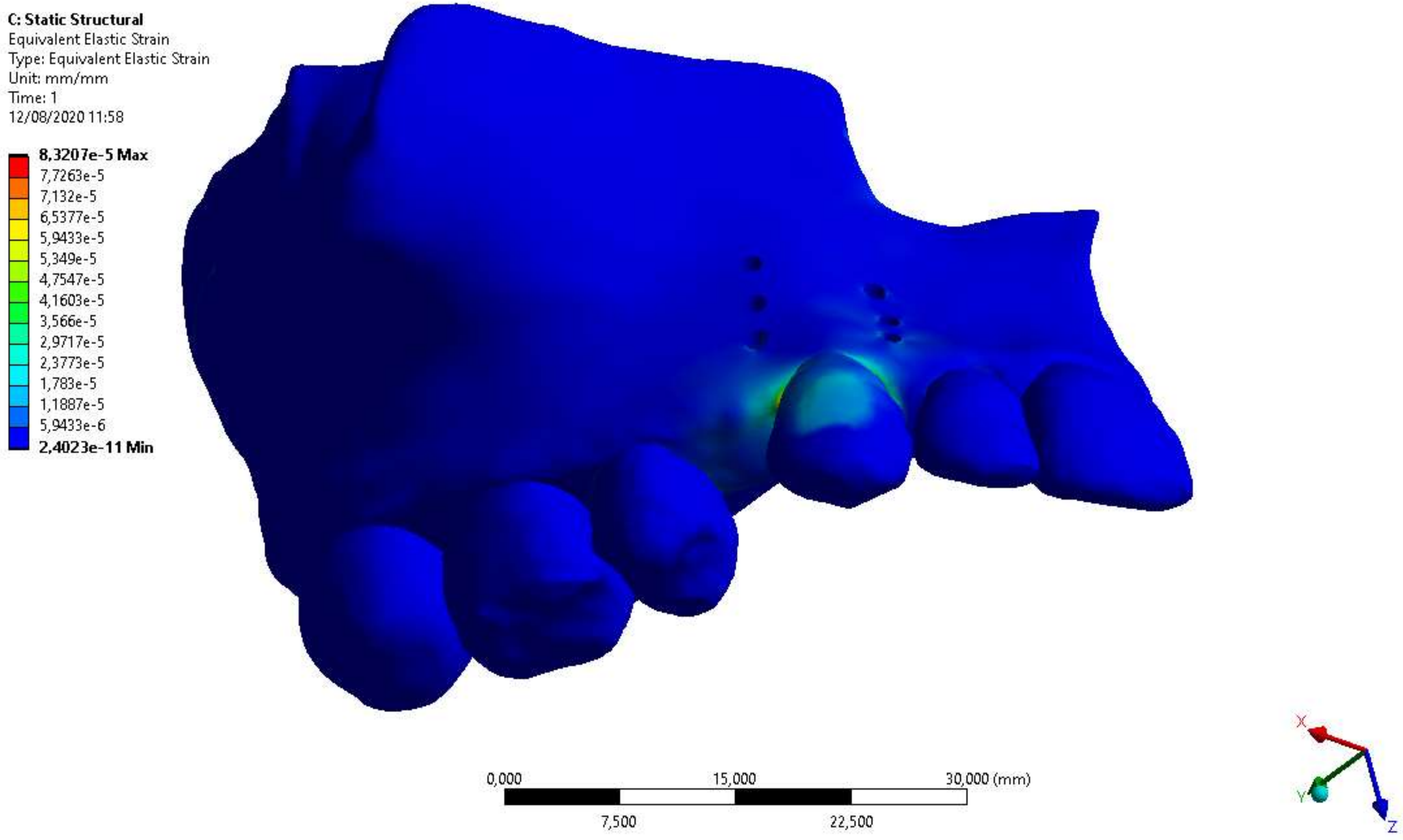
C: Static Structural
Equivalent Elastic Strain
Type: Equivalent Elastic Strain
Unit: mm/mm
Time: 1
12/08/2020 11:58
8,3207e-5 Max
7,7263e-5
7,132e-5
6,5377e-5
5,9433e-5
5,349e-5
4,7547e-5
4,1603e-5
3,566e-5
2,9717e-5
2,3773e-5
1,783e-5
1,1887e-5
5,9433e-6
2,4023e-11 Min
0,000
15,000
30,000 (mm)
7,500
22,500
X
Y
Z

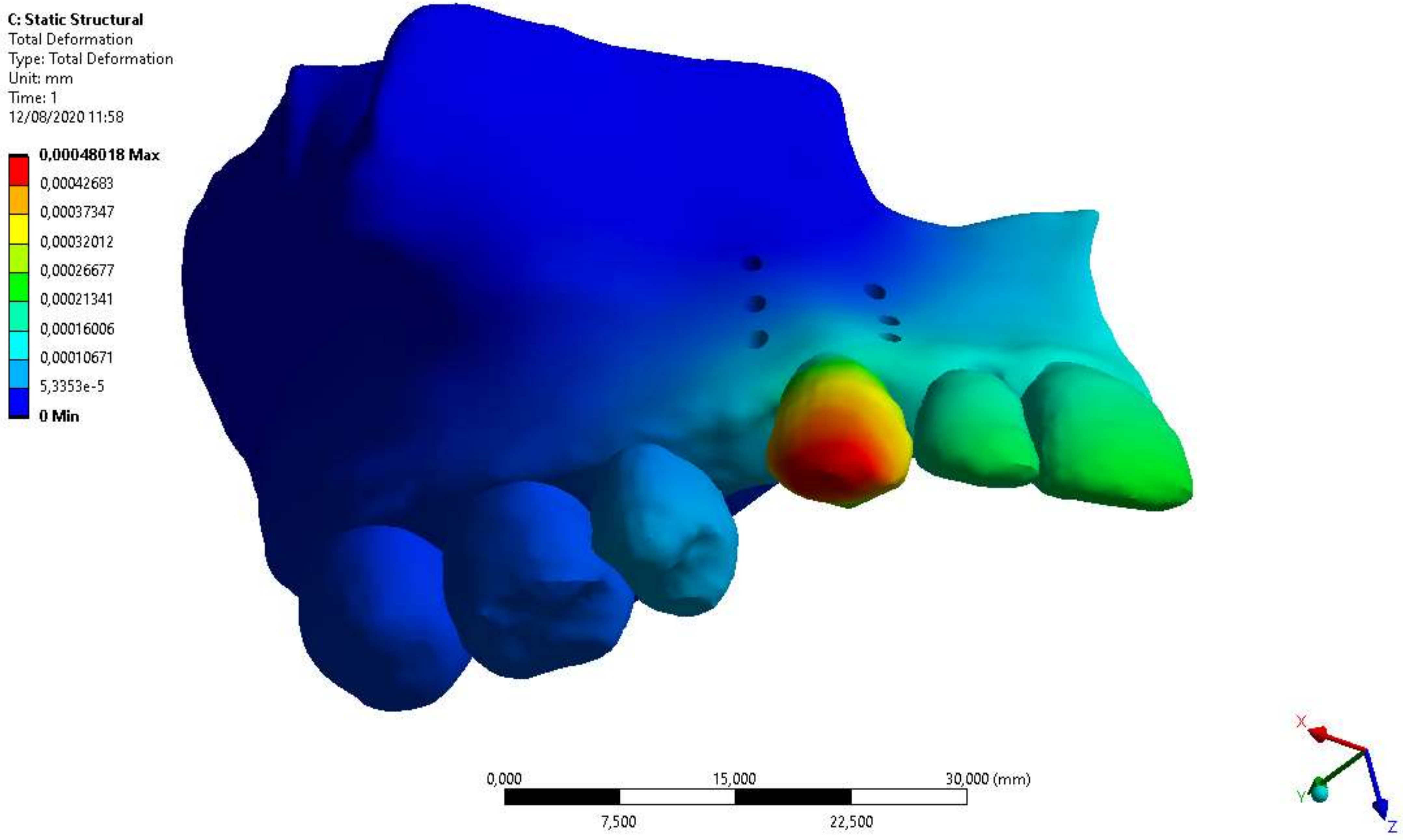

C: Static Structural
Total Deformation
Type: Total Deformation
Unit: mm
Time: 1
12/08/2020 11:58
0,00048018 Max
0,00042683
0,00037347
0,00032012
0,00026677
0,00021341
0,00016006
0,00010671
5,3353e-5
0 Min
0,000
15,000
30,000 (mm)
7,500
22,500
X
Y
Z

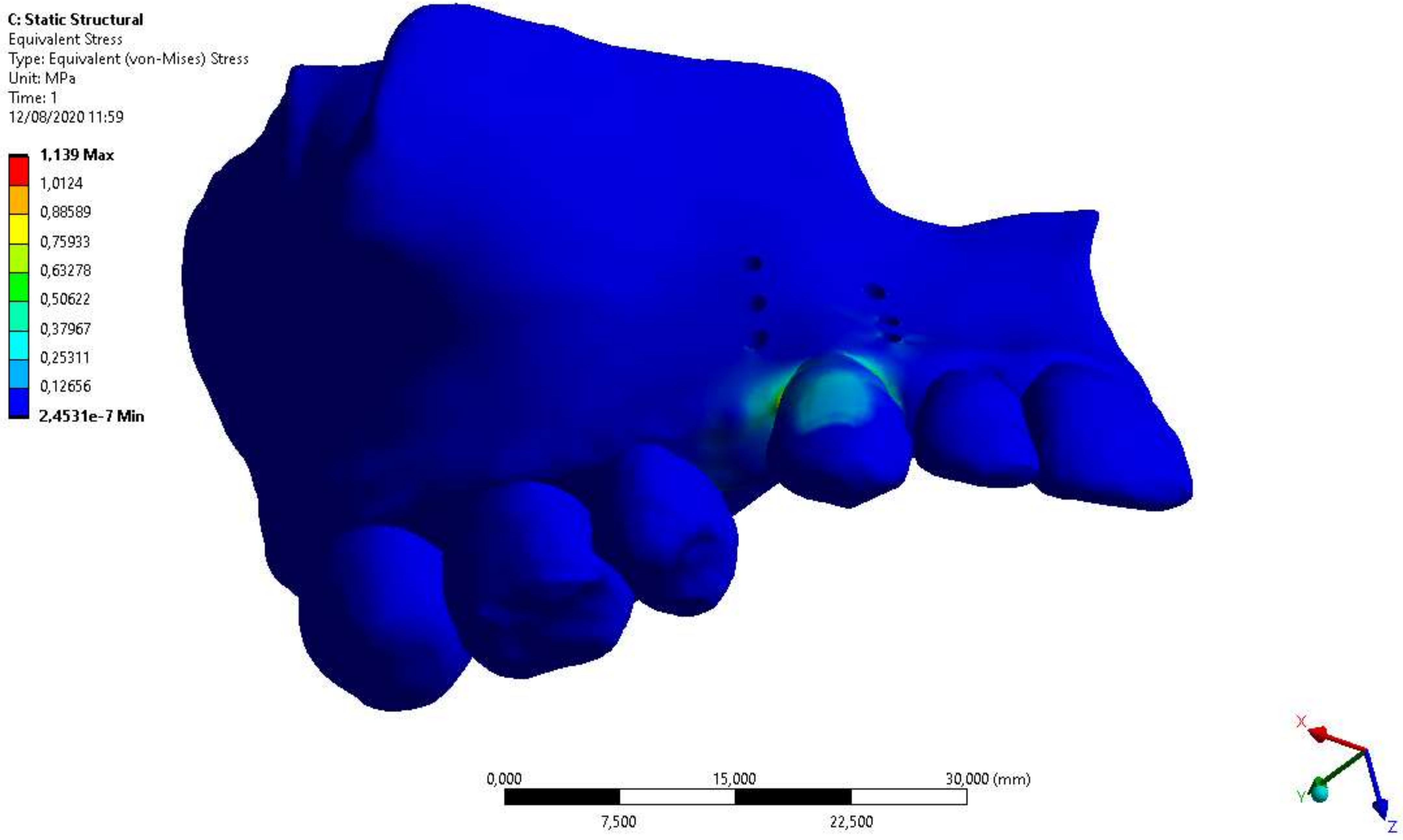

C: Static Structural
Equivalent Stress
Type: Equivalent (von-Mises) Stress
Unit: MPa
Time: 1
12/08/2020 11:59
1,139 Max
1,0124
0,88589
0,75933
0,63278
0,50622
0,37967
0,25311
0,12656
2,4531e-7 Min
0,000
15,000
30,000 (mm)
7,500
22,500
X
Y
Z

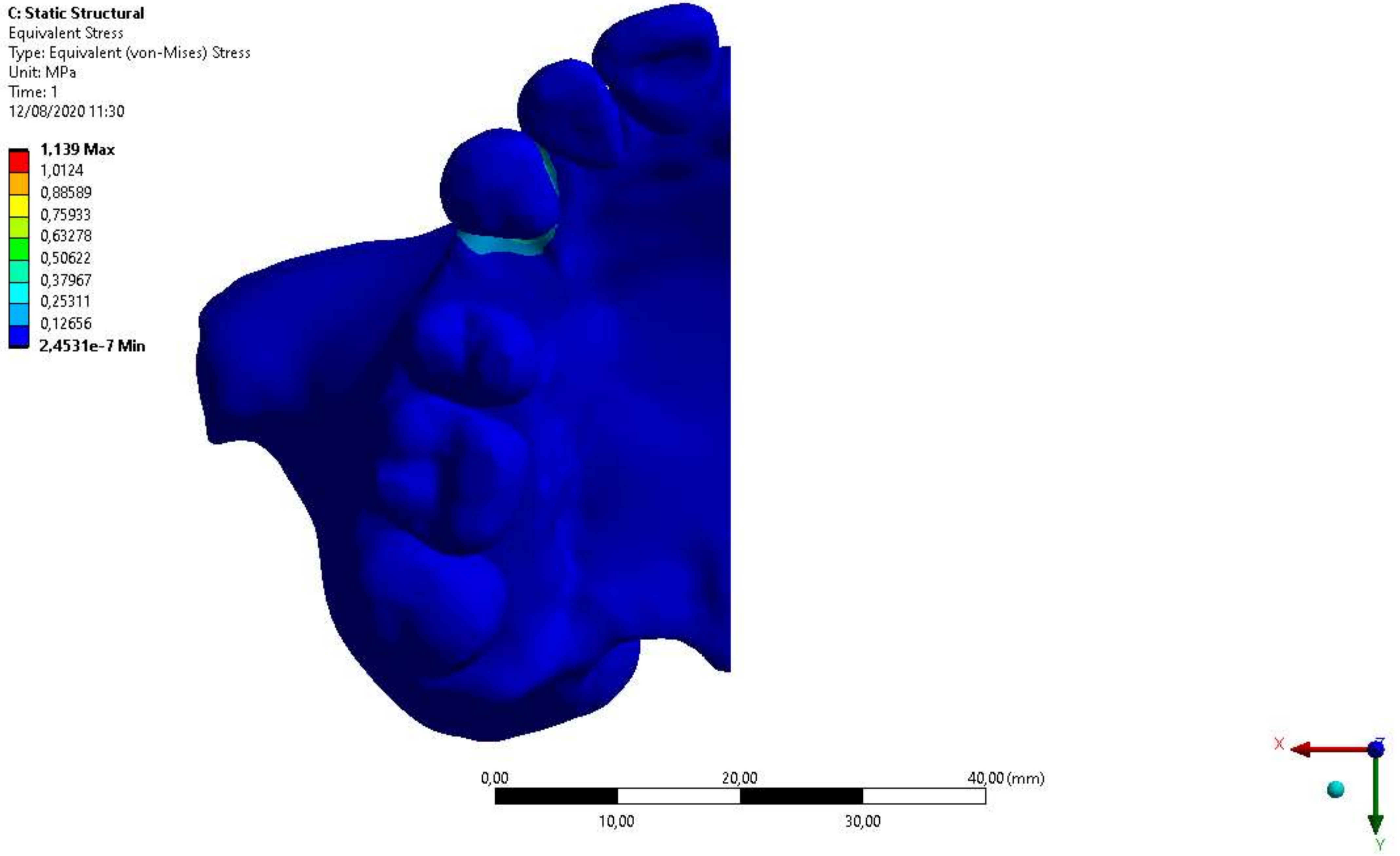
C: Static Structural
Equivalent Stress
Type: Equivalent (von-Mises) Stress
Unit: MPa
Time: 1
12/08/2020 11:30
1,139 Max
1,0124
0,88589
0,75933
0,63278
0,50622
0,37967
0,25311
0,12656
2,4531e-7 Min
0,00
20,00
40,00 (mm)
10,00
30,00
X
Z
Y

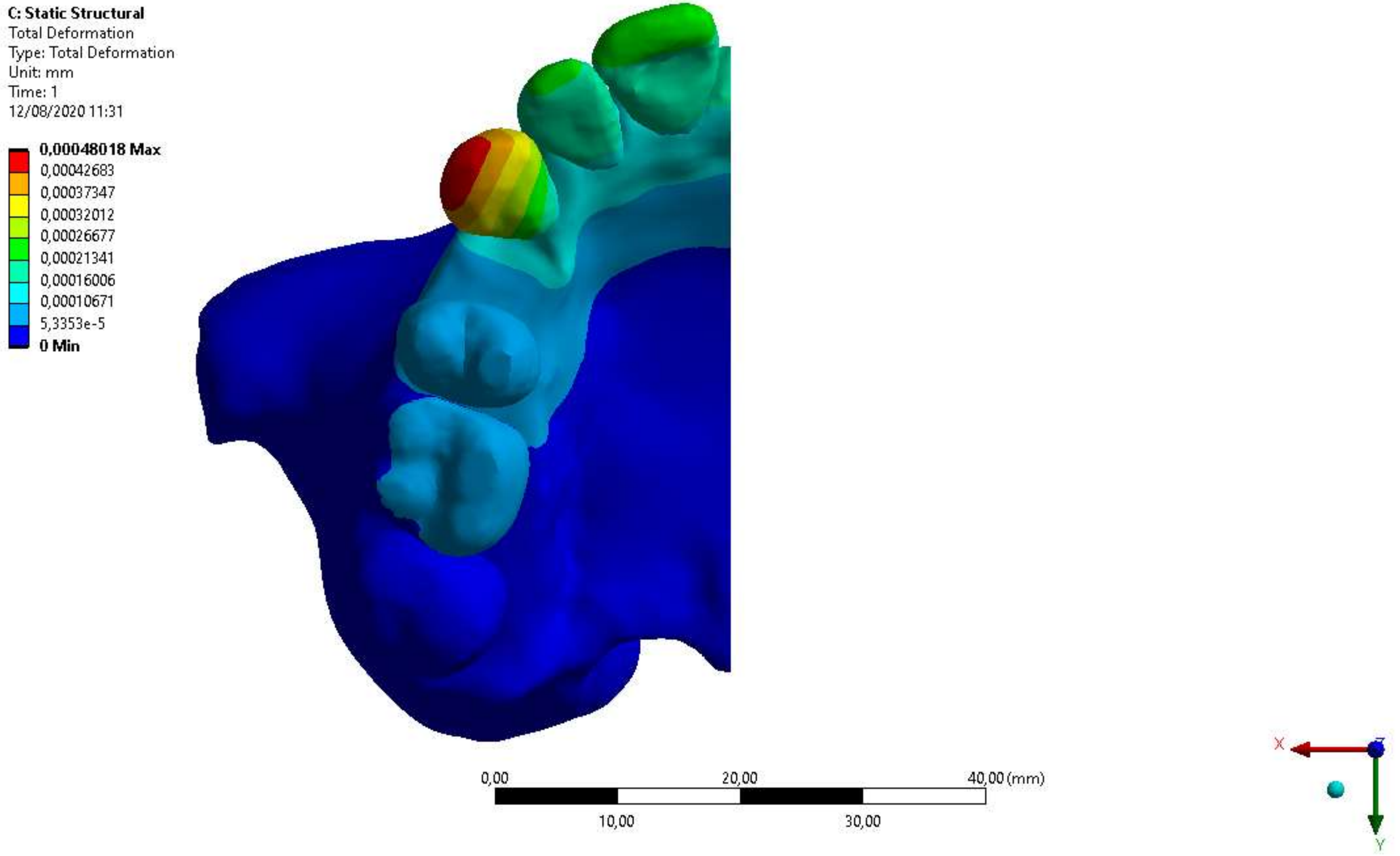
C: Static Structural
Total Deformation
Type: Total Deformation
Unit: mm
Time: 1
12/08/2020 11:31
0,00048018 Max
0,00042683
0,00037347
0,00032012
0,00026677
0,00021341
0,00016006
0,00010671
5,3353e-5
0 Min
0,00
10,00
20,00
30,00
40,00 (mm)
X
Y
Z

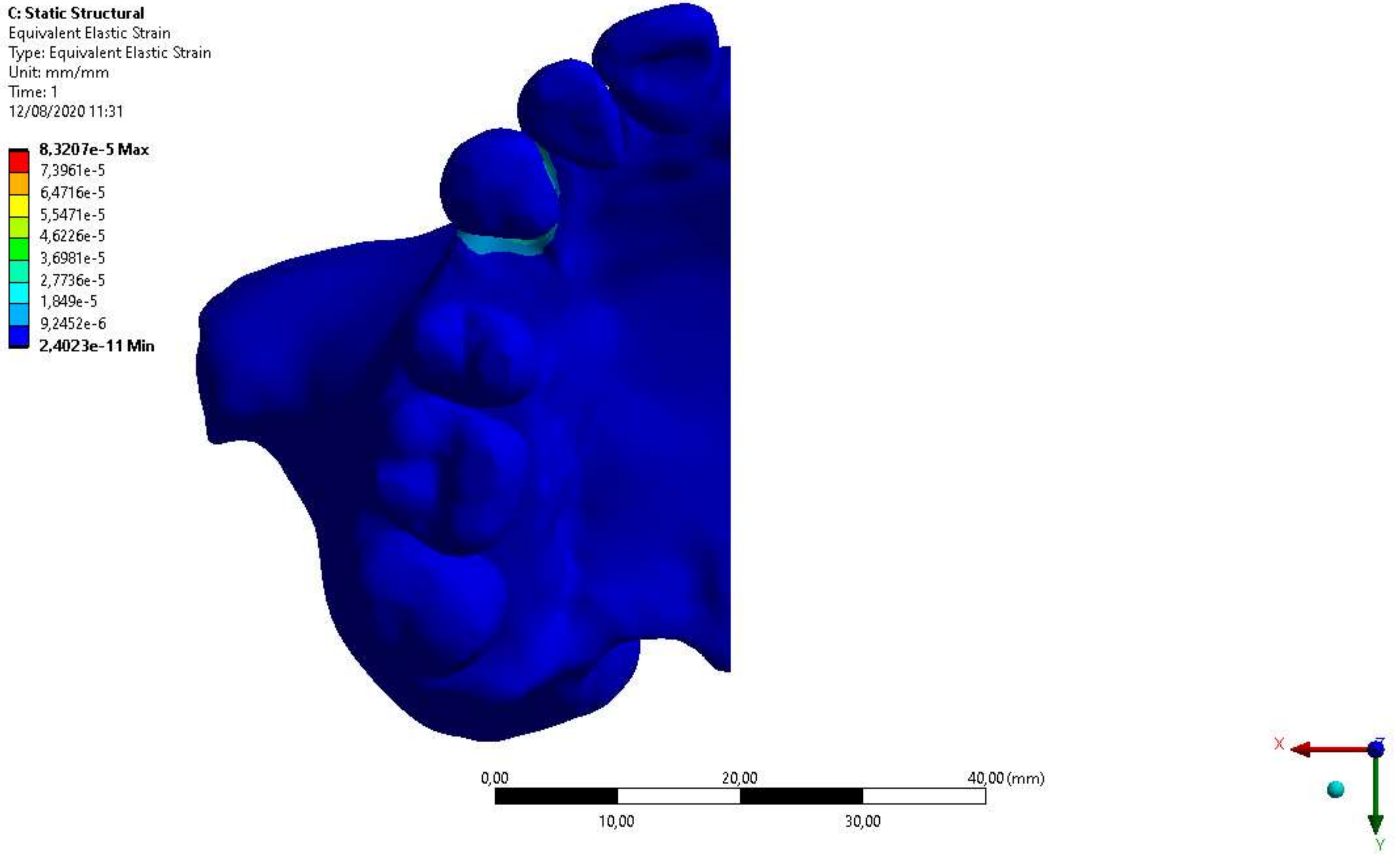
C: Static Structural
Equivalent Elastic Strain
Type: Equivalent Elastic Strain
Unit: mm/mm
Time: 1
12/08/2020 11:31
8,3207e-5 Max
7,3961e-5
6,4716e-5
5,5471e-5
4,6226e-5
3,6981e-5
2,7736e-5
1,849e-5
9,2452e-6
2,4023e-11 Min
0,00
20,00
40,00 (mm)
10,00
30,00
X
Y
Z

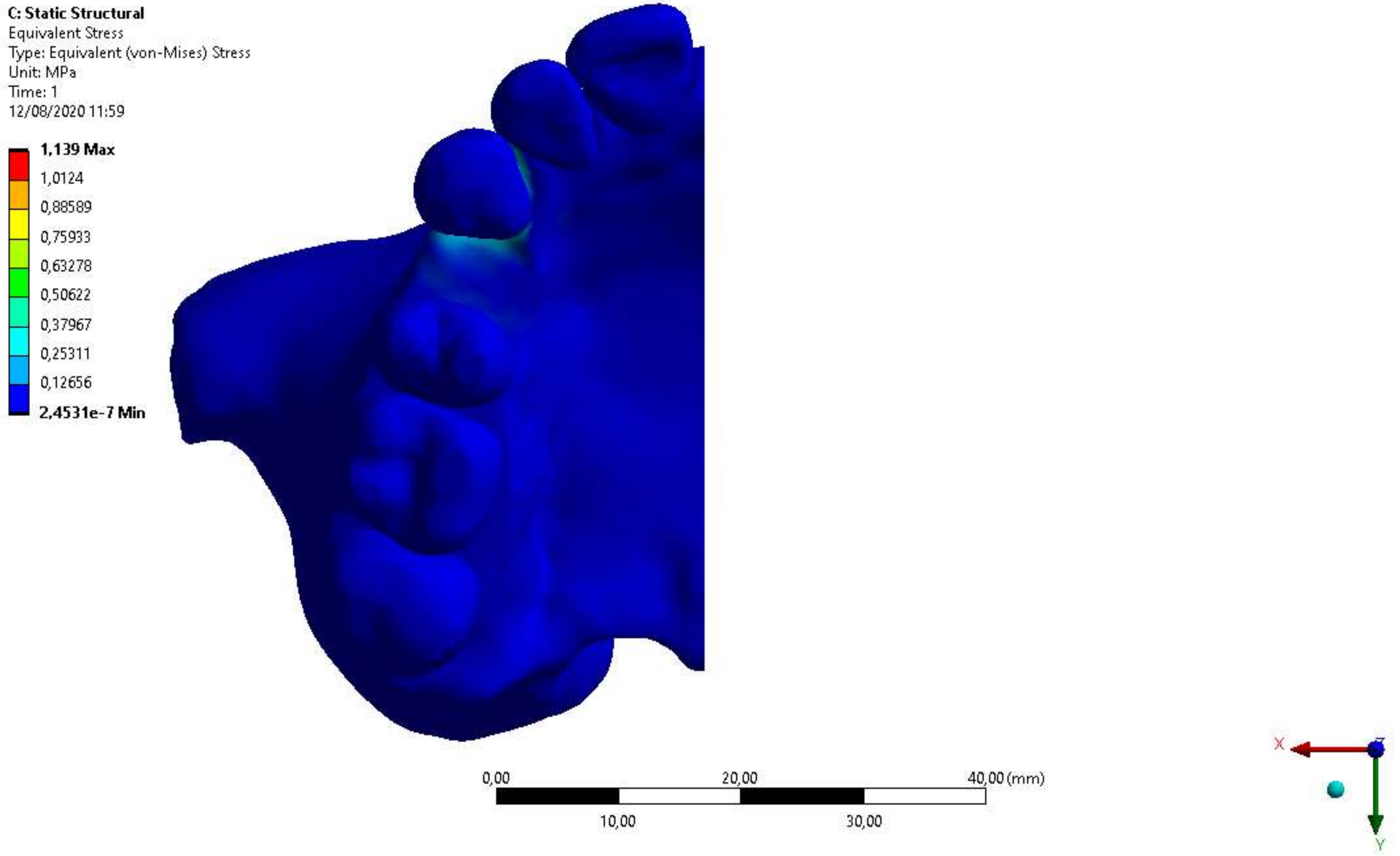
C: Static Structural
Equivalent Stress
Type: Equivalent (von-Mises) Stress
Unit: MPa
Time: 1
12/08/2020 11:59
1,139 Max
1,0124
0,88589
0,75933
0,63278
0,50622
0,37967
0,25311
0,12656
2,4531e-7 Min
0,00
20,00
40,00 (mm)
10,00
30,00
X
Y

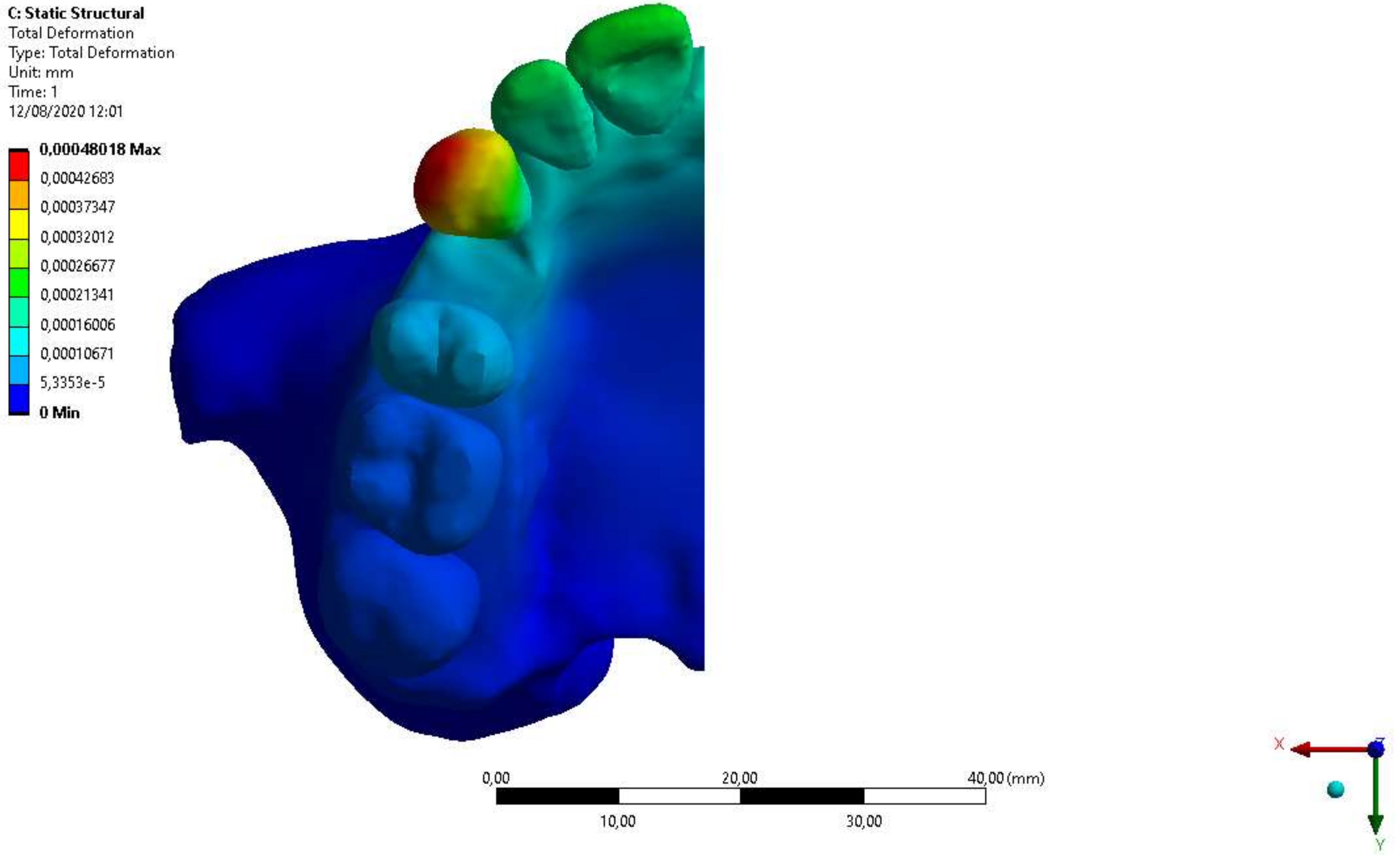

C: Static Structural
Total Deformation
Type: Total Deformation
Unit: mm
Time: 1
12/08/2020 12:01
0,00048018 Max
0,00042683
0,00037347
0,00032012
0,00026677
0,00021341
0,00016006
0,00010671
5,3353e-5
0 Min
0,00
20,00
40,00 (mm)
10,00
30,00
X
Z
Y

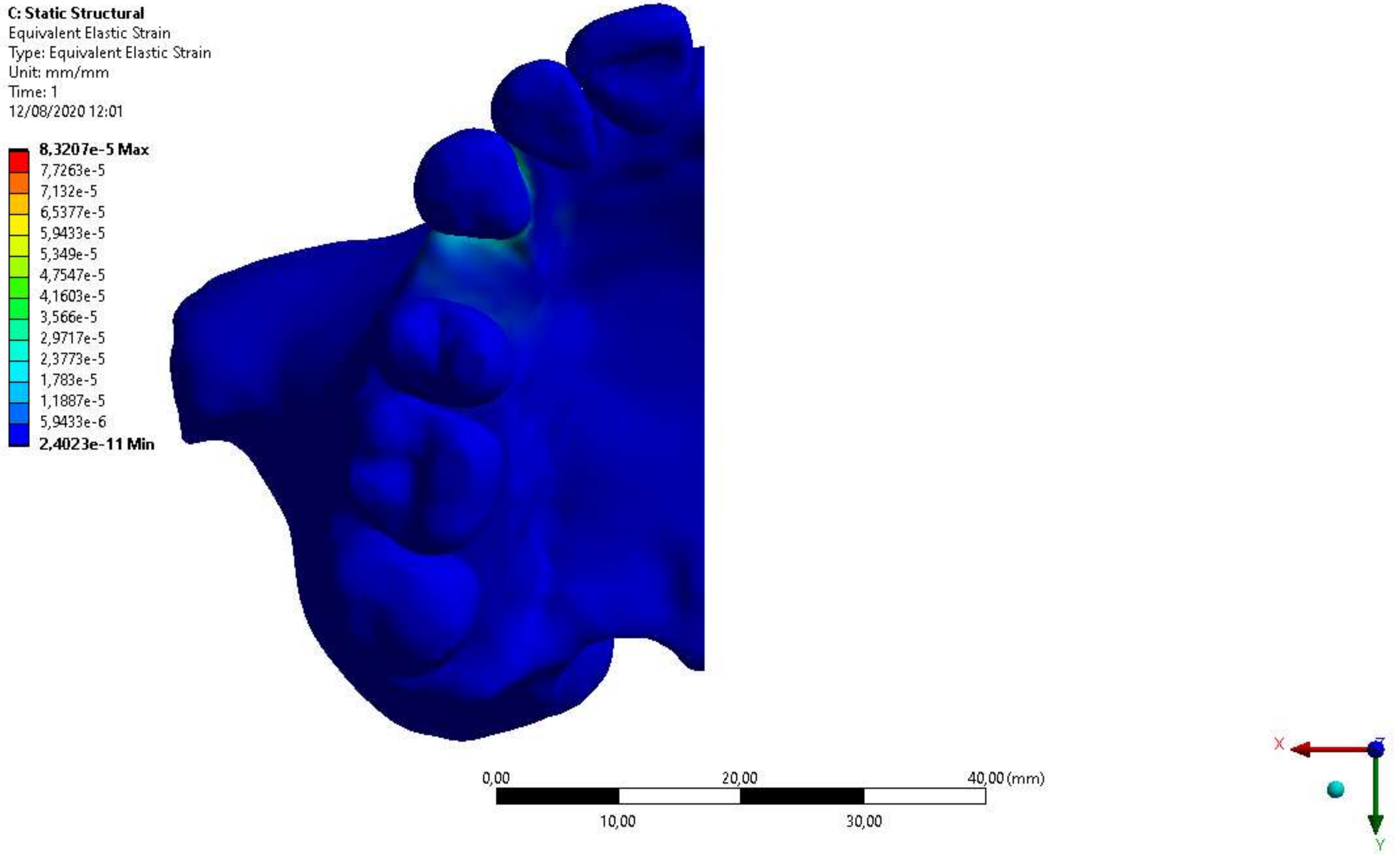
C: Static Structural
Equivalent Elastic Strain
Type: Equivalent Elastic Strain
Unit: mm/mm
Time: 1
12/08/2020 12:01
8,3207e-5 Max
7,7263e-5
7,132e-5
6,5377e-5
5,9433e-5
5,349e-5
4,7547e-5
4,1603e-5
3,566e-5
2,9717e-5
2,3773e-5
1,783e-5
1,1887e-5
5,9433e-6
2,4023e-11 Min
0,00
20,00
40,00 (mm)
10,00
30,00
X
Z
Y

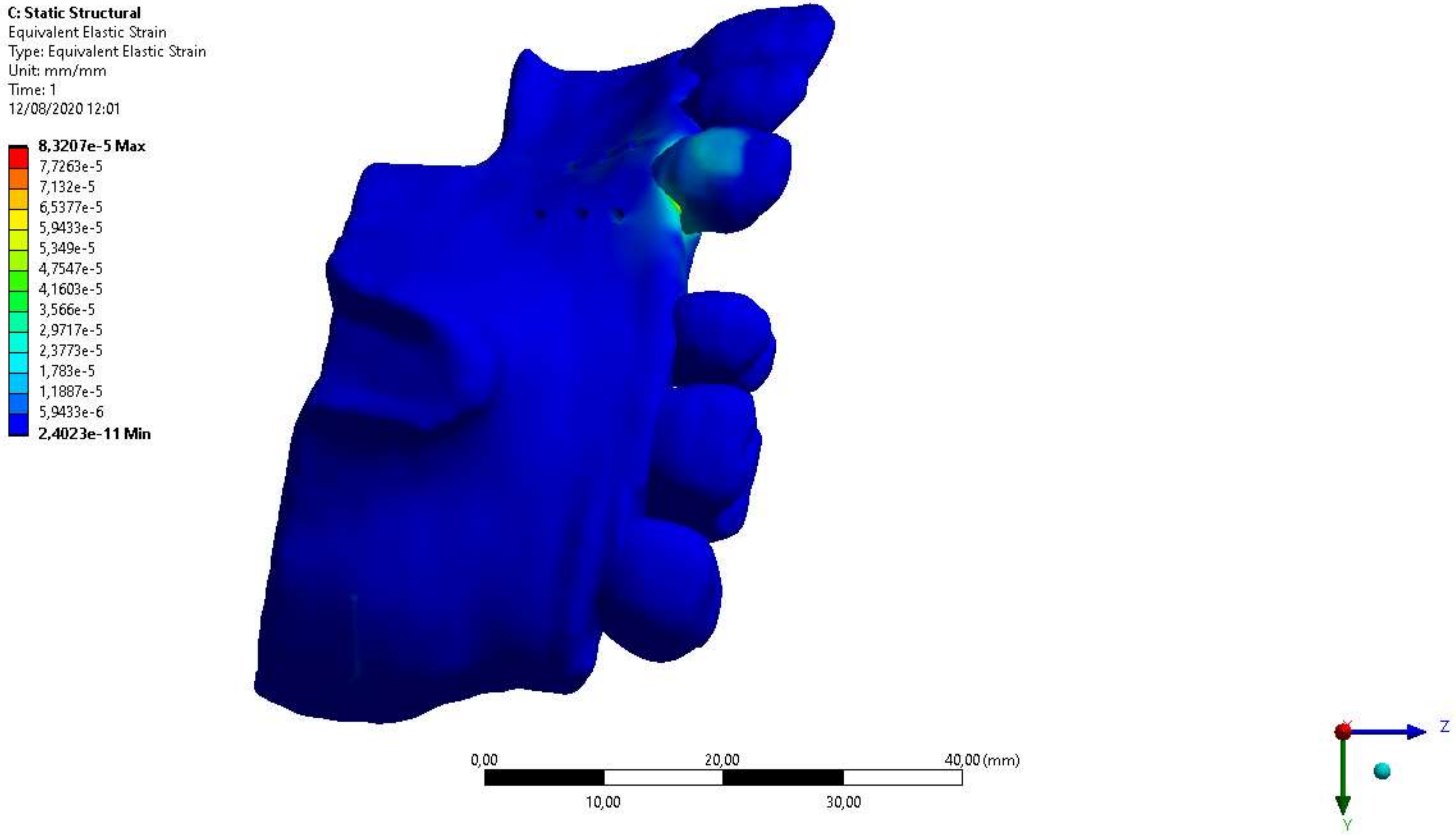

C: Static Structural
Equivalent Elastic Strain
Type: Equivalent Elastic Strain
Unit: mm/mm
Time: 1
12/08/2020 12:01
8,3207e-5 Max
7,7263e-5
7,132e-5
6,5377e-5
5,9433e-5
5,349e-5
4,7547e-5
4,1603e-5
3,566e-5
2,9717e-5
2,3773e-5
1,783e-5
1,1887e-5
5,9433e-6
2,4023e-11 Min
0,00
20,00
40,00 (mm)
10,00
30,00
Z
Y

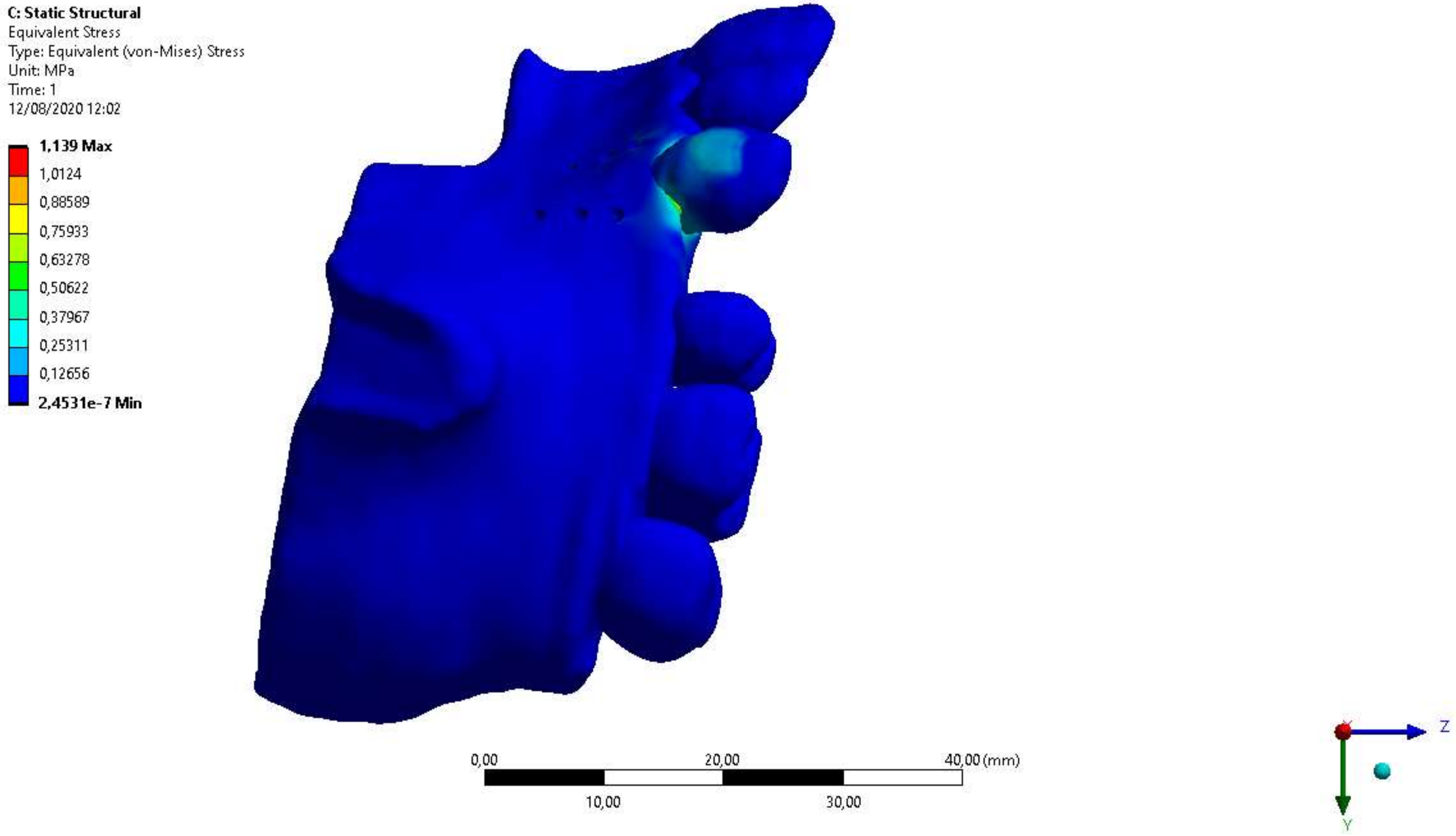
C: Static Structural
Equivalent Stress
Type: Equivalent (von-Mises) Stress
Unit: MPa
Time: 1
12/08/2020 12:02
1,139 Max
1,0124
0,88589
0,75933
0,63278
0,50622
0,37967
0,25311
0,12656
2,4531e-7 Min
0,00
20,00
40,00 (mm)
10,00
30,00
Z
Y

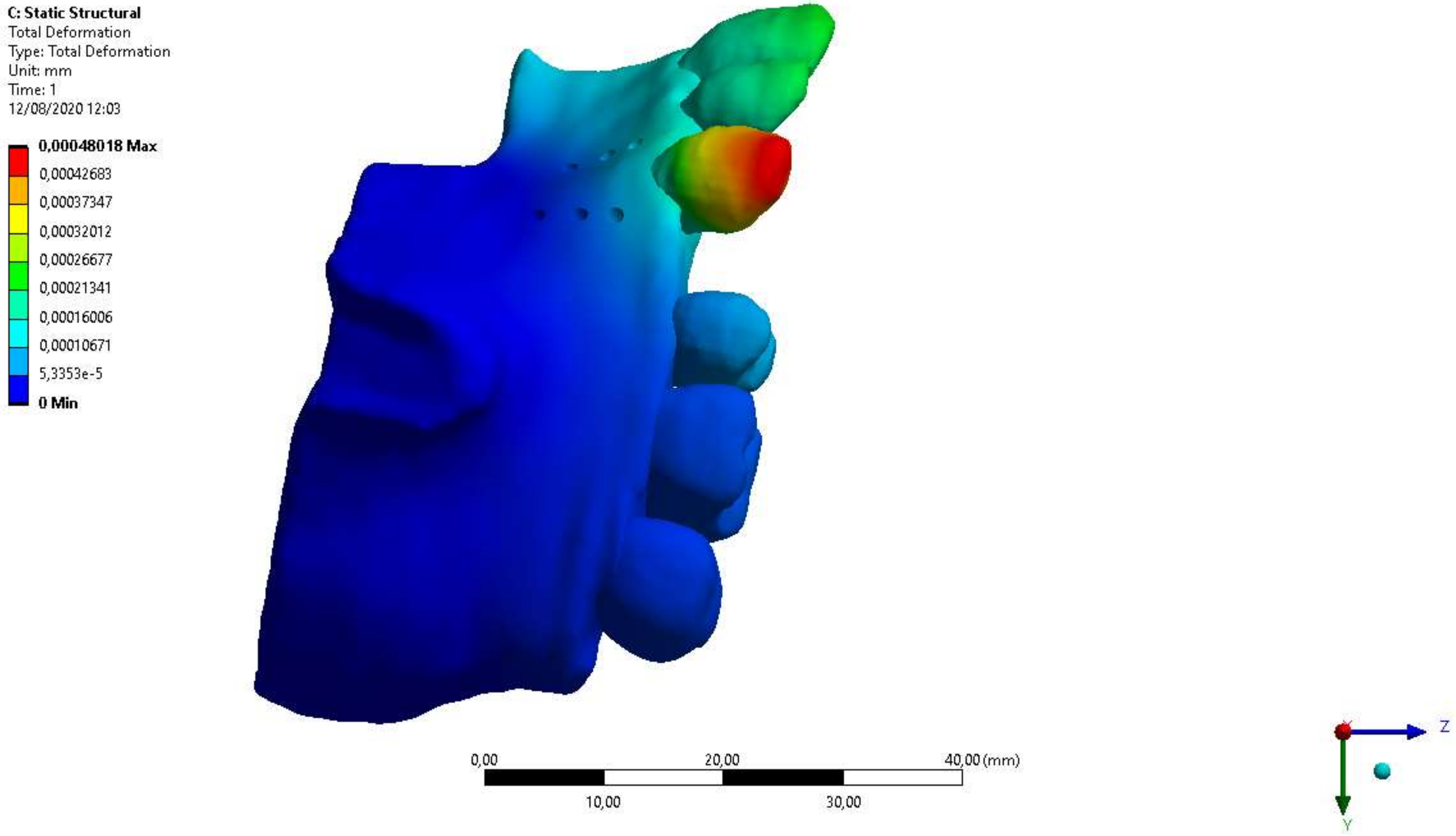
C: Static Structural
Total Deformation
Type: Total Deformation
Unit: mm
Time: 1
12/08/2020 12:03
0,00048018 Max
0,00042683
0,00037347
0,00032012
0,00026677
0,00021341
0,00016006
0,00010671
5,3353e-5
0 Min
0,00
20,00
40,00 (mm)
10,00
30,00
Z
Y

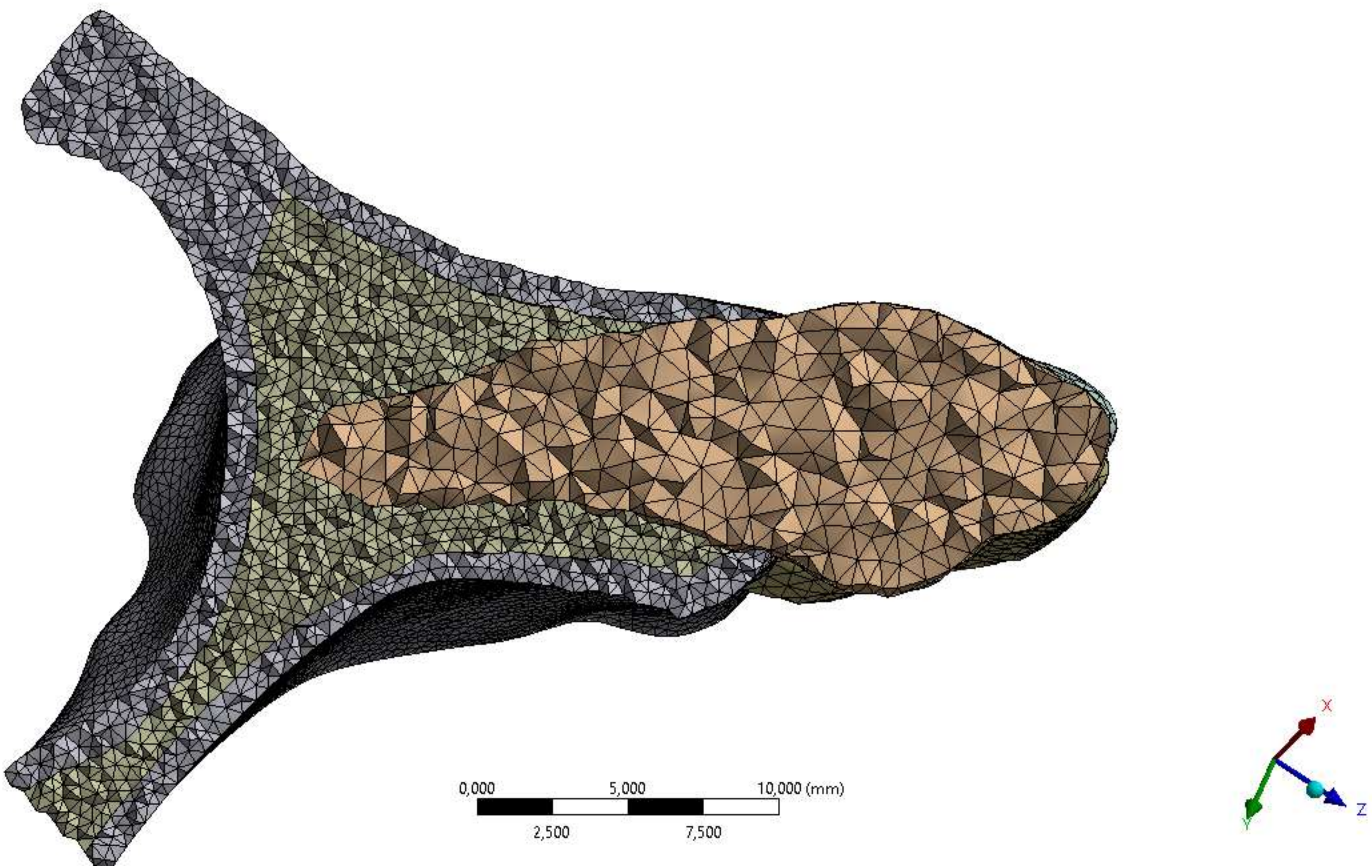

0,000
5,000
10,000 (mm)
2,500
7,500
X
Y
Z

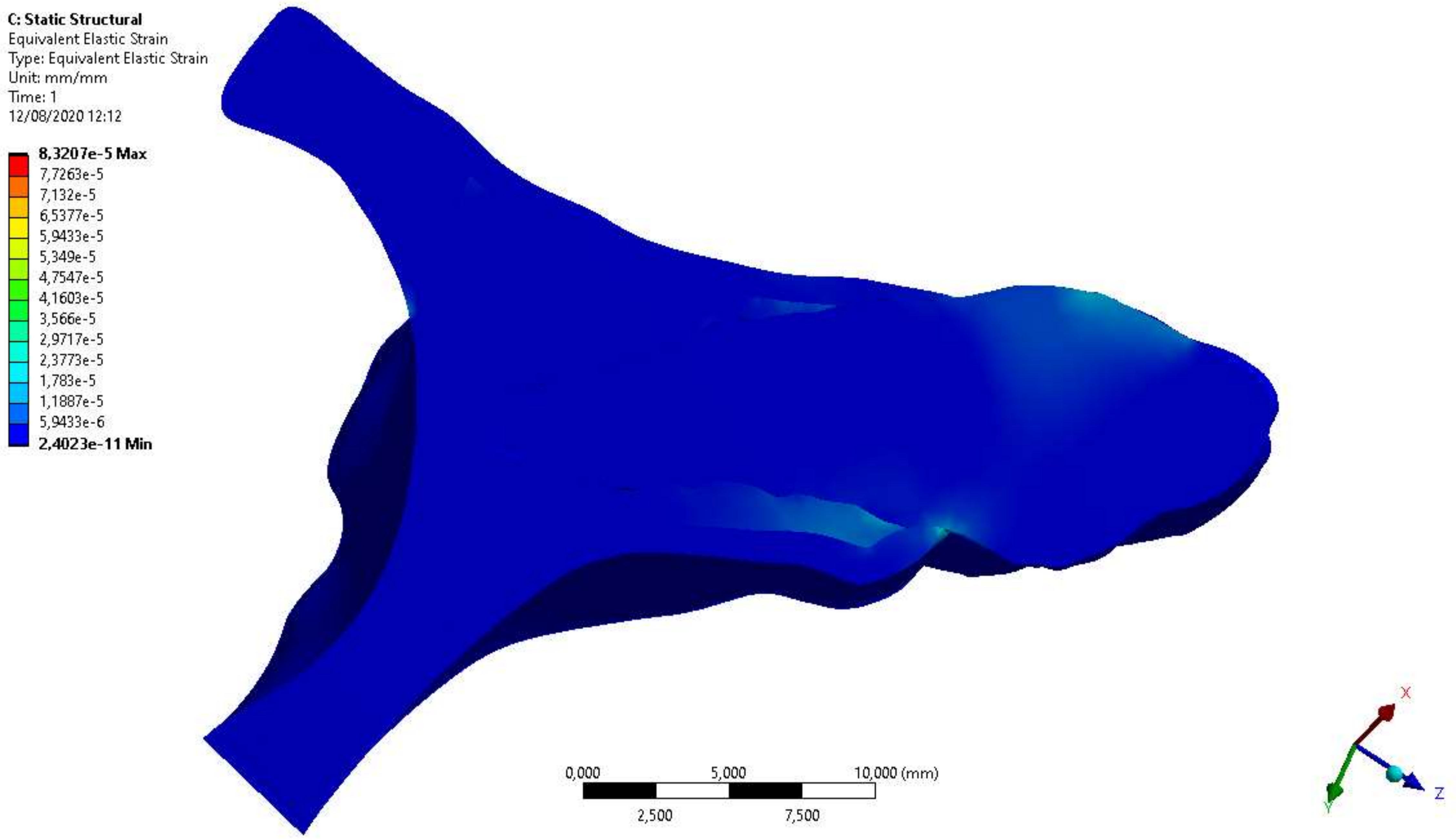
C: Static Structural
Equivalent Elastic Strain
Type: Equivalent Elastic Strain
Unit: mm/mm
Time: 1
12/08/2020 12:12
8,3207e-5 Max
7,7263e-5
7,132e-5
6,5377e-5
5,9433e-5
5,349e-5
4,7547e-5
4,1603e-5
3,566e-5
2,9717e-5
2,3773e-5
1,783e-5
1,1887e-5
5,9433e-6
2,4023e-11 Min
0,000
5,000
10,000 (mm)
2,500
7,500
X
Y
Z

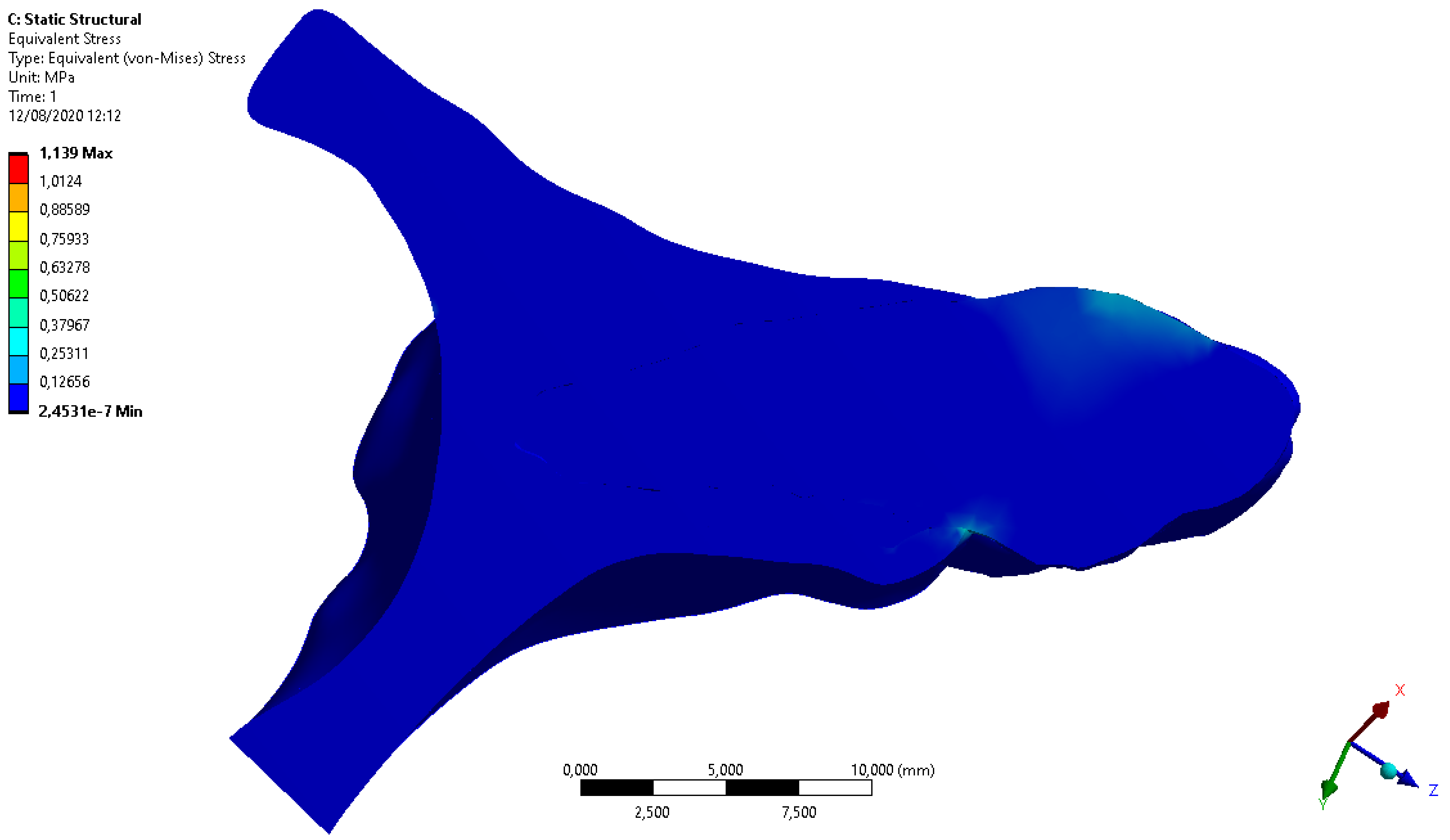
C: Static Structural
Equivalent Stress
Type: Equivalent (von-Mises) Stress
Unit: MPa
Time: 1
12/08/2020 12:12
1,139 Max
1,0124
0,88589
0,75933
0,63278
0,50622
0,37967
0,25311
0,12656
2,4531e-7 Min
0,000
5,000
10,000 (mm)
2,500
7,500
X
Y
Z

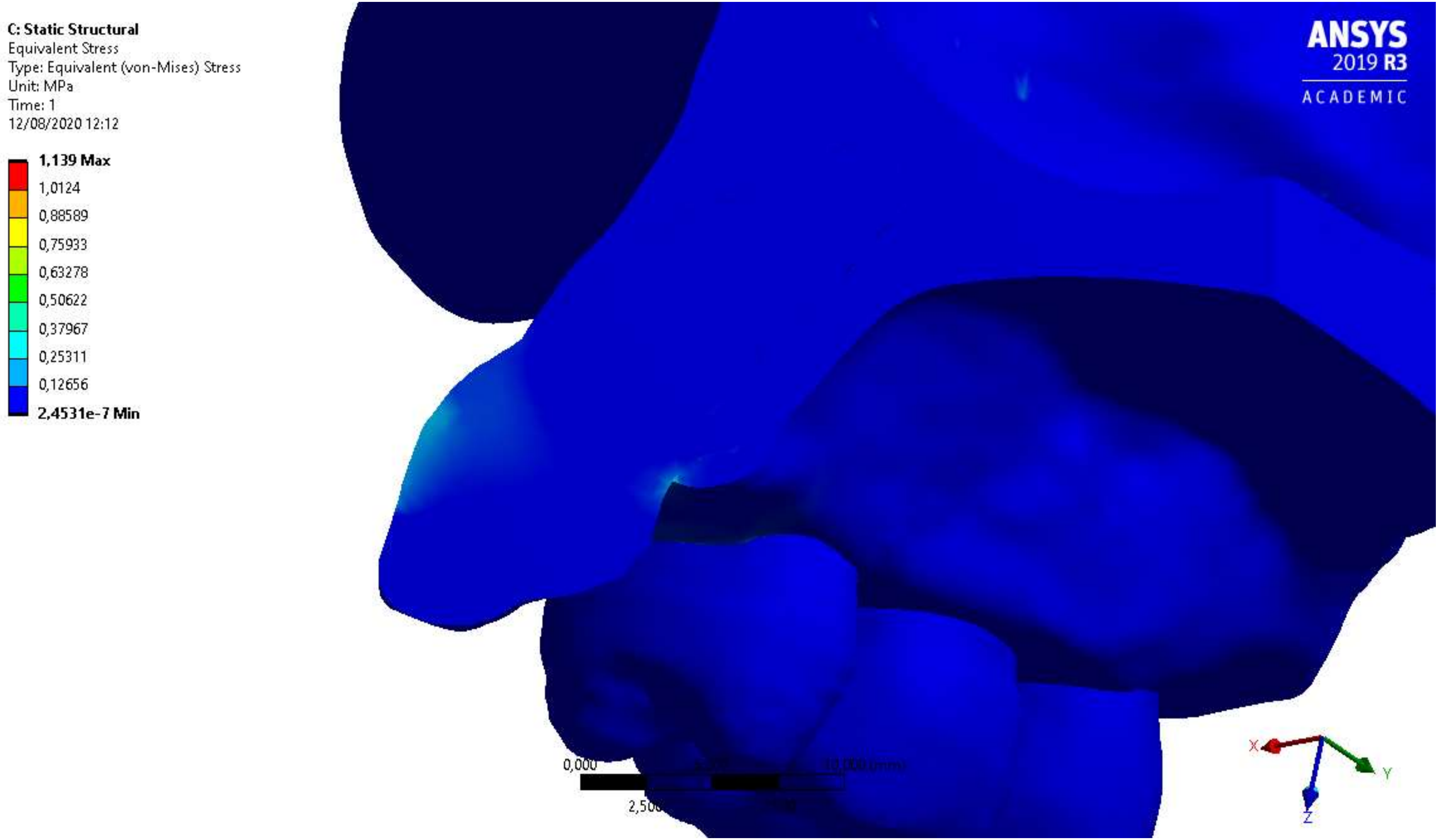
C: Static Structural
Equivalent Stress
Type: Equivalent (von-Mises) Stress
Unit: MPa
Time: 1
12/08/2020 12:12
1,139 Max
1,0124
0,88589
0,75933
0,63278
0,50622
0,37967
0,25311
0,12656
2,4531e-7 Min
ANSYS
2019 R3
ACADEMIC
0,000
X
Y
Z

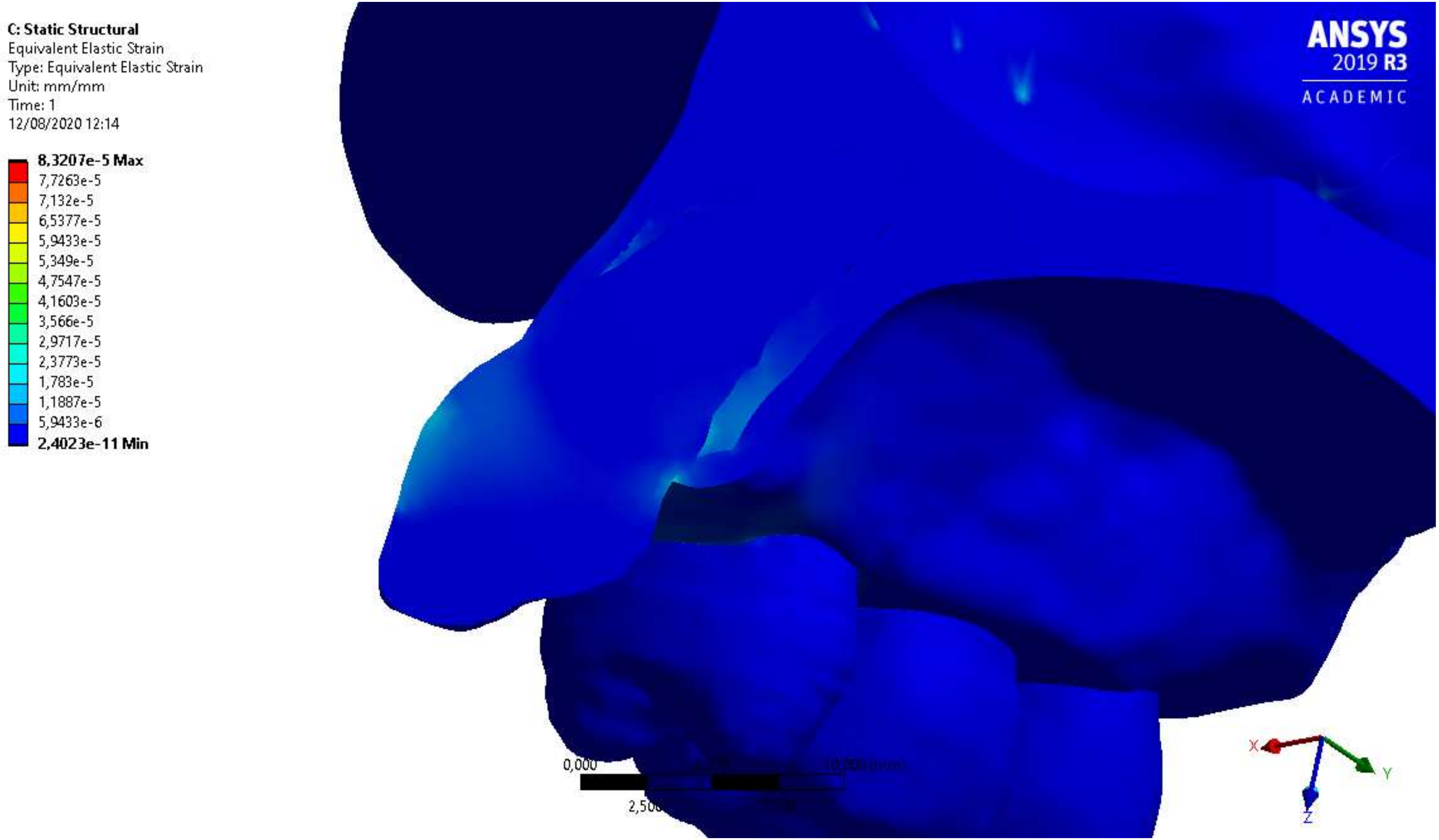
C: Static Structural
Equivalent Elastic Strain
Type: Equivalent Elastic Strain
Unit: mm/mm
Time: 1
12/08/2020 12:14
8,3207e-5 Max
7,7263e-5
7,132e-5
6,5377e-5
5,9433e-5
5,349e-5
4,7547e-5
4,1603e-5
3,566e-5
2,9717e-5
2,3773e-5
1,783e-5
1,1887e-5
5,9433e-6
2,4023e-11 Min
ANSYS
2019 R3
ACADEMIC
0,000
X
Y
Z

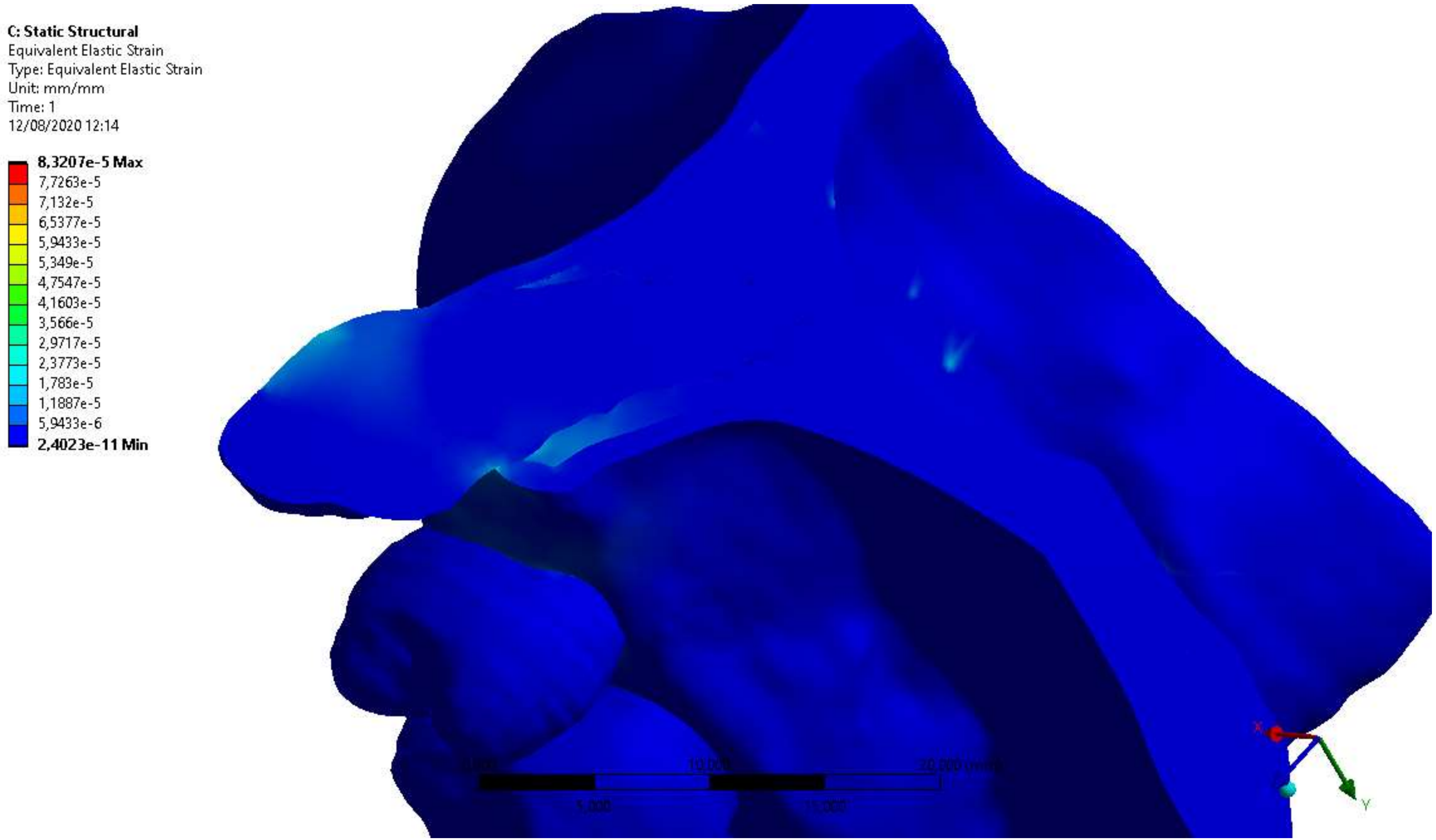
C: Static Structural
Equivalent Elastic Strain
Type: Equivalent Elastic Strain
Unit: mm/mm
Time: 1
12/08/2020 12:14
8,3207e-5 Max
7,7263e-5
7,132e-5
6,5377e-5
5,9433e-5
5,349e-5
4,7547e-5
4,1603e-5
3,566e-5
2,9717e-5
2,3773e-5
1,783e-5
1,1887e-5
5,9433e-6
2,4023e-11 Min
X
Y

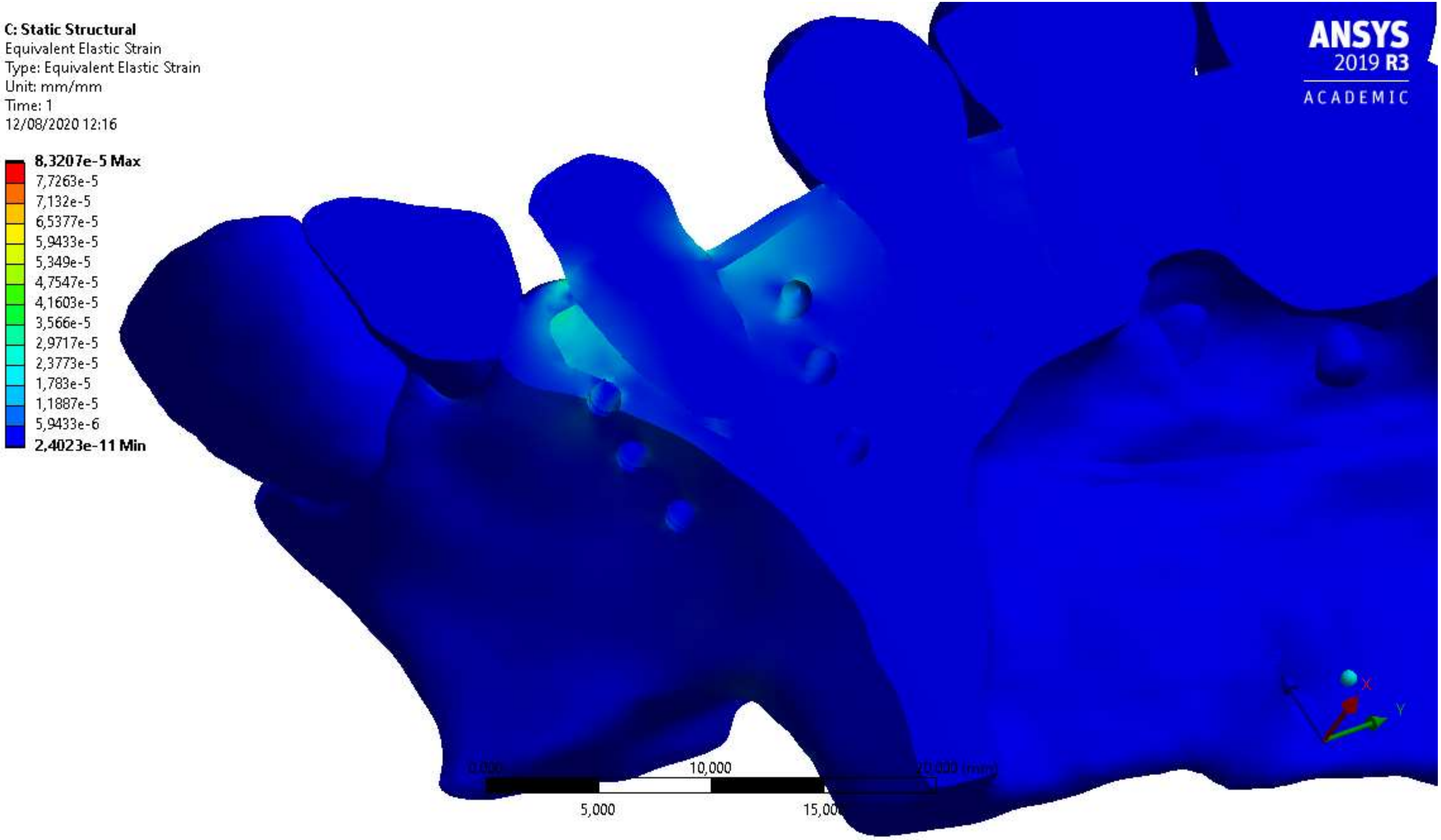

C: Static Structural
Equivalent Elastic Strain
Type: Equivalent Elastic Strain
Unit: mm/mm
Time: 1
12/08/2020 12:16
8,3207e-5 Max
7,7263e-5
7,132e-5
6,5377e-5
5,9433e-5
5,349e-5
4,7547e-5
4,1603e-5
3,566e-5
2,9717e-5
2,3773e-5
1,783e-5
1,1887e-5
5,9433e-6
2,4023e-11 Min
ANSYS
2019 R3
ACADEMIC
10,000
5,000
X
Y

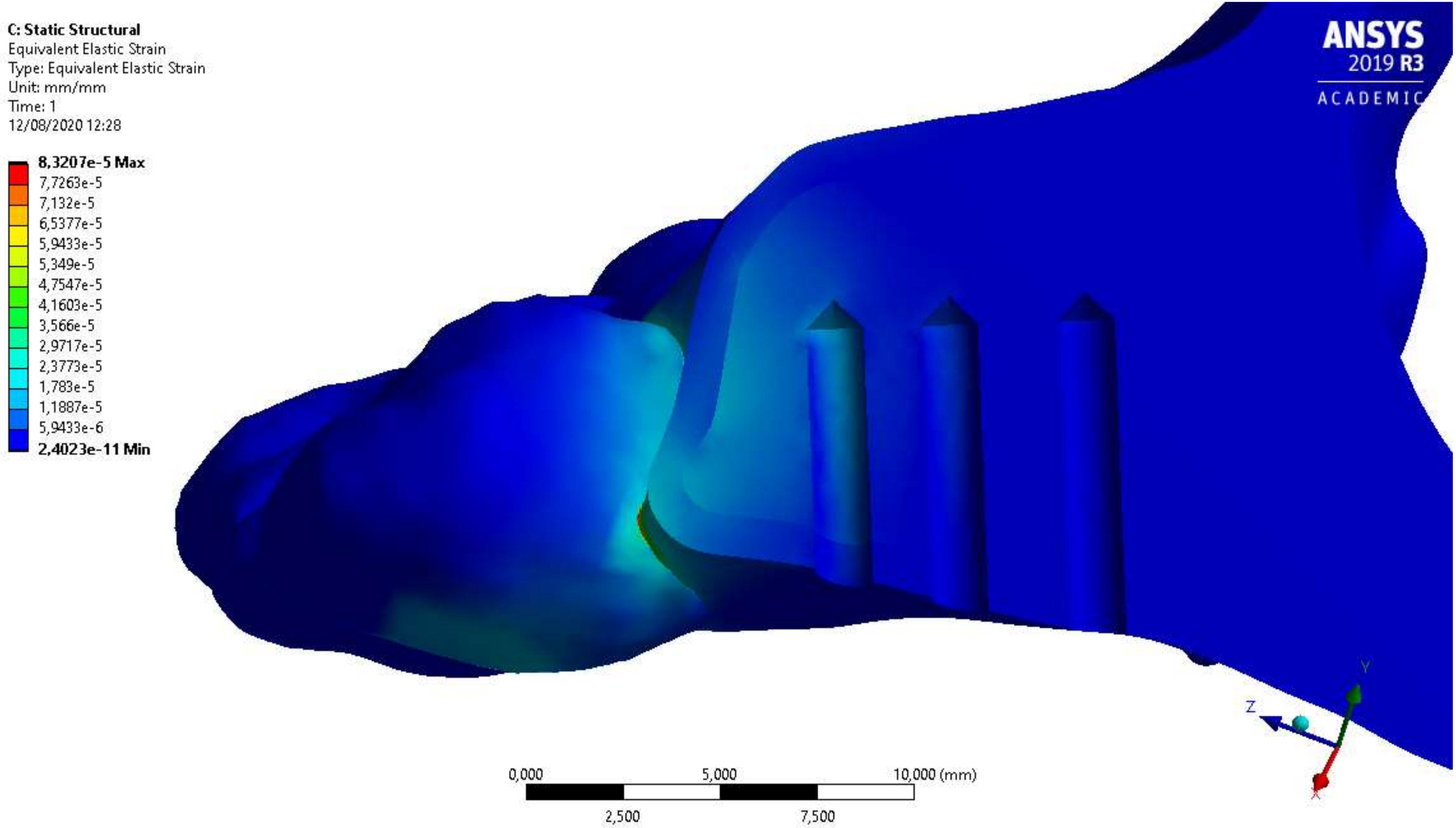
C: Static Structural
Equivalent Elastic Strain
Type: Equivalent Elastic Strain
Unit: mm/mm
Time: 1
12/08/2020 12:28
8,3207e-5 Max
7,7263e-5
7,132e-5
6,5377e-5
5,9433e-5
5,349e-5
4,7547e-5
4,1603e-5
3,566e-5
2,9717e-5
2,3773e-5
1,783e-5
1,1887e-5
5,9433e-6
2,4023e-11 Min
ANSYS
2019 R3
ACADEMIC
0,000
5,000
10,000 (mm)
2,500
7,500
Y
Z
X

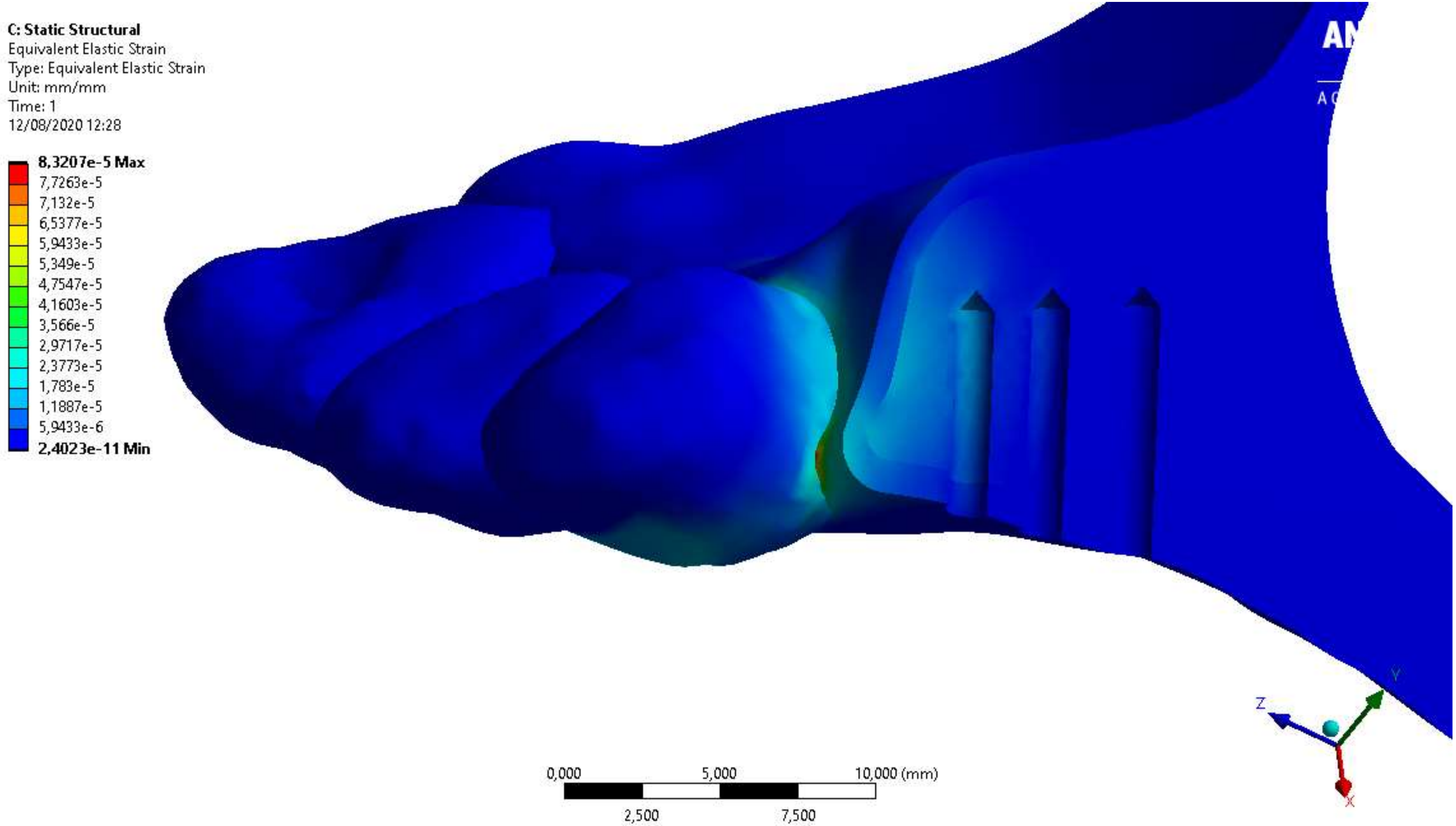
C: Static Structural
Equivalent Elastic Strain
Type: Equivalent Elastic Strain
Unit: mm/mm
Time: 1
12/08/2020 12:28
8,3207e-5 Max
7,7263e-5
7,132e-5
6,5377e-5
5,9433e-5
5,349e-5
4,7547e-5
4,1603e-5
3,566e-5
2,9717e-5
2,3773e-5
1,783e-5
1,1887e-5
5,9433e-6
2,4023e-11 Min
0,000
5,000
10,000 (mm)
2,500
7,500
Z
Y
X

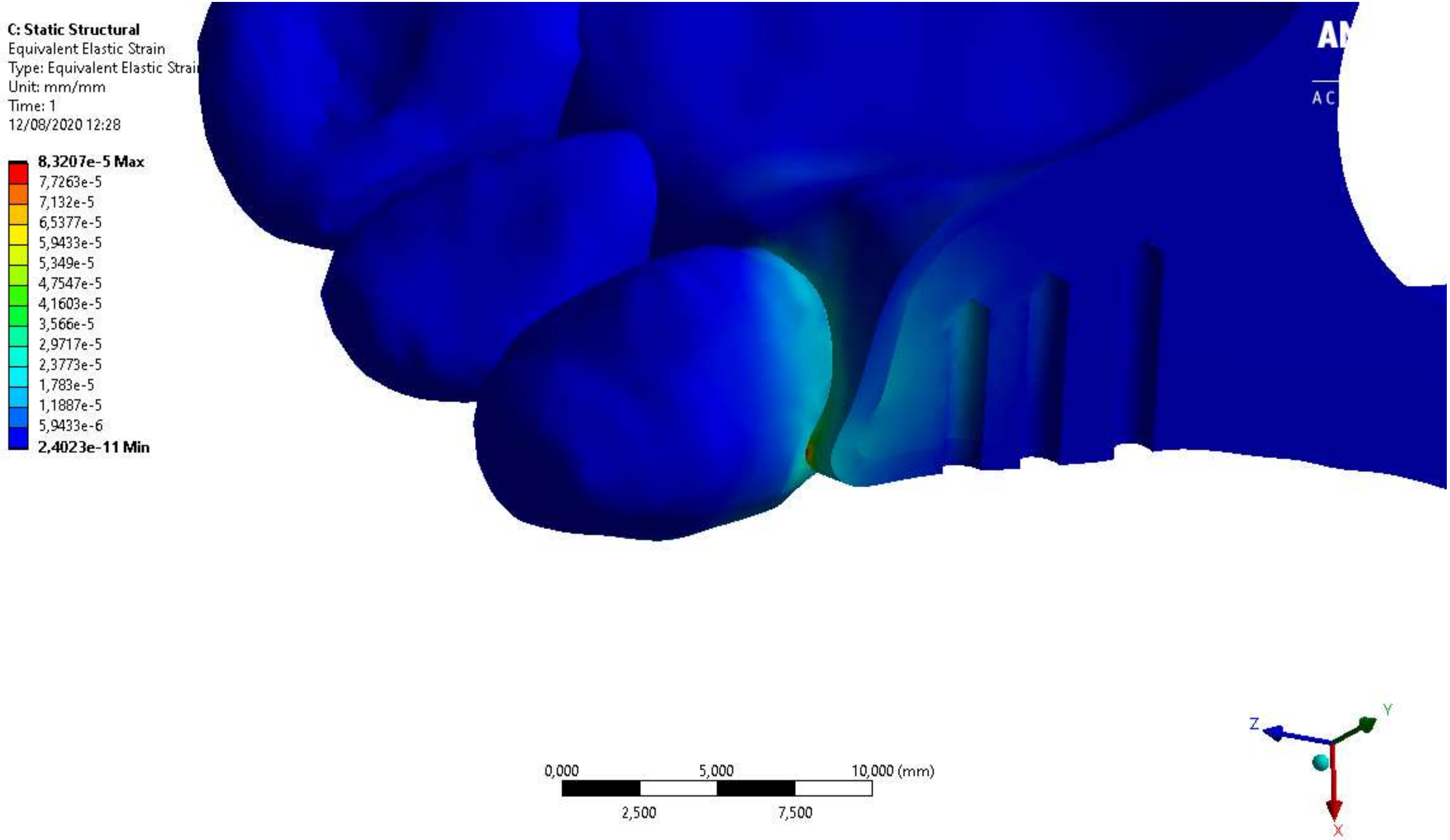
C: Static Structural
Equivalent Elastic Strain
Type: Equivalent Elastic Strai
Unit: mm/mm
Time: 1
12/08/2020 12:28
8,3207e-5 Max
7,7263e-5
7,132e-5
6,5377e-5
5,9433e-5
5,349e-5
4,7547e-5
4,1603e-5
3,566e-5
2,9717e-5
2,3773e-5
1,783e-5
1,1887e-5
5,9433e-6
2,4023e-11 Min
0,000
5,000
10,000 (mm)
2,500
7,500
Z
Y
X

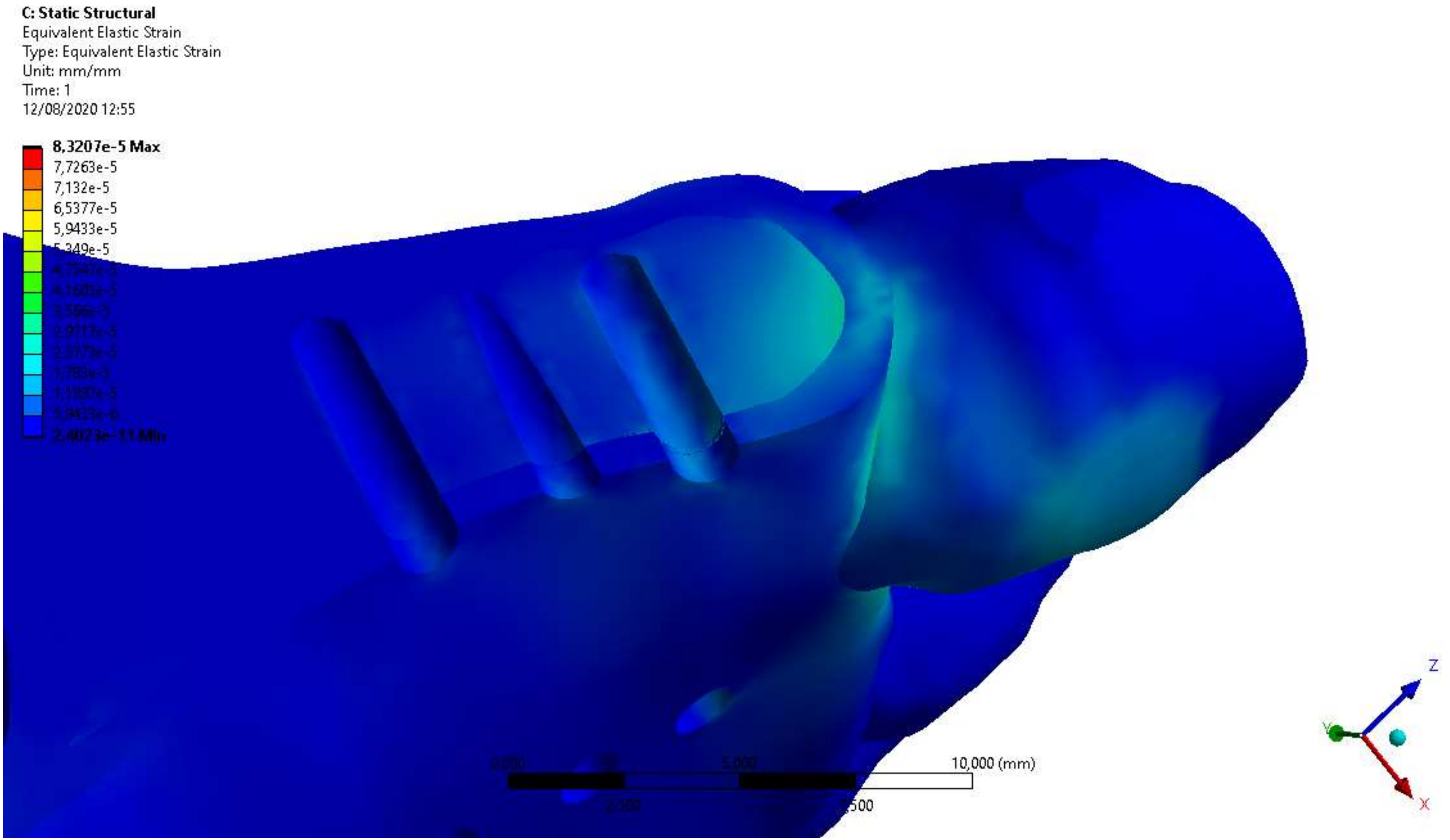
C: Static Structural
Equivalent Elastic Strain
Type: Equivalent Elastic Strain
Unit: mm/mm
Time: 1
12/08/2020 12:55
8,3207e-5 Max
7,7263e-5
7,132e-5
6,5377e-5
5,9433e-5
5,000
10,000 (mm)
Z
X

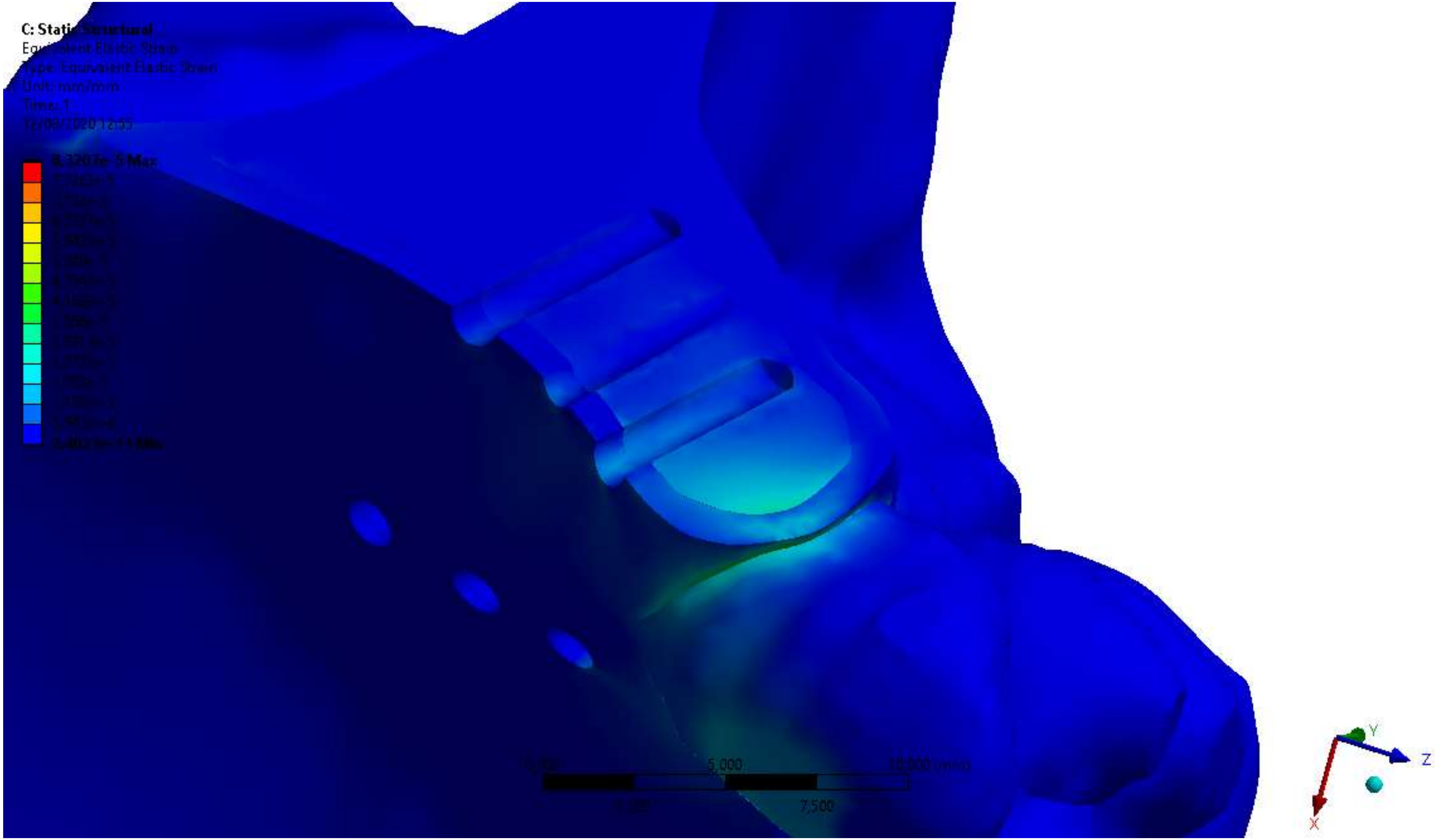
C: Static Structural
Equivalent Elastic Strain
Type: Equivalent Elastic Strain
Unit: mm/mm
Time: 1
5,000
10,000 (mm)
7,500
Y
Z
X

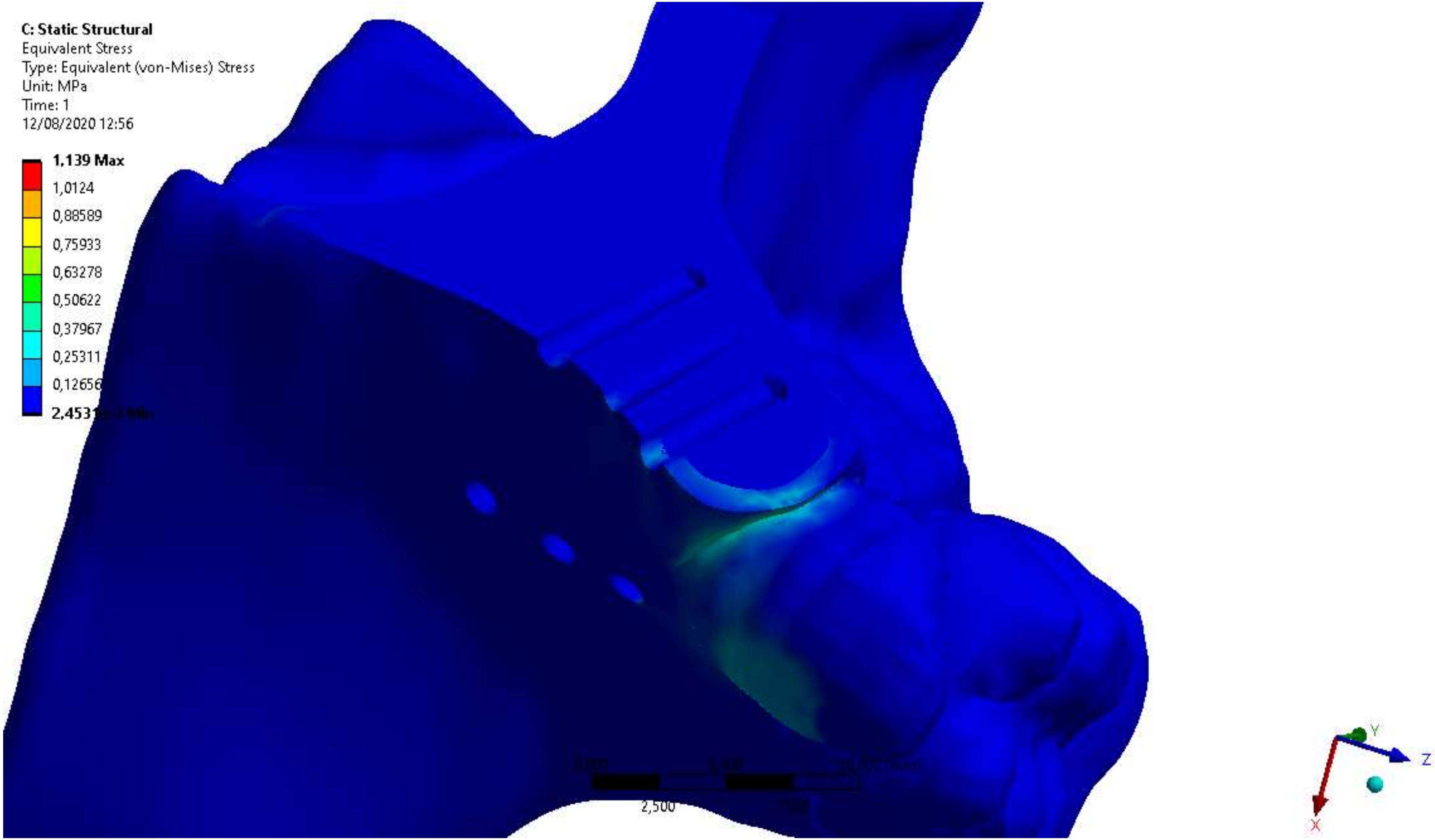
C: Static Structural
Equivalent Stress
Type: Equivalent (von-Mises) Stress
Unit: MPa
Time: 1
12/08/2020 12:56
1,139 Max
1,0124
0,88589
0,75933
0,63278
0,50622
0,37967
0,25311
0,12656
2,500
Y
Z
X

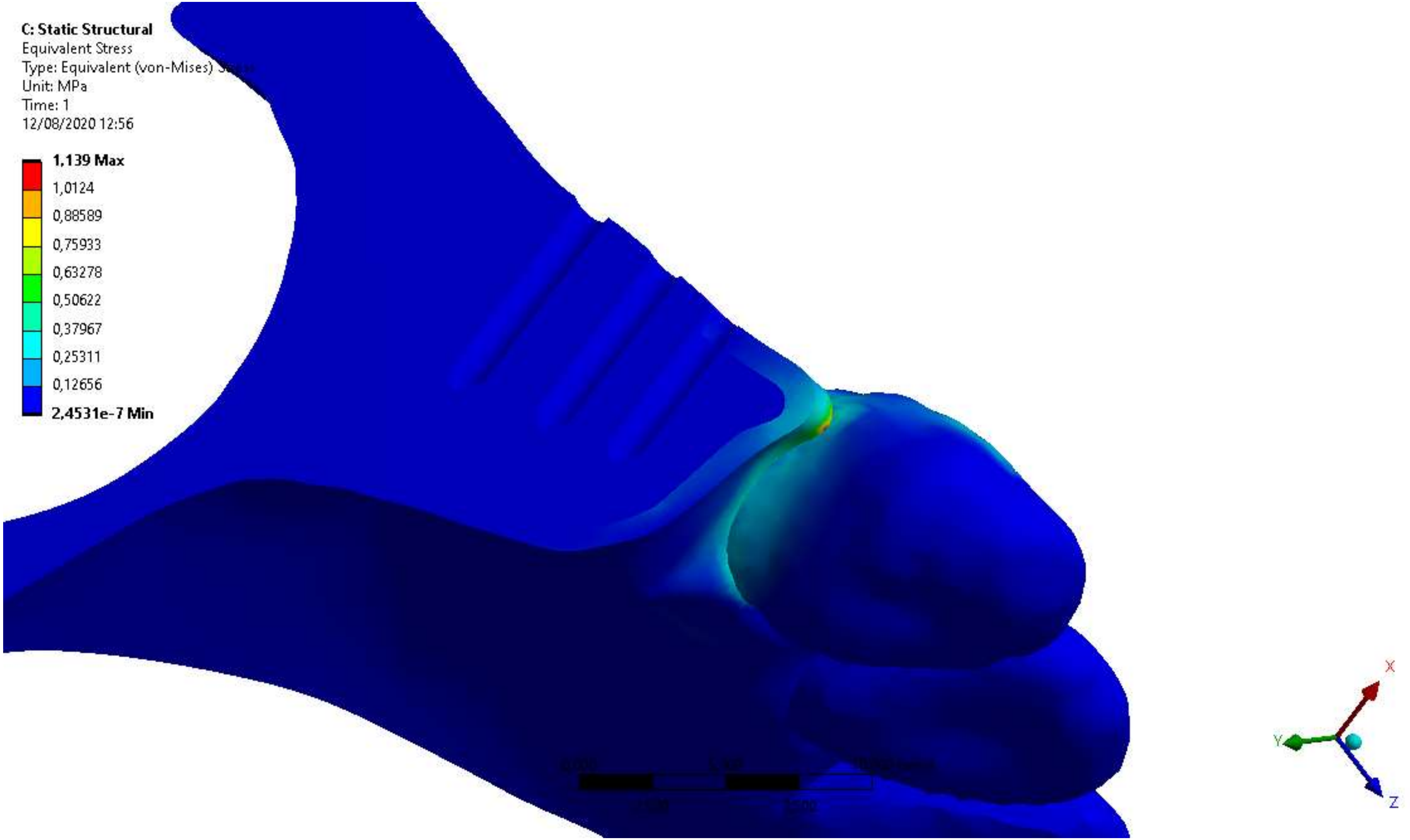
C: Static Structural
Equivalent Stress
Type: Equivalent (von-Mises)
Unit: MPa
Time: 1
12/08/2020 12:56
1,139 Max
1,0124
0,88589
0,75933
0,63278
0,50622
0,37967
0,25311
0,12656
2,4531e-7 Min
X
Y
Z

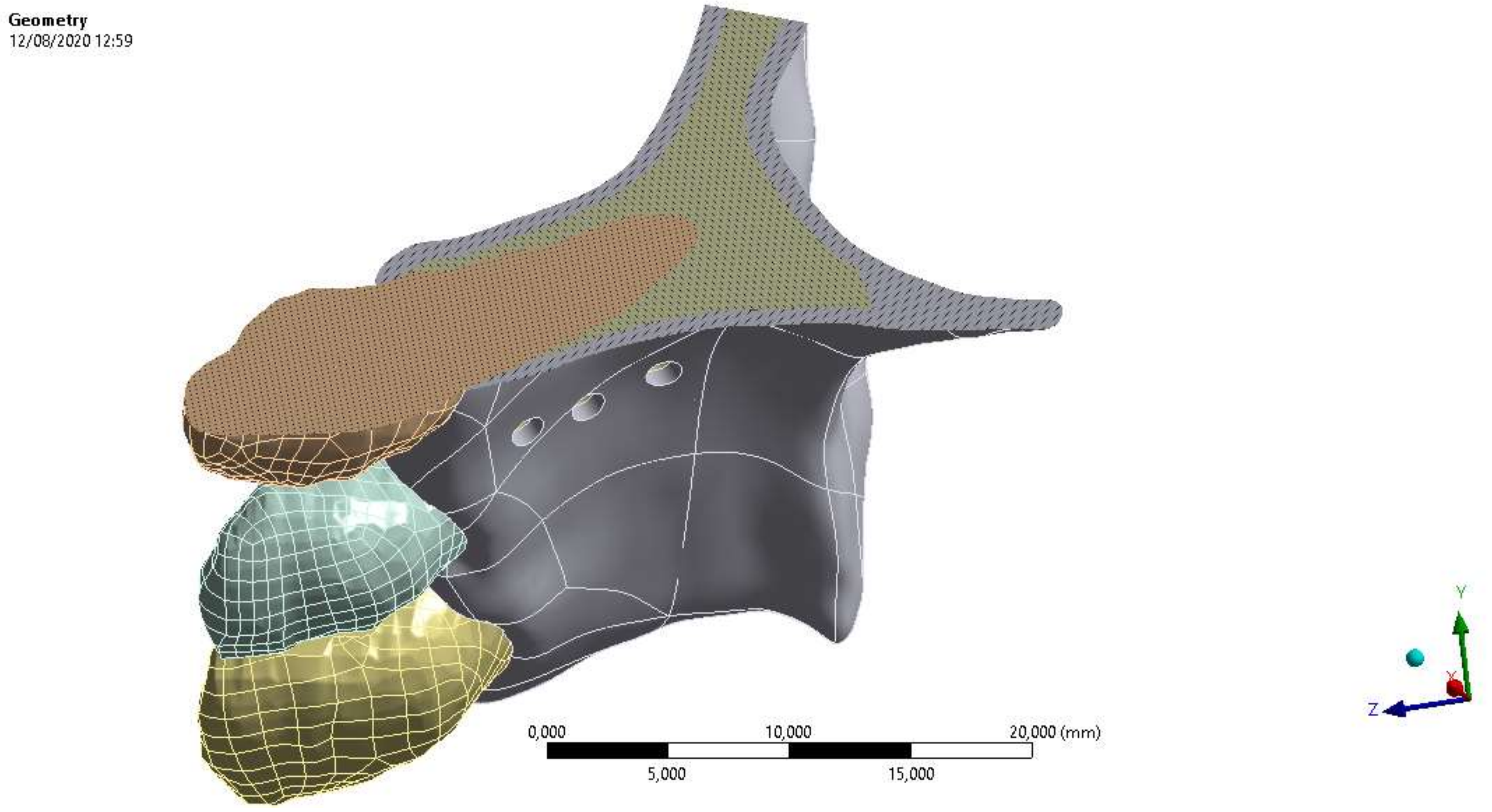
Geometry
12/08/2020 12:59
0,000
10,000
20,000 (mm)
5,000
15,000
Y
X
Z

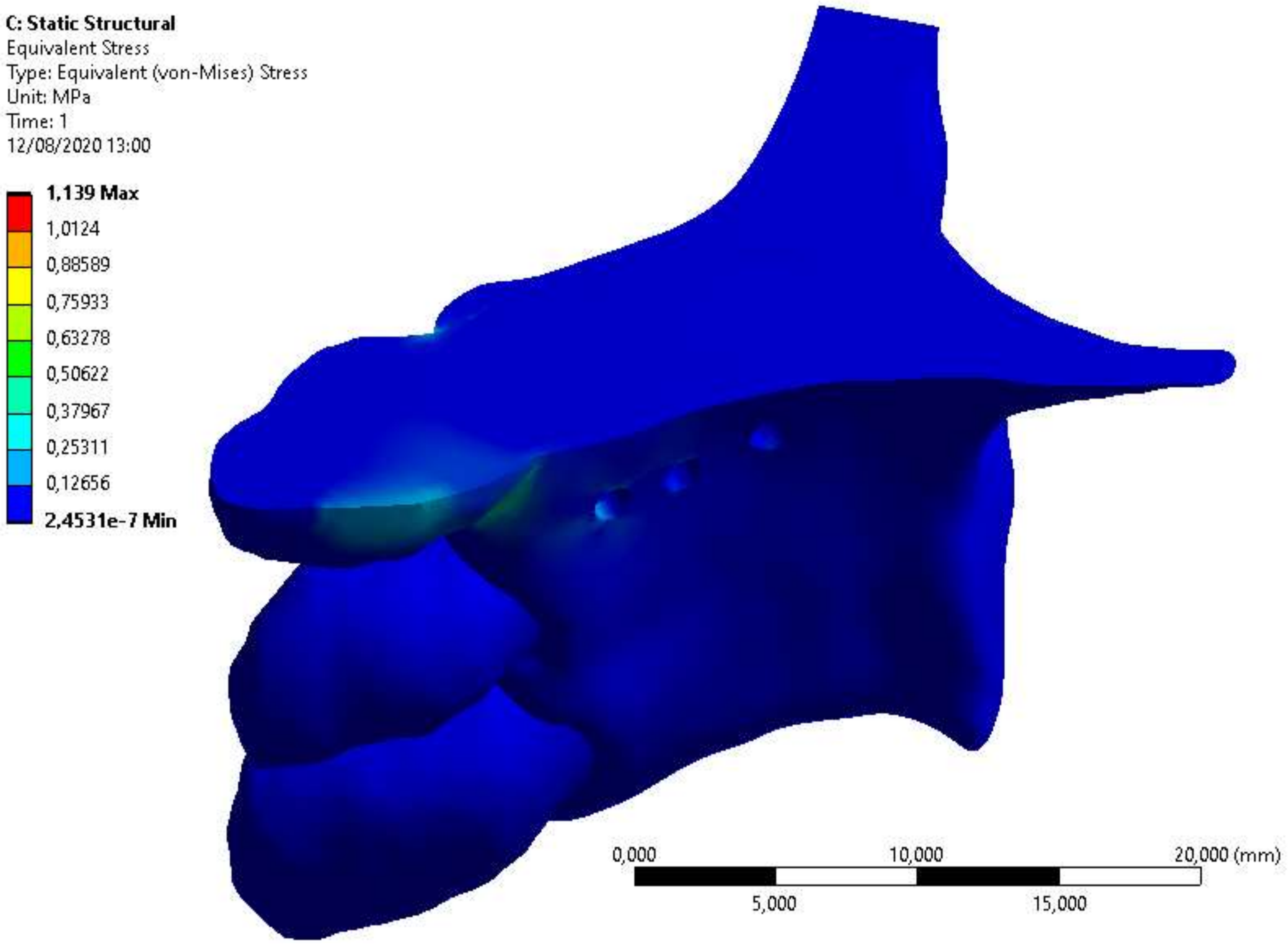
C: Static Structural
Equivalent Stress
Type: Equivalent (von-Mises) Stress
Unit: MPa
Time: 1
12/08/2020 13:00
1,139 Max
1,0124
0,88589
0,75933
0,63278
0,50622
0,37967
0,25311
0,12656
2,4531e-7 Min
0,000
10,000
20,000 (mm)
5,000
15,000
Y
X
Z

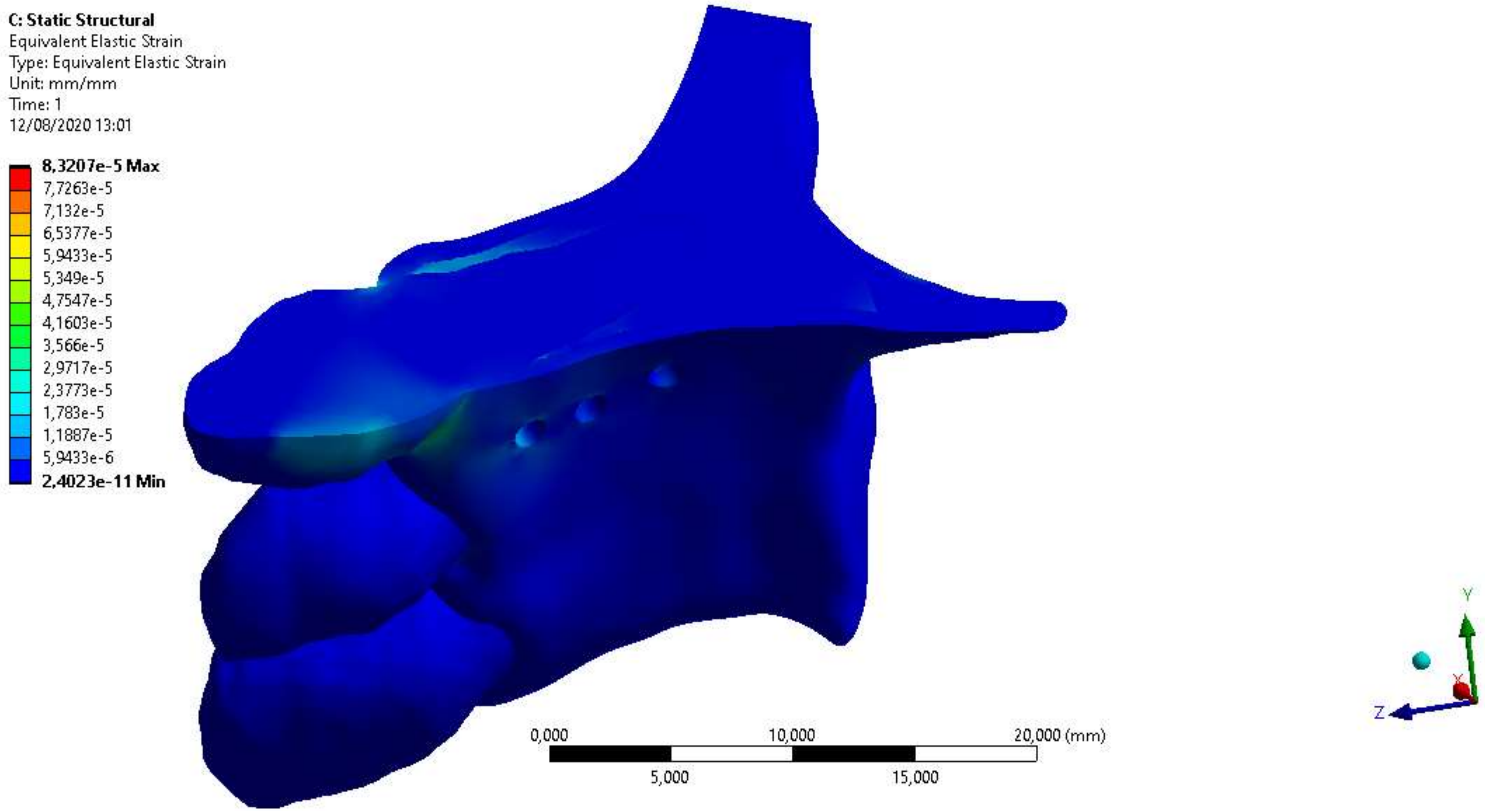
C: Static Structural
Equivalent Elastic Strain
Type: Equivalent Elastic Strain
Unit: mm/mm
Time: 1
12/08/2020 13:01
8,3207e-5 Max
7,7263e-5
7,132e-5
6,5377e-5
5,9433e-5
5,349e-5
4,7547e-5
4,1603e-5
3,566e-5
2,9717e-5
2,3773e-5
1,783e-5
1,1887e-5
5,9433e-6
2,4023e-11 Min
0,000
10,000
20,000 (mm)
5,000
15,000
Y
Z
X

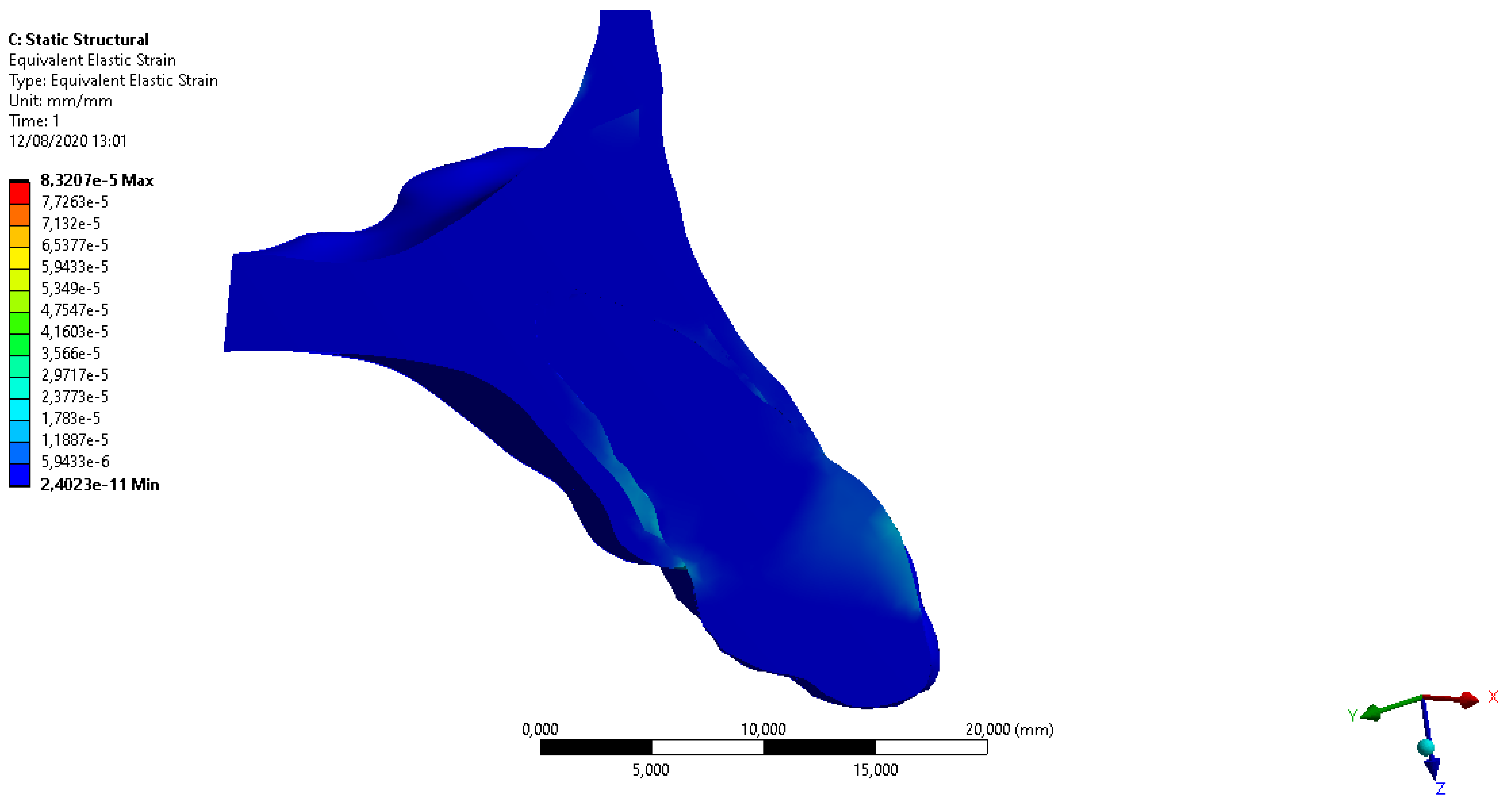
C: Static Structural
Equivalent Elastic Strain
Type: Equivalent Elastic Strain
Unit: mm/mm
Time: 1
12/08/2020 13:01
8,3207e-5 Max
7,7263e-5
7,132e-5
6,5377e-5
5,9433e-5
5,349e-5
4,7547e-5
4,1603e-5
3,566e-5
2,9717e-5
2,3773e-5
1,783e-5
1,1887e-5
5,9433e-6
2,4023e-11 Min
0,000
10,000
20,000 (mm)
5,000
15,000
Y
X
Z

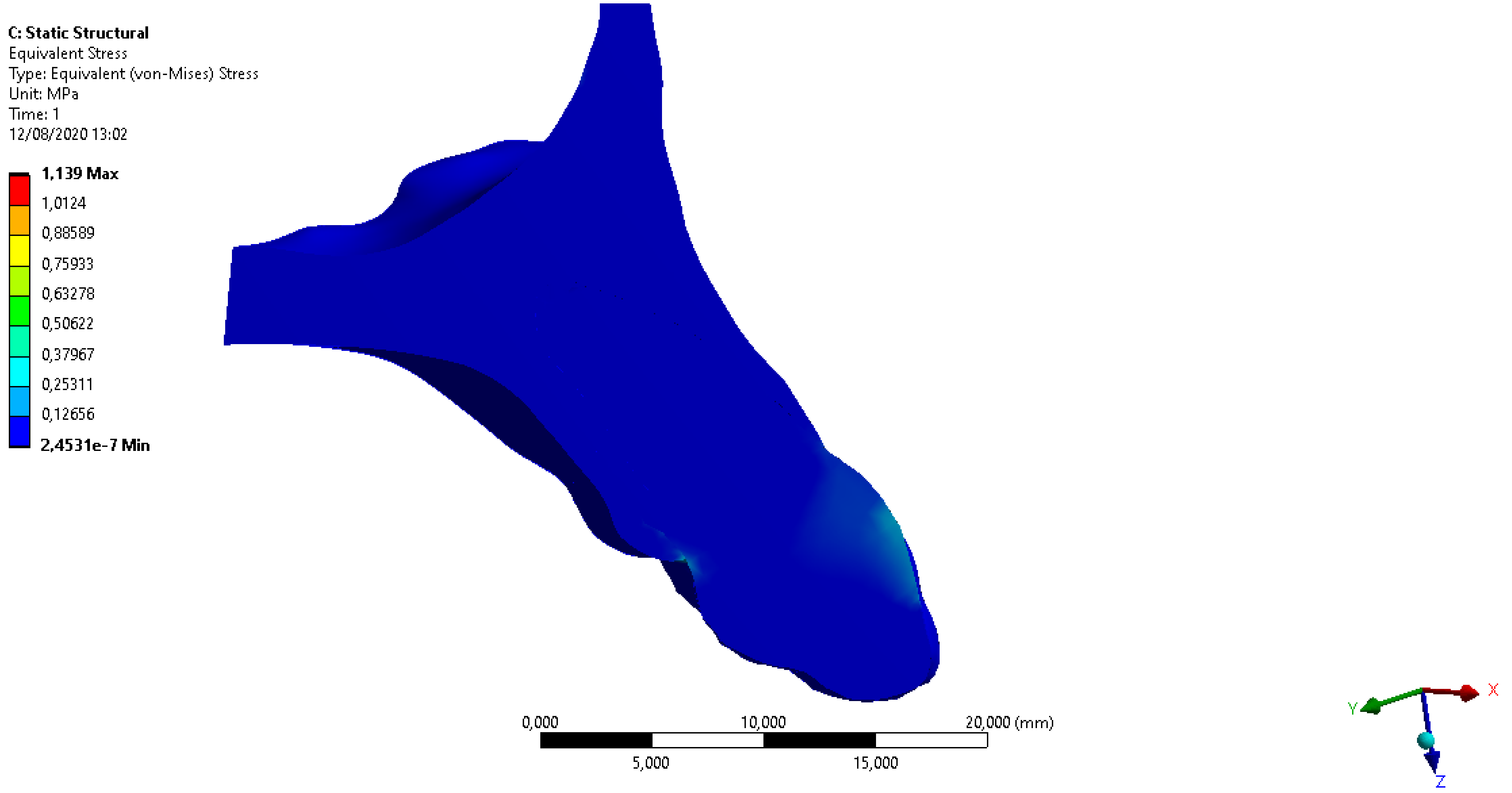
C: Static Structural
Equivalent Stress
Type: Equivalent (von-Mises) Stress
Unit: MPa
Time: 1
12/08/2020 13:02
1,139 Max
1,0124
0,88589
0,75933
0,63278
0,50622
0,37967
0,25311
0,12656
2,4531e-7 Min
0,000
10,000
20,000 (mm)
5,000
15,000
X
Y
Z

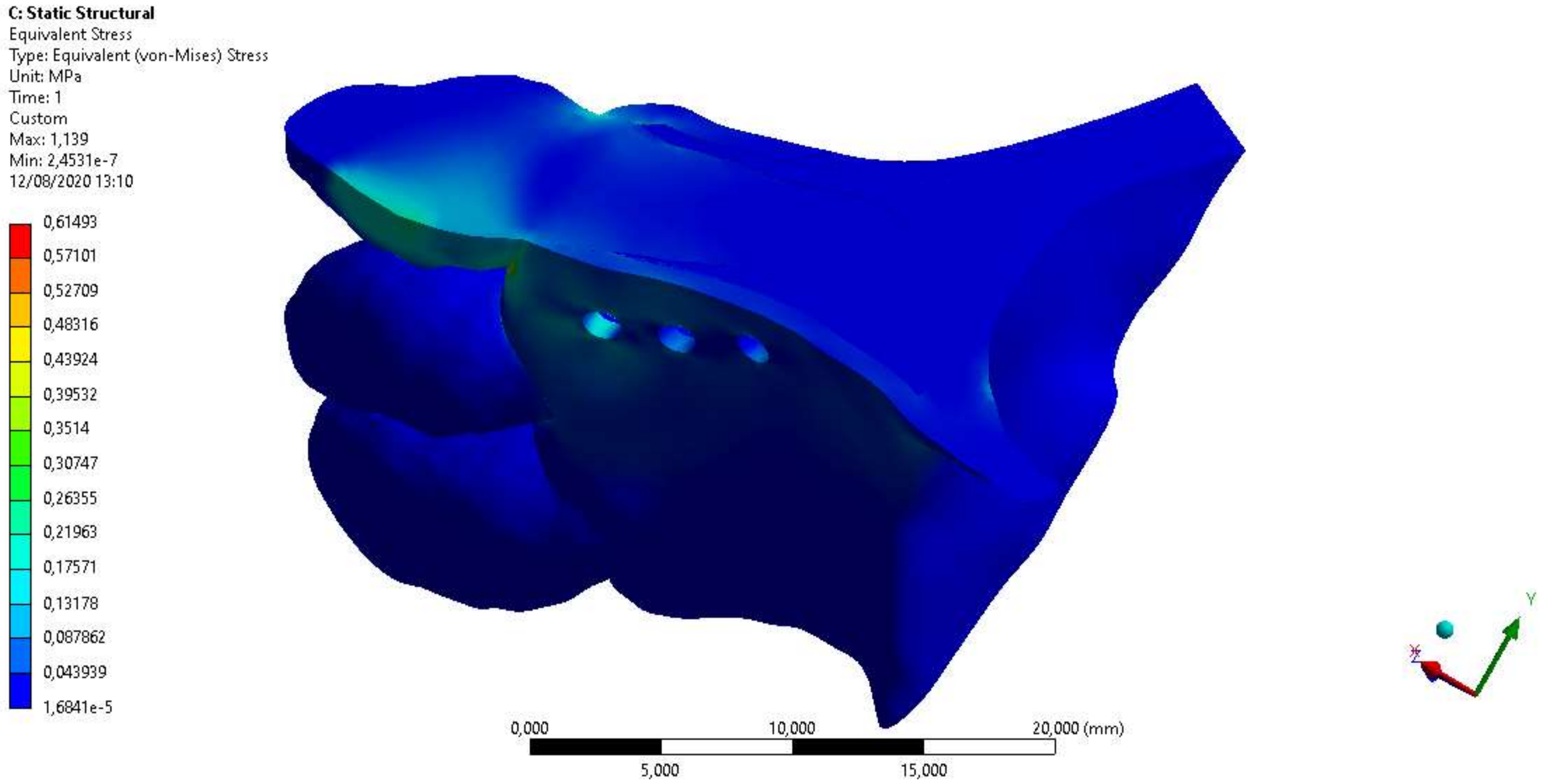
C: Static Structural
Equivalent Stress
Type: Equivalent (von-Mises) Stress
Unit: MPa
Time: 1
Custom
Max: 1,139
Min: 2,4531e-7
12/08/2020 13:10
0,61493
0,57101
0,52709
0,48316
0,43924
0,39532
0,3514
0,30747
0,26355
0,21963
0,17571
0,13178
0,087862
0,043939
1,6841e-5
0,000
10,000
20,000 (mm)
5,000
15,000
Y

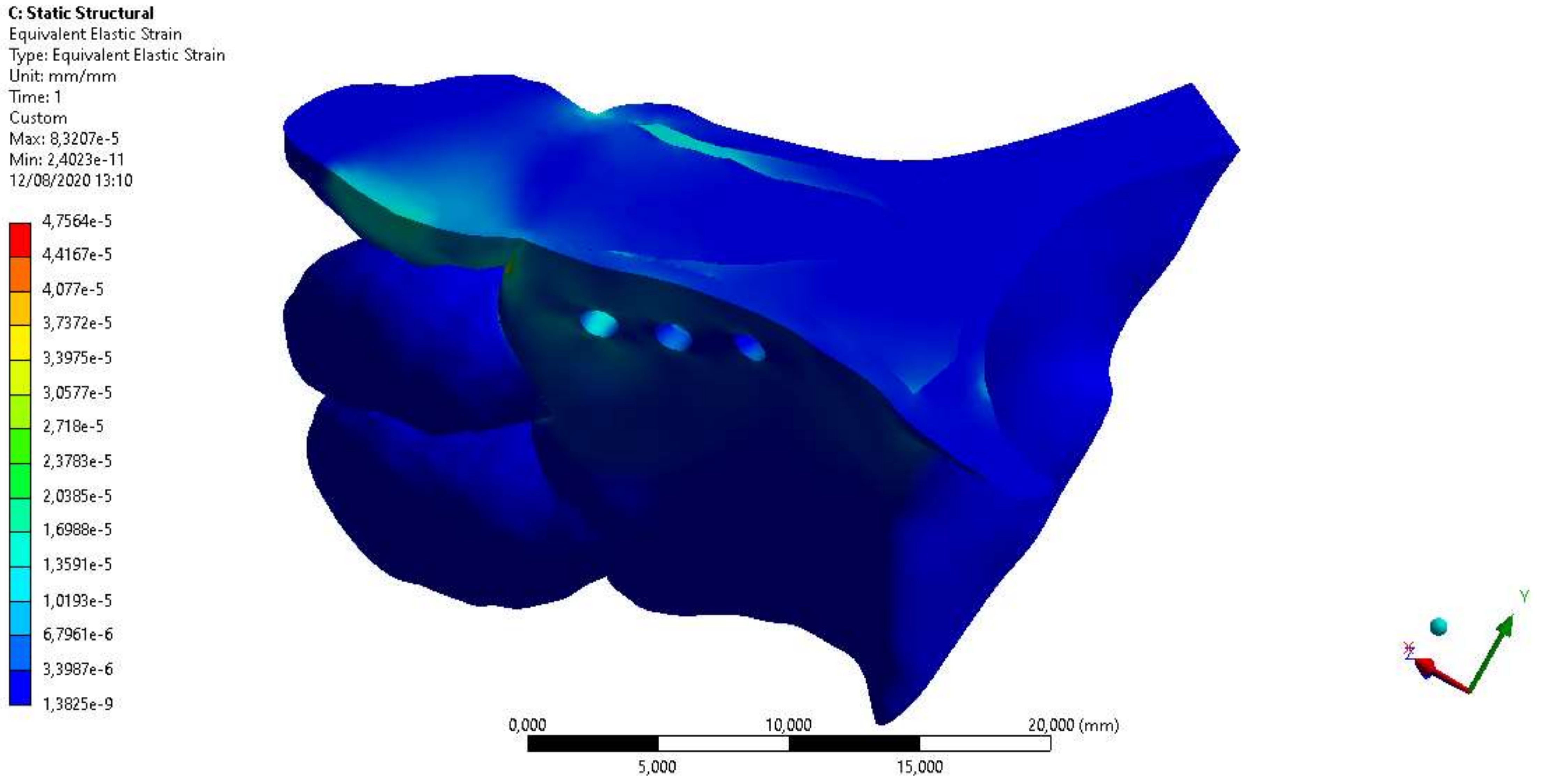
C: Static Structural
Equivalent Elastic Strain
Type: Equivalent Elastic Strain
Unit: mm/mm
Time: 1
Custom
Max: 8,3207e-5
Min: 2,4023e-11
12/08/2020 13:10
4,7564e-5
4,4167e-5
4,077e-5
3,7372e-5
3,3975e-5
3,0577e-5
2,718e-5
2,3783e-5
2,0385e-5
1,6988e-5
1,3591e-5
1,0193e-5
6,7961e-6
3,3987e-6
1,3825e-9
0,000
10,000
20,000 (mm)
5,000
15,000
Y

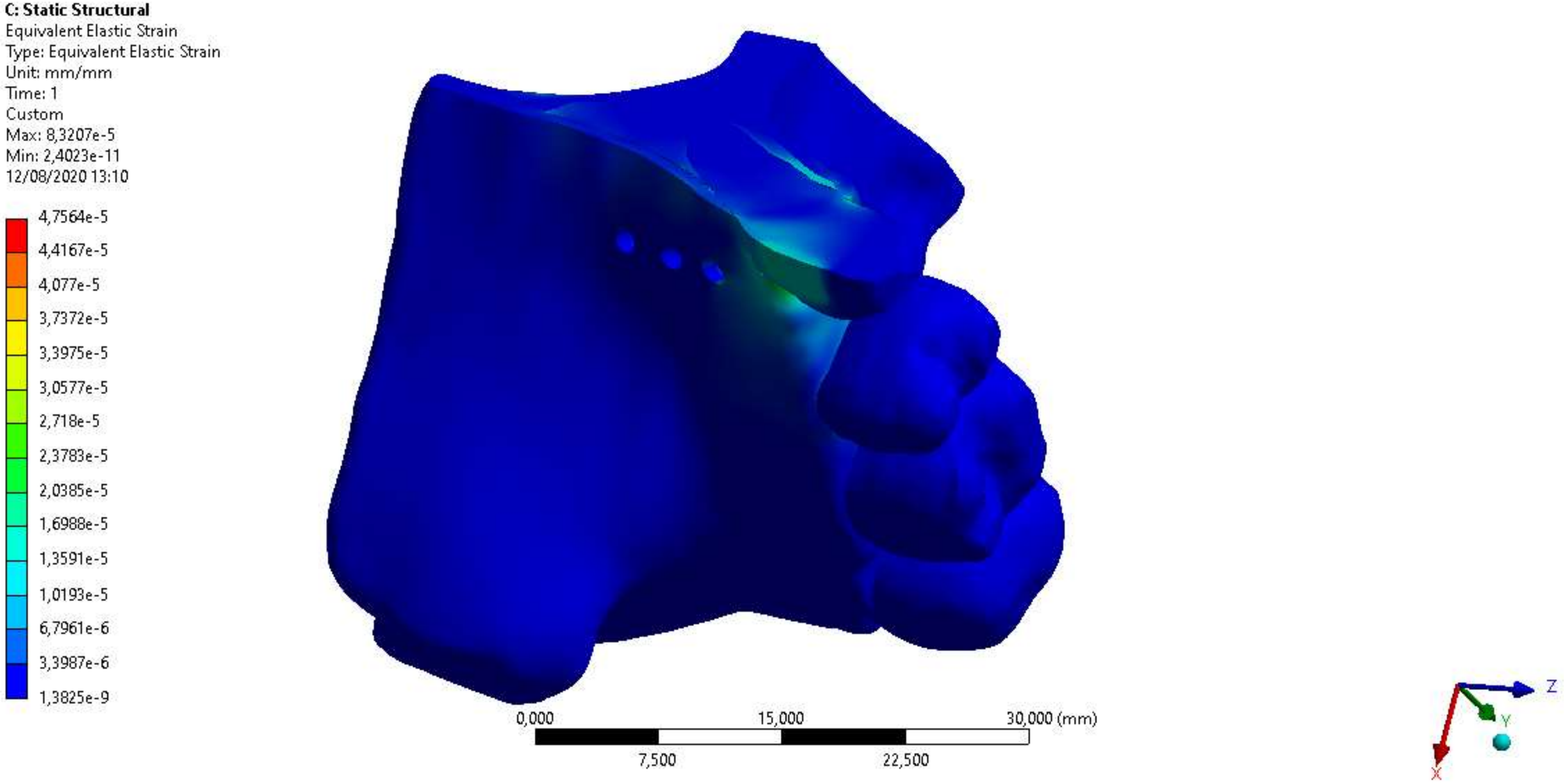
C: Static Structural
Equivalent Elastic Strain
Type: Equivalent Elastic Strain
Unit: mm/mm
Time: 1
Custom
Max: 8,3207e-5
Min: 2,4023e-11
12/08/2020 13:10
4,7564e-5
4,4167e-5
4,077e-5
3,7372e-5
3,3975e-5
3,0577e-5
2,718e-5
2,3783e-5
2,0385e-5
1,6988e-5
1,3591e-5
1,0193e-5
6,7961e-6
3,3987e-6
1,3825e-9
0,000
15,000
30,000 (mm)
7,500
22,500
Z
Y
X

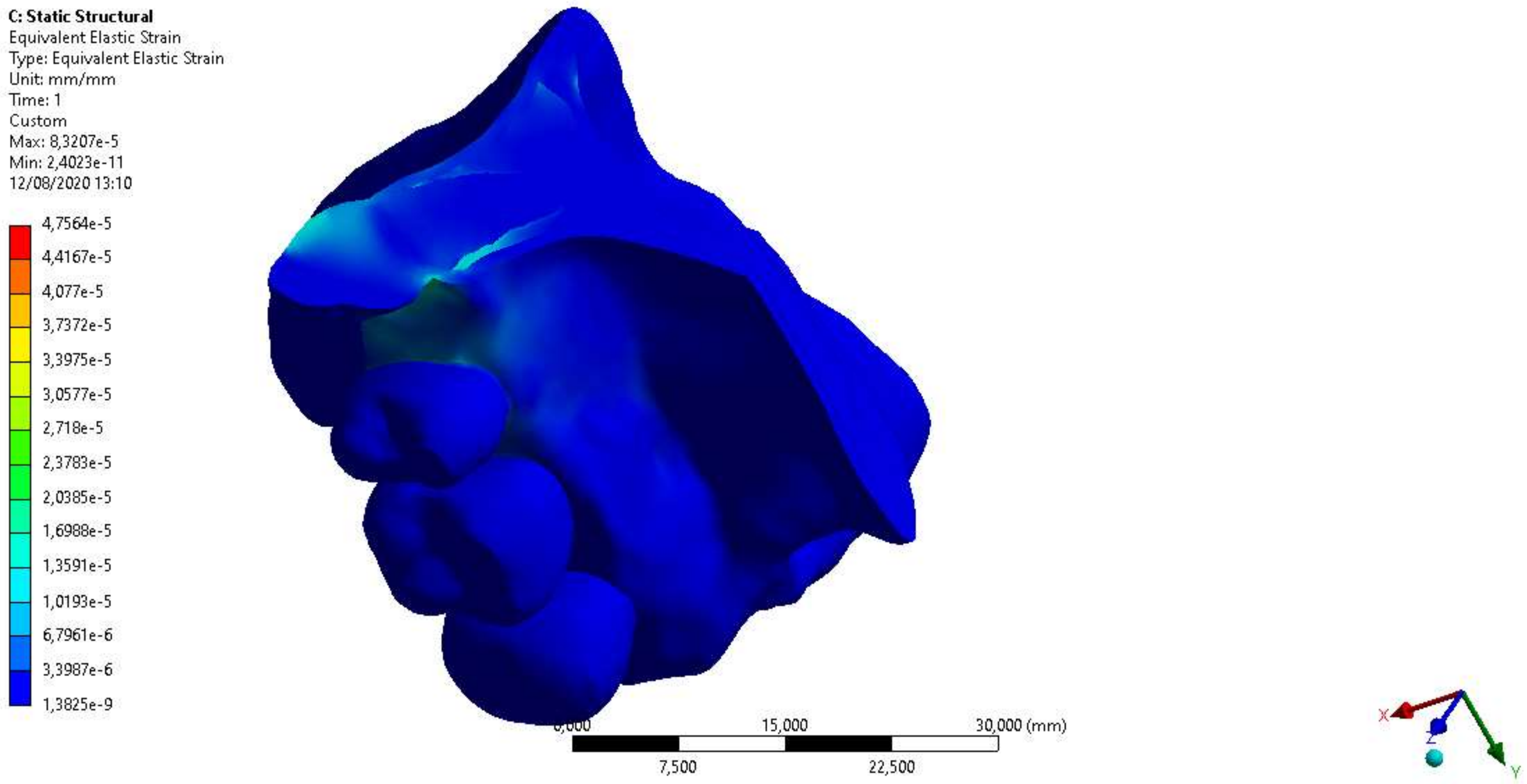
C: Static Structural
Equivalent Elastic Strain
Type: Equivalent Elastic Strain
Unit: mm/mm
Time: 1
Custom
Max: 8,3207e-5
Min: 2,4023e-11
12/08/2020 13:10
4,7564e-5
4,4167e-5
4,077e-5
3,7372e-5
3,3975e-5
3,0577e-5
2,718e-5
2,3783e-5
2,0385e-5
1,6988e-5
1,3591e-5
1,0193e-5
6,7961e-6
3,3987e-6
1,3825e-9
0,000
15,000
30,000 (mm)
7,500
22,500
X
Y
Z

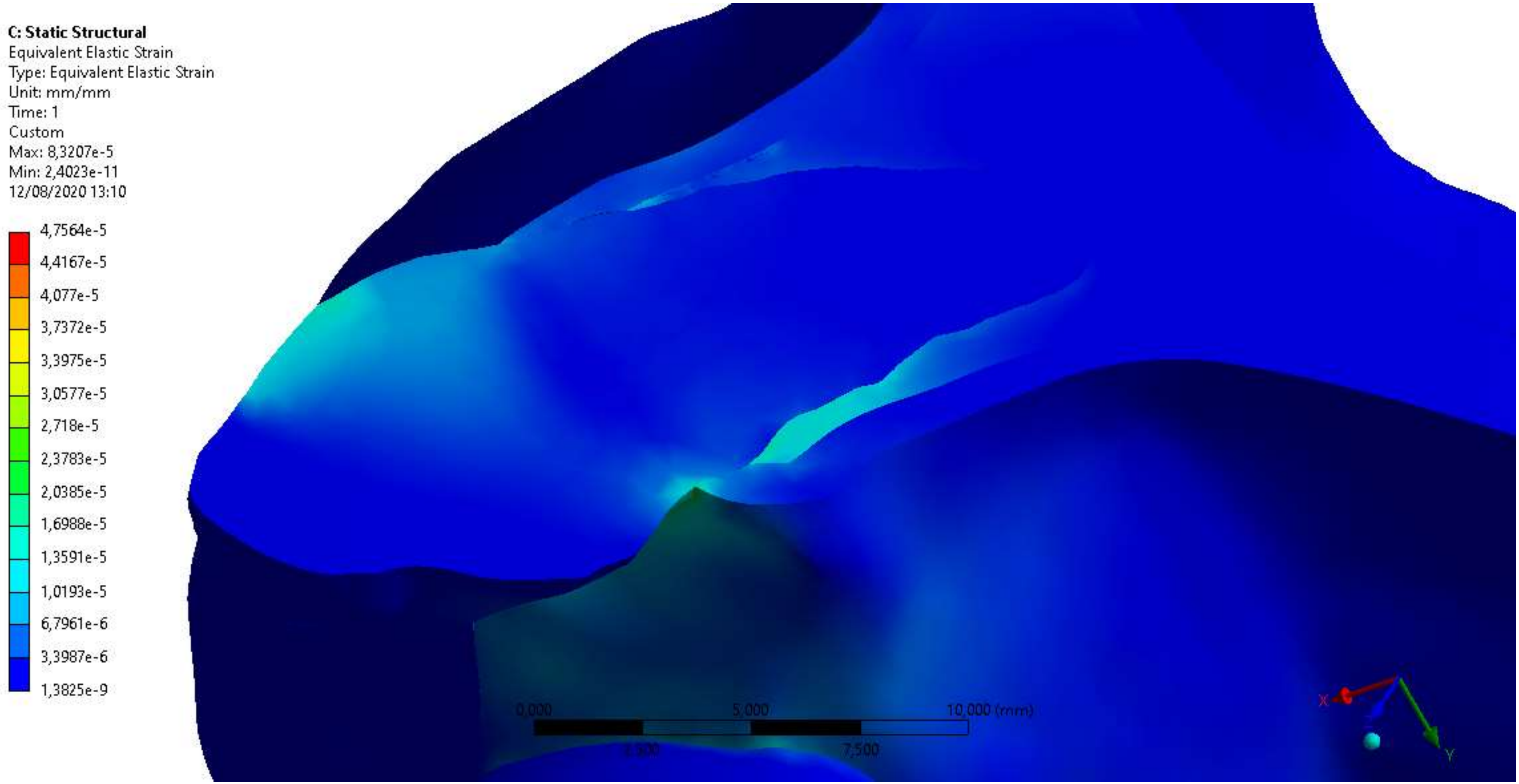
C: Static Structural
Equivalent Elastic Strain
Type: Equivalent Elastic Strain
Unit: mm/mm
Time: 1
Custom
Max: 8,3207e-5
Min: 2,4023e-11
12/08/2020 13:10
4,7564e-5
4,4167e-5
4,077e-5
3,7372e-5
3,3975e-5
3,0577e-5
2,718e-5
2,3783e-5
2,0385e-5
1,6988e-5
1,3591e-5
1,0193e-5
6,7961e-6
3,3987e-6
1,3825e-9
0,000
5,000
10,000 (mm)
7,500
X
Y

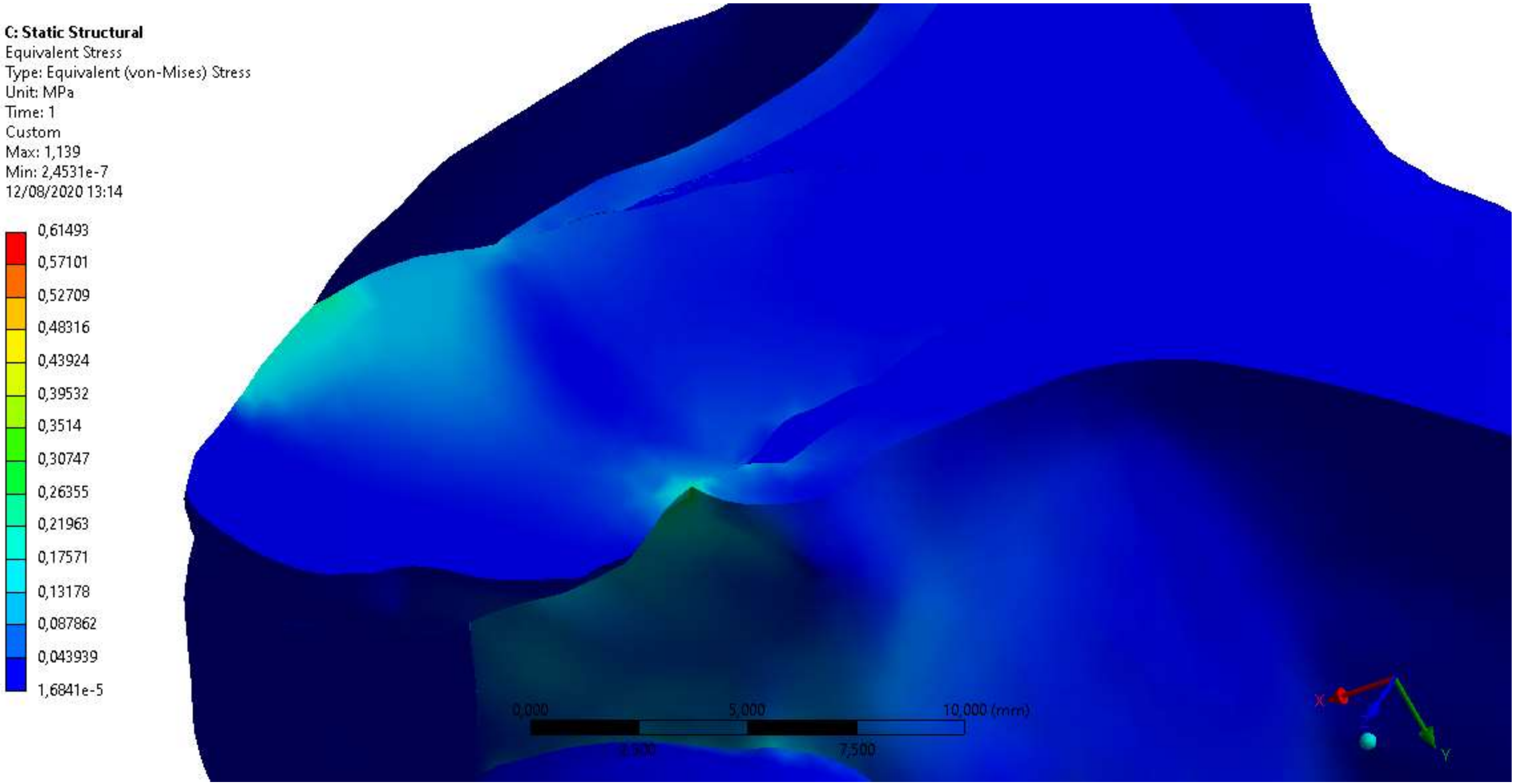
C: Static Structural
Equivalent Stress
Type: Equivalent (von-Mises) Stress
Unit: MPa
Time: 1
Custom
Max: 1,139
Min: 2,4531e-7
12/08/2020 13:14
0,61493
0,57101
0,52709
0,48316
0,43924
0,39532
0,3514
0,30747
0,26355
0,21963
0,17571
0,13178
0,087862
0,043939
1,6841e-5
0,000
5,000
10,000 (mm)
7,500
X
Y

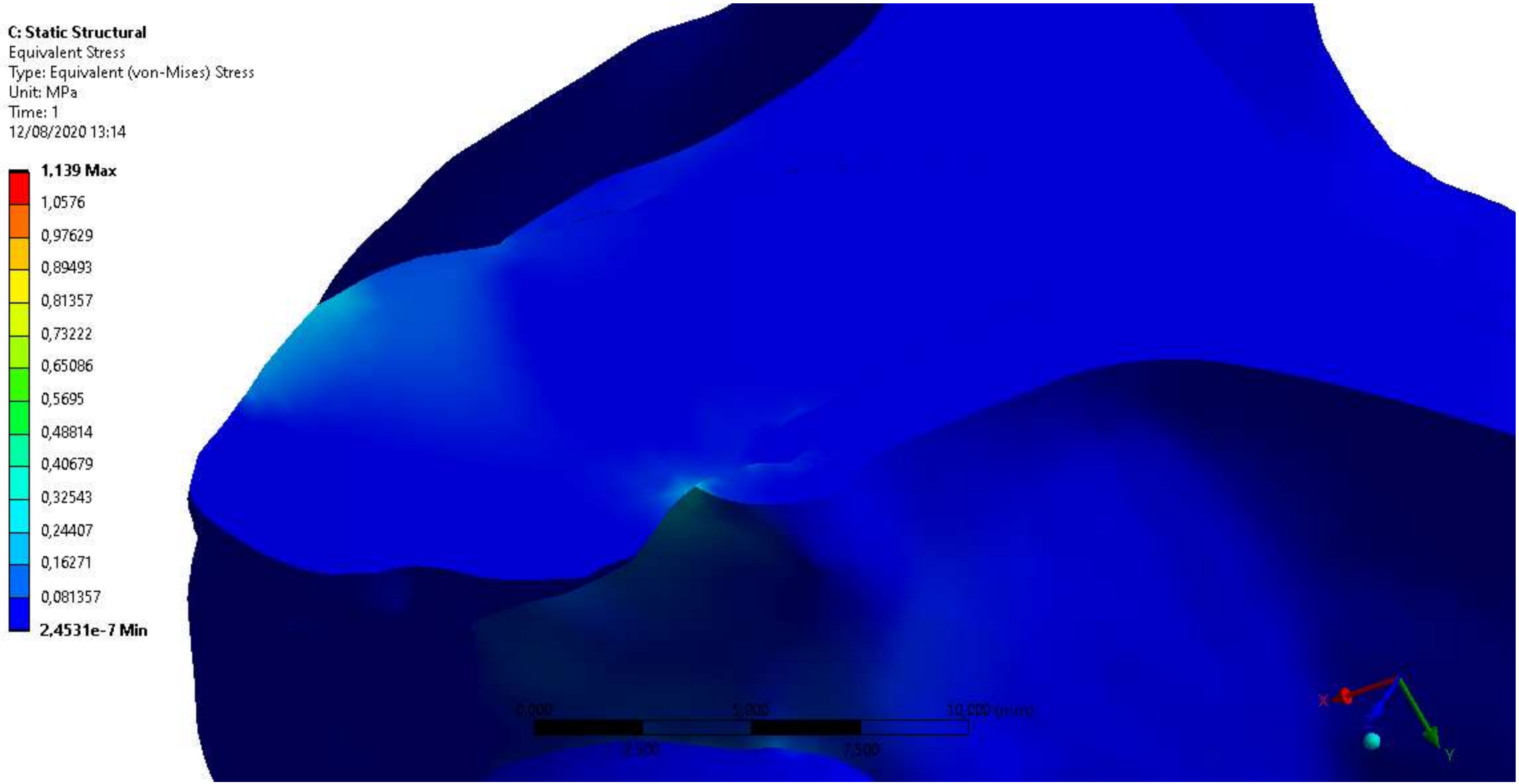
C: Static Structural
Equivalent Stress
Type: Equivalent (von-Mises) Stress
Unit: MPa
Time: 1
12/08/2020 13:14
1,139 Max
1,0576
0,97629
0,89493
0,81357
0,73222
0,65086
0,5695
0,48814
0,40679
0,32543
0,24407
0,16271
0,081357
2,4531e-7 Min

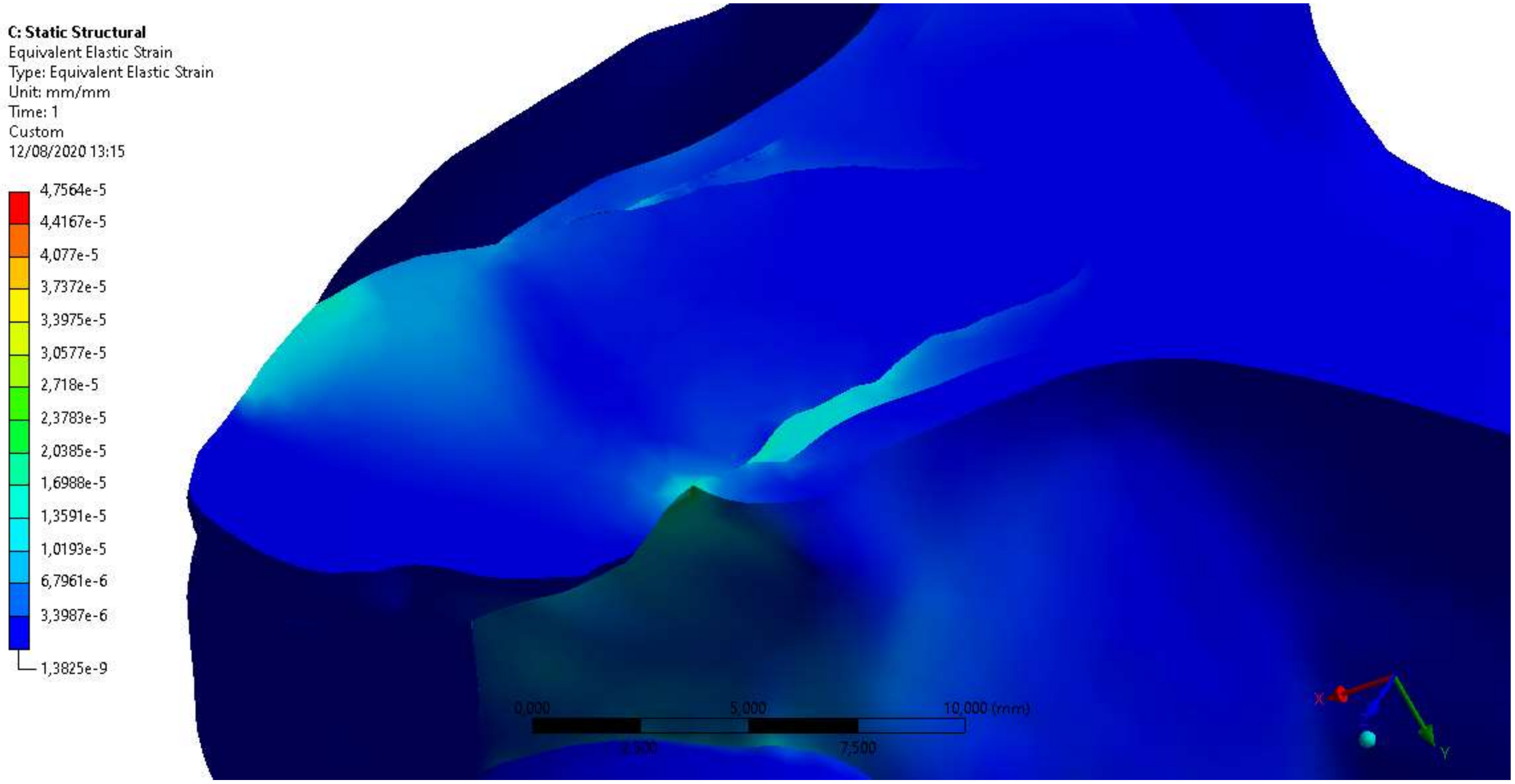
C: Static Structural
Equivalent Elastic Strain
Type: Equivalent Elastic Strain
Unit: mm/mm
Time: 1
Custom
12/08/2020 13:15
4,7564e-5
4,4167e-5
4,077e-5
3,7372e-5
3,3975e-5
3,0577e-5
2,718e-5
2,3783e-5
2,0385e-5
1,6988e-5
1,3591e-5
1,0193e-5
6,7961e-6
3,3987e-6
1,3825e-9
0,000
5,000
10,000 (mm)
7,500
X
Y

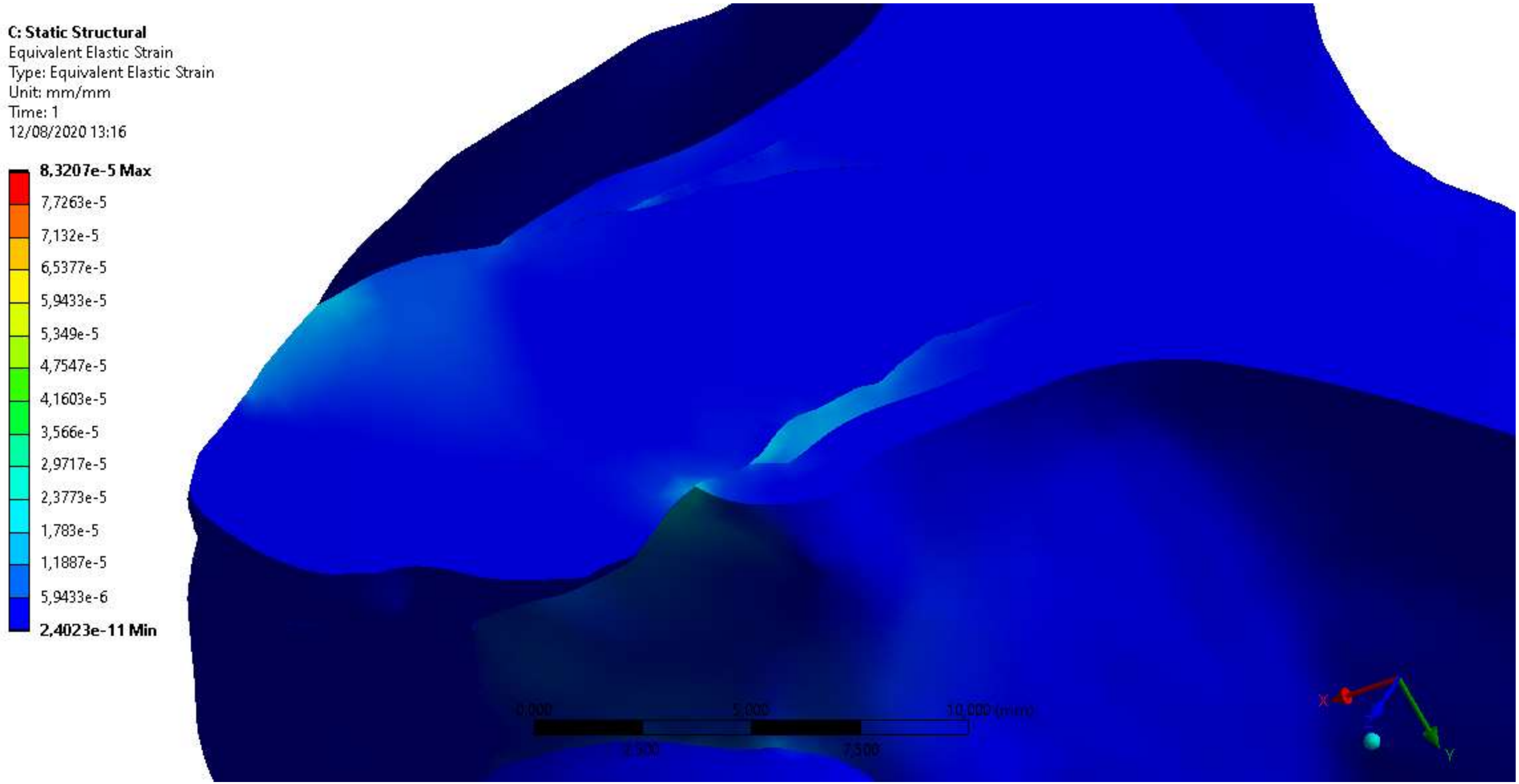

C: Static Structural
Equivalent Elastic Strain
Type: Equivalent Elastic Strain
Unit: mm/mm
Time: 1
12/08/2020 13:16
8,3207e-5 Max
7,7263e-5
7,132e-5
6,5377e-5
5,9433e-5
5,349e-5
4,7547e-5
4,1603e-5
3,566e-5
2,9717e-5
2,3773e-5
1,783e-5
1,1887e-5
5,9433e-6
2,4023e-11 Min
0,000
5,000
10,000 (mm)
7,500
X
Y
