# Supplementary material for: Micro-osteoperforation for enhancement of orthodontic movement: A mechanical analysis using the finite element method
Source: PLoS One. 2024 Aug 19;19(8):e0308739. doi: 10.1371/journal.pone.0308739 (PMC11332926; doi:10.1371/journal.pone.0308739)
Supplement: S3 File — (PDF) [file pone.0308739.s003.pdf]

S3. Analysis 1 without perforations

Dente

Fatigue Data at zero mean stress comes from 1998 ASME BPV Code, Section 8, Div 2, Table 5-110.1

Density	1,96e-06 kg/mm³

Structural

Isotropic Elasticity

Derive from	Young's Modulus and Poisson's Ratio
Young's Modulus	14700 MPa
Poisson's Ratio	0,31000
Bulk Modulus	12895 MPa
Shear Modulus	5610,7 MPa
Isotropic Secant Coefficient of Thermal Expansion	1,2e-05 1/°C
Compressive Ultimate Strength	0 MPa
Compressive Yield Strength	250,00 MPa

Osso Medular

Density	4,1e-07 kg/mm³

Structural

Isotropic Elasticity

Derive from	Young's Modulus and Poisson's Ratio
Young's Modulus	1370,0 MPa
Poisson's Ratio	0,30000
Bulk Modulus	1141,7 MPa
Shear Modulus	526,92 MPa

Osso Cortical Isotropico

Density	1,99e-06 kg/mm^3

Structural

Isotropic Elasticity

Derive from	Young's Modulus and Poisson's Ratio
Young's Modulus	13700 MPa
Poisson's Ratio	0,30000
Bulk Modulus	11417 MPa
Shear Modulus	5269,2 MPa

LigamentoPeriodotal

Fatigue Data at zero mean stress comes from 1998 ASME BPV Code, Section 8, Div 2, Table 5-110.1

Density	1,2e-06 kg/mm^3

Structural

Isotropic Elasticity

Derive from	Young's Modulus and Poisson's Ratio
Young's Modulus	0,068000 MPa
Poisson's Ratio	0,45000
Bulk Modulus	0,22667 MPa
Shear Modulus	0,023448 MPa

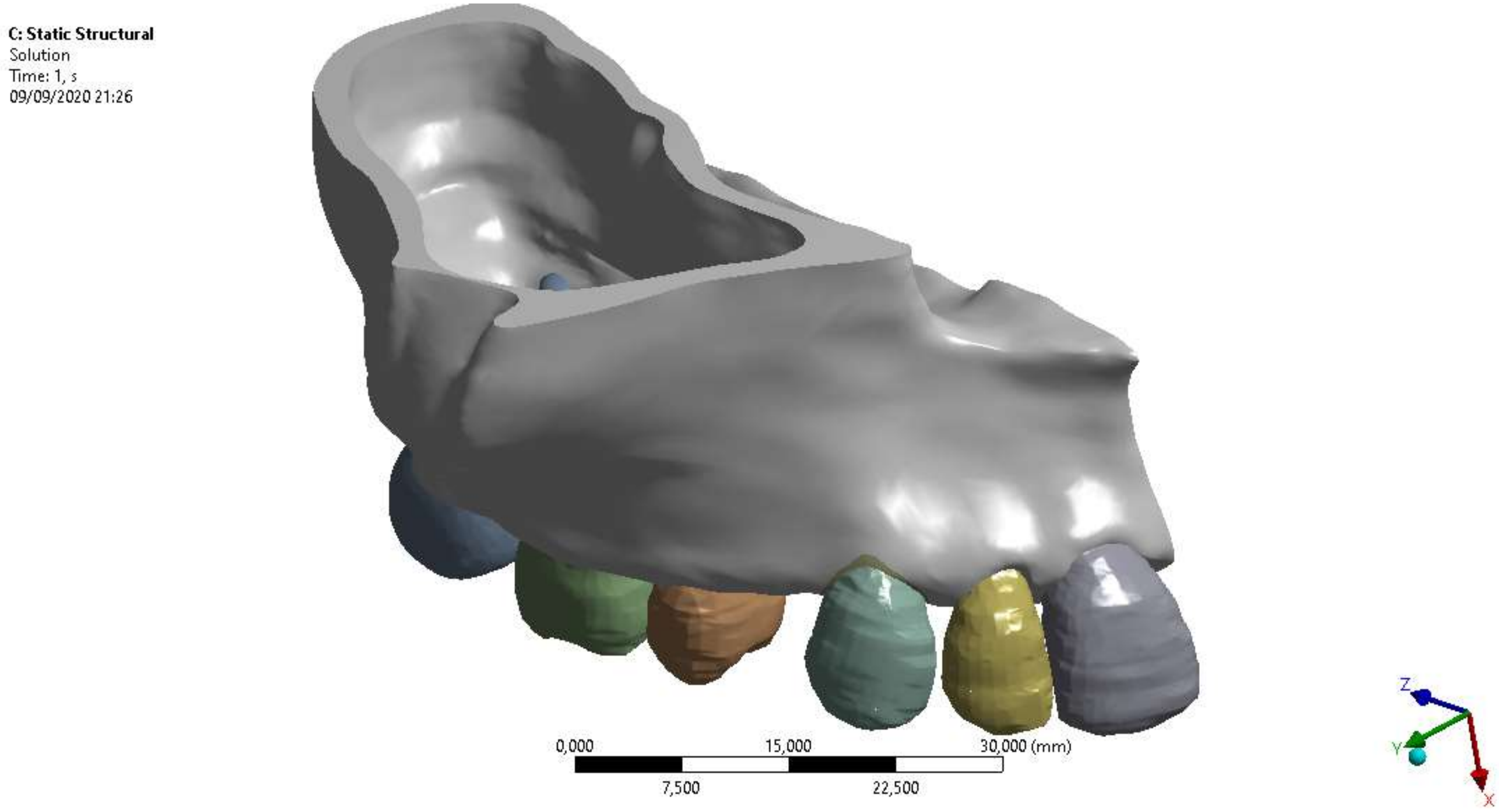
C: Static Structural
Solution
Time: 1, s
09/09/2020 21:26
0,000
15,000
30,000 (mm)
7,500
22,500
Z
Y
X

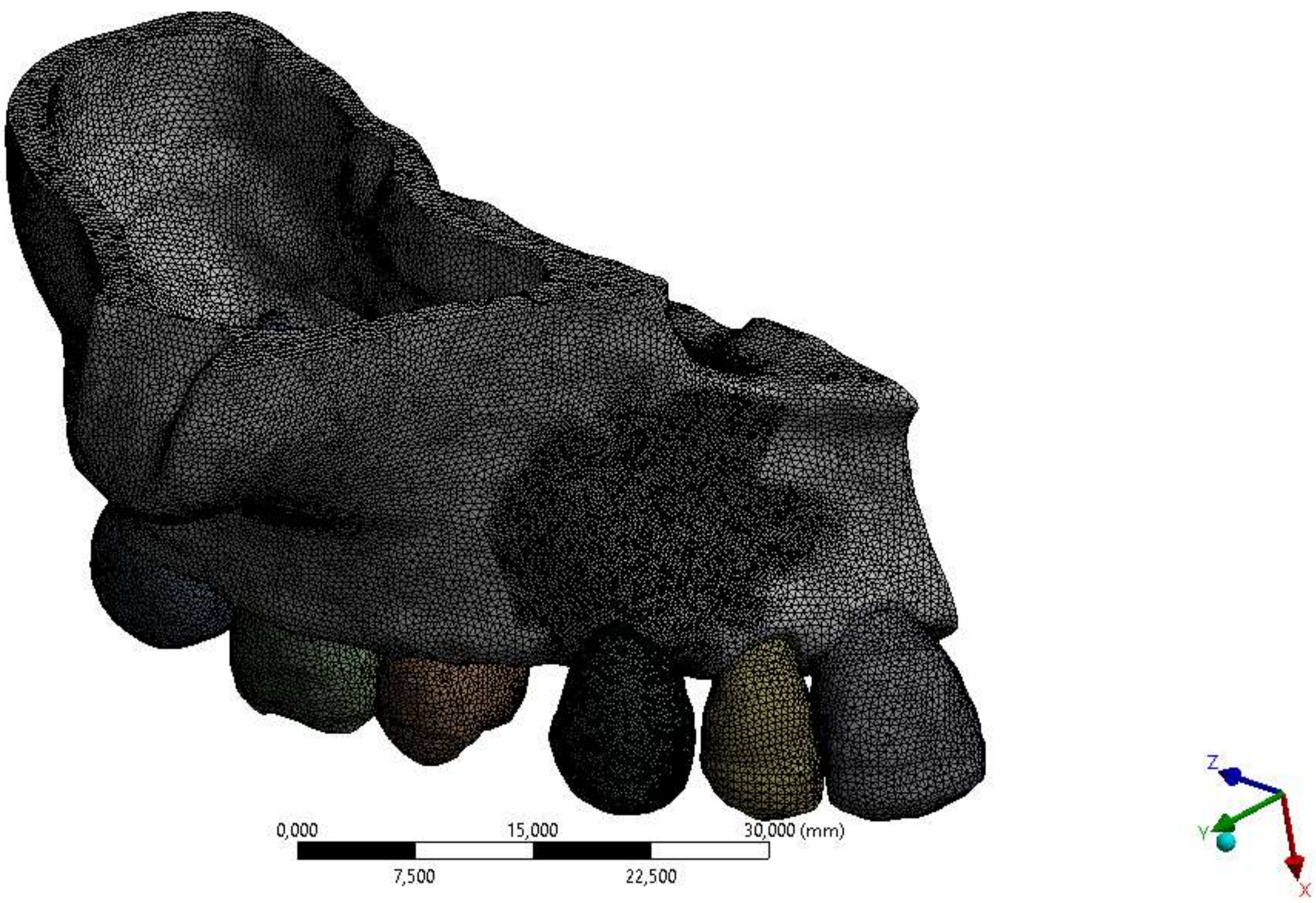

0,000
15,000
30,000 (mm)
7,500
22,500
Z
Y
X

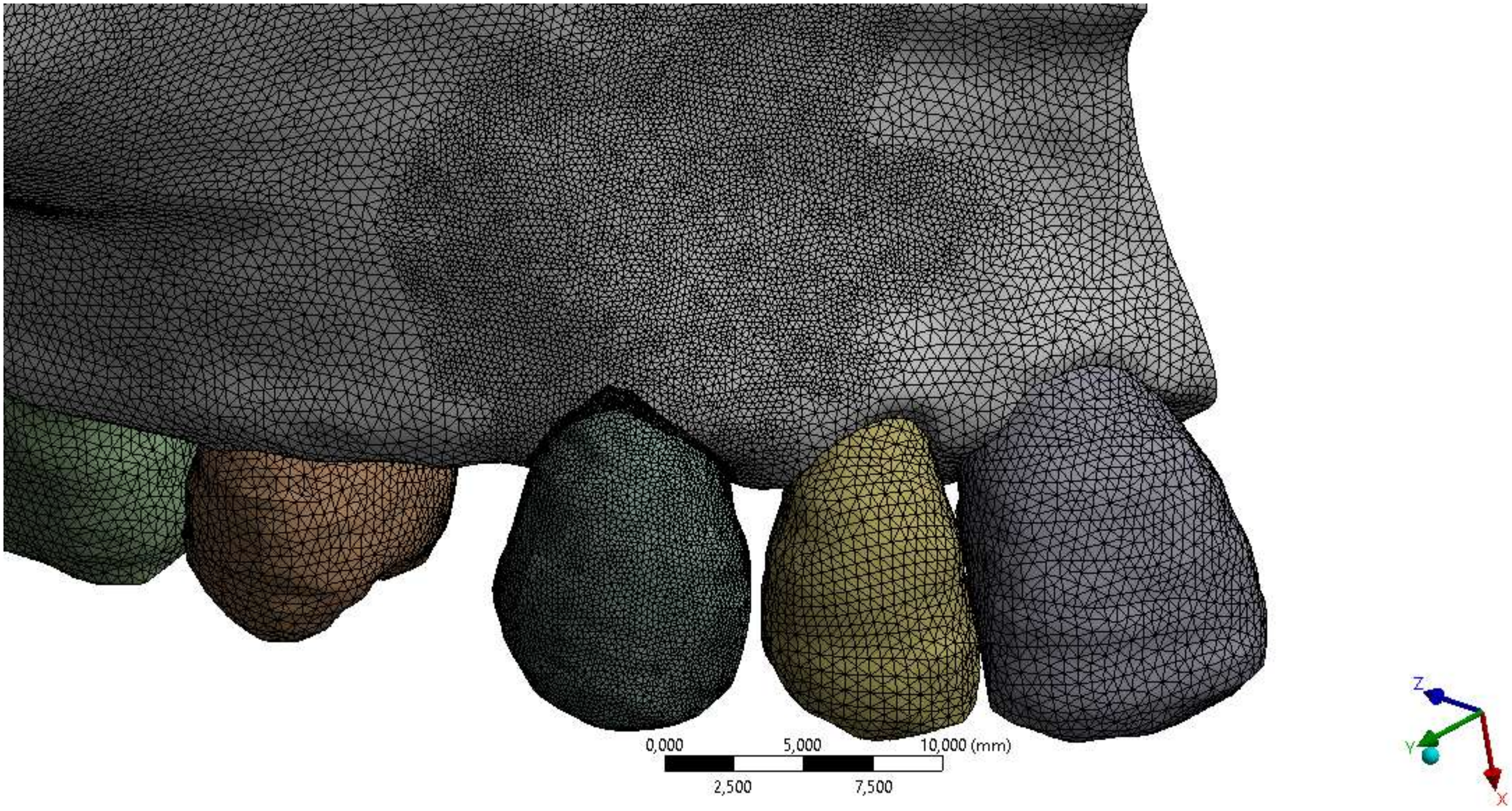

0,000
5,000
10,000 (mm)
2,500
7,500
Z
Y
X

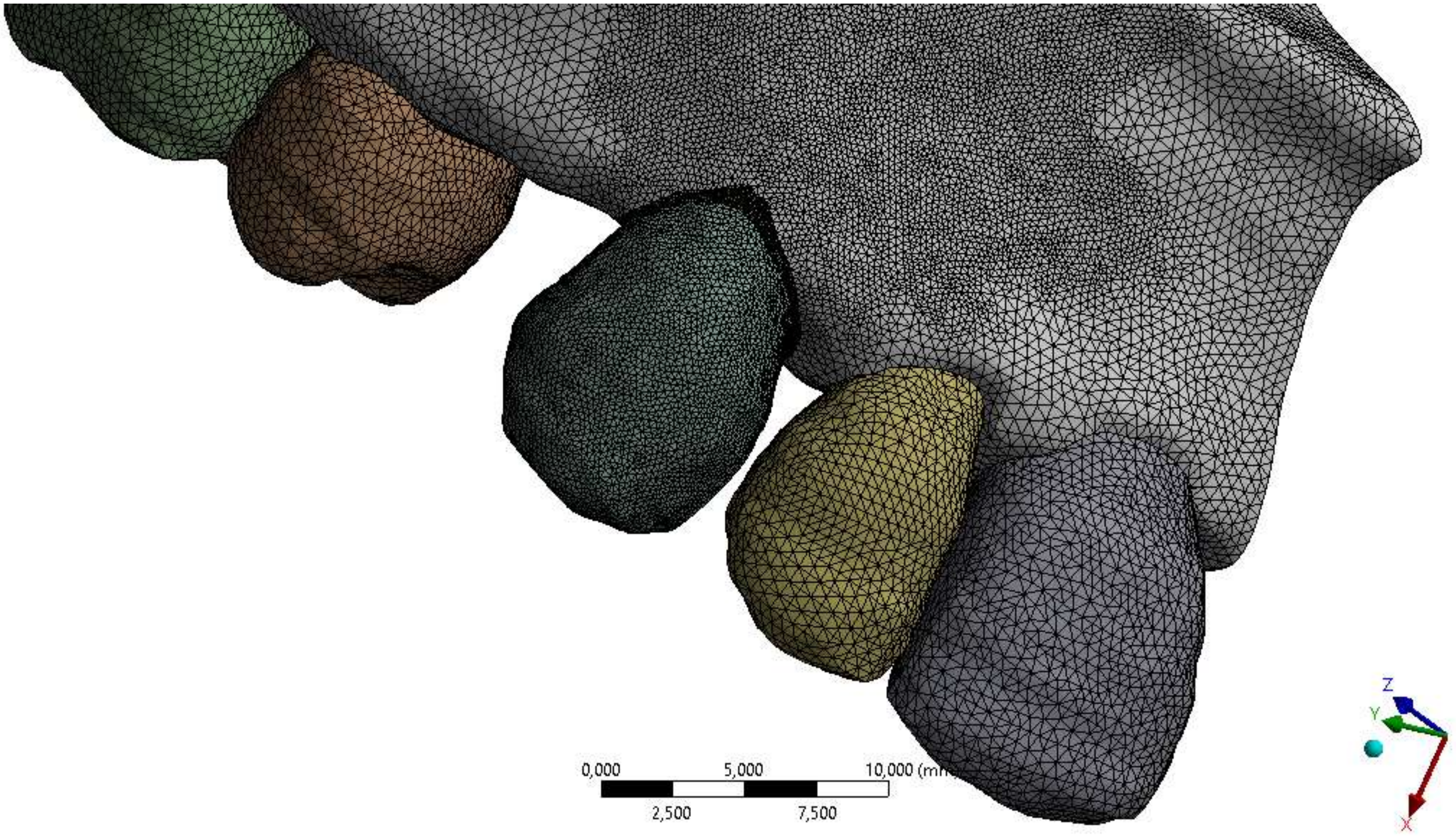
0,000
5,000
10,000 (mm)
2,500
7,500
Z
Y
X

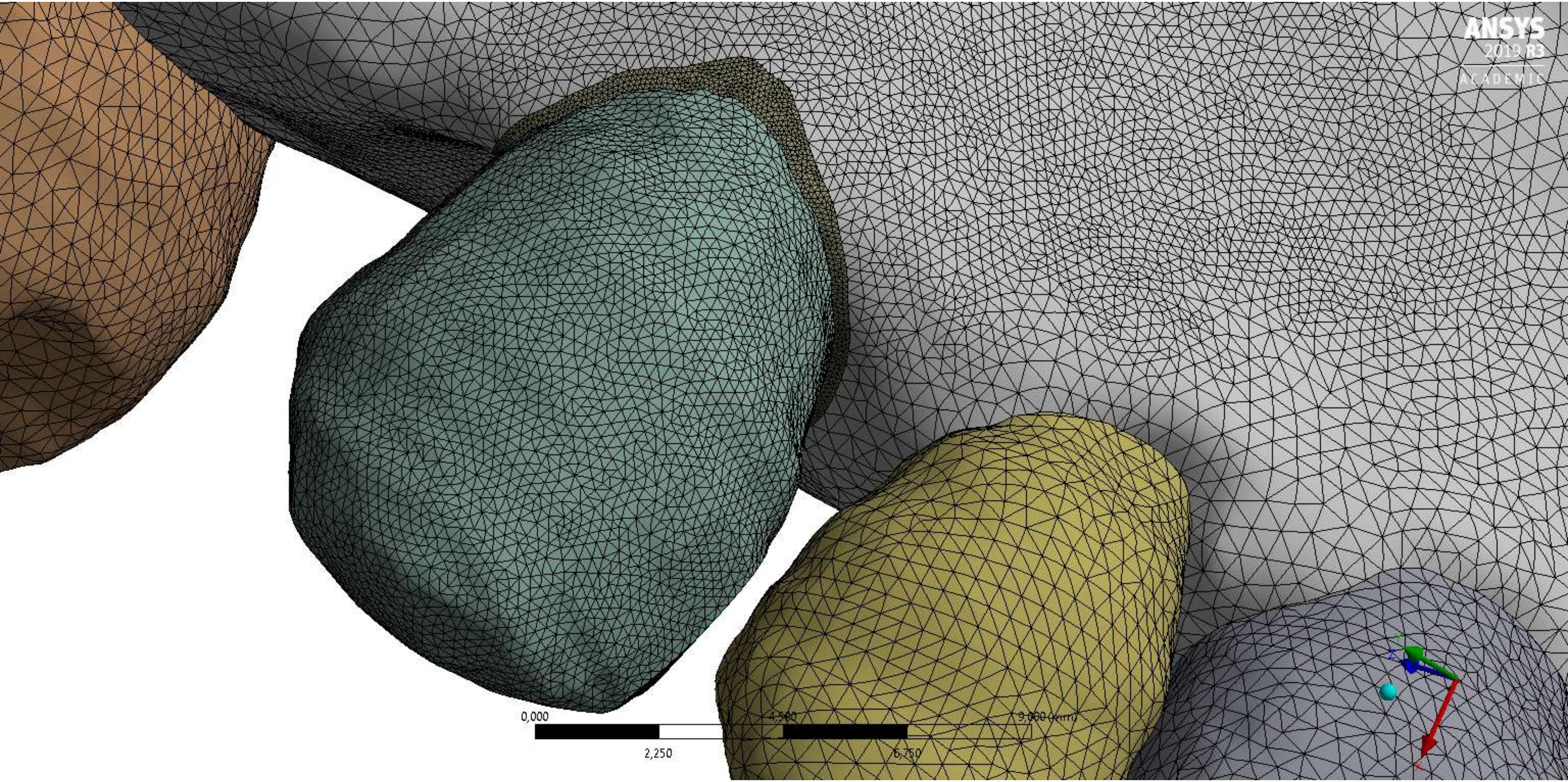
ANSYS
2019 R3
ACADEMIC
0,000
4,500
9,000 (mm)
2,250
6,750

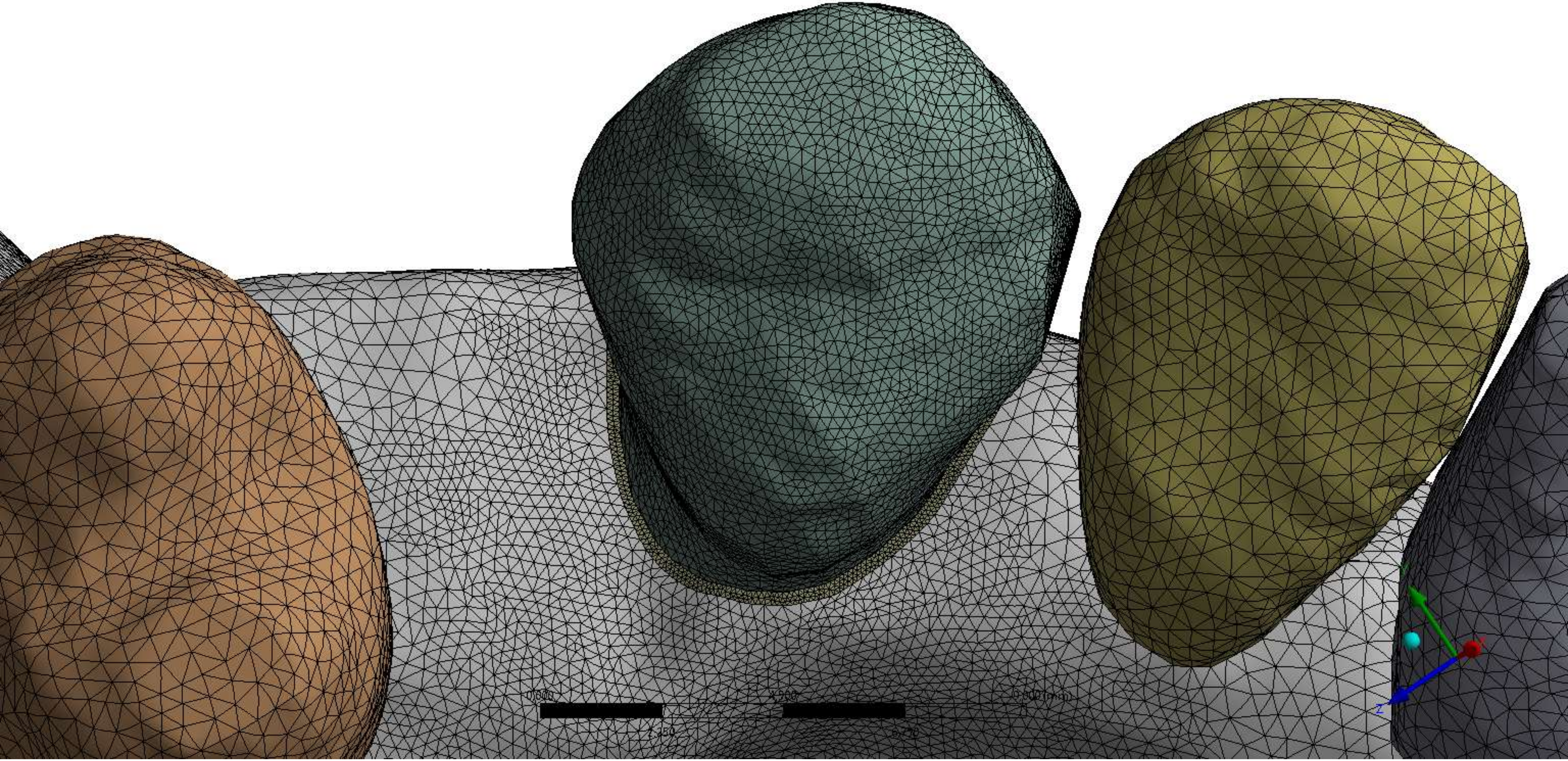

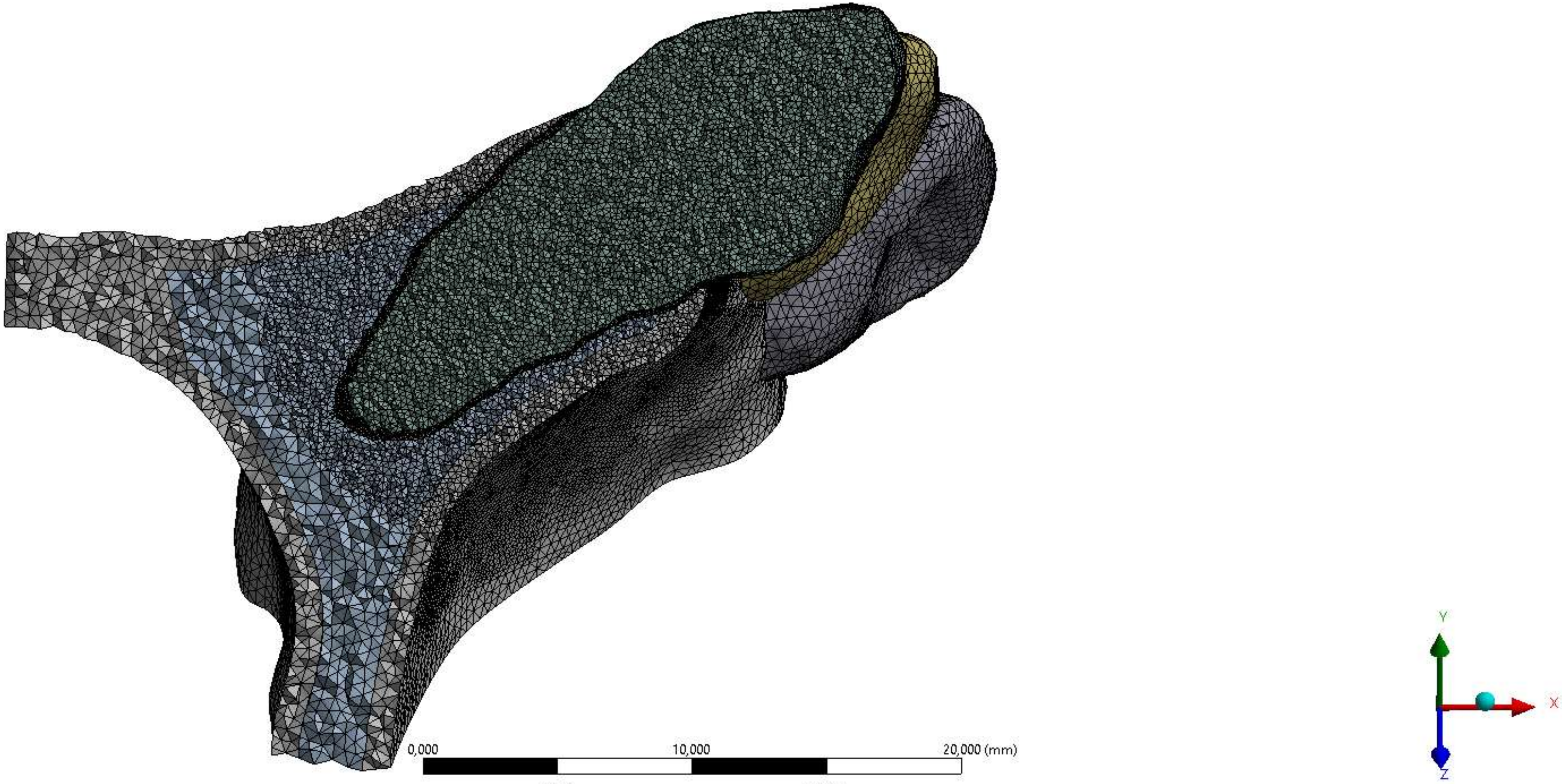

0,000
10,000
20,000 (mm)
5,000
15,000
Y
X
Z

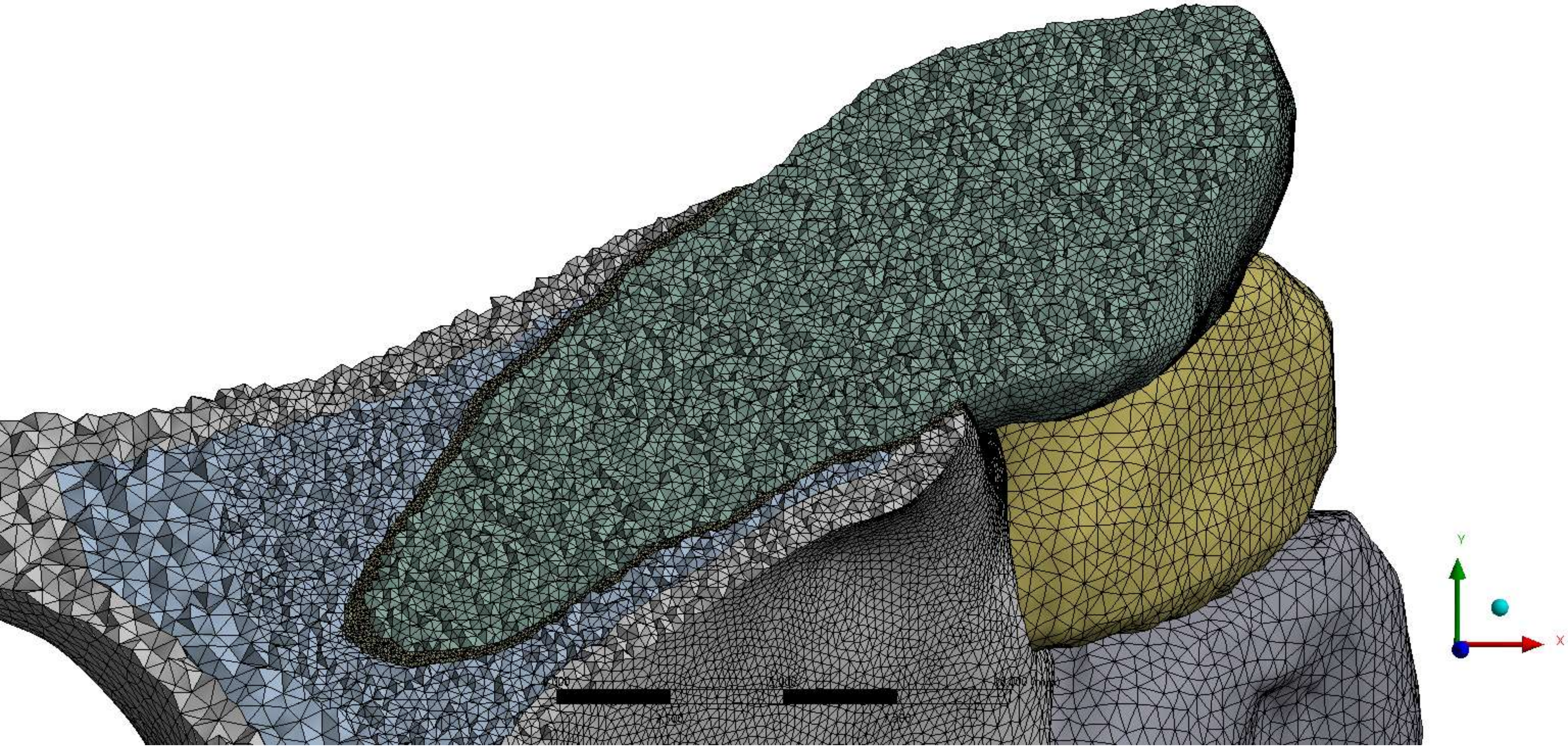
Y
X

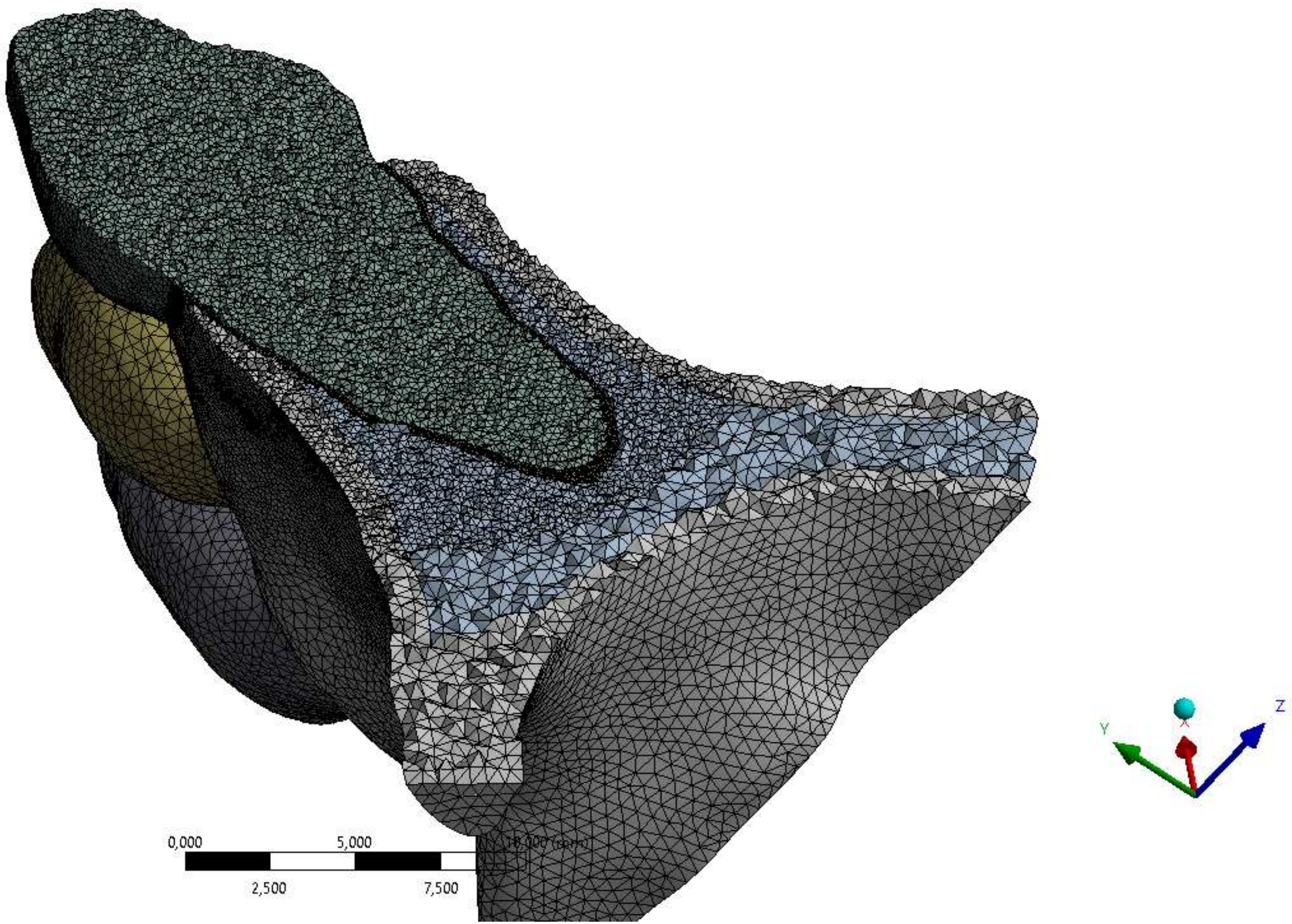
0,000
5,000
2,500
7,500
Y
Z

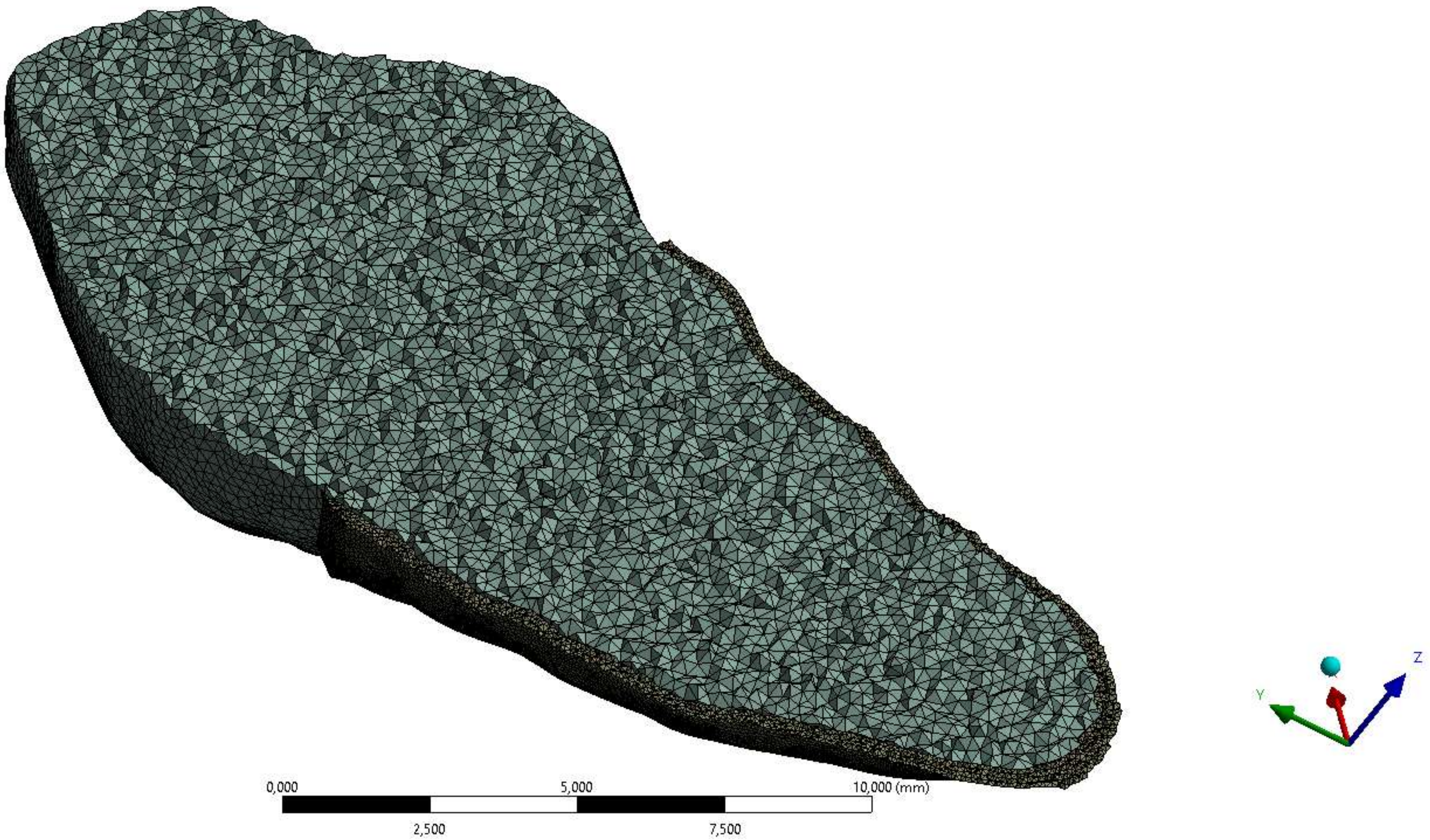
0,000
5,000
10,000 (mm)
2,500
7,500
Y
Z

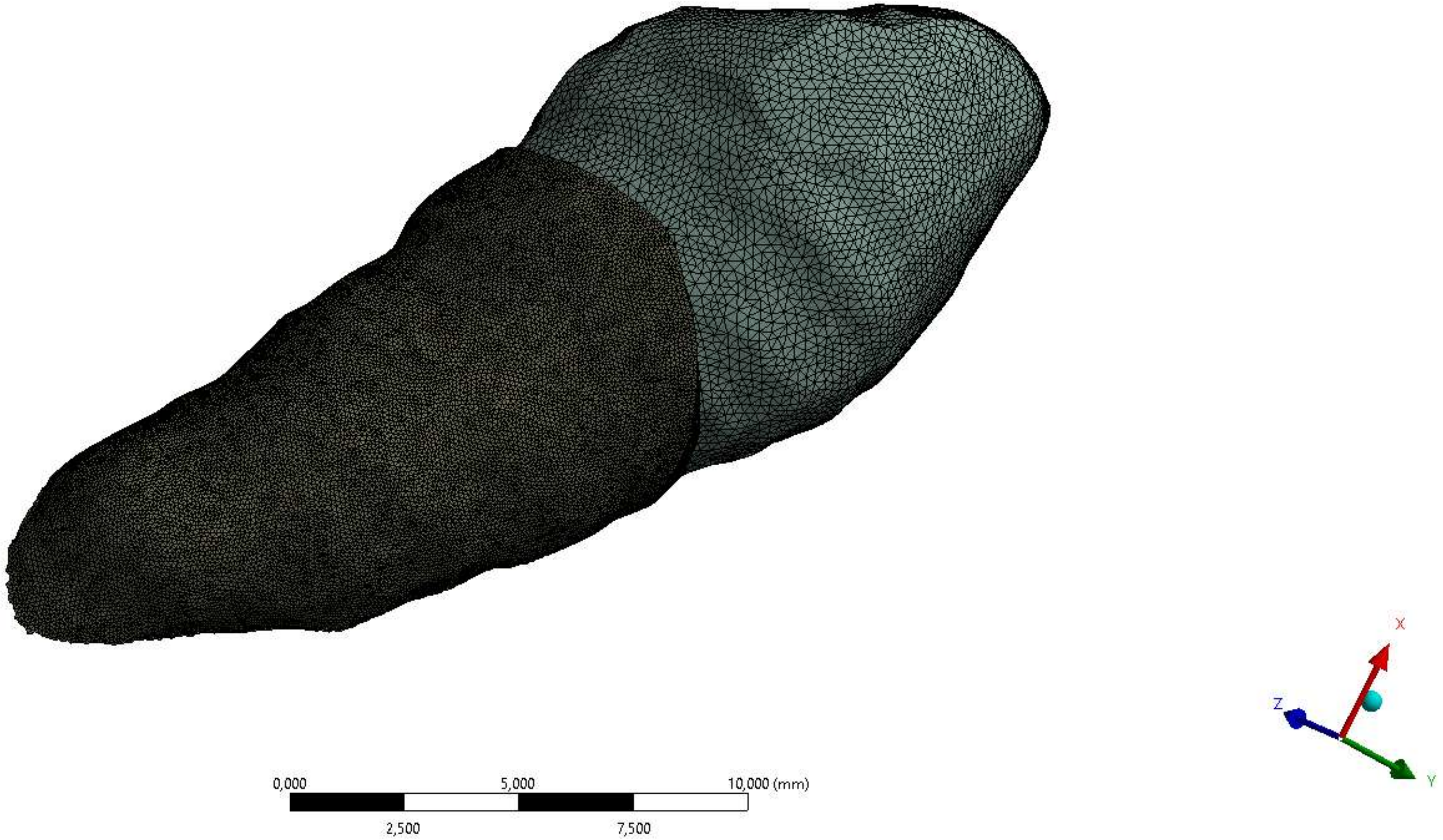
0,000
5,000
10,000 (mm)
2,500
7,500
X
Z
Y

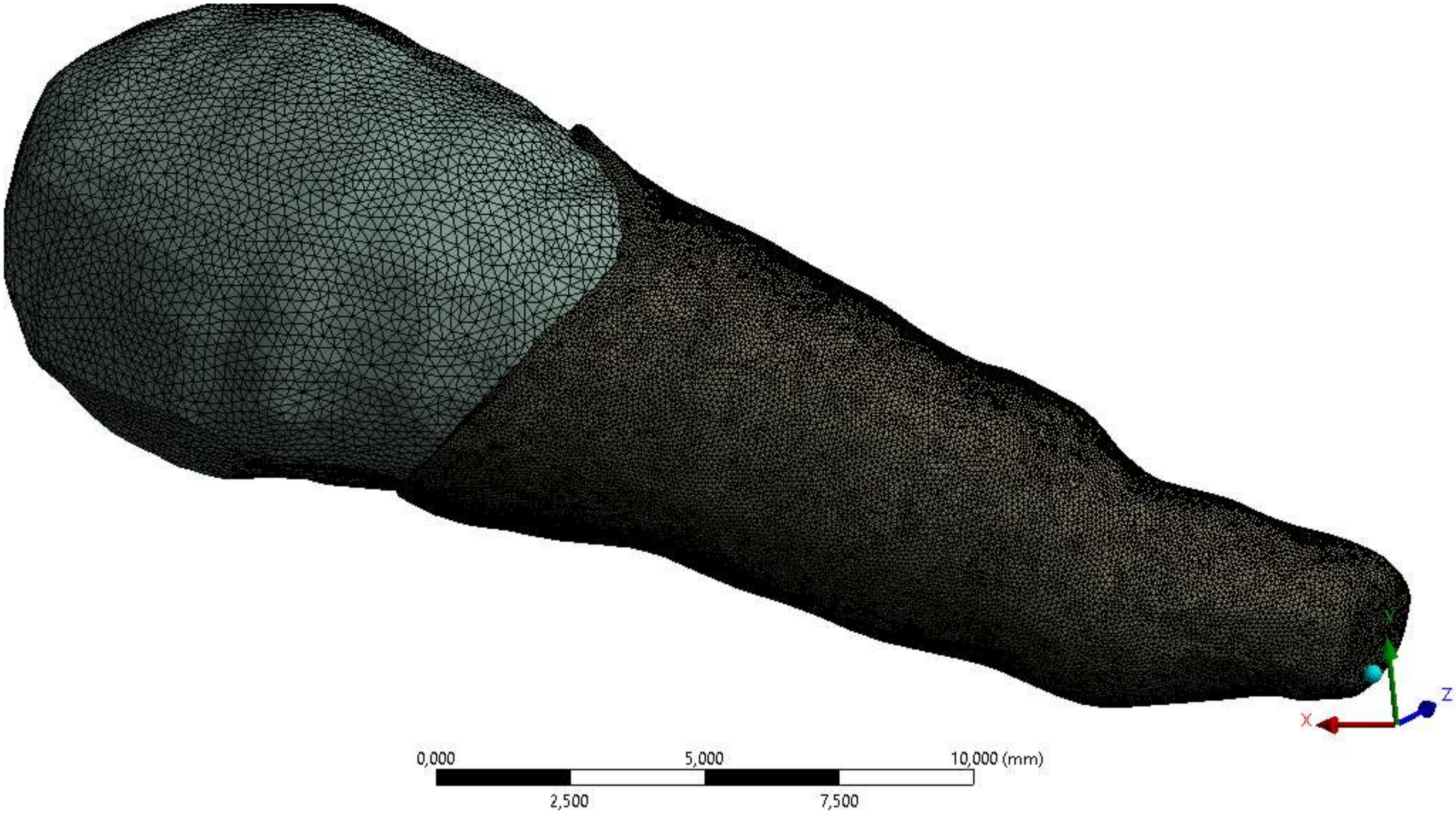

0,000
5,000
10,000 (mm)
2,500
7,500
Y
Z
X

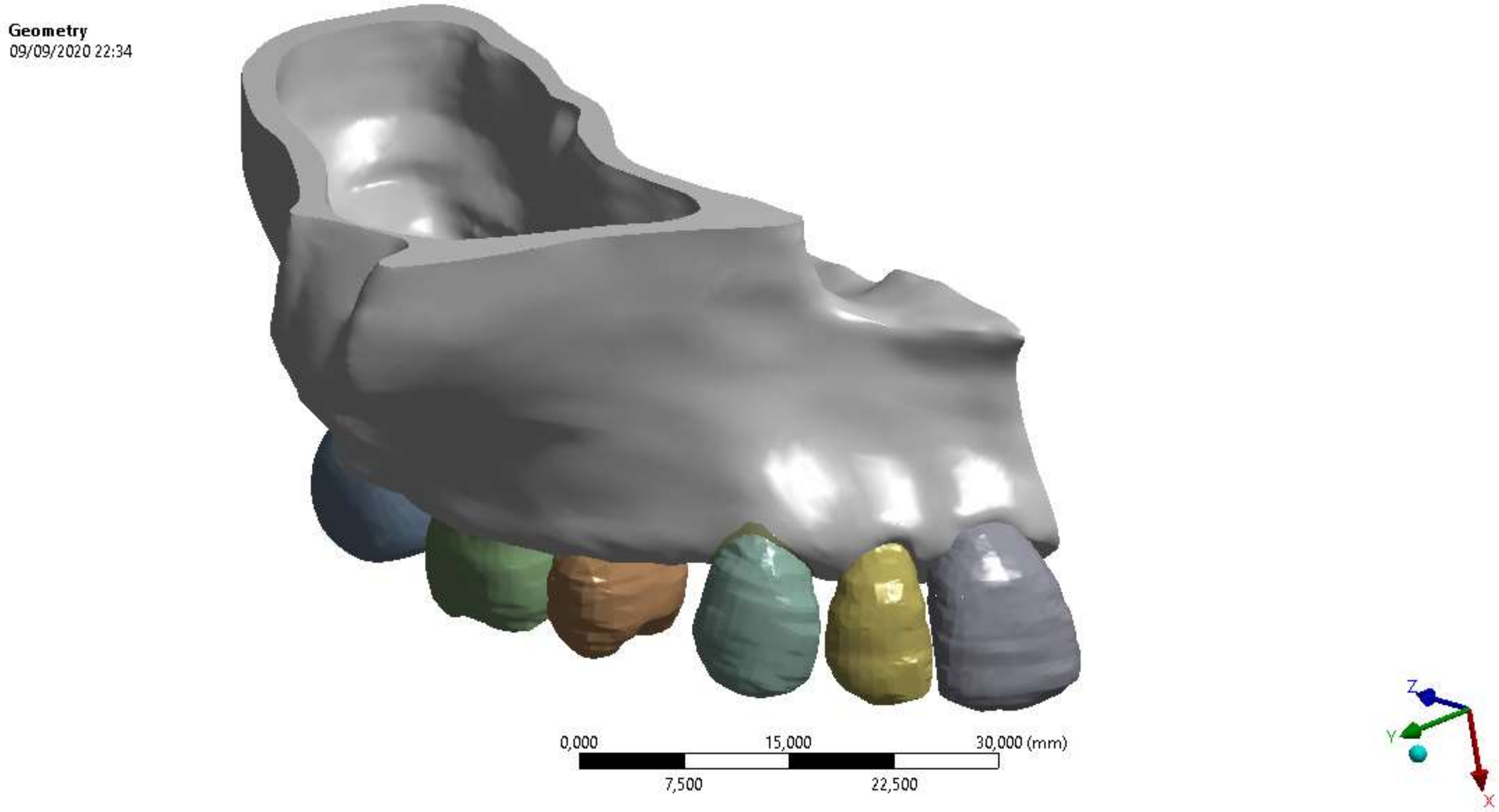
Geometry
09/09/2020 22:34
0,000
15,000
30,000 (mm)
7,500
22,500
Z
Y
X

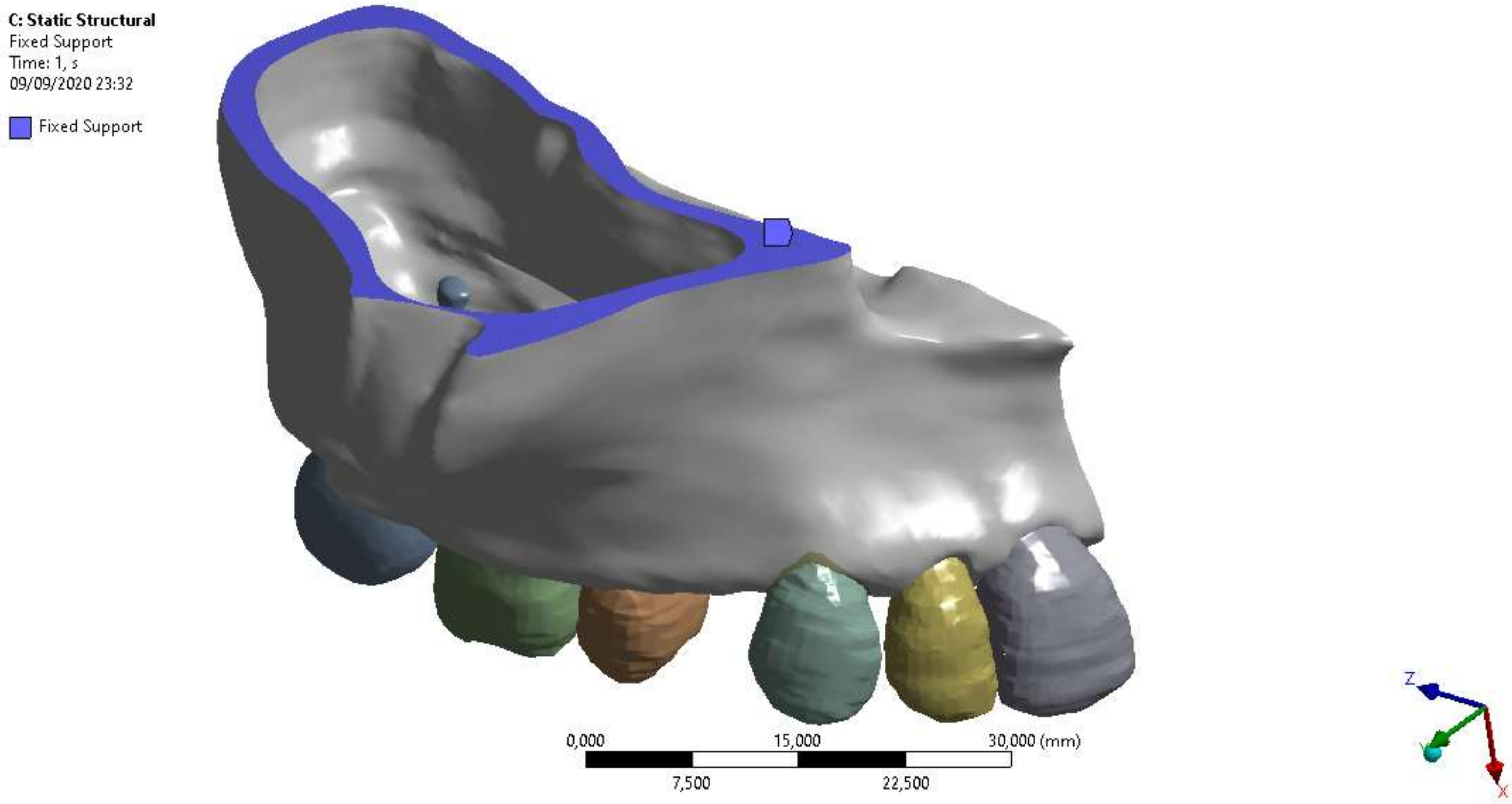
C: Static Structural
Fixed Support
Time: 1, s
09/09/2020 23:32
Fixed Support
0,000
15,000
30,000 (mm)
7,500
22,500
Z
Y
X

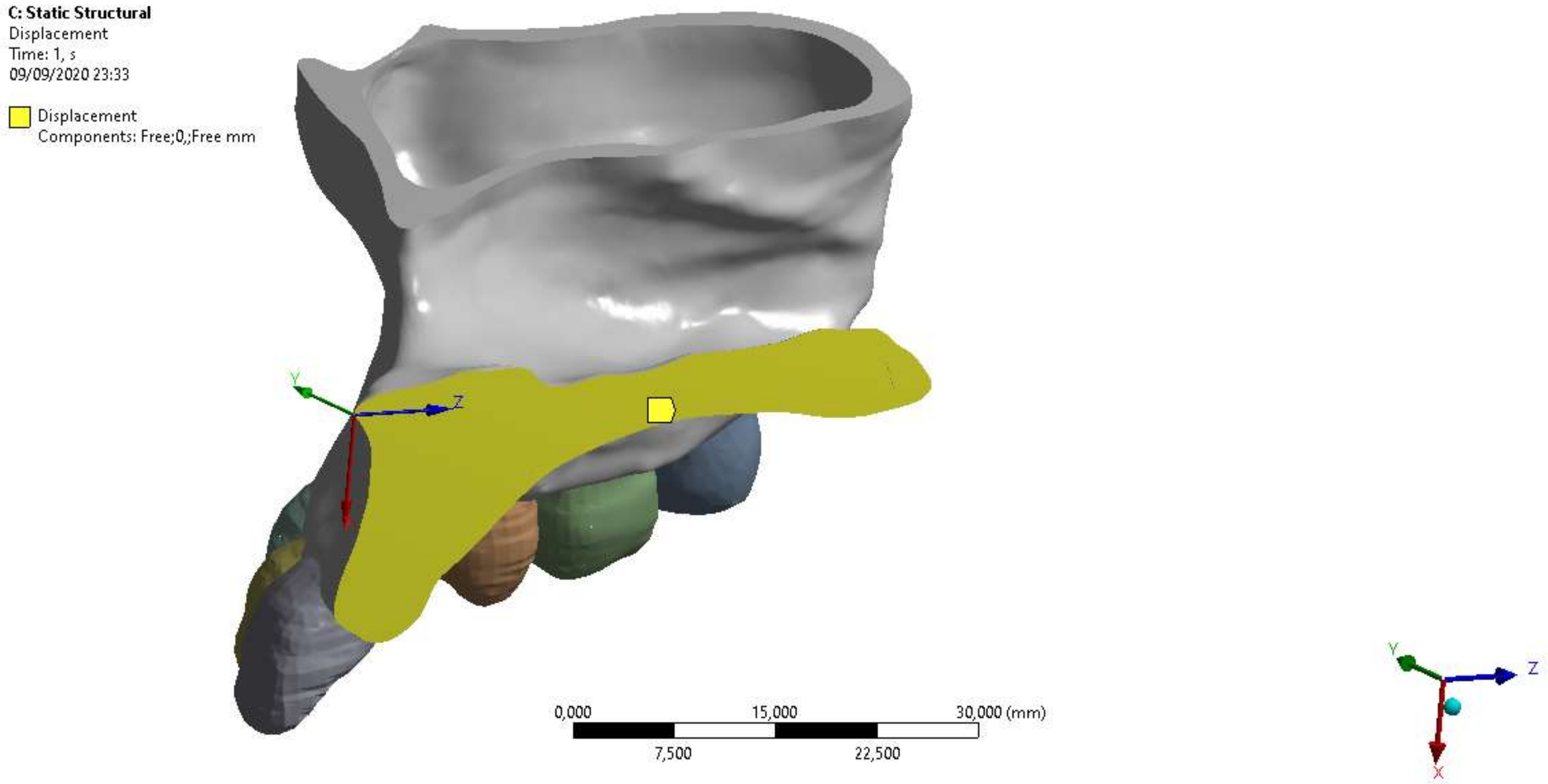
C: Static Structural
Displacement
Time: 1, s
09/09/2020 23:33
Displacement
Components: Free;0,;Free mm
Y
Z
X
0,000
15,000
30,000 (mm)
7,500
22,500

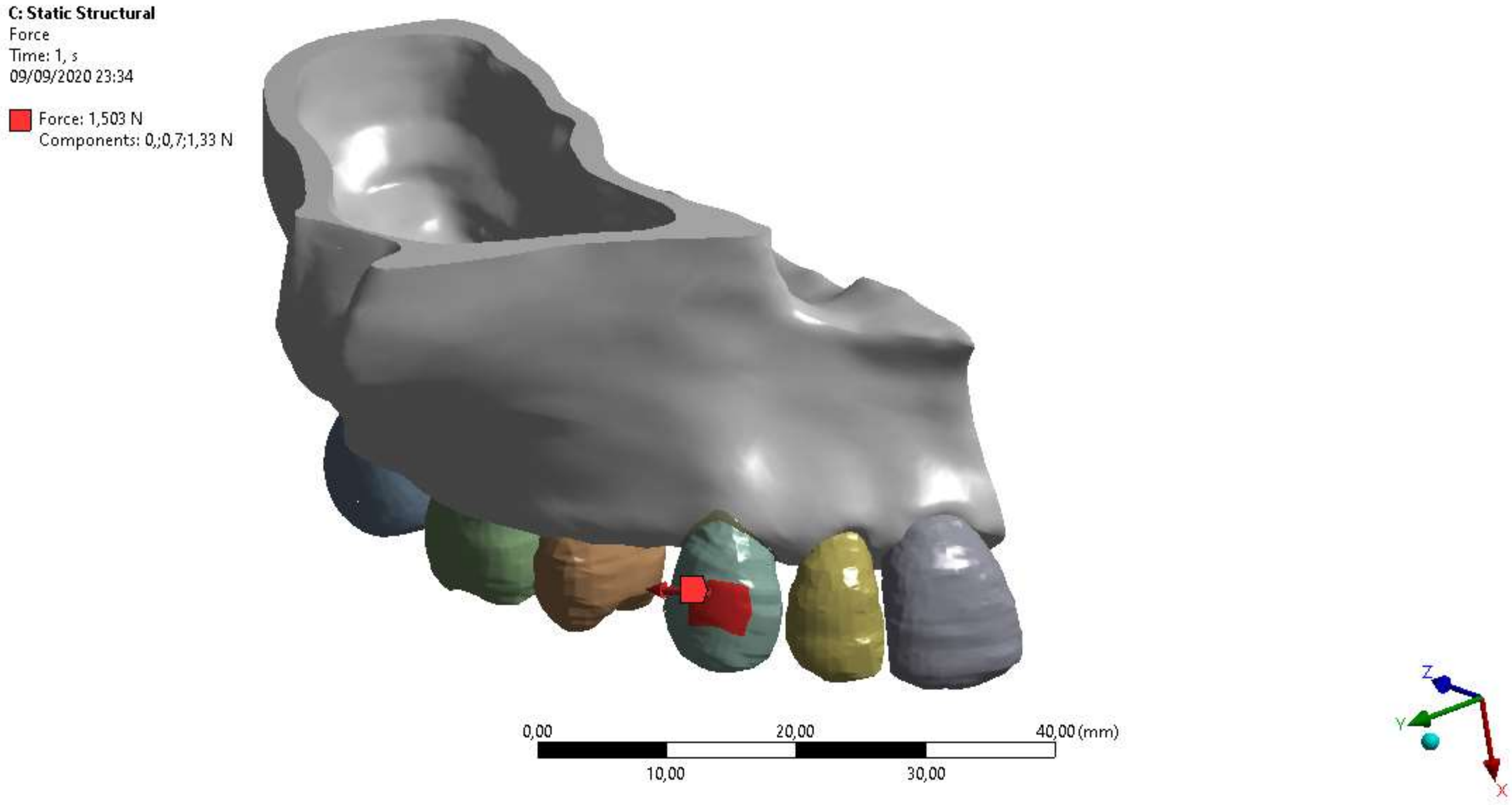
C: Static Structural
Force
Time: 1, s
09/09/2020 23:34
Force: 1,503 N
Components: 0,;0,7;1,33 N
0,00
20,00
40,00 (mm)
10,00
30,00
Z
Y
X

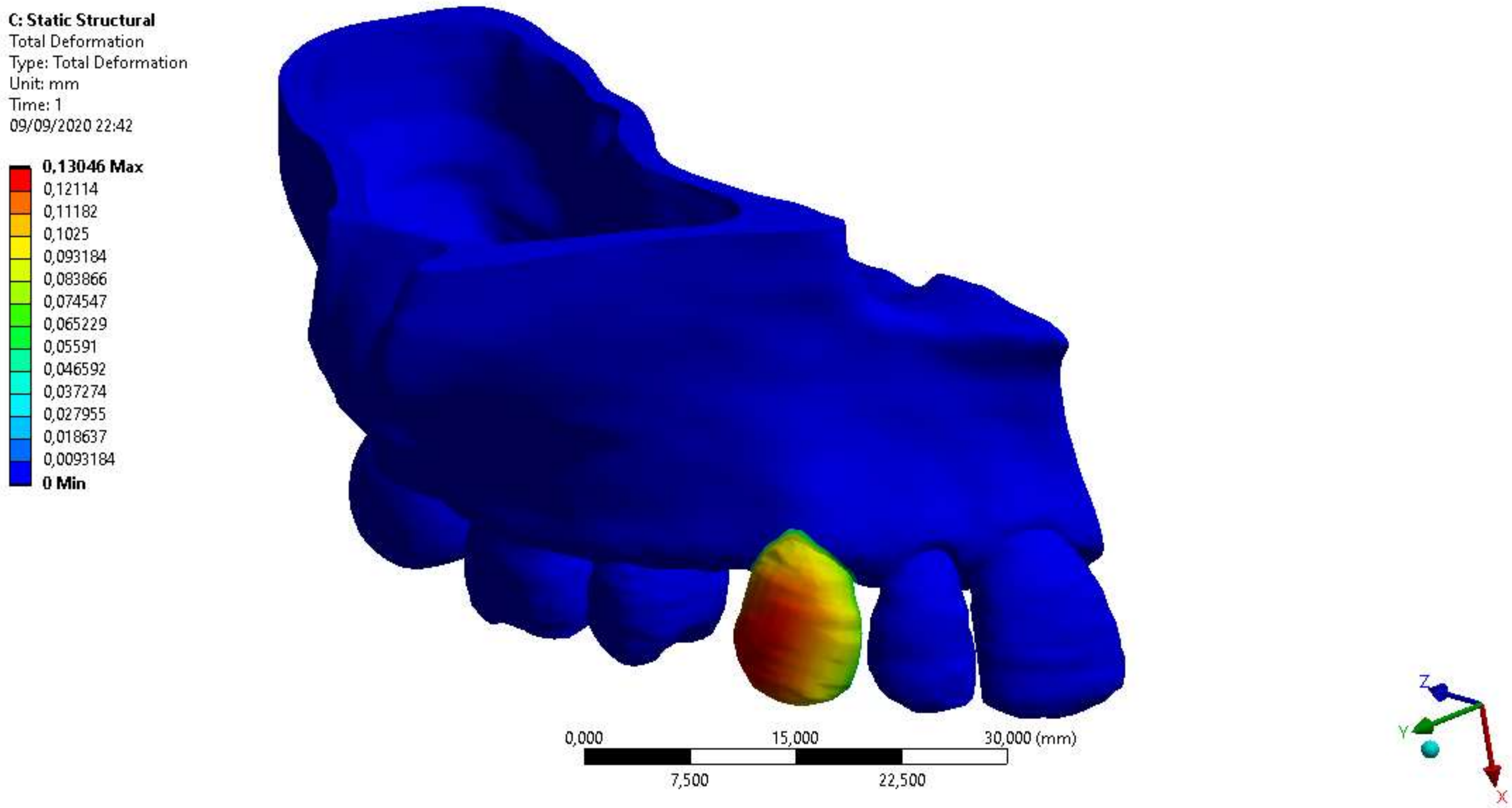
C: Static Structural
Total Deformation
Type: Total Deformation
Unit: mm
Time: 1
09/09/2020 22:42
0,13046 Max
0,12114
0,11182
0,1025
0,093184
0,083866
0,074547
0,065229
0,05591
0,046592
0,037274
0,027955
0,018637
0,0093184
0 Min
0,000
15,000
30,000 (mm)
7,500
22,500
Z
Y
X

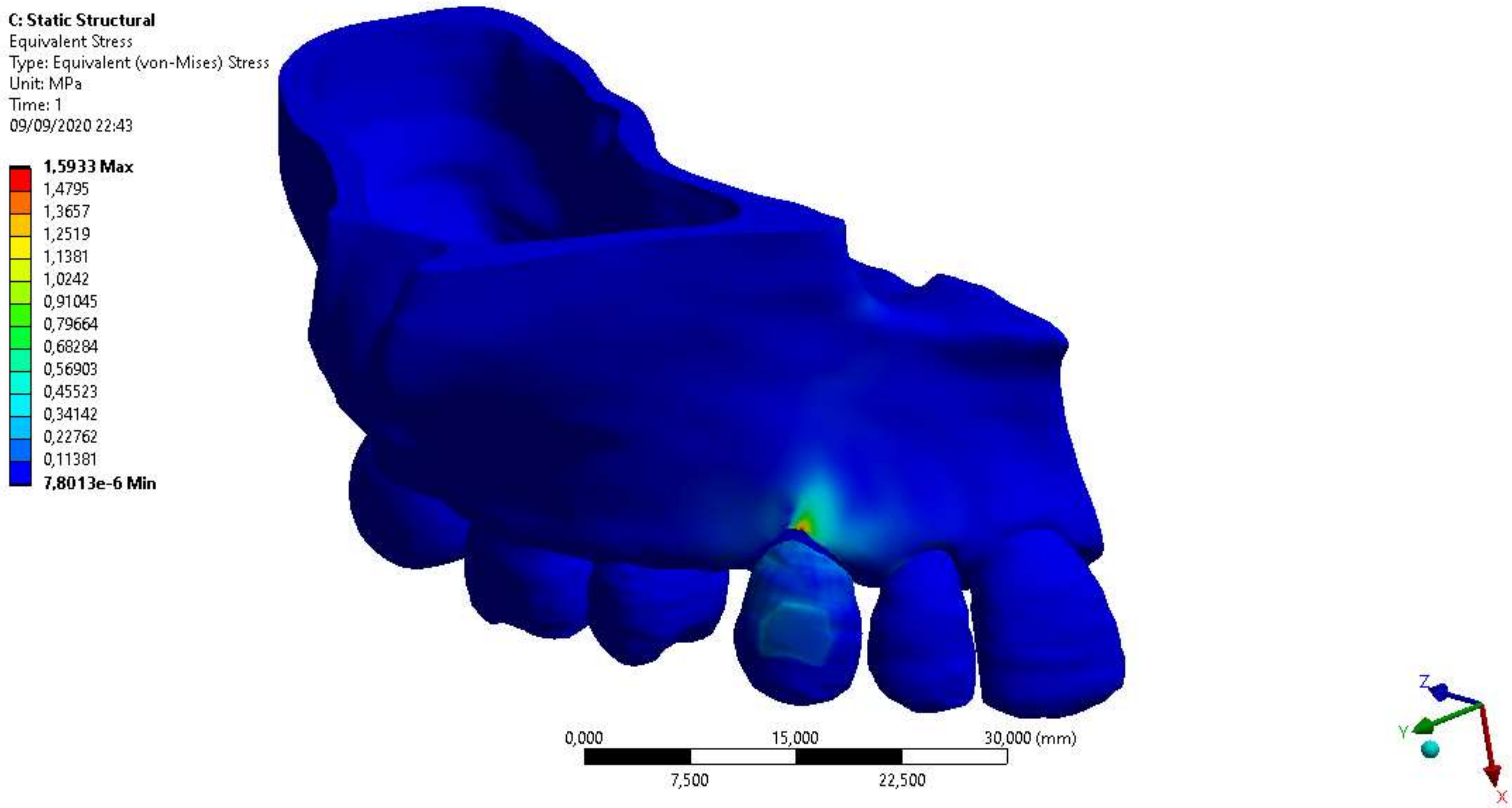
C: Static Structural
Equivalent Stress
Type: Equivalent (von-Mises) Stress
Unit: MPa
Time: 1
09/09/2020 22:43
1,5933 Max
1,4795
1,3657
1,2519
1,1381
1,0242
0,91045
0,79664
0,68284
0,56903
0,45523
0,34142
0,22762
0,11381
7,8013e-6 Min
0,000
15,000
30,000 (mm)
7,500
22,500
Z
Y
X

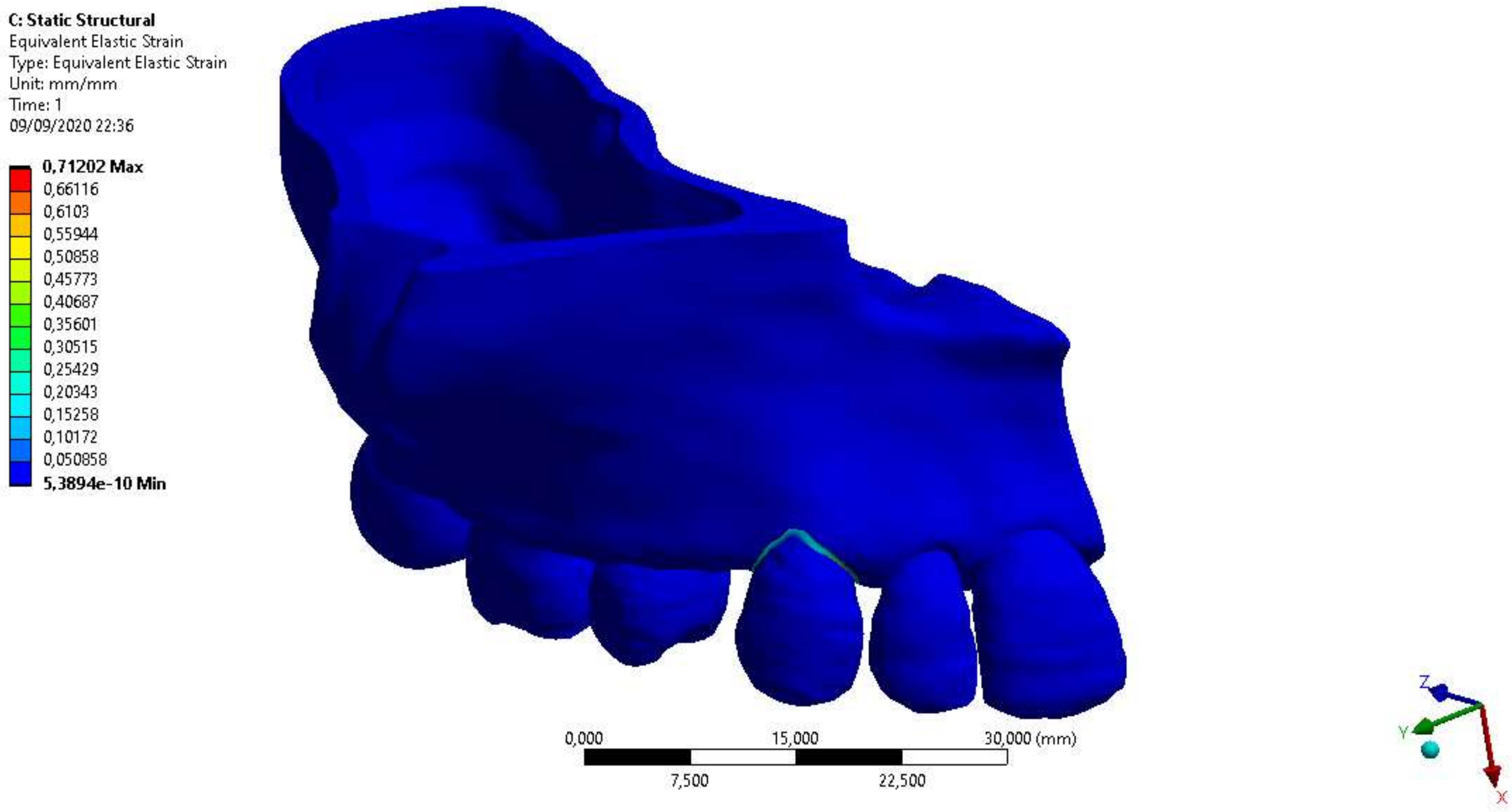
C: Static Structural
Equivalent Elastic Strain
Type: Equivalent Elastic Strain
Unit: mm/mm
Time: 1
09/09/2020 22:36
0,71202 Max
0,66116
0,6103
0,55944
0,50858
0,45773
0,40687
0,35601
0,30515
0,25429
0,20343
0,15258
0,10172
0,050858
5,3894e-10 Min
0,000
15,000
30,000 (mm)
7,500
22,500
Z
Y
X

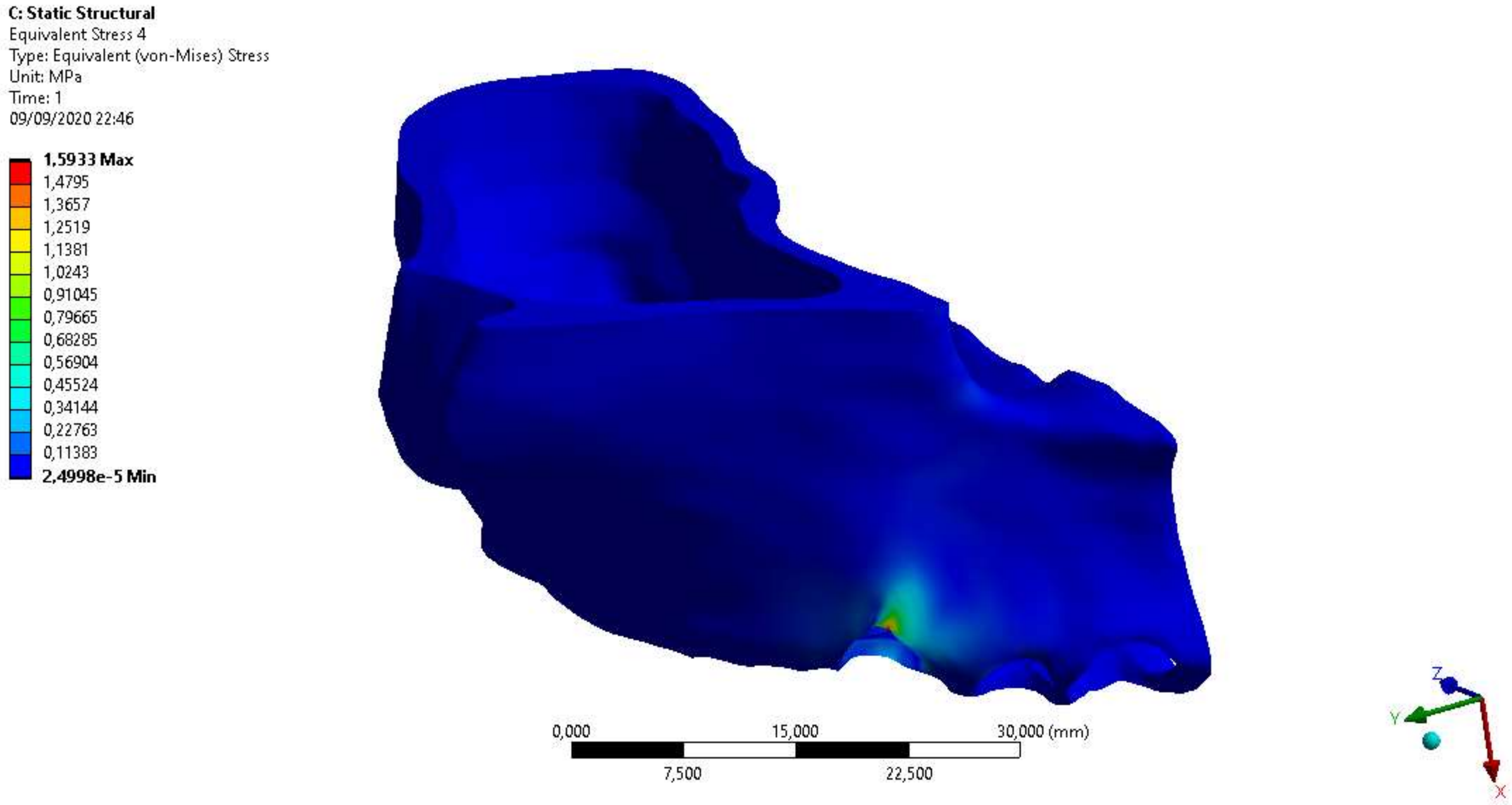
C: Static Structural
Equivalent Stress 4
Type: Equivalent (von-Mises) Stress
Unit: MPa
Time: 1
09/09/2020 22:46
1,5933 Max
1,4795
1,3657
1,2519
1,1381
1,0243
0,91045
0,79665
0,68285
0,56904
0,45524
0,34144
0,22763
0,11383
2,4998e-5 Min
0,000
15,000
30,000 (mm)
7,500
22,500
Z
Y
X

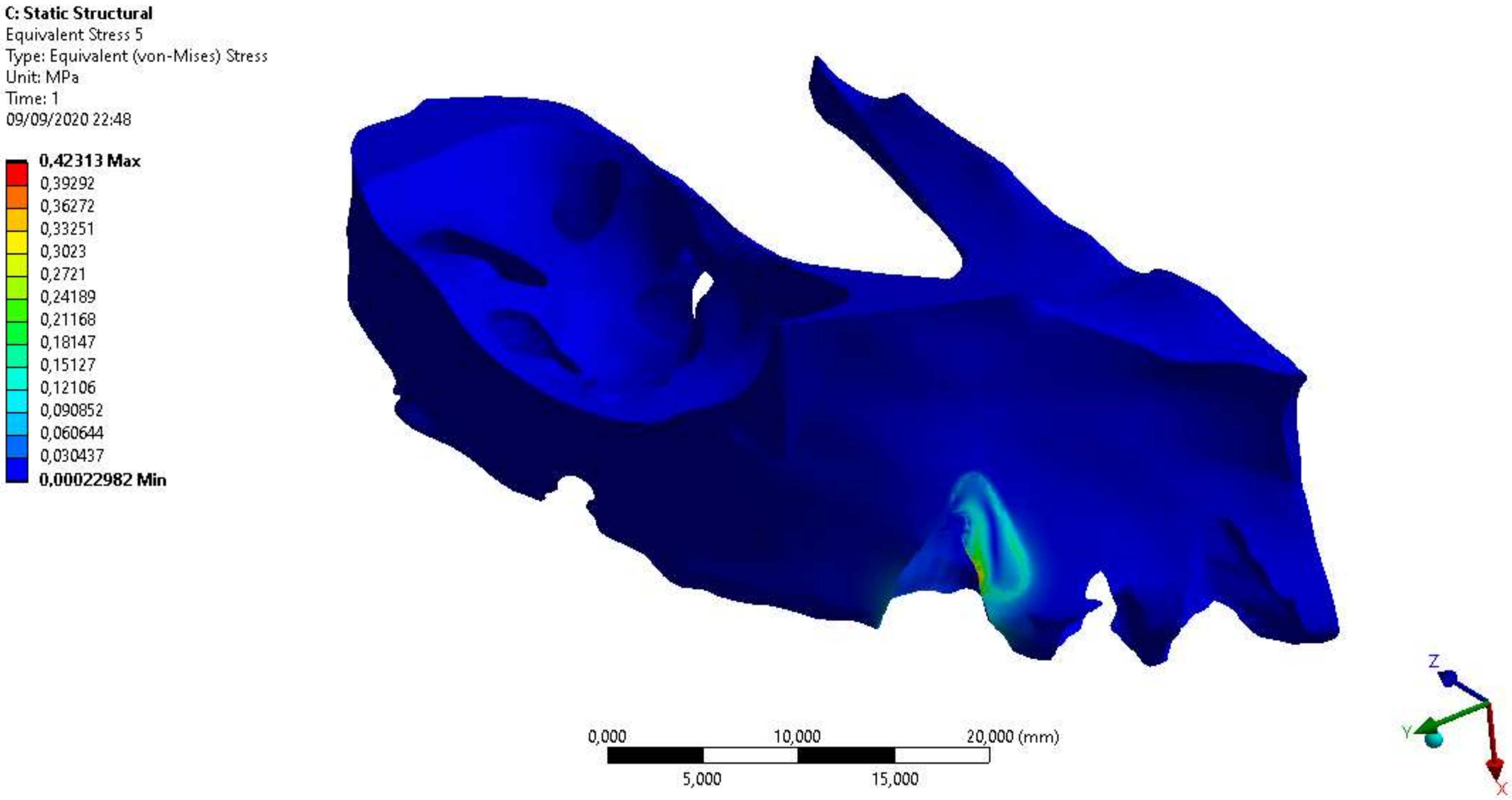
C: Static Structural
Equivalent Stress 5
Type: Equivalent (von-Mises) Stress
Unit: MPa
Time: 1
09/09/2020 22:48
0,42313 Max
0,39292
0,36272
0,33251
0,3023
0,2721
0,24189
0,21168
0,18147
0,15127
0,12106
0,090852
0,060644
0,030437
0,00022982 Min
0,000
10,000
20,000 (mm)
5,000
15,000
Z
Y
X

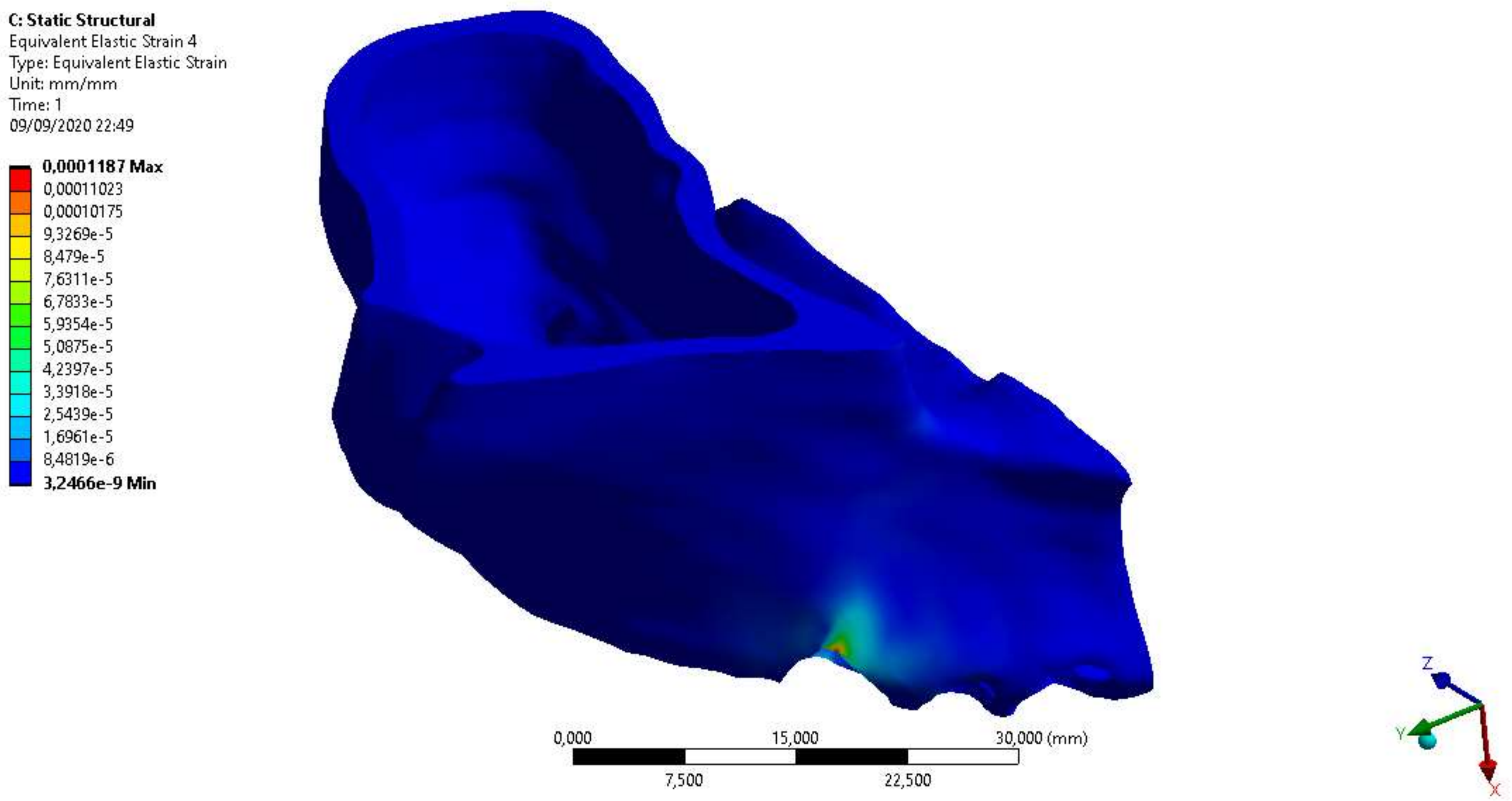

C: Static Structural
Equivalent Elastic Strain 4
Type: Equivalent Elastic Strain
Unit: mm/mm
Time: 1
09/09/2020 22:49
0,0001187 Max
0,00011023
0,00010175
9,3269e-5
8,479e-5
7,6311e-5
6,7833e-5
5,9354e-5
5,0875e-5
4,2397e-5
3,3918e-5
2,5439e-5
1,6961e-5
8,4819e-6
3,2466e-9 Min
0,000
15,000
30,000 (mm)
7,500
22,500
Z
Y
X

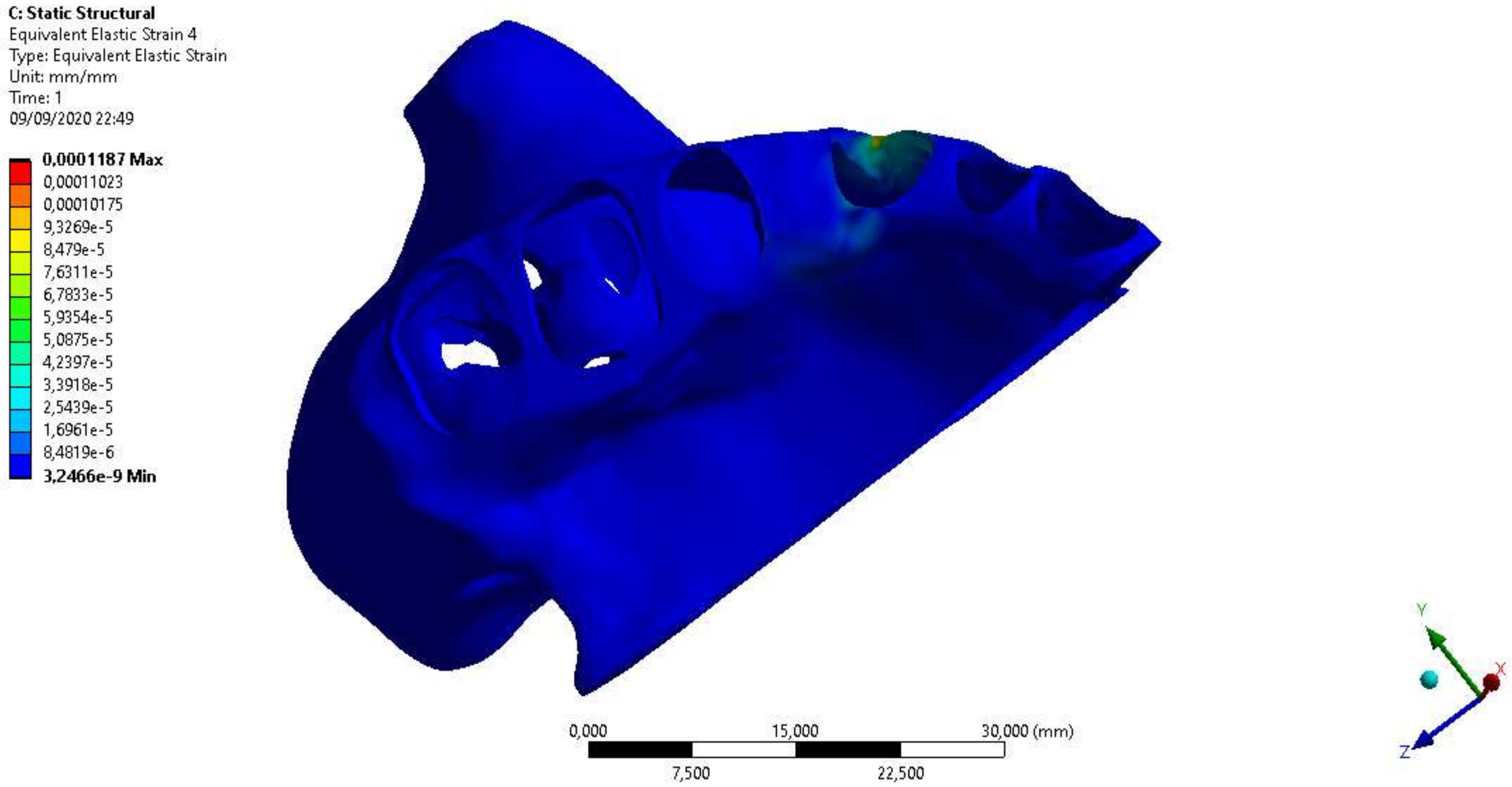
C: Static Structural
Equivalent Elastic Strain 4
Type: Equivalent Elastic Strain
Unit: mm/mm
Time: 1
09/09/2020 22:49
0,0001187 Max
0,00011023
0,00010175
9,3269e-5
8,479e-5
7,6311e-5
6,7833e-5
5,9354e-5
5,0875e-5
4,2397e-5
3,3918e-5
2,5439e-5
1,6961e-5
8,4819e-6
3,2466e-9 Min
0,000
15,000
30,000 (mm)
7,500
22,500
Y
X
Z

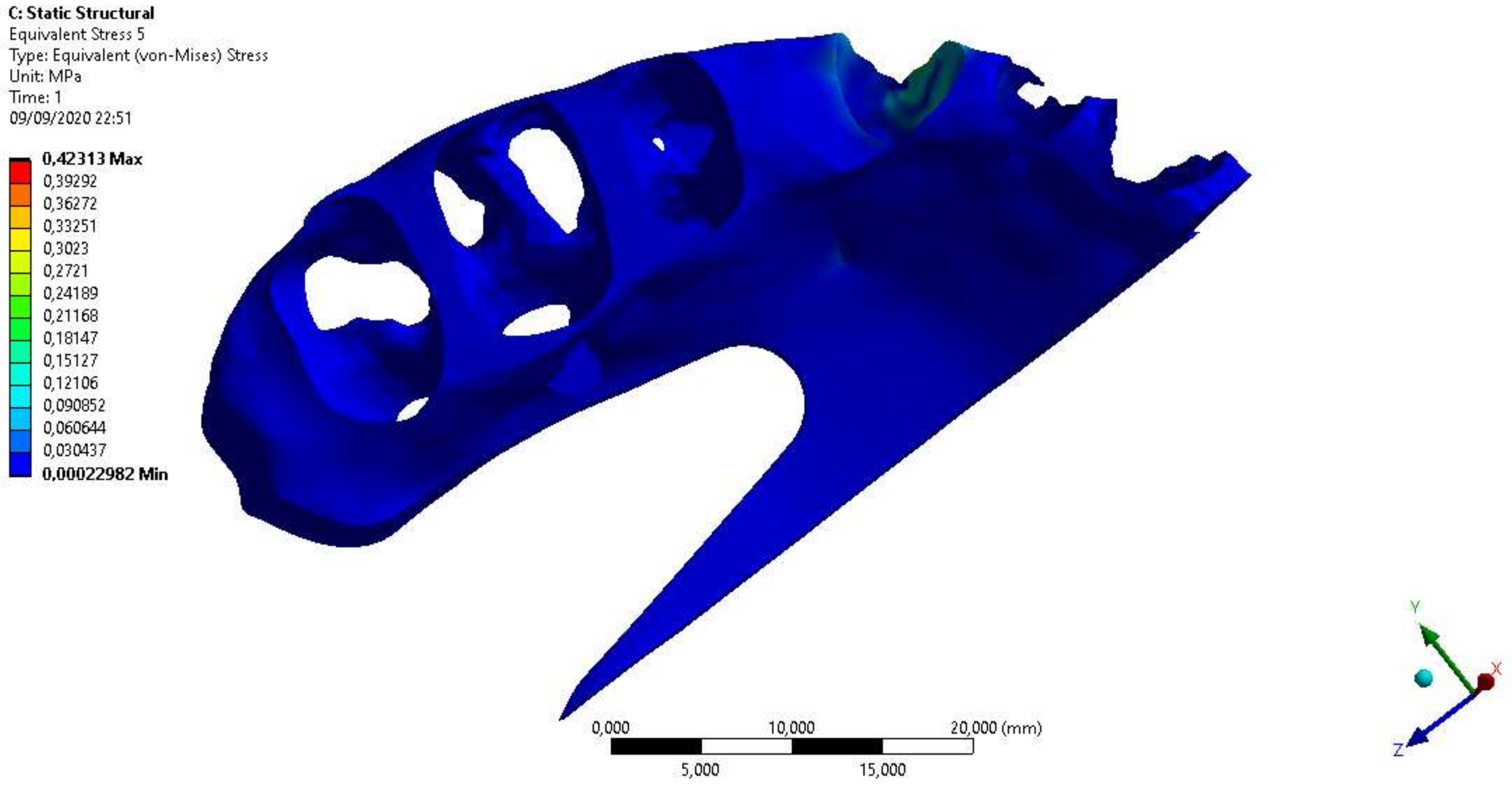
C: Static Structural
Equivalent Stress 5
Type: Equivalent (von-Mises) Stress
Unit: MPa
Time: 1
09/09/2020 22:51
0,42313 Max
0,39292
0,36272
0,33251
0,3023
0,2721
0,24189
0,21168
0,18147
0,15127
0,12106
0,090852
0,060644
0,030437
0,00022982 Min
0,000
10,000
20,000 (mm)
5,000
15,000
Y
X
Z

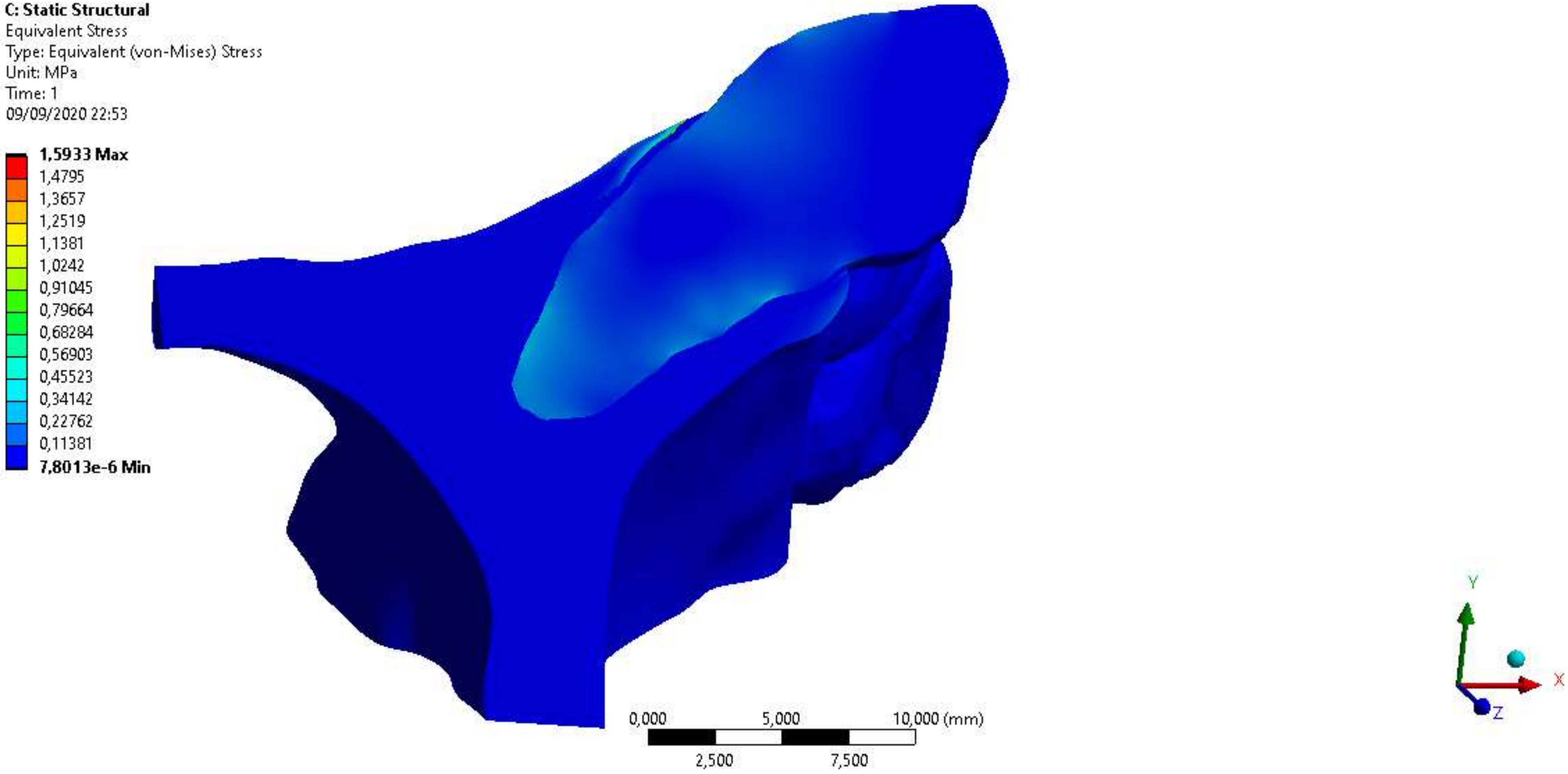
C: Static Structural
Equivalent Stress
Type: Equivalent (von-Mises) Stress
Unit: MPa
Time: 1
09/09/2020 22:53
1,5933 Max
1,4795
1,3657
1,2519
1,1381
1,0242
0,91045
0,79664
0,68284
0,56903
0,45523
0,34142
0,22762
0,11381
7,8013e-6 Min
0,000
5,000
10,000 (mm)
2,500
7,500
Y
X
Z

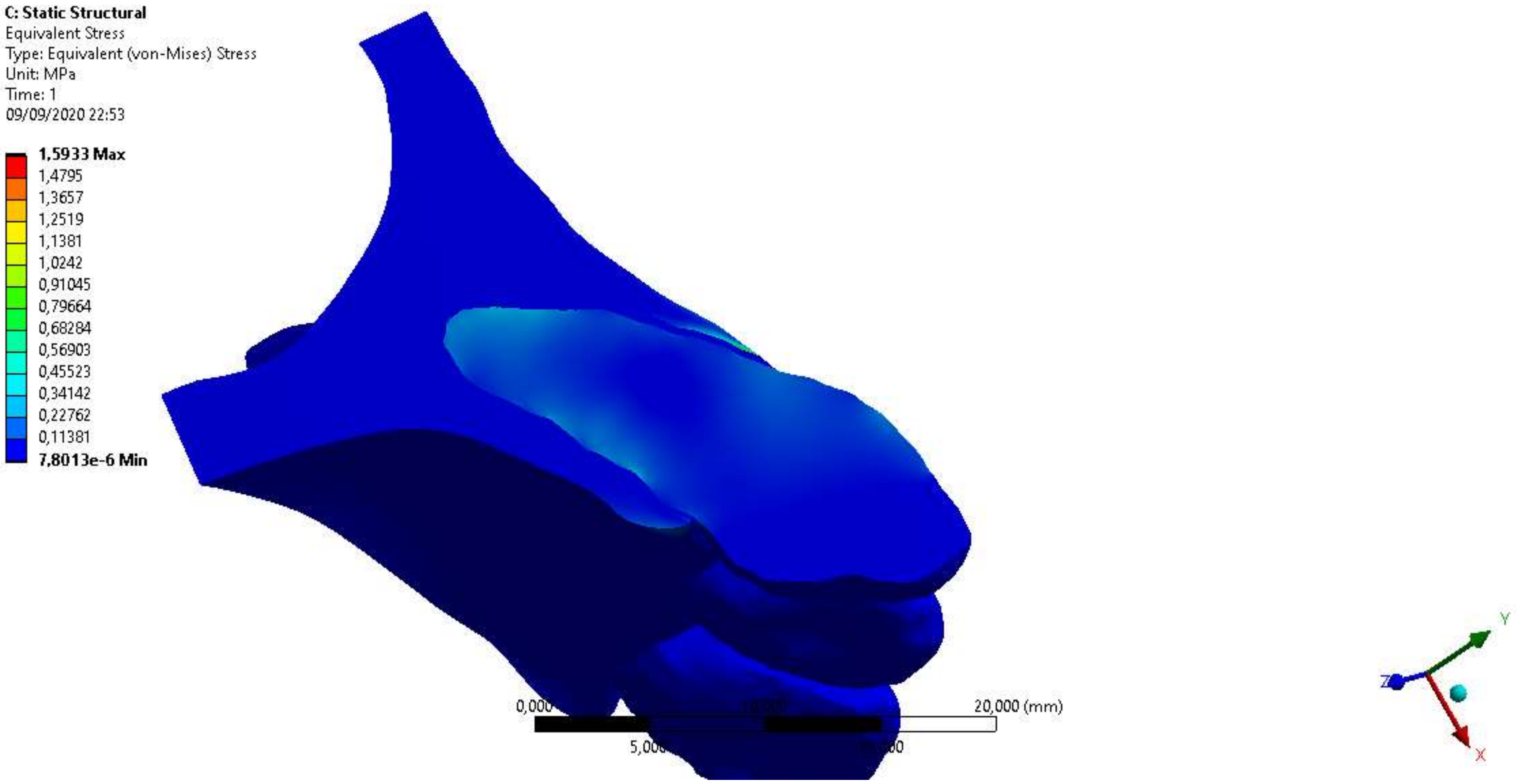
C: Static Structural
Equivalent Stress
Type: Equivalent (von-Mises) Stress
Unit: MPa
Time: 1
09/09/2020 22:53
1,5933 Max
1,4795
1,3657
1,2519
1,1381
1,0242
0,91045
0,79664
0,68284
0,56903
0,45523
0,34142
0,22762
0,11381
7,8013e-6 Min
0,000
5,000
20,000 (mm)
Y
Z
X

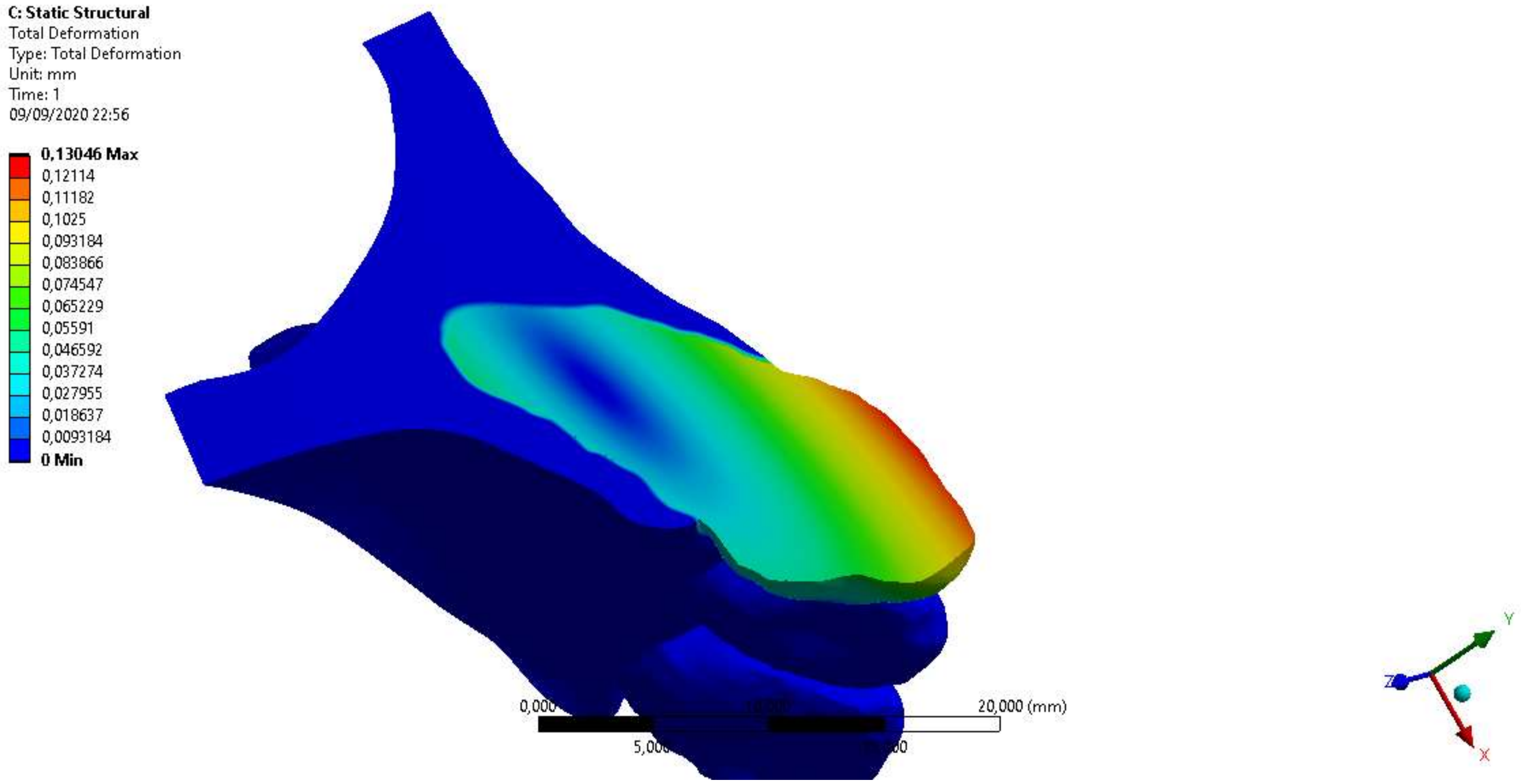
C: Static Structural
Total Deformation
Type: Total Deformation
Unit: mm
Time: 1
09/09/2020 22:56
0,13046 Max
0,12114
0,11182
0,1025
0,093184
0,083866
0,074547
0,065229
0,05591
0,046592
0,037274
0,027955
0,018637
0,0093184
0 Min
0,000
20,000 (mm)
Y
Z
X

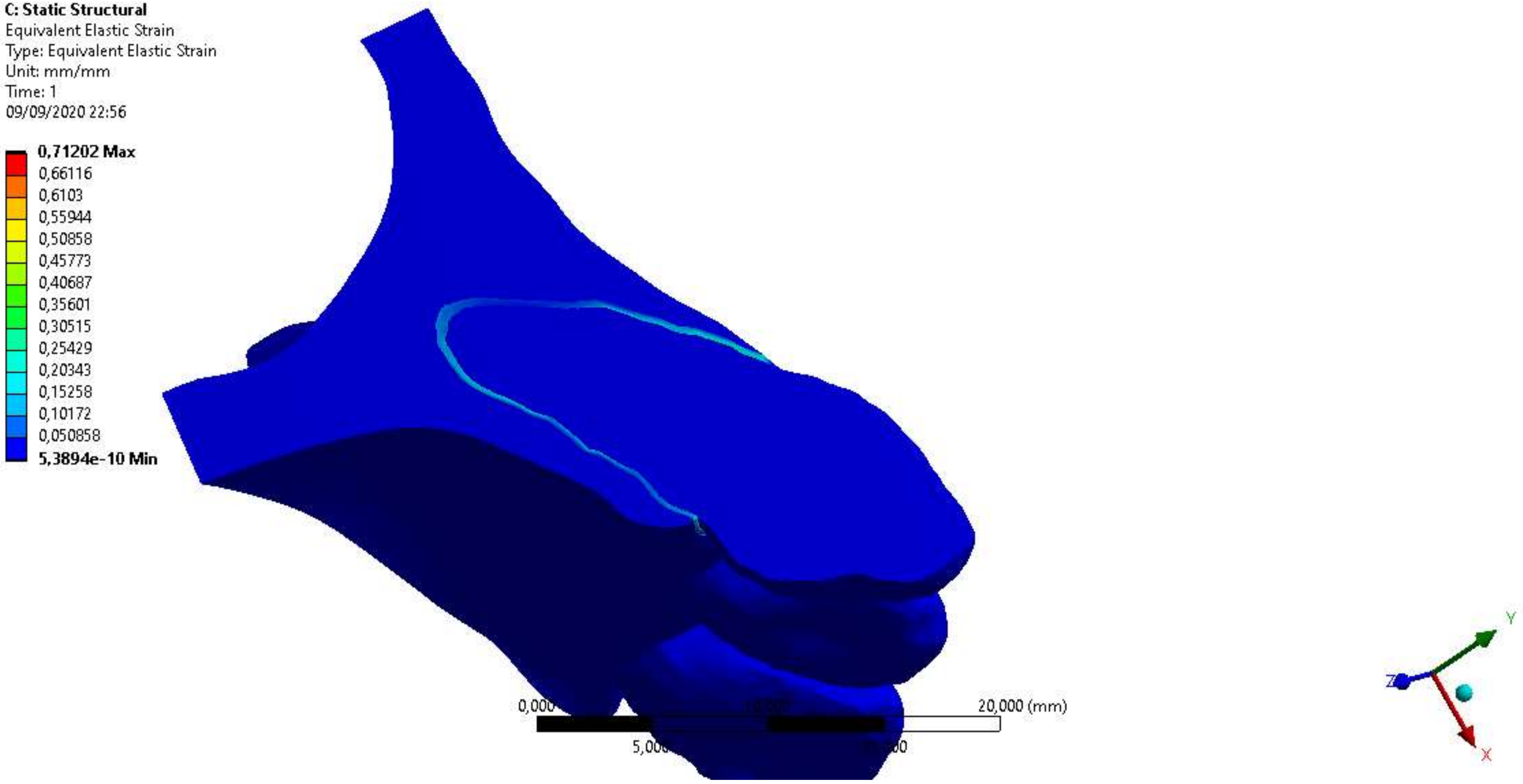
C: Static Structural
Equivalent Elastic Strain
Type: Equivalent Elastic Strain
Unit: mm/mm
Time: 1
09/09/2020 22:56
0,71202 Max
0,66116
0,6103
0,55944
0,50858
0,45773
0,40687
0,35601
0,30515
0,25429
0,20343
0,15258
0,10172
0,050858
5,3894e-10 Min
0,000
20,000 (mm)
Y
Z
X

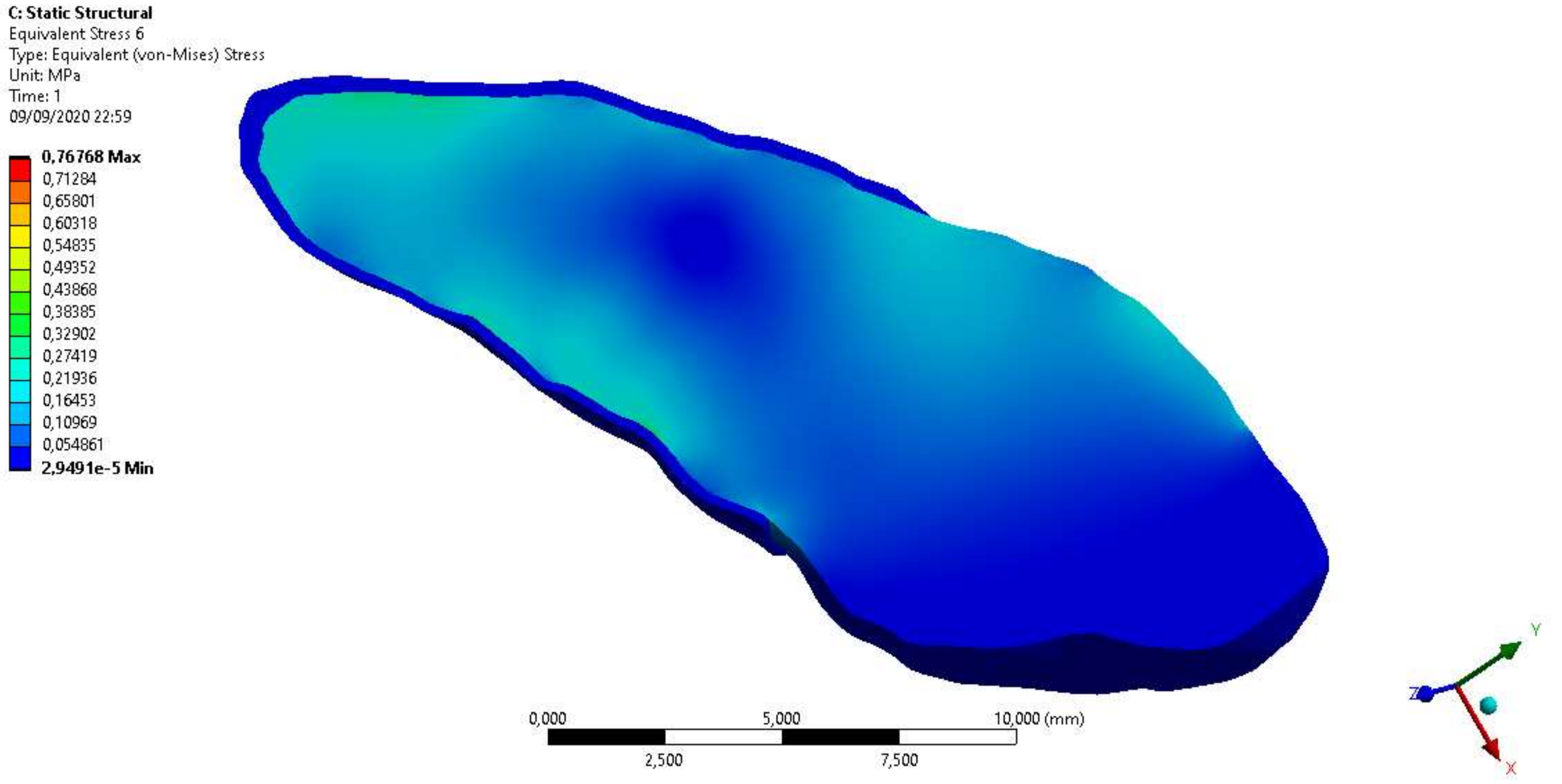
C: Static Structural
Equivalent Stress 6
Type: Equivalent (von-Mises) Stress
Unit: MPa
Time: 1
09/09/2020 22:59
0,76768 Max
0,71284
0,65801
0,60318
0,54835
0,49352
0,43868
0,38385
0,32902
0,27419
0,21936
0,16453
0,10969
0,054861
2,9491e-5 Min
0,000
5,000
10,000 (mm)
2,500
7,500
Y
Z
X

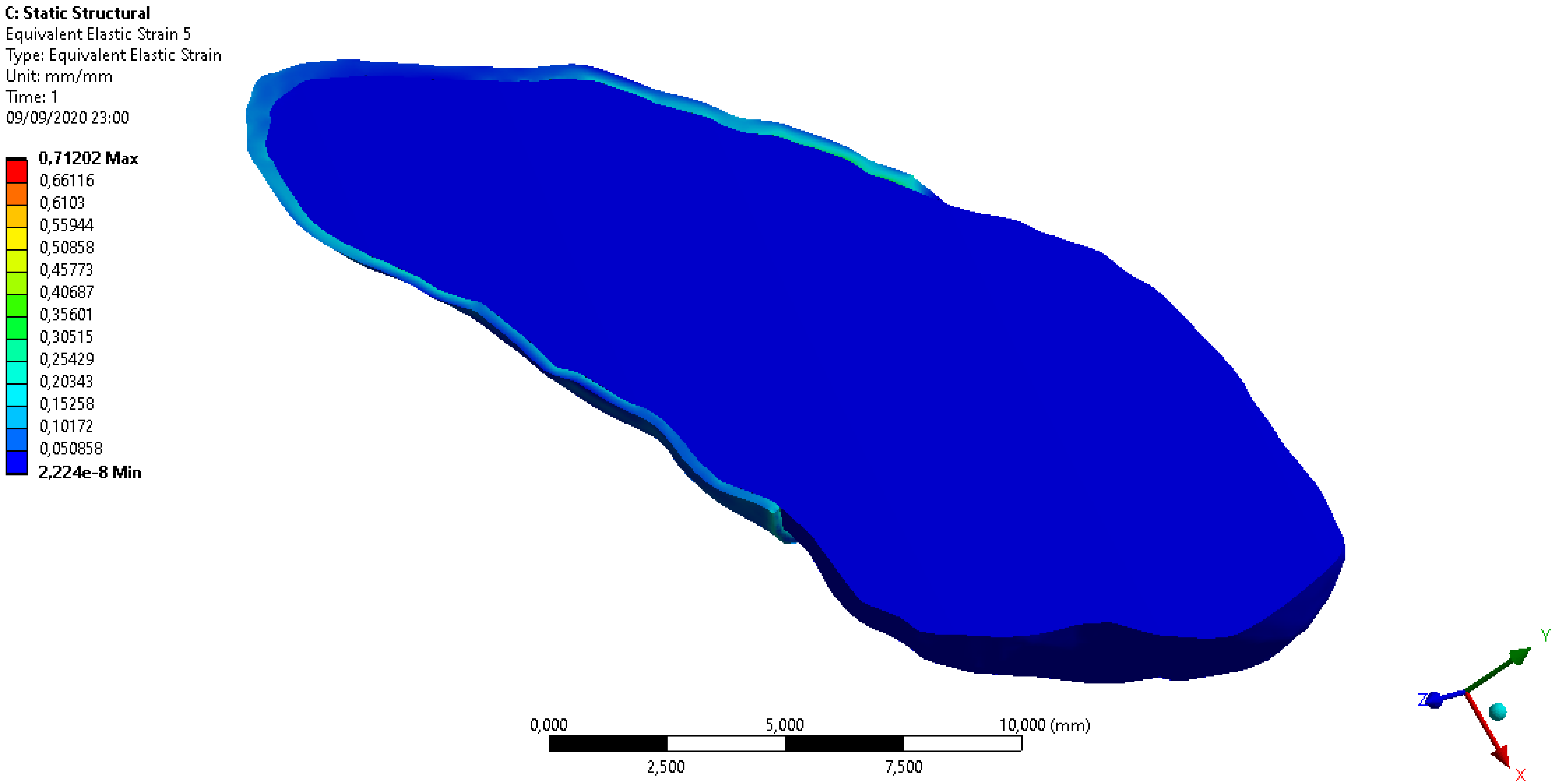
C: Static Structural
Equivalent Elastic Strain 5
Type: Equivalent Elastic Strain
Unit: mm/mm
Time: 1
09/09/2020 23:00
0,71202 Max
0,66116
0,6103
0,55944
0,50858
0,45773
0,40687
0,35601
0,30515
0,25429
0,20343
0,15258
0,10172
0,050858
2,224e-8 Min
0,000
5,000
10,000 (mm)
2,500
7,500
Y
Z
X

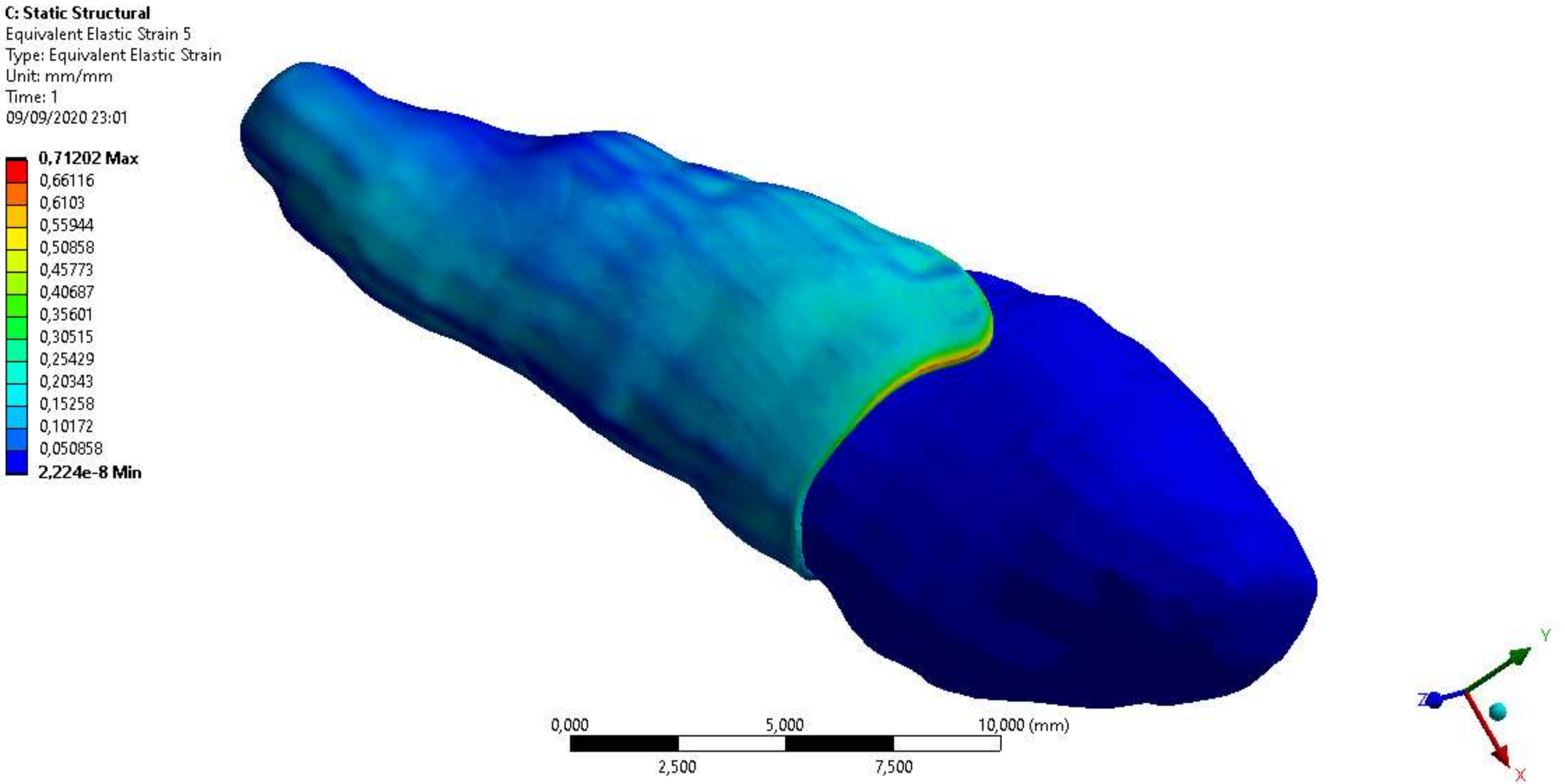
C: Static Structural
Equivalent Elastic Strain 5
Type: Equivalent Elastic Strain
Unit: mm/mm
Time: 1
09/09/2020 23:01
0,71202 Max
0,66116
0,6103
0,55944
0,50858
0,45773
0,40687
0,35601
0,30515
0,25429
0,20343
0,15258
0,10172
0,050858
2,224e-8 Min
0,000
5,000
10,000 (mm)
2,500
7,500
Y
Z
X

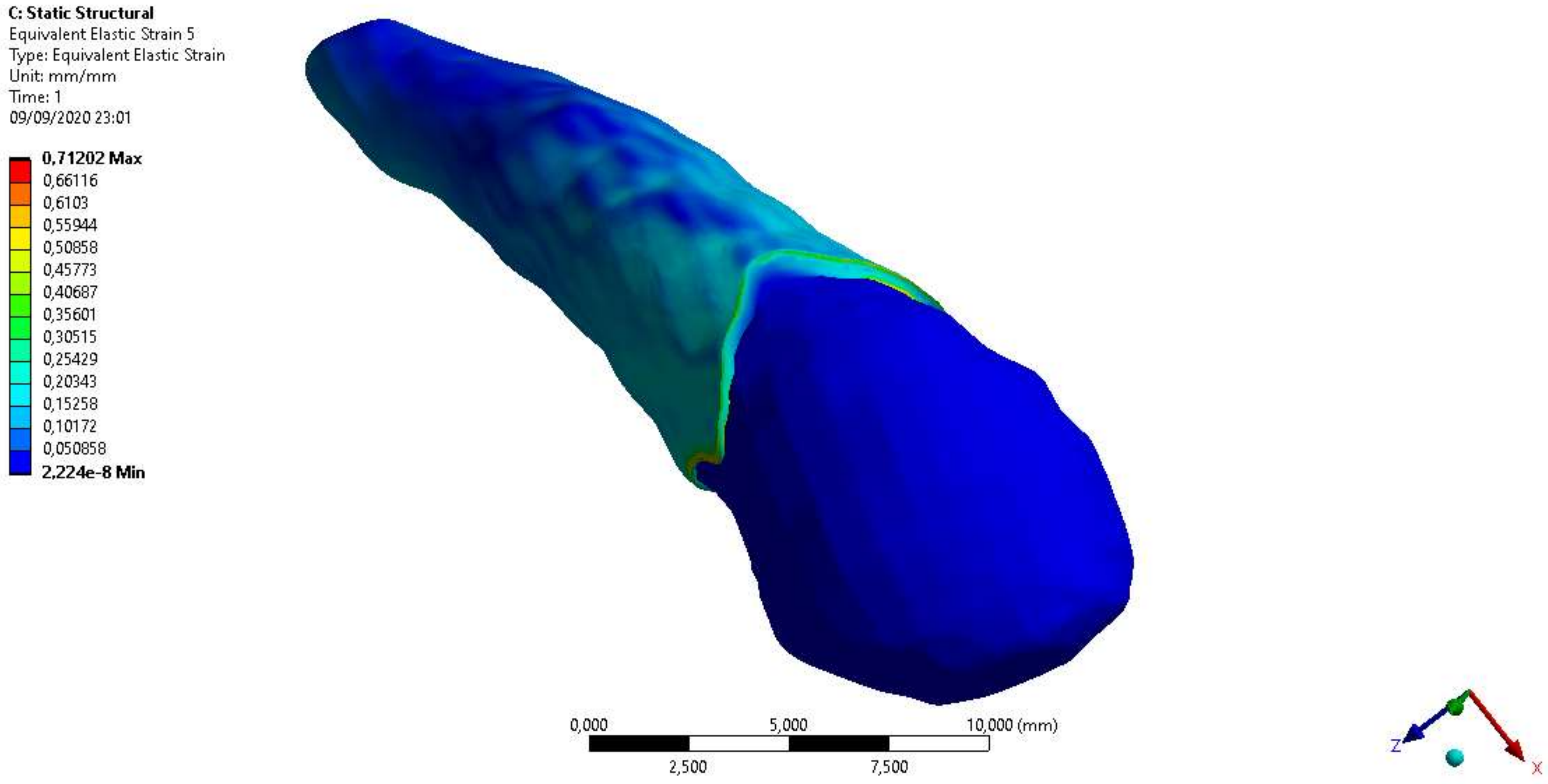
C: Static Structural
Equivalent Elastic Strain 5
Type: Equivalent Elastic Strain
Unit: mm/mm
Time: 1
09/09/2020 23:01
0,71202 Max
0,66116
0,6103
0,55944
0,50858
0,45773
0,40687
0,35601
0,30515
0,25429
0,20343
0,15258
0,10172
0,050858
2,224e-8 Min
0,000
5,000
10,000 (mm)
2,500
7,500
Z
X

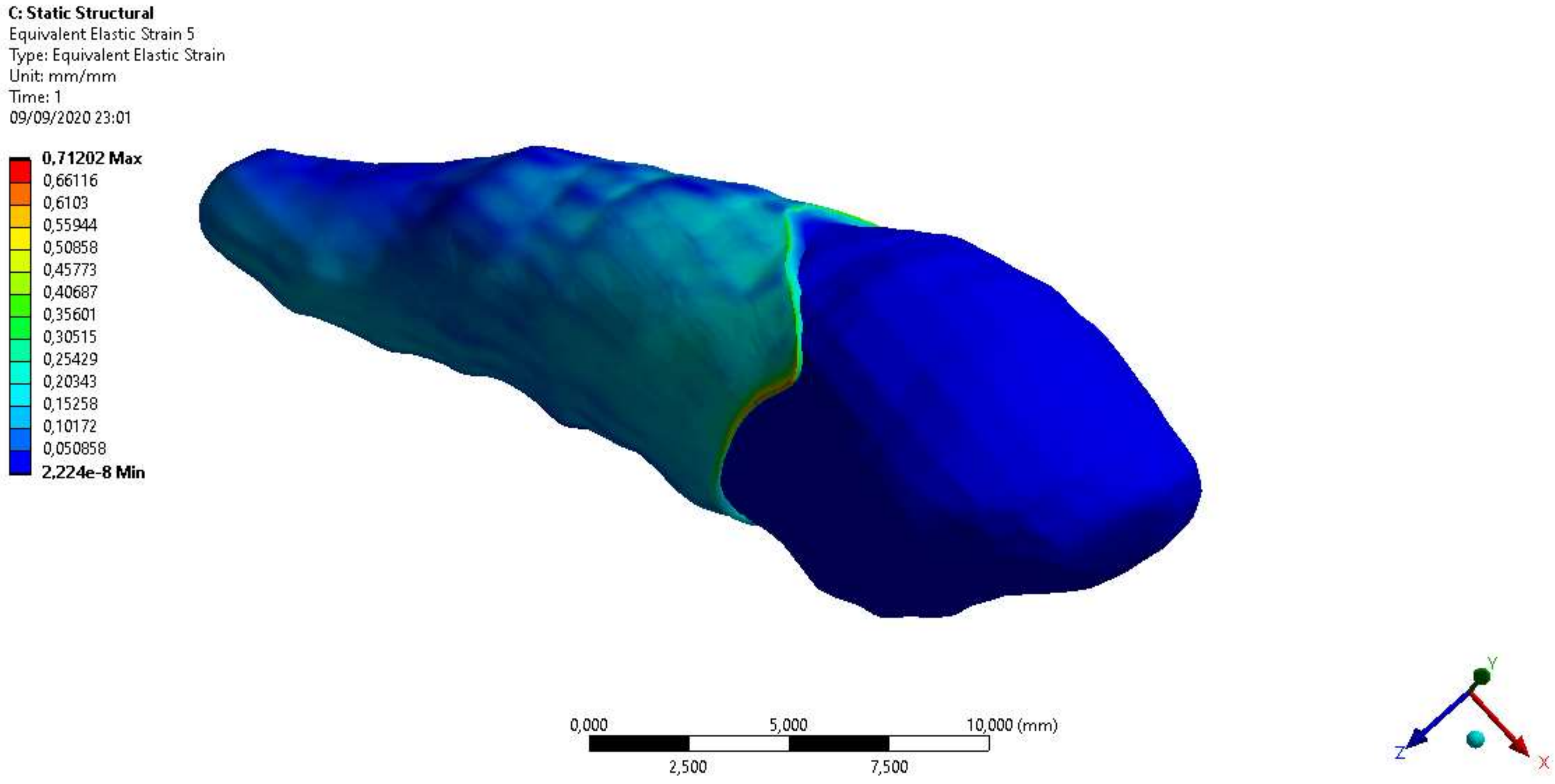
C: Static Structural
Equivalent Elastic Strain 5
Type: Equivalent Elastic Strain
Unit: mm/mm
Time: 1
09/09/2020 23:01
0,71202 Max
0,66116
0,6103
0,55944
0,50858
0,45773
0,40687
0,35601
0,30515
0,25429
0,20343
0,15258
0,10172
0,050858
2,224e-8 Min
0,000
5,000
10,000 (mm)
2,500
7,500
Y
Z
X

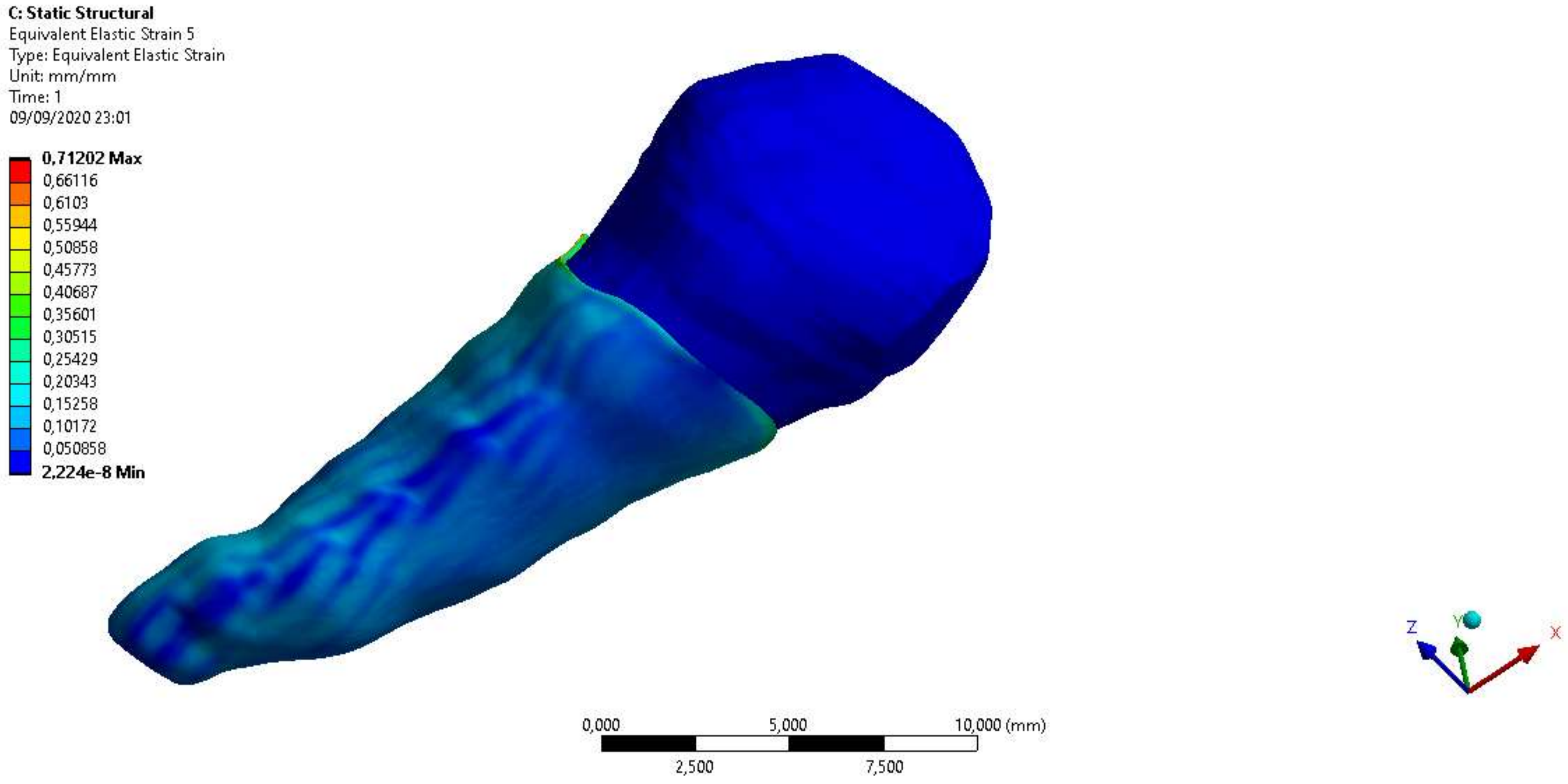

C: Static Structural
Equivalent Elastic Strain 5
Type: Equivalent Elastic Strain
Unit: mm/mm
Time: 1
09/09/2020 23:01
0,71202 Max
0,66116
0,6103
0,55944
0,50858
0,45773
0,40687
0,35601
0,30515
0,25429
0,20343
0,15258
0,10172
0,050858
2,224e-8 Min
0,000
5,000
10,000 (mm)
2,500
7,500
Z
Y
X

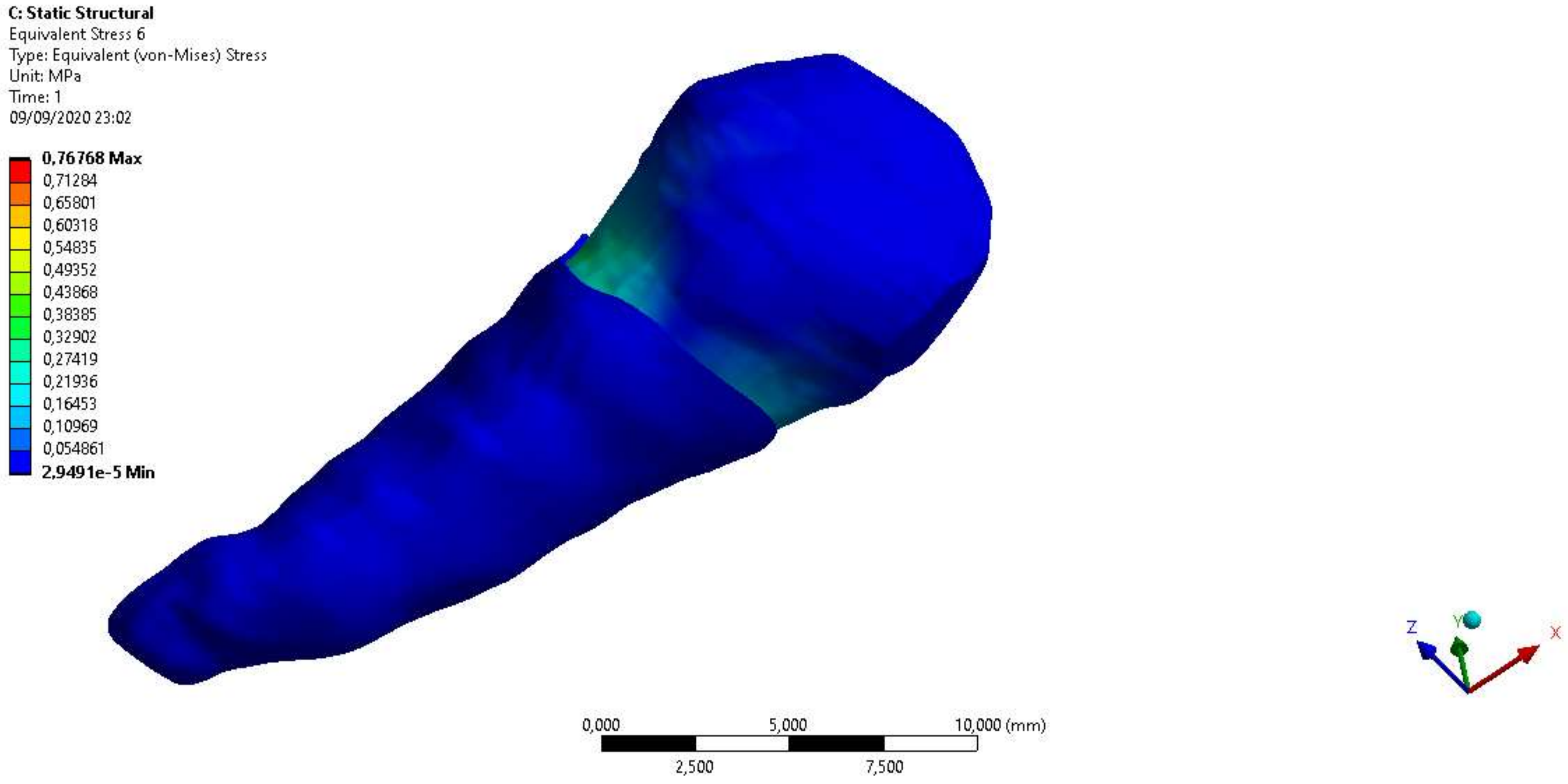
C: Static Structural
Equivalent Stress 6
Type: Equivalent (von-Mises) Stress
Unit: MPa
Time: 1
09/09/2020 23:02
0,76768 Max
0,71284
0,65801
0,60318
0,54835
0,49352
0,43868
0,38385
0,32902
0,27419
0,21936
0,16453
0,10969
0,054861
2,9491e-5 Min
0,000
5,000
10,000 (mm)
2,500
7,500
Z
Y
X

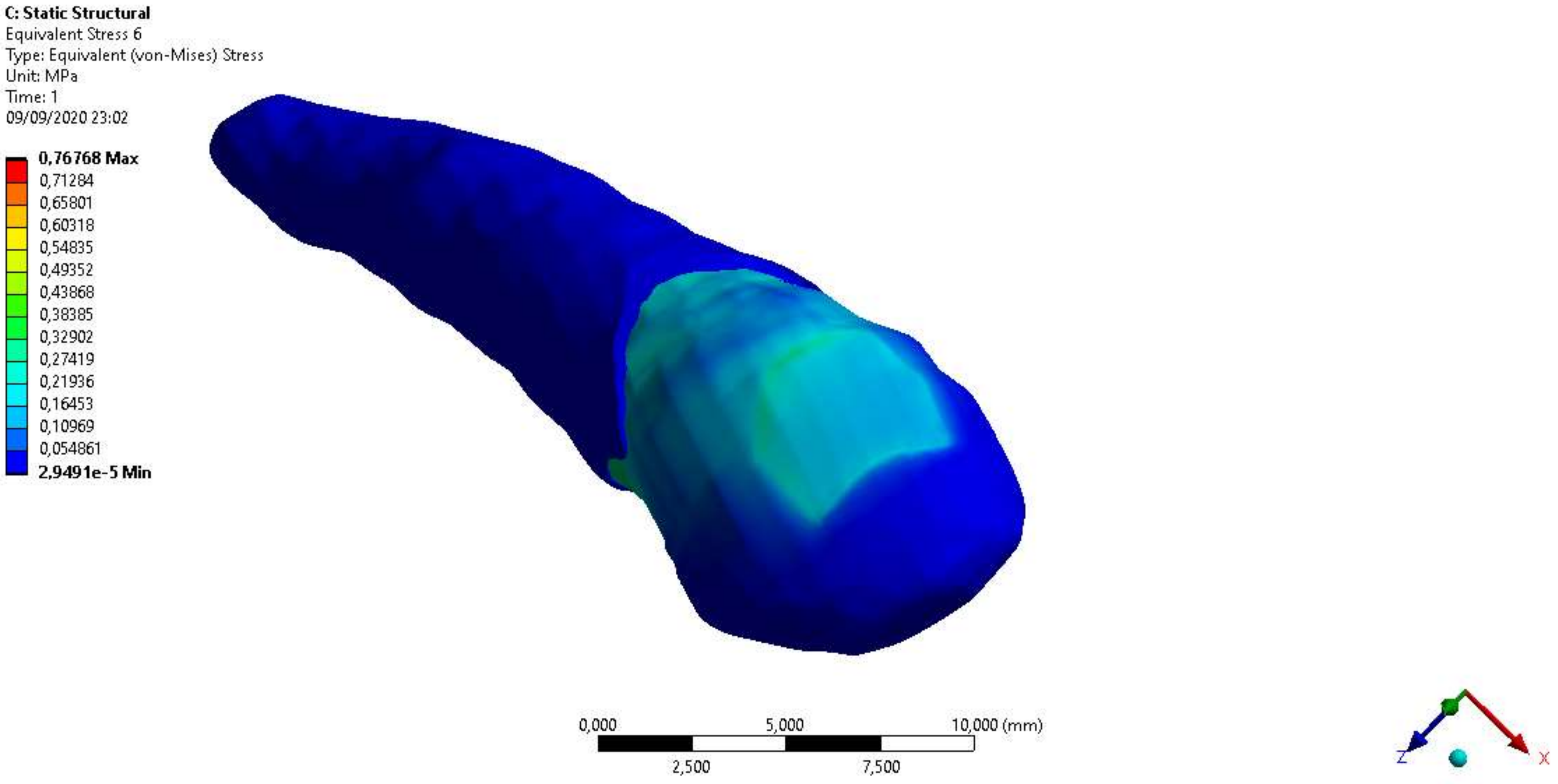
C: Static Structural
Equivalent Stress 6
Type: Equivalent (von-Mises) Stress
Unit: MPa
Time: 1
09/09/2020 23:02
0,76768 Max
0,71284
0,65801
0,60318
0,54835
0,49352
0,43868
0,38385
0,32902
0,27419
0,21936
0,16453
0,10969
0,054861
2,9491e-5 Min
0,000
5,000
10,000 (mm)
2,500
7,500
Z
X

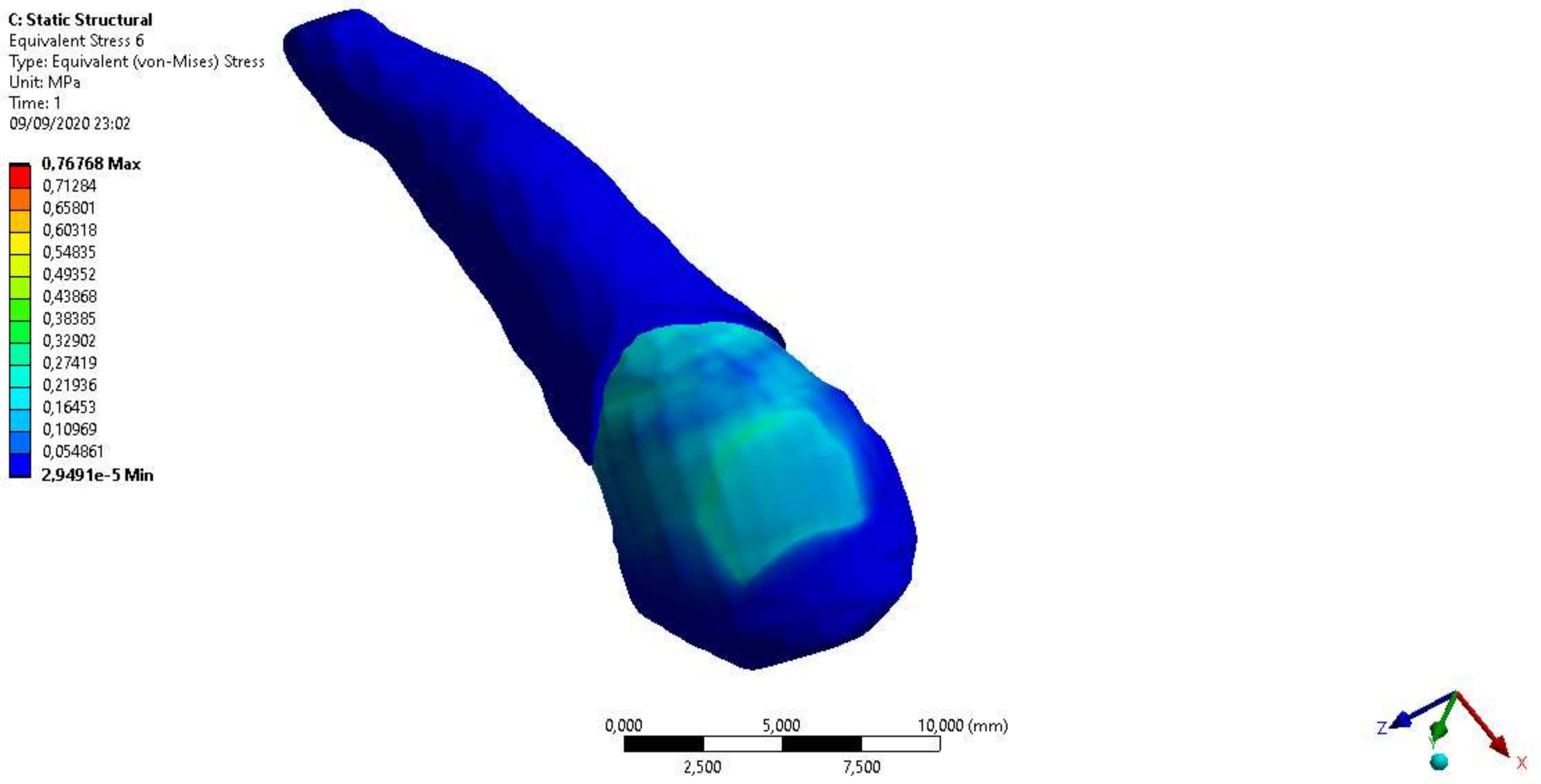
C: Static Structural
Equivalent Stress 6
Type: Equivalent (von-Mises) Stress
Unit: MPa
Time: 1
09/09/2020 23:02
0,76768 Max
0,71284
0,65801
0,60318
0,54835
0,49352
0,43868
0,38385
0,32902
0,27419
0,21936
0,16453
0,10969
0,054861
2,9491e-5 Min
0,000
5,000
10,000 (mm)
2,500
7,500
Z
Y
X

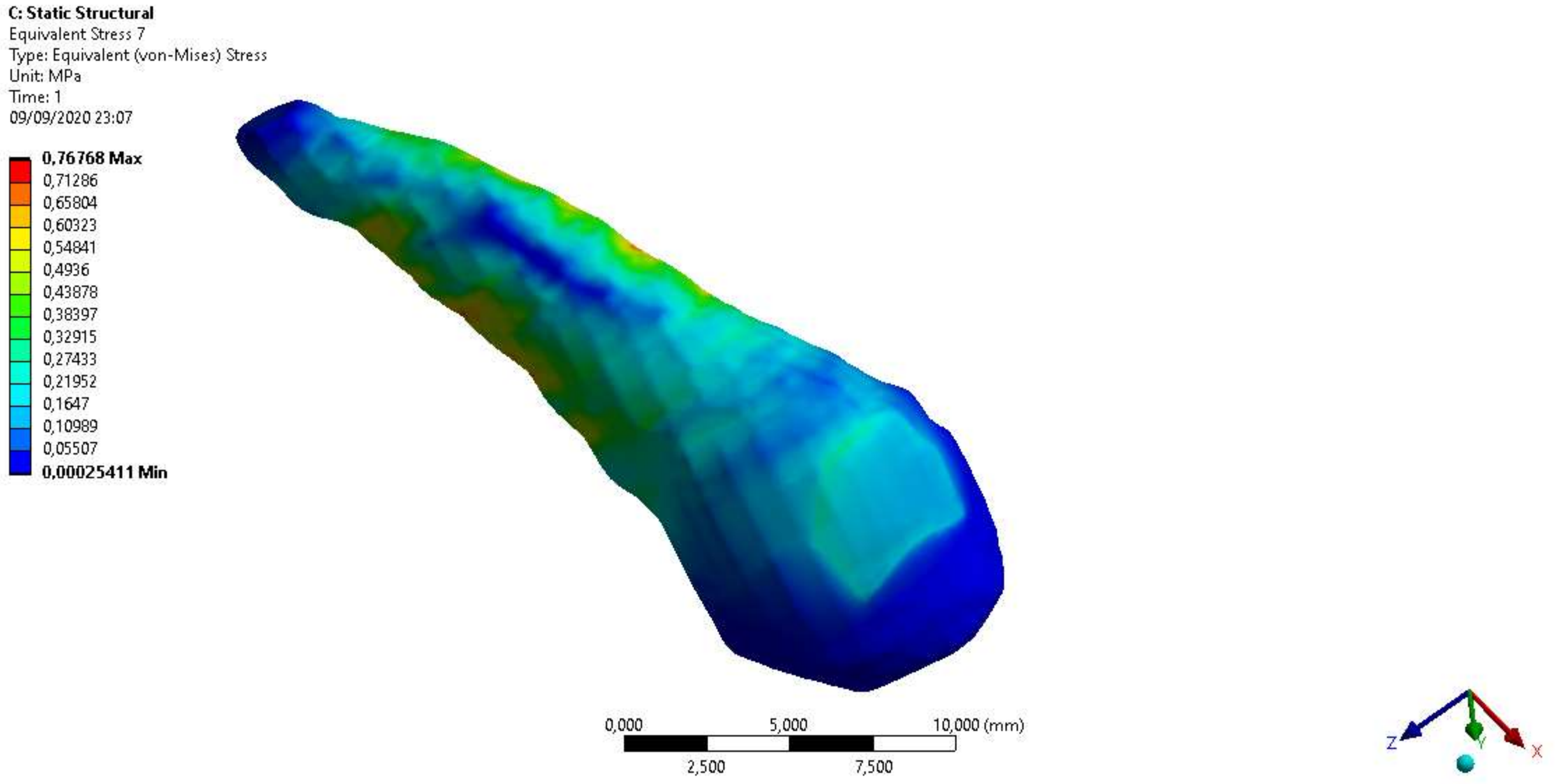
C: Static Structural
Equivalent Stress 7
Type: Equivalent (von-Mises) Stress
Unit: MPa
Time: 1
09/09/2020 23:07
0,76768 Max
0,71286
0,65804
0,60323
0,54841
0,4936
0,43878
0,38397
0,32915
0,27433
0,21952
0,1647
0,10989
0,05507
0,00025411 Min
0,000
5,000
10,000 (mm)
2,500
7,500
Z
Y
X

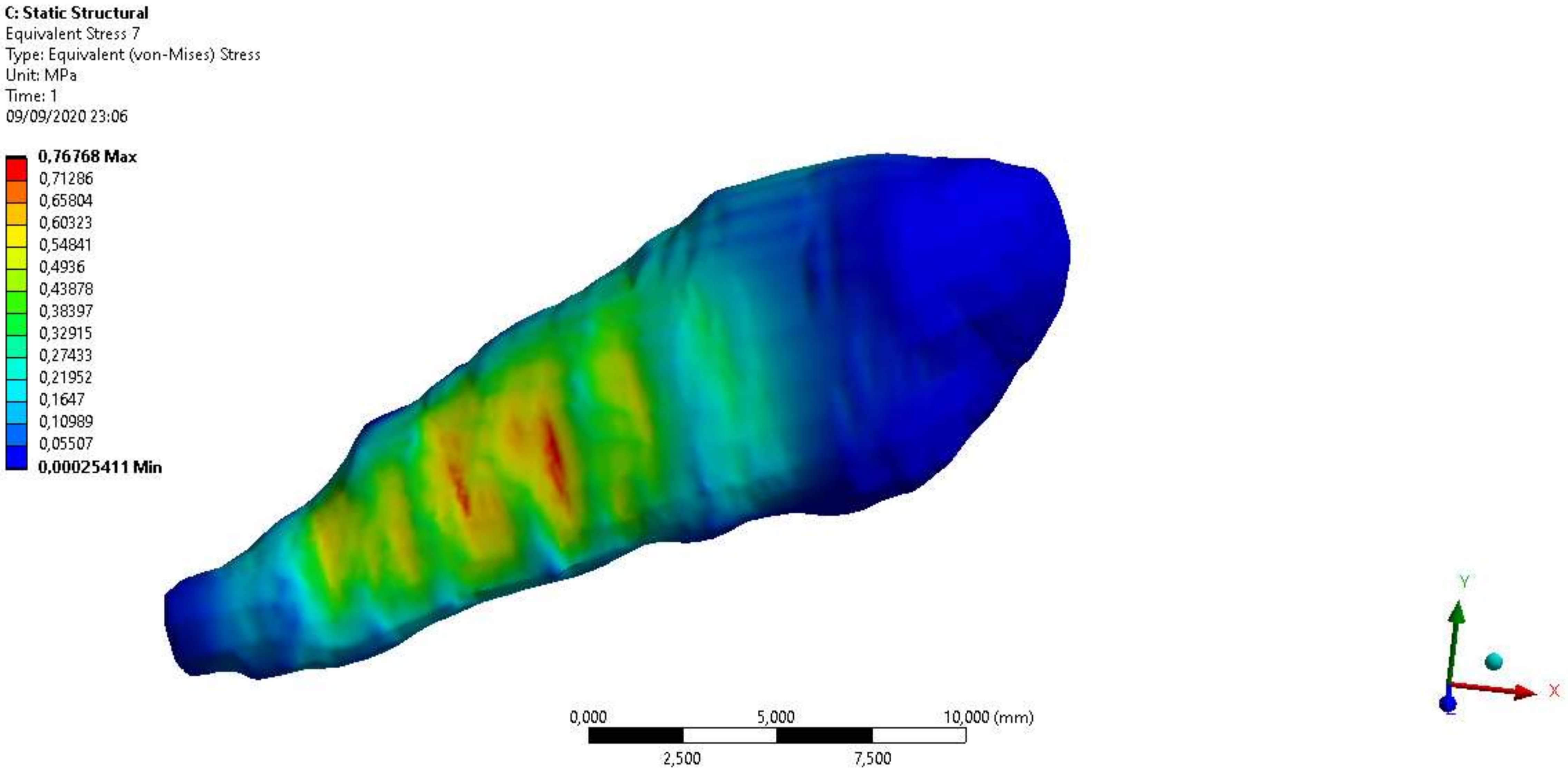
C: Static Structural
Equivalent Stress 7
Type: Equivalent (von-Mises) Stress
Unit: MPa
Time: 1
09/09/2020 23:06
0,76768 Max
0,71286
0,65804
0,60323
0,54841
0,4936
0,43878
0,38397
0,32915
0,27433
0,21952
0,1647
0,10989
0,05507
0,00025411 Min
0,000
5,000
10,000 (mm)
2,500
7,500
Y
X

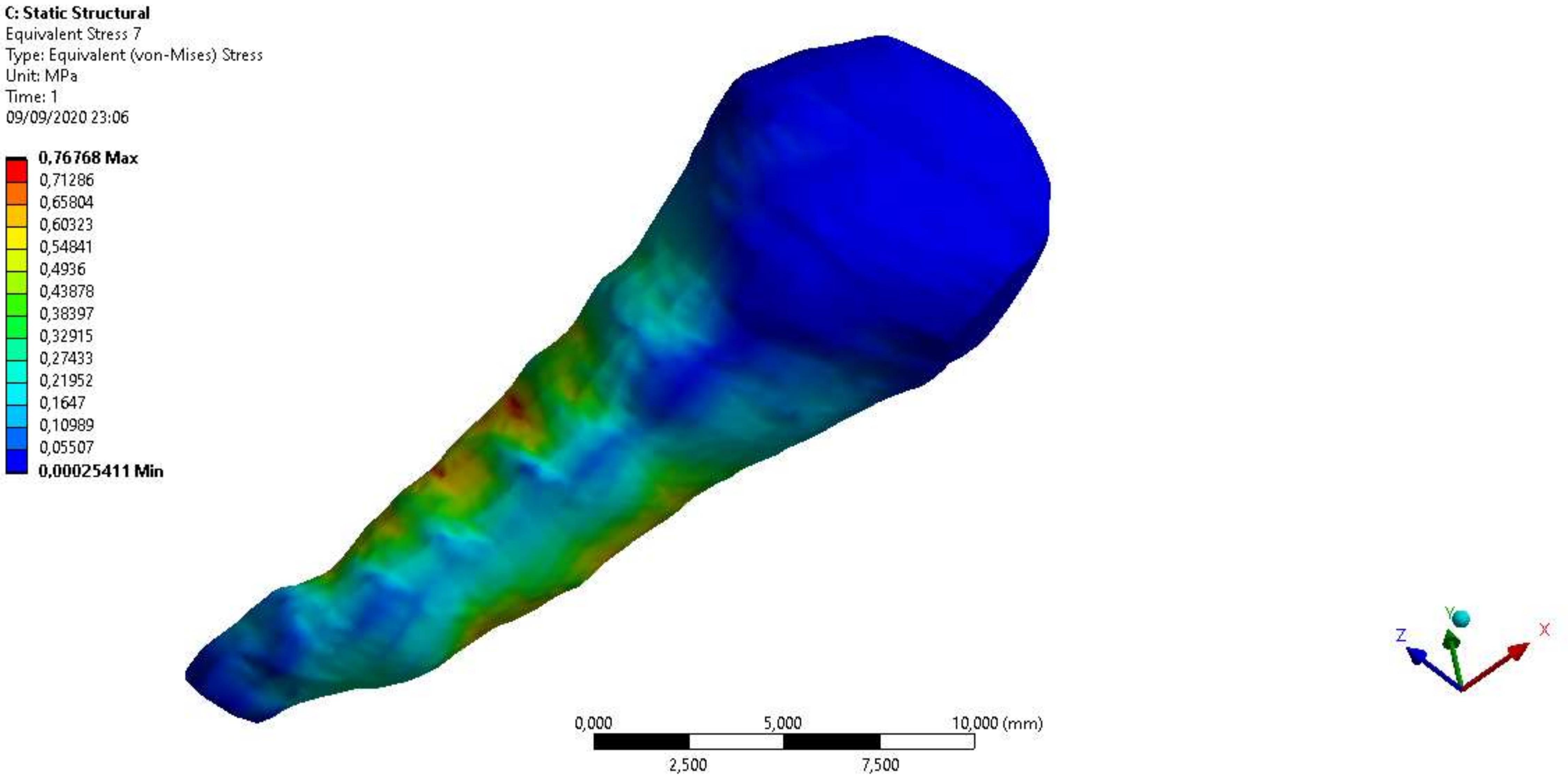
C: Static Structural
Equivalent Stress 7
Type: Equivalent (von-Mises) Stress
Unit: MPa
Time: 1
09/09/2020 23:06
0,76768 Max
0,71286
0,65804
0,60323
0,54841
0,4936
0,43878
0,38397
0,32915
0,27433
0,21952
0,1647
0,10989
0,05507
0,00025411 Min
0,000
5,000
10,000 (mm)
2,500
7,500
Z
Y
X

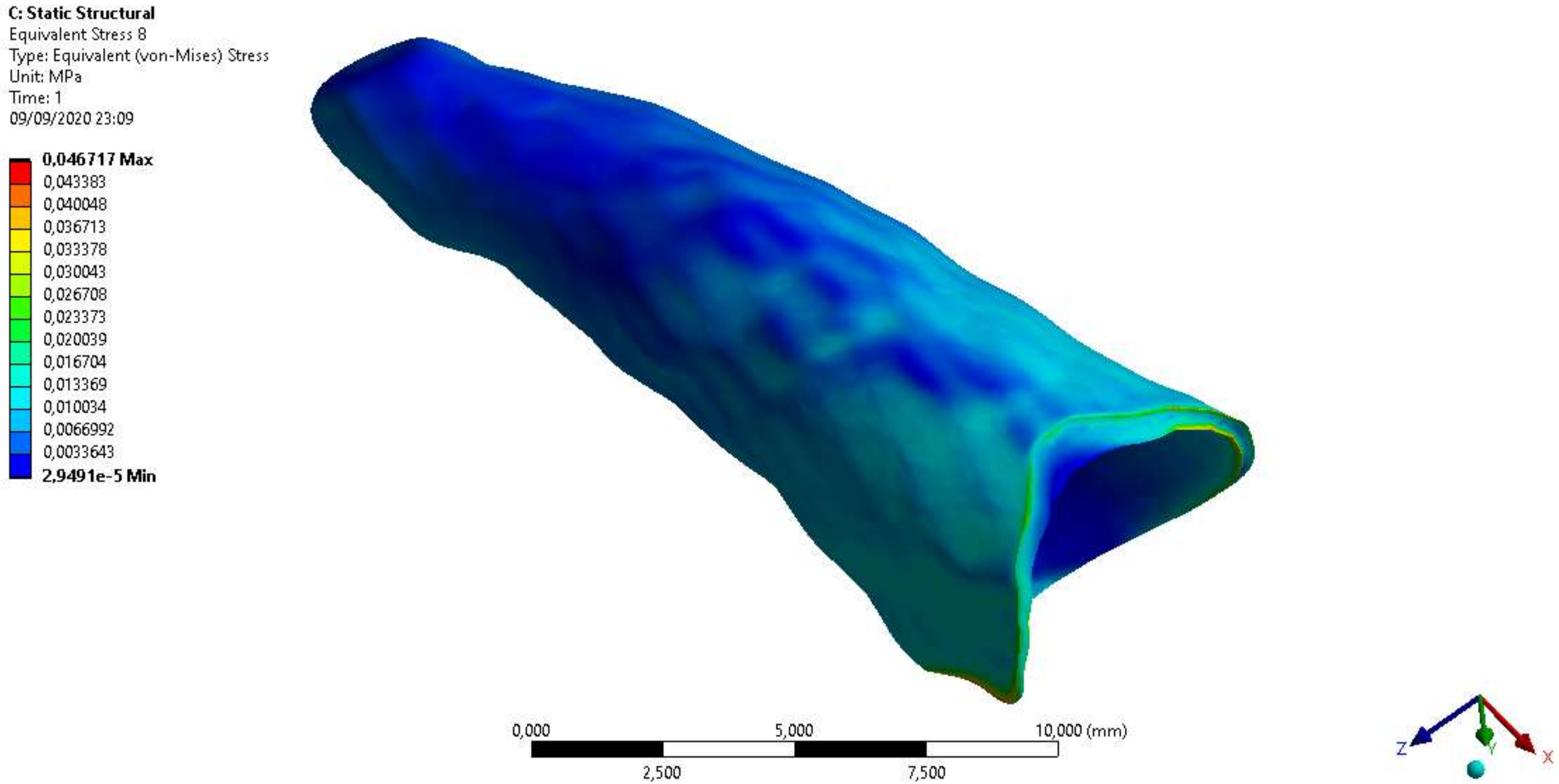

C: Static Structural
Equivalent Stress 8
Type: Equivalent (von-Mises) Stress
Unit: MPa
Time: 1
09/09/2020 23:09
0,046717 Max
0,043383
0,040048
0,036713
0,033378
0,030043
0,026708
0,023373
0,020039
0,016704
0,013369
0,010034
0,0066992
0,0033643
2,9491e-5 Min
0,000
5,000
10,000 (mm)
2,500
7,500
Z
Y
X

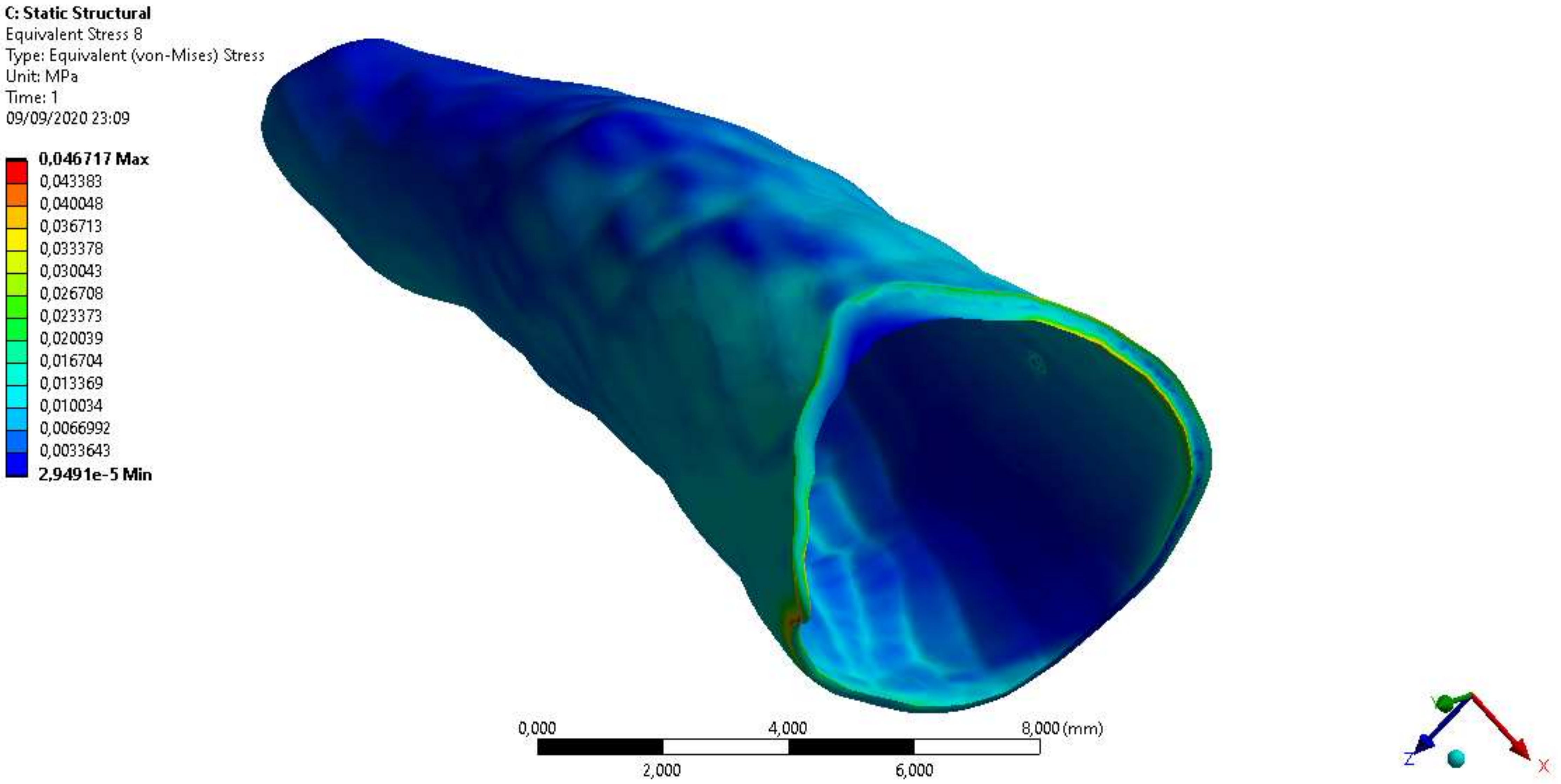
C: Static Structural
Equivalent Stress 8
Type: Equivalent (von-Mises) Stress
Unit: MPa
Time: 1
09/09/2020 23:09
0,046717 Max
0,043383
0,040048
0,036713
0,033378
0,030043
0,026708
0,023373
0,020039
0,016704
0,013369
0,010034
0,0066992
0,0033643
2,9491e-5 Min
0,000
4,000
8,000 (mm)
2,000
6,000
Z
X

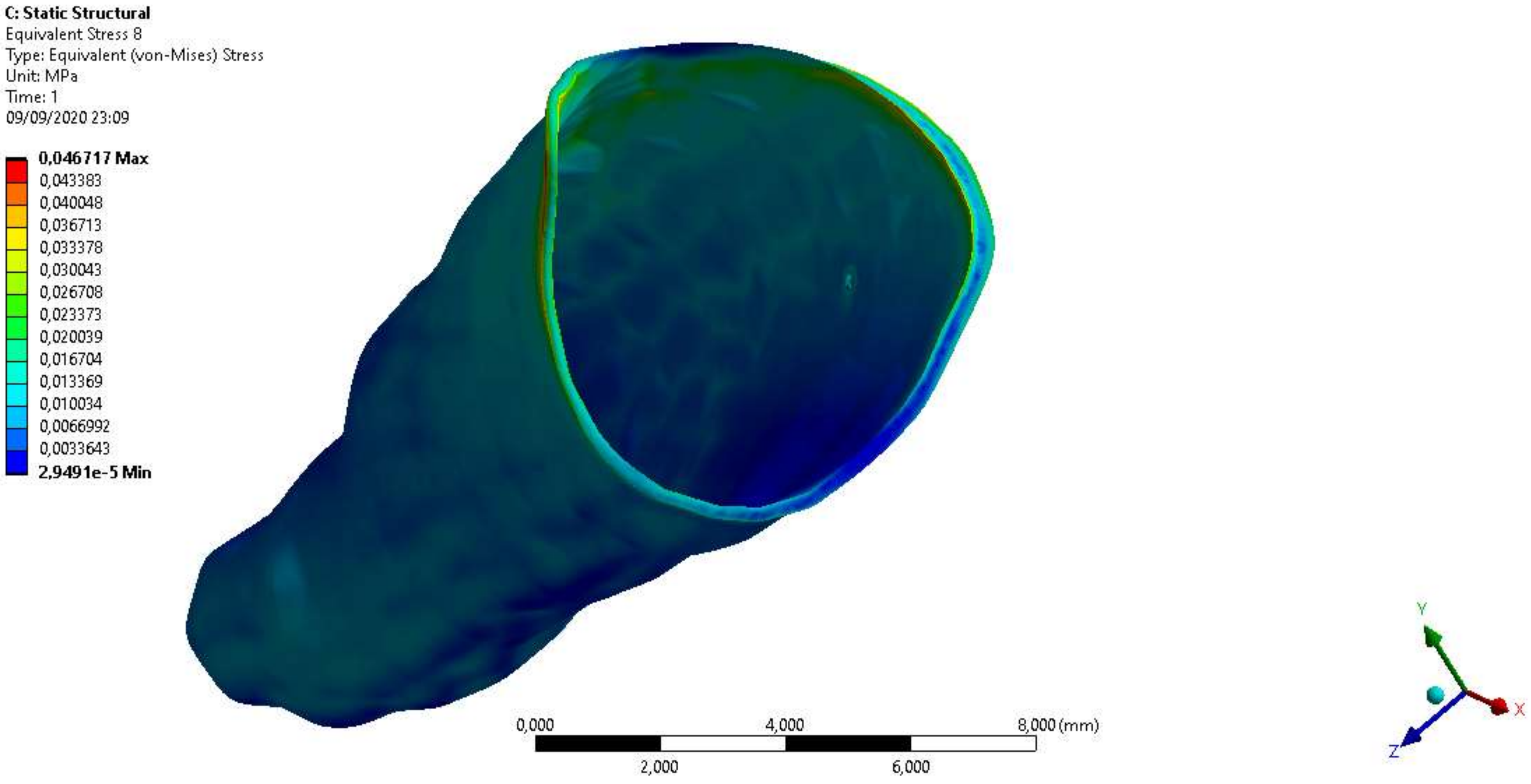
C: Static Structural
Equivalent Stress 8
Type: Equivalent (von-Mises) Stress
Unit: MPa
Time: 1
09/09/2020 23:09
0,046717 Max
0,043383
0,040048
0,036713
0,033378
0,030043
0,026708
0,023373
0,020039
0,016704
0,013369
0,010034
0,0066992
0,0033643
2,9491e-5 Min
0,000
4,000
8,000 (mm)
2,000
6,000
Y
X
Z

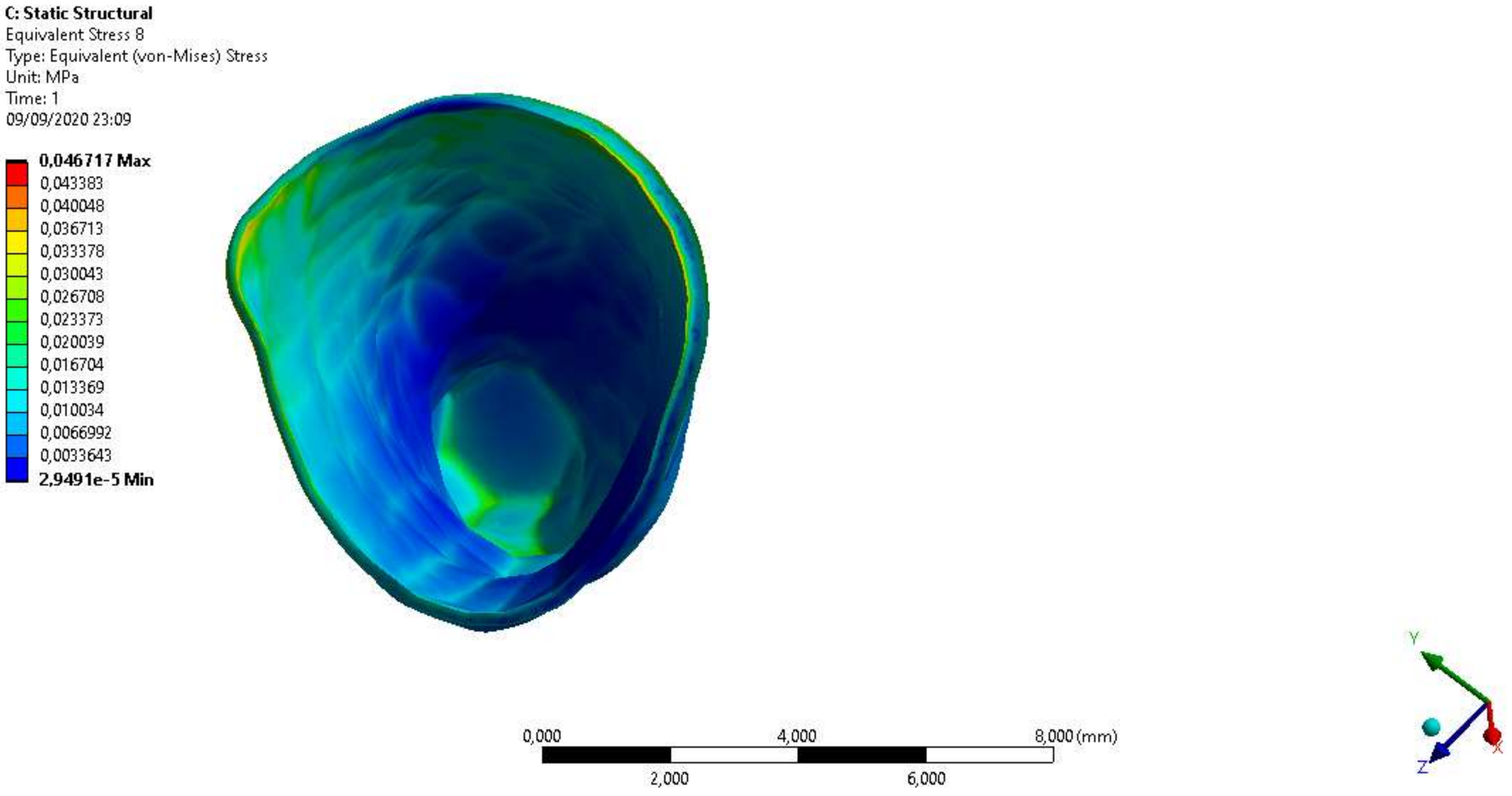
C: Static Structural
Equivalent Stress 8
Type: Equivalent (von-Mises) Stress
Unit: MPa
Time: 1
09/09/2020 23:09
0,046717 Max
0,043383
0,040048
0,036713
0,033378
0,030043
0,026708
0,023373
0,020039
0,016704
0,013369
0,010034
0,0066992
0,0033643
2,9491e-5 Min
0,000
4,000
8,000 (mm)
2,000
6,000
Y
Z
X

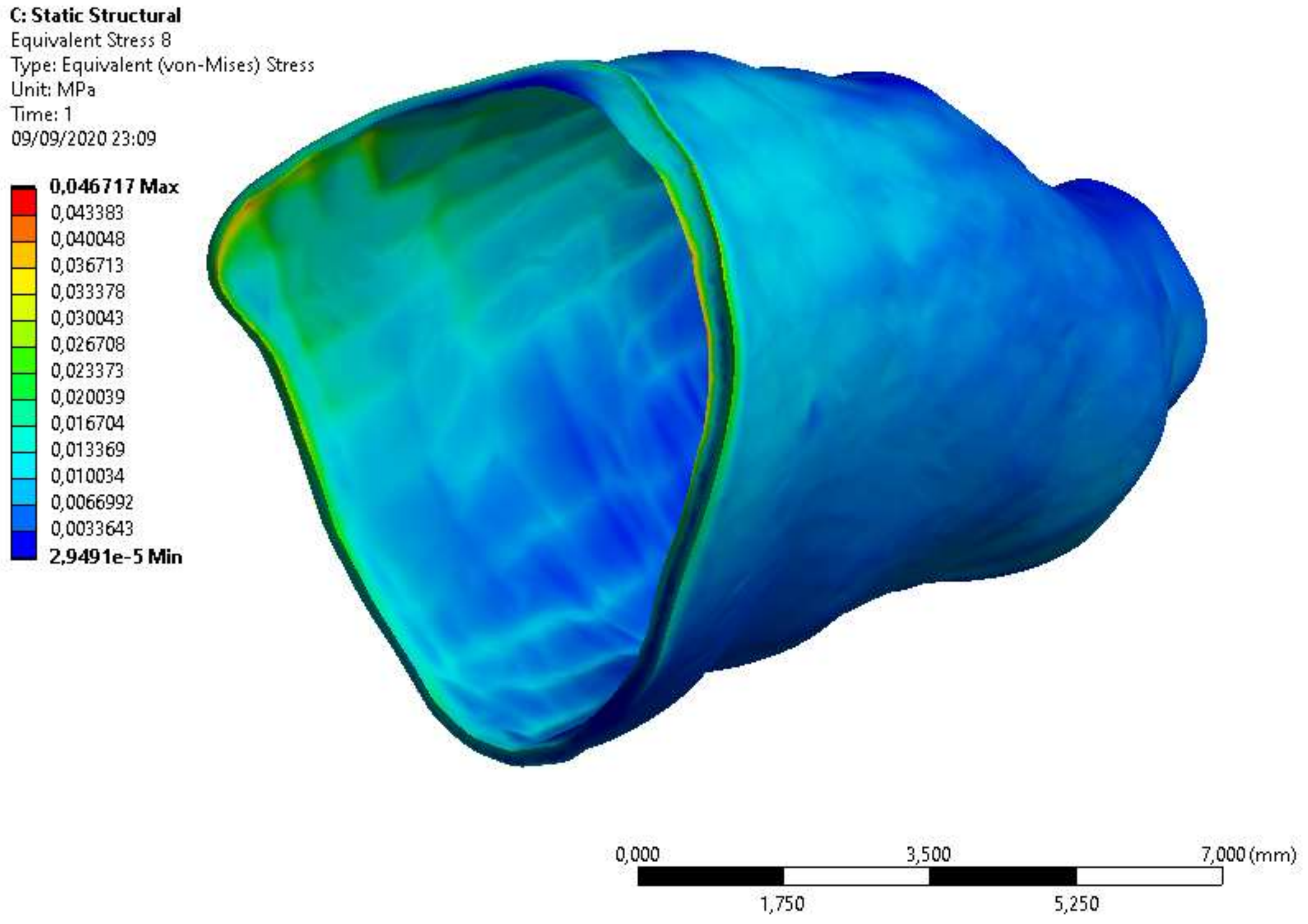
C: Static Structural
Equivalent Stress 8
Type: Equivalent (von-Mises) Stress
Unit: MPa
Time: 1
09/09/2020 23:09
0,046717 Max
0,043383
0,040048
0,036713
0,033378
0,030043
0,026708
0,023373
0,020039
0,016704
0,013369
0,010034
0,0066992
0,0033643
2,9491e-5 Min
0,000
3,500
7,000 (mm)
1,750
5,250
Y
Z
X

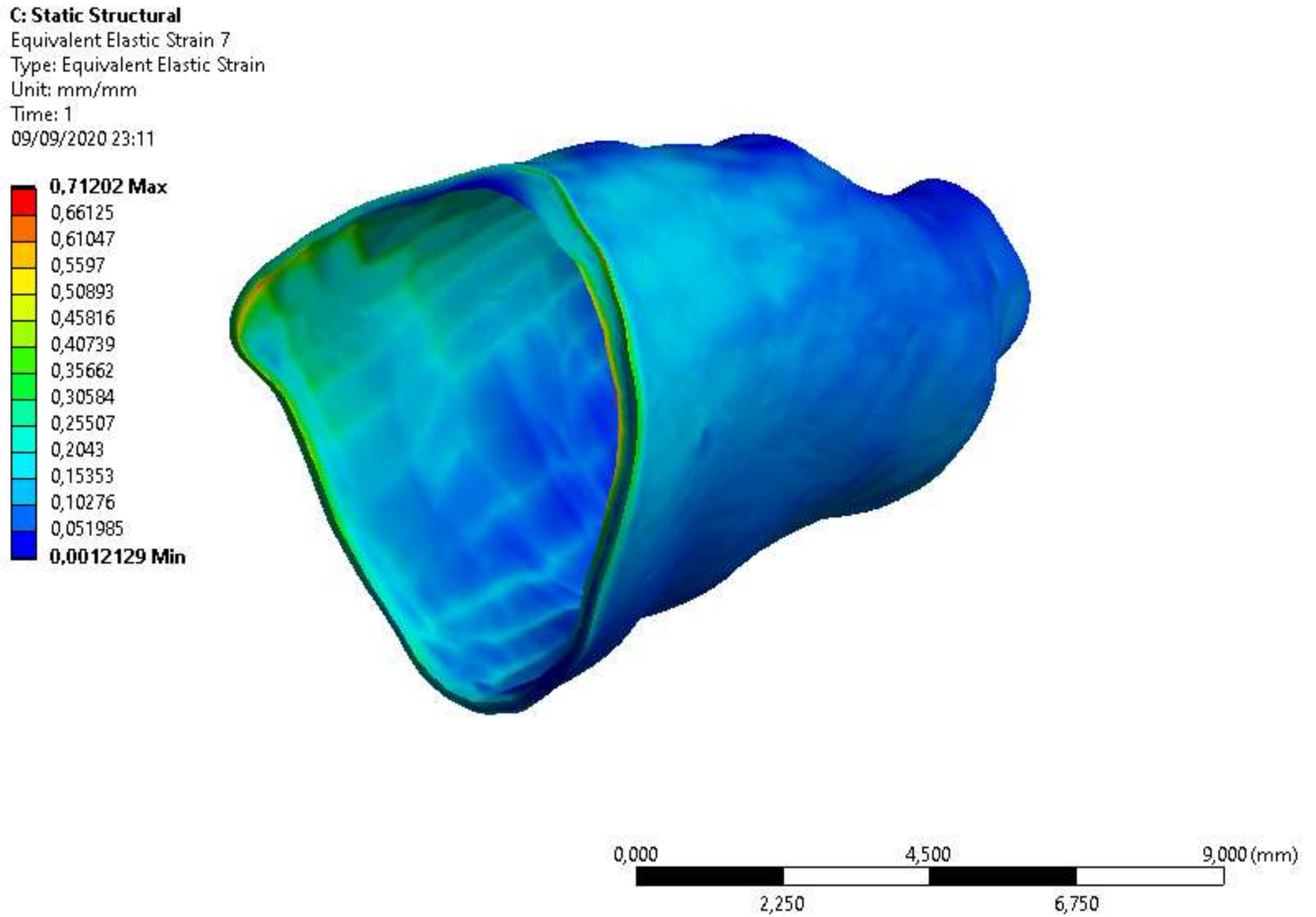
C: Static Structural
Equivalent Elastic Strain 7
Type: Equivalent Elastic Strain
Unit: mm/mm
Time: 1
09/09/2020 23:11
0,71202 Max
0,66125
0,61047
0,5597
0,50893
0,45816
0,40739
0,35662
0,30584
0,25507
0,2043
0,15353
0,10276
0,051985
0,0012129 Min
0,000
4,500
9,000 (mm)
2,250
6,750
Y
Z
X

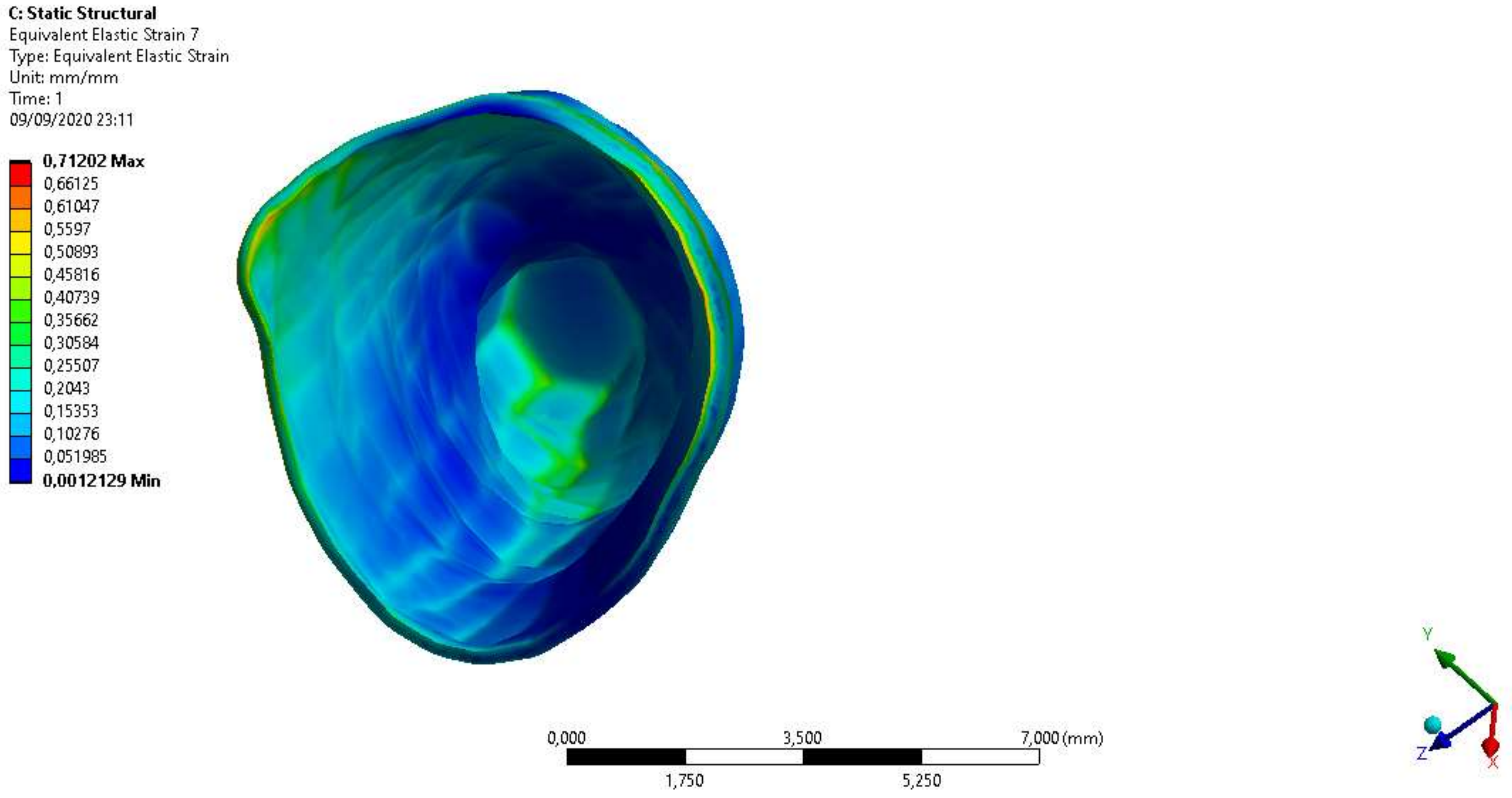
C: Static Structural
Equivalent Elastic Strain 7
Type: Equivalent Elastic Strain
Unit: mm/mm
Time: 1
09/09/2020 23:11
0,71202 Max
0,66125
0,61047
0,5597
0,50893
0,45816
0,40739
0,35662
0,30584
0,25507
0,2043
0,15353
0,10276
0,051985
0,0012129 Min
0,000
3,500
7,000 (mm)
1,750
5,250
Y
Z
X

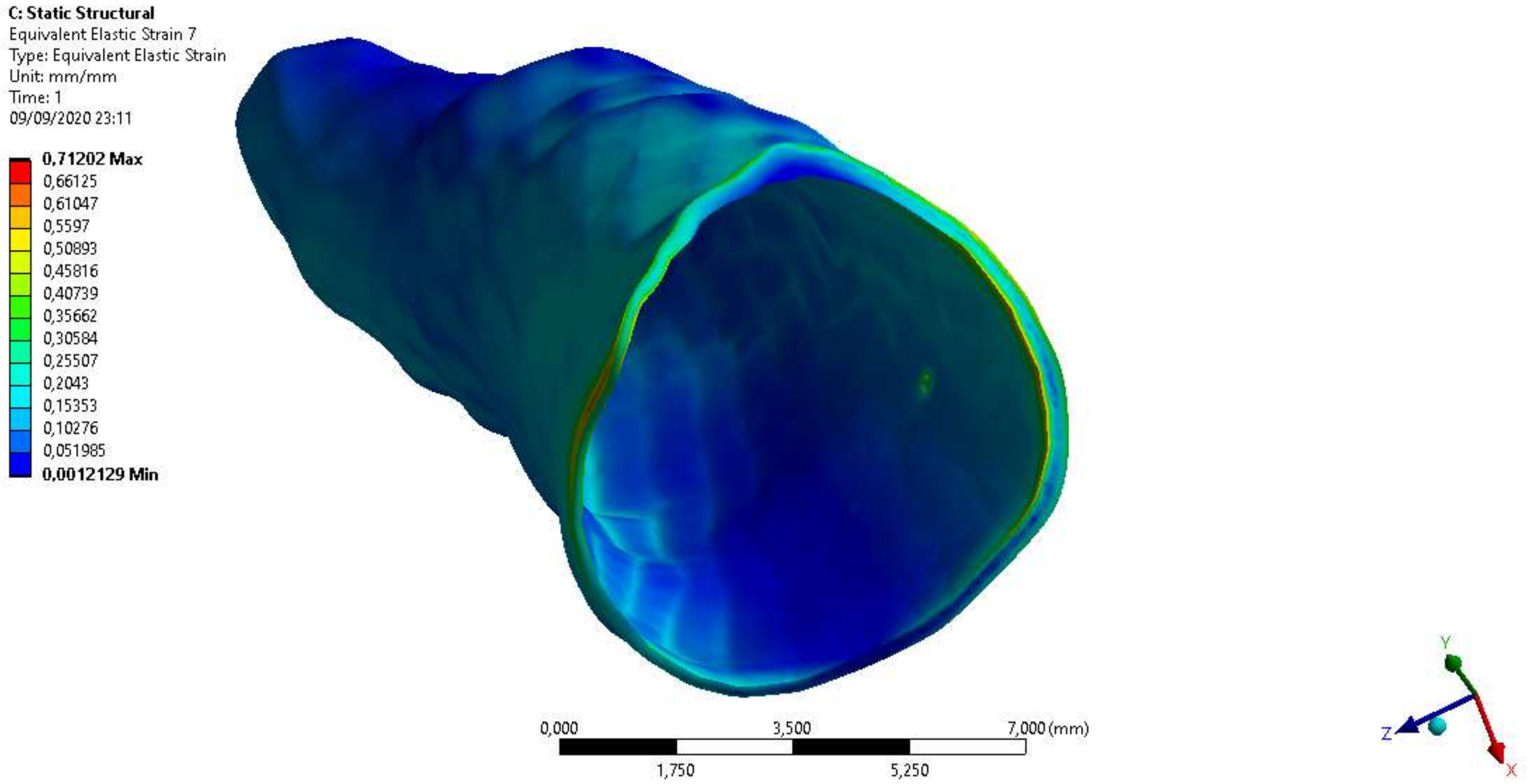
C: Static Structural
Equivalent Elastic Strain 7
Type: Equivalent Elastic Strain
Unit: mm/mm
Time: 1
09/09/2020 23:11
0,71202 Max
0,66125
0,61047
0,5597
0,50893
0,45816
0,40739
0,35662
0,30584
0,25507
0,2043
0,15353
0,10276
0,051985
0,0012129 Min
0,000
3,500
7,000 (mm)
1,750
5,250
Y
Z
X

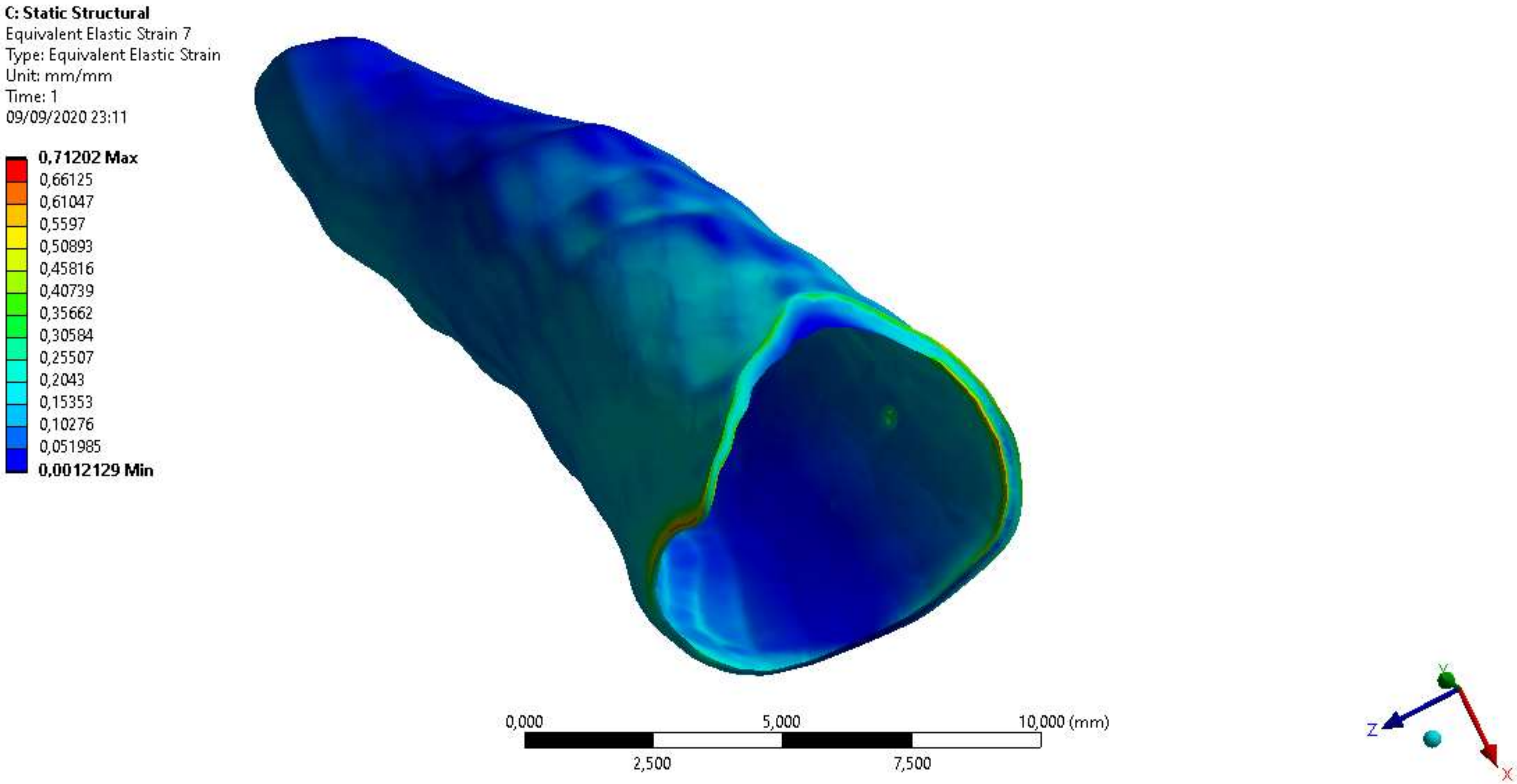
C: Static Structural
Equivalent Elastic Strain 7
Type: Equivalent Elastic Strain
Unit: mm/mm
Time: 1
09/09/2020 23:11
0,71202 Max
0,66125
0,61047
0,5597
0,50893
0,45816
0,40739
0,35662
0,30584
0,25507
0,2043
0,15353
0,10276
0,051985
0,0012129 Min
0,000
5,000
10,000 (mm)
2,500
7,500
Y
Z
X

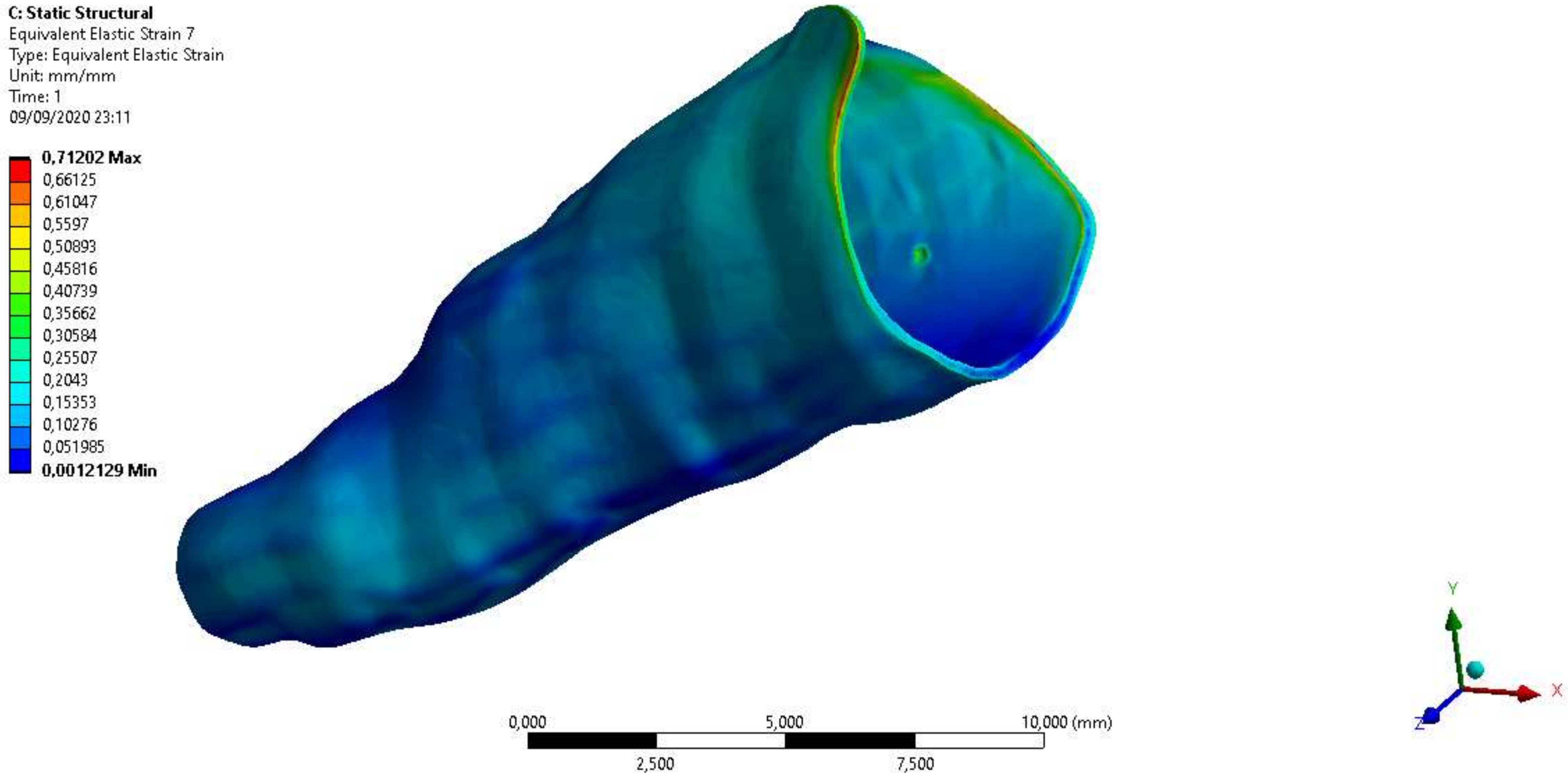

C: Static Structural
Equivalent Elastic Strain 7
Type: Equivalent Elastic Strain
Unit: mm/mm
Time: 1
09/09/2020 23:11
0,71202 Max
0,66125
0,61047
0,5597
0,50893
0,45816
0,40739
0,35662
0,30584
0,25507
0,2043
0,15353
0,10276
0,051985
0,0012129 Min
0,000
5,000
10,000 (mm)
2,500
7,500
Y
X
Z

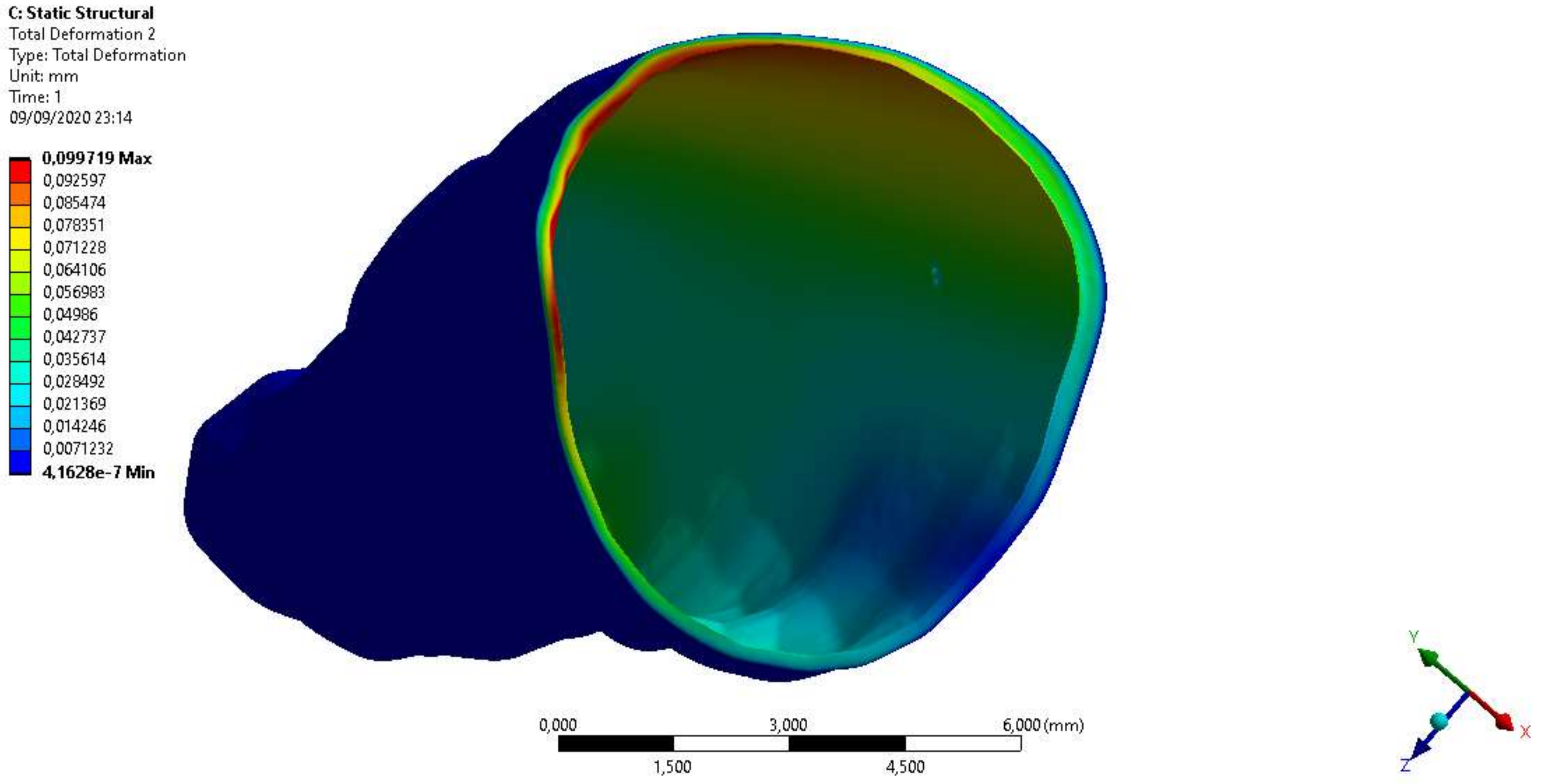
C: Static Structural
Total Deformation 2
Type: Total Deformation
Unit: mm
Time: 1
09/09/2020 23:14
0,099719 Max
0,092597
0,085474
0,078351
0,071228
0,064106
0,056983
0,04986
0,042737
0,035614
0,028492
0,021369
0,014246
0,0071232
4,1628e-7 Min
0,000
3,000
6,000 (mm)
1,500
4,500
Y
X
Z

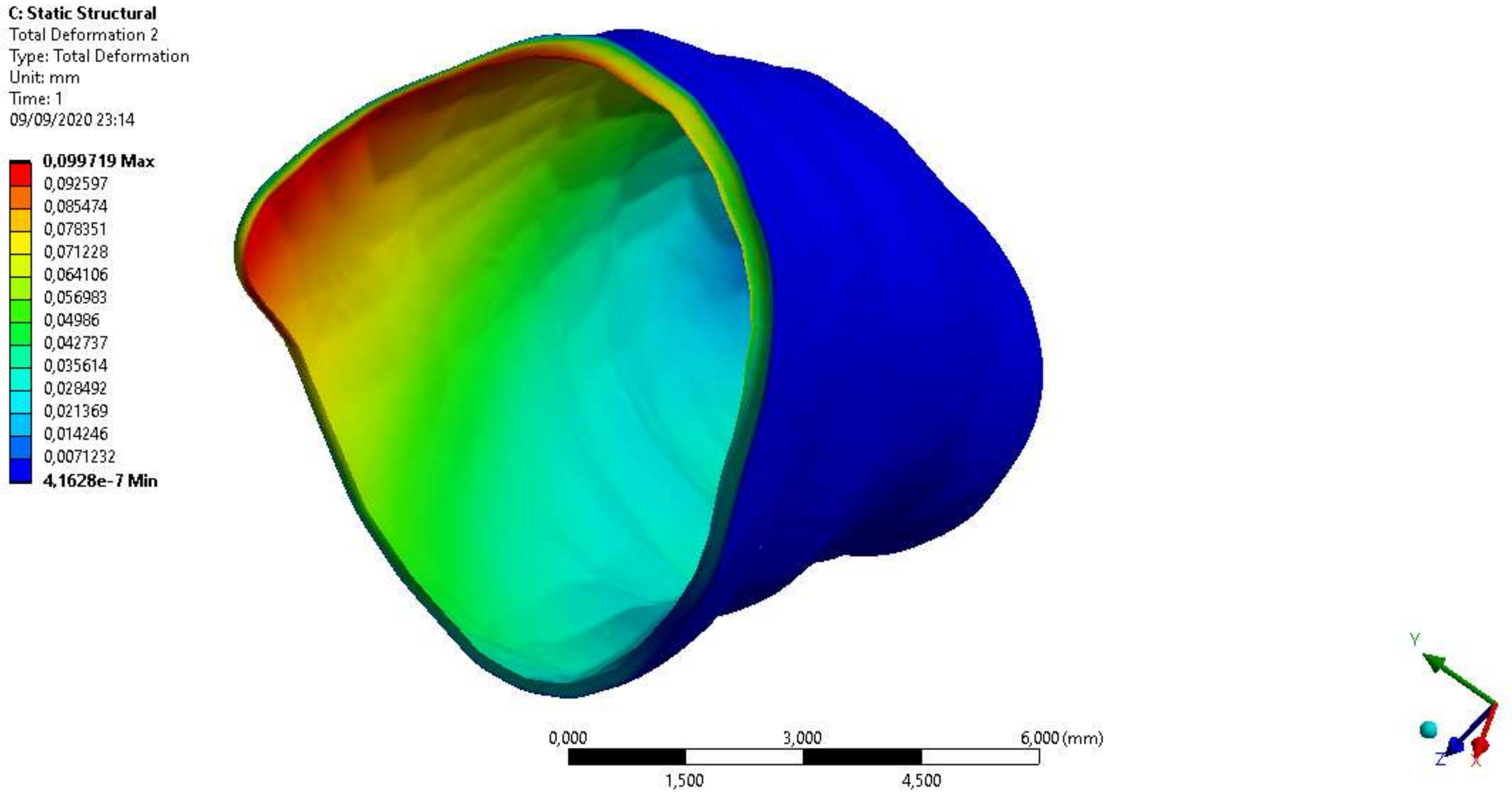
C: Static Structural
Total Deformation 2
Type: Total Deformation
Unit: mm
Time: 1
09/09/2020 23:14
0,099719 Max
0,092597
0,085474
0,078351
0,071228
0,064106
0,056983
0,04986
0,042737
0,035614
0,028492
0,021369
0,014246
0,0071232
4,1628e-7 Min
0,000
3,000
6,000 (mm)
1,500
4,500
Y
Z
X

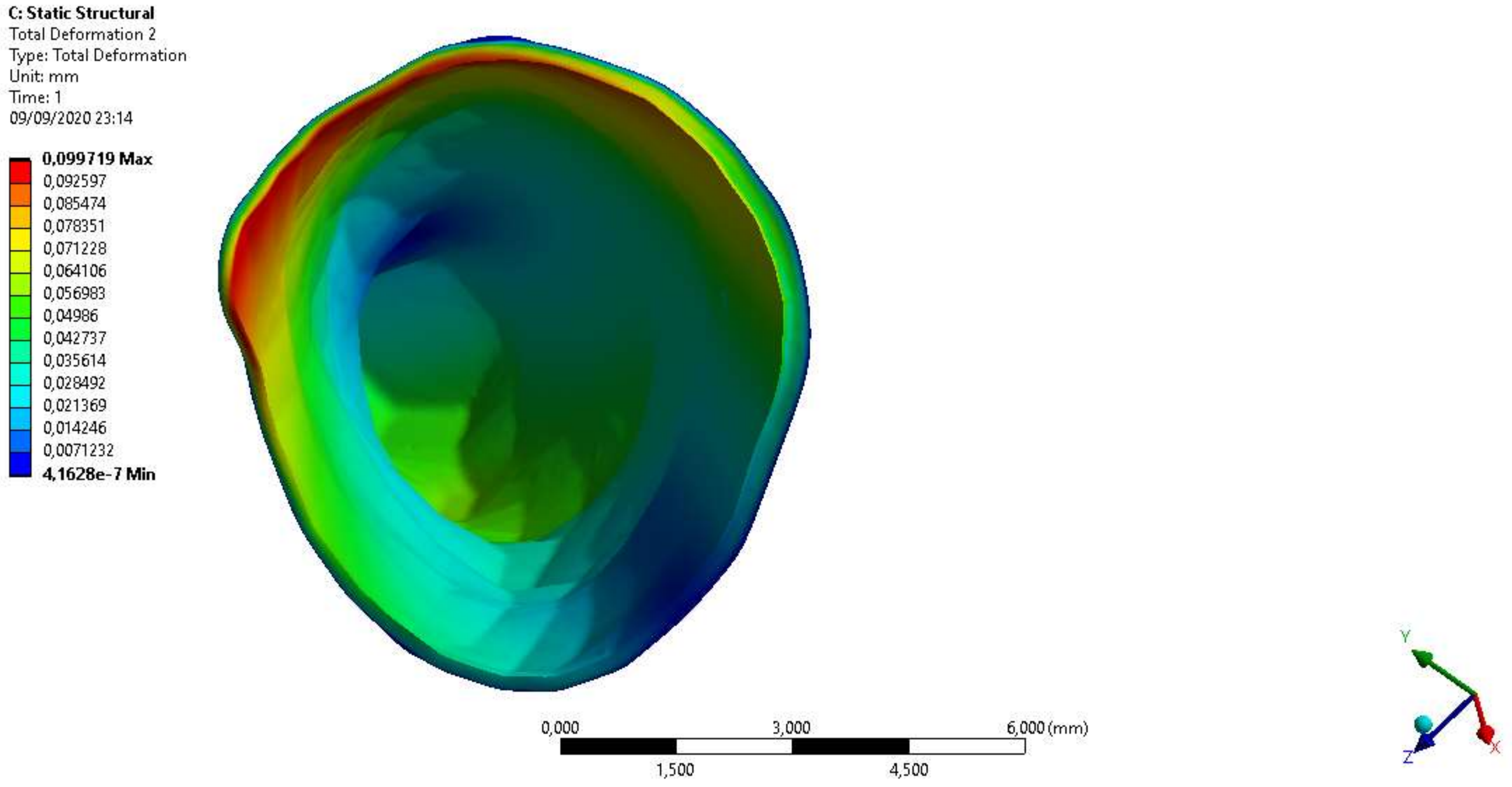

C: Static Structural
Total Deformation 2
Type: Total Deformation
Unit: mm
Time: 1
09/09/2020 23:14
0,099719 Max
0,092597
0,085474
0,078351
0,071228
0,064106
0,056983
0,04986
0,042737
0,035614
0,028492
0,021369
0,014246
0,0071232
4,1628e-7 Min
0,000
3,000
6,000 (mm)
1,500
4,500
Y
Z
X

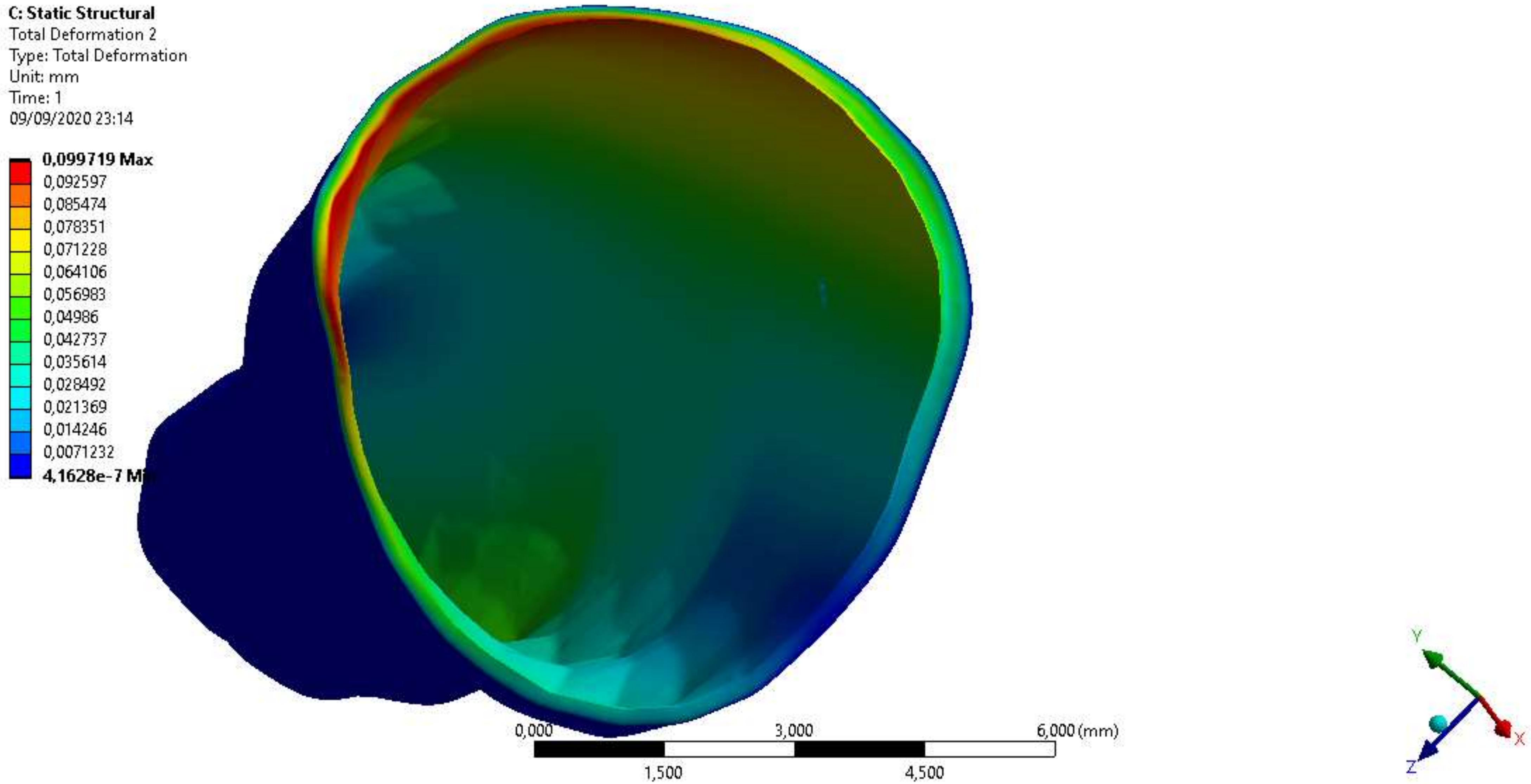
C: Static Structural
Total Deformation 2
Type: Total Deformation
Unit: mm
Time: 1
09/09/2020 23:14
0,099719 Max
0,092597
0,085474
0,078351
0,071228
0,064106
0,056983
0,04986
0,042737
0,035614
0,028492
0,021369
0,014246
0,0071232
4,1628e-7 Mi
0,000
3,000
6,000 (mm)
1,500
4,500
Y
X
Z

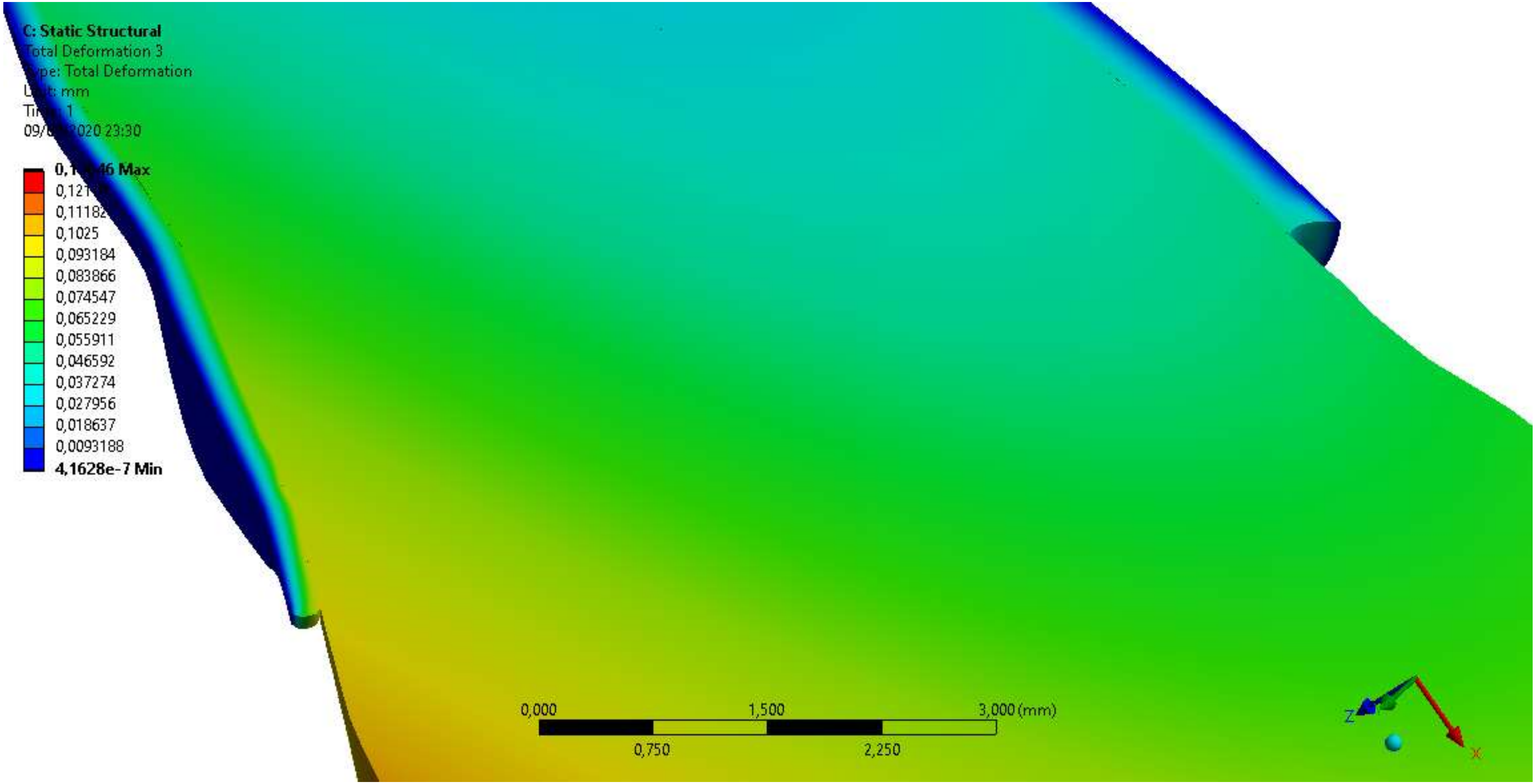
C: Static Structural
Total Deformation 3
Type: Total Deformation
Unit: mm
0,111182
0,1025
0,093184
0,083866
0,074547
0,065229
0,055911
0,046592
0,037274
0,027956
0,018637
0,0093188
4,1628e-7 Min
0,000
1,500
3,000 (mm)
0,750
2,250
Z
X

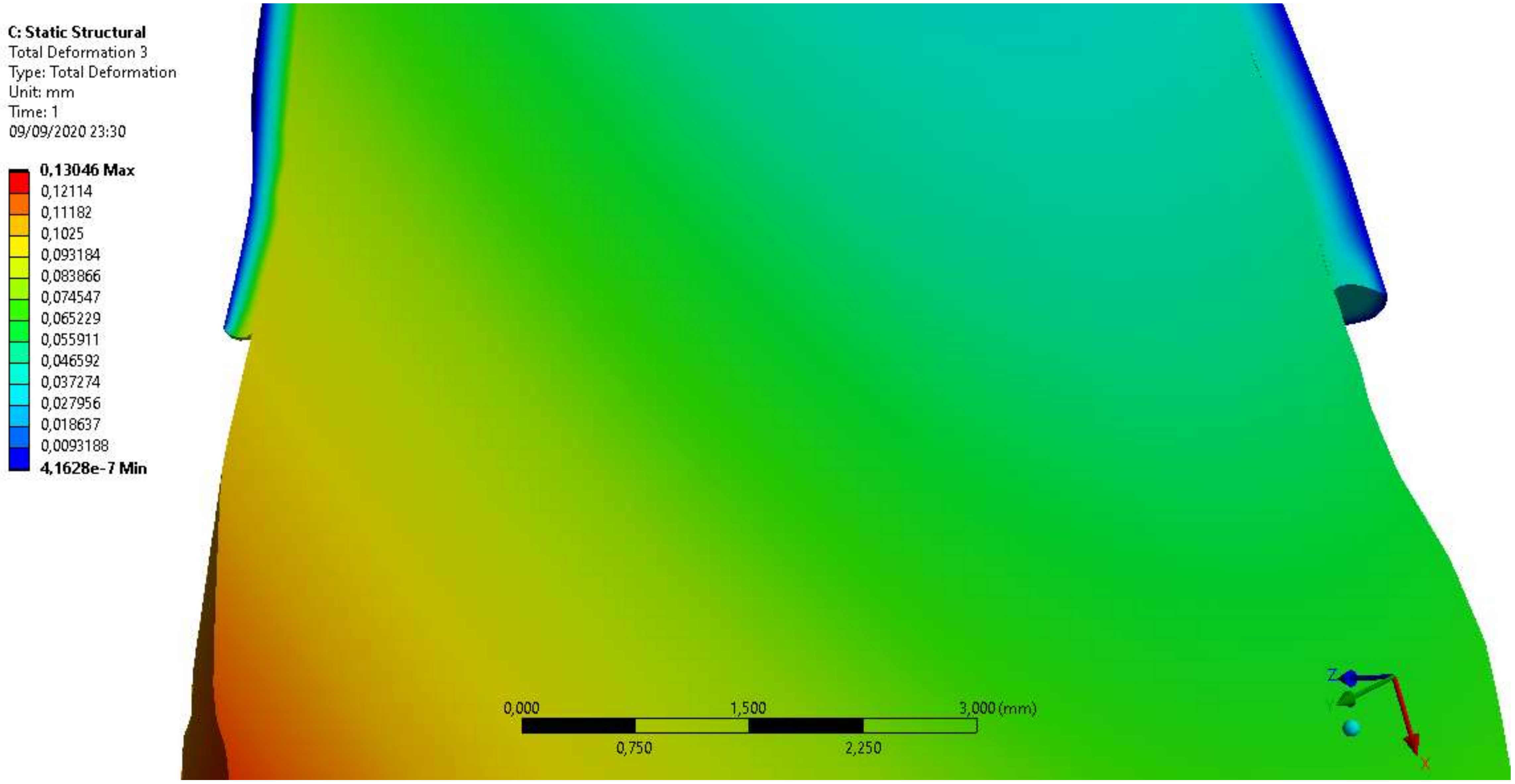
C: Static Structural
Total Deformation 3
Type: Total Deformation
Unit: mm
Time: 1
09/09/2020 23:30
0,13046 Max
0,12114
0,11182
0,1025
0,093184
0,083866
0,074547
0,065229
0,055911
0,046592
0,037274
0,027956
0,018637
0,0093188
4,1628e-7 Min
0,000
1,500
3,000 (mm)
0,750
2,250

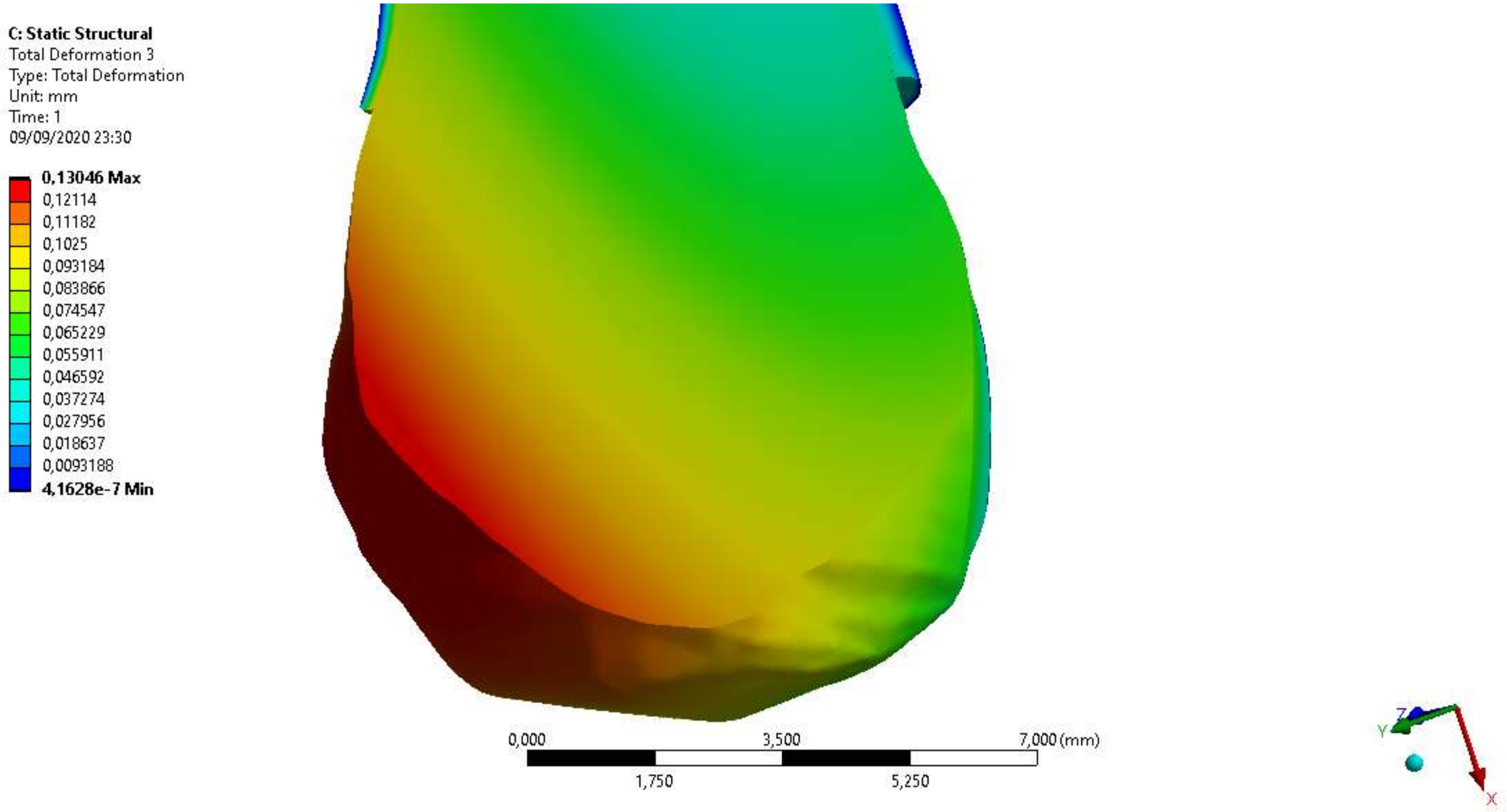
C: Static Structural
Total Deformation 3
Type: Total Deformation
Unit: mm
Time: 1
09/09/2020 23:30
0,13046 Max
0,12114
0,11182
0,1025
0,093184
0,083866
0,074547
0,065229
0,055911
0,046592
0,037274
0,027956
0,018637
0,0093188
4,1628e-7 Min
0,000
3,500
7,000 (mm)
1,750
5,250
Z
Y
X
