# Supplementary material for: Micro-osteoperforation for enhancement of orthodontic movement: A mechanical analysis using the finite element method
Source: PLoS One. 2024 Aug 19;19(8):e0308739. doi: 10.1371/journal.pone.0308739 (PMC11332926; doi:10.1371/journal.pone.0308739)
Supplement: S4 File — (PDF) [file pone.0308739.s004.pdf]

S4. Analysis 2 with perforations

Dente

Fatigue Data at zero mean stress comes from 1998 ASME BPV Code, Section 8, Div 2, Table 5-110.1

Density	1,96e-06 kg/mm³

Structural

Isotropic Elasticity

Derive from	Young's Modulus and Poisson's Ratio
Young's Modulus	14700 MPa
Poisson's Ratio	0,31000
Bulk Modulus	12895 MPa
Shear Modulus	5610,7 MPa
Isotropic Secant Coefficient of Thermal Expansion	1,2e-05 1/°C
Compressive Ultimate Strength	0 MPa
Compressive Yield Strength	250,00 MPa

Osso Medular

Density	4,1e-07 kg/mm³

Structural

Isotropic Elasticity

Derive from	Young's Modulus and Poisson's Ratio
Young's Modulus	1370,0 MPa
Poisson's Ratio	0,30000
Bulk Modulus	1141,7 MPa
Shear Modulus	526,92 MPa

Osso Cortical Isotropico

Density	1,99e-06 kg/mm³

Structural

Isotropic Elasticity

Derive from	Young's Modulus and Poisson's Ratio
Young's Modulus	13700 MPa
Poisson's Ratio	0,30000
Bulk Modulus	11417 MPa
Shear Modulus	5269,2 MPa

LigamentoPeriodotal

Fatigue Data at zero mean stress comes from 1998 ASME BPV Code, Section 8, Div 2, Table 5-110.1

Density	1,2e-06 kg/mm³

Structural

Isotropic Elasticity

Derive from	Young's Modulus and Poisson's Ratio
Young's Modulus	0,068000 MPa
Poisson's Ratio	0,45000
Bulk Modulus	0,22667 MPa
Shear Modulus	0,023448 MPa

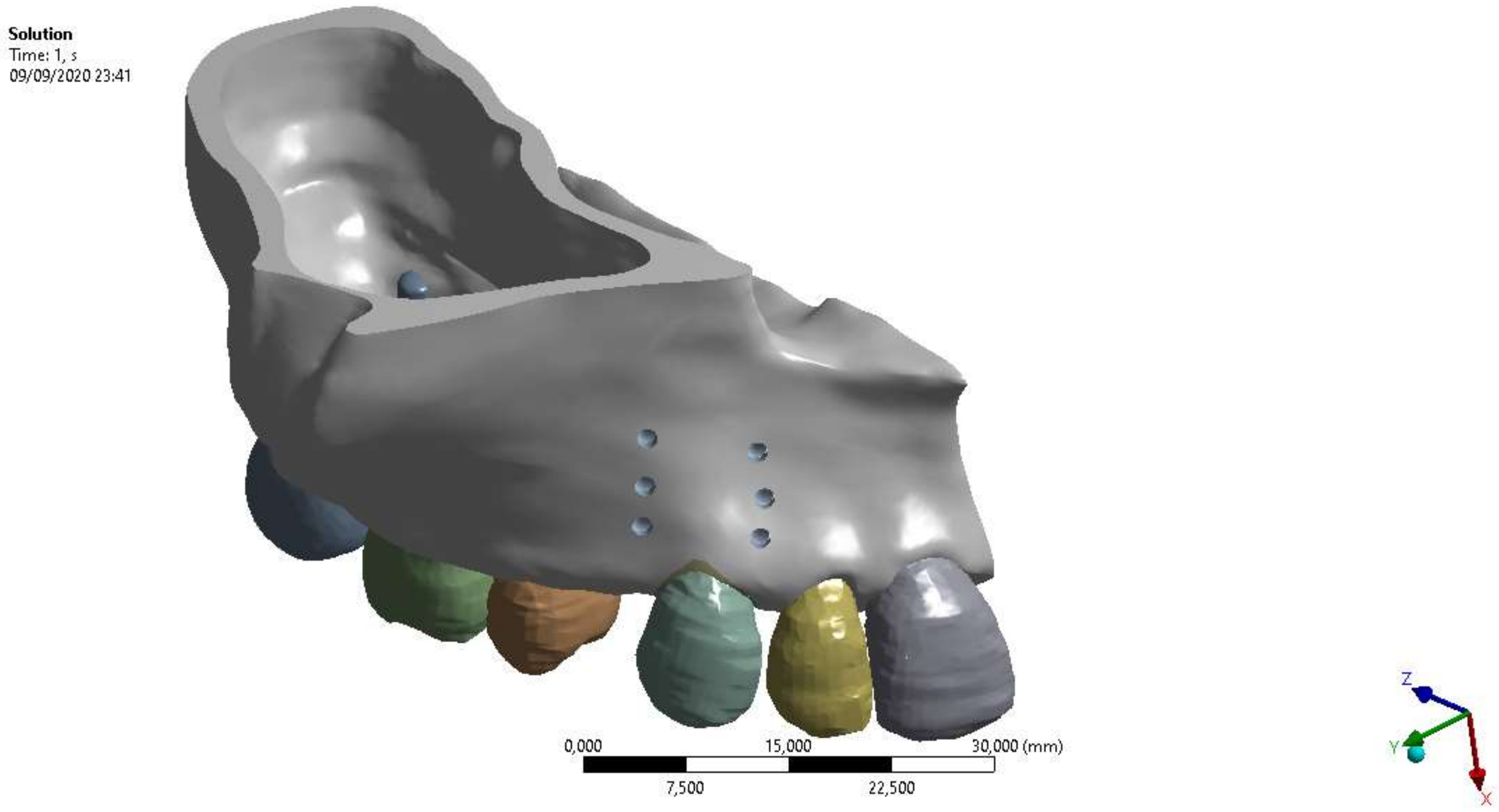
Solution
Time: 1, s
09/09/2020 23:41
0,000
15,000
30,000 (mm)
7,500
22,500
Z
Y
X

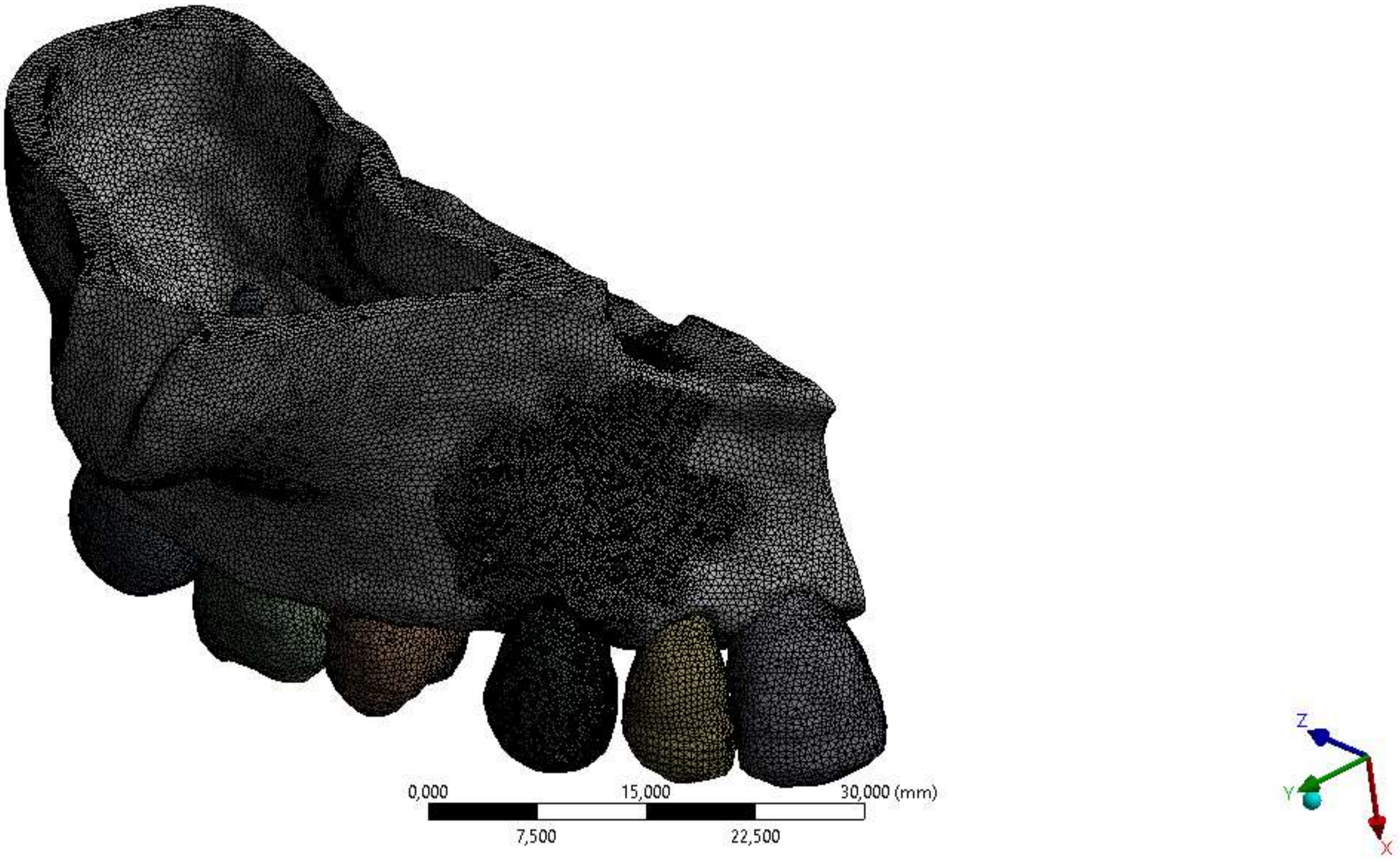

0,000
15,000
30,000 (mm)
7,500
22,500
Z
Y
X

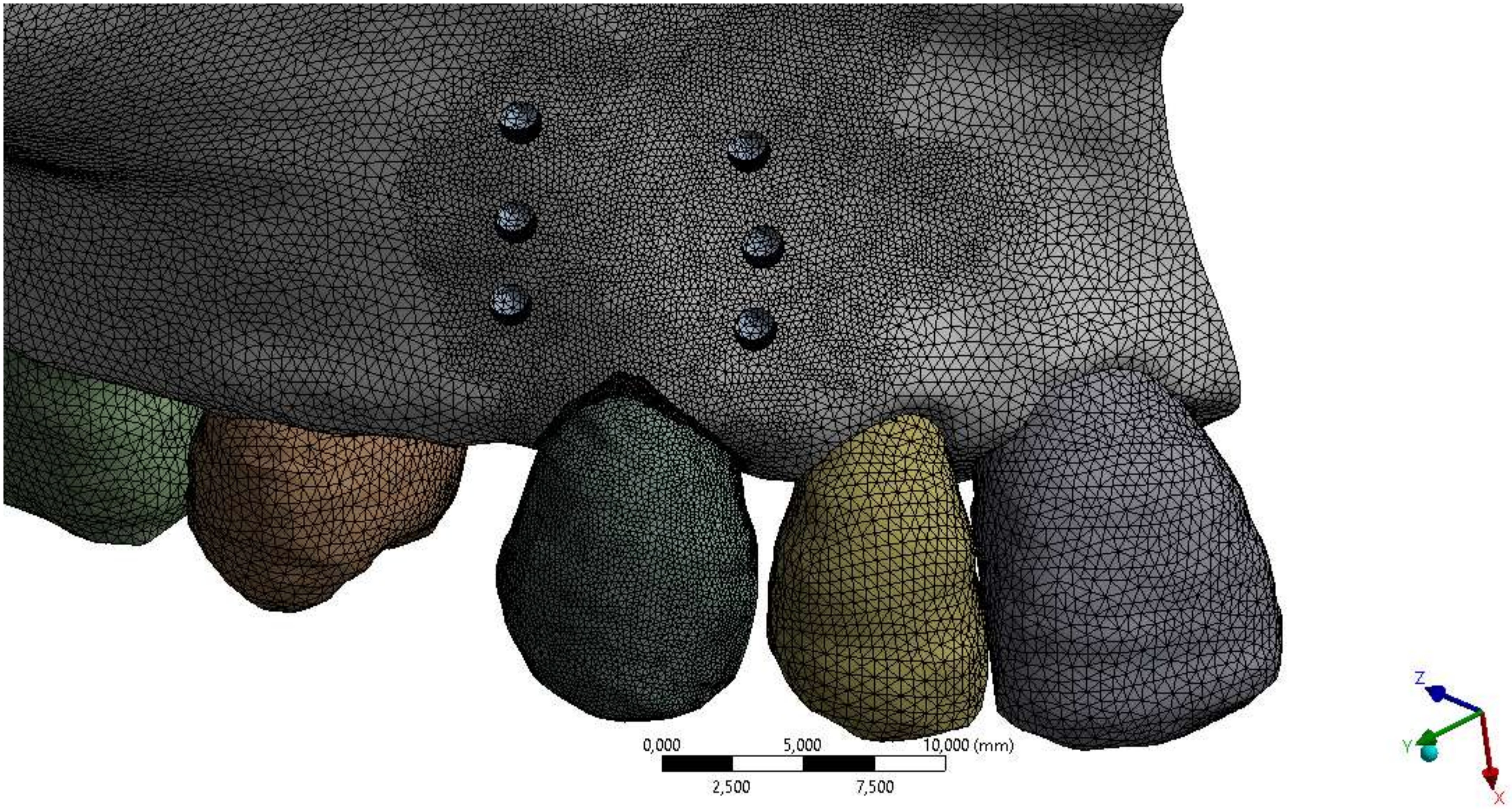

0,000
5,000
10,000 (mm)
2,500
7,500
Z
Y
X

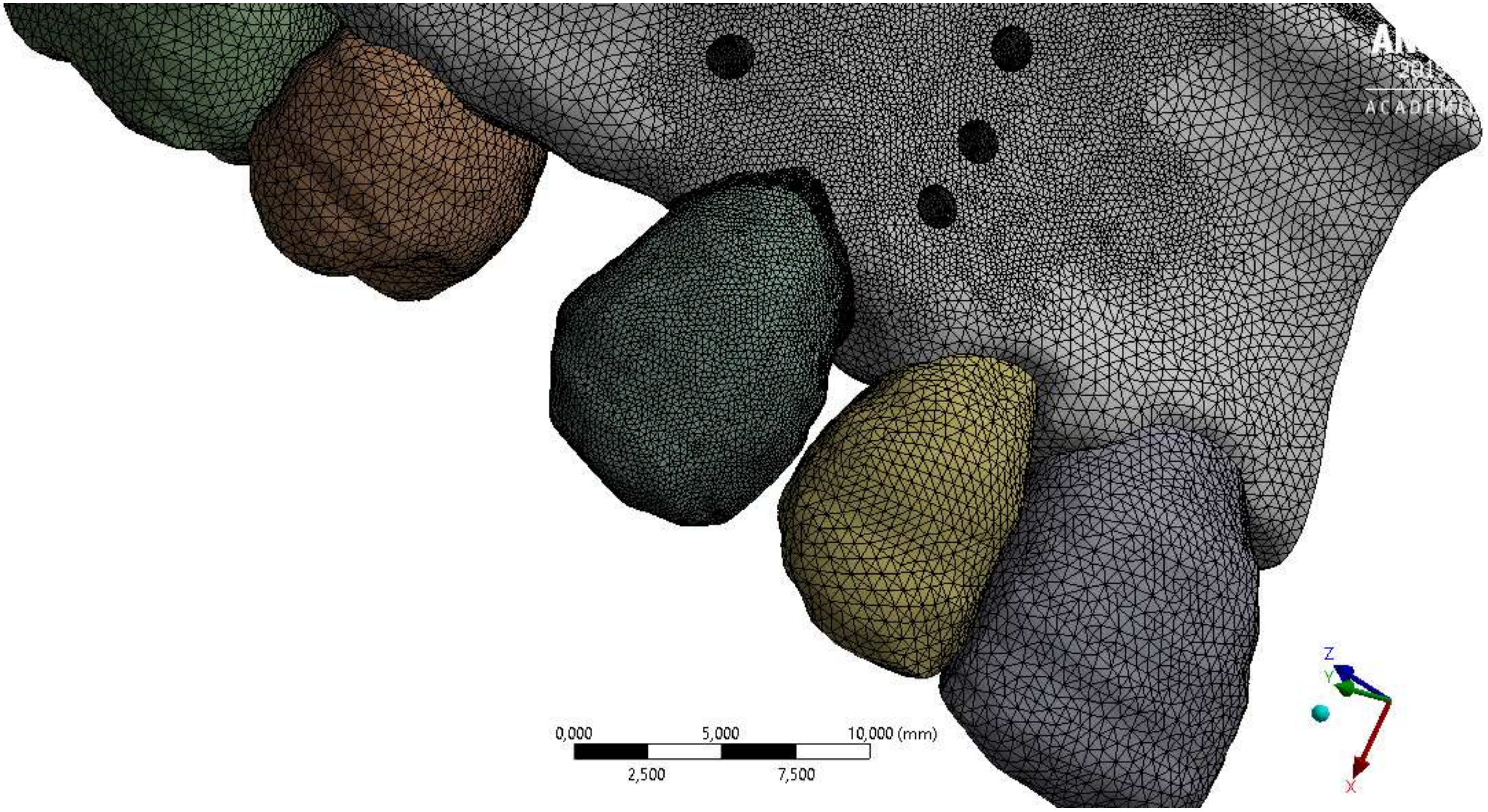
0,000
5,000
10,000 (mm)
2,500
7,500
Z
Y
X

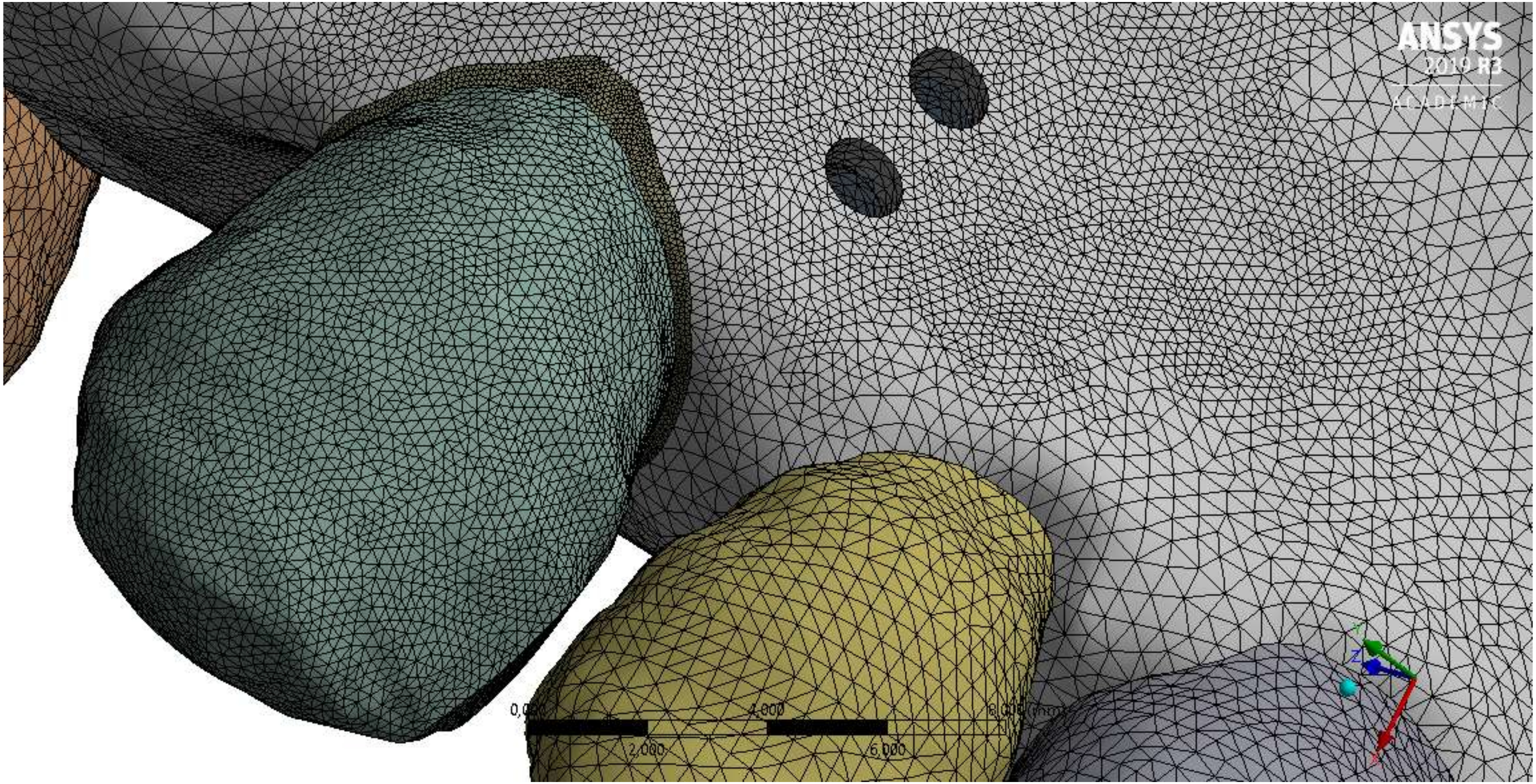
ANSYS
2019 R3
ACADEMIC
4,000
2,000
6,000

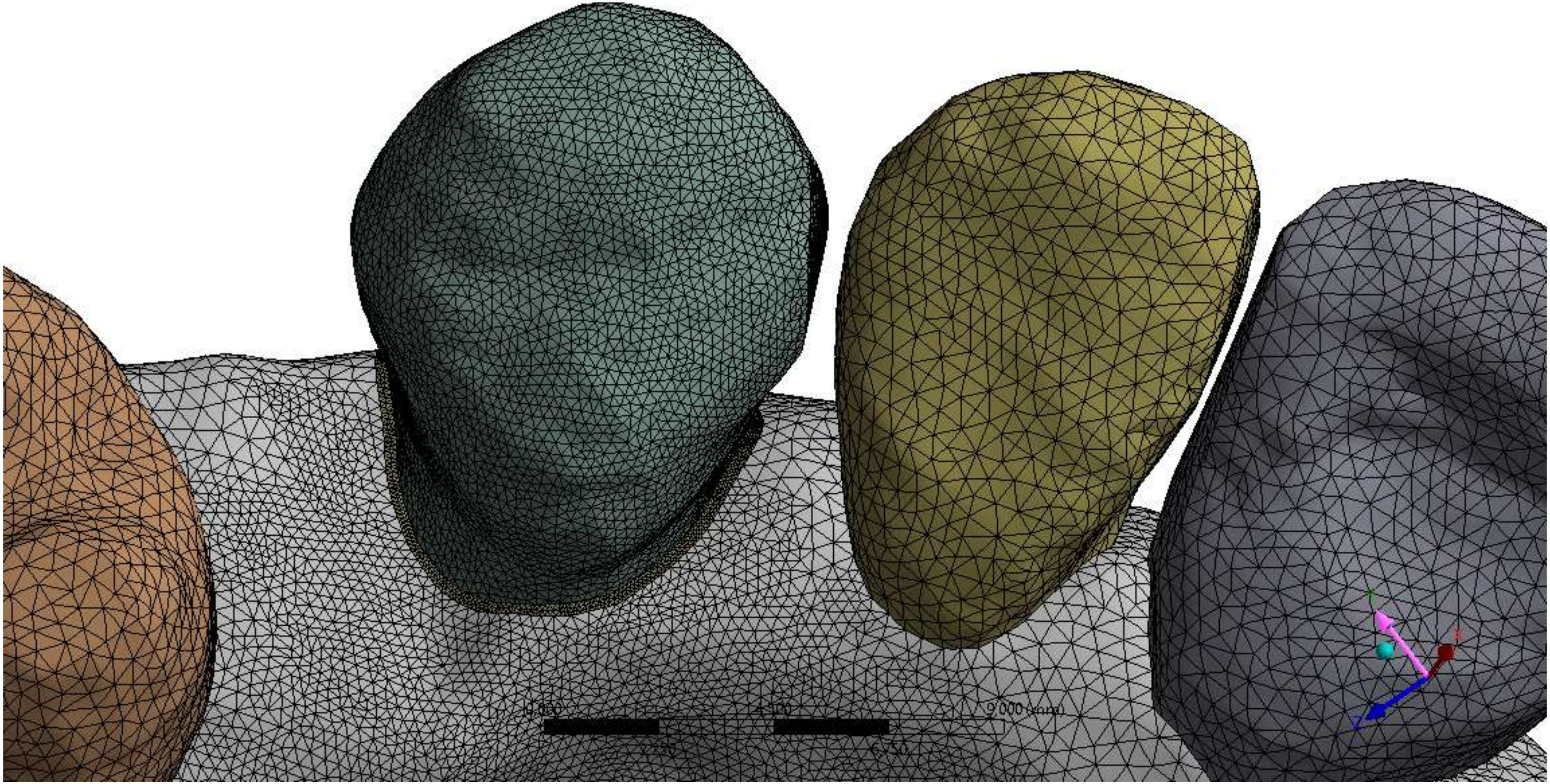
9.000 (mm)

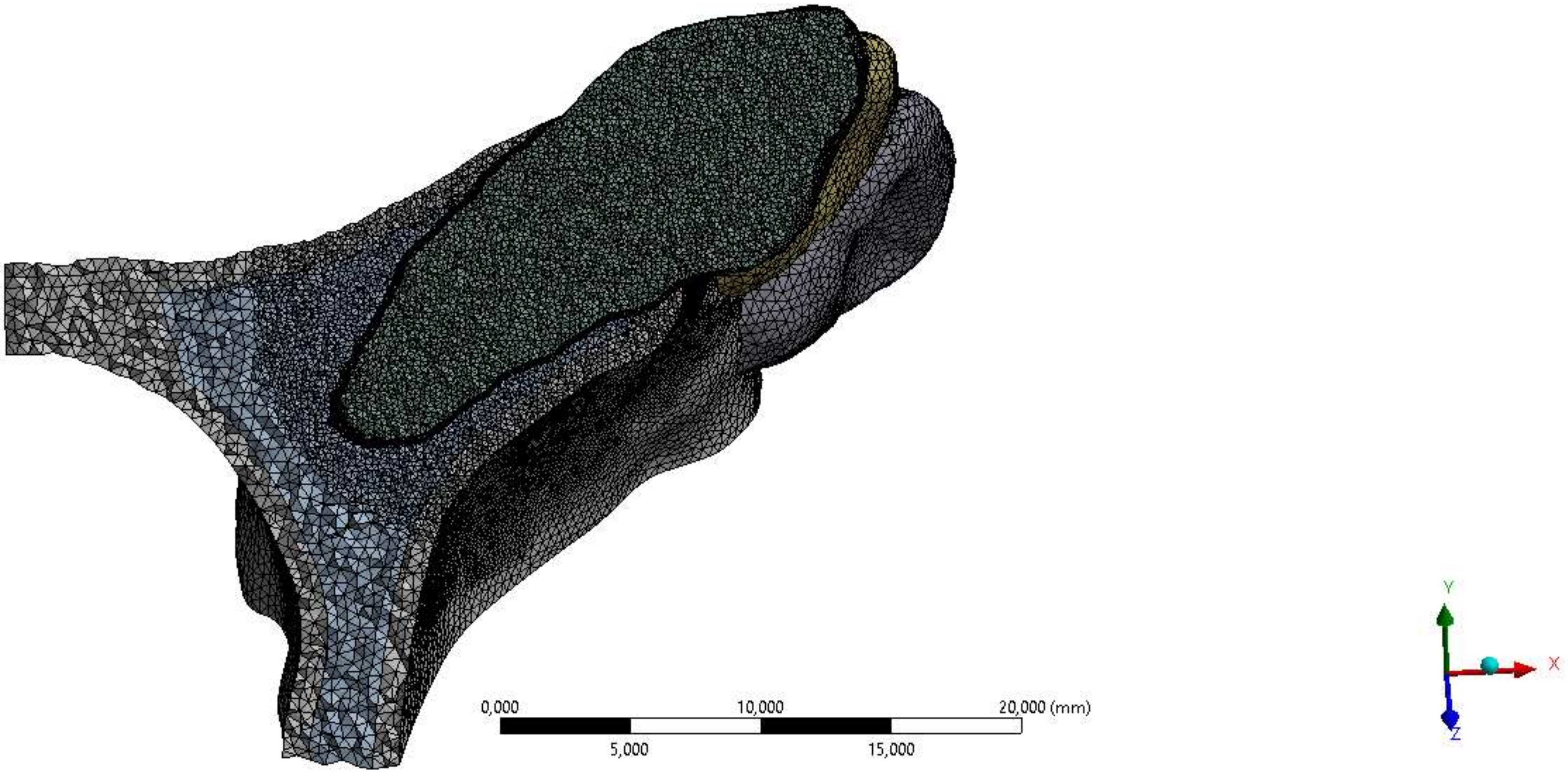
0,000
10,000
20,000 (mm)
5,000
15,000
Y
X
Z

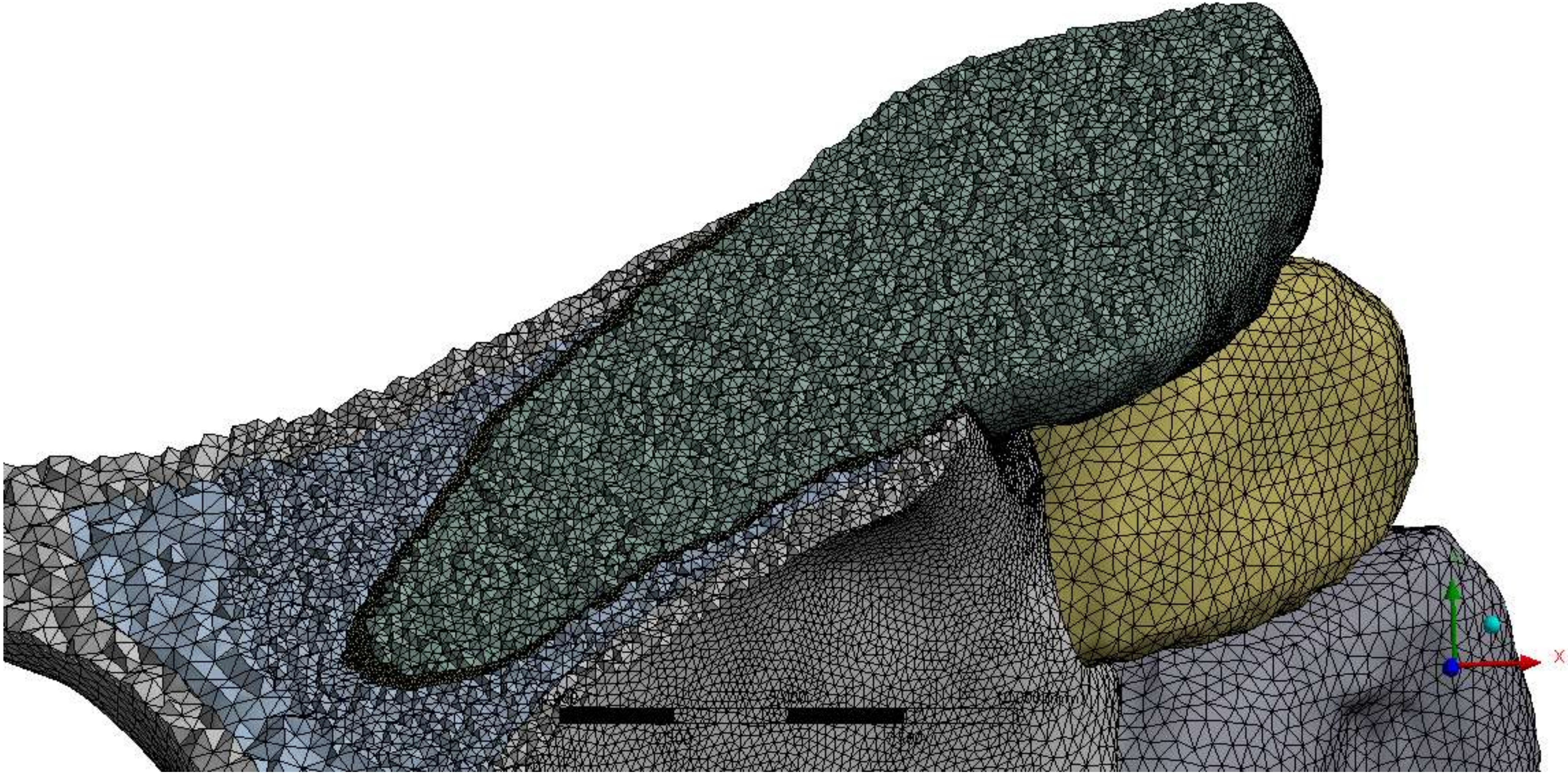
X

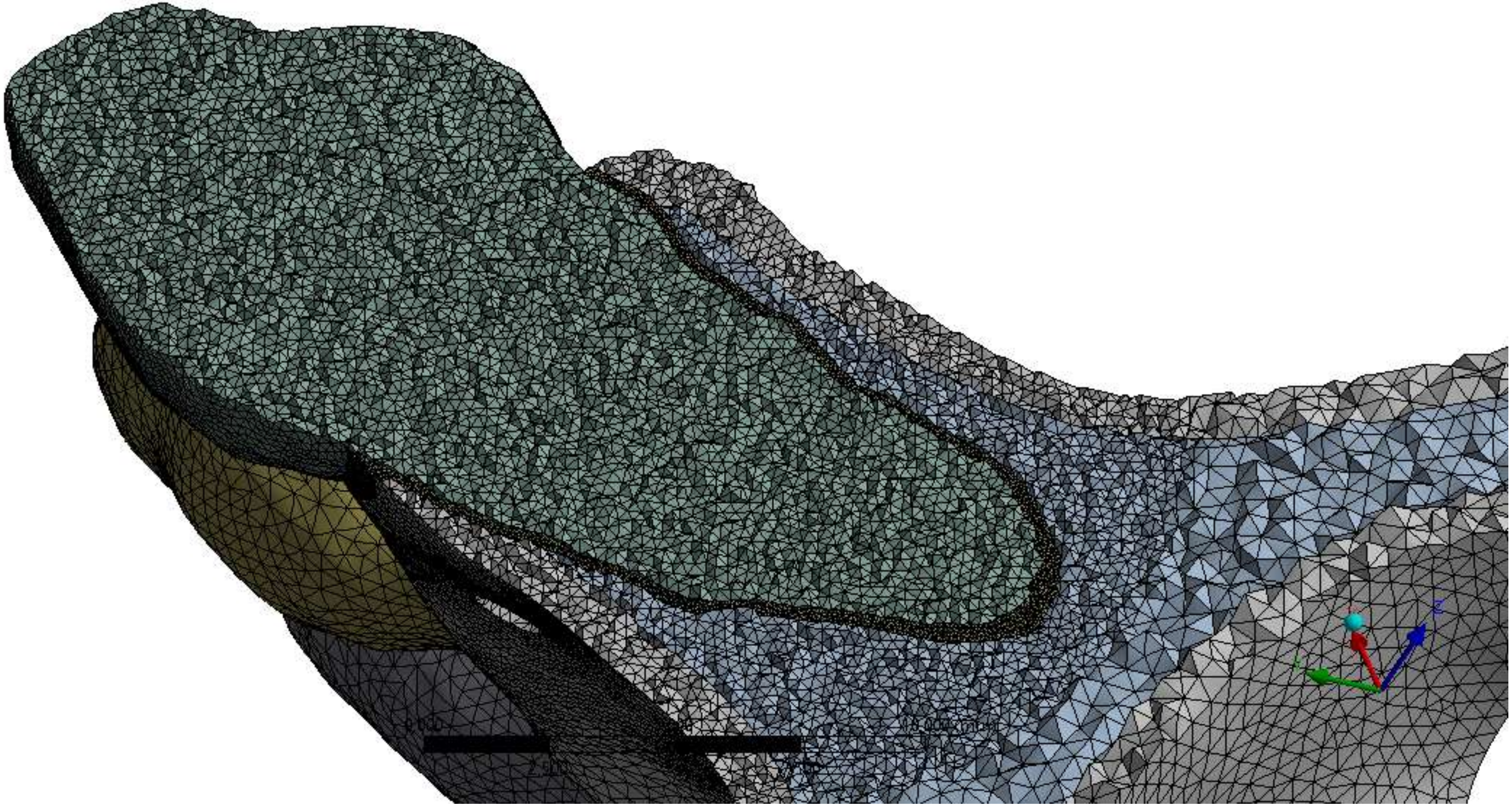

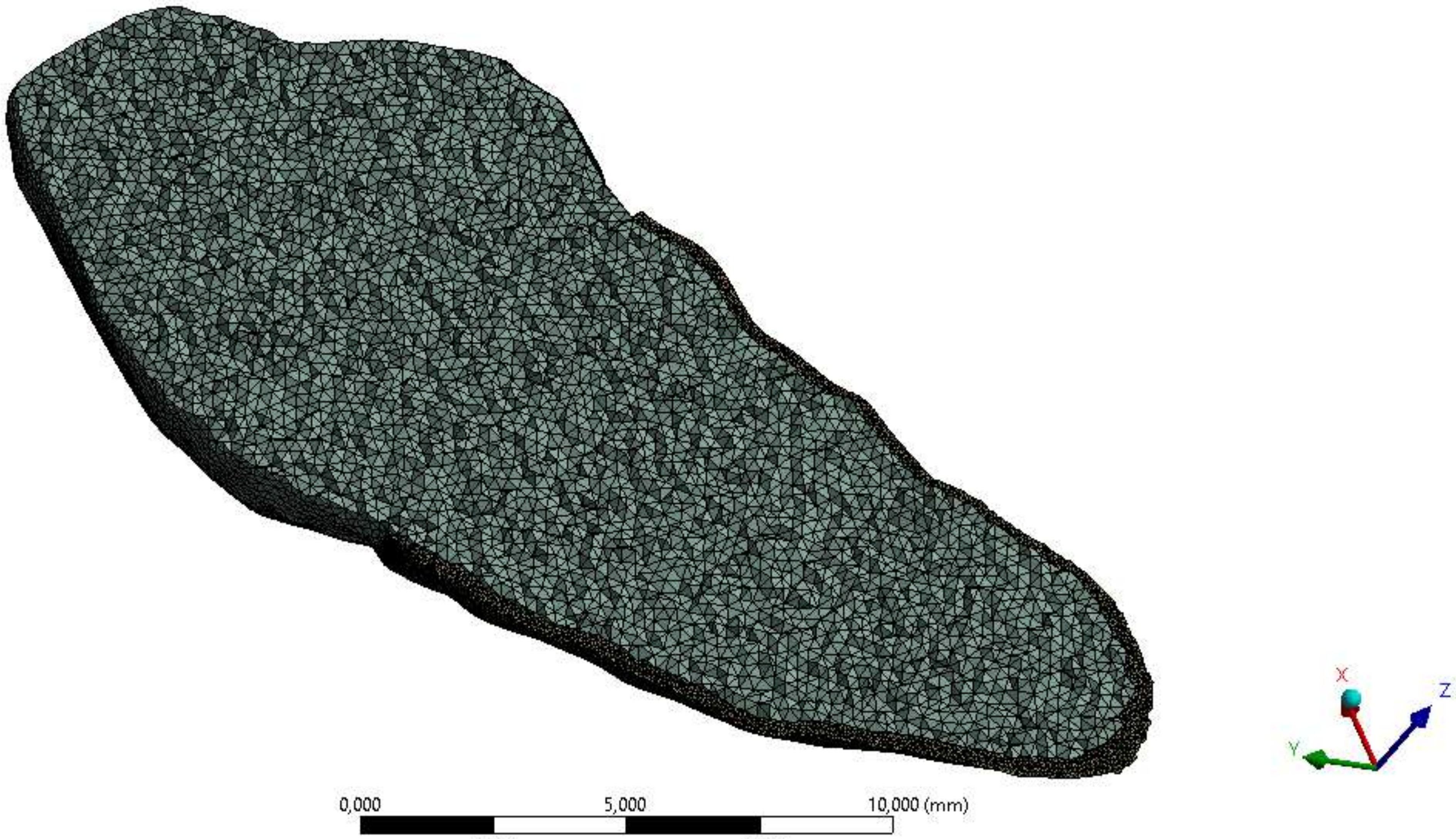
X
Z
Y
0,000
5,000
10,000 (mm)
2,500
7,500

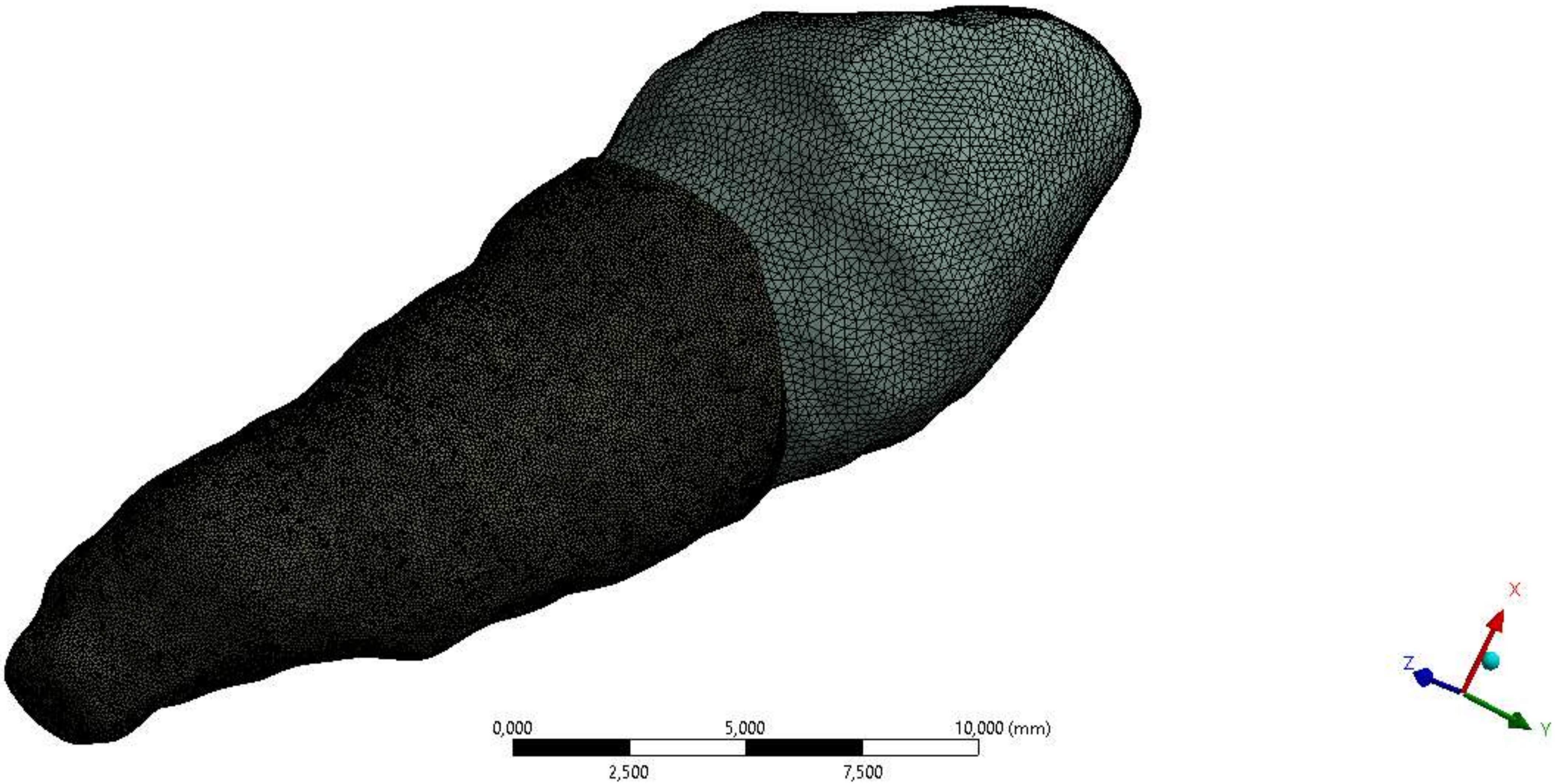
0,000
5,000
10,000 (mm)
2,500
7,500
X
Y
Z

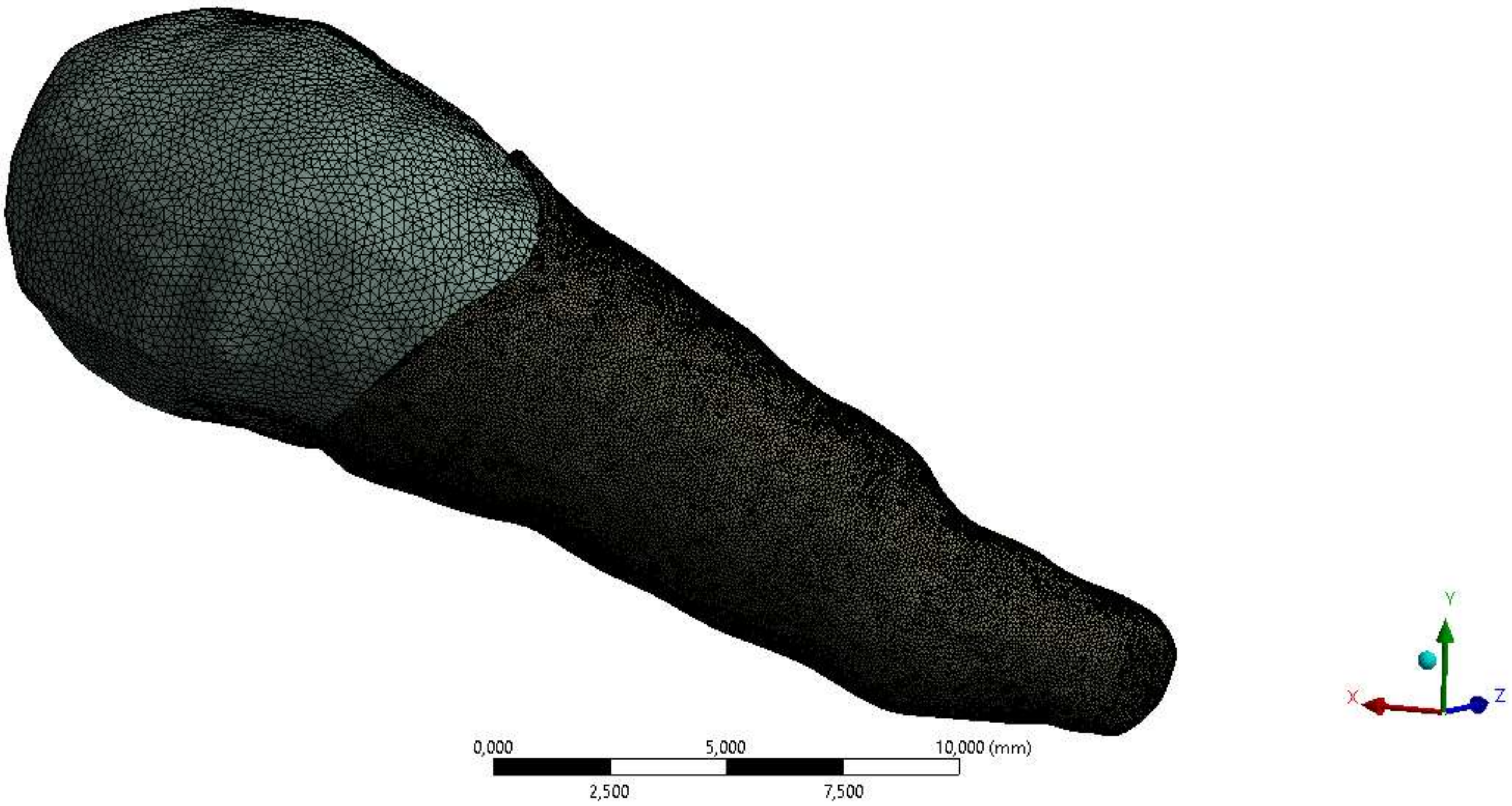

0,000
5,000
10,000 (mm)
2,500
7,500
Y
X
Z

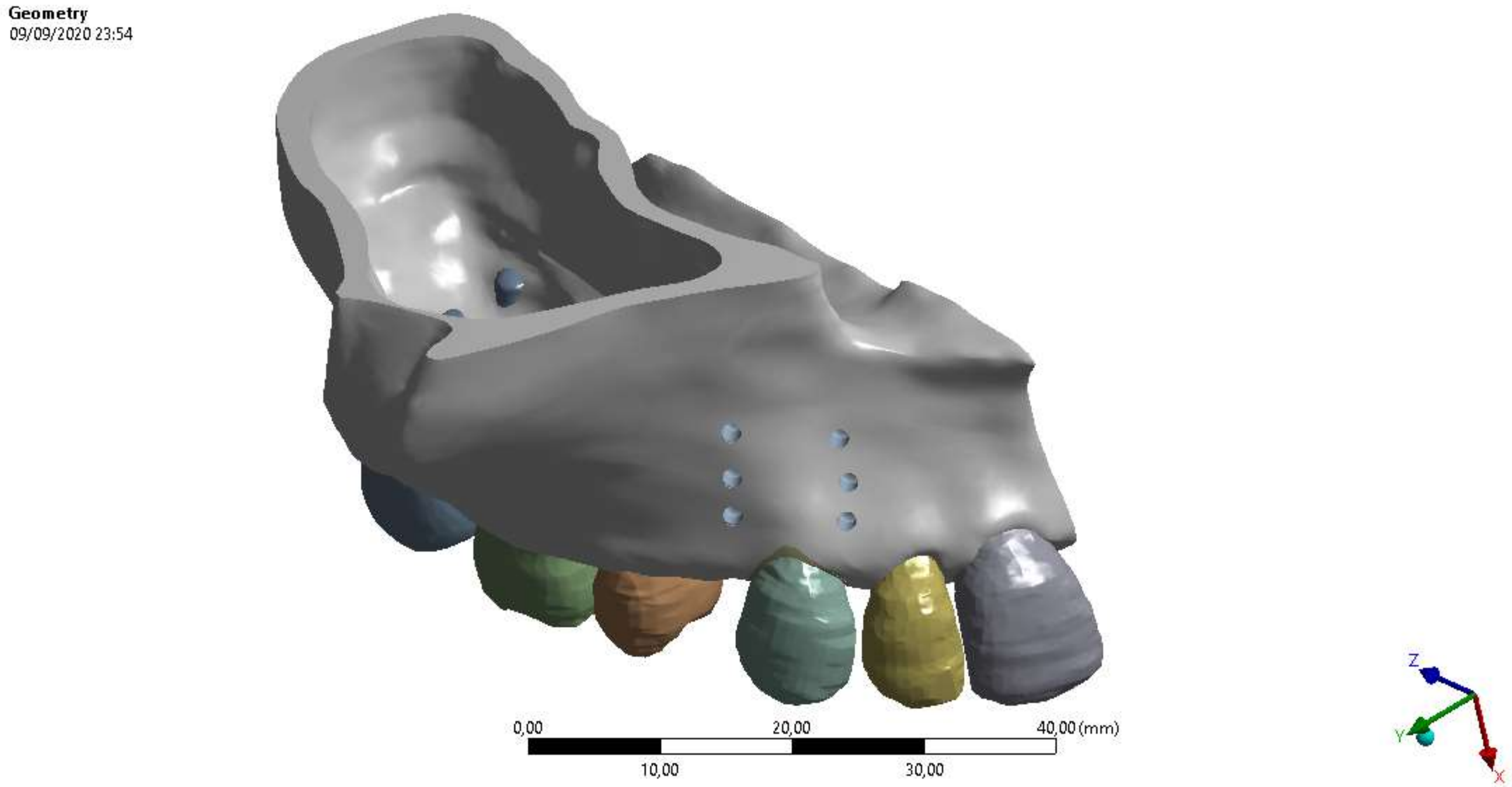
Geometry
09/09/2020 23:54
0,00
20,00
40,00 (mm)
10,00
30,00
Z
Y
X

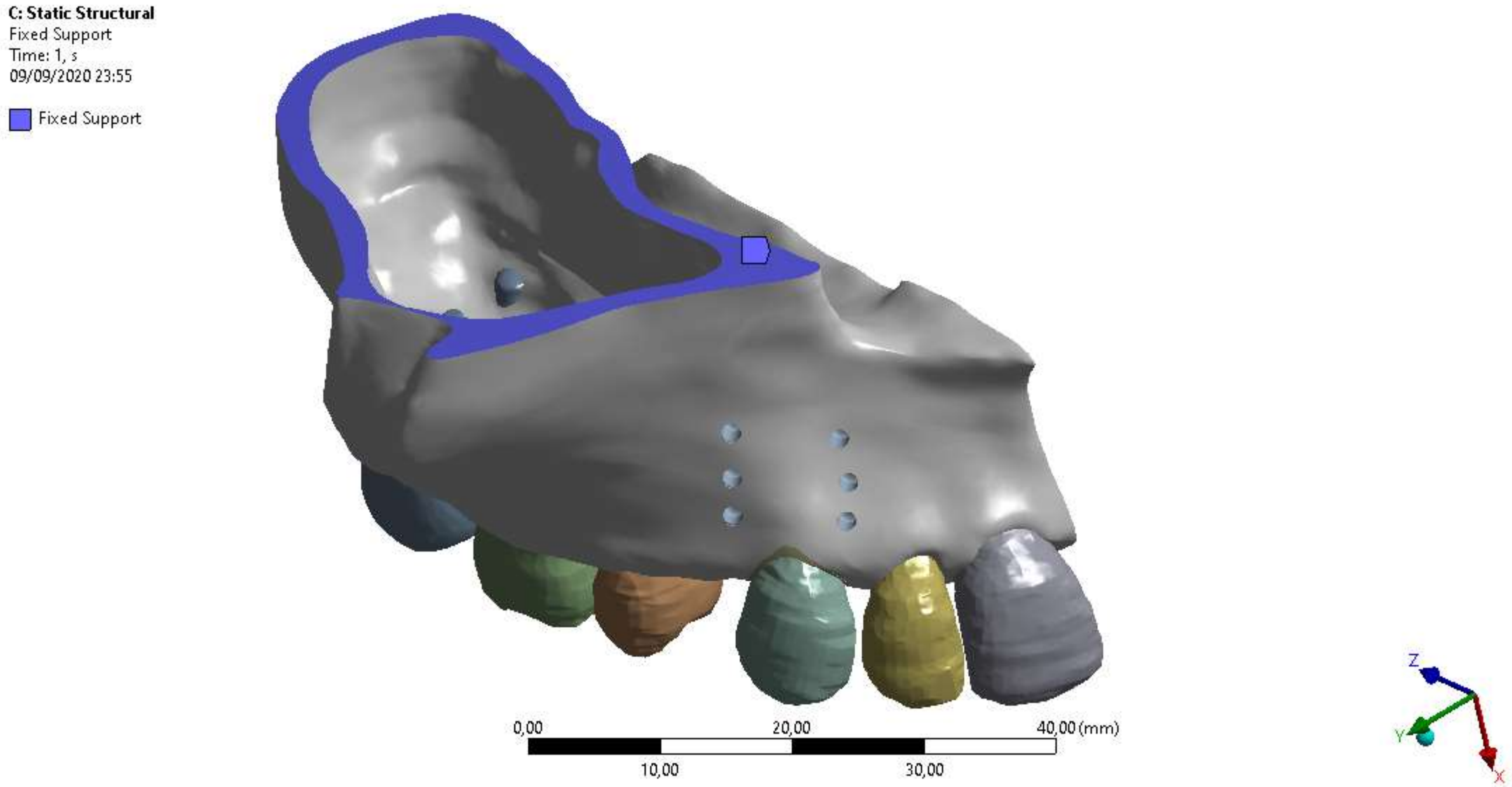
C: Static Structural
Fixed Support
Time: 1, s
09/09/2020 23:55
Fixed Support
0,00
20,00
40,00 (mm)
10,00
30,00
Z
Y
X

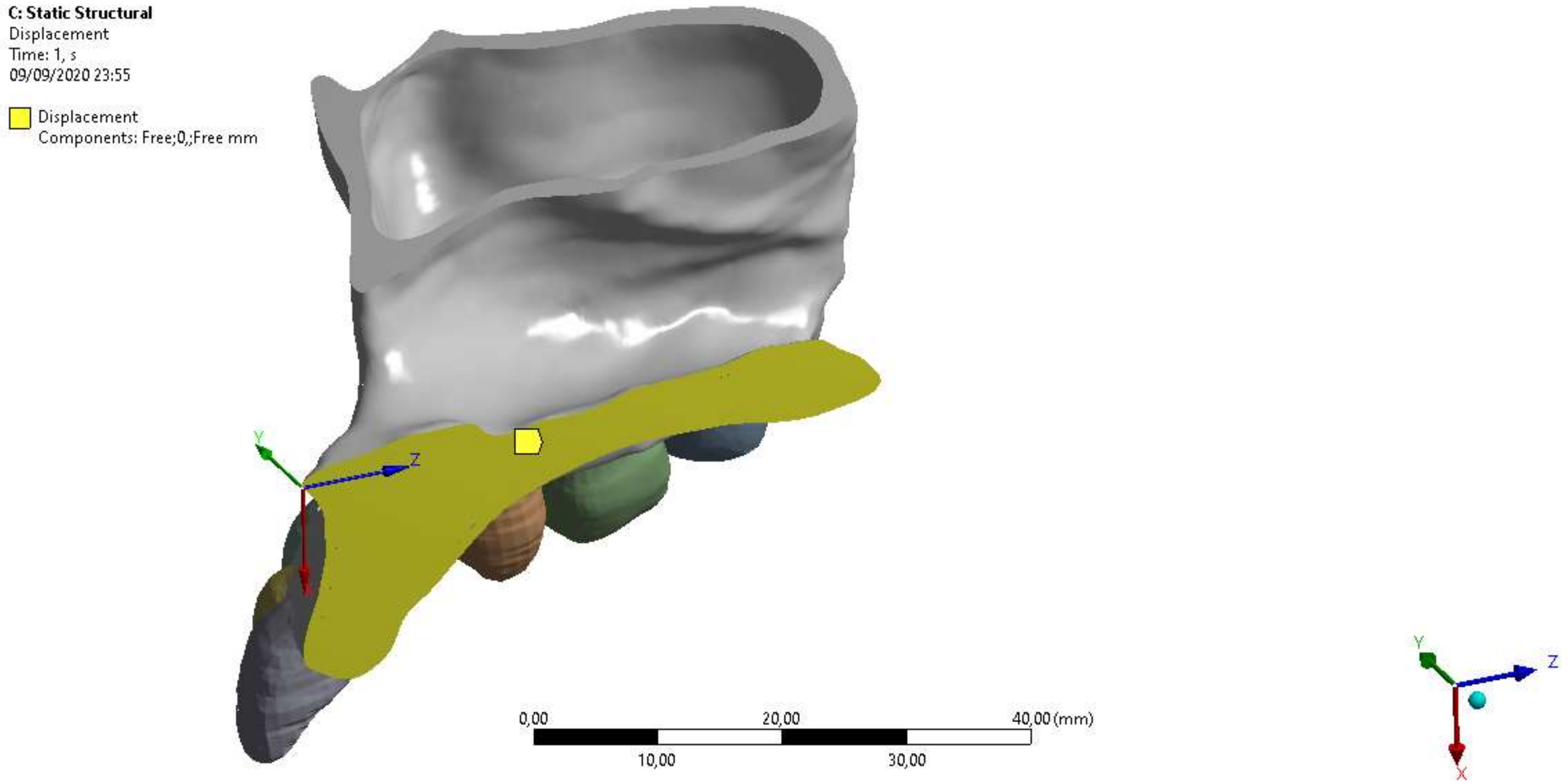
C: Static Structural
Displacement
Time: 1, s
09/09/2020 23:55
Displacement
Components: Free;0,;Free mm
0,00
20,00
40,00 (mm)
10,00
30,00
Y
Z
X

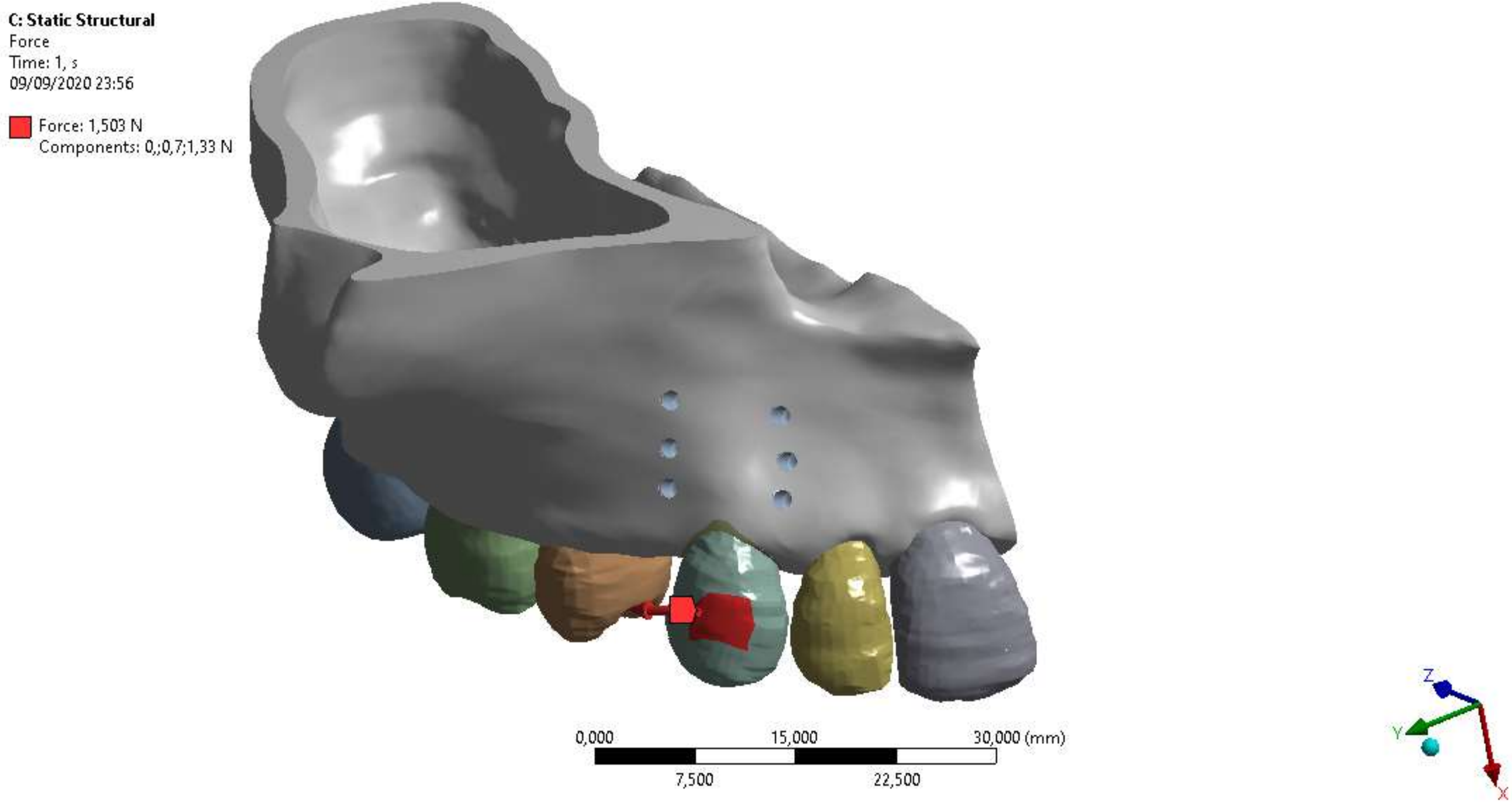
C: Static Structural
Force
Time: 1, s
09/09/2020 23:56
Force: 1,503 N
Components: 0,;0,7;1,33 N
0,000
15,000
30,000 (mm)
7,500
22,500
Z
Y
X

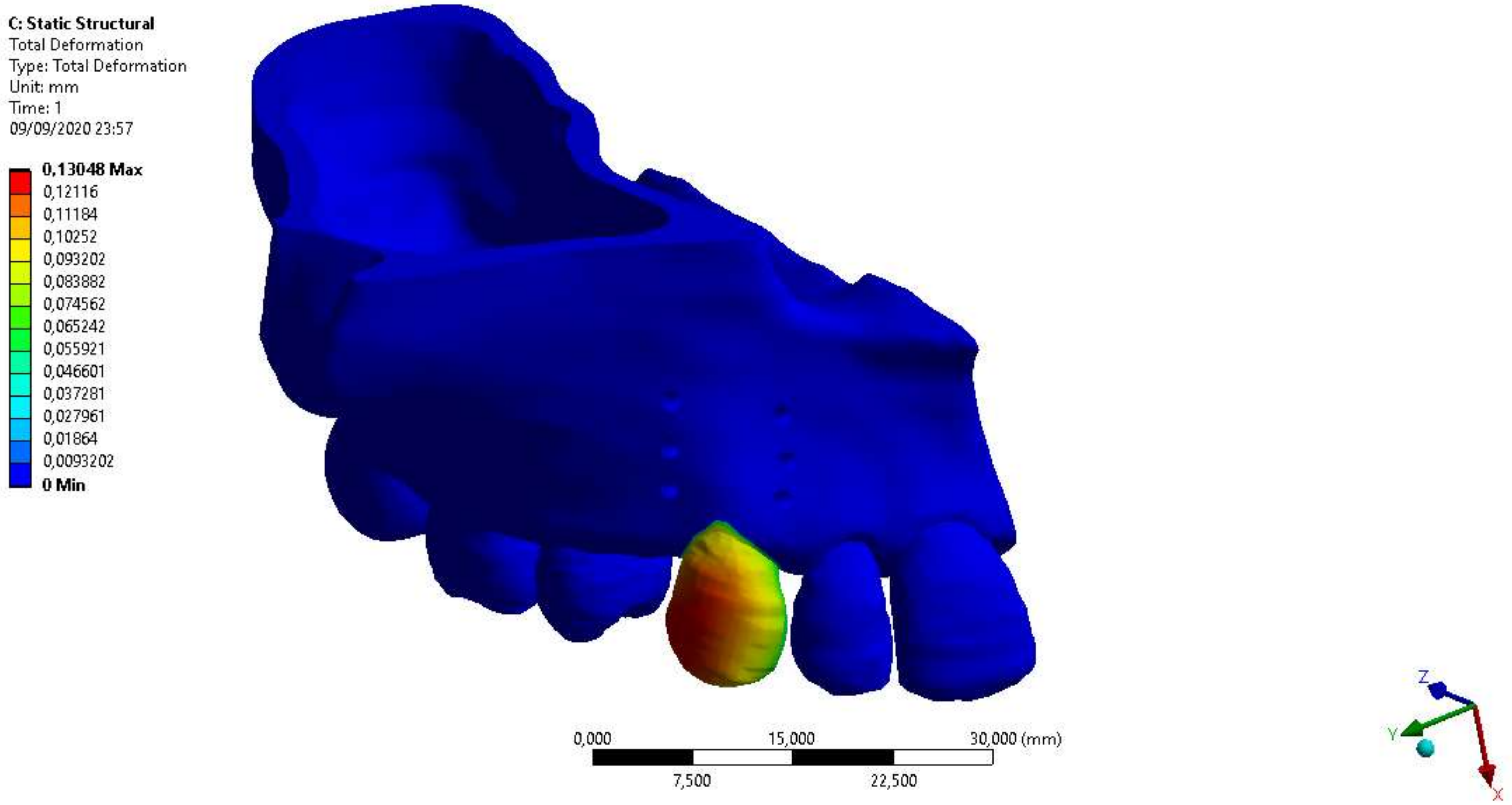
C: Static Structural
Total Deformation
Type: Total Deformation
Unit: mm
Time: 1
09/09/2020 23:57
0,13048 Max
0,12116
0,11184
0,10252
0,093202
0,083882
0,074562
0,065242
0,055921
0,046601
0,037281
0,027961
0,01864
0,0093202
0 Min
0,000
15,000
30,000 (mm)
7,500
22,500
Z
Y
X

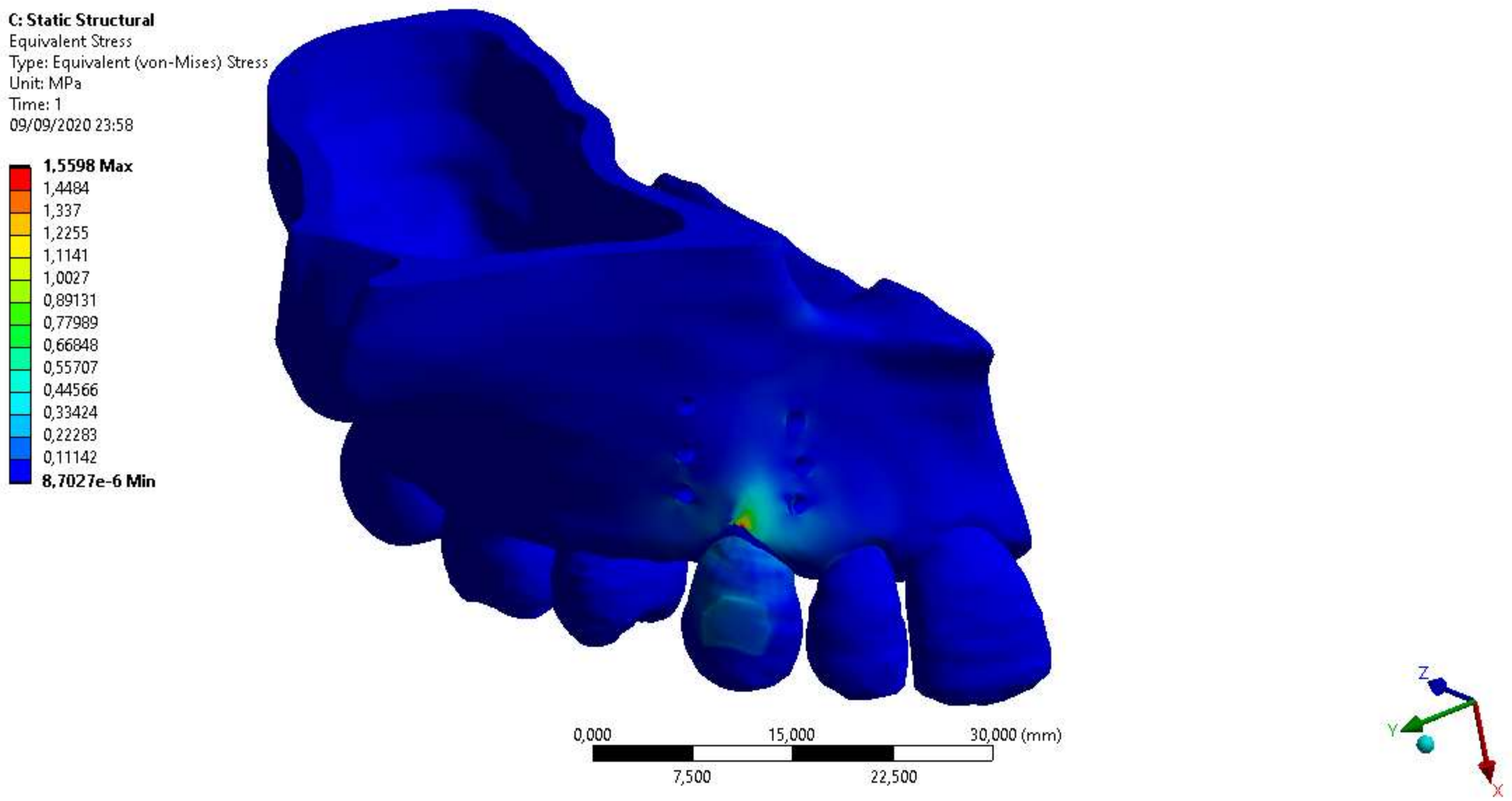
C: Static Structural
Equivalent Stress
Type: Equivalent (von-Mises) Stress
Unit: MPa
Time: 1
09/09/2020 23:58
1,5598 Max
1,4484
1,337
1,2255
1,1141
1,0027
0,89131
0,77989
0,66848
0,55707
0,44566
0,33424
0,22283
0,11142
8,7027e-6 Min
0,000
15,000
30,000 (mm)
7,500
22,500
Z
Y
X

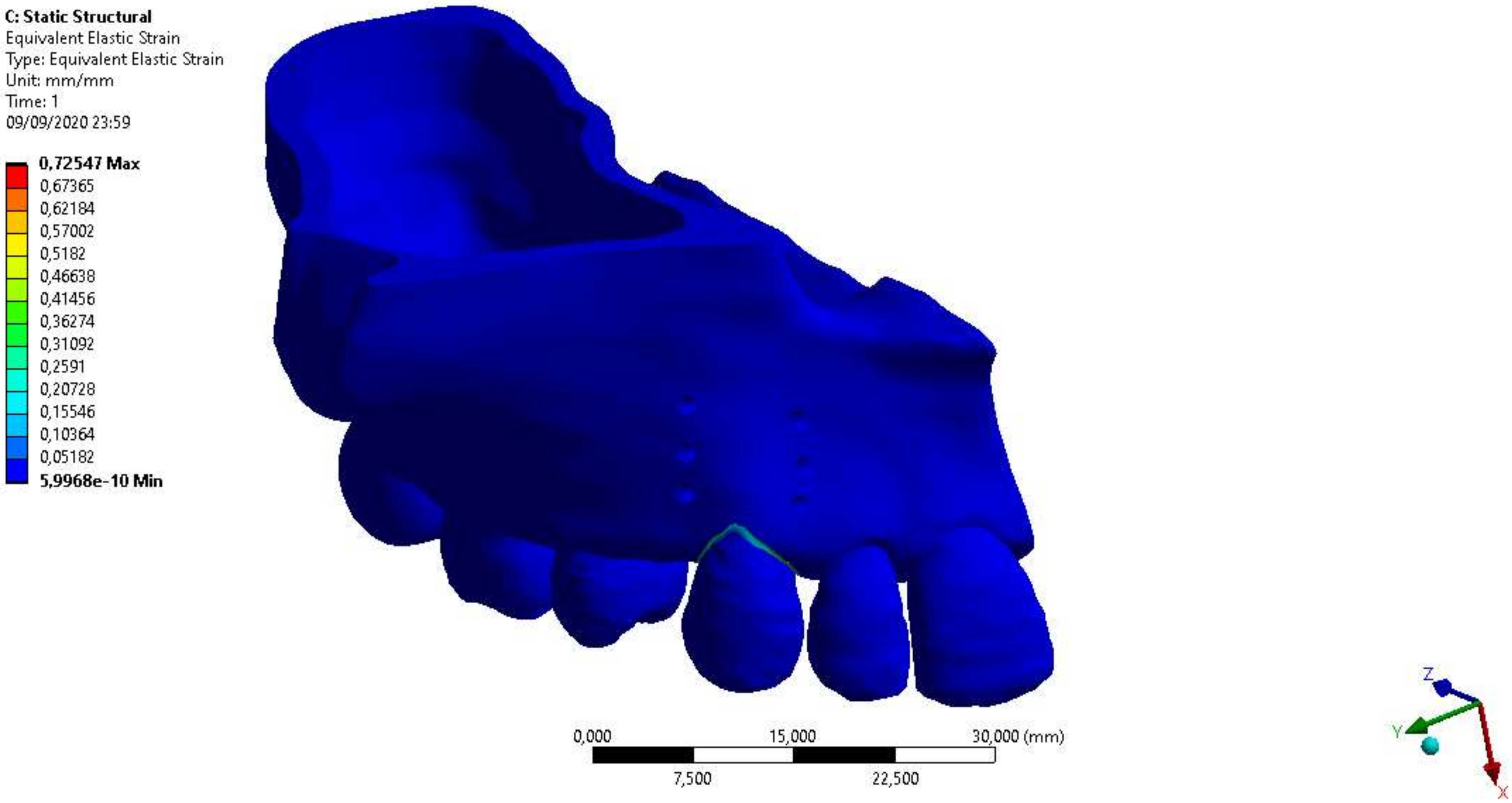
C: Static Structural
Equivalent Elastic Strain
Type: Equivalent Elastic Strain
Unit: mm/mm
Time: 1
09/09/2020 23:59
0,72547 Max
0,67365
0,62184
0,57002
0,5182
0,46638
0,41456
0,36274
0,31092
0,2591
0,20728
0,15546
0,10364
0,05182
5,9968e-10 Min
0,000
15,000
30,000 (mm)
7,500
22,500
Z
Y
X

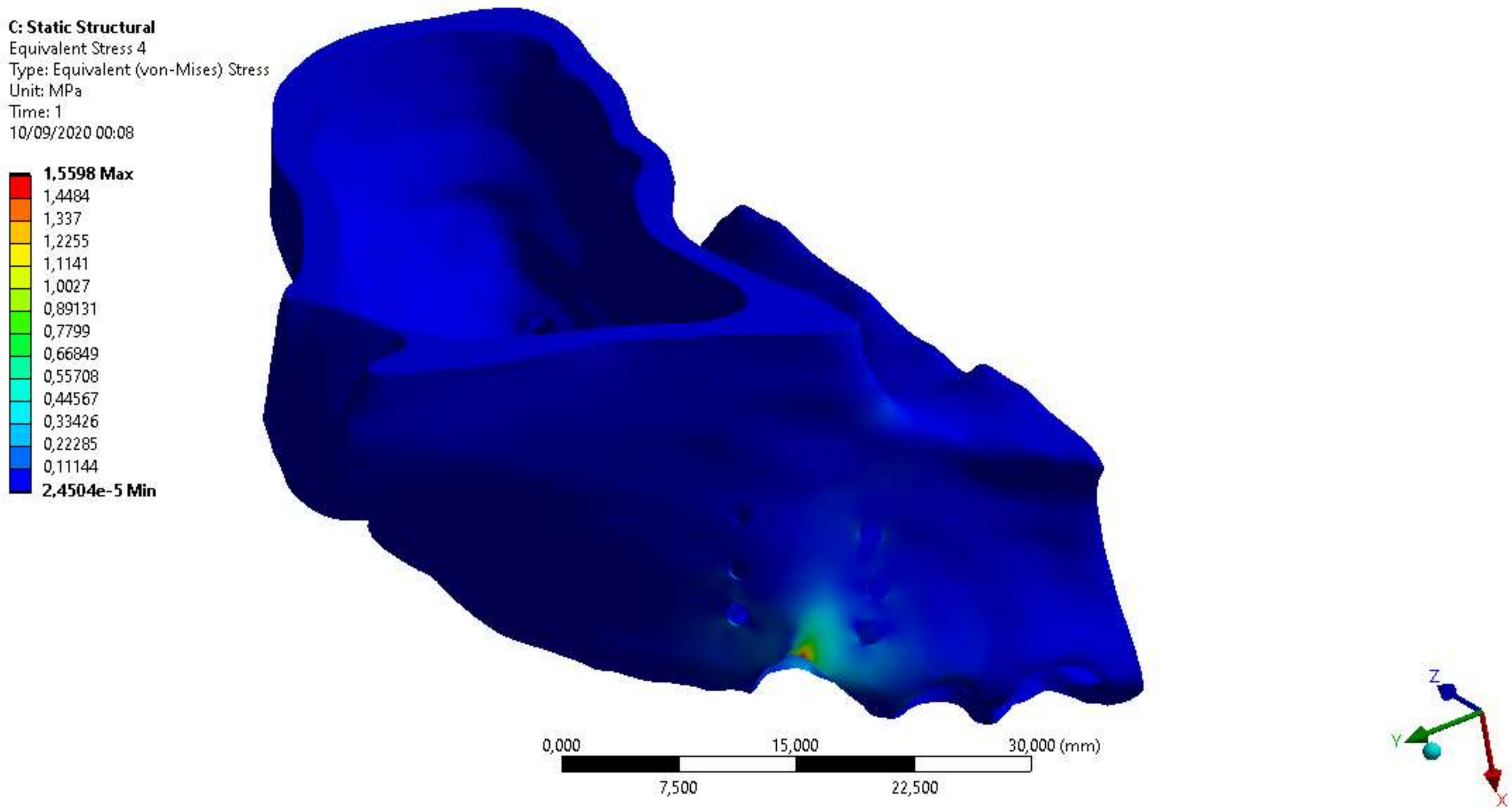
C: Static Structural
Equivalent Stress 4
Type: Equivalent (von-Mises) Stress
Unit: MPa
Time: 1
10/09/2020 00:08
1,5598 Max
1,4484
1,337
1,2255
1,1141
1,0027
0,89131
0,7799
0,66849
0,55708
0,44567
0,33426
0,22285
0,11144
2,4504e-5 Min
0,000
15,000
30,000 (mm)
7,500
22,500
Z
Y
X

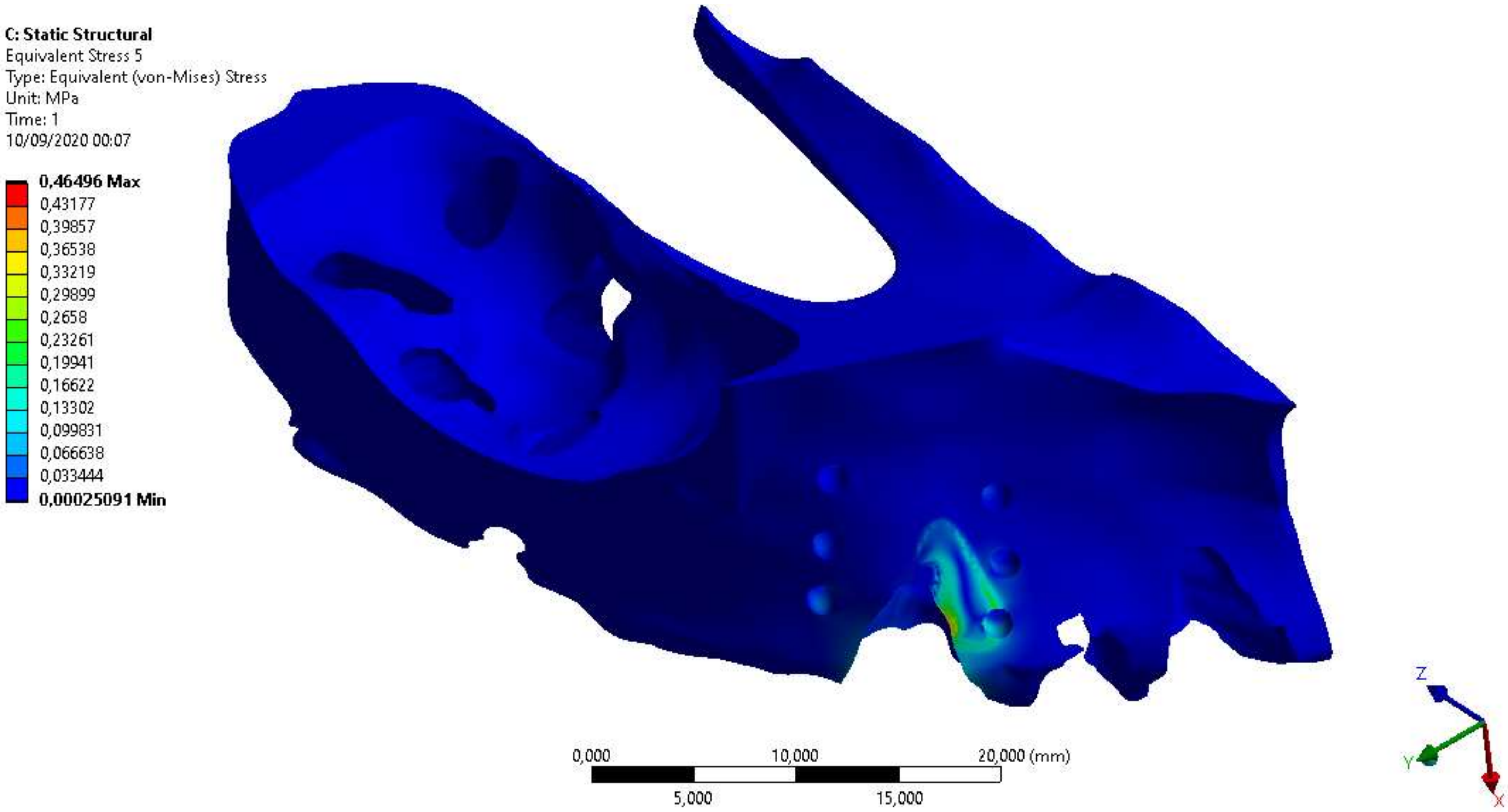
C: Static Structural
Equivalent Stress 5
Type: Equivalent (von-Mises) Stress
Unit: MPa
Time: 1
10/09/2020 00:07
0,46496 Max
0,43177
0,39857
0,36538
0,33219
0,29899
0,2658
0,23261
0,19941
0,16622
0,13302
0,099831
0,066638
0,033444
0,00025091 Min
0,000
10,000
20,000 (mm)
5,000
15,000
Z
Y
X

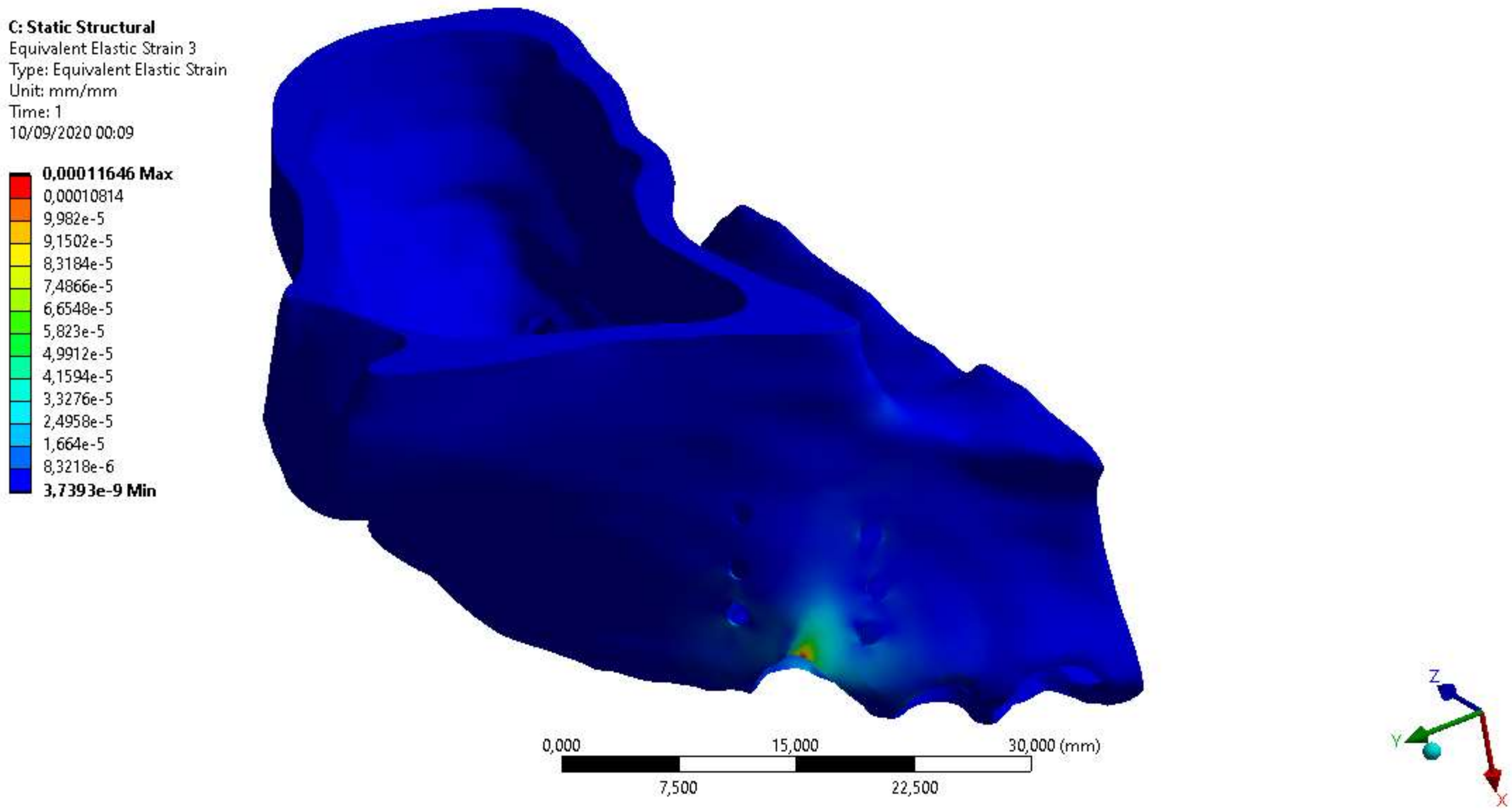
C: Static Structural
Equivalent Elastic Strain 3
Type: Equivalent Elastic Strain
Unit: mm/mm
Time: 1
10/09/2020 00:09
0,00011646 Max
0,00010814
9,982e-5
9,1502e-5
8,3184e-5
7,4866e-5
6,6548e-5
5,823e-5
4,9912e-5
4,1594e-5
3,3276e-5
2,4958e-5
1,664e-5
8,3218e-6
3,7393e-9 Min
0,000
15,000
30,000 (mm)
7,500
22,500
Z
Y
X

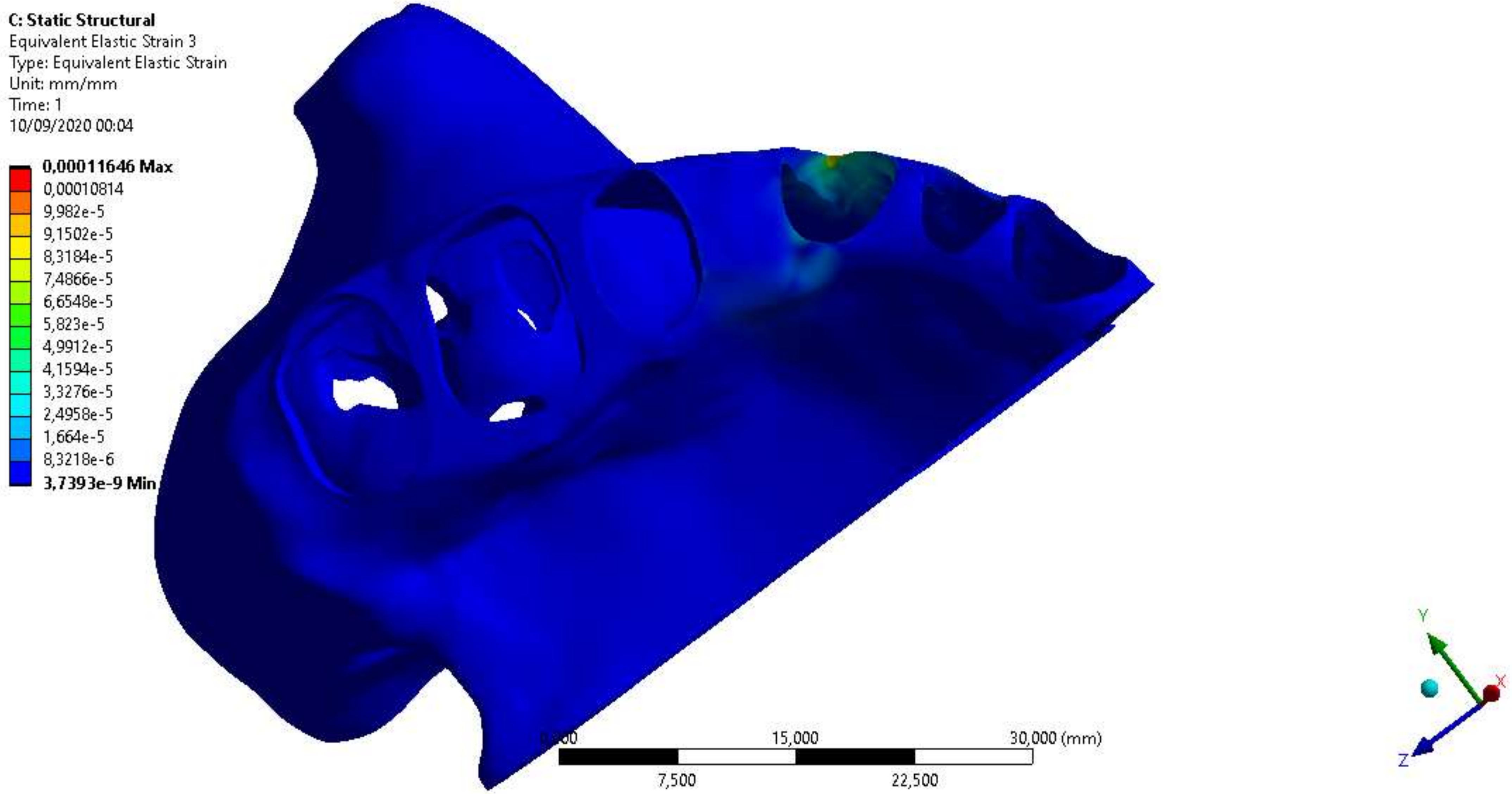
C: Static Structural
Equivalent Elastic Strain 3
Type: Equivalent Elastic Strain
Unit: mm/mm
Time: 1
10/09/2020 00:04
0,00011646 Max
0,00010814
9,982e-5
9,1502e-5
8,3184e-5
7,4866e-5
6,6548e-5
5,823e-5
4,9912e-5
4,1594e-5
3,3276e-5
2,4958e-5
1,664e-5
8,3218e-6
3,7393e-9 Min
15,000
30,000 (mm)
7,500
22,500
Y
X
Z

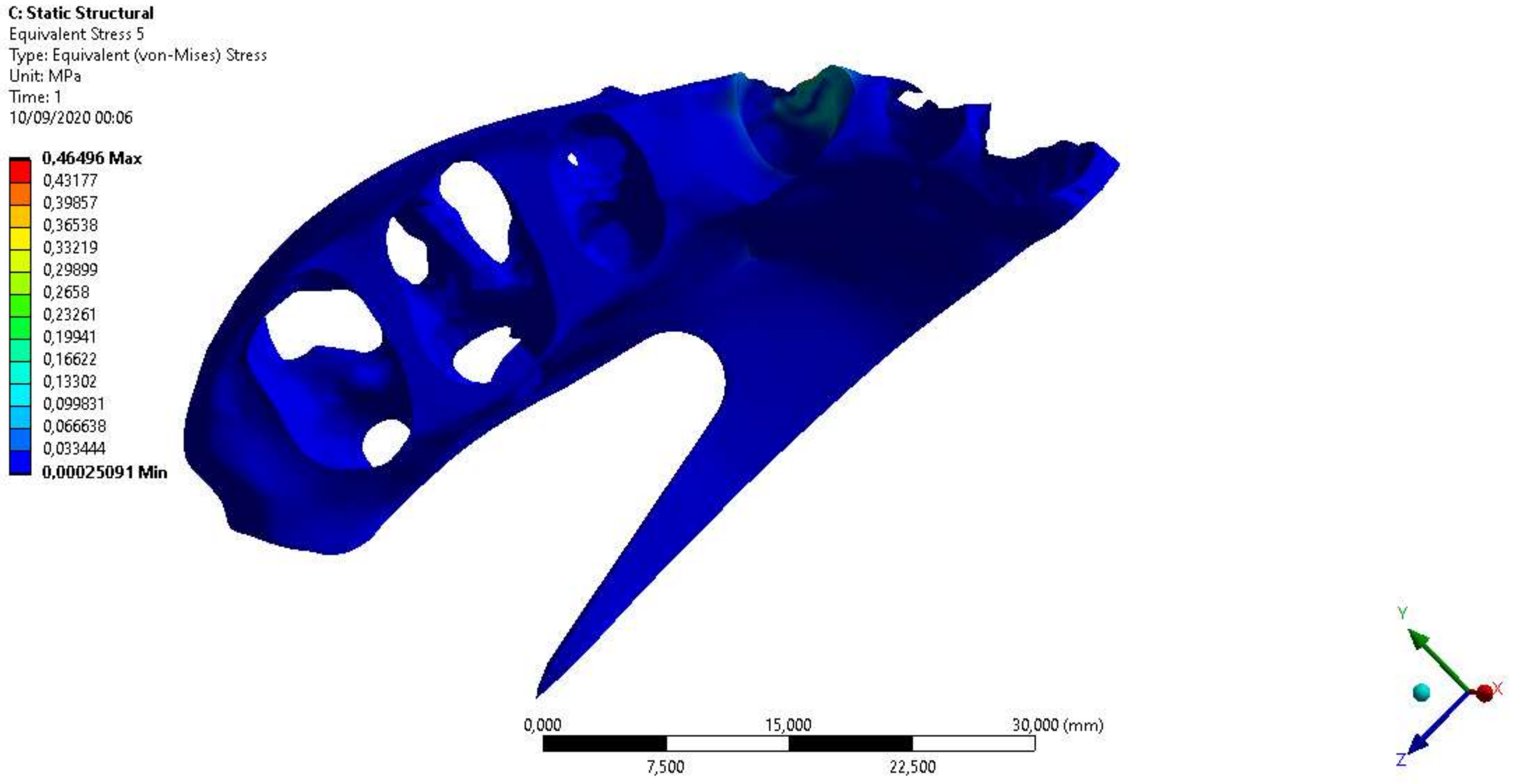
C: Static Structural
Equivalent Stress 5
Type: Equivalent (von-Mises) Stress
Unit: MPa
Time: 1
10/09/2020 00:06
0,46496 Max
0,43177
0,39857
0,36538
0,33219
0,29899
0,2658
0,23261
0,19941
0,16622
0,13302
0,099831
0,066638
0,033444
0,00025091 Min
0,000
15,000
30,000 (mm)
7,500
22,500
Y
X
Z

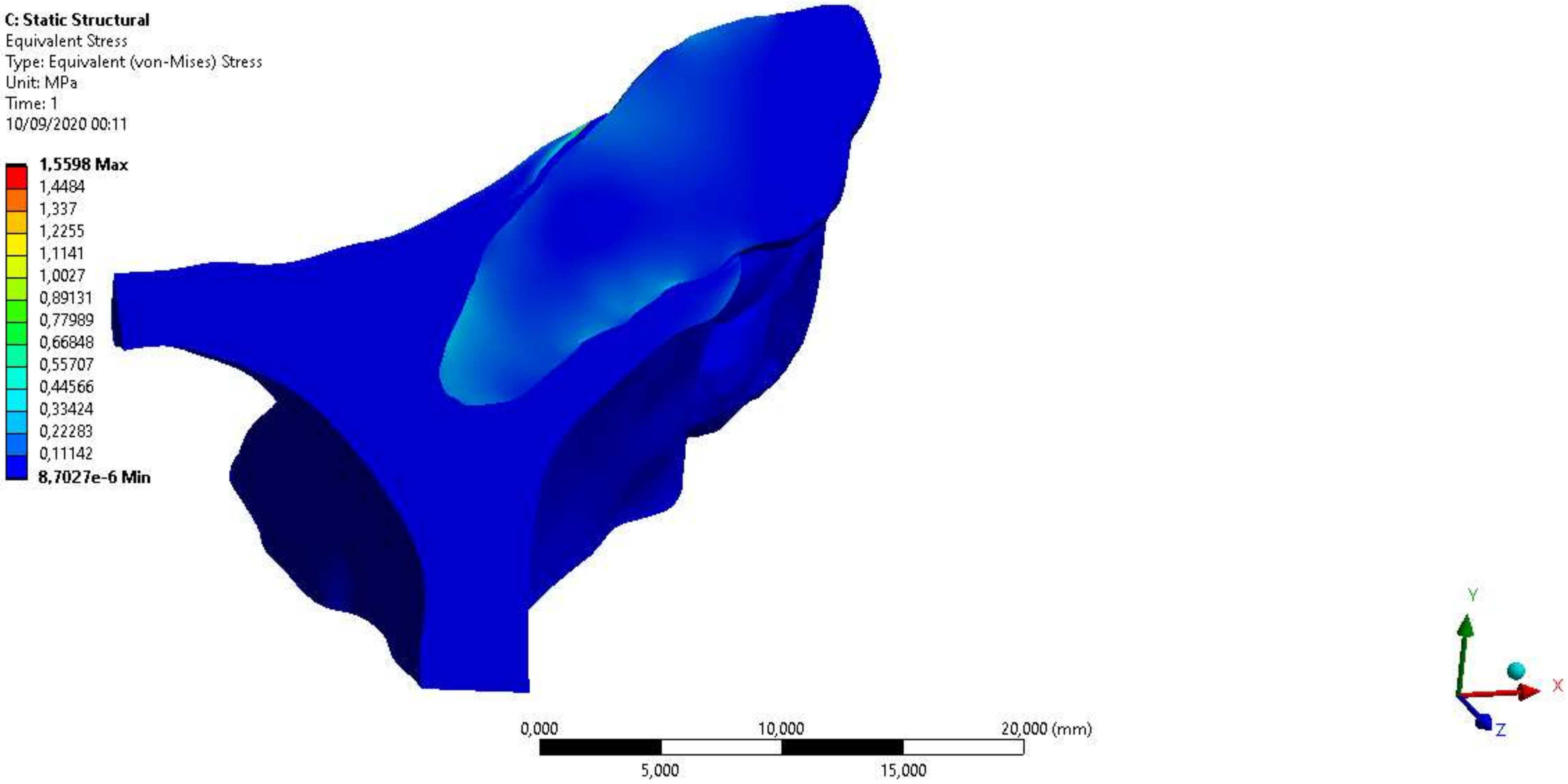
C: Static Structural
Equivalent Stress
Type: Equivalent (von-Mises) Stress
Unit: MPa
Time: 1
10/09/2020 00:11
1,5598 Max
1,4484
1,337
1,2255
1,1141
1,0027
0,89131
0,77989
0,66848
0,55707
0,44566
0,33424
0,22283
0,11142
8,7027e-6 Min
0,000
5,000
10,000
15,000
20,000 (mm)
Y
X
Z

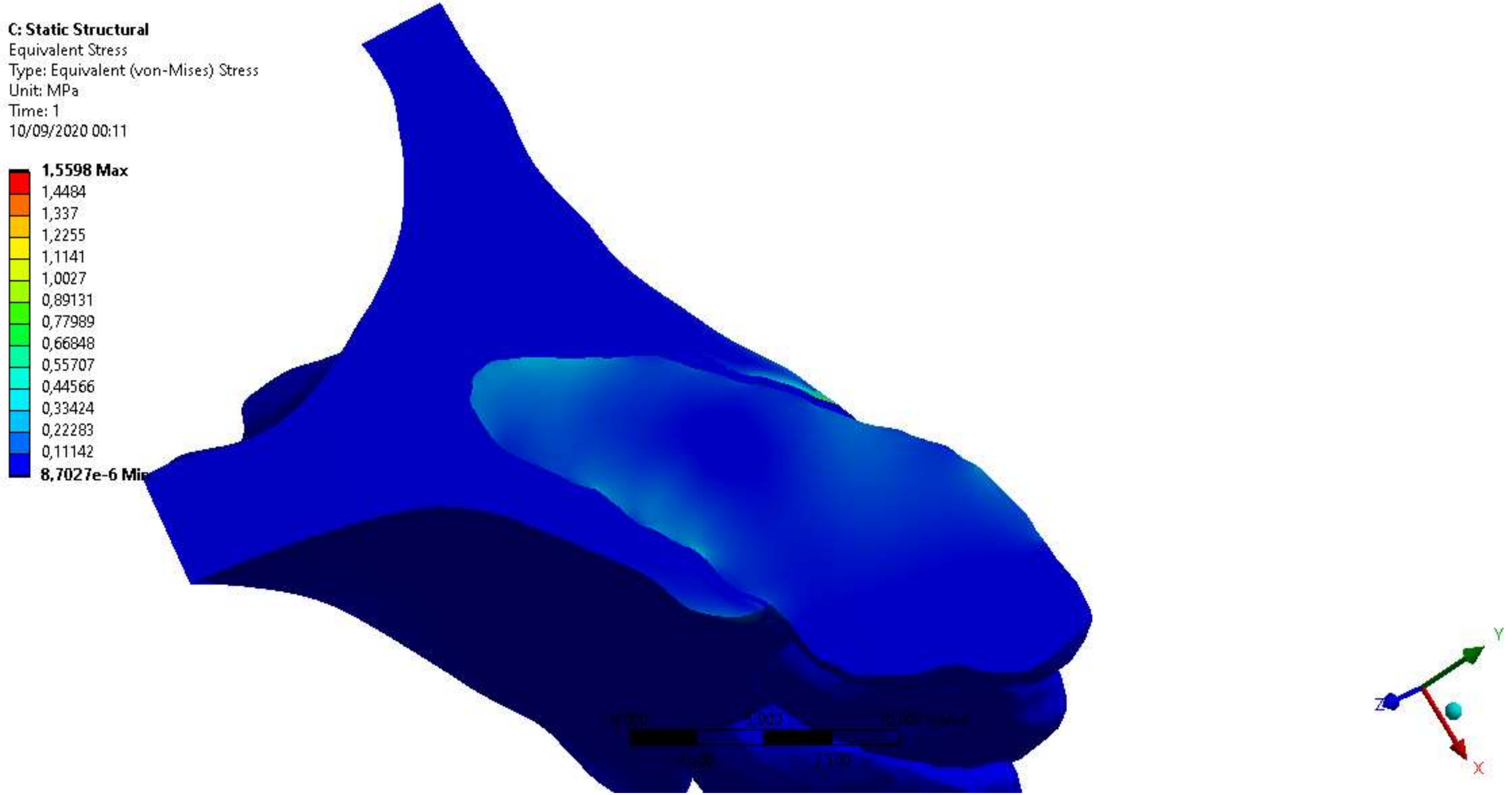
C: Static Structural
Equivalent Stress
Type: Equivalent (von-Mises) Stress
Unit: MPa
Time: 1
10/09/2020 00:11
1,5598 Max
1,4484
1,337
1,2255
1,1141
1,0027
0,89131
0,77989
0,66848
0,55707
0,44566
0,33424
0,22283
0,11142
8,7027e-6 Min
Y
Z
X

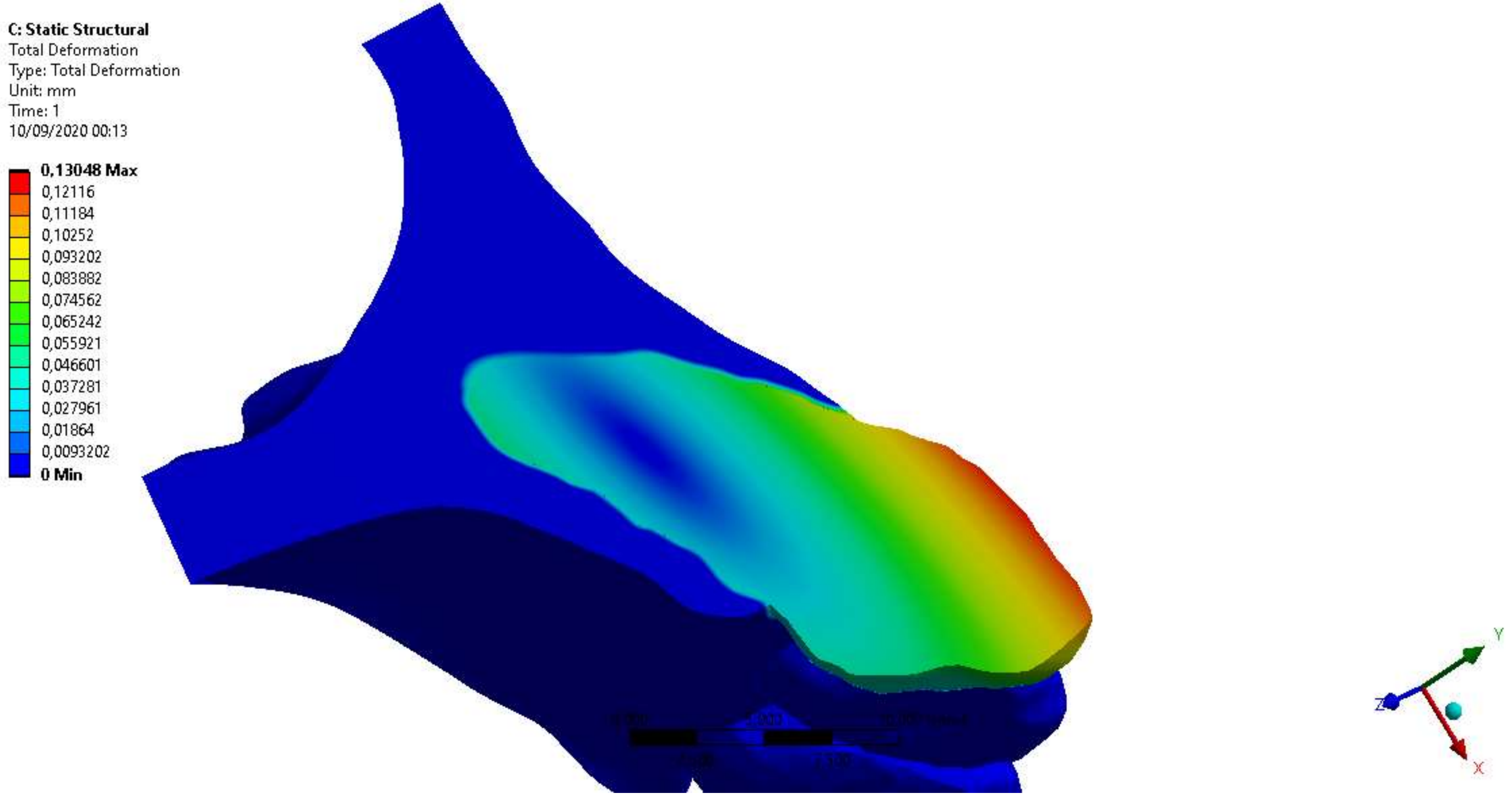
C: Static Structural
Total Deformation
Type: Total Deformation
Unit: mm
Time: 1
10/09/2020 00:13
0,13048 Max
0,12116
0,11184
0,10252
0,093202
0,083882
0,074562
0,065242
0,055921
0,046601
0,037281
0,027961
0,01864
0,0093202
0 Min
Y
Z
X

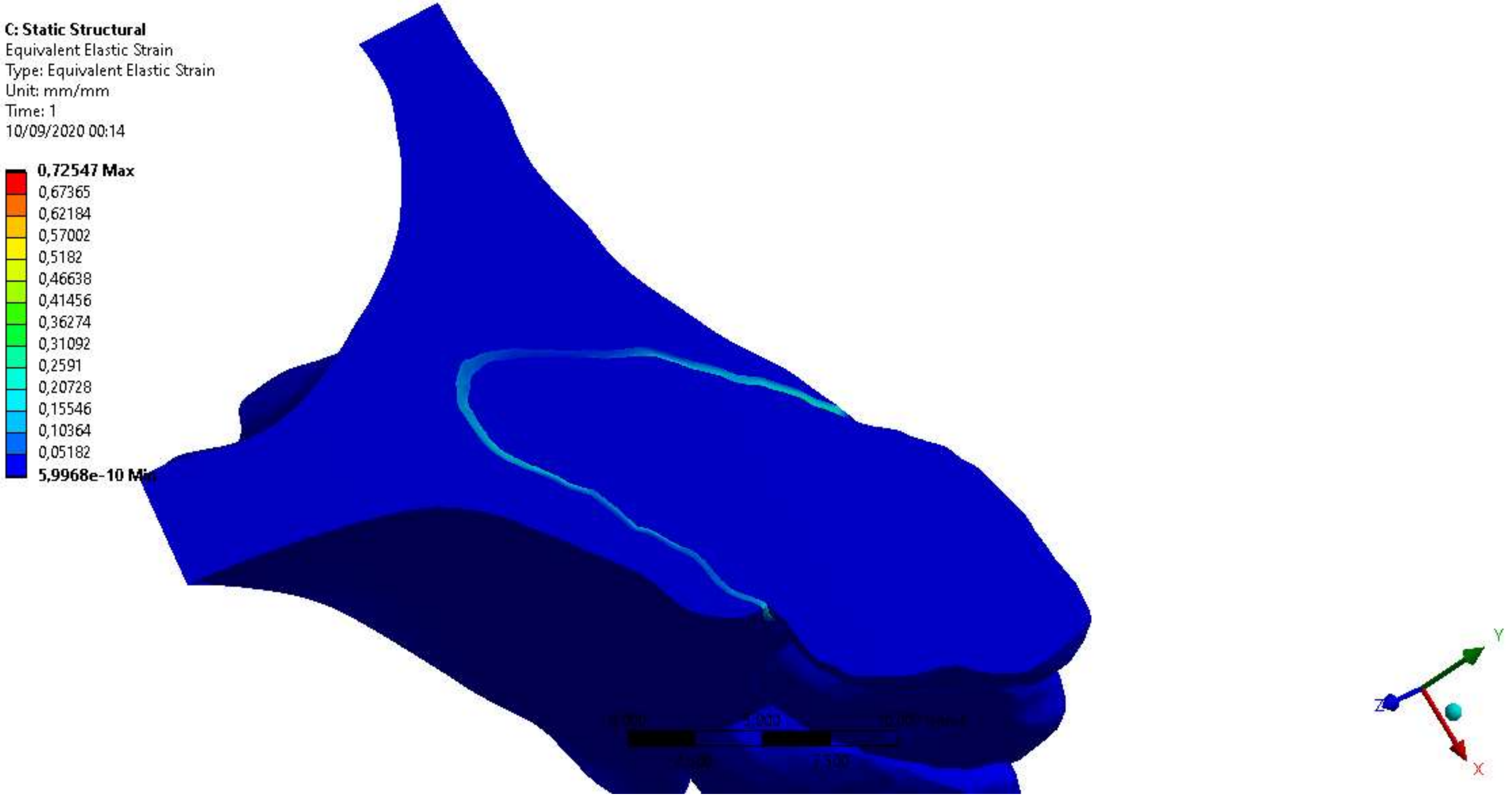
C: Static Structural
Equivalent Elastic Strain
Type: Equivalent Elastic Strain
Unit: mm/mm
Time: 1
10/09/2020 00:14
0,72547 Max
0,67365
0,62184
0,57002
0,5182
0,46638
0,41456
0,36274
0,31092
0,2591
0,20728
0,15546
0,10364
0,05182
5,9968e-10 Min
Y
Z
X

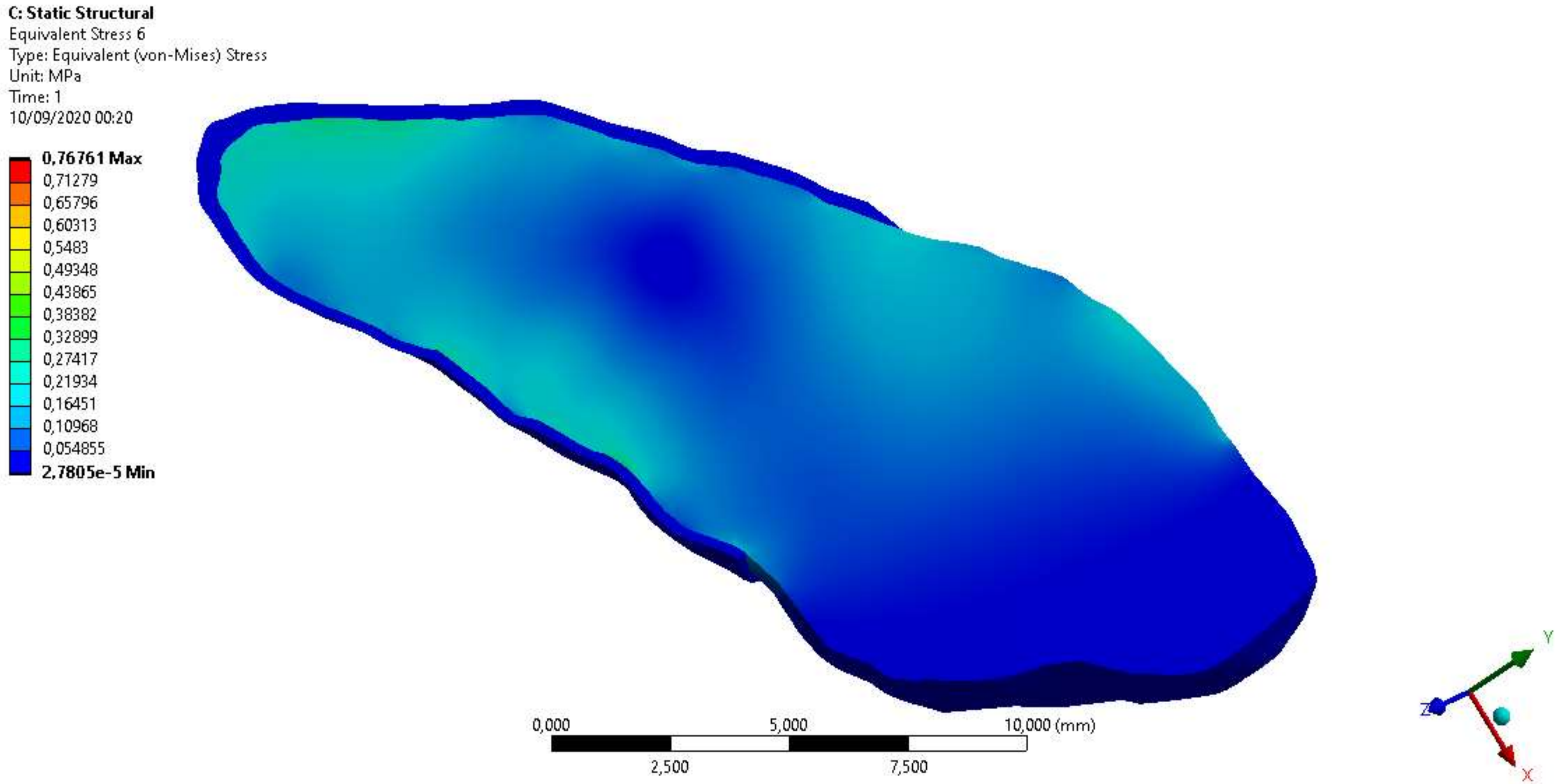
C: Static Structural
Equivalent Stress 6
Type: Equivalent (von-Mises) Stress
Unit: MPa
Time: 1
10/09/2020 00:20
0,76761 Max
0,71279
0,65796
0,60313
0,5483
0,49348
0,43865
0,38382
0,32899
0,27417
0,21934
0,16451
0,10968
0,054855
2,7805e-5 Min
0,000
5,000
10,000 (mm)
2,500
7,500
Y
Z
X

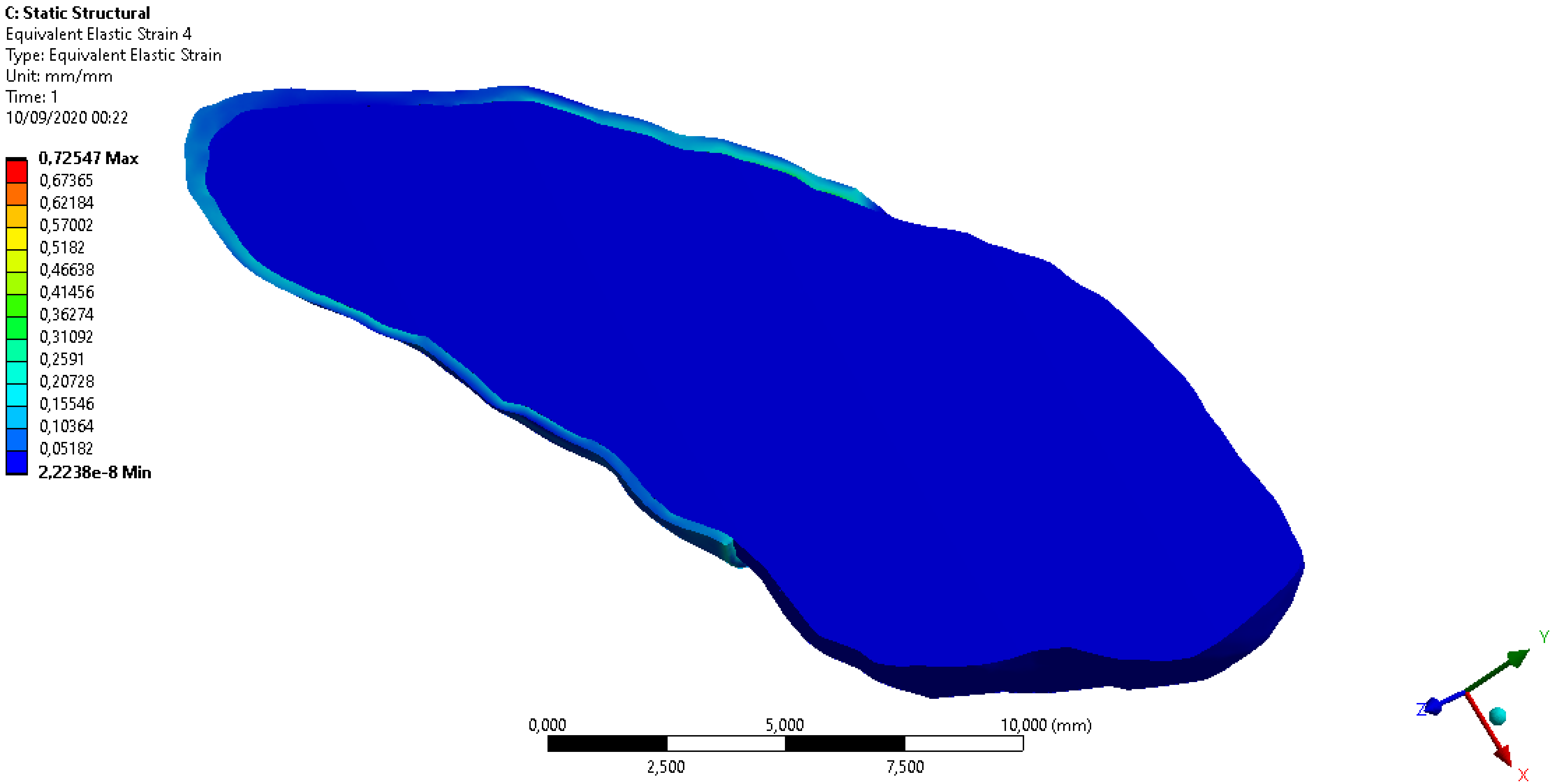

C: Static Structural
Equivalent Elastic Strain 4
Type: Equivalent Elastic Strain
Unit: mm/mm
Time: 1
10/09/2020 00:22
0,72547 Max
0,67365
0,62184
0,57002
0,5182
0,46638
0,41456
0,36274
0,31092
0,2591
0,20728
0,15546
0,10364
0,05182
2,2238e-8 Min
0,000
5,000
10,000 (mm)
2,500
7,500
Y
Z
X

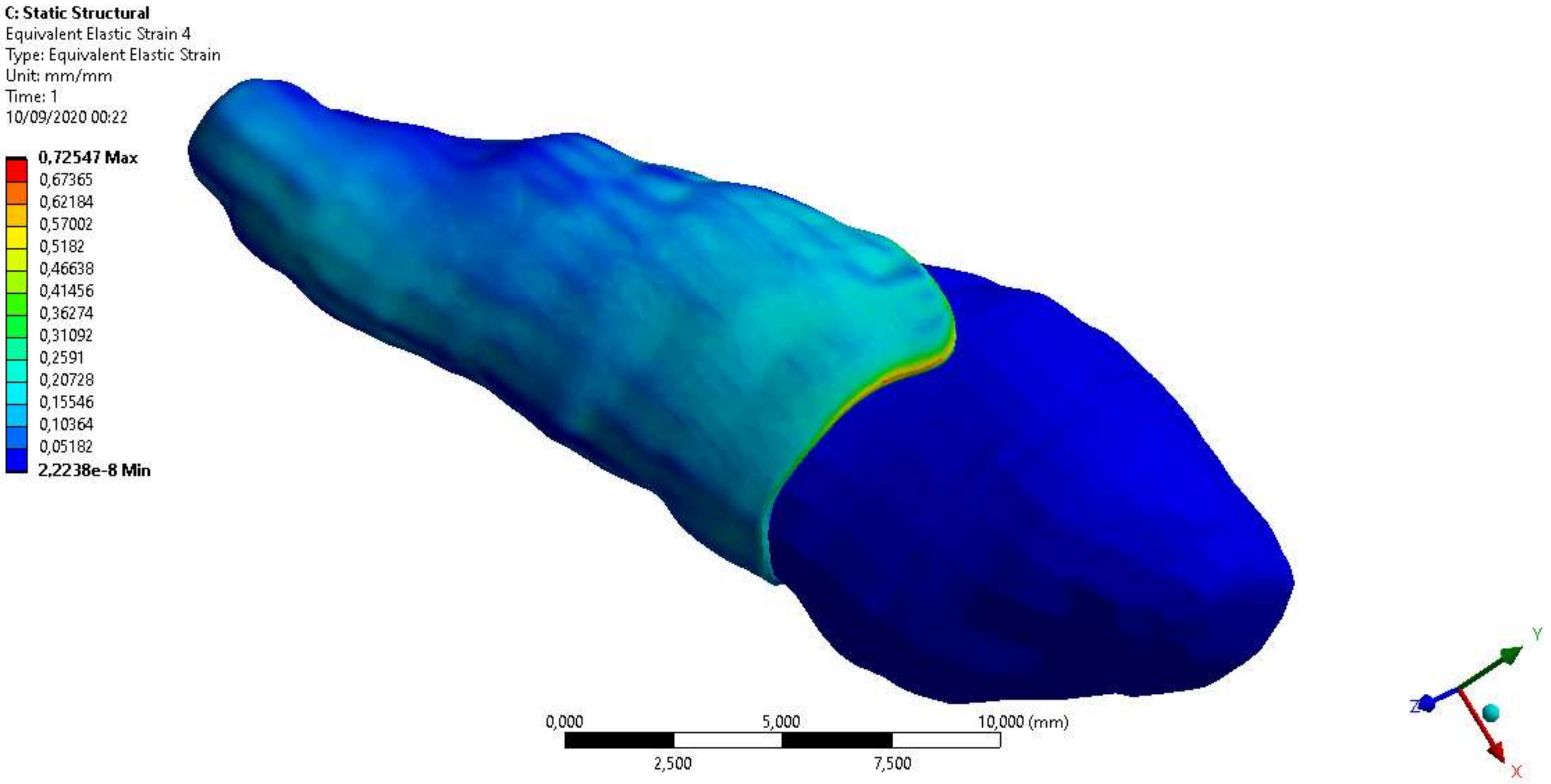
C: Static Structural
Equivalent Elastic Strain 4
Type: Equivalent Elastic Strain
Unit: mm/mm
Time: 1
10/09/2020 00:22
0,72547 Max
0,67365
0,62184
0,57002
0,5182
0,46638
0,41456
0,36274
0,31092
0,2591
0,20728
0,15546
0,10364
0,05182
2,2238e-8 Min
0,000
5,000
10,000 (mm)
2,500
7,500
Y
Z
X

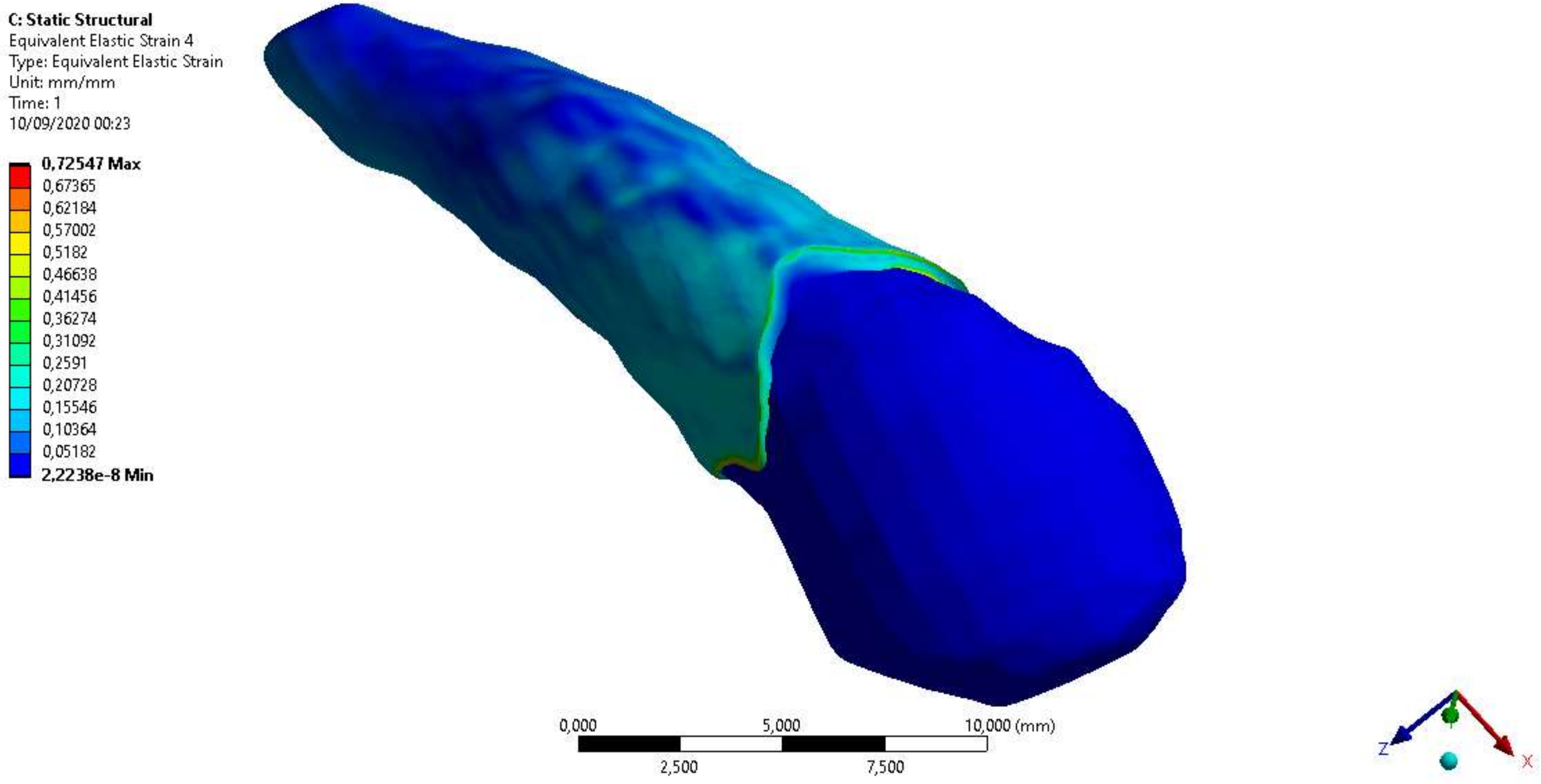
C: Static Structural
Equivalent Elastic Strain 4
Type: Equivalent Elastic Strain
Unit: mm/mm
Time: 1
10/09/2020 00:23
0,72547 Max
0,67365
0,62184
0,57002
0,5182
0,46638
0,41456
0,36274
0,31092
0,2591
0,20728
0,15546
0,10364
0,05182
2,2238e-8 Min
0,000
5,000
10,000 (mm)
2,500
7,500
Z
X

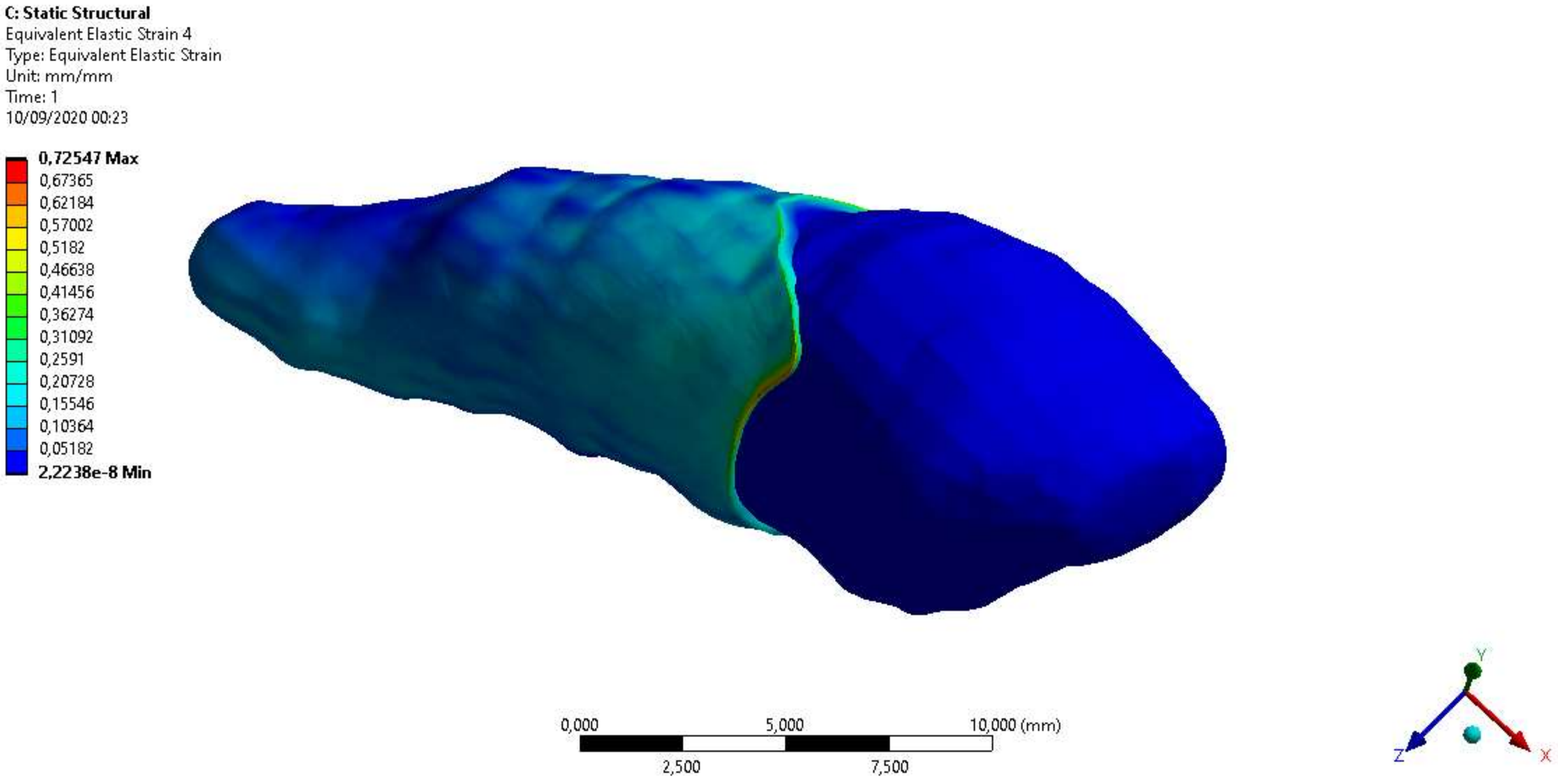
C: Static Structural
Equivalent Elastic Strain 4
Type: Equivalent Elastic Strain
Unit: mm/mm
Time: 1
10/09/2020 00:23
0,72547 Max
0,67365
0,62184
0,57002
0,5182
0,46638
0,41456
0,36274
0,31092
0,2591
0,20728
0,15546
0,10364
0,05182
2,2238e-8 Min
0,000
5,000
10,000 (mm)
2,500
7,500
Y
Z
X

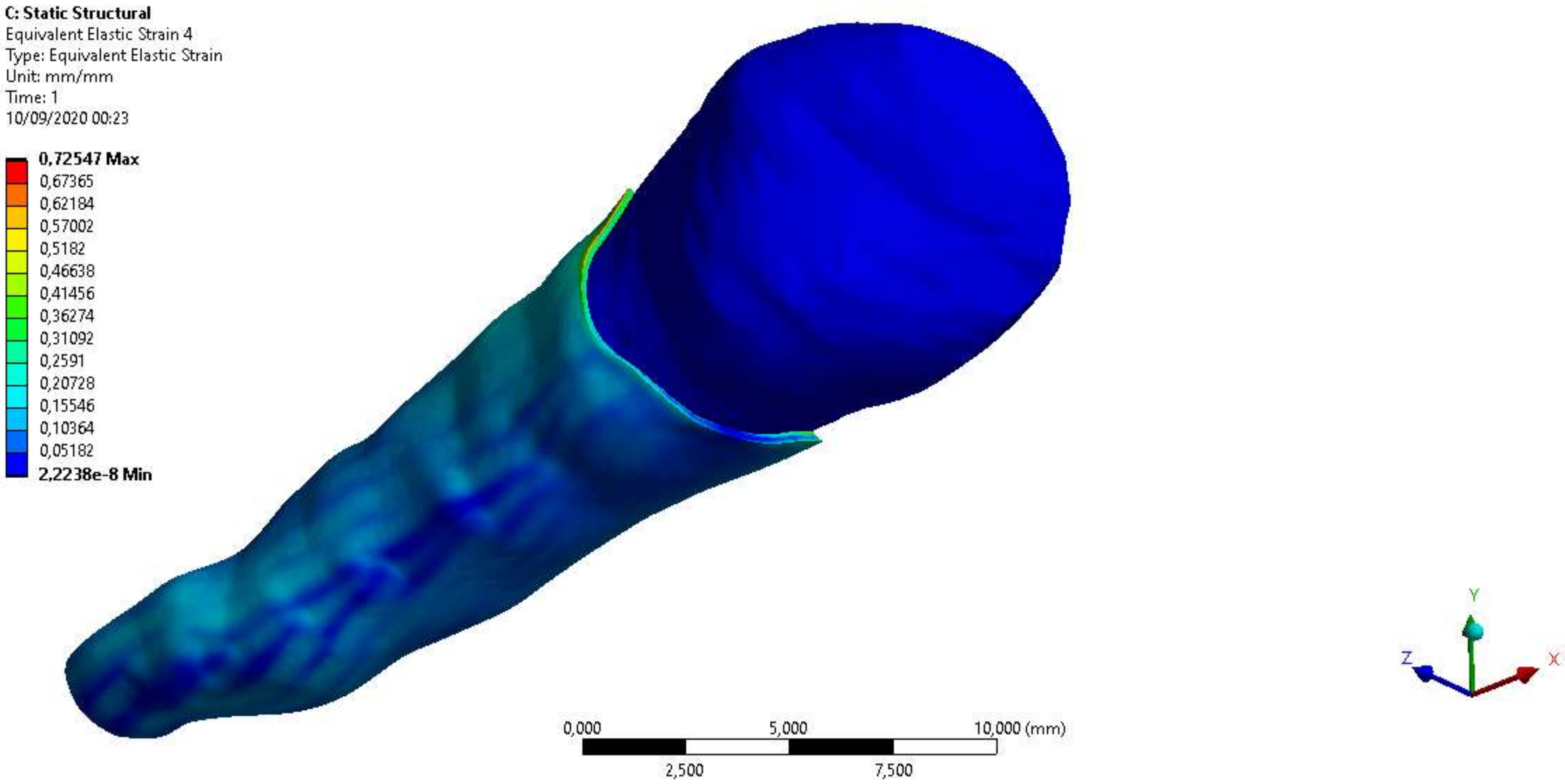
C: Static Structural
Equivalent Elastic Strain 4
Type: Equivalent Elastic Strain
Unit: mm/mm
Time: 1
10/09/2020 00:23
0,72547 Max
0,67365
0,62184
0,57002
0,5182
0,46638
0,41456
0,36274
0,31092
0,2591
0,20728
0,15546
0,10364
0,05182
2,2238e-8 Min
0,000
5,000
10,000 (mm)
2,500
7,500
Y
Z
X

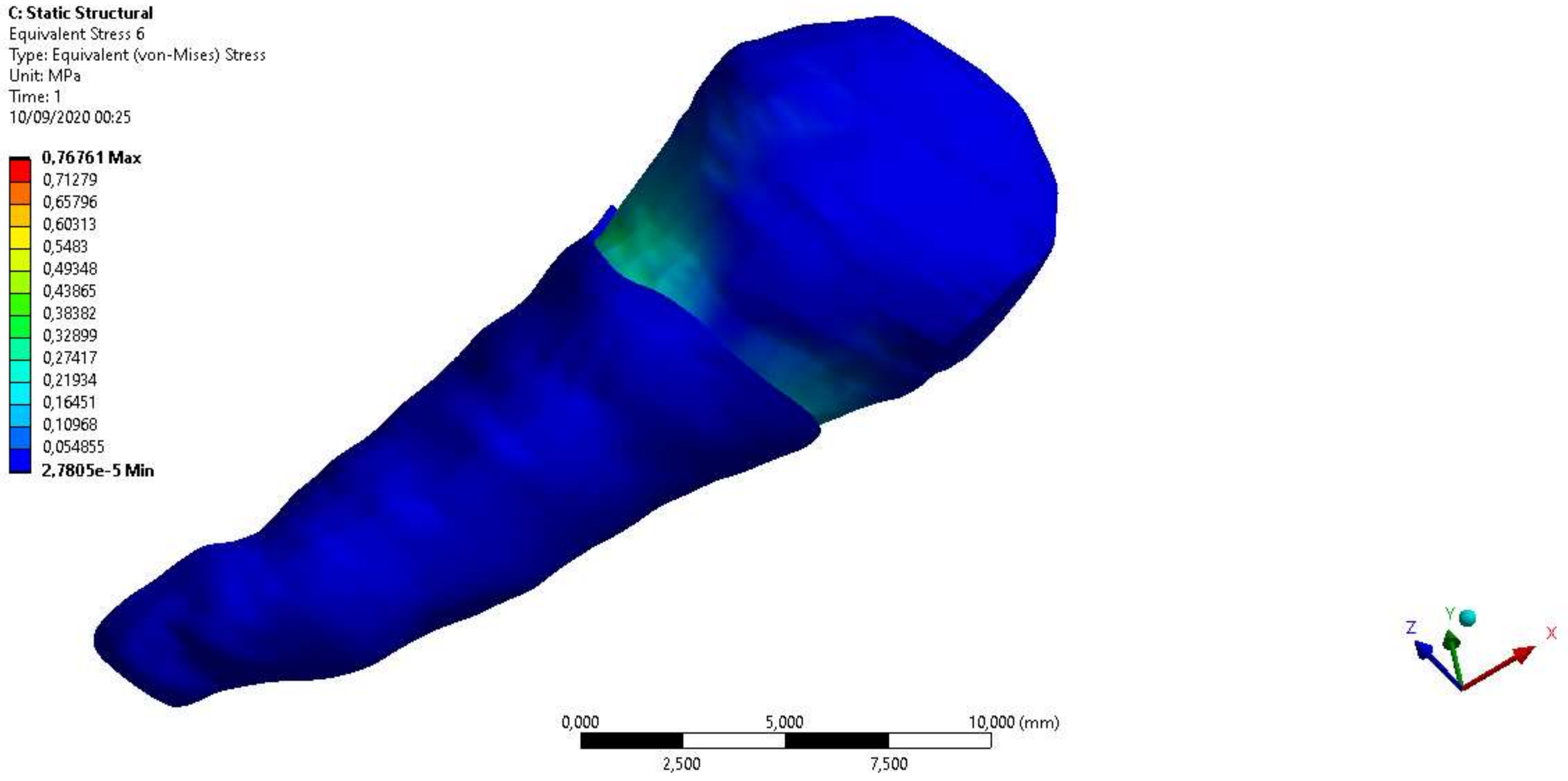
C: Static Structural
Equivalent Stress 6
Type: Equivalent (von-Mises) Stress
Unit: MPa
Time: 1
10/09/2020 00:25
0,76761 Max
0,71279
0,65796
0,60313
0,5483
0,49348
0,43865
0,38382
0,32899
0,27417
0,21934
0,16451
0,10968
0,054855
2,7805e-5 Min
0,000
5,000
10,000 (mm)
2,500
7,500
Z
Y
X

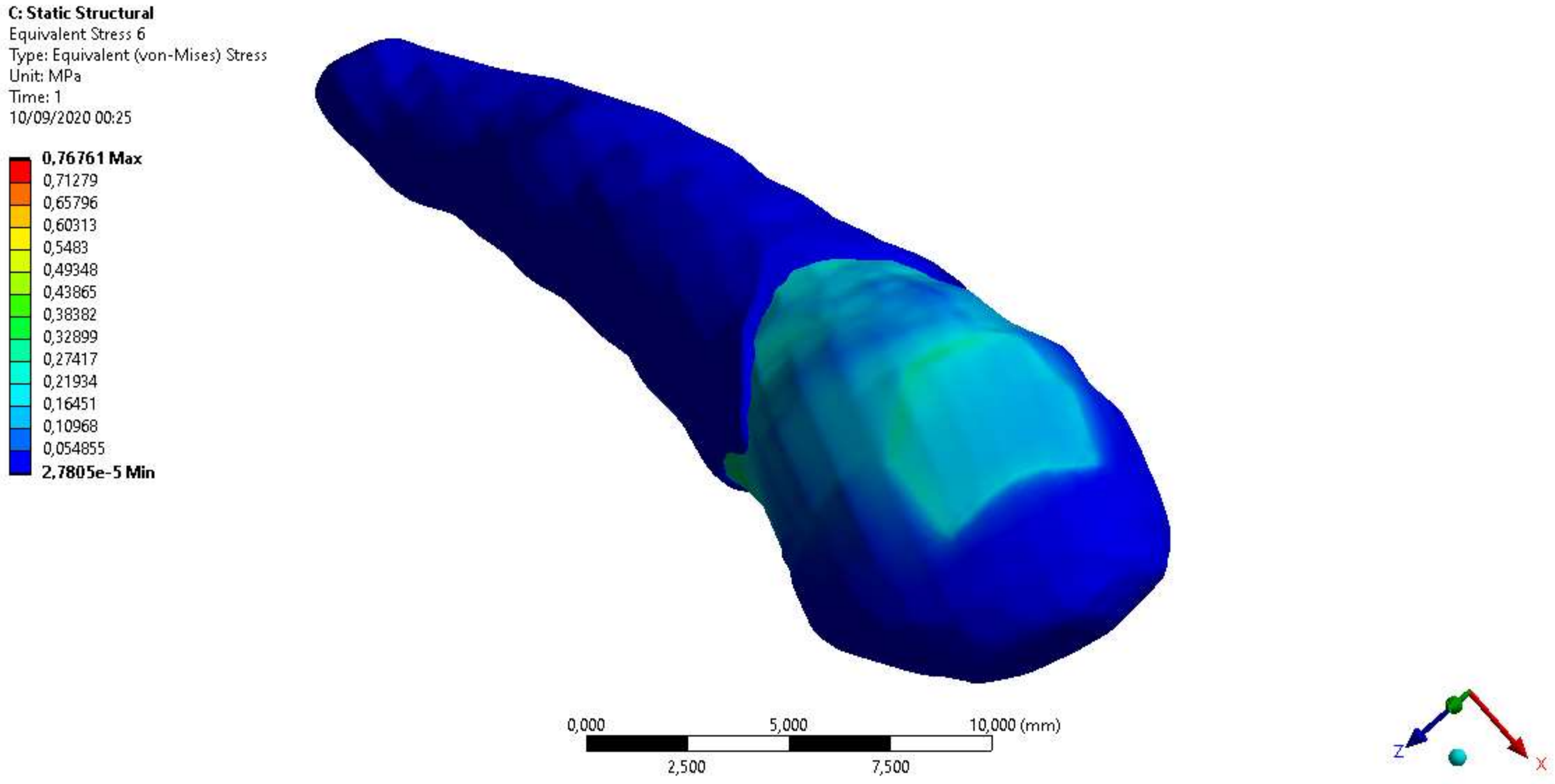
C: Static Structural
Equivalent Stress 6
Type: Equivalent (von-Mises) Stress
Unit: MPa
Time: 1
10/09/2020 00:25
0,76761 Max
0,71279
0,65796
0,60313
0,5483
0,49348
0,43865
0,38382
0,32899
0,27417
0,21934
0,16451
0,10968
0,054855
2,7805e-5 Min
0,000
5,000
10,000 (mm)
2,500
7,500
Z
X

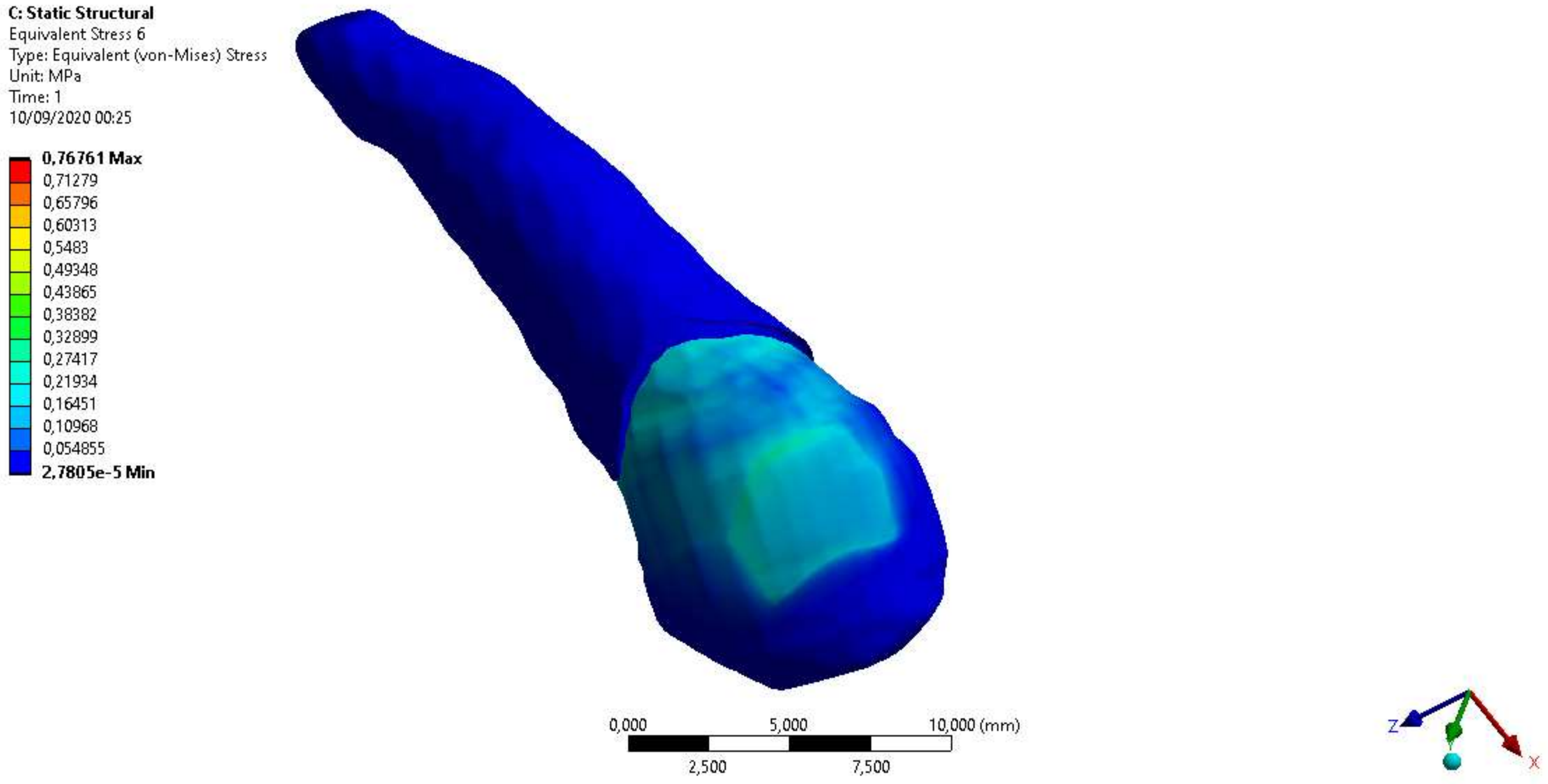
C: Static Structural
Equivalent Stress 6
Type: Equivalent (von-Mises) Stress
Unit: MPa
Time: 1
10/09/2020 00:25
0,76761 Max
0,71279
0,65796
0,60313
0,5483
0,49348
0,43865
0,38382
0,32899
0,27417
0,21934
0,16451
0,10968
0,054855
2,7805e-5 Min
0,000
5,000
10,000 (mm)
2,500
7,500
Z
Y
X

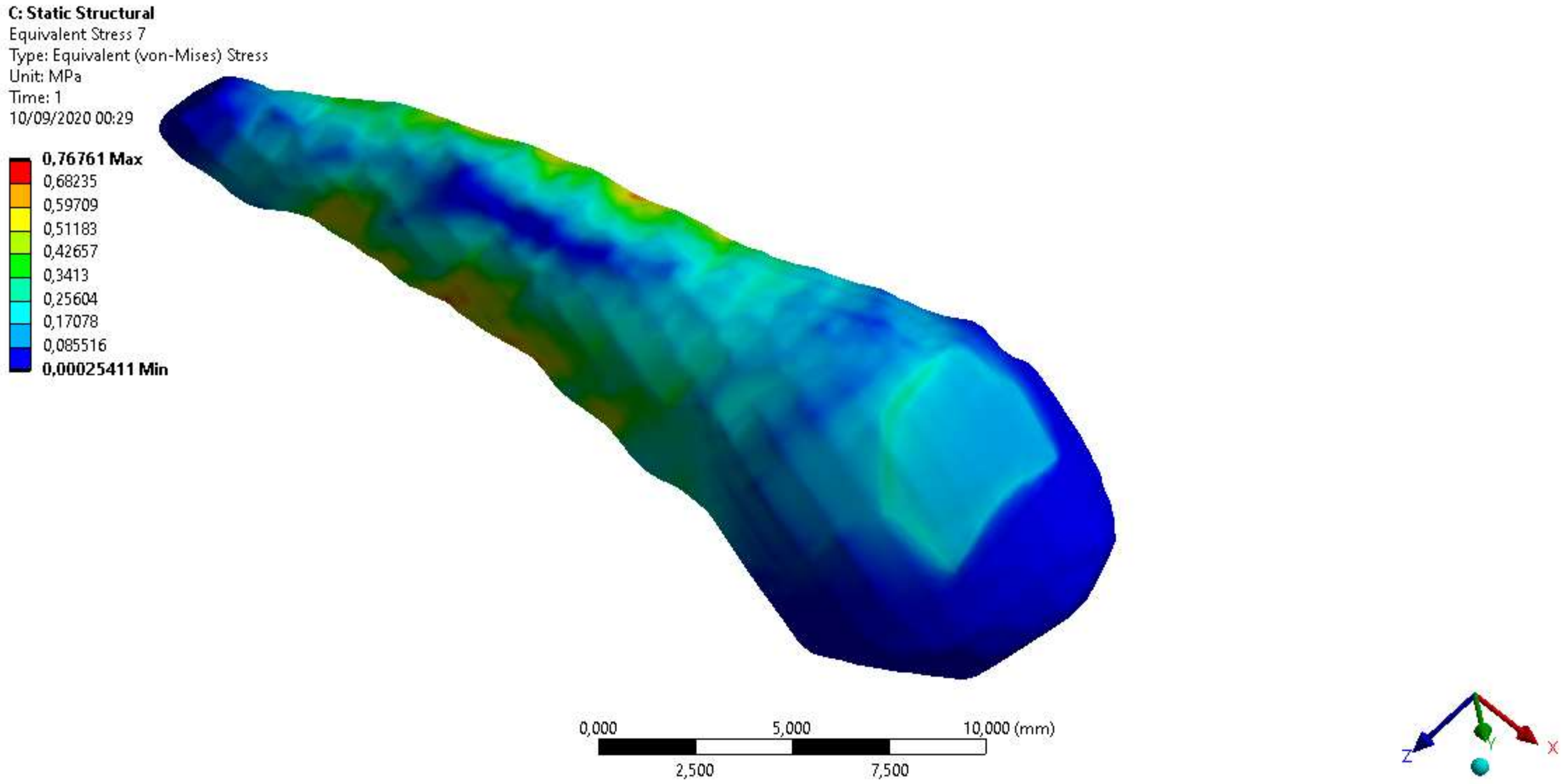
C: Static Structural
Equivalent Stress 7
Type: Equivalent (von-Mises) Stress
Unit: MPa
Time: 1
10/09/2020 00:29
0,76761 Max
0,68235
0,59709
0,51183
0,42657
0,3413
0,25604
0,17078
0,085516
0,00025411 Min
0,000
5,000
10,000 (mm)
2,500
7,500
Z
Y
X

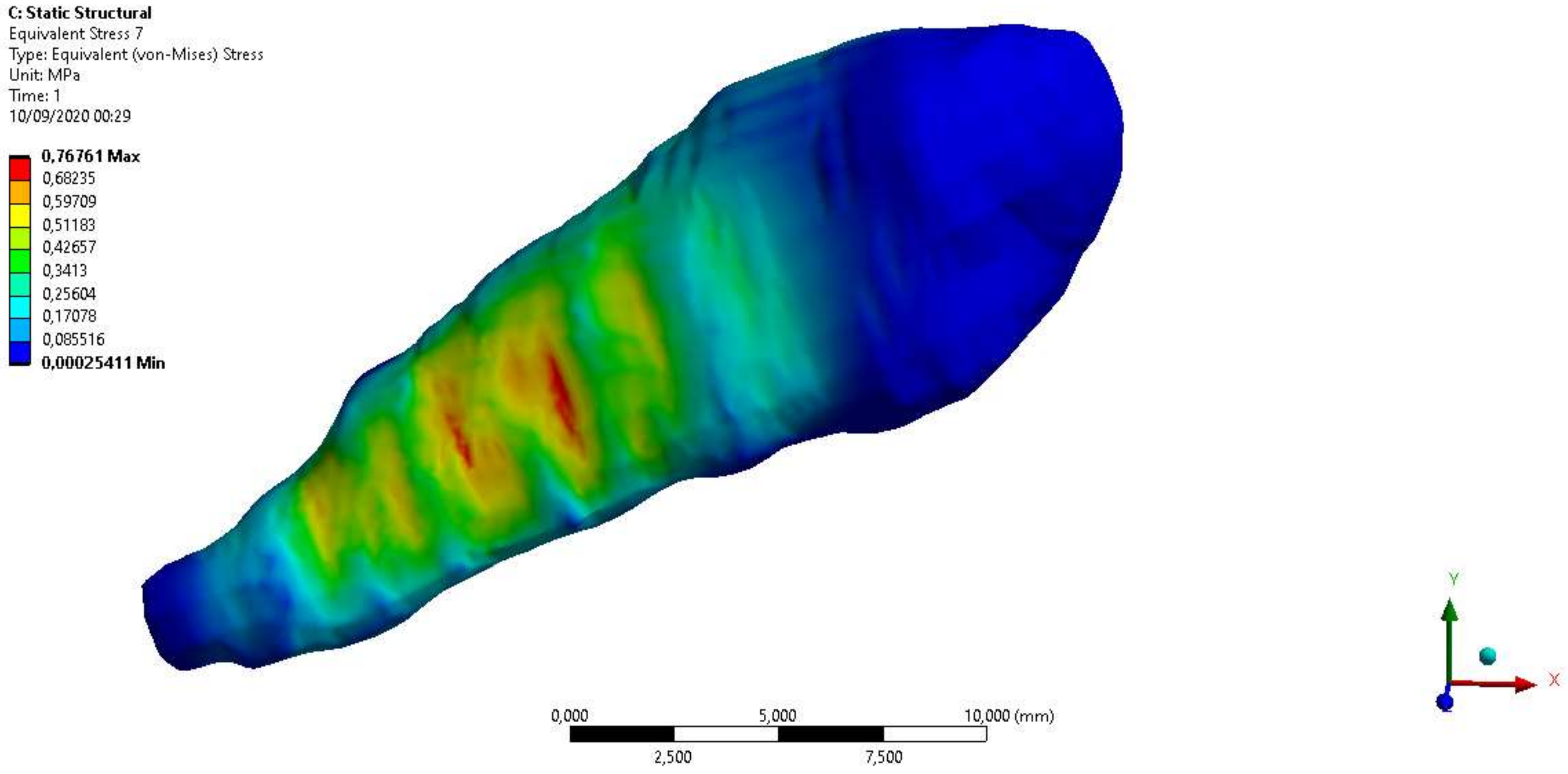
C: Static Structural
Equivalent Stress 7
Type: Equivalent (von-Mises) Stress
Unit: MPa
Time: 1
10/09/2020 00:29
0,76761 Max
0,68235
0,59709
0,51183
0,42657
0,3413
0,25604
0,17078
0,085516
0,00025411 Min
0,000
5,000
10,000 (mm)
2,500
7,500
Y
X

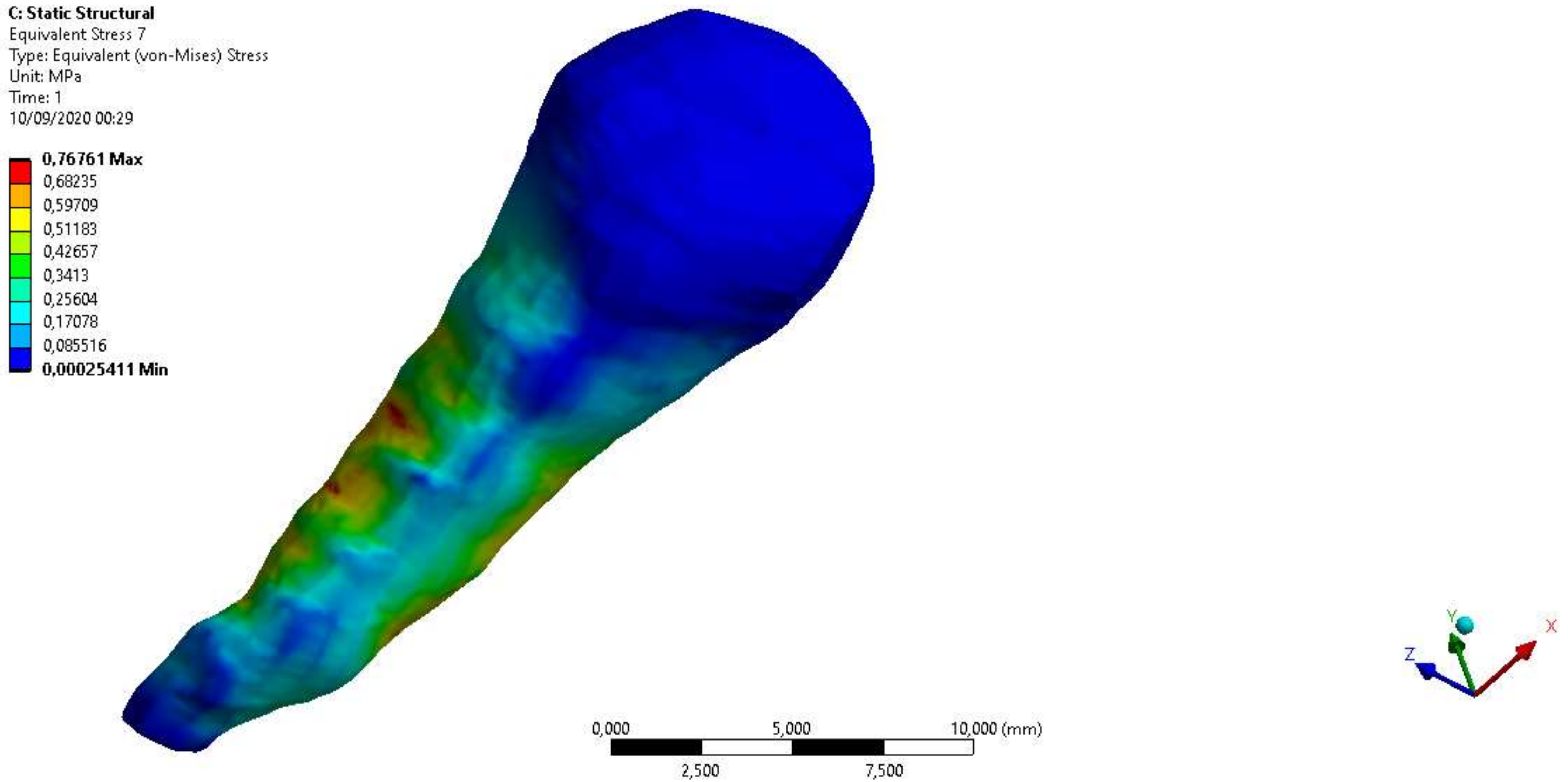
C: Static Structural
Equivalent Stress 7
Type: Equivalent (von-Mises) Stress
Unit: MPa
Time: 1
10/09/2020 00:29
0,76761 Max
0,68235
0,59709
0,51183
0,42657
0,3413
0,25604
0,17078
0,085516
0,00025411 Min
0,000
5,000
10,000 (mm)
2,500
7,500
Y
X
Z

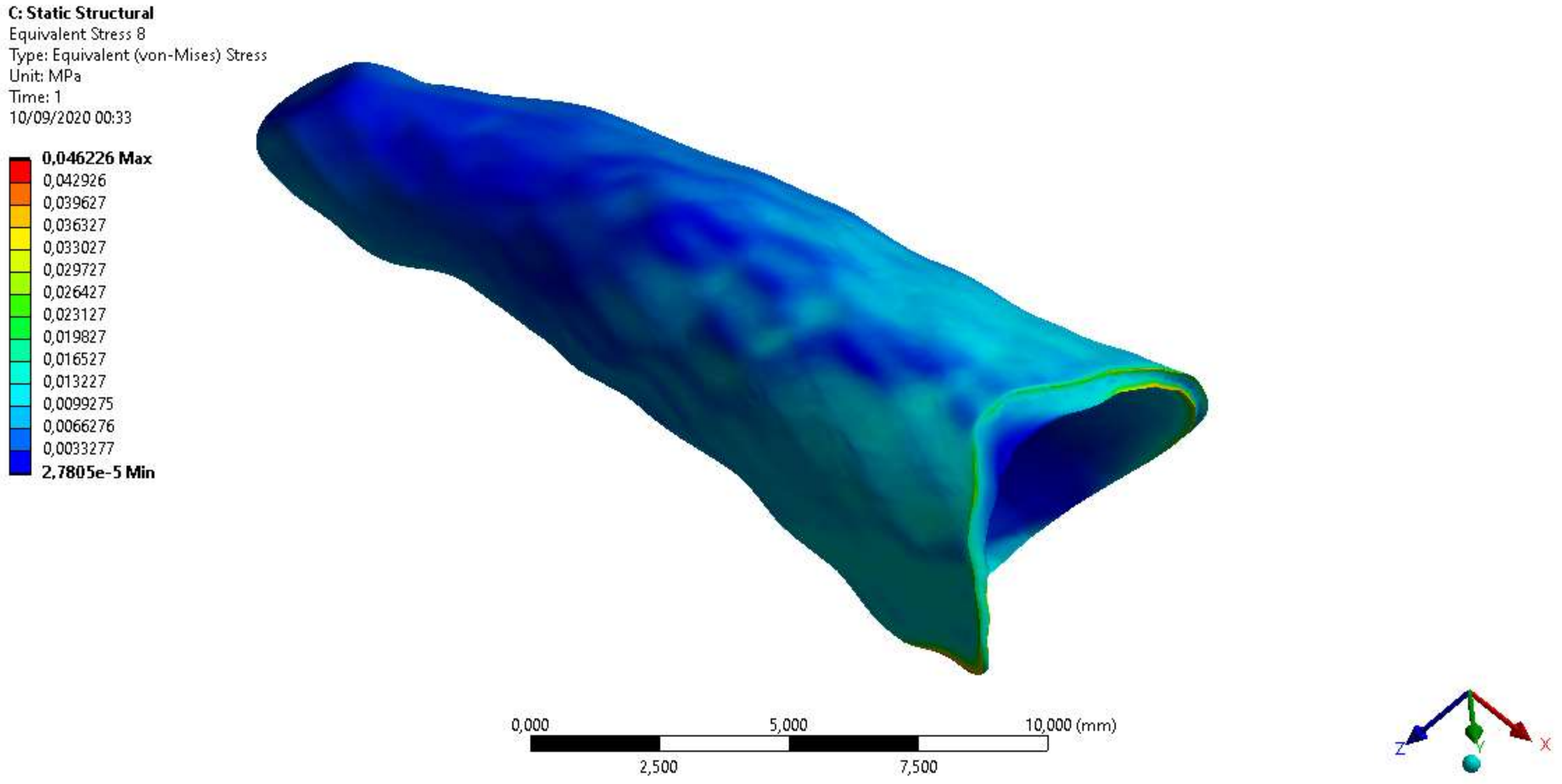
C: Static Structural
Equivalent Stress 8
Type: Equivalent (von-Mises) Stress
Unit: MPa
Time: 1
10/09/2020 00:33
0,046226 Max
0,042926
0,039627
0,036327
0,033027
0,029727
0,026427
0,023127
0,019827
0,016527
0,013227
0,0099275
0,0066276
0,0033277
2,7805e-5 Min
0,000
5,000
10,000 (mm)
2,500
7,500
Z
Y
X

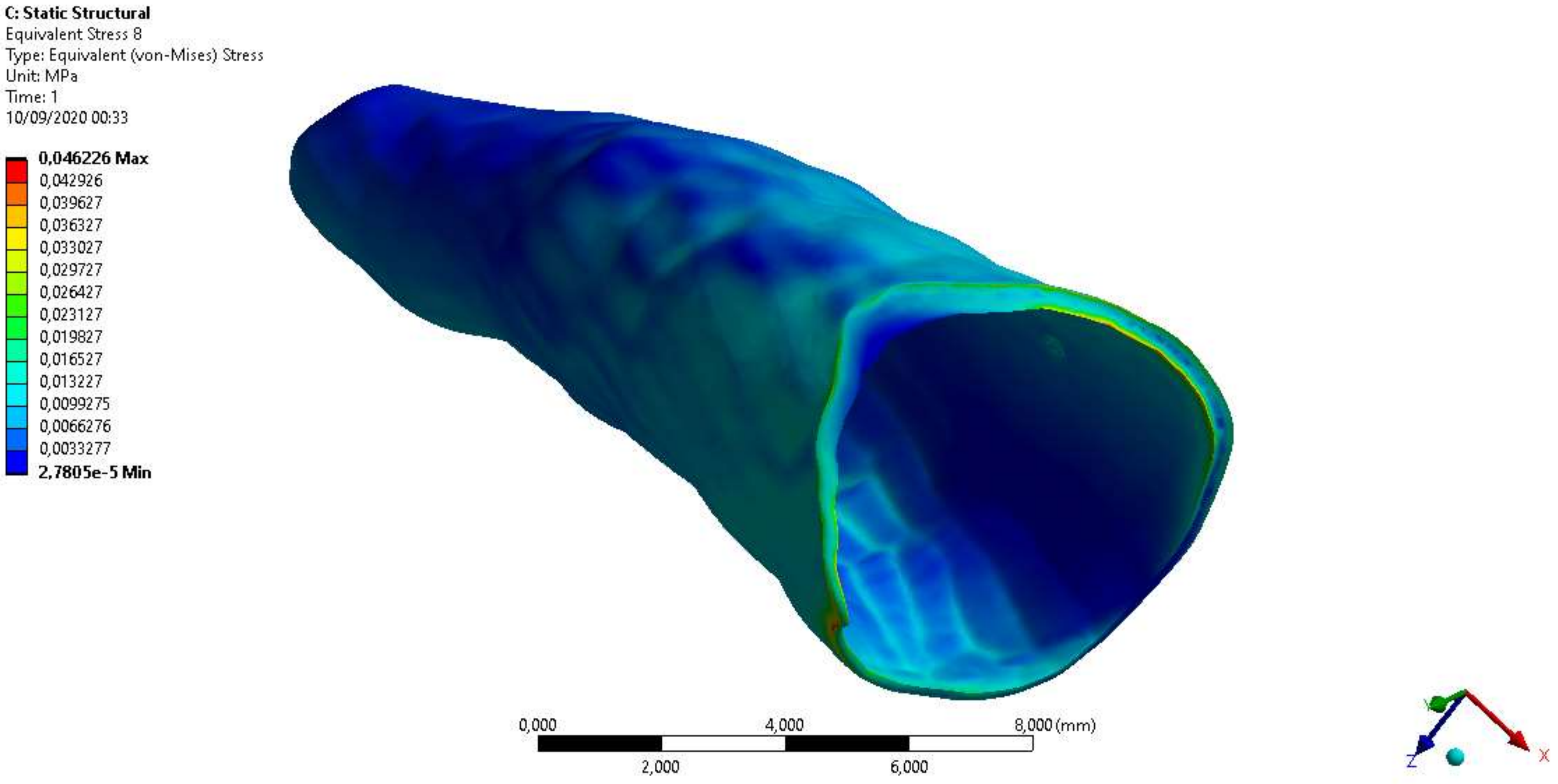
C: Static Structural
Equivalent Stress 8
Type: Equivalent (von-Mises) Stress
Unit: MPa
Time: 1
10/09/2020 00:33
0,046226 Max
0,042926
0,039627
0,036327
0,033027
0,029727
0,026427
0,023127
0,019827
0,016527
0,013227
0,0099275
0,0066276
0,0033277
2,7805e-5 Min
0,000
4,000
8,000 (mm)
2,000
6,000
Y
Z
X

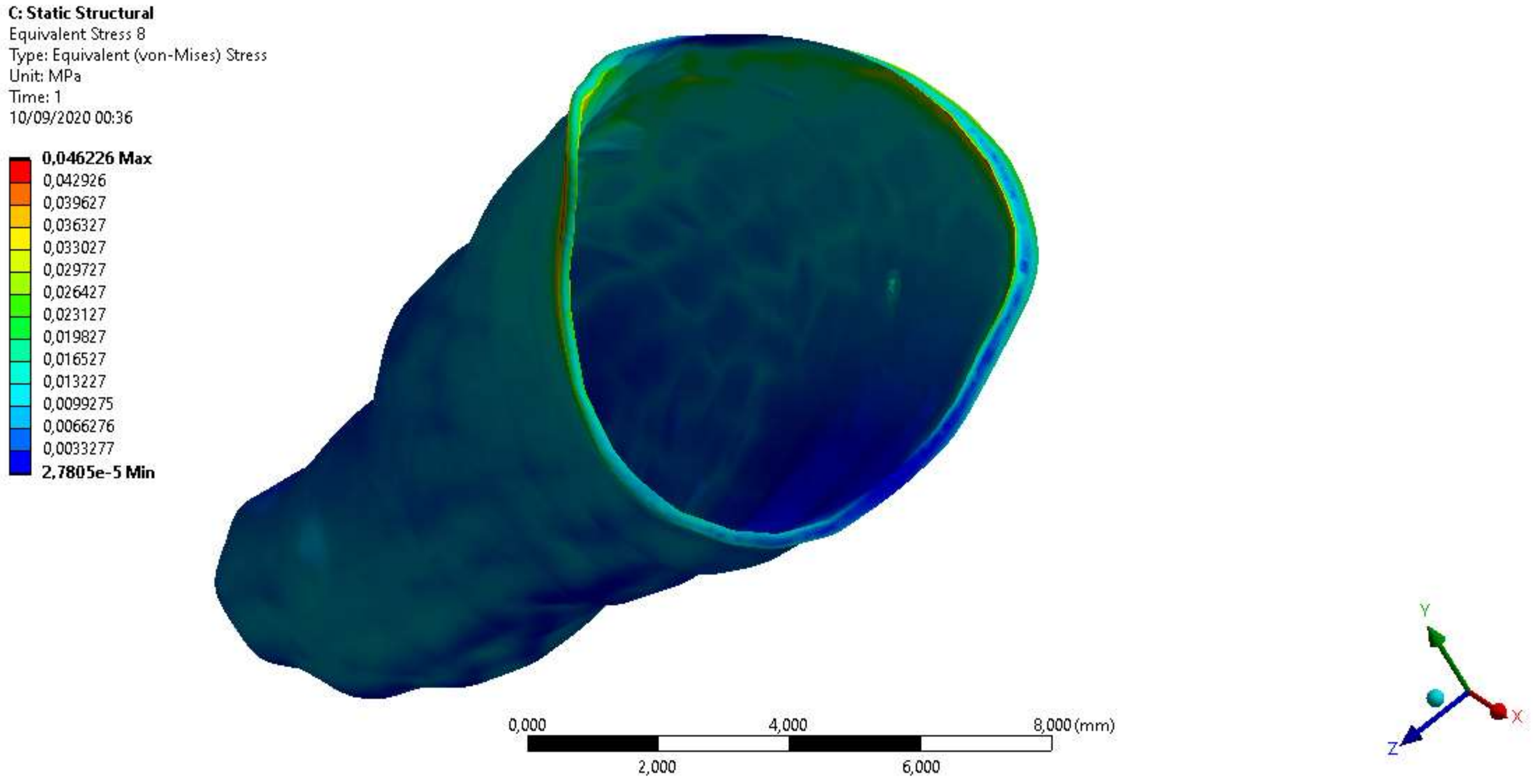
C: Static Structural
Equivalent Stress 8
Type: Equivalent (von-Mises) Stress
Unit: MPa
Time: 1
10/09/2020 00:36
0,046226 Max
0,042926
0,039627
0,036327
0,033027
0,029727
0,026427
0,023127
0,019827
0,016527
0,013227
0,0099275
0,0066276
0,0033277
2,7805e-5 Min
0,000
4,000
8,000 (mm)
2,000
6,000
Y
X
Z

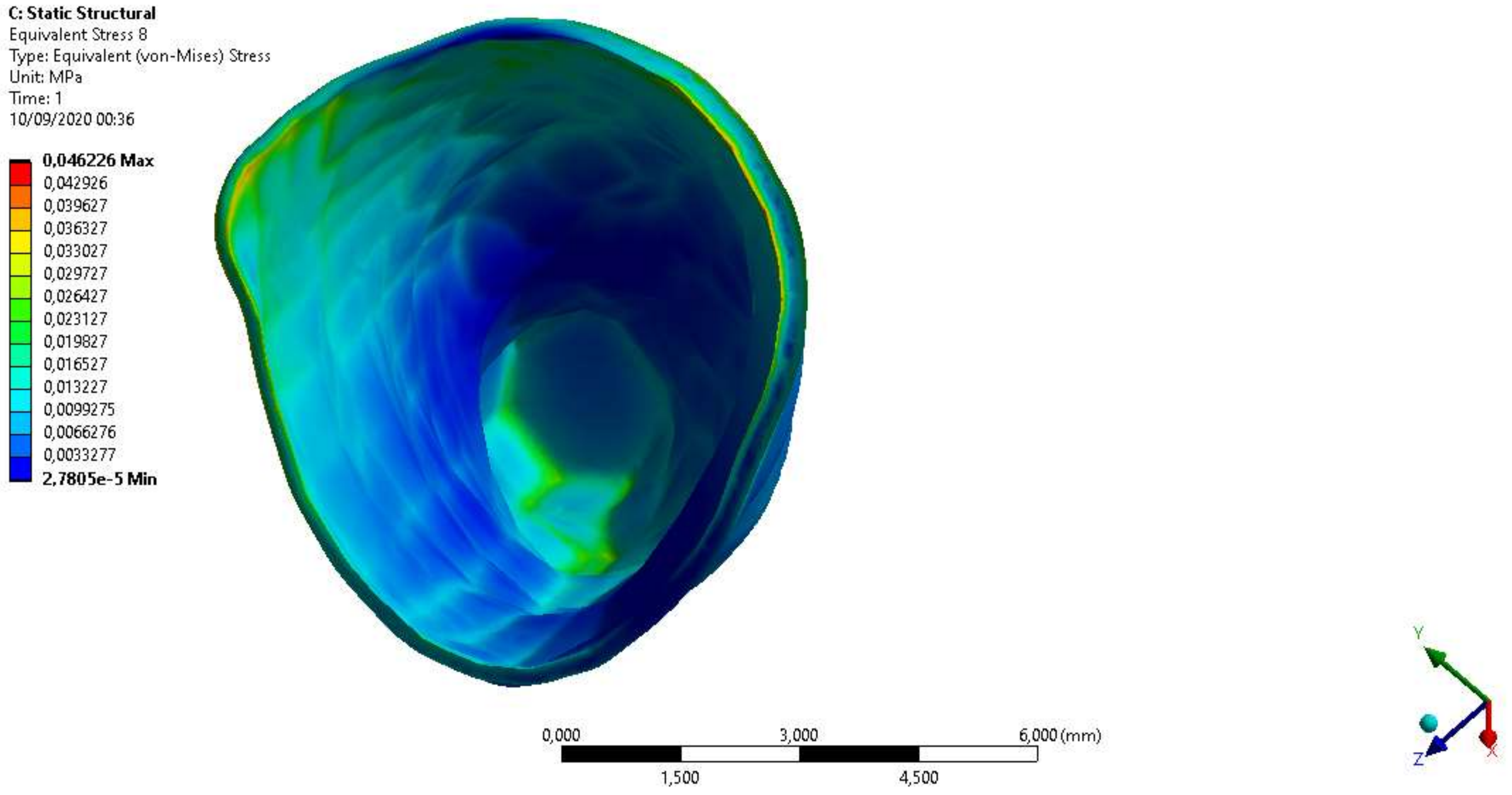
C: Static Structural
Equivalent Stress 8
Type: Equivalent (von-Mises) Stress
Unit: MPa
Time: 1
10/09/2020 00:36
0,046226 Max
0,042926
0,039627
0,036327
0,033027
0,029727
0,026427
0,023127
0,019827
0,016527
0,013227
0,0099275
0,0066276
0,0033277
2,7805e-5 Min
0,000
3,000
6,000 (mm)
1,500
4,500
Y
Z
X

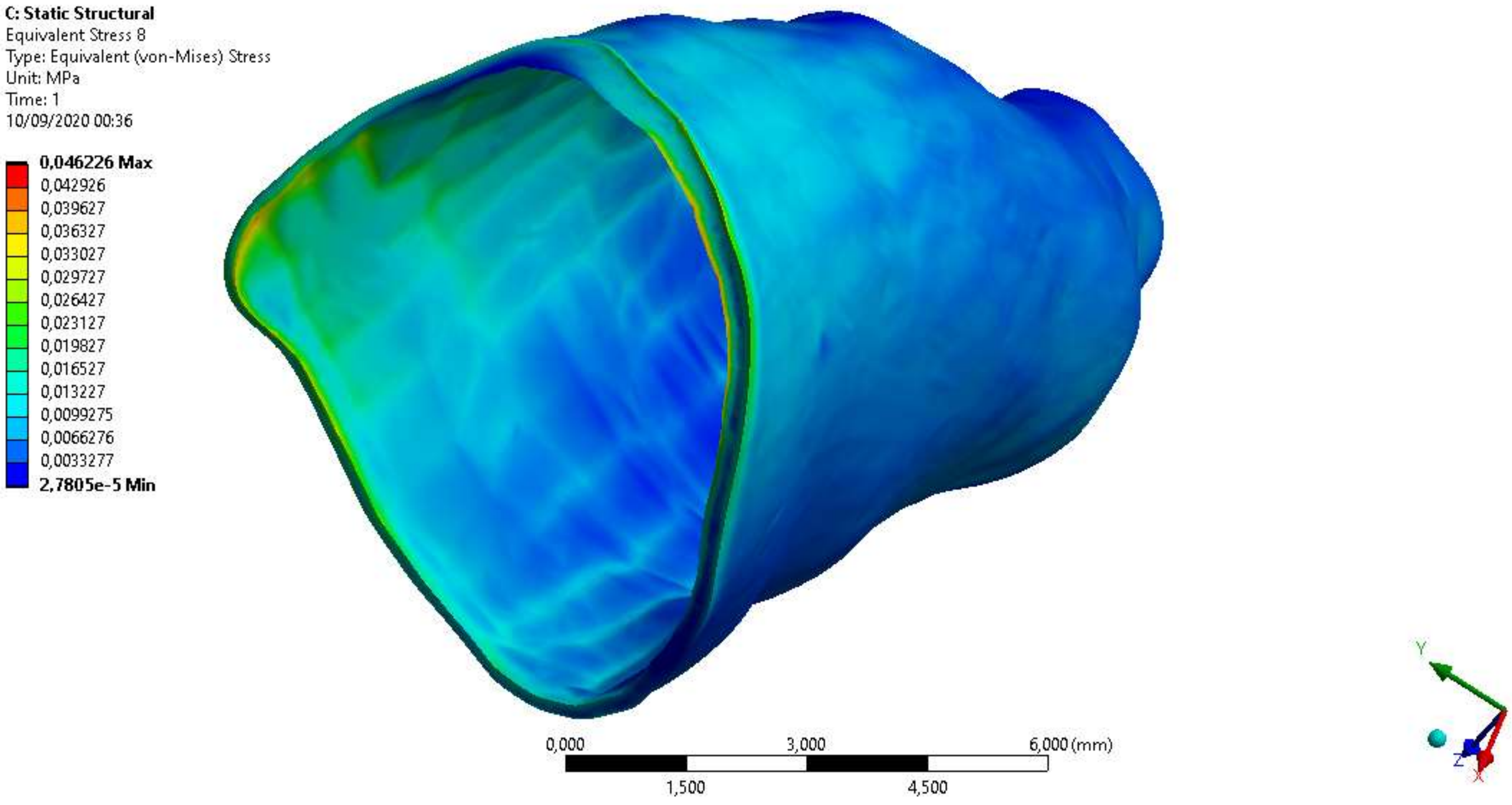
C: Static Structural
Equivalent Stress 8
Type: Equivalent (von-Mises) Stress
Unit: MPa
Time: 1
10/09/2020 00:36
0,046226 Max
0,042926
0,039627
0,036327
0,033027
0,029727
0,026427
0,023127
0,019827
0,016527
0,013227
0,0099275
0,0066276
0,0033277
2,7805e-5 Min
0,000
3,000
6,000 (mm)
1,500
4,500
Y
Z
X

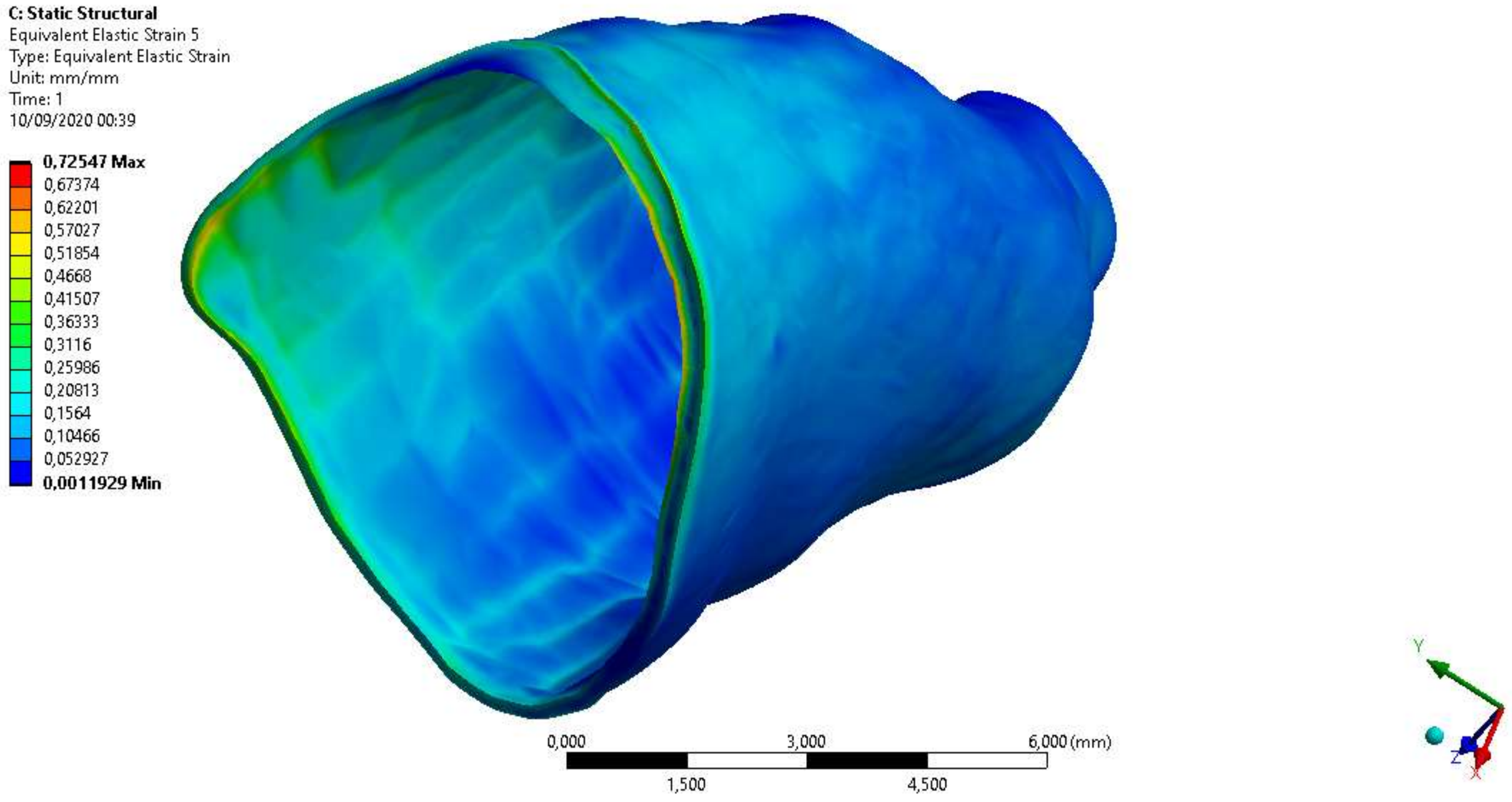
C: Static Structural
Equivalent Elastic Strain 5
Type: Equivalent Elastic Strain
Unit: mm/mm
Time: 1
10/09/2020 00:39
0,72547 Max
0,67374
0,62201
0,57027
0,51854
0,4668
0,41507
0,36333
0,3116
0,25986
0,20813
0,1564
0,10466
0,052927
0,0011929 Min
0,000
3,000
6,000 (mm)
1,500
4,500
Y
Z
X

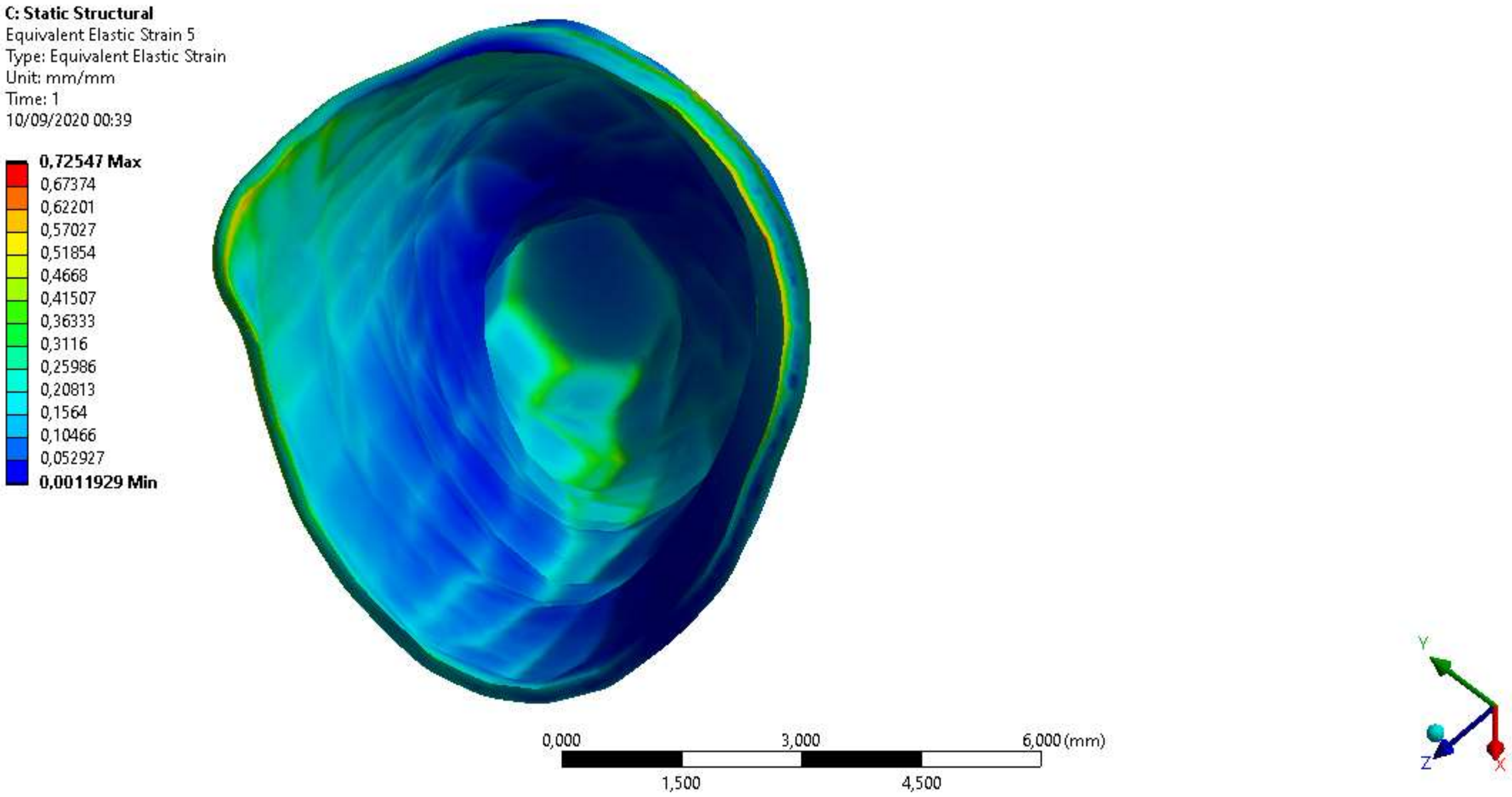
C: Static Structural
Equivalent Elastic Strain 5
Type: Equivalent Elastic Strain
Unit: mm/mm
Time: 1
10/09/2020 00:39
0,72547 Max
0,67374
0,62201
0,57027
0,51854
0,4668
0,41507
0,36333
0,3116
0,25986
0,20813
0,1564
0,10466
0,052927
0,0011929 Min
0,000
3,000
6,000 (mm)
1,500
4,500
Y
Z
X

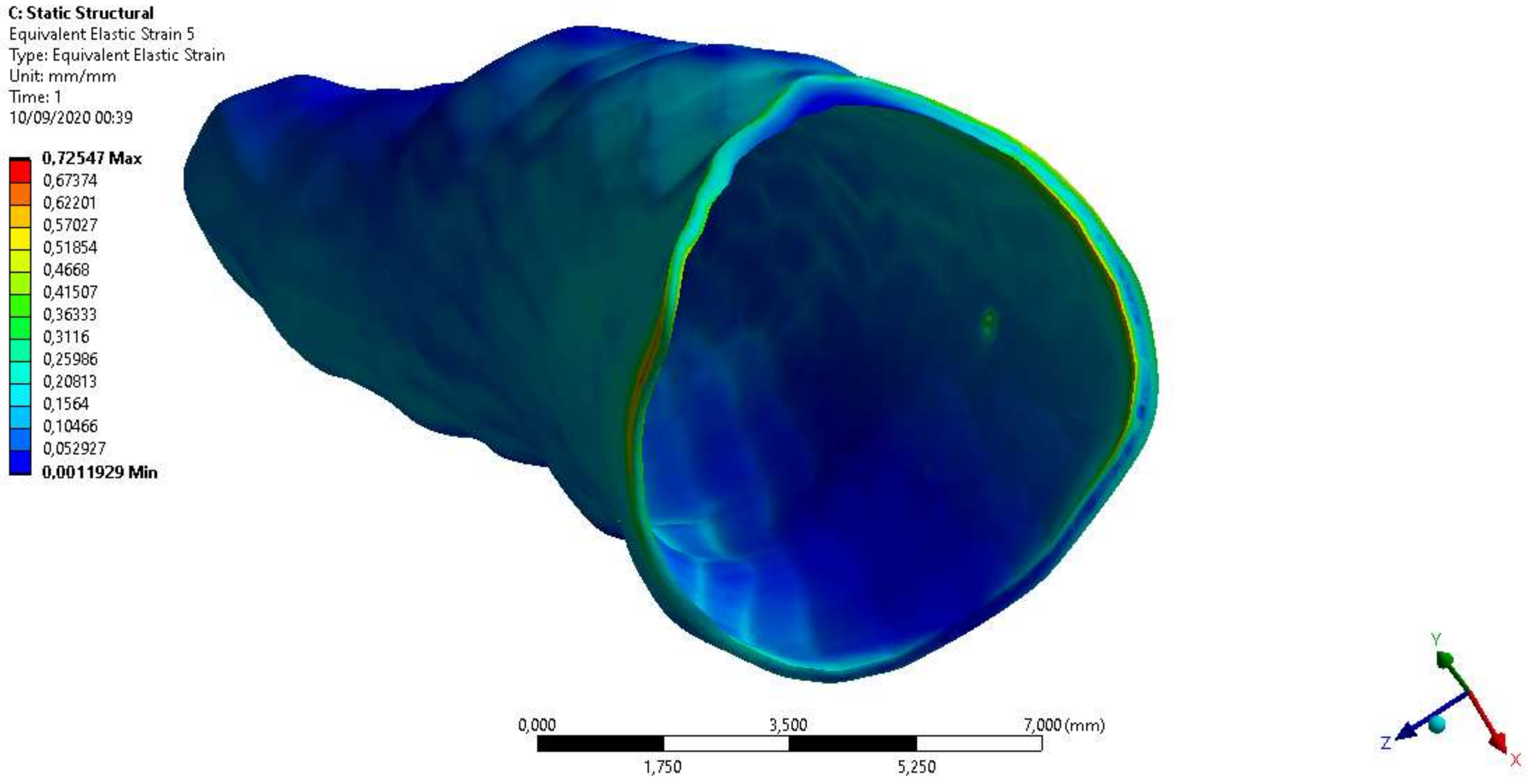
C: Static Structural
Equivalent Elastic Strain 5
Type: Equivalent Elastic Strain
Unit: mm/mm
Time: 1
10/09/2020 00:39
0,72547 Max
0,67374
0,62201
0,57027
0,51854
0,4668
0,41507
0,36333
0,3116
0,25986
0,20813
0,1564
0,10466
0,052927
0,0011929 Min
0,000
3,500
7,000 (mm)
1,750
5,250
Y
Z
X

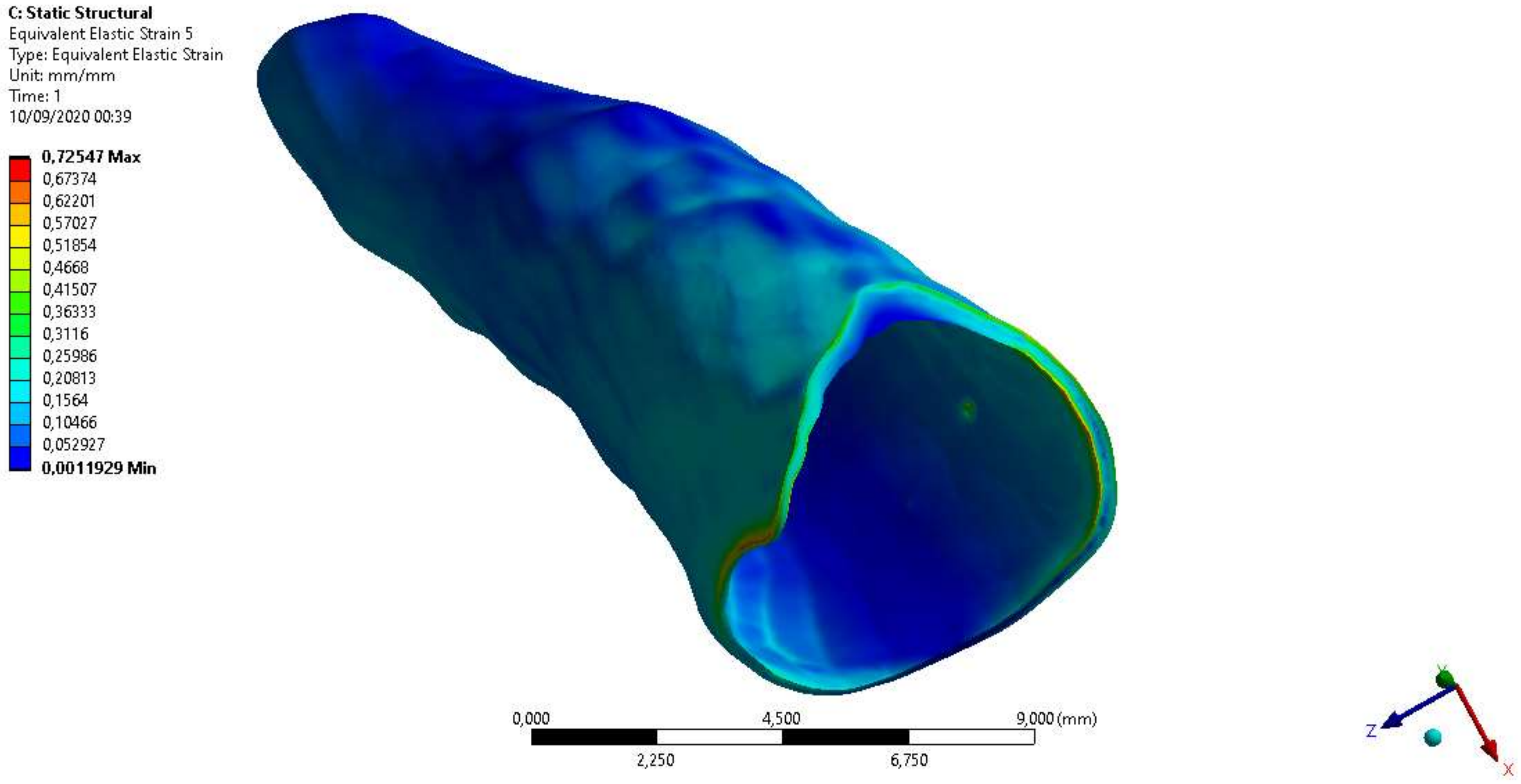
C: Static Structural
Equivalent Elastic Strain 5
Type: Equivalent Elastic Strain
Unit: mm/mm
Time: 1
10/09/2020 00:39
0,72547 Max
0,67374
0,62201
0,57027
0,51854
0,4668
0,41507
0,36333
0,3116
0,25986
0,20813
0,1564
0,10466
0,052927
0,0011929 Min
0,000
4,500
9,000 (mm)
2,250
6,750
Y
Z
X

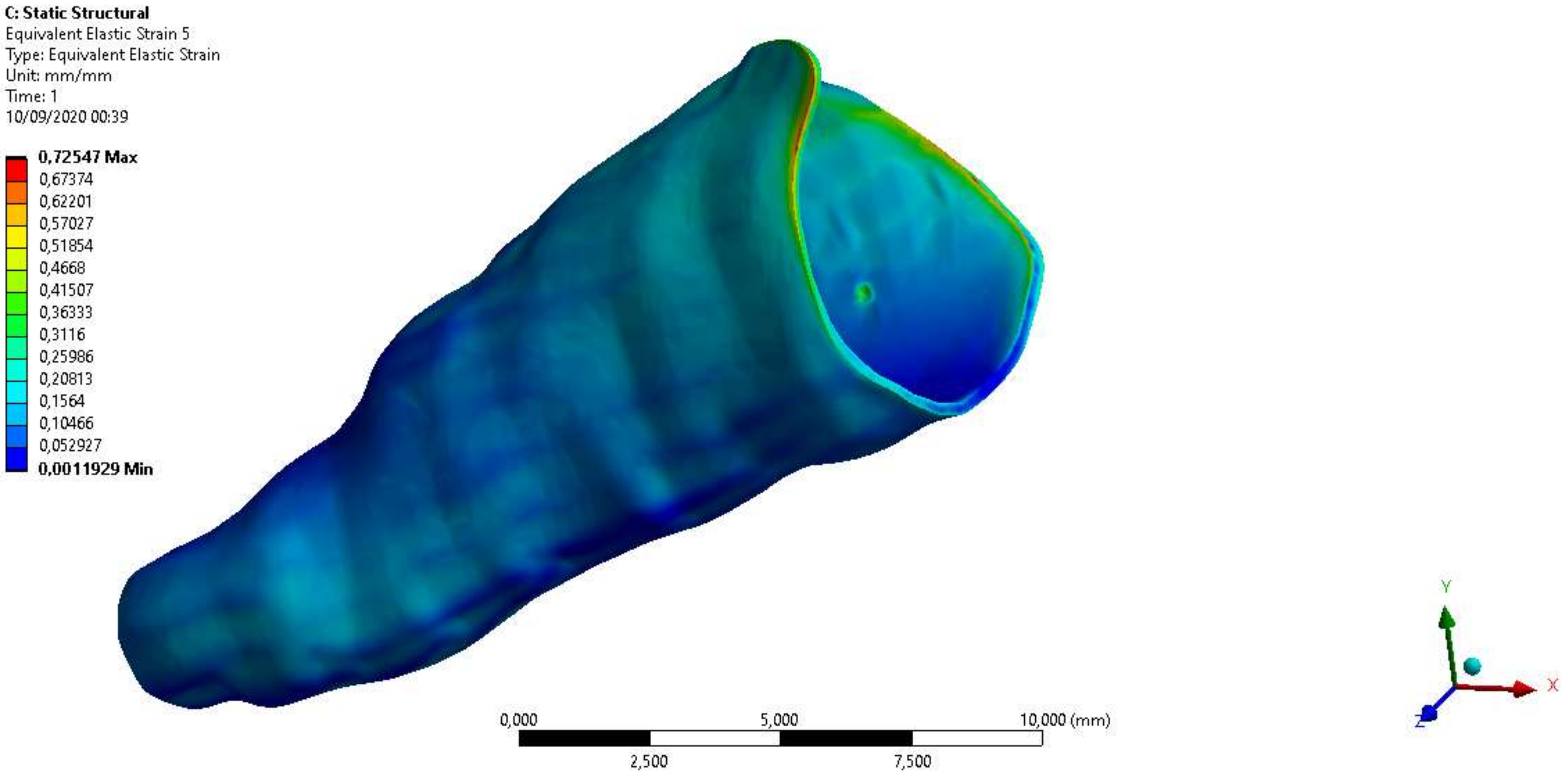
C: Static Structural
Equivalent Elastic Strain 5
Type: Equivalent Elastic Strain
Unit: mm/mm
Time: 1
10/09/2020 00:39
0,72547 Max
0,67374
0,62201
0,57027
0,51854
0,4668
0,41507
0,36333
0,3116
0,25986
0,20813
0,1564
0,10466
0,052927
0,0011929 Min
0,000
5,000
10,000 (mm)
2,500
7,500
Y
X
Z

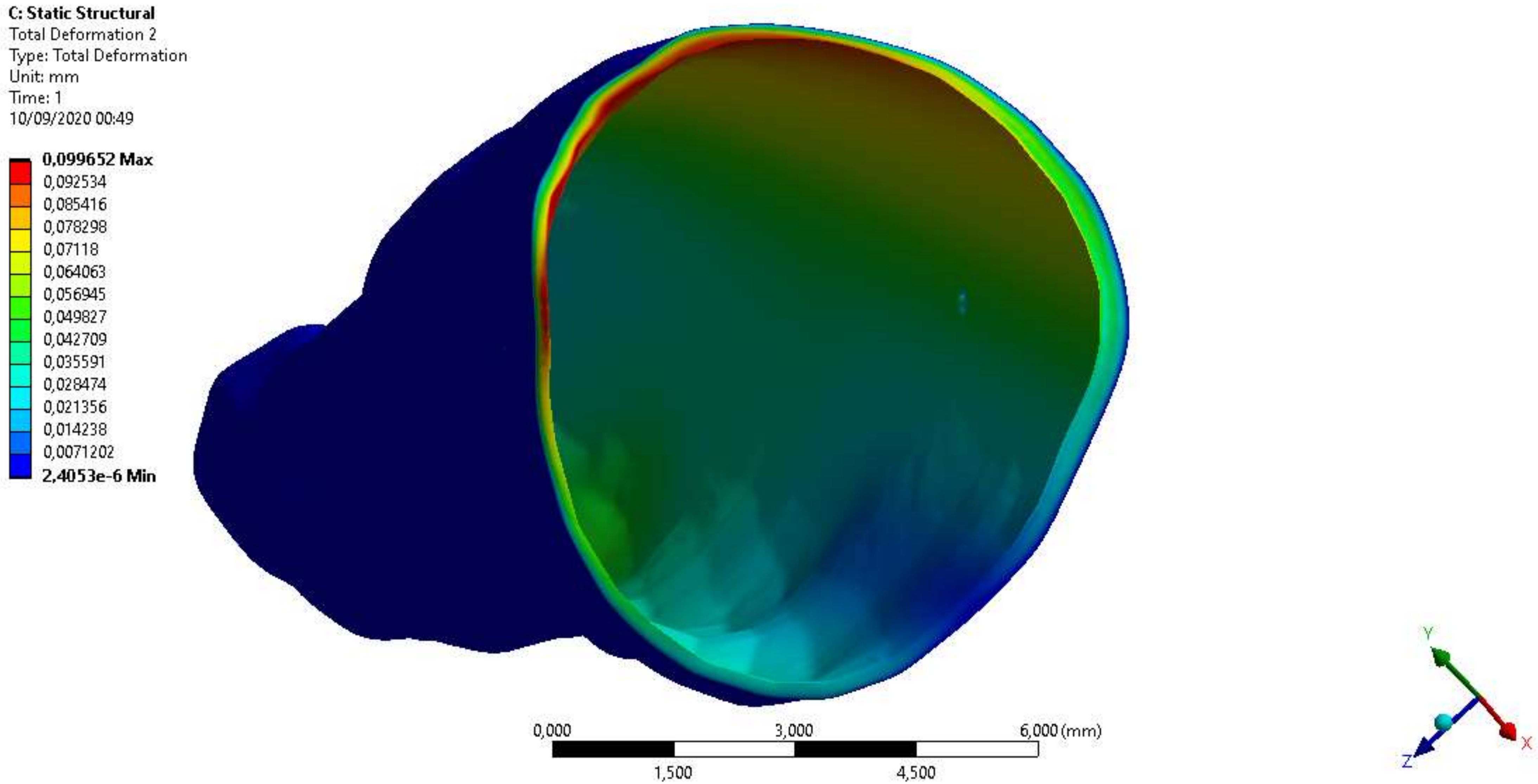

C: Static Structural
Total Deformation 2
Type: Total Deformation
Unit: mm
Time: 1
10/09/2020 00:49
0,099652 Max
0,092534
0,085416
0,078298
0,07118
0,064063
0,056945
0,049827
0,042709
0,035591
0,028474
0,021356
0,014238
0,0071202
2,4053e-6 Min
0,000
3,000
6,000 (mm)
1,500
4,500
Y
X
Z

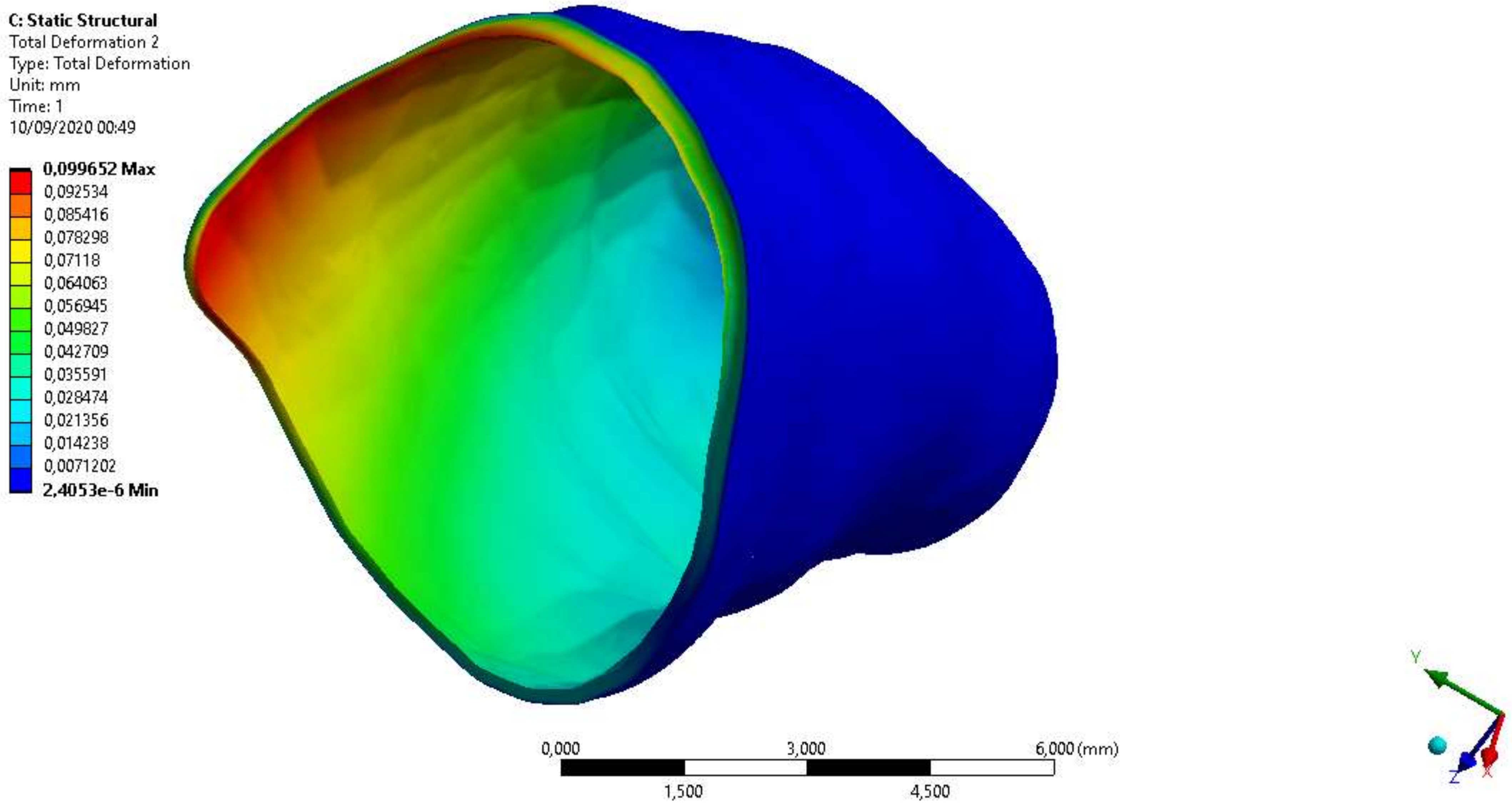
C: Static Structural
Total Deformation 2
Type: Total Deformation
Unit: mm
Time: 1
10/09/2020 00:49
0,099652 Max
0,092534
0,085416
0,078298
0,07118
0,064063
0,056945
0,049827
0,042709
0,035591
0,028474
0,021356
0,014238
0,0071202
2,4053e-6 Min
0,000
3,000
6,000 (mm)
1,500
4,500
Y
Z
X

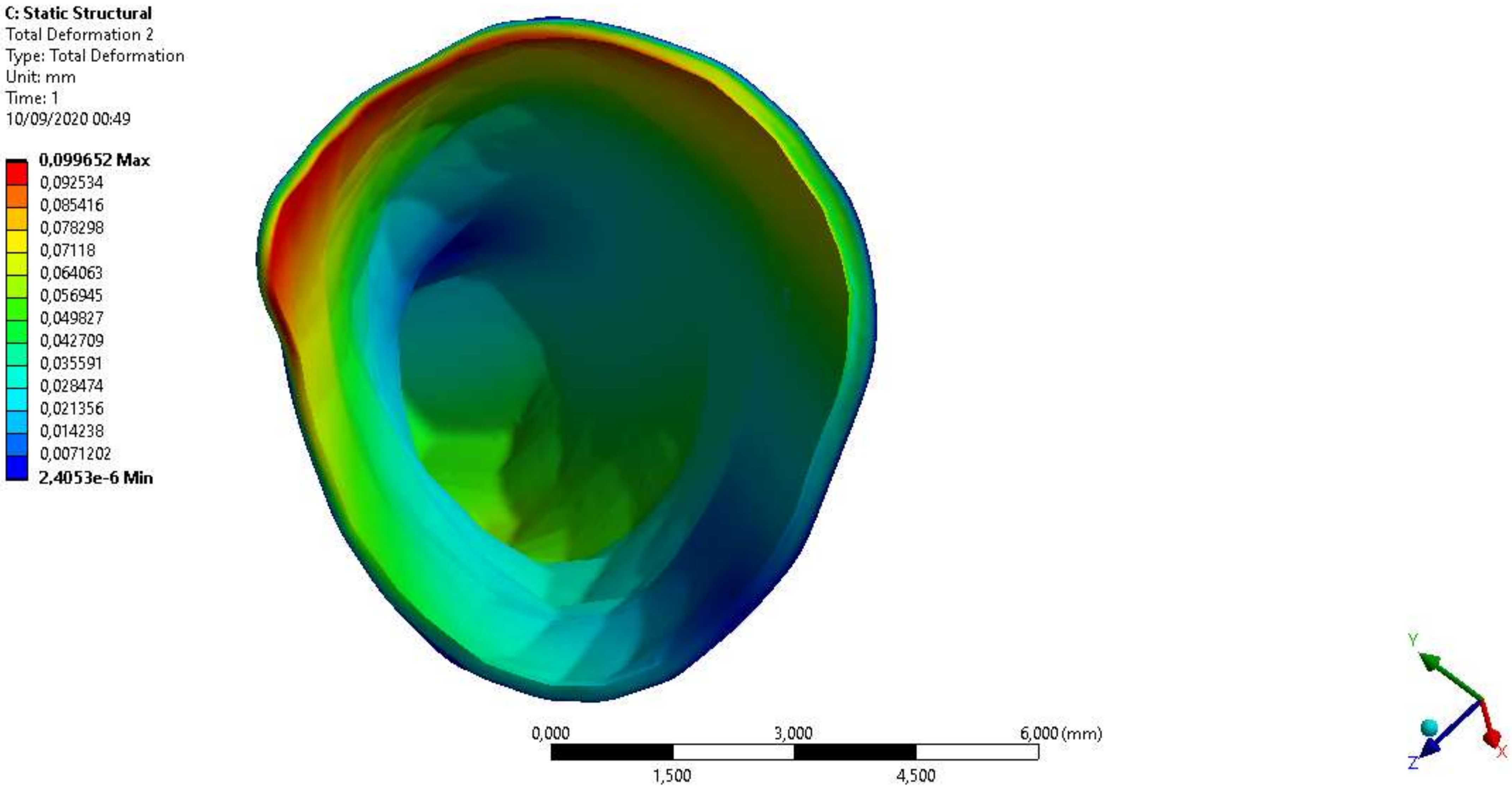
C: Static Structural
Total Deformation 2
Type: Total Deformation
Unit: mm
Time: 1
10/09/2020 00:49
0,099652 Max
0,092534
0,085416
0,078298
0,07118
0,064063
0,056945
0,049827
0,042709
0,035591
0,028474
0,021356
0,014238
0,0071202
2,4053e-6 Min
0,000
3,000
6,000 (mm)
1,500
4,500
Y
Z
X

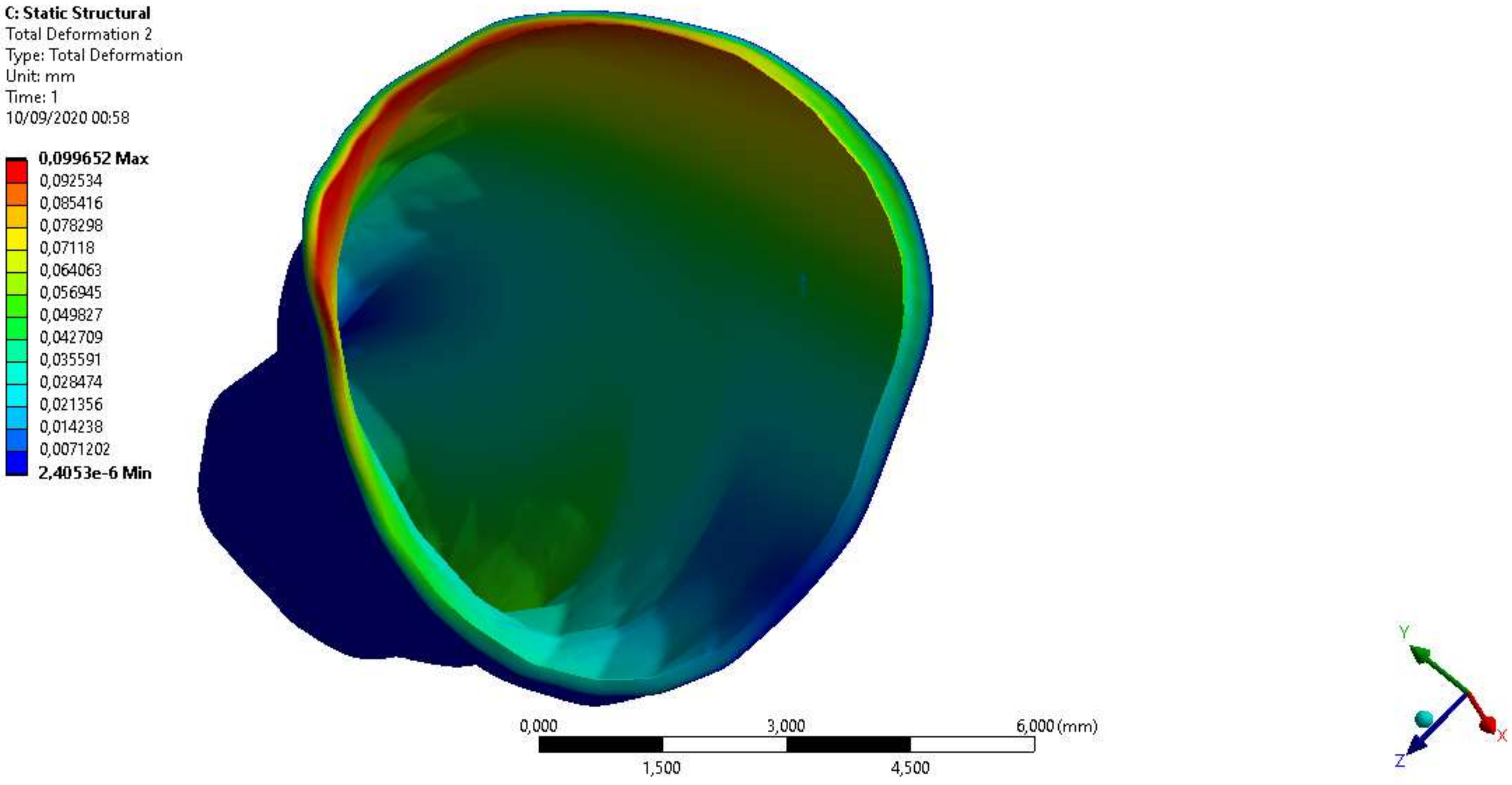
C: Static Structural
Total Deformation 2
Type: Total Deformation
Unit: mm
Time: 1
10/09/2020 00:58
0,099652 Max
0,092534
0,085416
0,078298
0,07118
0,064063
0,056945
0,049827
0,042709
0,035591
0,028474
0,021356
0,014238
0,0071202
2,4053e-6 Min
0,000
3,000
6,000 (mm)
1,500
4,500
Y
X
Z

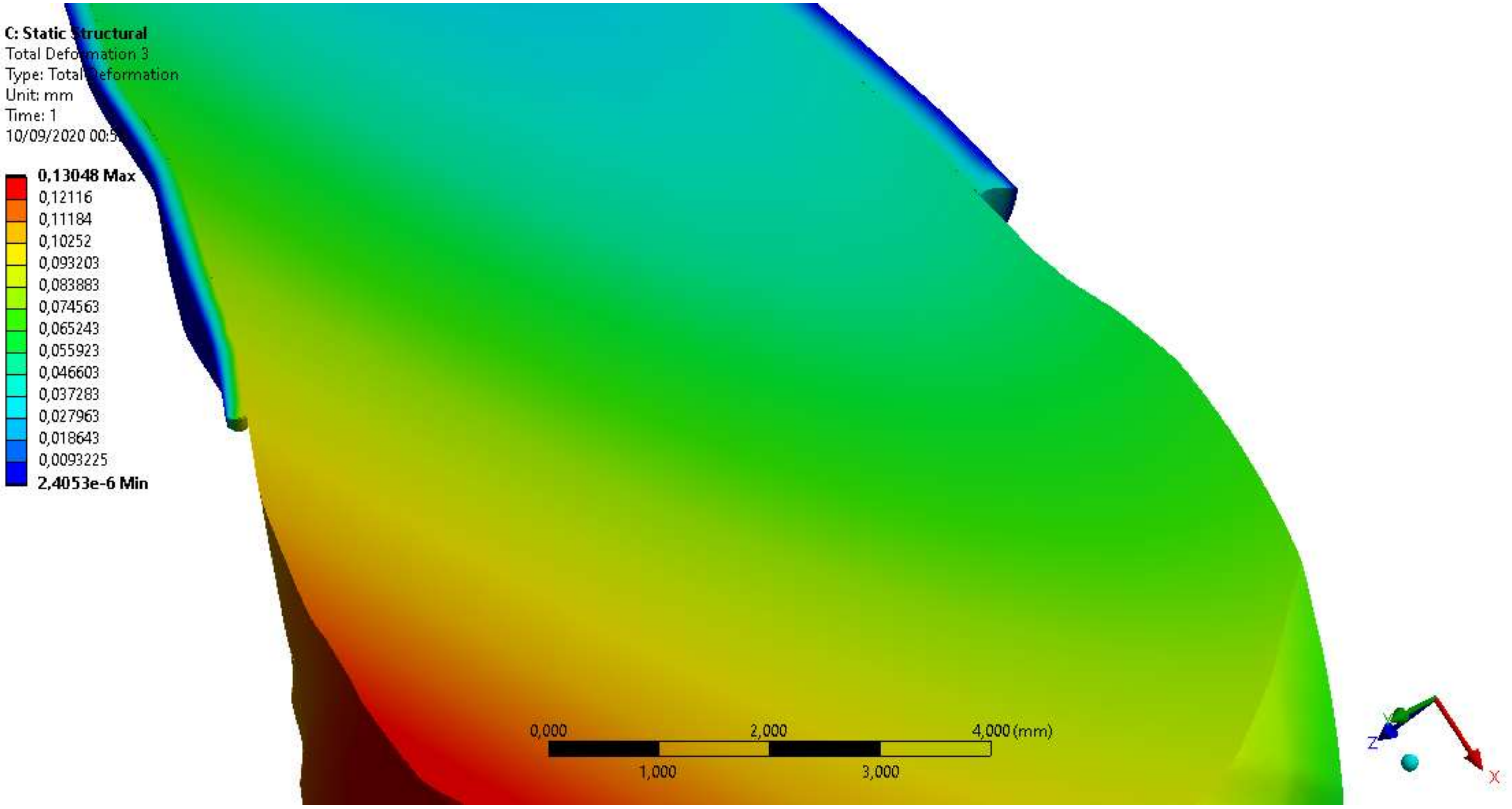
C: Static Structural
Total Deformation 3
Type: Total Deformation
Unit: mm
Time: 1
0,13048 Max
0,12116
0,11184
0,10252
0,093203
0,083883
0,074563
0,065243
0,055923
0,046603
0,037283
0,027963
0,018643
0,0093225
2,4053e-6 Min
0,000
2,000
4,000 (mm)
1,000
3,000
Z
X

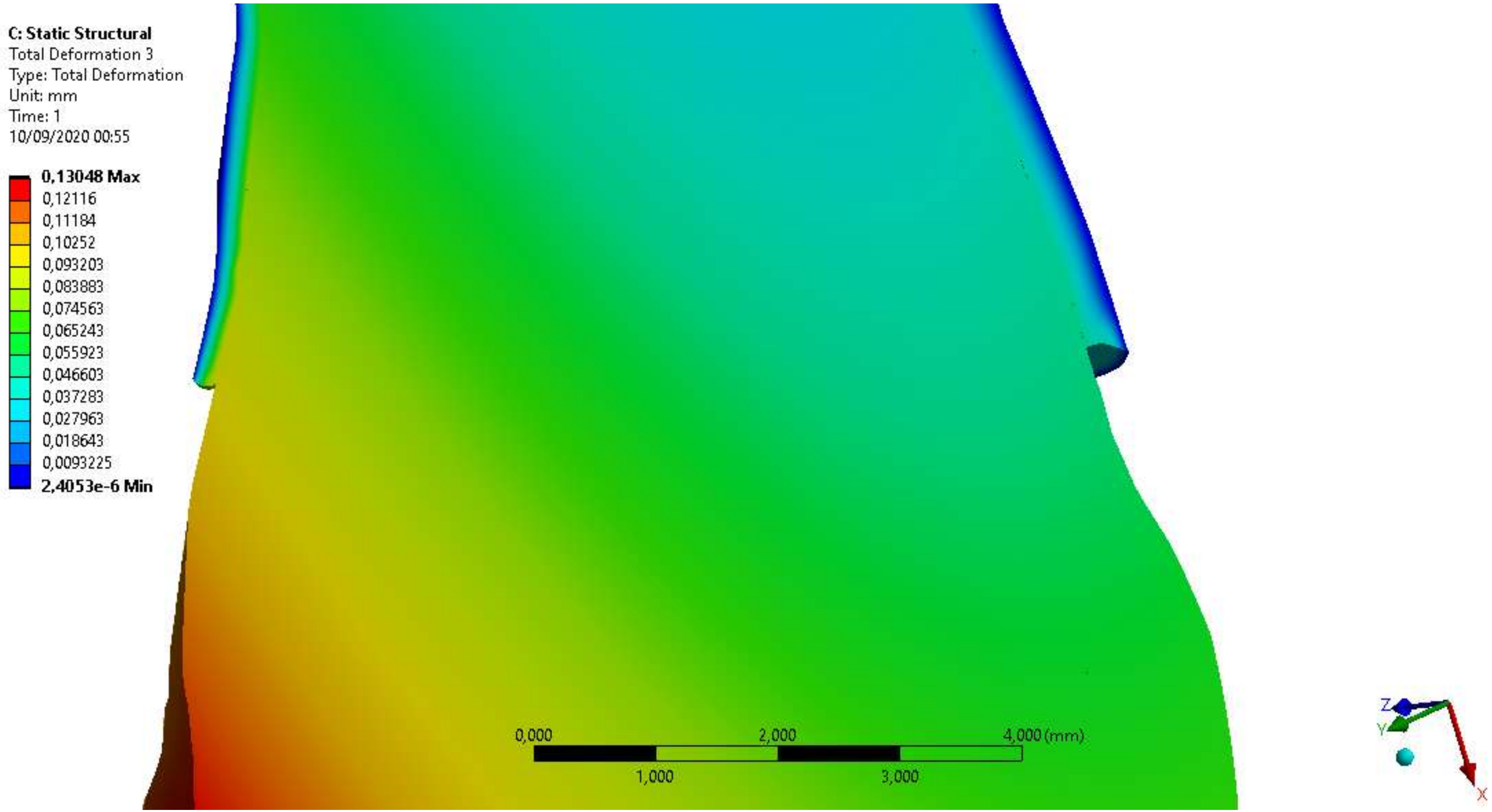
C: Static Structural
Total Deformation 3
Type: Total Deformation
Unit: mm
Time: 1
10/09/2020 00:55
0,13048 Max
0,12116
0,11184
0,10252
0,093203
0,083883
0,074563
0,065243
0,055923
0,046603
0,037283
0,027963
0,018643
0,0093225
2,4053e-6 Min
0,000
2,000
4,000 (mm)
1,000
3,000
Z
Y
X

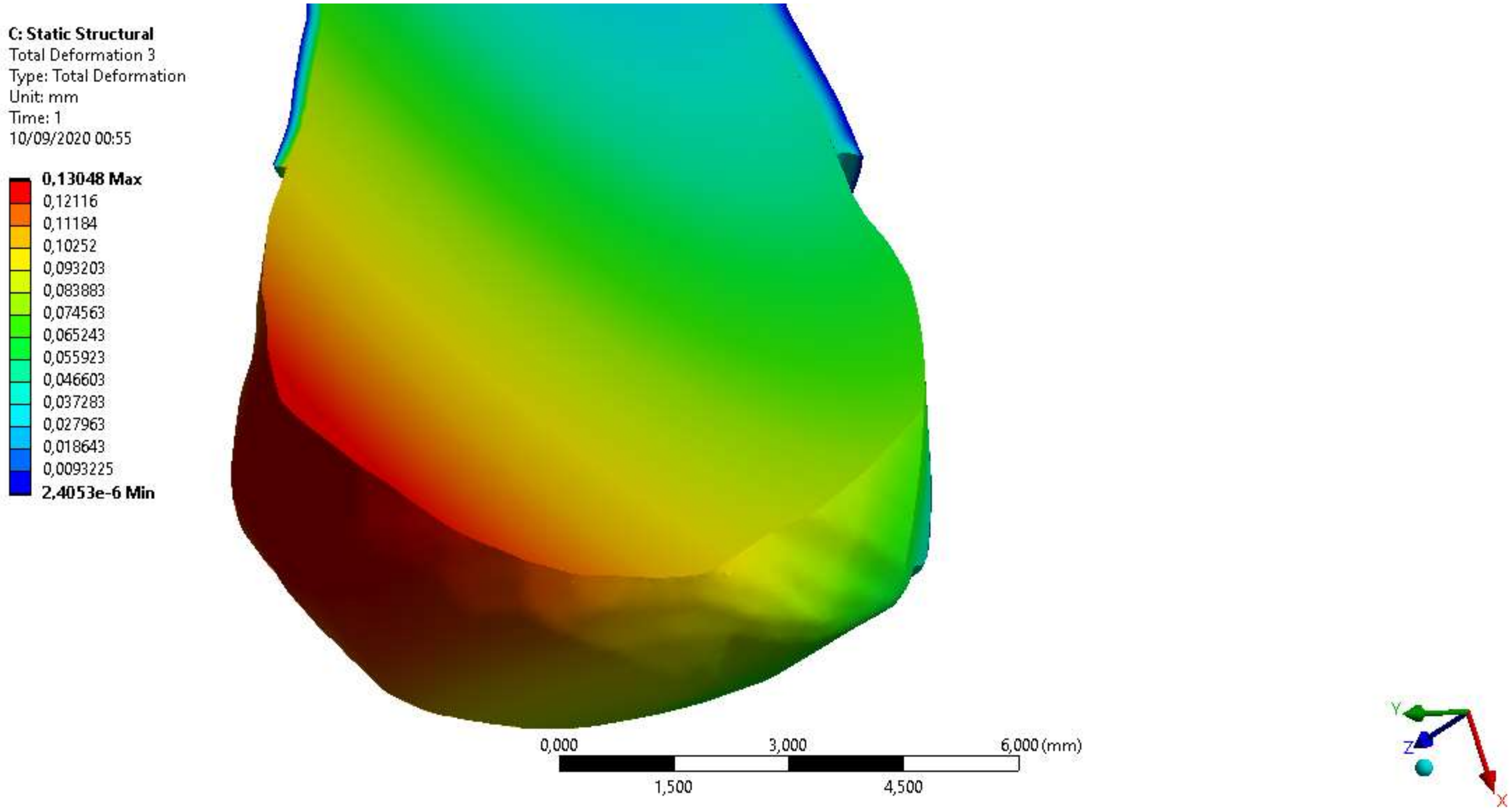
C: Static Structural
Total Deformation 3
Type: Total Deformation
Unit: mm
Time: 1
10/09/2020 00:55
0,13048 Max
0,12116
0,11184
0,10252
0,093203
0,083883
0,074563
0,065243
0,055923
0,046603
0,037283
0,027963
0,018643
0,0093225
2,4053e-6 Min
0,000
3,000
6,000 (mm)
1,500
4,500
Y
Z
X
