# Supplementary material for: Micro-osteoperforation for enhancement of orthodontic movement: A mechanical analysis using the finite element method
Source: PLoS One. 2024 Aug 19;19(8):e0308739. doi: 10.1371/journal.pone.0308739 (PMC11332926; doi:10.1371/journal.pone.0308739)
Supplement: S8 File — (PDF) [file pone.0308739.s008.pdf]

S8. Analysis 4 with perforations with moment

Dente

Fatigue Data at zero mean stress comes from 1998 ASME BPV Code, Section 8, Div 2, Table 5-110.1

Density	1,96e-06 kg/mm³

Structural

Isotropic Elasticity

Derive from	Young's Modulus and Poisson's Ratio
Young's Modulus	14700 MPa
Poisson's Ratio	0,31000
Bulk Modulus	12895 MPa
Shear Modulus	5610,7 MPa
Isotropic Secant Coefficient of Thermal Expansion	1,2e-05 1/°C
Compressive Ultimate Strength	0 MPa
Compressive Yield Strength	250,00 MPa

Osso Medular

Density	4,1e-07 kg/mm³

Structural

Isotropic Elasticity

Derive from	Young's Modulus and Poisson's Ratio
Young's Modulus	1370,0 MPa
Poisson's Ratio	0,30000
Bulk Modulus	1141,7 MPa
Shear Modulus	526,92 MPa

Osso Cortical Isotropico

Density	1,99e-06 kg/mm³

Structural

Isotropic Elasticity

Derive from	Young's Modulus and Poisson's Ratio
Young's Modulus	13700 MPa
Poisson's Ratio	0,30000
Bulk Modulus	11417 MPa
Shear Modulus	5269,2 MPa

LigamentoPeriodotal

Fatigue Data at zero mean stress comes from 1998 ASME BPV Code, Section 8, Div 2, Table 5-110.1

Density	1,2e-06 kg/mm³

Structural

Isotropic Elasticity

Derive from	Young's Modulus and Poisson's Ratio
Young's Modulus	0,068000 MPa
Poisson's Ratio	0,45000
Bulk Modulus	0,22667 MPa
Shear Modulus	0,023448 MPa

Tecido Granulomatoso

Density	4,1e-07 kg/mm^3

Structural

Isotropic Elasticity

Derive from	Young's Modulus and Poisson's Ratio
Young's Modulus	1,0000 MPa
Poisson's Ratio	0,49000
Bulk Modulus	16,667 MPa
Shear Modulus	0,33557 MPa

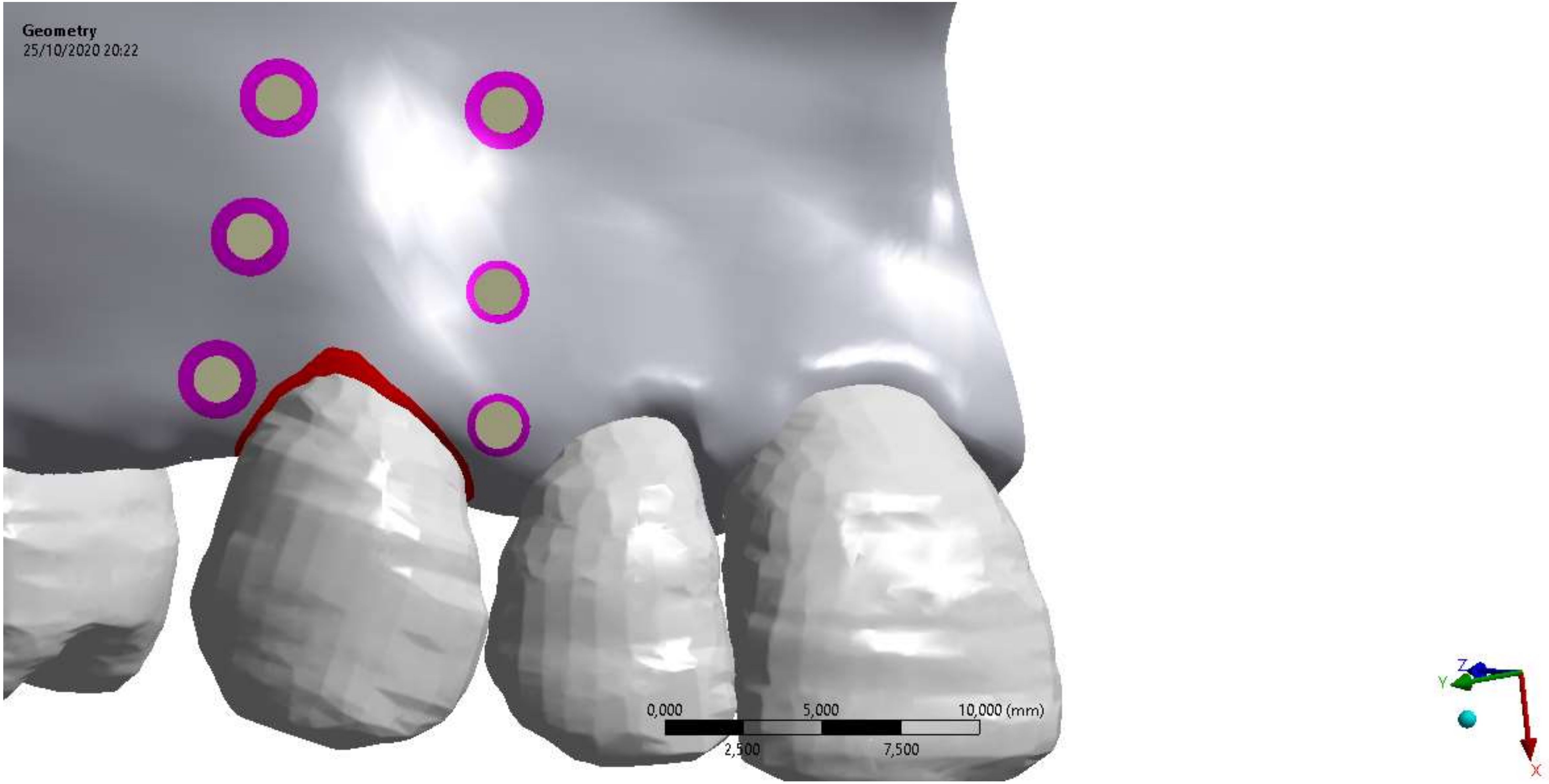
Geometry
25/10/2020 20:22
0,000
5,000
10,000 (mm)
2,500
7,500
Z
Y
X

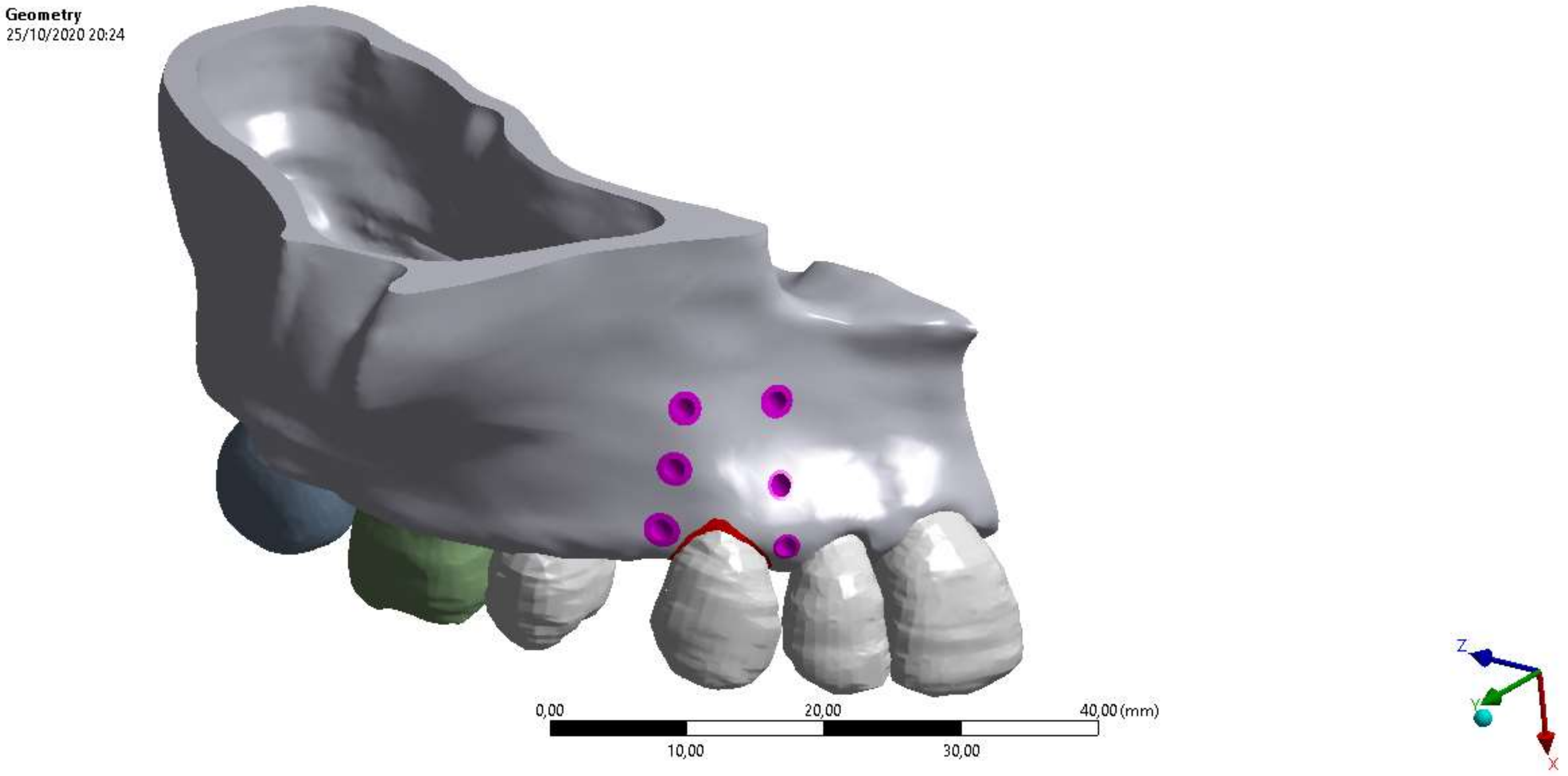
Geometry
25/10/2020 20:24
0,00
20,00
40,00 (mm)
10,00
30,00
Z
Y
X

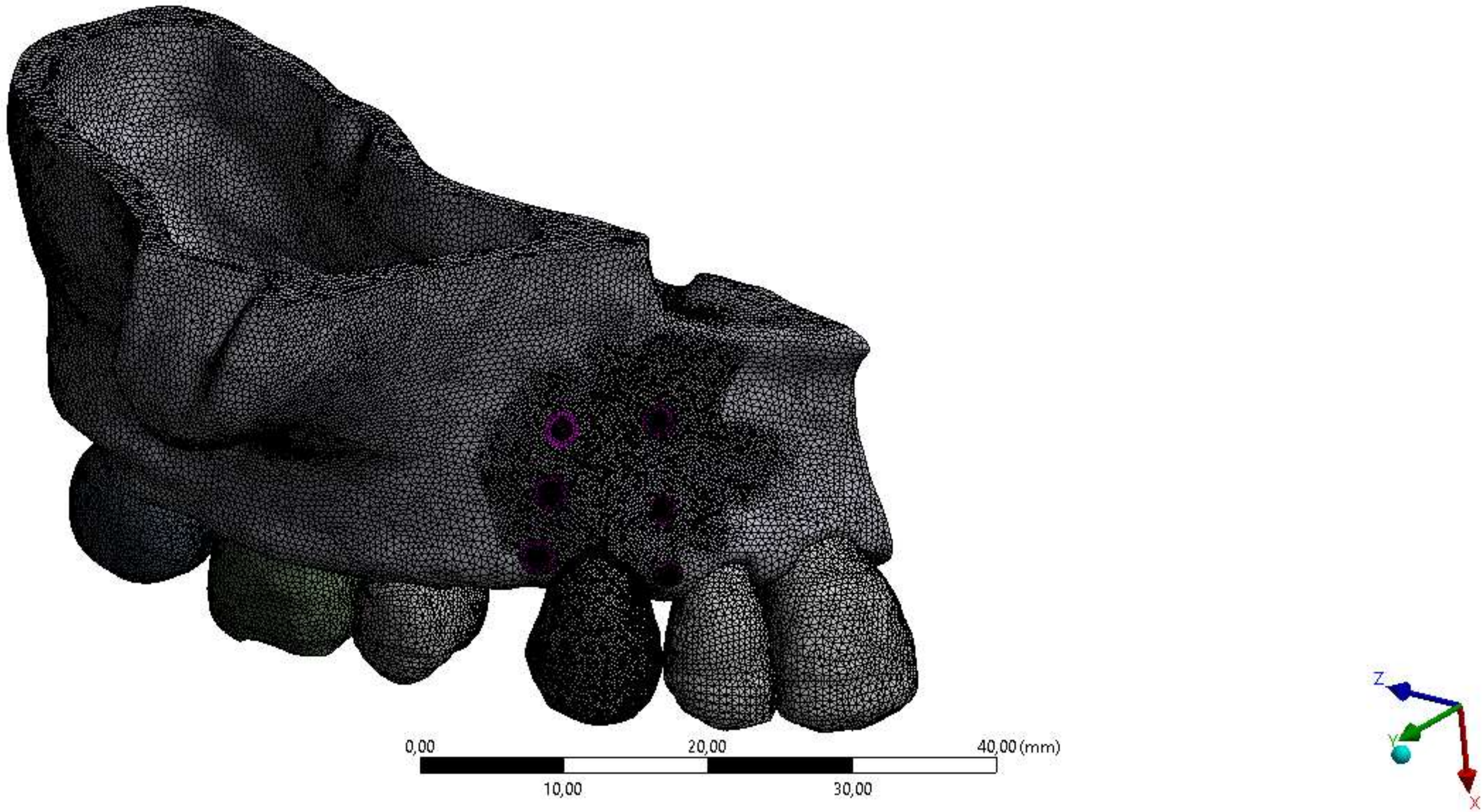

0,00
20,00
40,00 (mm)
10,00
30,00
Z
Y
X

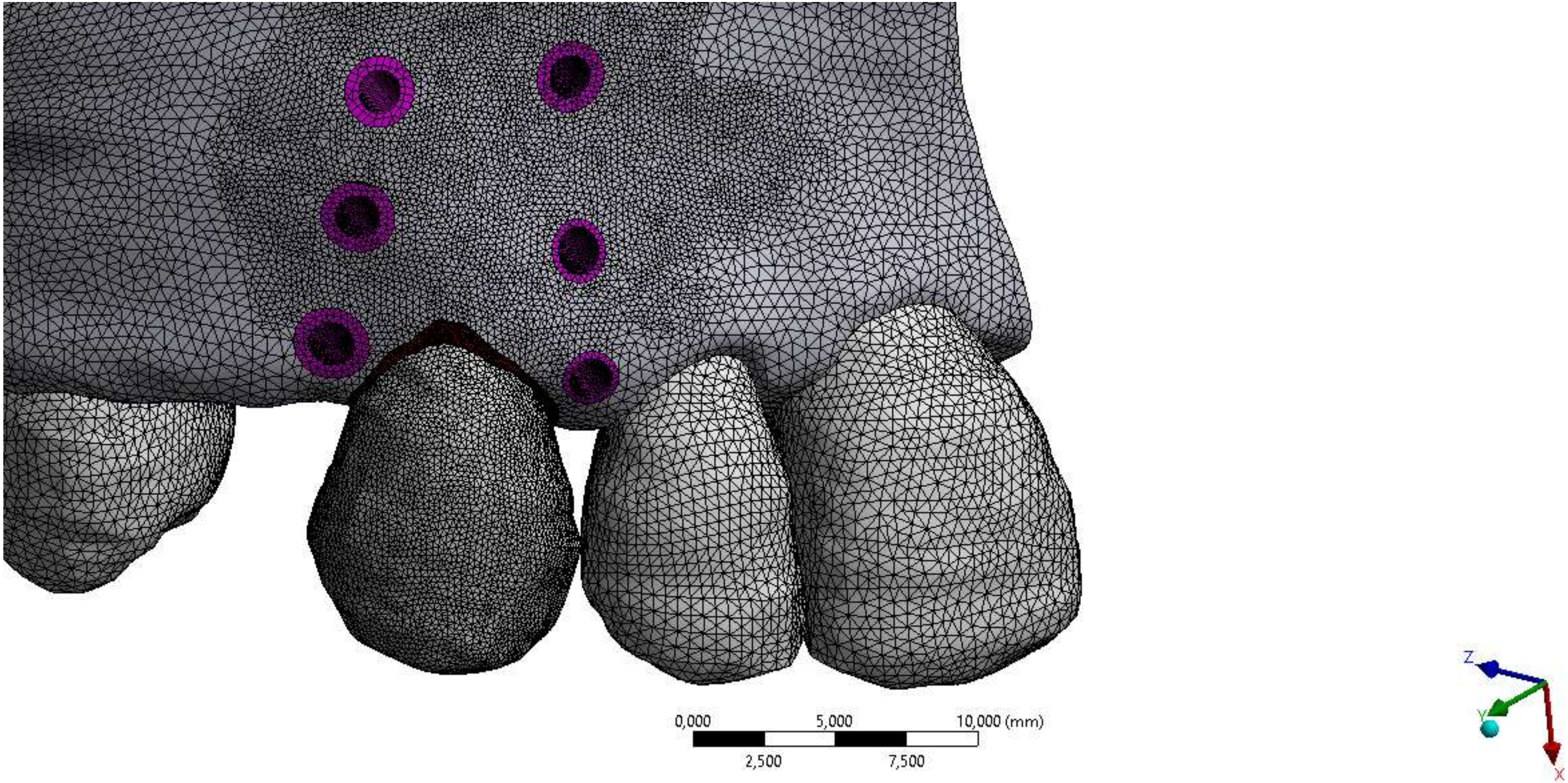
0,000
5,000
10,000 (mm)
2,500
7,500
Z
Y
X

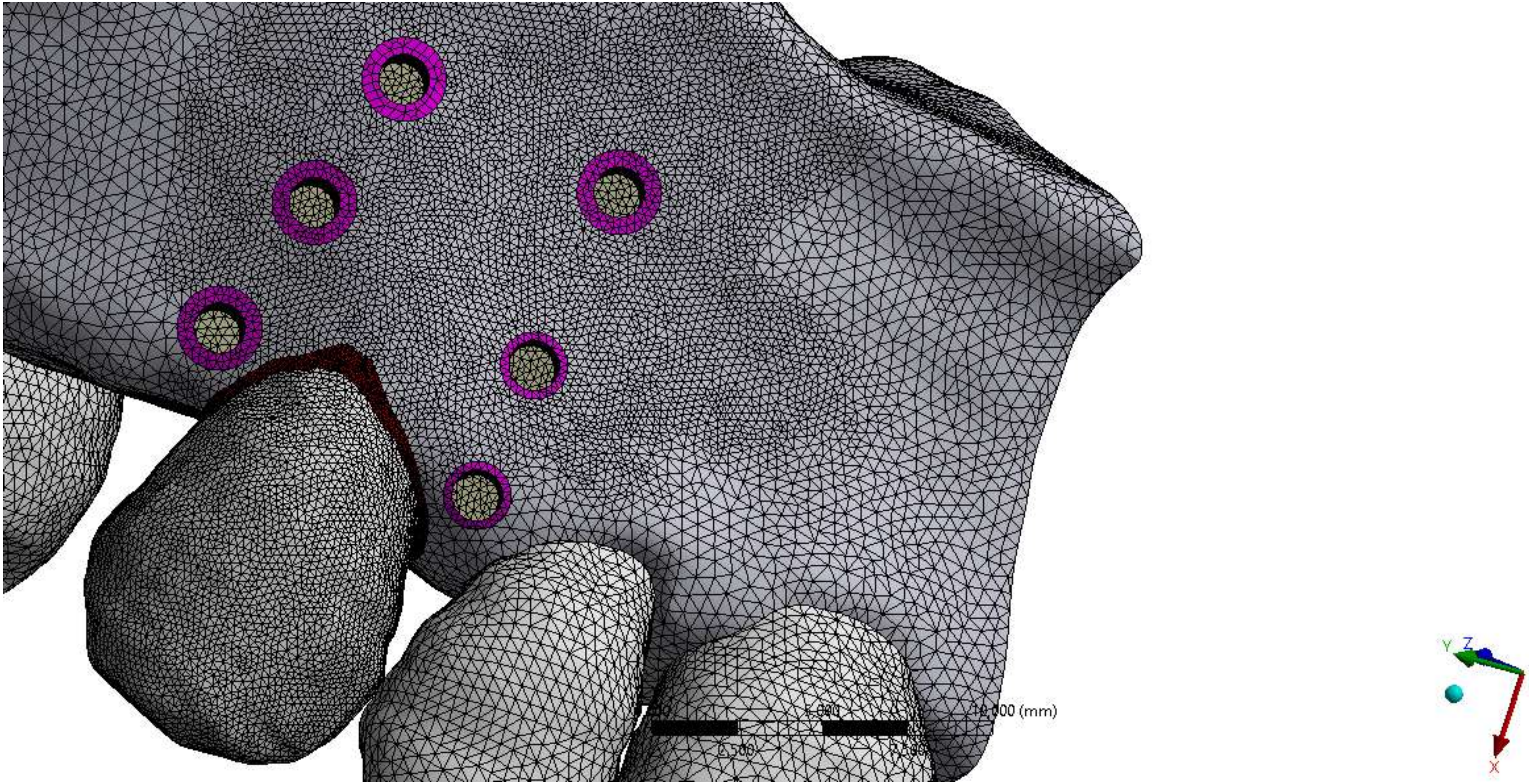
10.000 (mm)
2.500
Y
Z
X

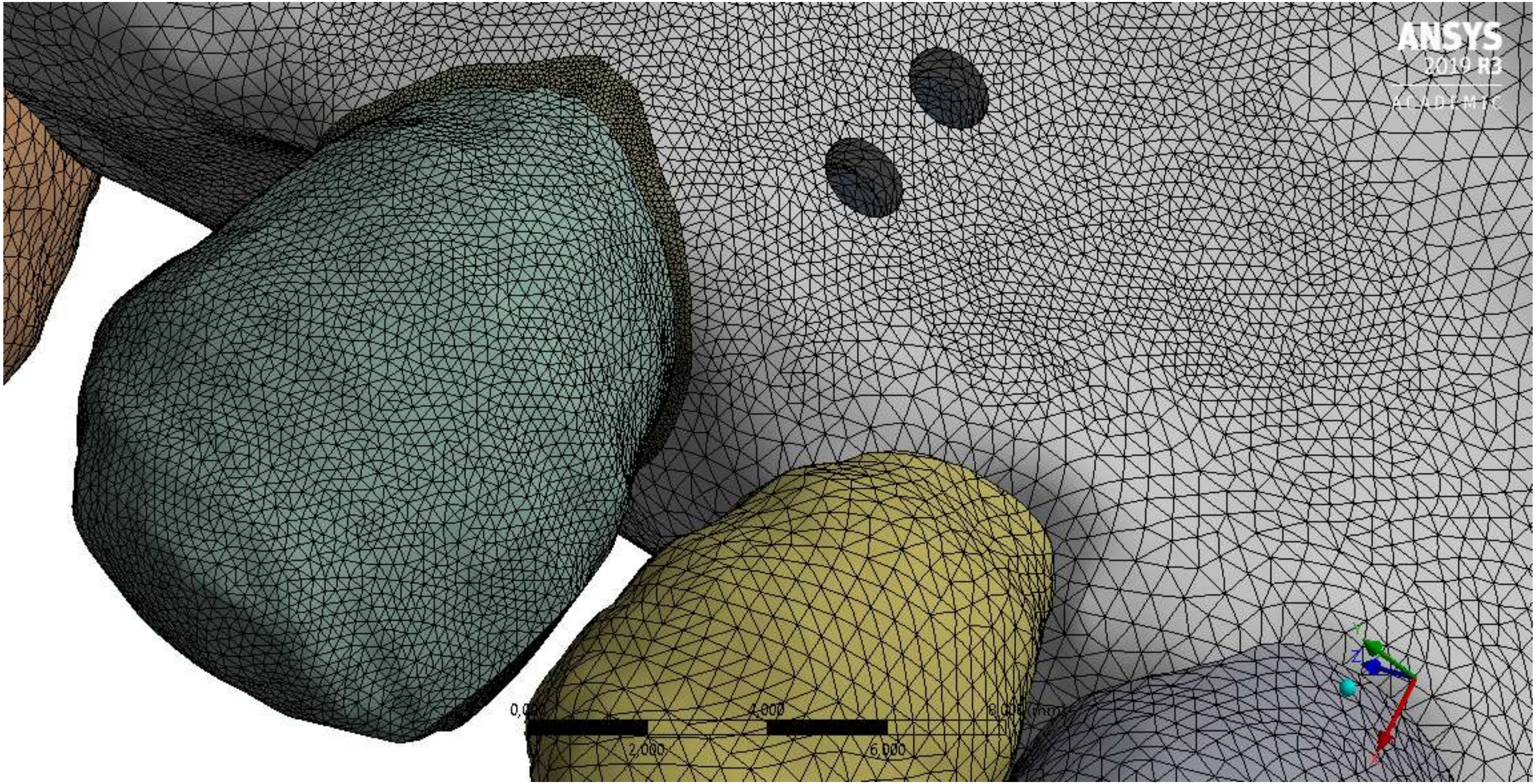
ANSYS
2019 R3
ACADEMIC
4,000
2,000
6,000

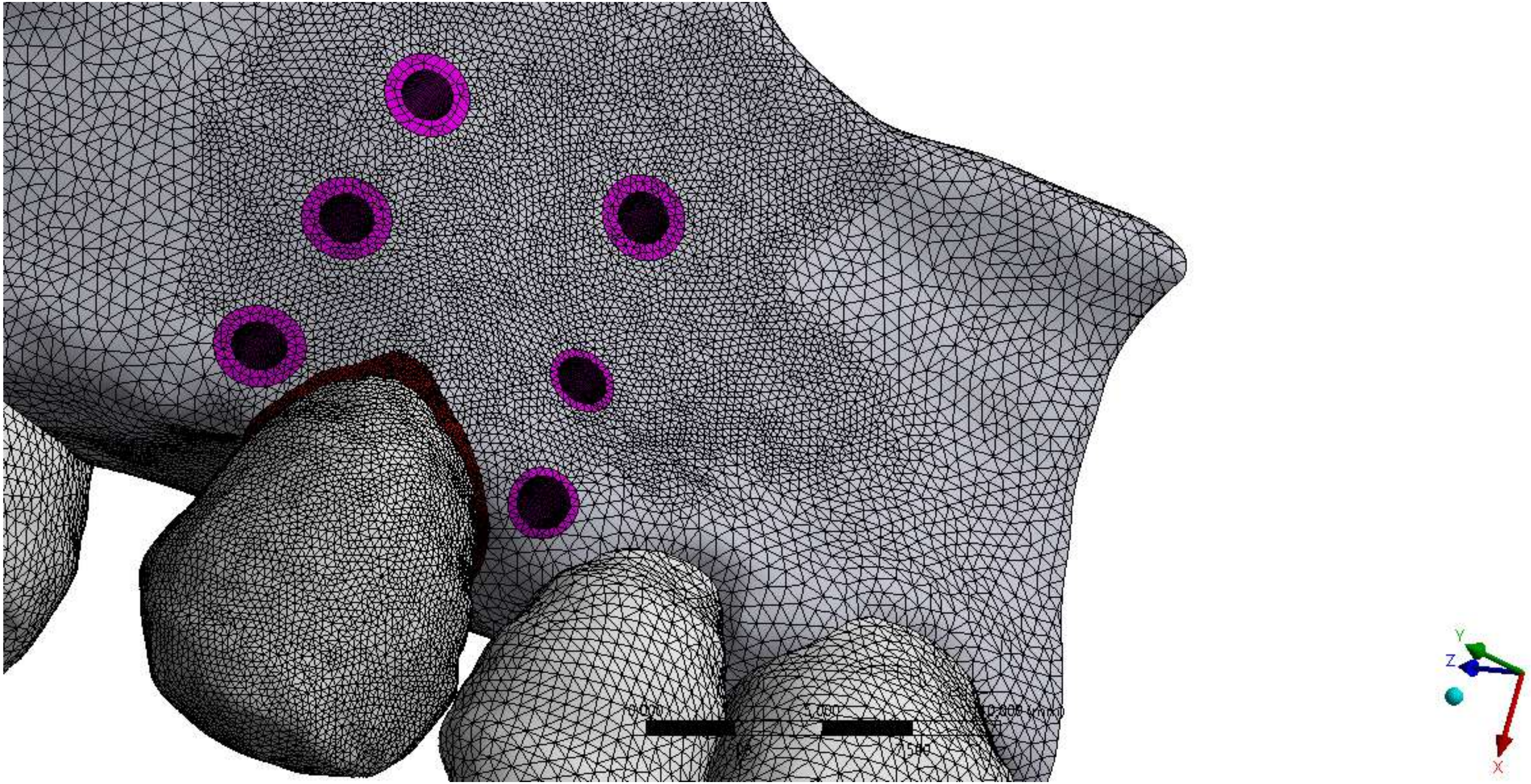
Y
Z
X

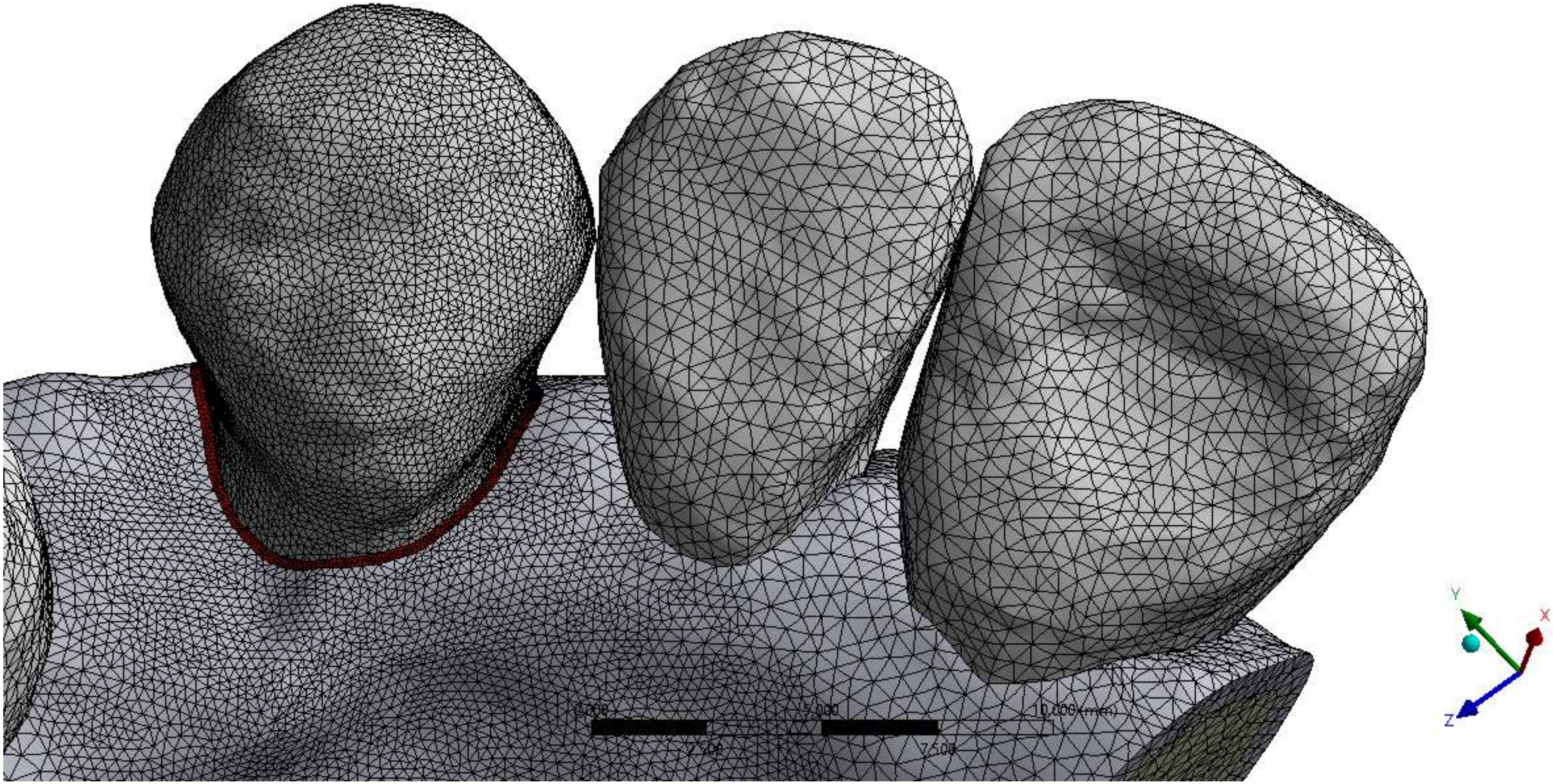
0.000
5.000
10.000 (mm)
7.500
Y
X
Z

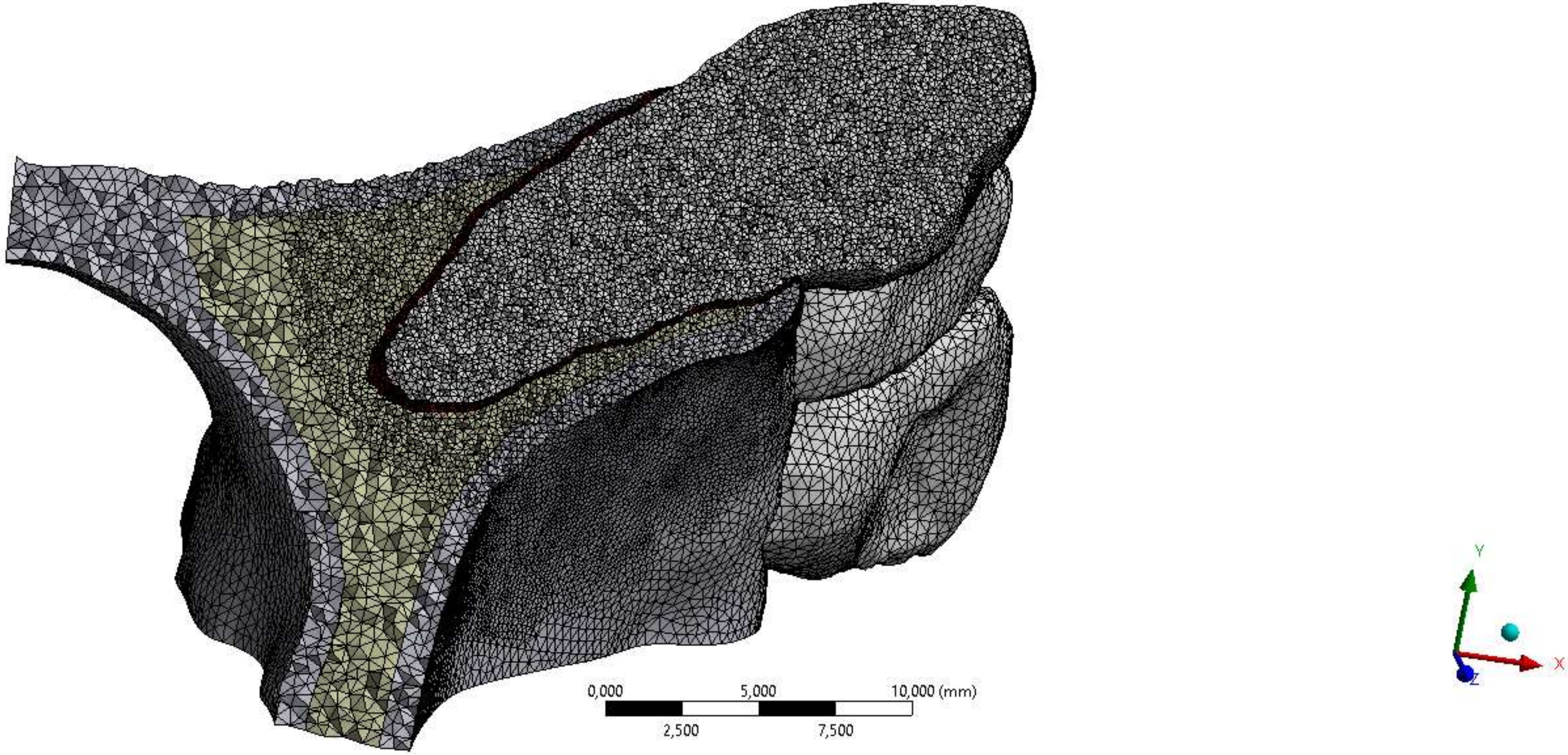

0,000
5,000
10,000 (mm)
2,500
7,500
Y
X
Z

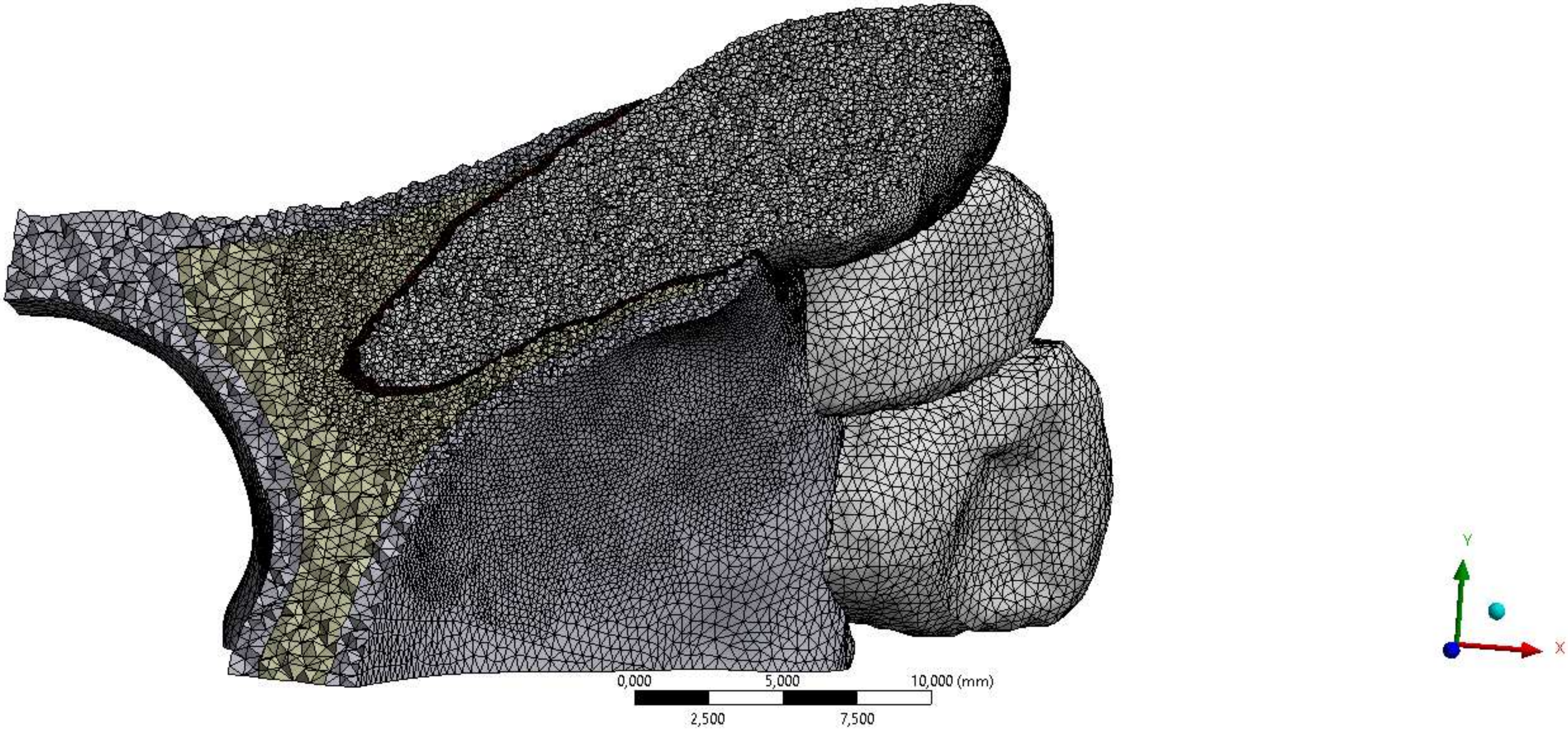
0,000
5,000
10,000 (mm)
2,500
7,500
Y
X

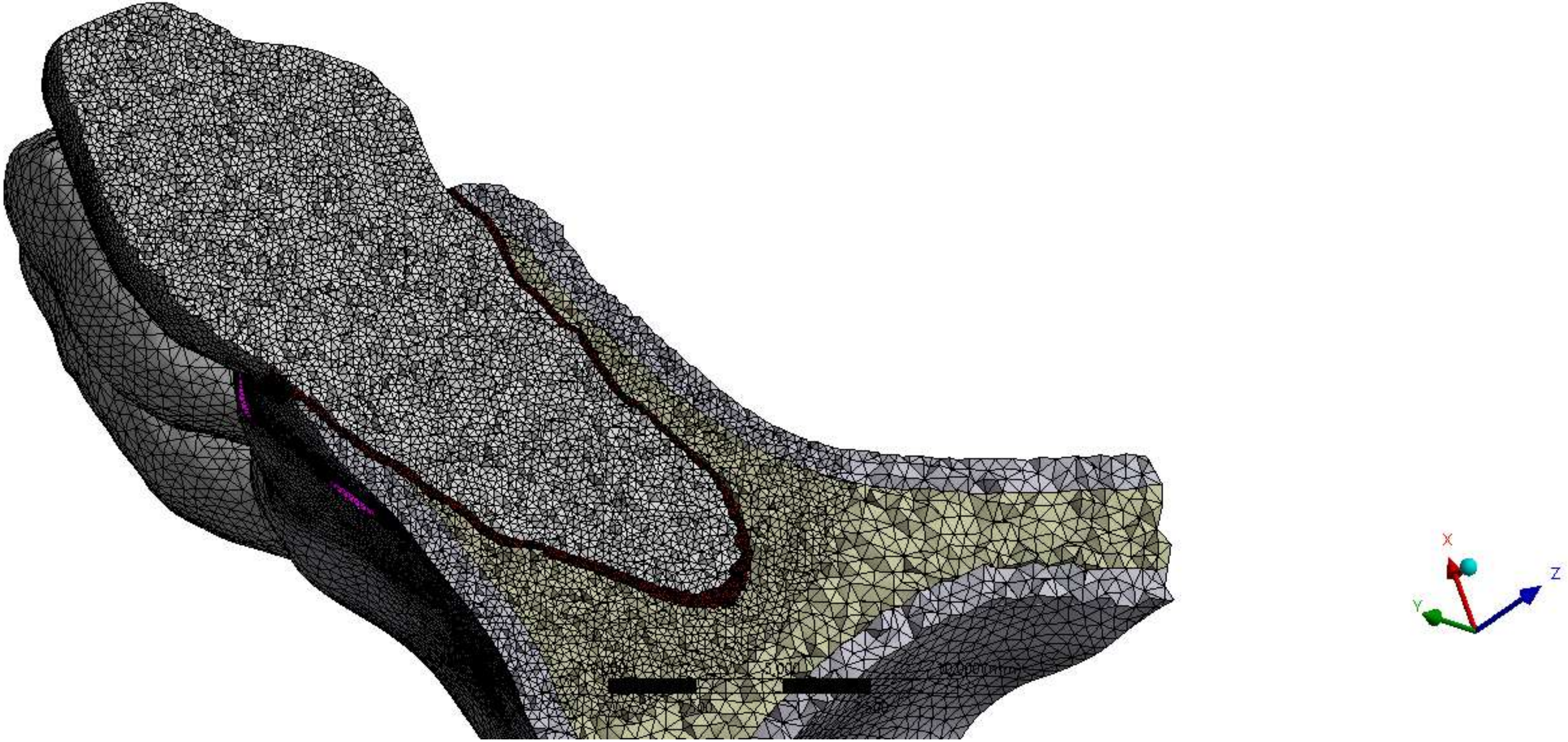
5.000
X
Y
Z

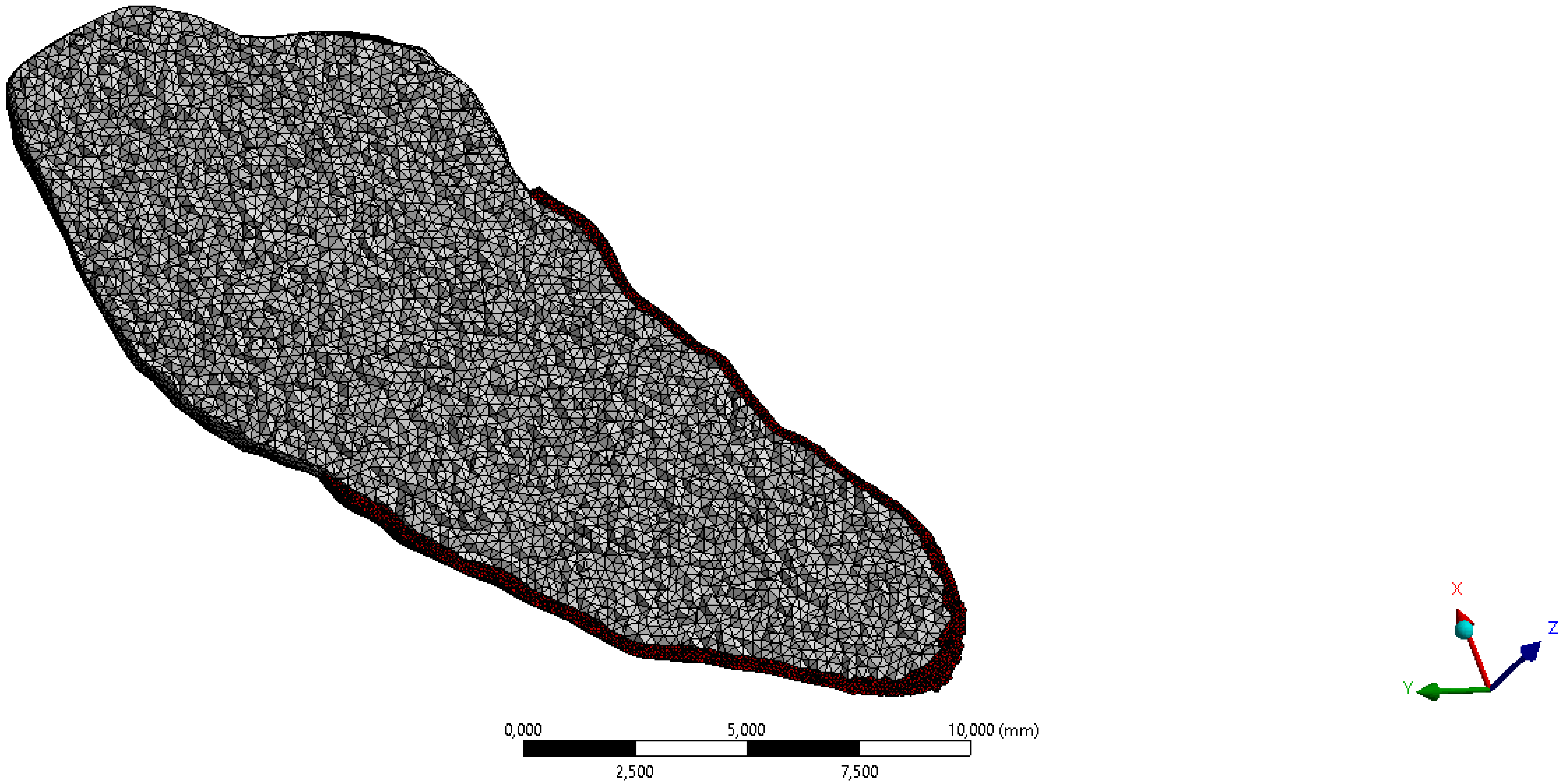
0,000
5,000
10,000 (mm)
2,500
7,500
X
Y
Z

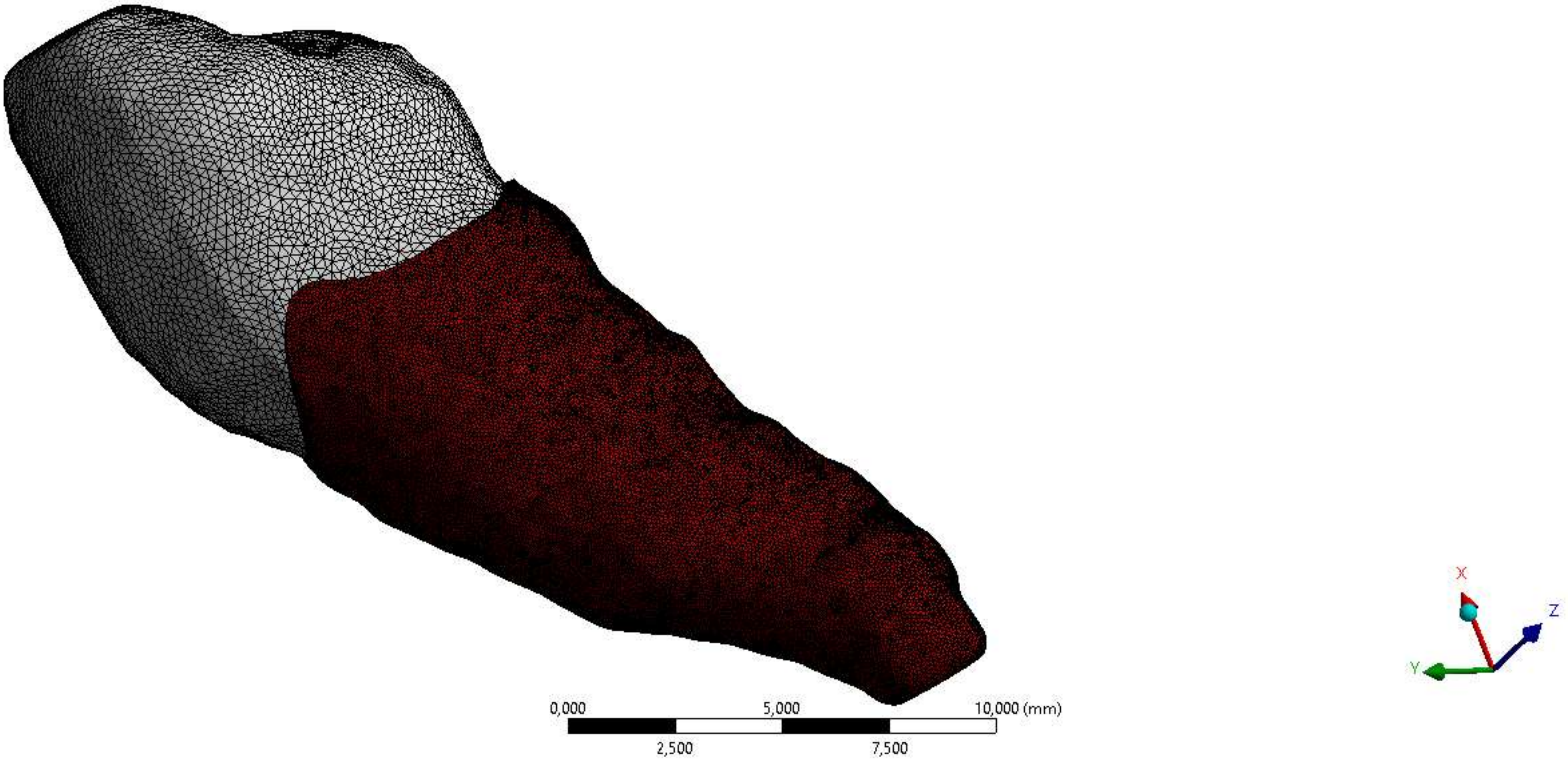
0,000
5,000
10,000 (mm)
2,500
7,500
X
Z
Y

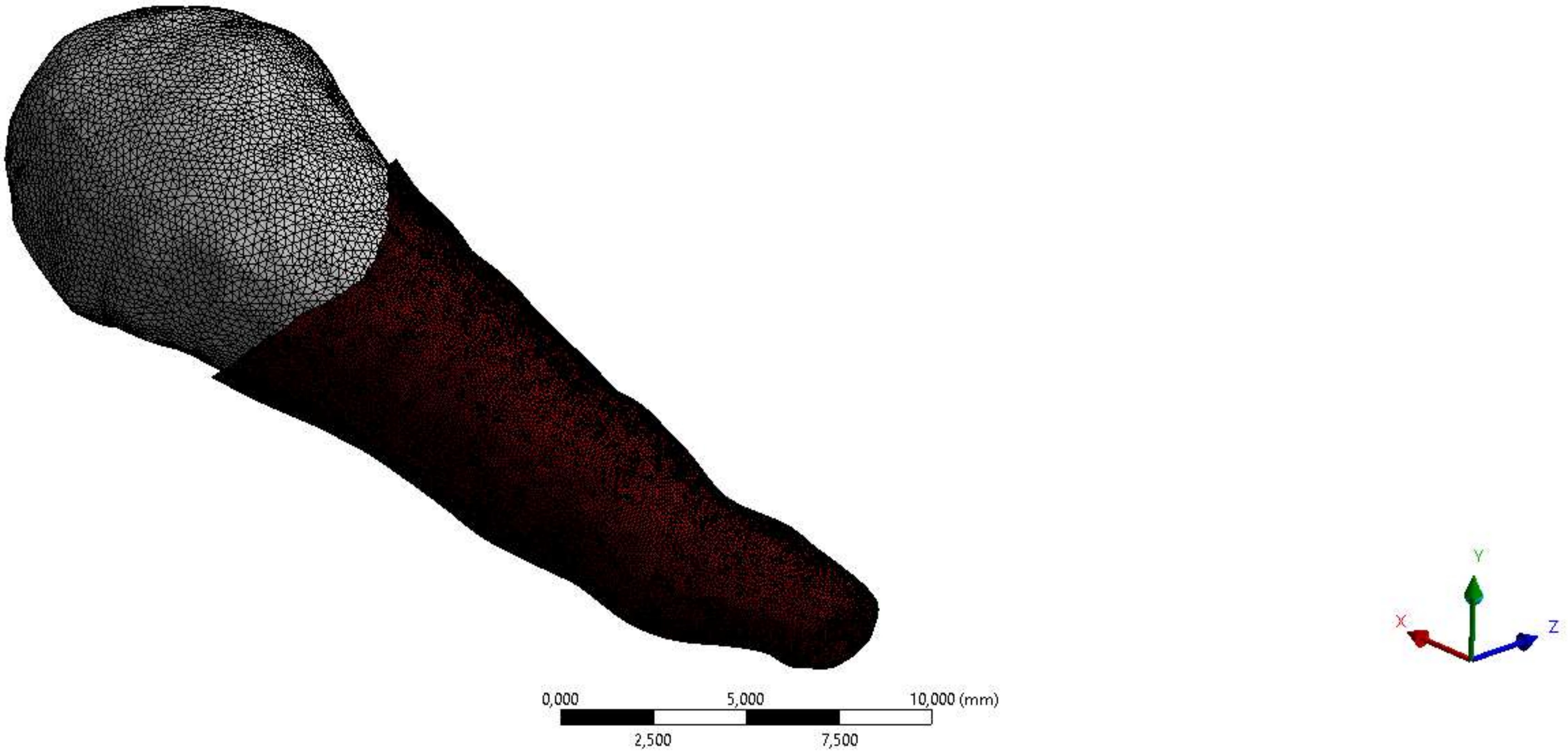
0,000
5,000
10,000 (mm)
2,500
7,500
Y
X
Z

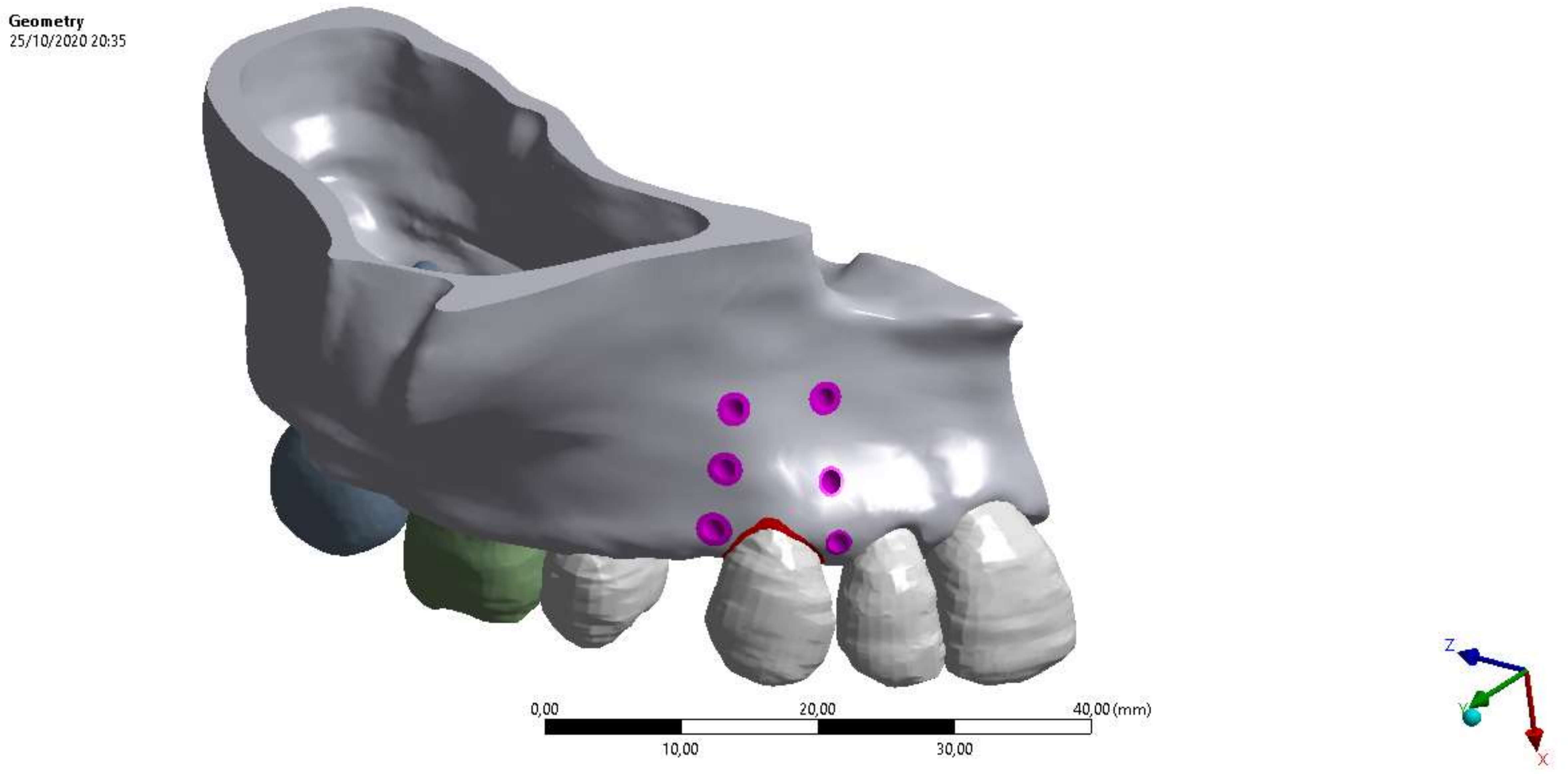
Geometry
25/10/2020 20:35
0,00
20,00
40,00 (mm)
10,00
30,00
Z
Y
X

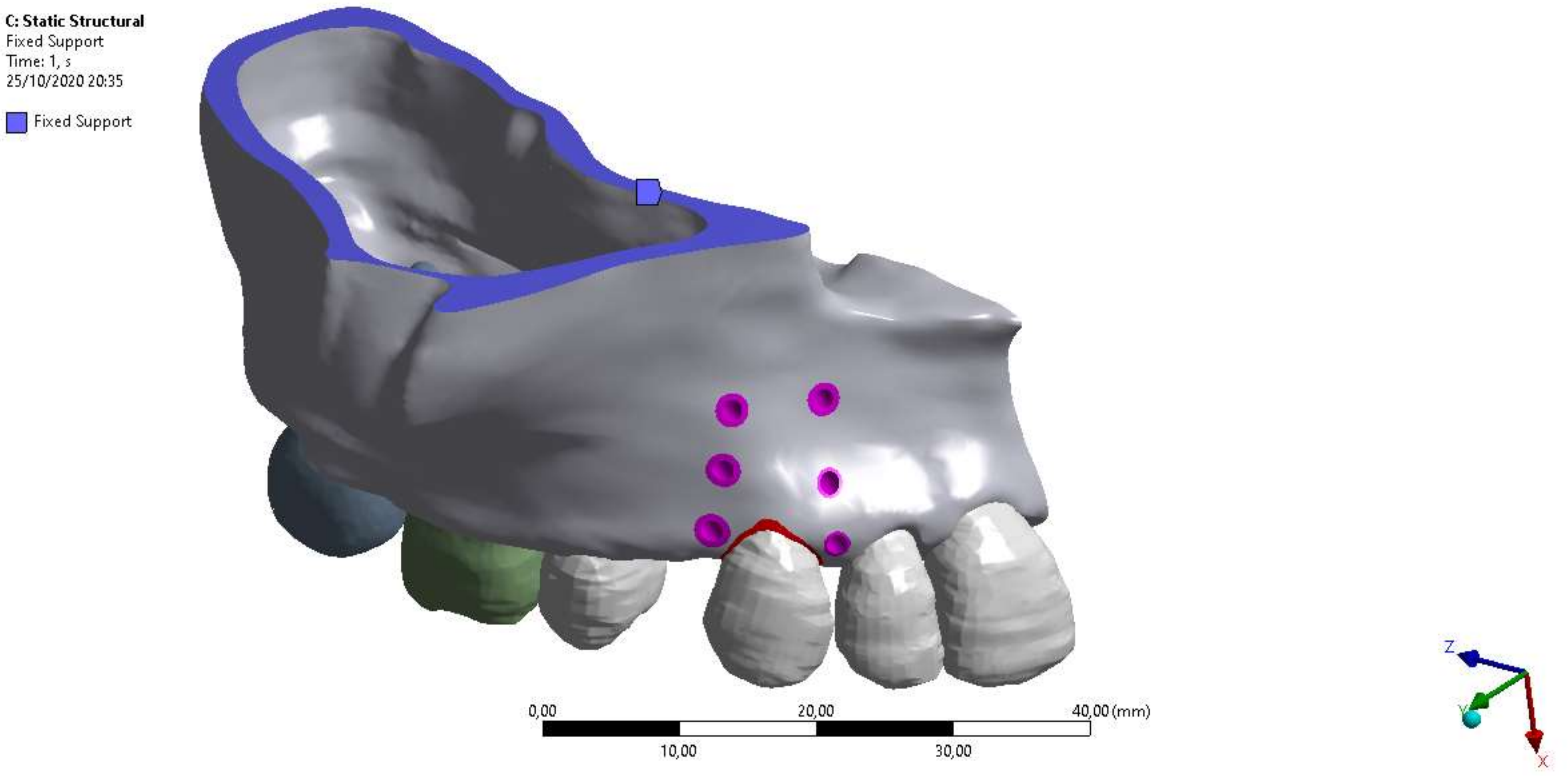

C: Static Structural
Fixed Support
Time: 1, s
25/10/2020 20:35
Fixed Support
0,00
20,00
40,00 (mm)
10,00
30,00
Z
Y
X

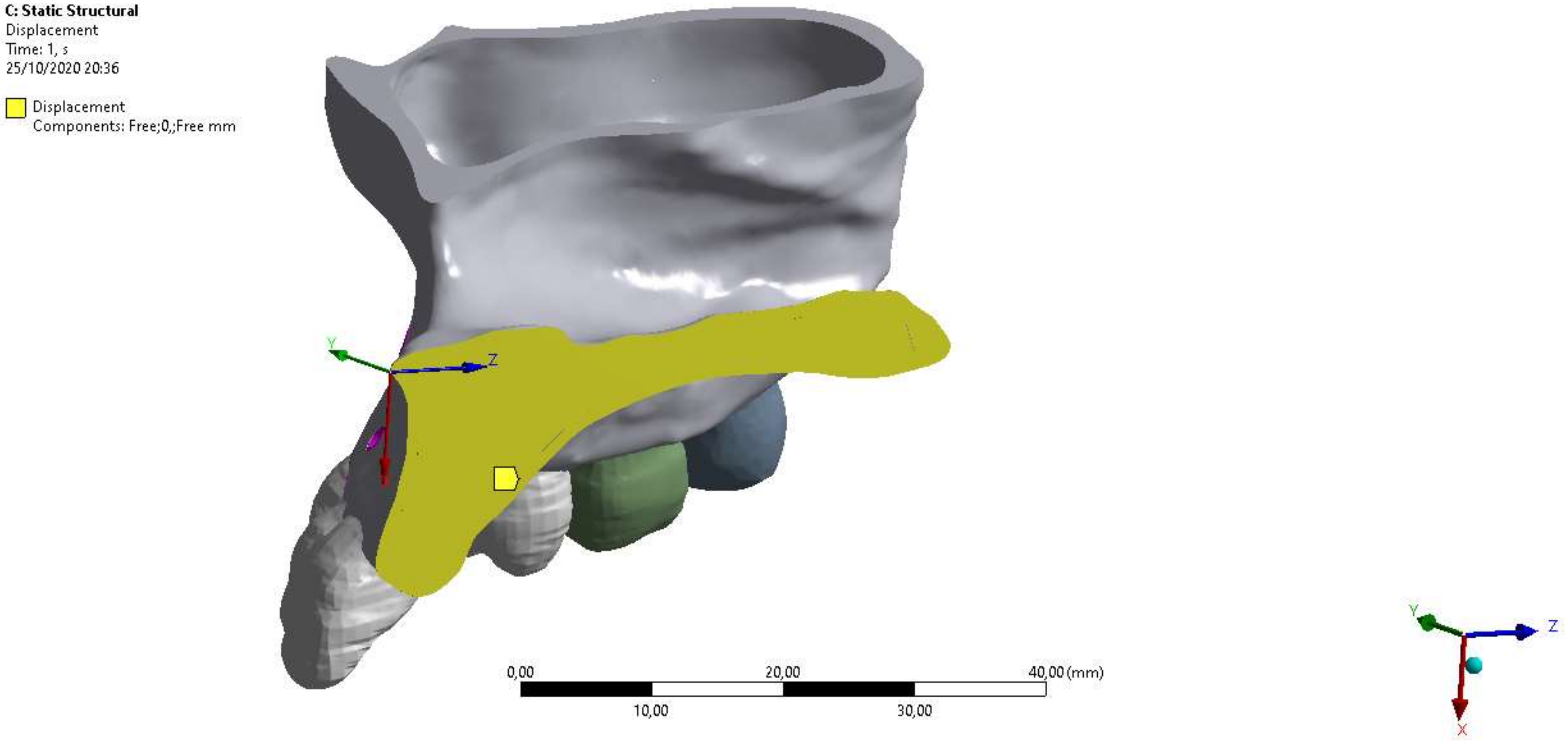
C: Static Structural
Displacement
Time: 1, s
25/10/2020 20:36
Displacement
Components: Free;0,;Free mm
0,00
20,00
40,00 (mm)
10,00
30,00
Y
Z
X

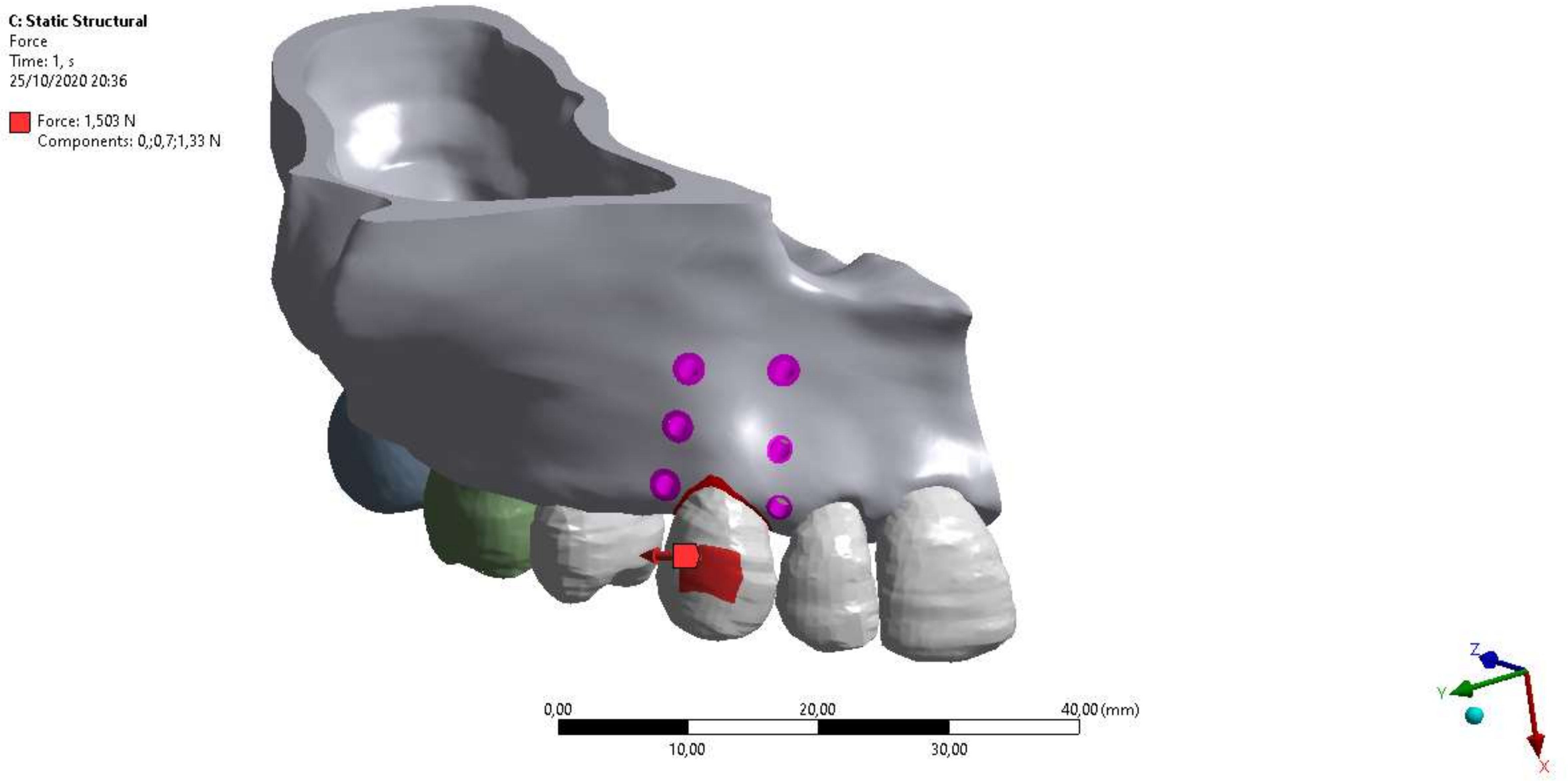
C: Static Structural
Force
Time: 1, s
25/10/2020 20:36
Force: 1,503 N
Components: 0,;0,7;1,33 N
0,00
10,00
20,00
30,00
40,00 (mm)
Z
Y
X

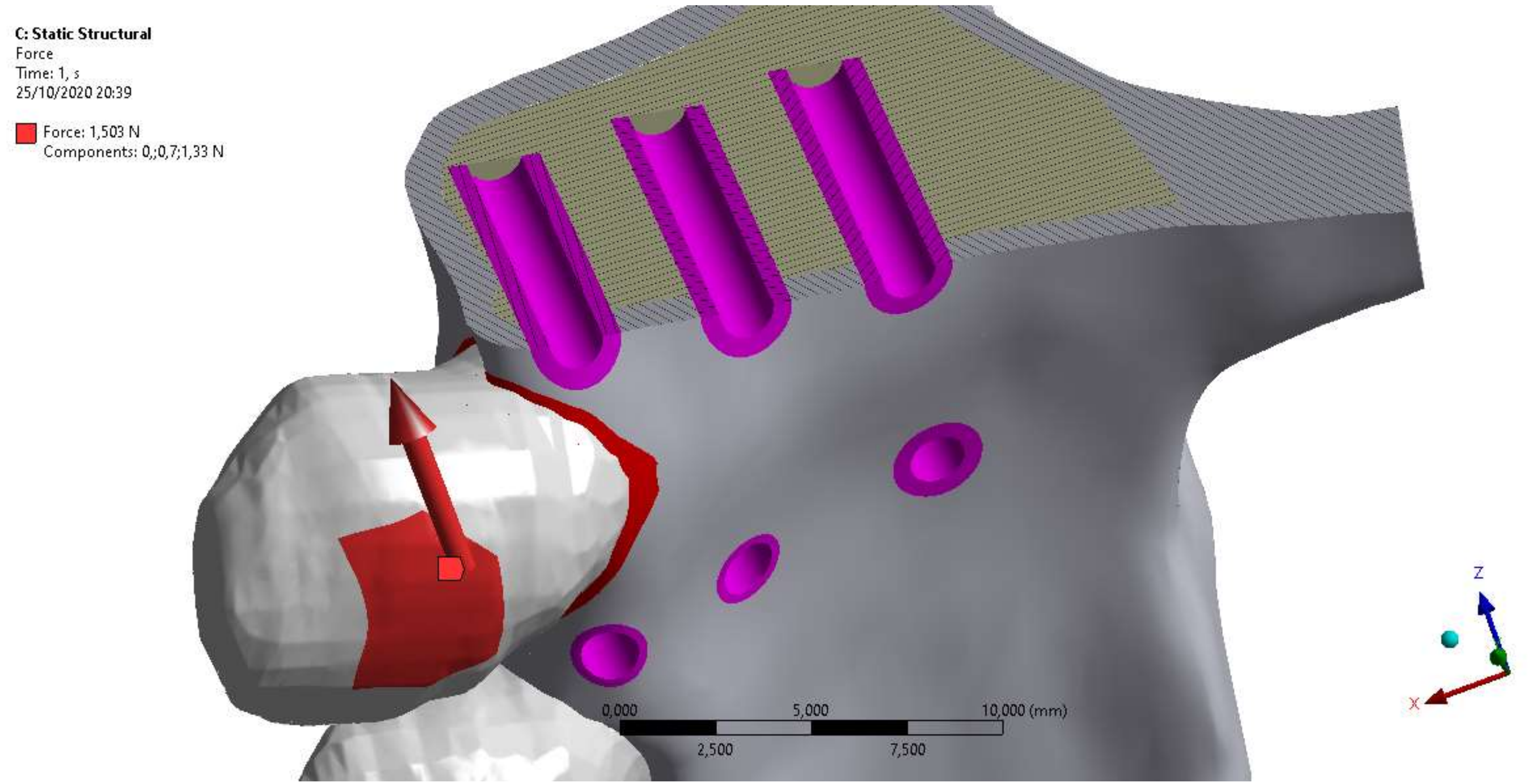
C: Static Structural
Force
Time: 1, s
25/10/2020 20:39
Force: 1,503 N
Components: 0,;0,7;1,33 N
0,000
5,000
10,000 (mm)
2,500
7,500
Z
X

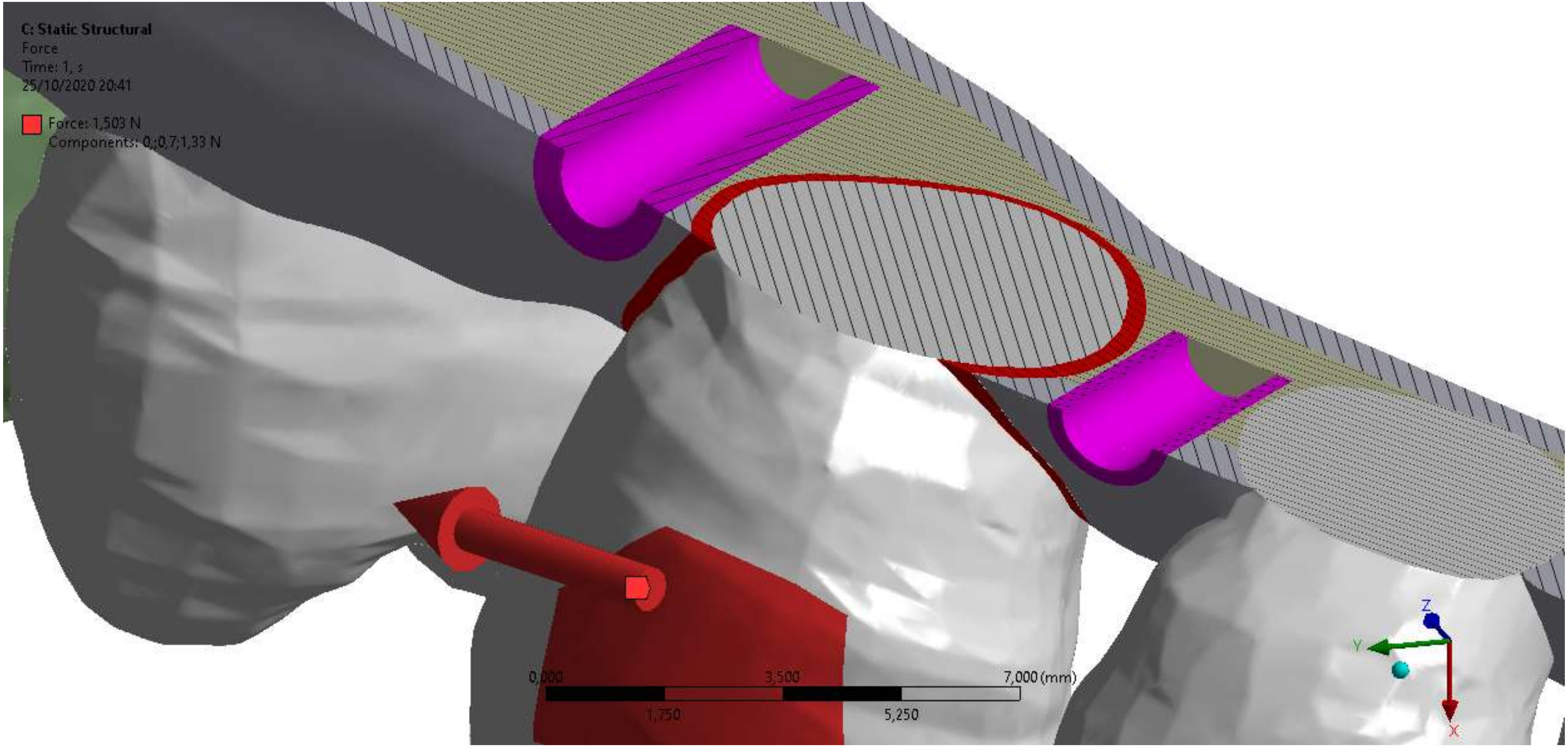
C: Static Structural
Force
Time: 1, s
25/10/2020 20:41
Force: 1,503 N
Components: 0,;0,7;1,33 N
0,000
3,500
7,000 (mm)
1,750
5,250
Z
Y
X

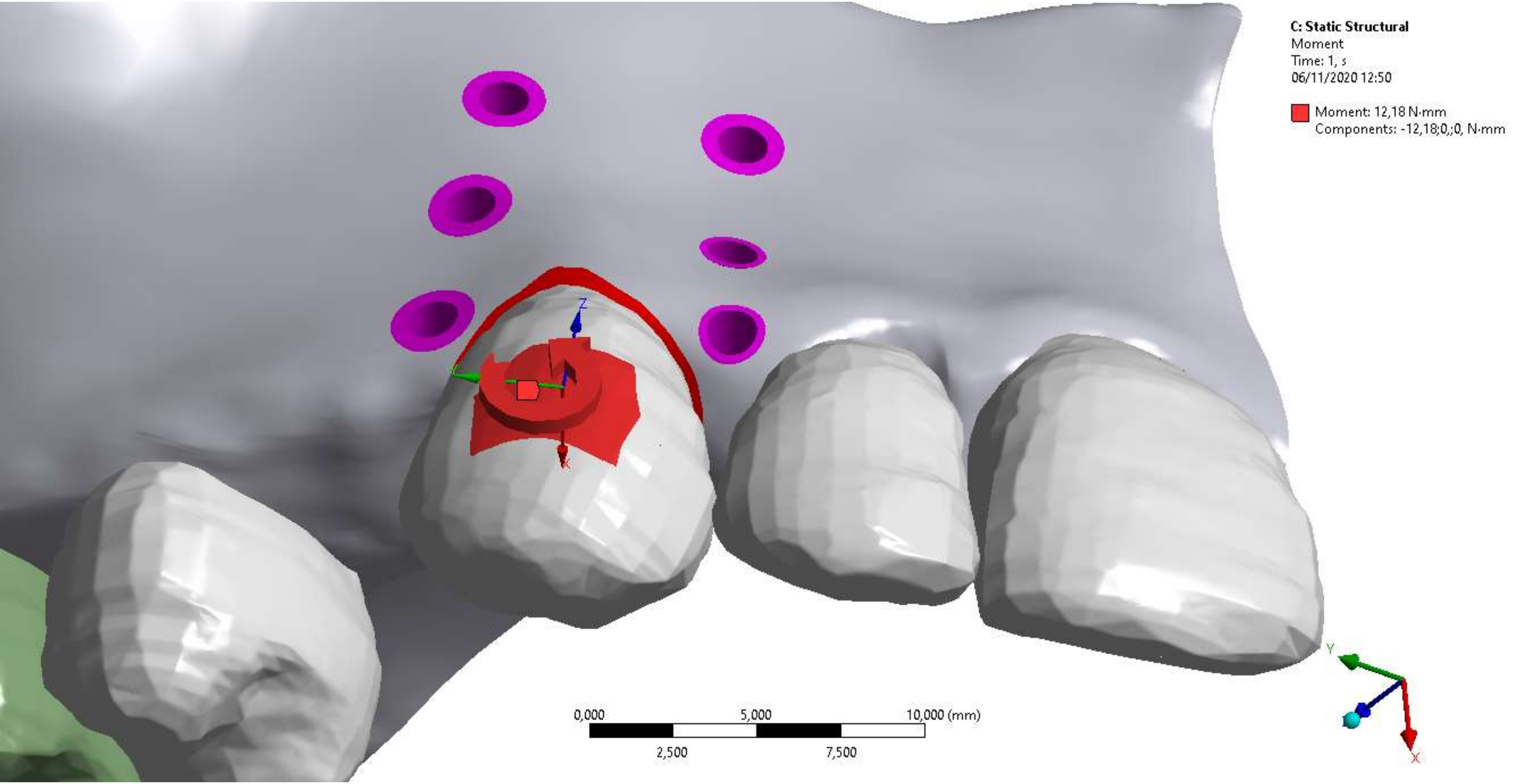
C: Static Structural
Moment
Time: 1, s
06/11/2020 12:50
Moment: 12,18 N·mm
Components: -12,18;0,;0, N·mm
0,000
5,000
10,000 (mm)
2,500
7,500

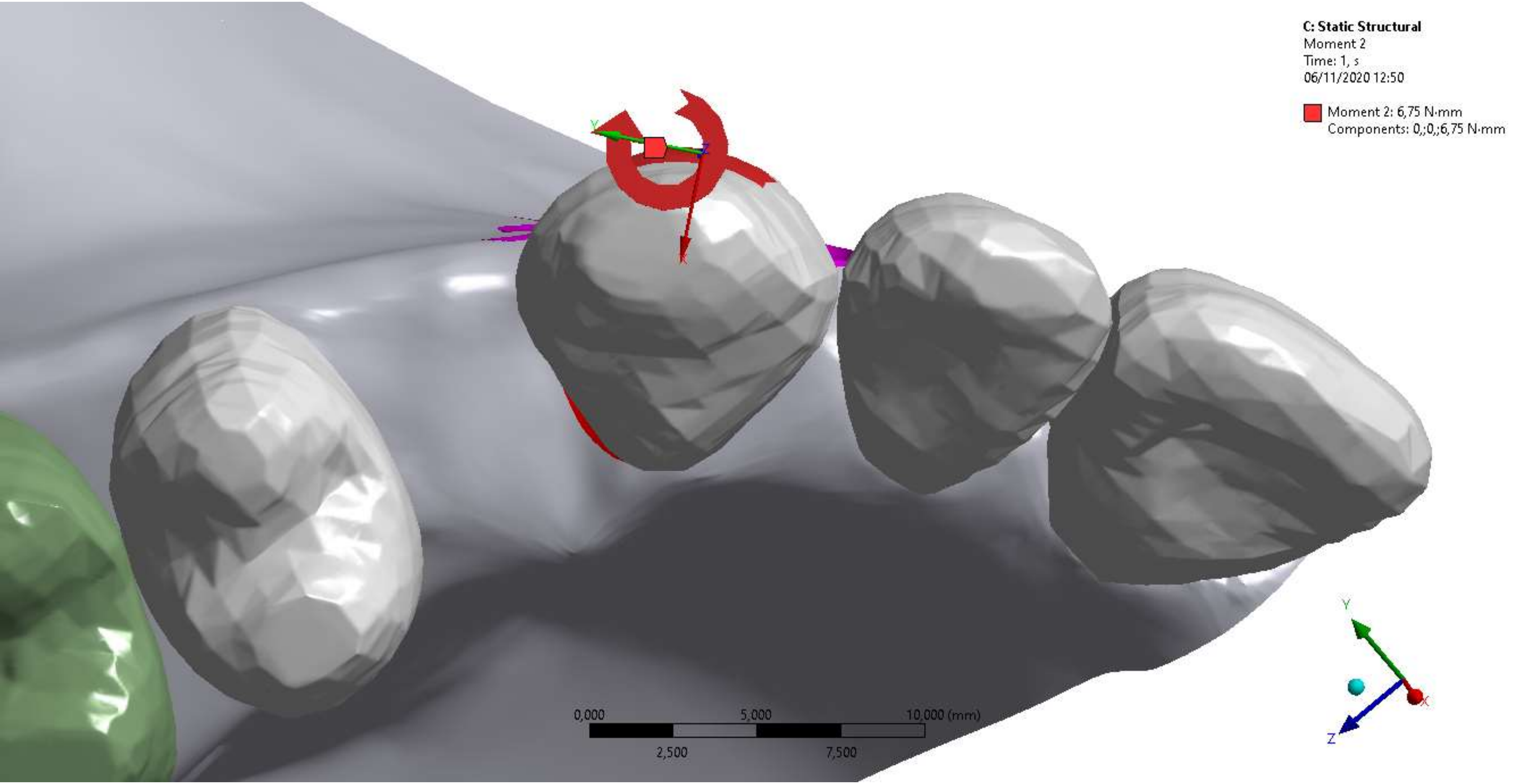
C: Static Structural
Moment 2
Time: 1, s
06/11/2020 12:50
Moment 2: 6,75 N·mm
Components: 0,;0,;6,75 N·mm
0,000
5,000
10,000 (mm)
2,500
7,500

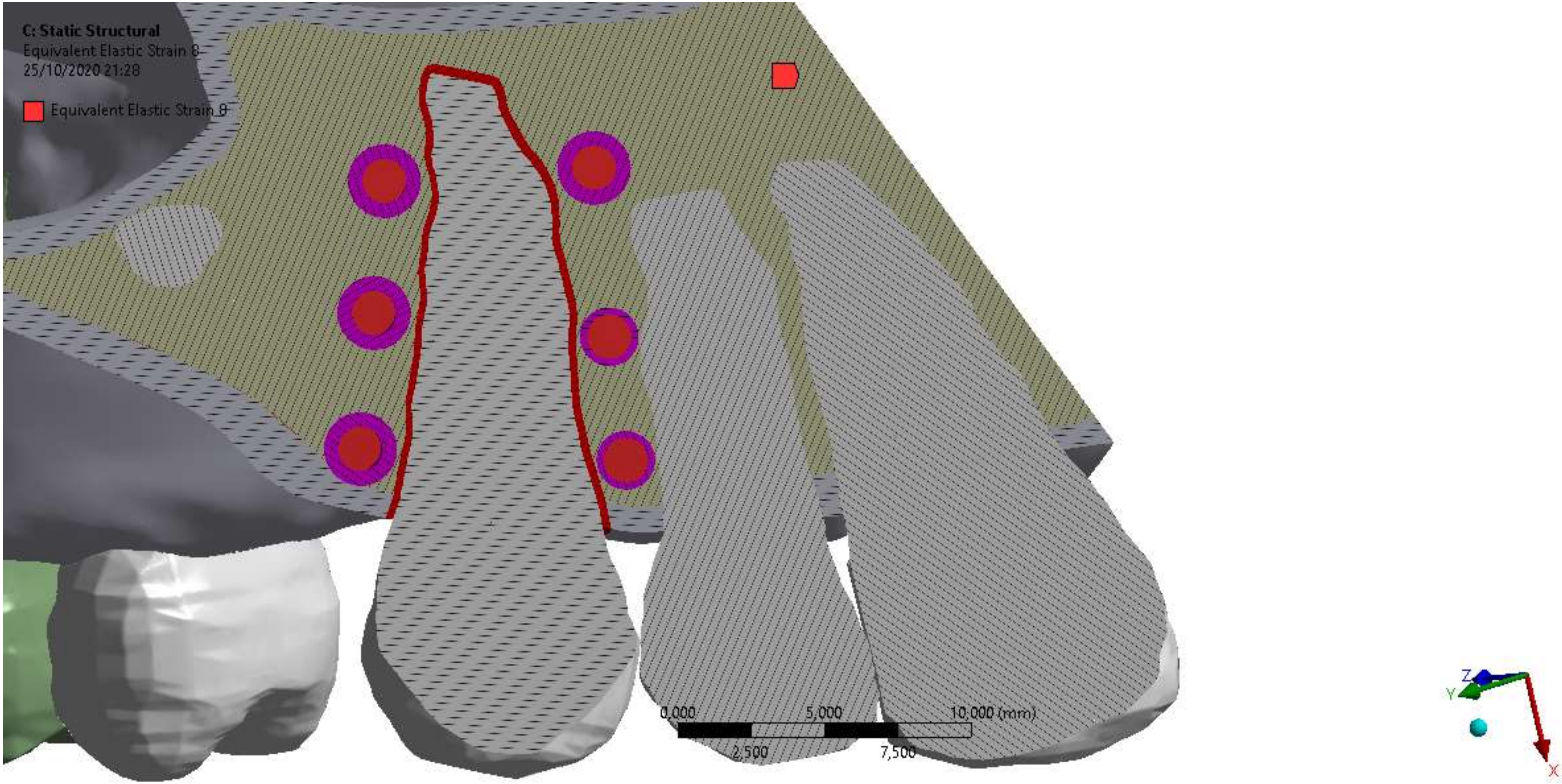
C: Static Structural
Equivalent Elastic Strain 8
25/10/2020 21:28
Equivalent Elastic Strain 8
0,000
5,000
10,000 (mm)
2,500
7,500
Z
Y
X

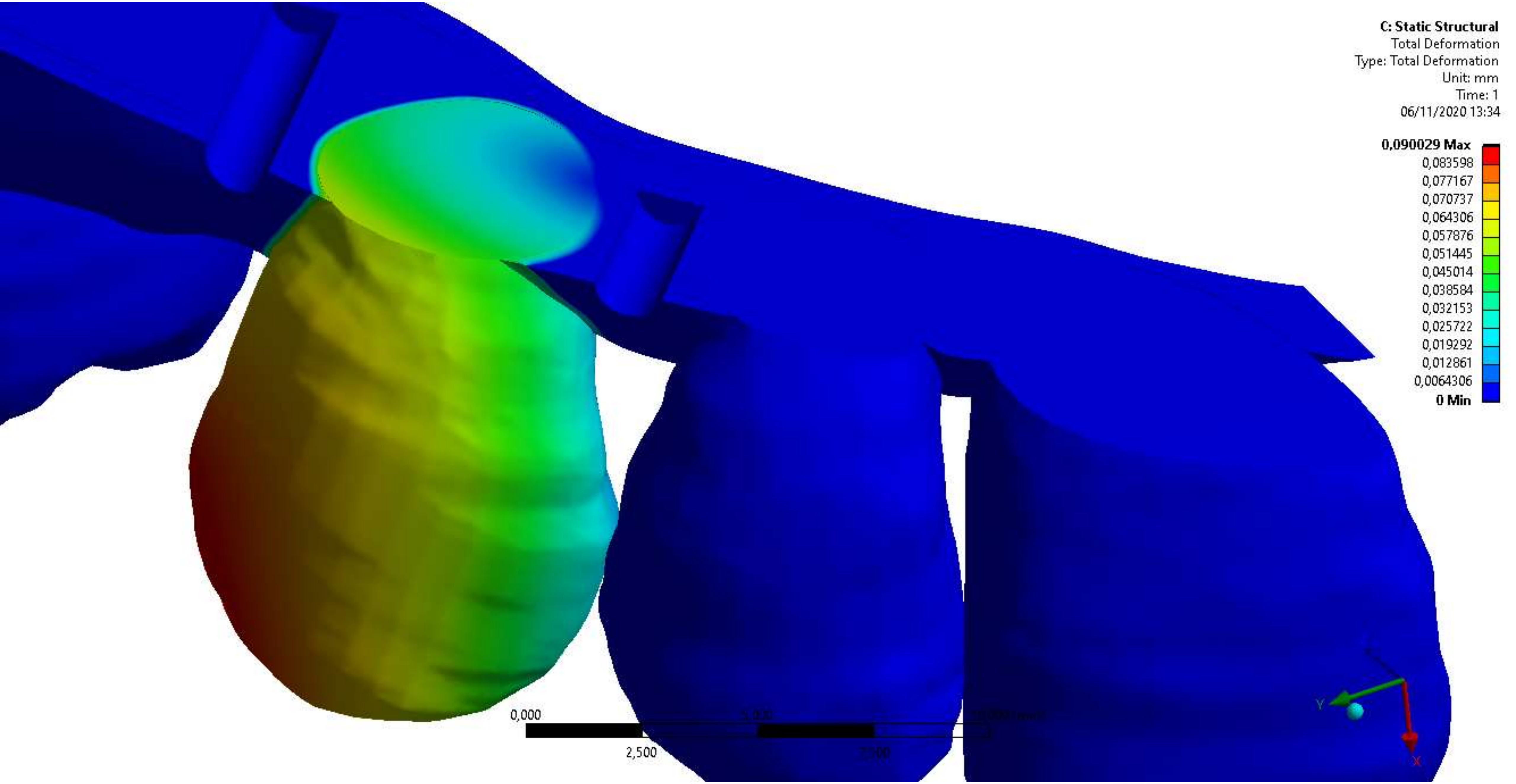
C: Static Structural
Total Deformation
Type: Total Deformation
Unit: mm
Time: 1
06/11/2020 13:34
0,090029 Max
0,083598
0,077167
0,070737
0,064306
0,057876
0,051445
0,045014
0,038584
0,032153
0,025722
0,019292
0,012861
0,0064306
0 Min
0,000
2,500

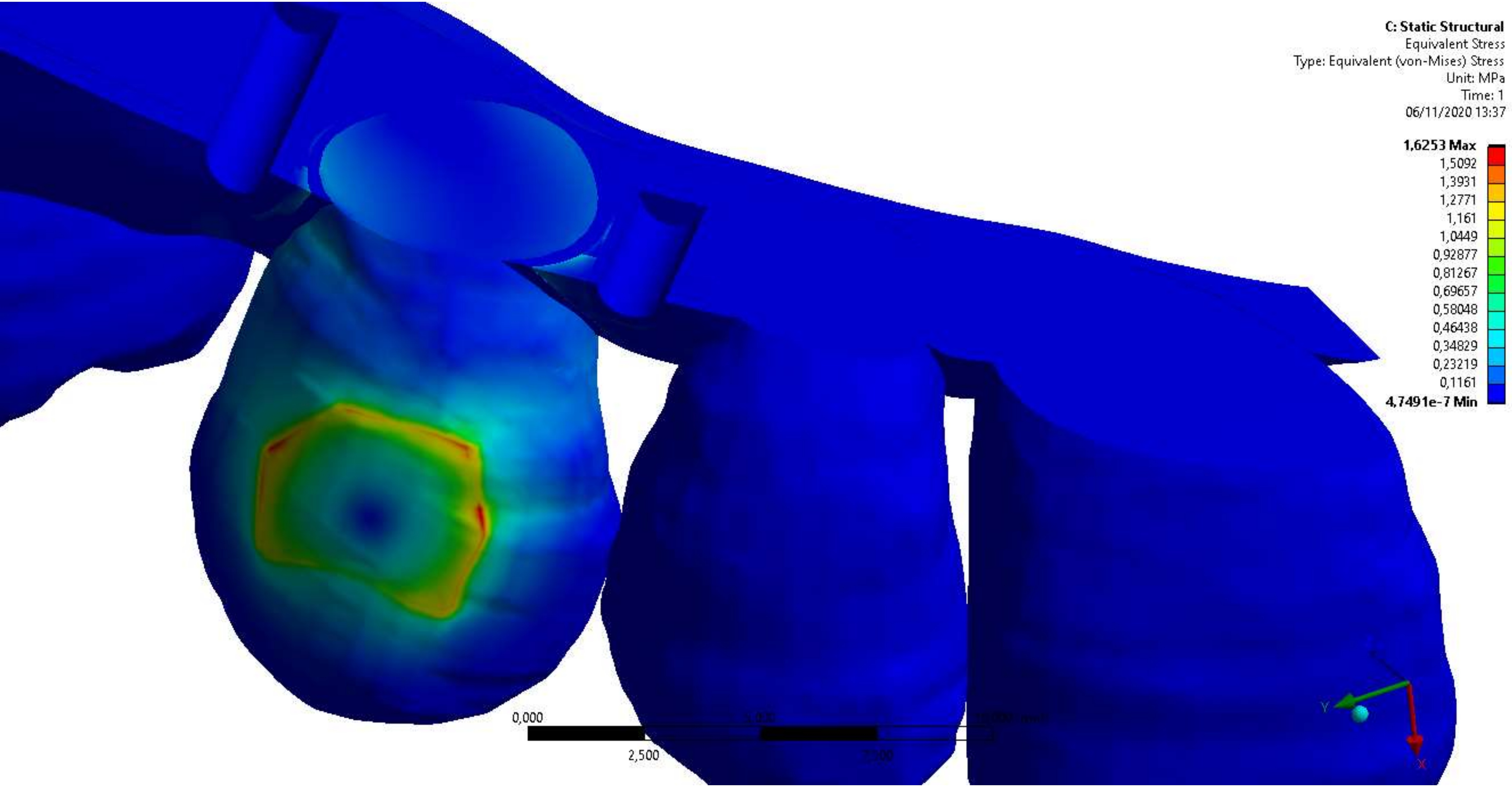
C: Static Structural
Equivalent Stress
Type: Equivalent (von-Mises) Stress
Unit: MPa
Time: 1
06/11/2020 13:37
1,6253 Max
1,5092
1,3931
1,2771
1,161
1,0449
0,92877
0,81267
0,69657
0,58048
0,46438
0,34829
0,23219
0,1161
4,7491e-7 Min
0,000
2,500
Y
X

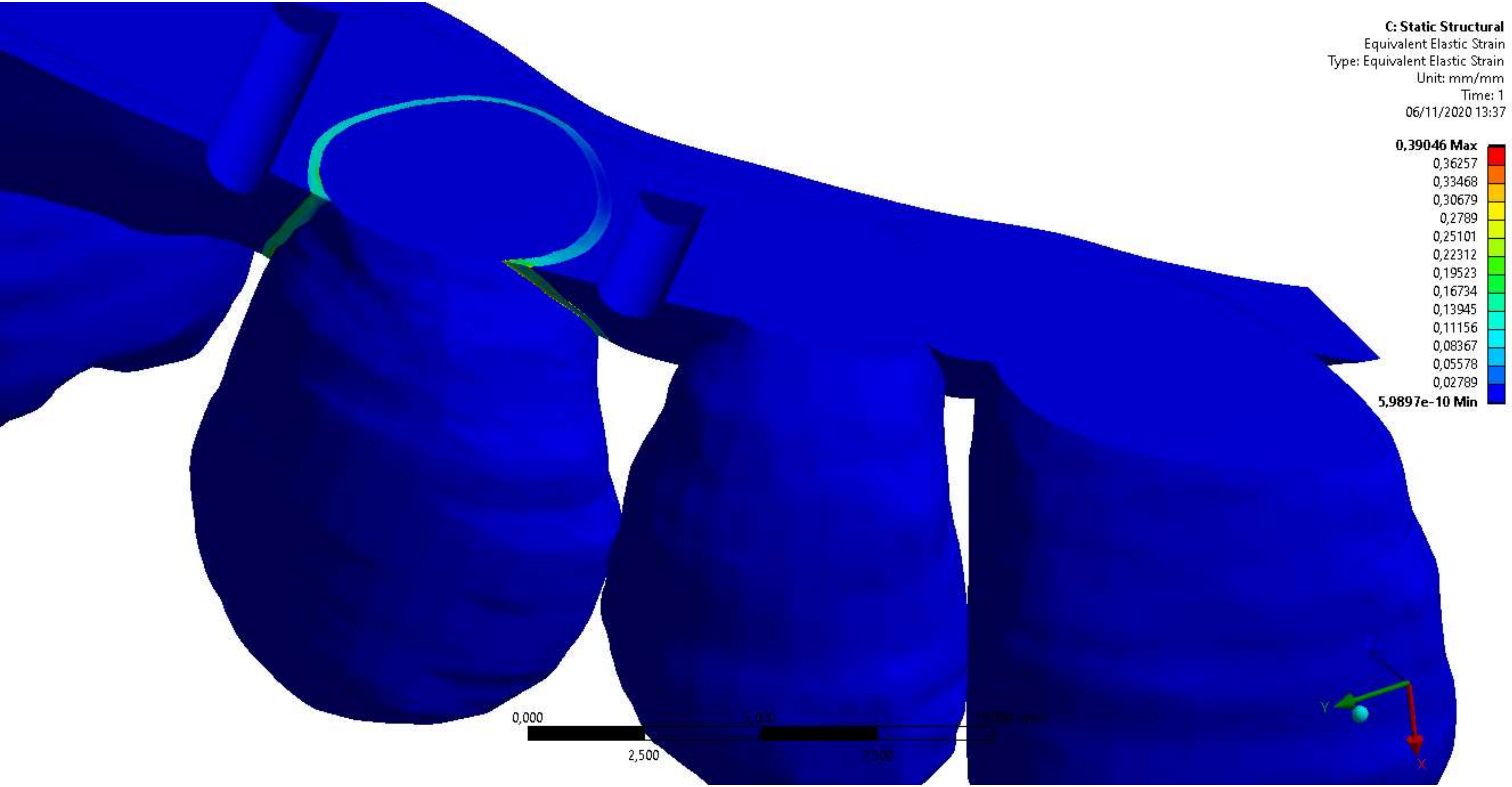

C: Static Structural
Equivalent Elastic Strain
Type: Equivalent Elastic Strain
Unit: mm/mm
Time: 1
06/11/2020 13:37
0,39046 Max
0,36257
0,33468
0,30679
0,2789
0,25101
0,22312
0,19523
0,16734
0,13945
0,11156
0,08367
0,05578
0,02789
5,9897e-10 Min
0,000
2,500
Y
X

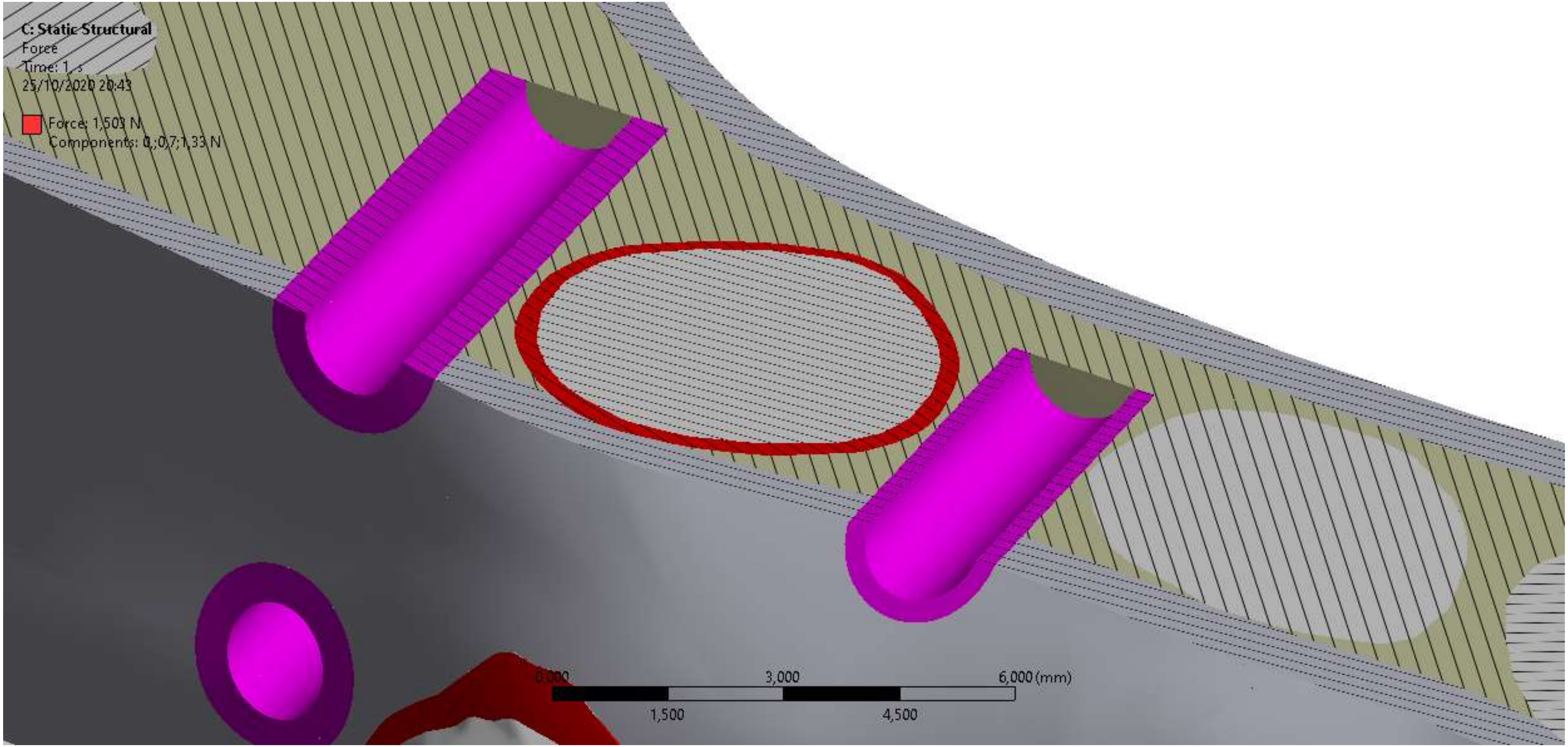
C: Static Structural
Force
Time: 1, s
25/10/2020 20:43
Force: 1,503 N
Components: 0,;0,7;1,33 N
0,000
3,000
6,000 (mm)
1,500
4,500

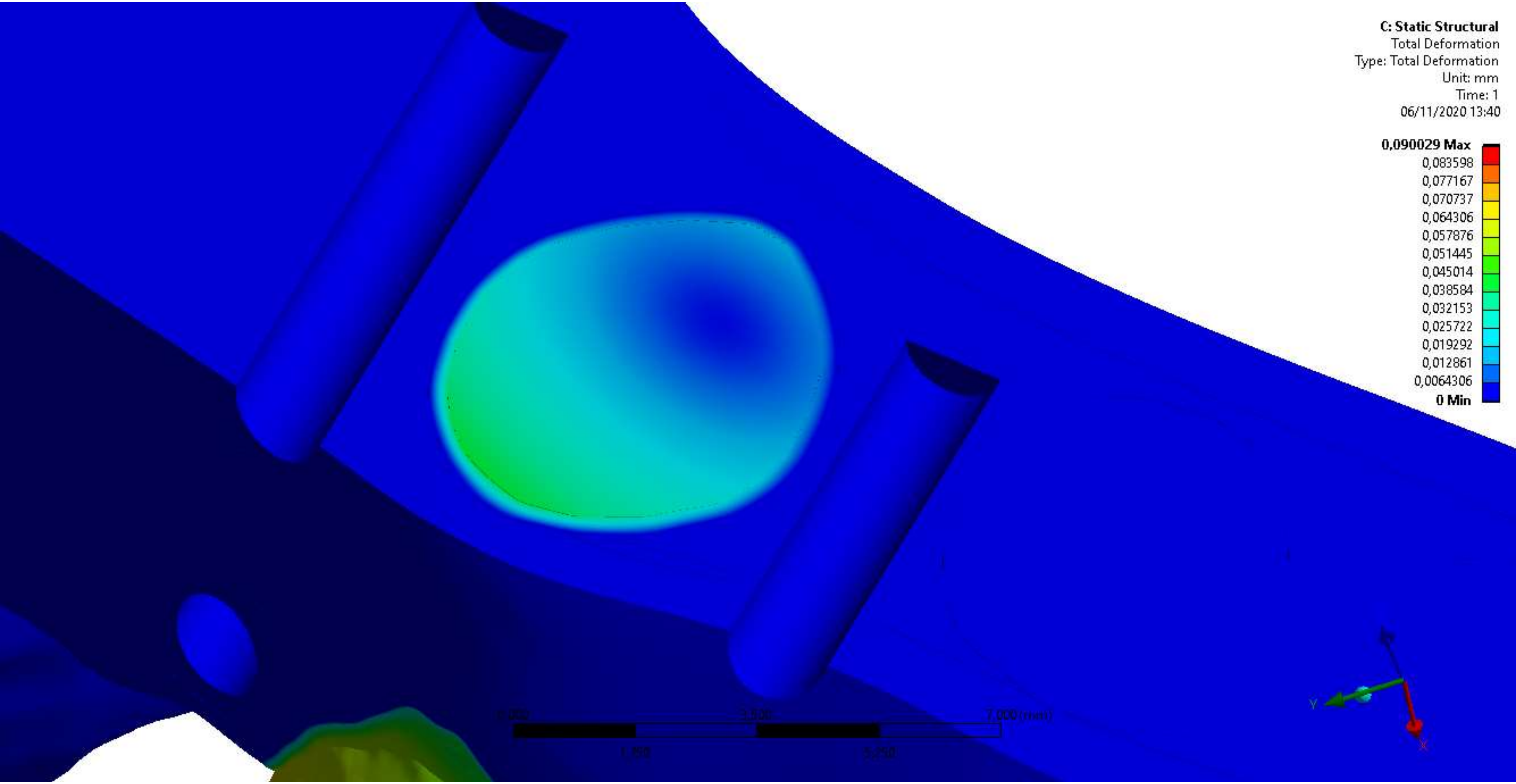
C: Static Structural
Total Deformation
Type: Total Deformation
Unit: mm
Time: 1
06/11/2020 13:40
0,090029 Max
0,083598
0,077167
0,070737
0,064306
0,057876
0,051445
0,045014
0,038584
0,032153
0,025722
0,019292
0,012861
0,0064306
0 Min

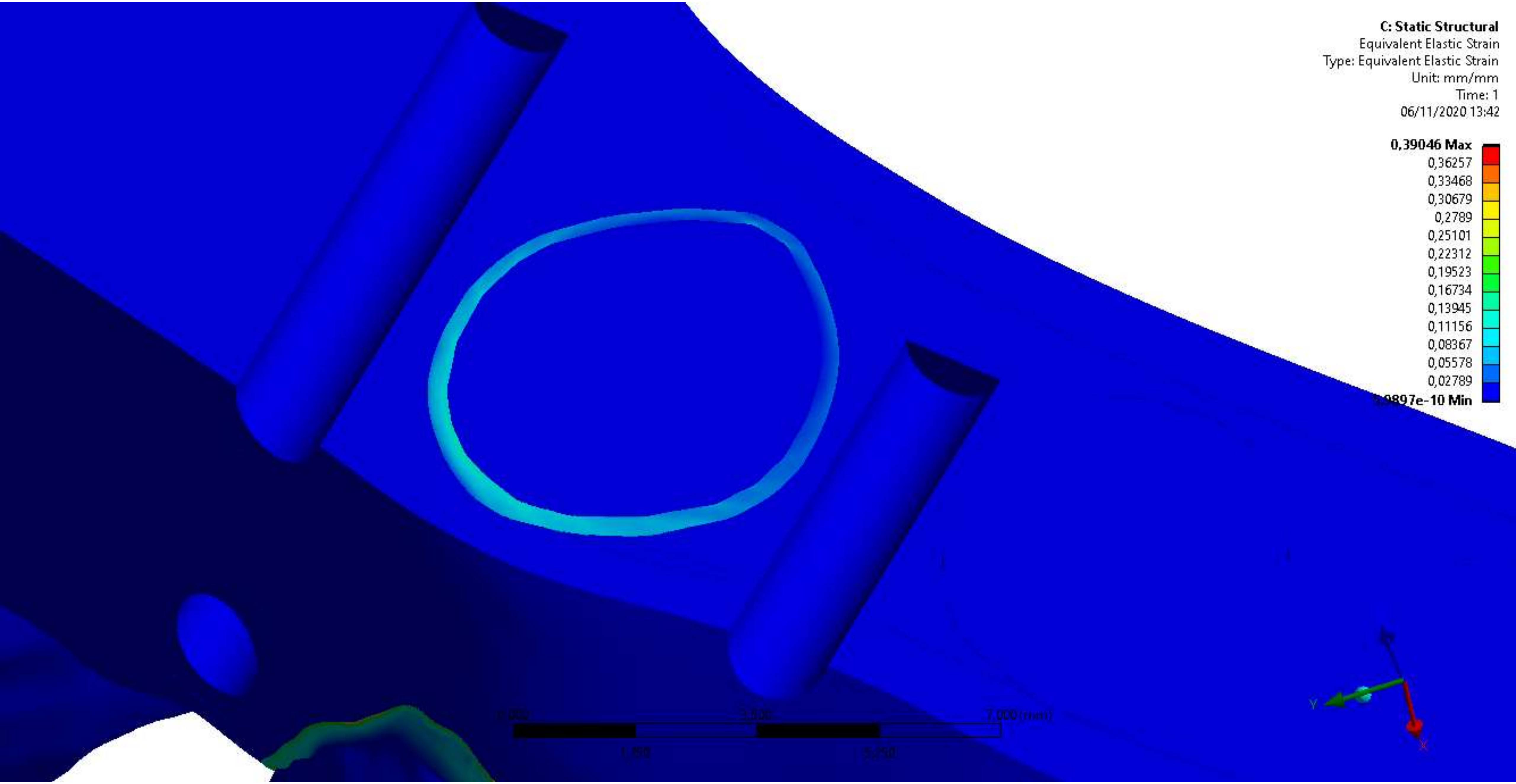
C: Static Structural
Equivalent Elastic Strain
Type: Equivalent Elastic Strain
Unit: mm/mm
Time: 1
06/11/2020 13:42
0,39046 Max
0,36257
0,33468
0,30679
0,2789
0,25101
0,22312
0,19523
0,16734
0,13945
0,11156
0,08367
0,05578
0,02789
897e-10 Min
7,000 (mm)

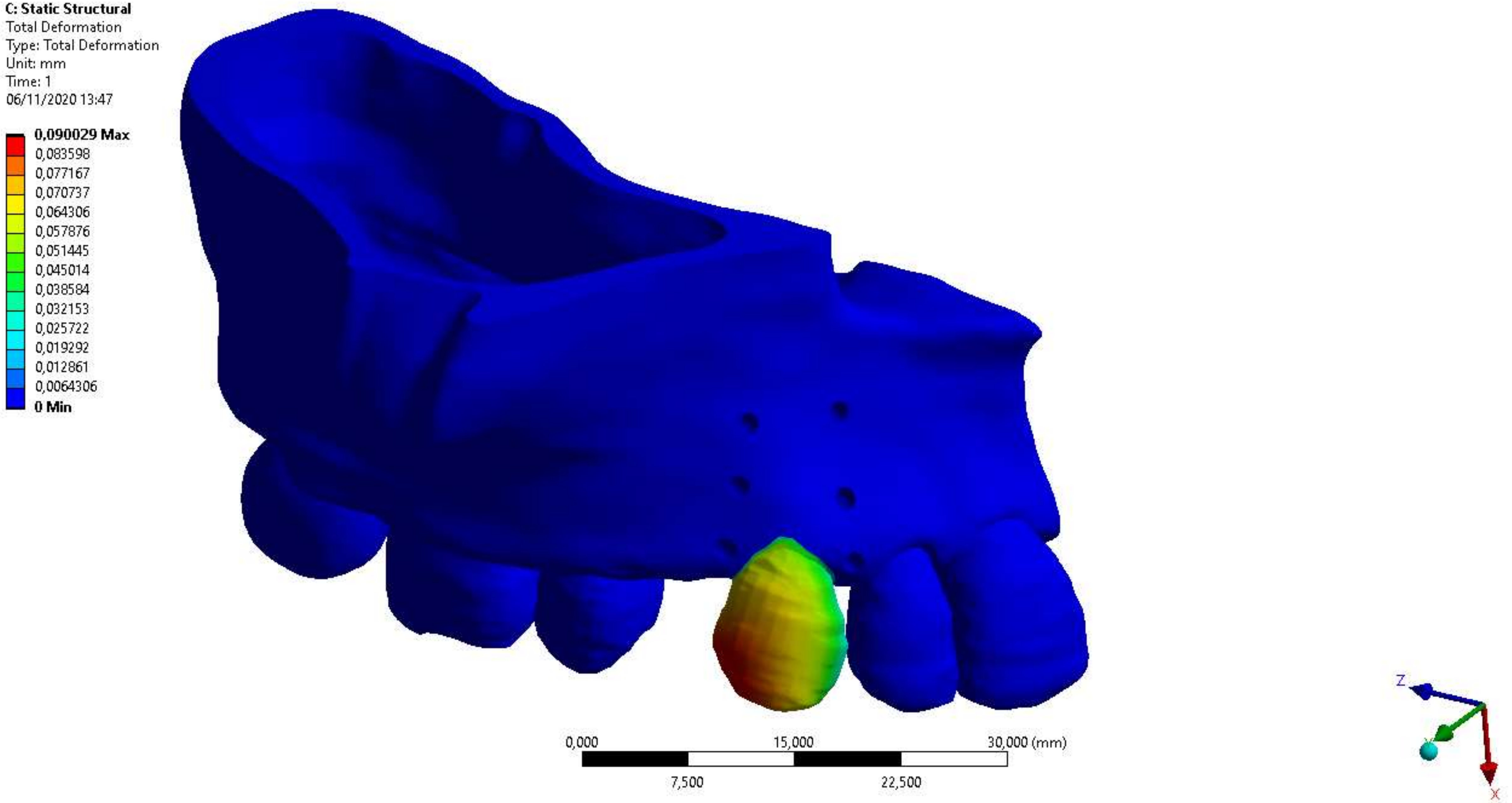
C: Static Structural
Total Deformation
Type: Total Deformation
Unit: mm
Time: 1
06/11/2020 13:47
0,090029 Max
0,083598
0,077167
0,070737
0,064306
0,057876
0,051445
0,045014
0,038584
0,032153
0,025722
0,019292
0,012861
0,0064306
0 Min
0,000
15,000
30,000 (mm)
7,500
22,500
Z
X

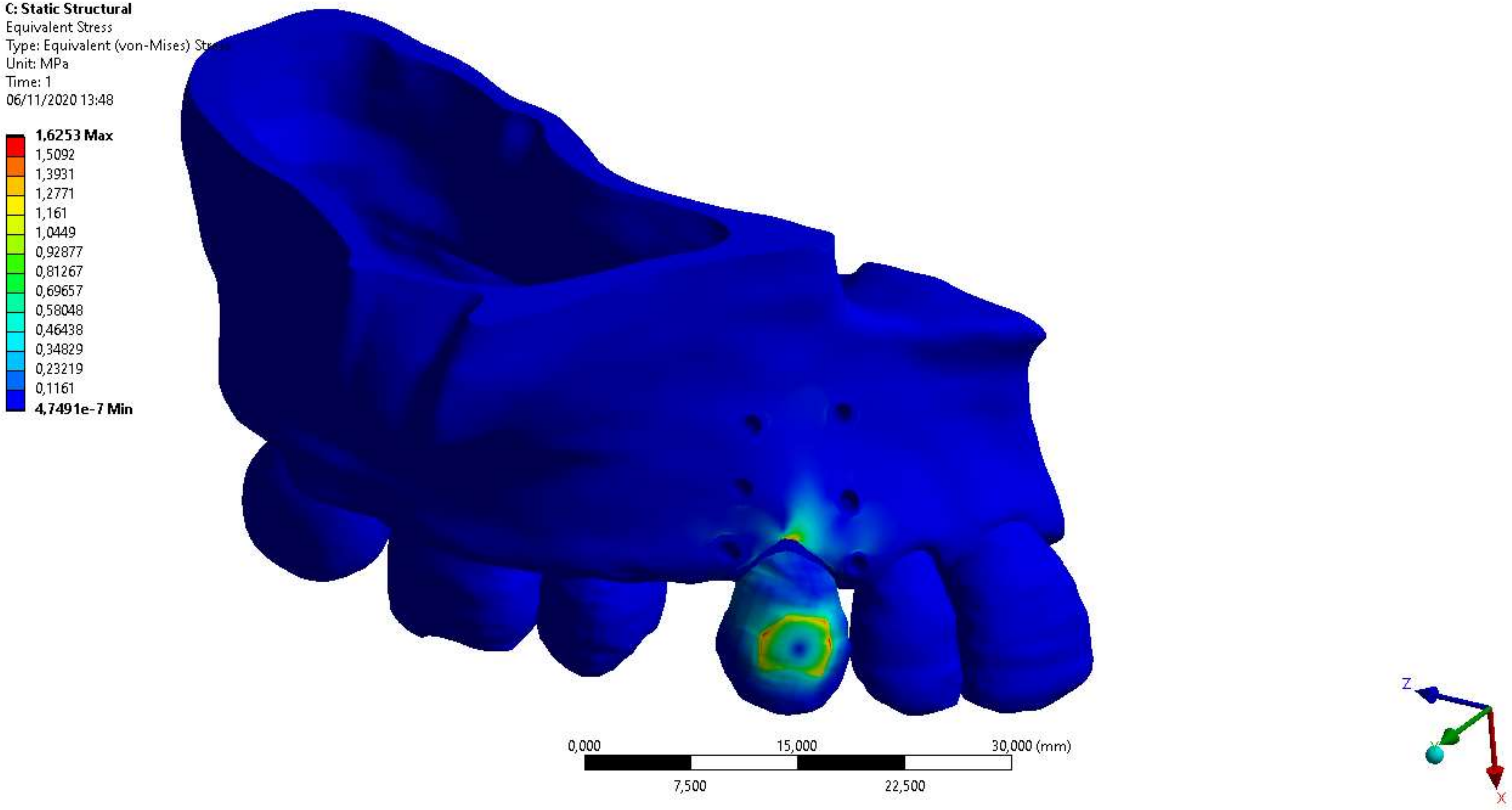

C: Static Structural
Equivalent Stress
Type: Equivalent (von-Mises) Stress
Unit: MPa
Time: 1
06/11/2020 13:48
1,6253 Max
1,5092
1,3931
1,2771
1,161
1,0449
0,92877
0,81267
0,69657
0,58048
0,46438
0,34829
0,23219
0,1161
4,7491e-7 Min
0,000
15,000
30,000 (mm)
7,500
22,500
Z
Y
X

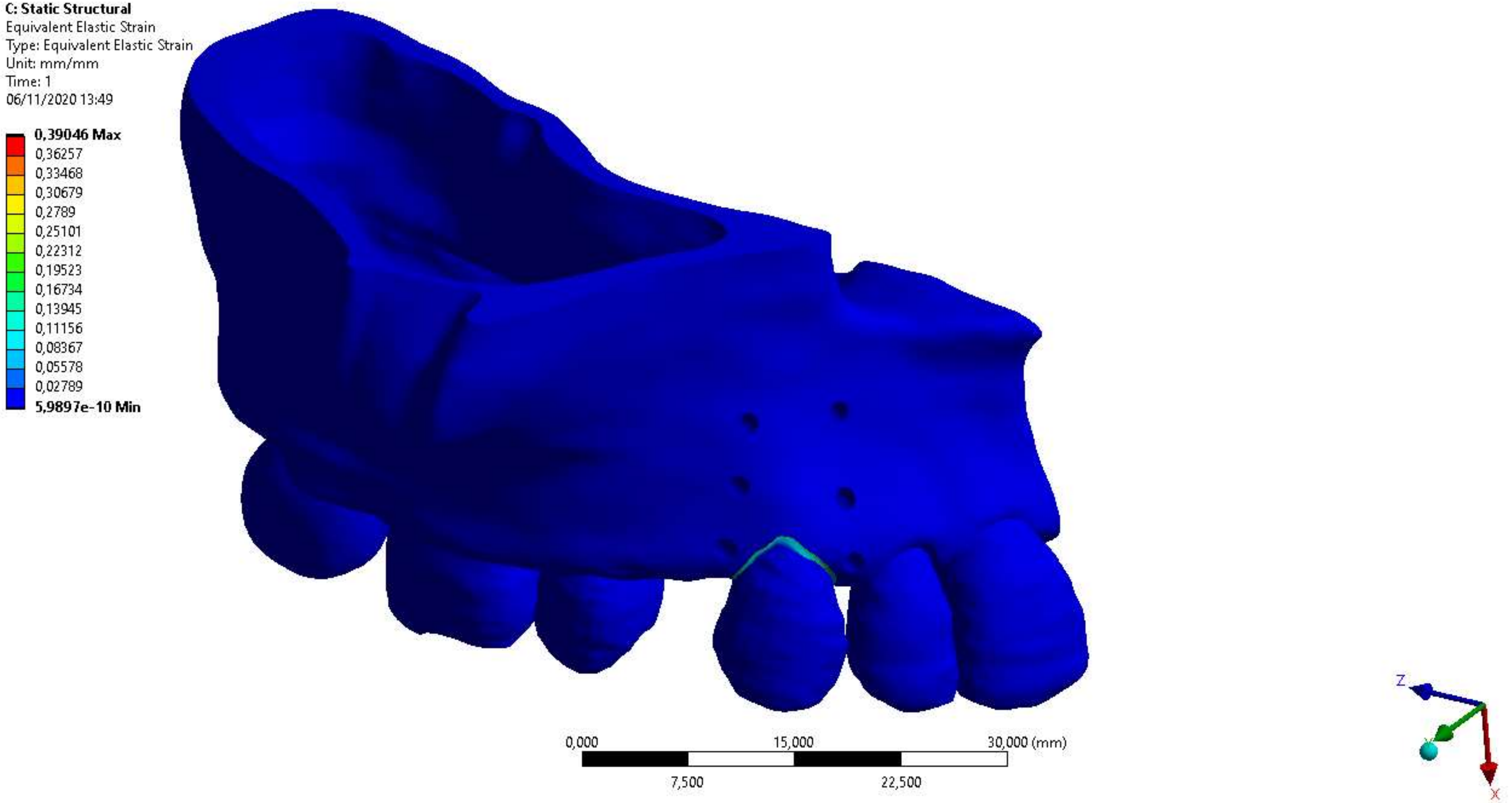
C: Static Structural
Equivalent Elastic Strain
Type: Equivalent Elastic Strain
Unit: mm/mm
Time: 1
06/11/2020 13:49
0,39046 Max
0,36257
0,33468
0,30679
0,2789
0,25101
0,22312
0,19523
0,16734
0,13945
0,11156
0,08367
0,05578
0,02789
5,9897e-10 Min
0,000
15,000
30,000 (mm)
7,500
22,500
Z
Y
X

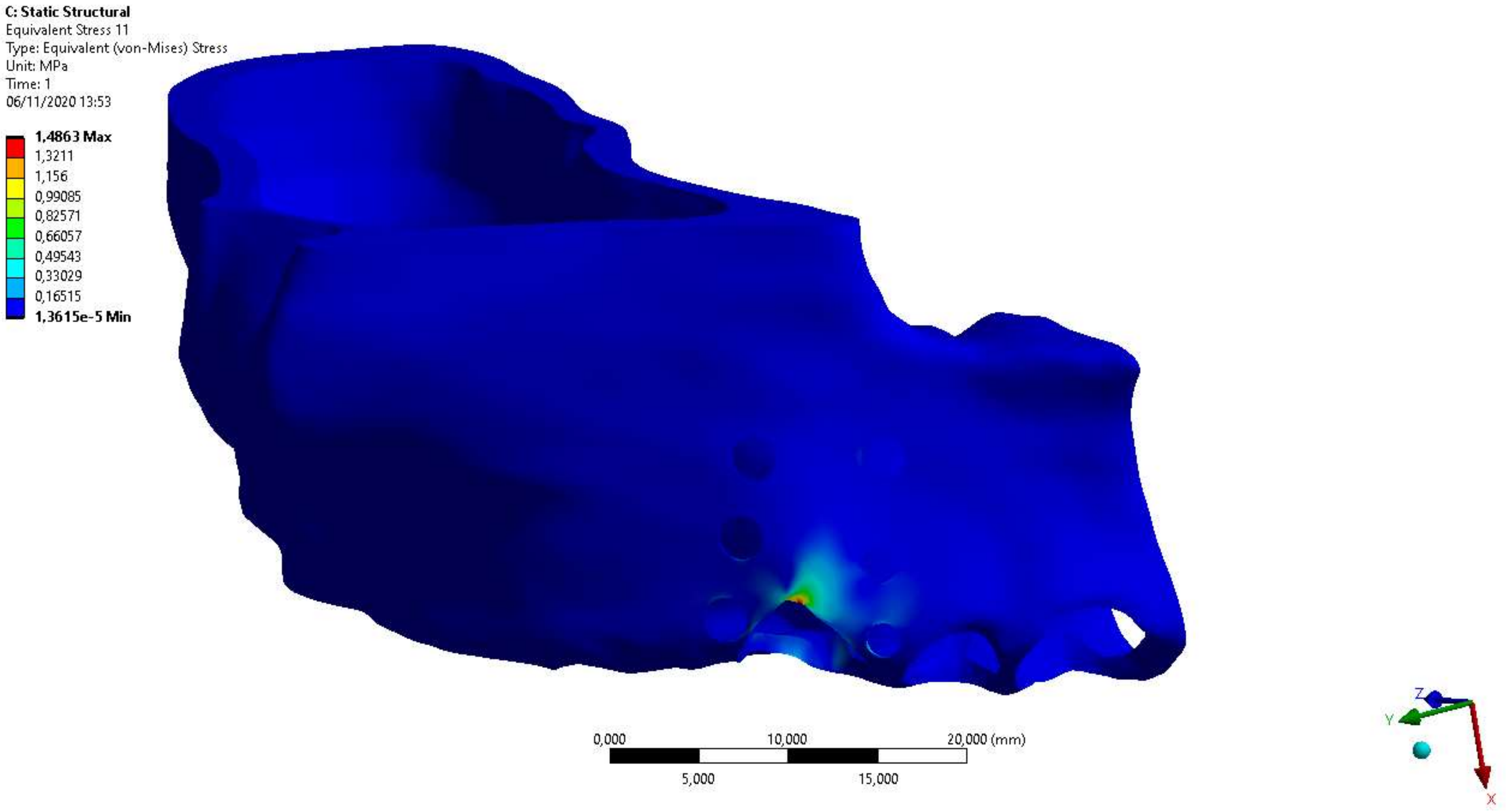
C: Static Structural
Equivalent Stress 11
Type: Equivalent (von-Mises) Stress
Unit: MPa
Time: 1
06/11/2020 13:53
1,4863 Max
1,3211
1,156
0,99085
0,82571
0,66057
0,49543
0,33029
0,16515
1,3615e-5 Min
0,000
10,000
20,000 (mm)
5,000
15,000
Z
Y
X

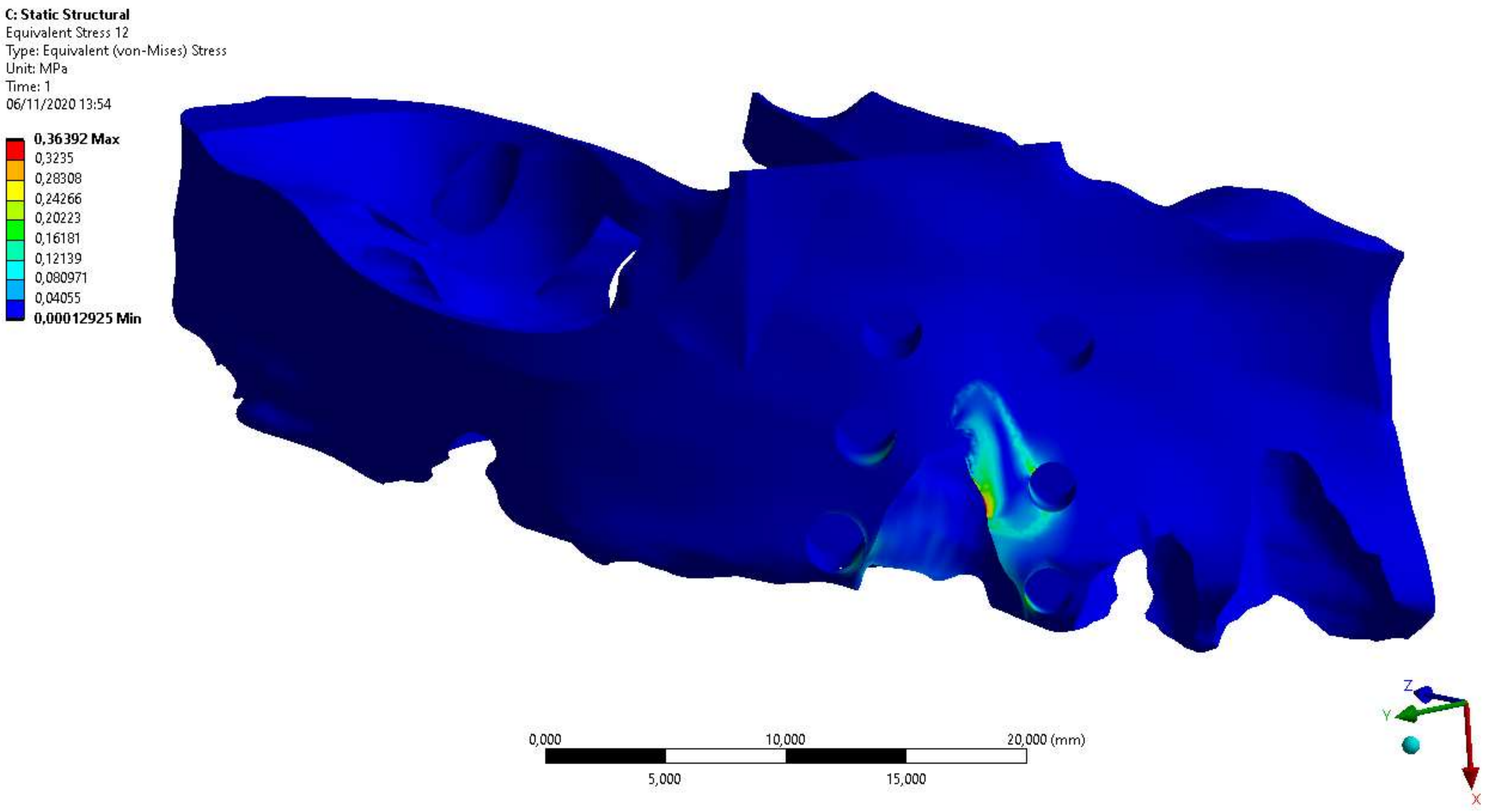
C: Static Structural
Equivalent Stress 12
Type: Equivalent (von-Mises) Stress
Unit: MPa
Time: 1
06/11/2020 13:54
0,36392 Max
0,3235
0,28308
0,24266
0,20223
0,16181
0,12139
0,080971
0,04055
0,00012925 Min
0,000
10,000
20,000 (mm)
5,000
15,000
Z
Y
X

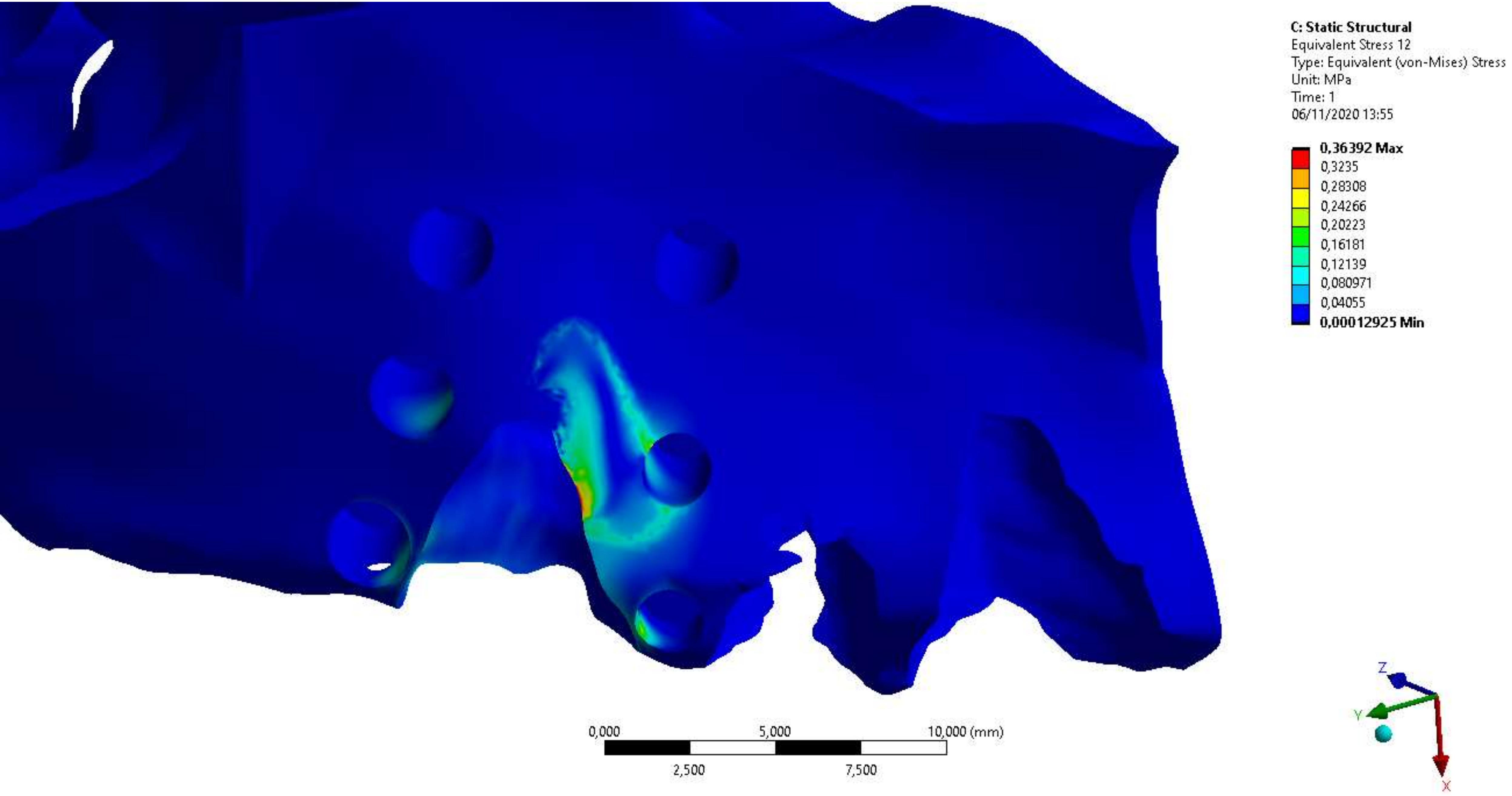

C: Static Structural
Equivalent Stress 12
Type: Equivalent (von-Mises) Stress
Unit: MPa
Time: 1
06/11/2020 13:55
0,36392 Max
0,3235
0,28308
0,24266
0,20223
0,16181
0,12139
0,080971
0,04055
0,00012925 Min
0,000
5,000
10,000 (mm)
2,500
7,500
Z
Y
X

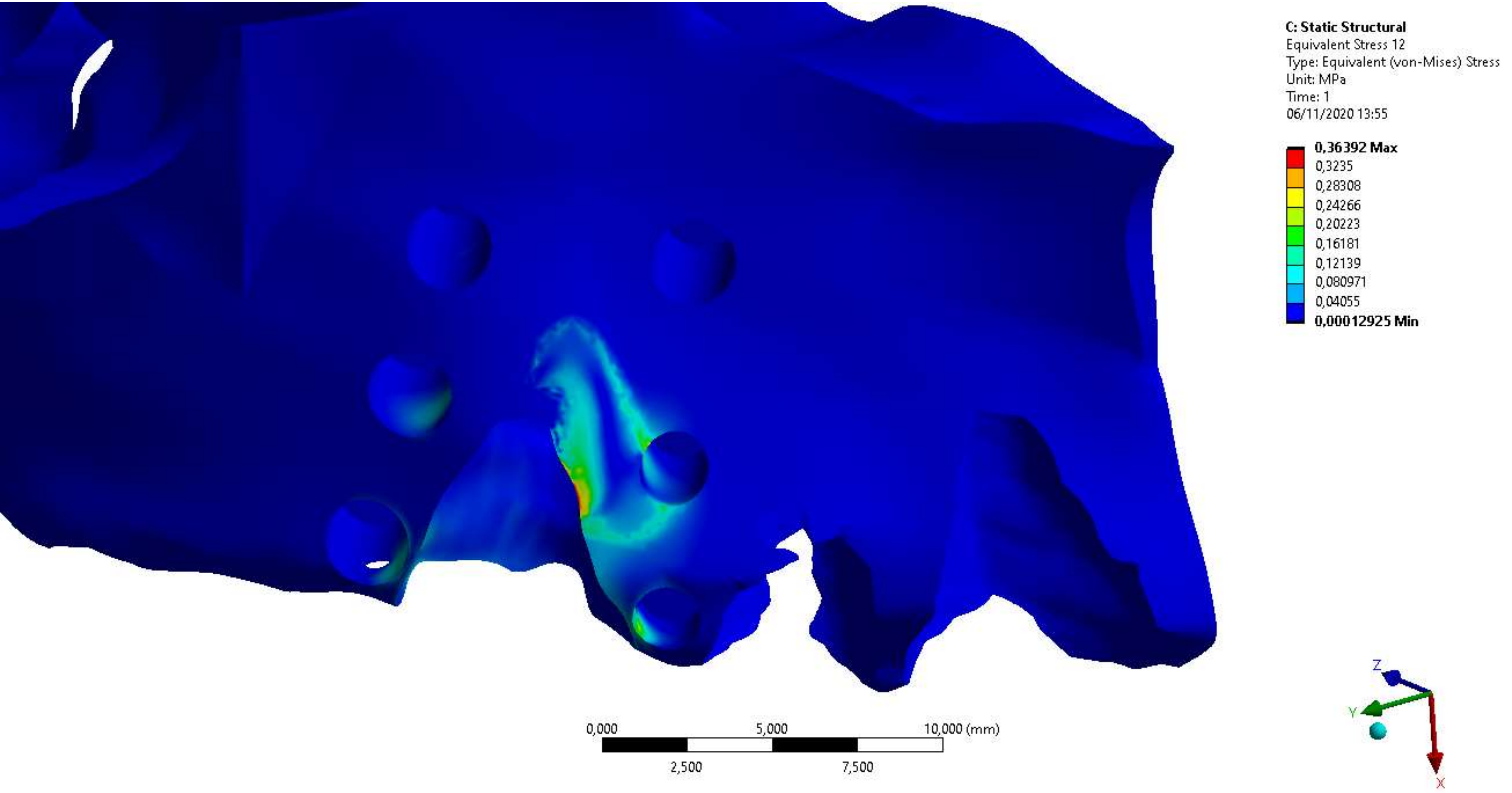
C: Static Structural
Equivalent Stress 12
Type: Equivalent (von-Mises) Stress
Unit: MPa
Time: 1
06/11/2020 13:55
0,36392 Max
0,3235
0,28308
0,24266
0,20223
0,16181
0,12139
0,080971
0,04055
0,00012925 Min
0,000
5,000
10,000 (mm)
2,500
7,500
Z
Y
X

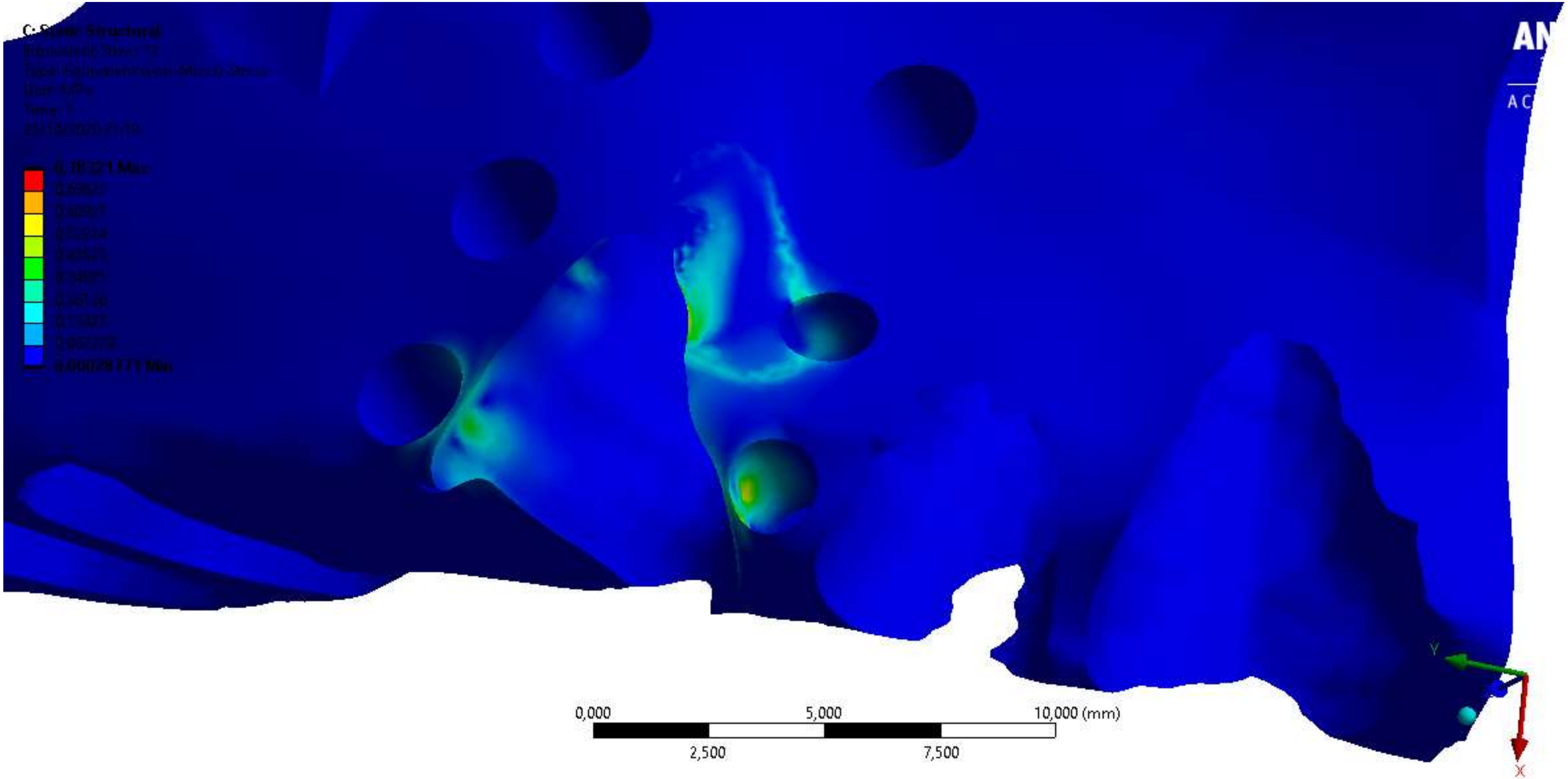
0,000
5,000
10,000 (mm)
2,500
7,500
Y
X

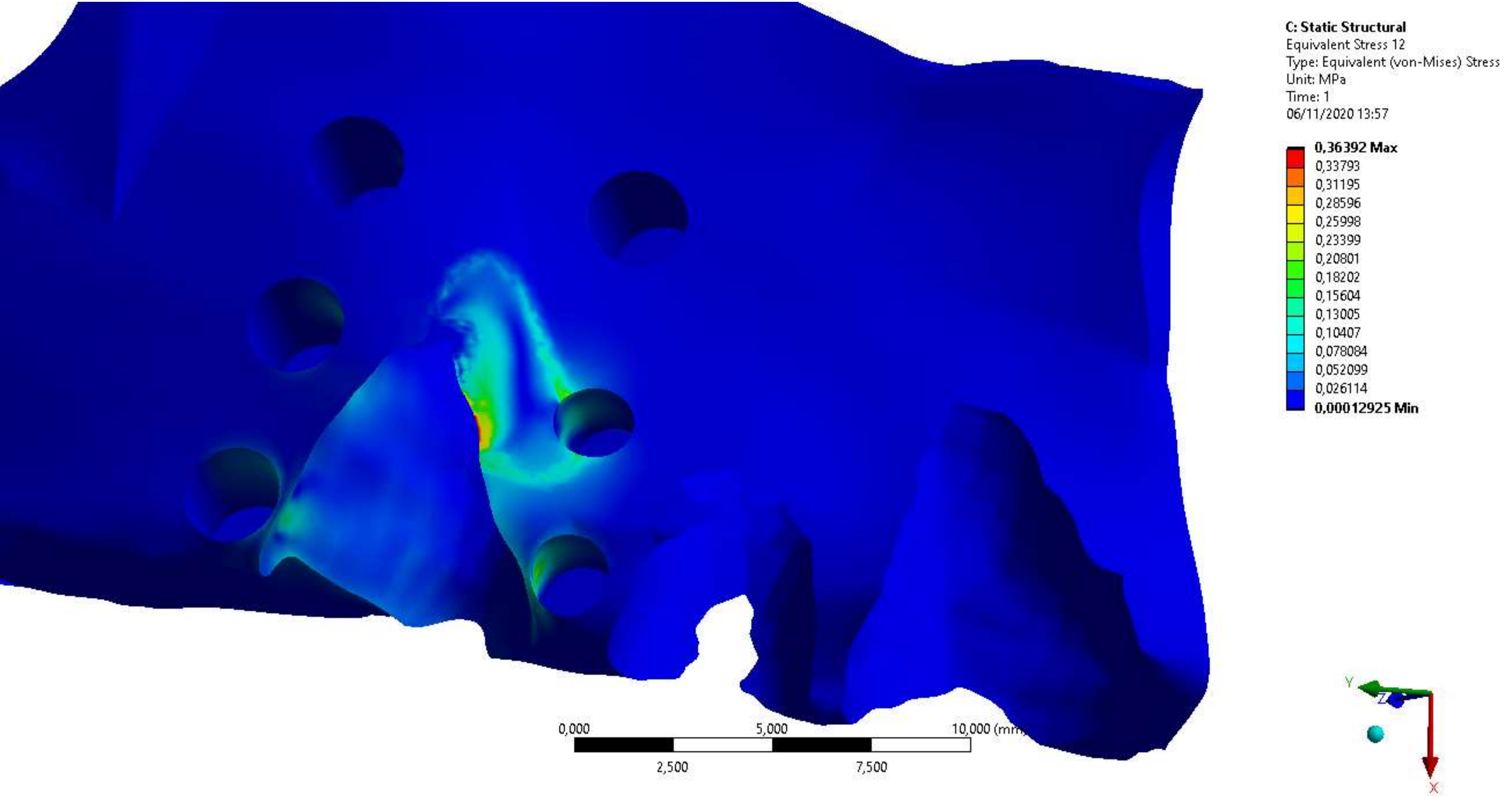

C: Static Structural
Equivalent Stress 12
Type: Equivalent (von-Mises) Stress
Unit: MPa
Time: 1
06/11/2020 13:57
0,36392 Max
0,33793
0,31195
0,28596
0,25998
0,23399
0,20801
0,18202
0,15604
0,13005
0,10407
0,078084
0,052099
0,026114
0,00012925 Min
0,000
5,000
10,000 (mm)
2,500
7,500
Y
Z
X

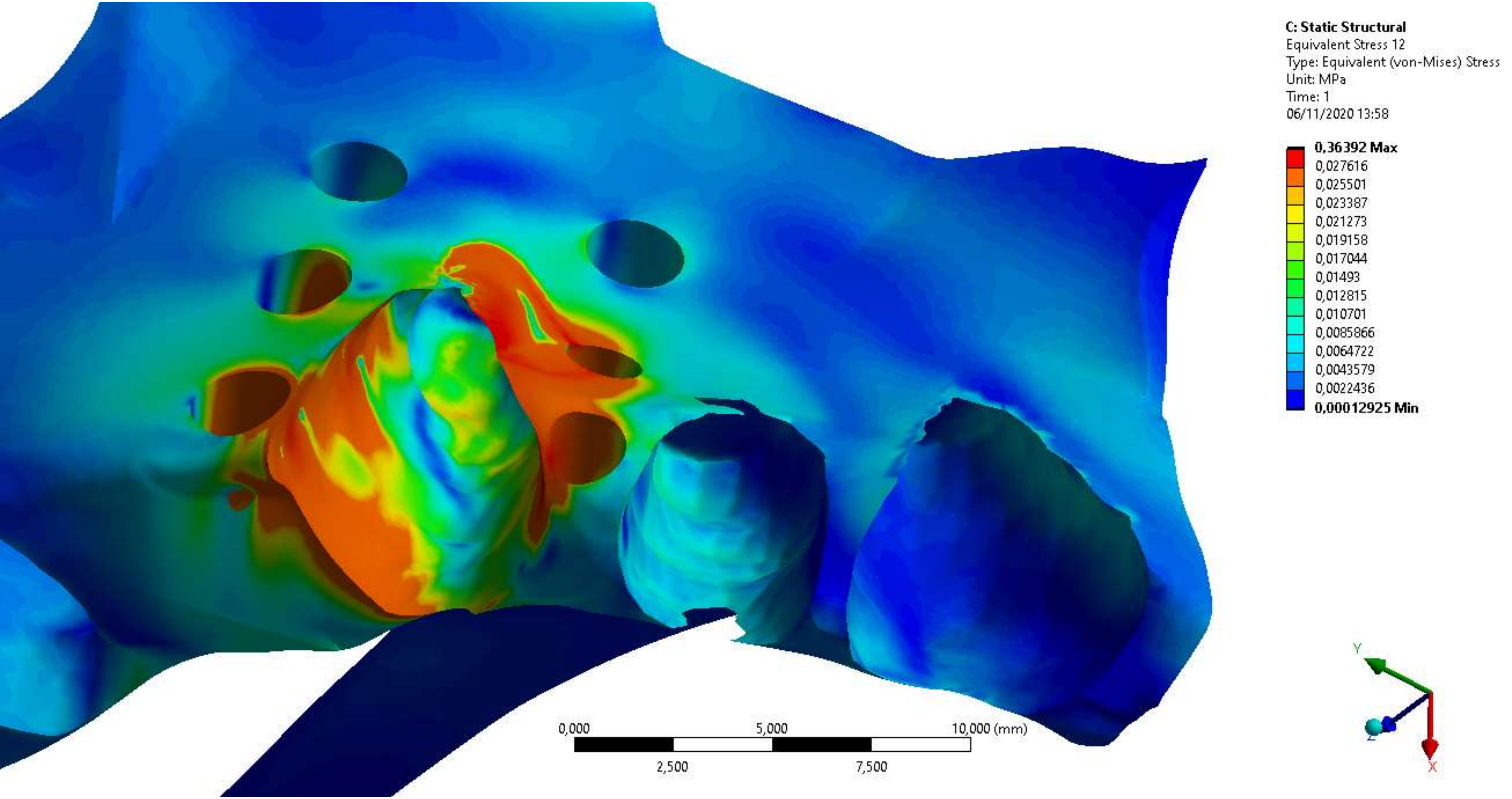

C: Static Structural
Equivalent Stress 12
Type: Equivalent (von-Mises) Stress
Unit: MPa
Time: 1
06/11/2020 13:58
0,36392 Max
0,027616
0,025501
0,023387
0,021273
0,019158
0,017044
0,01493
0,012815
0,010701
0,0085866
0,0064722
0,0043579
0,0022436
0,00012925 Min
0,000
5,000
10,000 (mm)
2,500
7,500
Y
Z
X

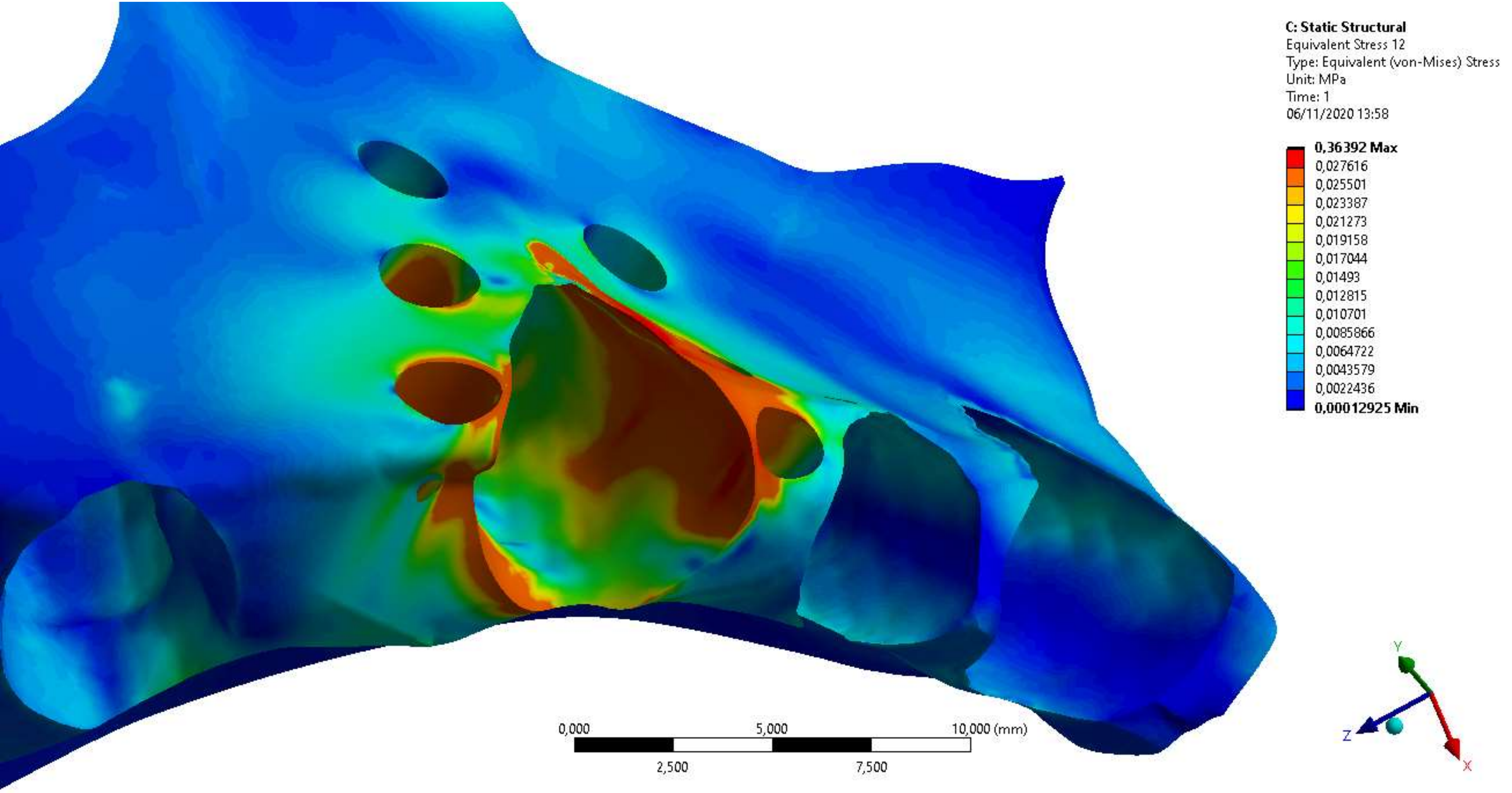

C: Static Structural
Equivalent Stress 12
Type: Equivalent (von-Mises) Stress
Unit: MPa
Time: 1
06/11/2020 13:58
0,36392 Max
0,027616
0,025501
0,023387
0,021273
0,019158
0,017044
0,01493
0,012815
0,010701
0,0085866
0,0064722
0,0043579
0,0022436
0,00012925 Min
0,000
5,000
10,000 (mm)
2,500
7,500
Y
Z
X

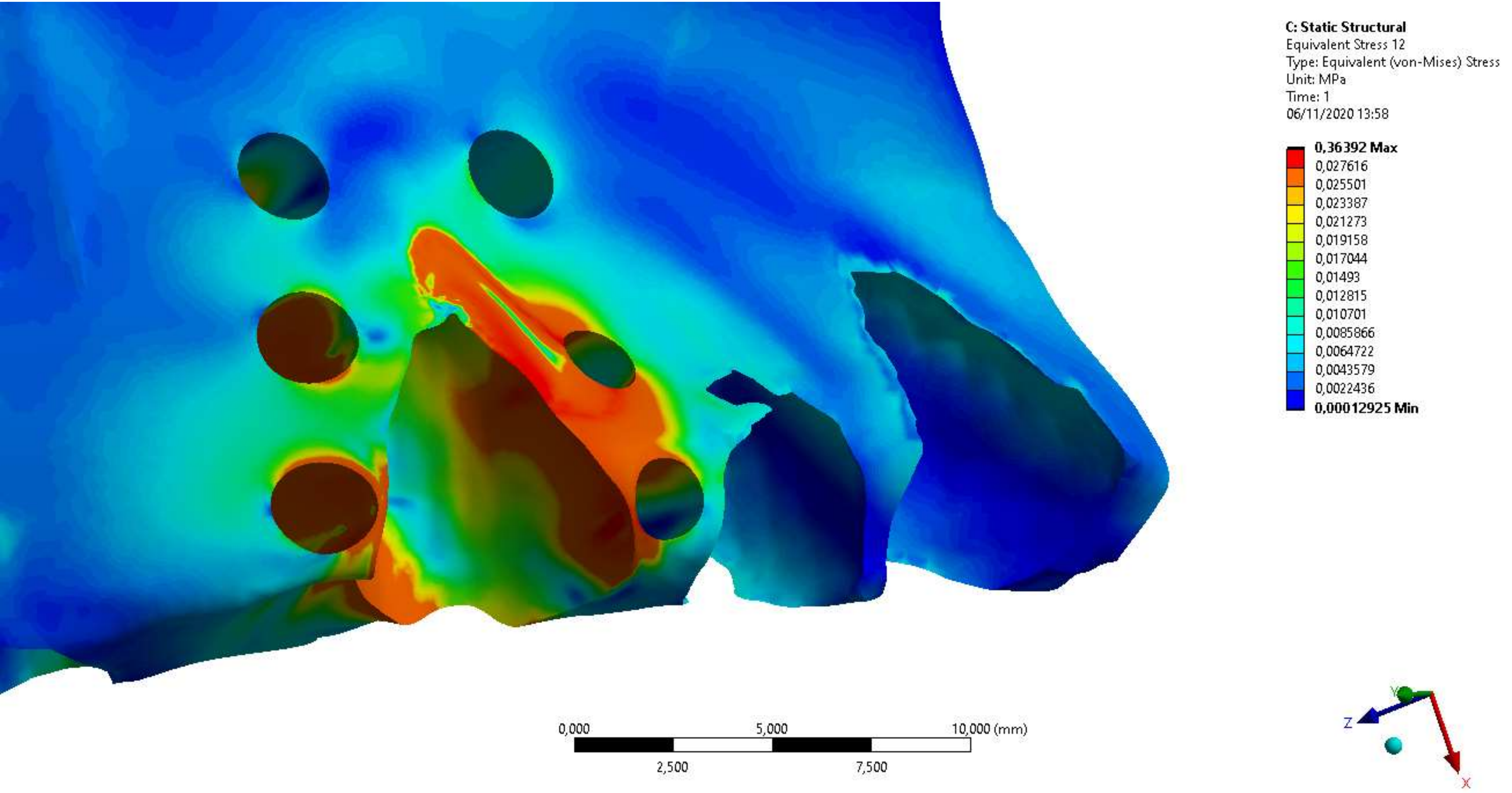

C: Static Structural
Equivalent Stress 12
Type: Equivalent (von-Mises) Stress
Unit: MPa
Time: 1
06/11/2020 13:58
0,36392 Max
0,027616
0,025501
0,023387
0,021273
0,019158
0,017044
0,01493
0,012815
0,010701
0,0085866
0,0064722
0,0043579
0,0022436
0,00012925 Min
0,000
5,000
10,000 (mm)
2,500
7,500
Y
Z
X

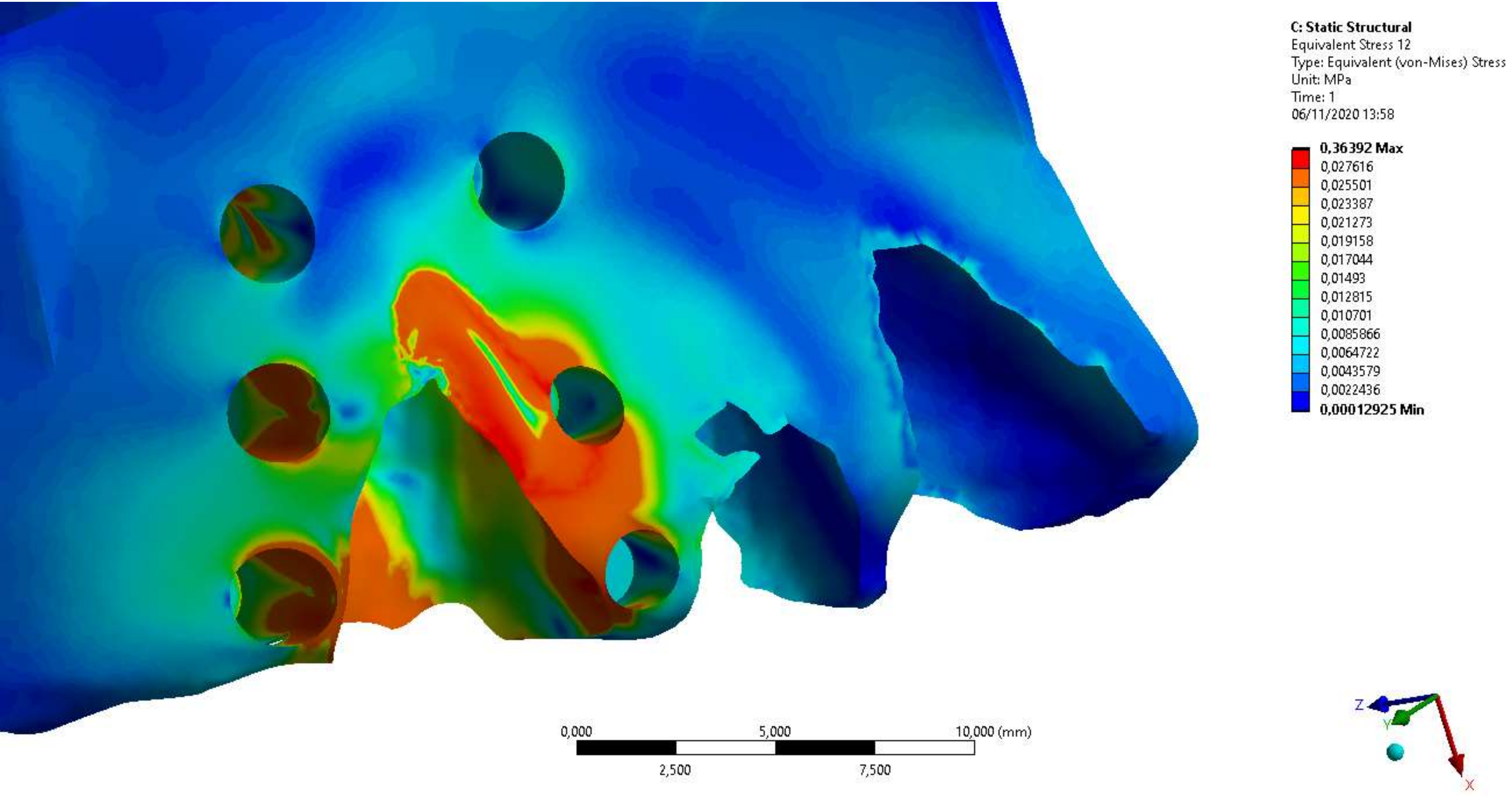
C: Static Structural
Equivalent Stress 12
Type: Equivalent (von-Mises) Stress
Unit: MPa
Time: 1
06/11/2020 13:58
0,36392 Max
0,027616
0,025501
0,023387
0,021273
0,019158
0,017044
0,01493
0,012815
0,010701
0,0085866
0,0064722
0,0043579
0,0022436
0,00012925 Min
0,000
5,000
10,000 (mm)
2,500
7,500
Z
Y
X

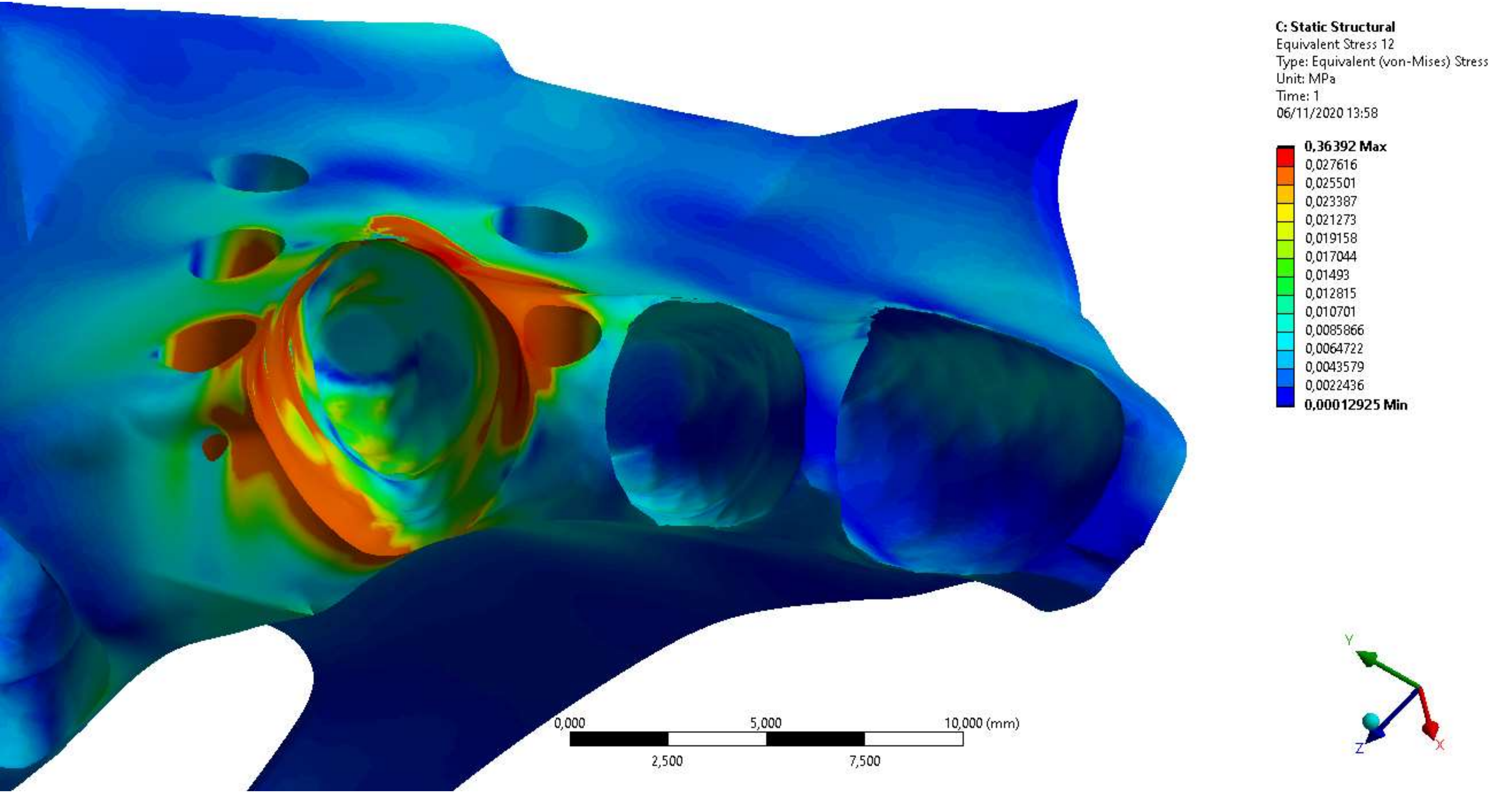
C: Static Structural
Equivalent Stress 12
Type: Equivalent (von-Mises) Stress
Unit: MPa
Time: 1
06/11/2020 13:58
0,36392 Max
0,027616
0,025501
0,023387
0,021273
0,019158
0,017044
0,01493
0,012815
0,010701
0,0085866
0,0064722
0,0043579
0,0022436
0,00012925 Min
0,000
5,000
10,000 (mm)
2,500
7,500
Y
Z
X

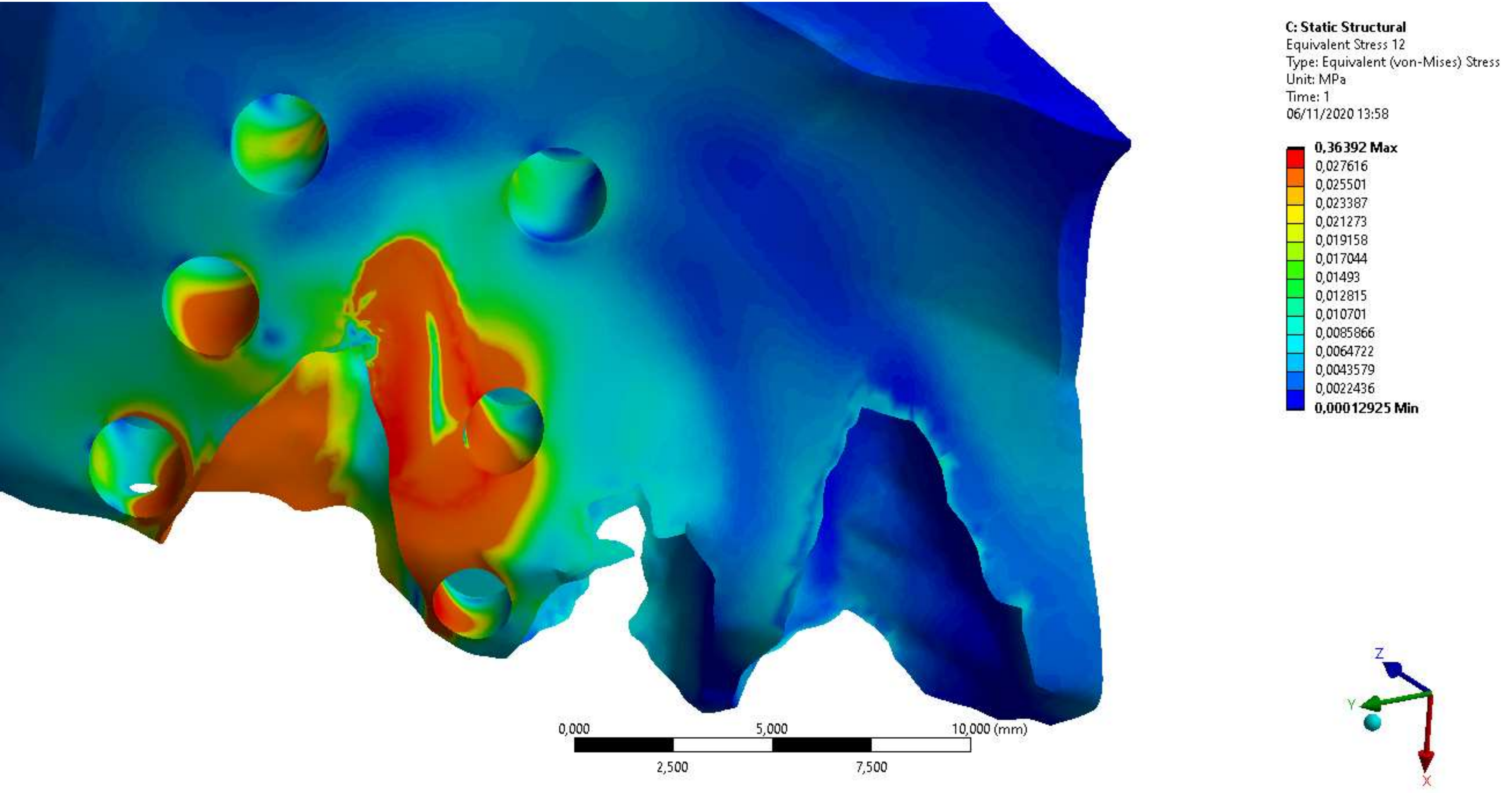

C: Static Structural
Equivalent Stress 12
Type: Equivalent (von-Mises) Stress
Unit: MPa
Time: 1
06/11/2020 13:58
0,36392 Max
0,027616
0,025501
0,023387
0,021273
0,019158
0,017044
0,01493
0,012815
0,010701
0,0085866
0,0064722
0,0043579
0,0022436
0,00012925 Min
0,000
5,000
10,000 (mm)
2,500
7,500
Z
Y
X

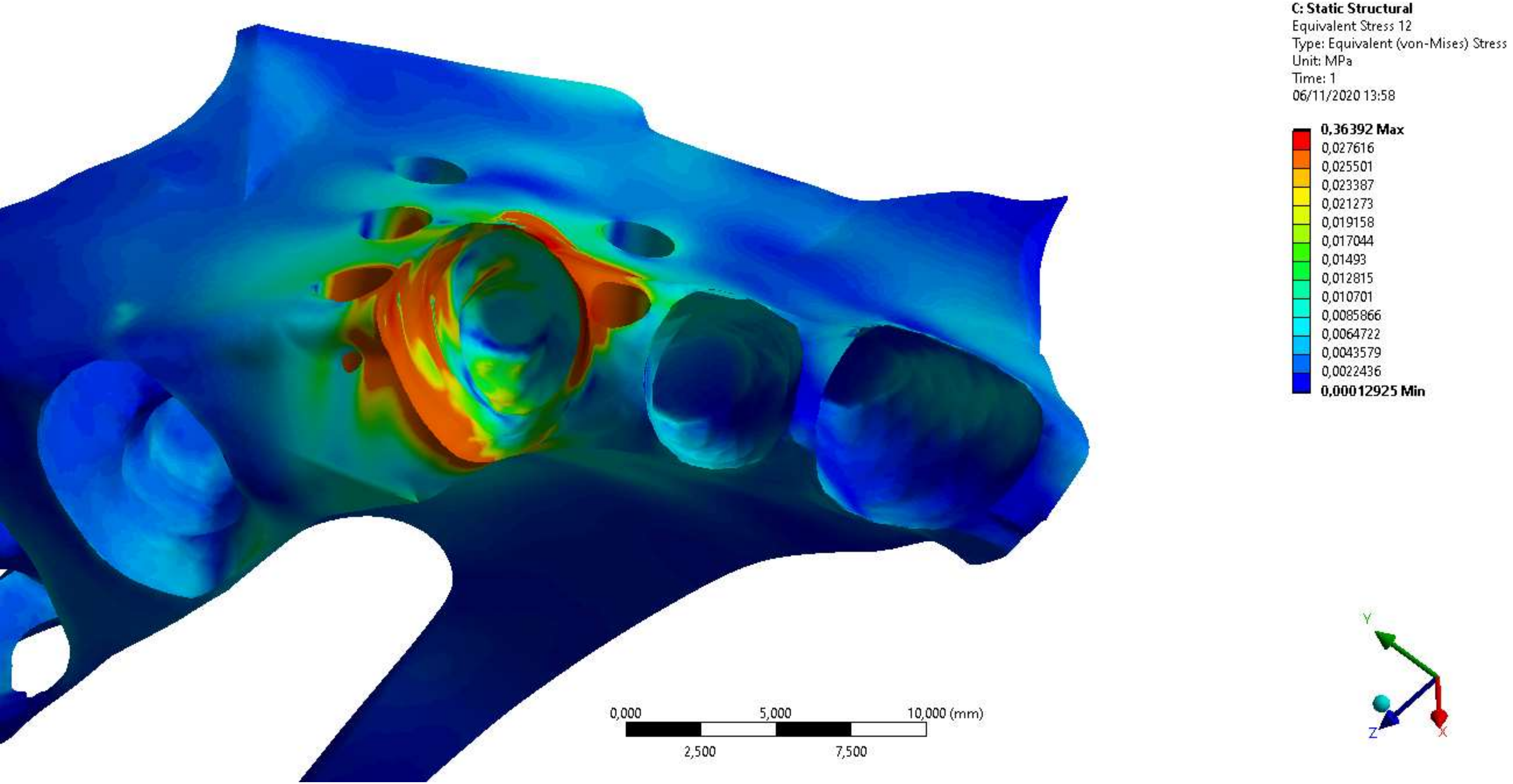
C: Static Structural
Equivalent Stress 12
Type: Equivalent (von-Mises) Stress
Unit: MPa
Time: 1
06/11/2020 13:58
0,36392 Max
0,027616
0,025501
0,023387
0,021273
0,019158
0,017044
0,01493
0,012815
0,010701
0,0085866
0,0064722
0,0043579
0,0022436
0,00012925 Min
0,000
5,000
10,000 (mm)
2,500
7,500
Y
Z
X

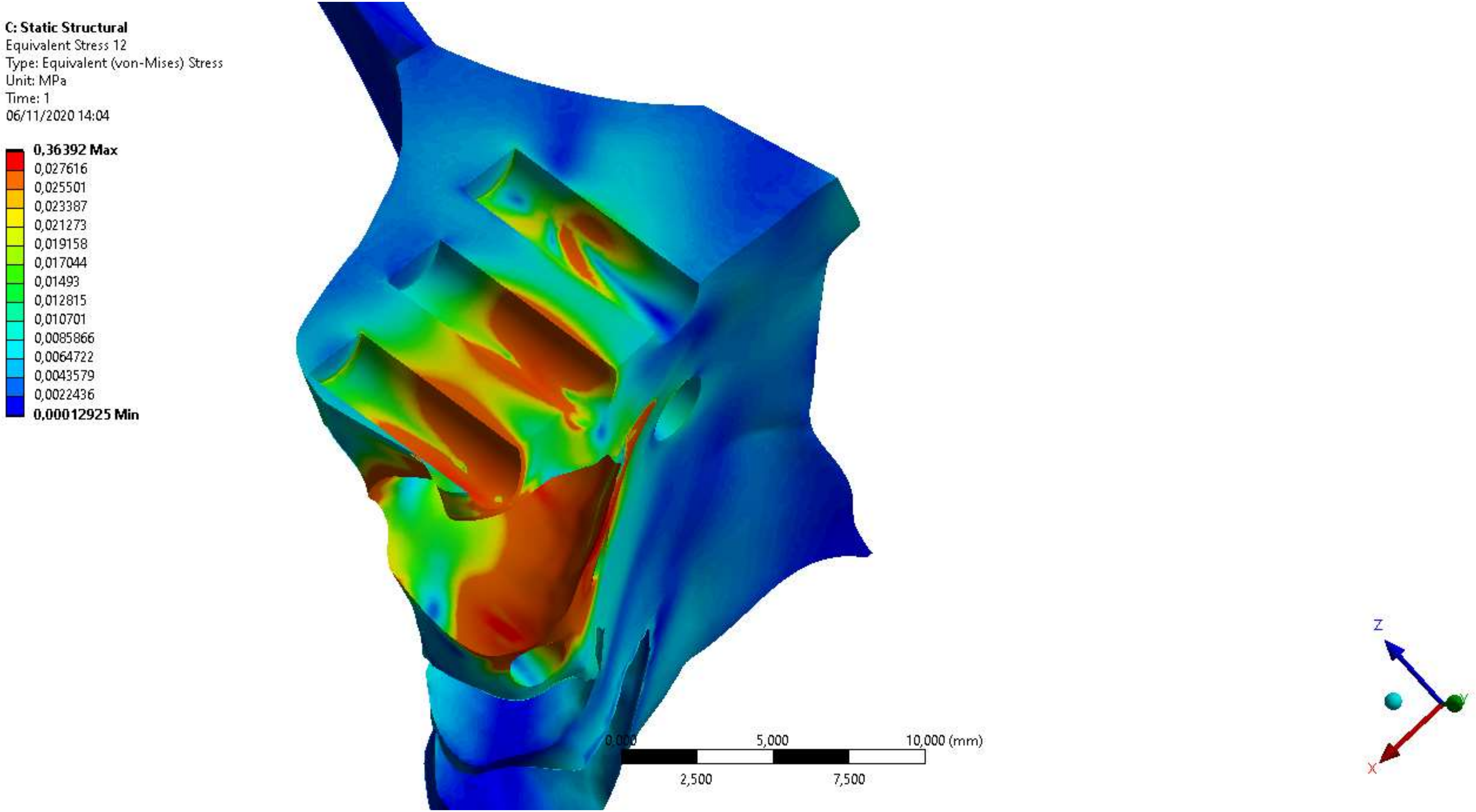
C: Static Structural
Equivalent Stress 12
Type: Equivalent (von-Mises) Stress
Unit: MPa
Time: 1
06/11/2020 14:04
0,36392 Max
0,027616
0,025501
0,023387
0,021273
0,019158
0,017044
0,01493
0,012815
0,010701
0,0085866
0,0064722
0,0043579
0,0022436
0,00012925 Min
0,000
5,000
10,000 (mm)
2,500
7,500
Z
Y
X

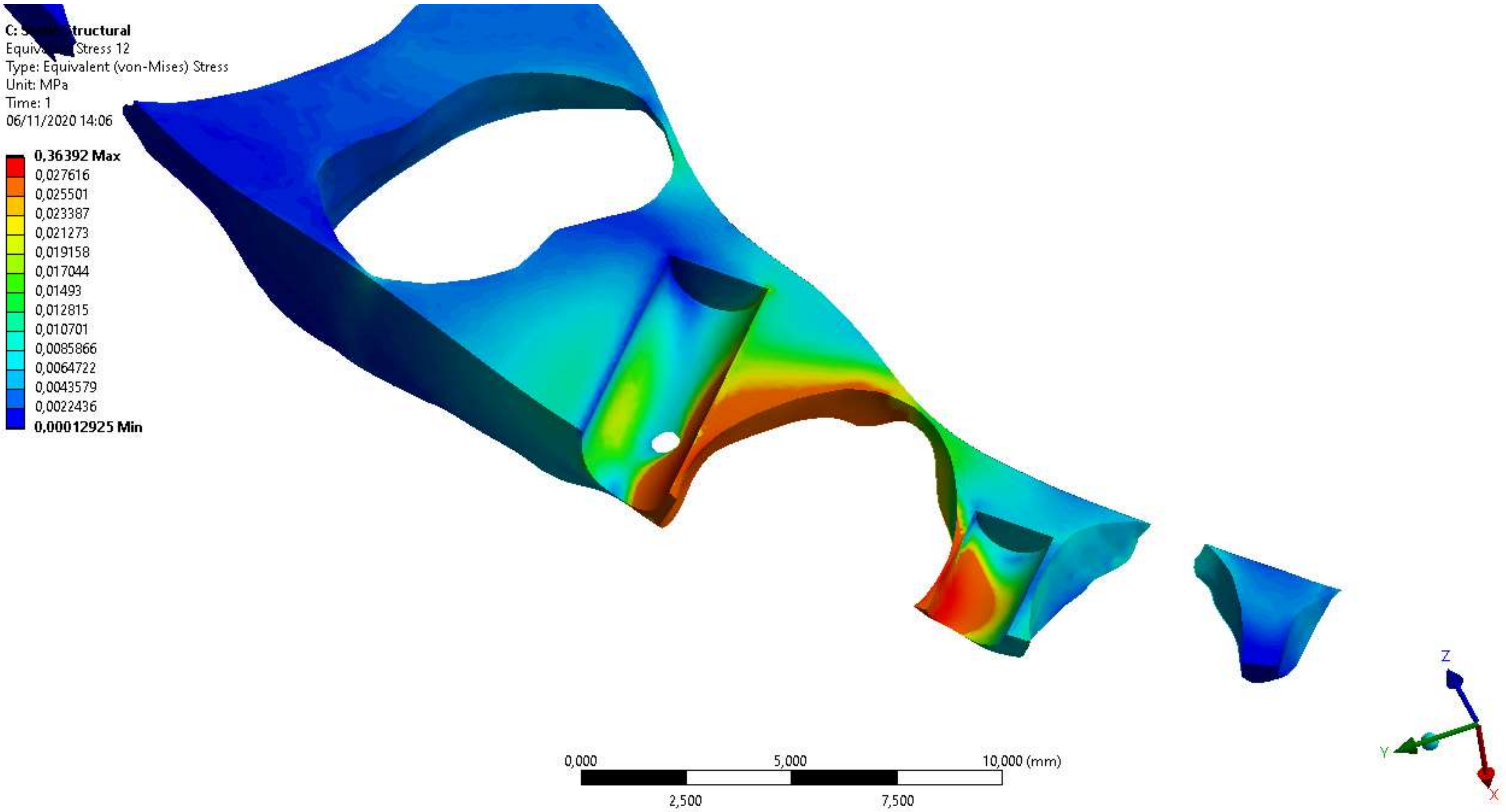

Type: Equivalent (von-Mises) Stress
Unit: MPa
Time: 1
06/11/2020 14:06
0,36392 Max
0,027616
0,025501
0,023387
0,021273
0,019158
0,017044
0,01493
0,012815
0,010701
0,0085866
0,0064722
0,0043579
0,0022436
0,00012925 Min
0,000
5,000
10,000 (mm)
2,500
7,500
Z
Y
X

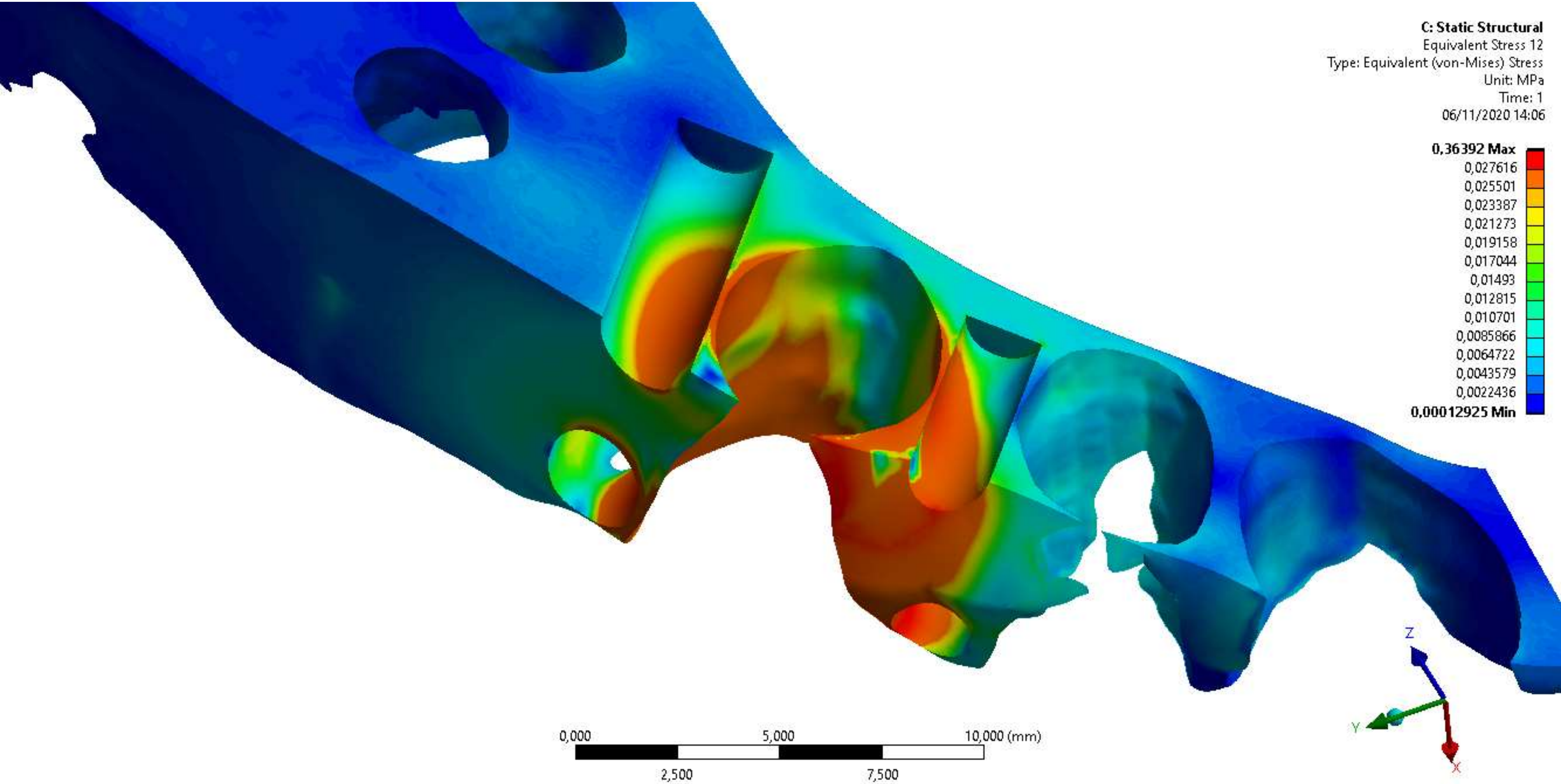
C: Static Structural
Equivalent Stress 12
Type: Equivalent (von-Mises) Stress
Unit: MPa
Time: 1
06/11/2020 14:06
0,36392 Max
0,027616
0,025501
0,023387
0,021273
0,019158
0,017044
0,01493
0,012815
0,010701
0,0085866
0,0064722
0,0043579
0,0022436
0,00012925 Min
0,000
5,000
10,000 (mm)
2,500
7,500
Z
Y
X

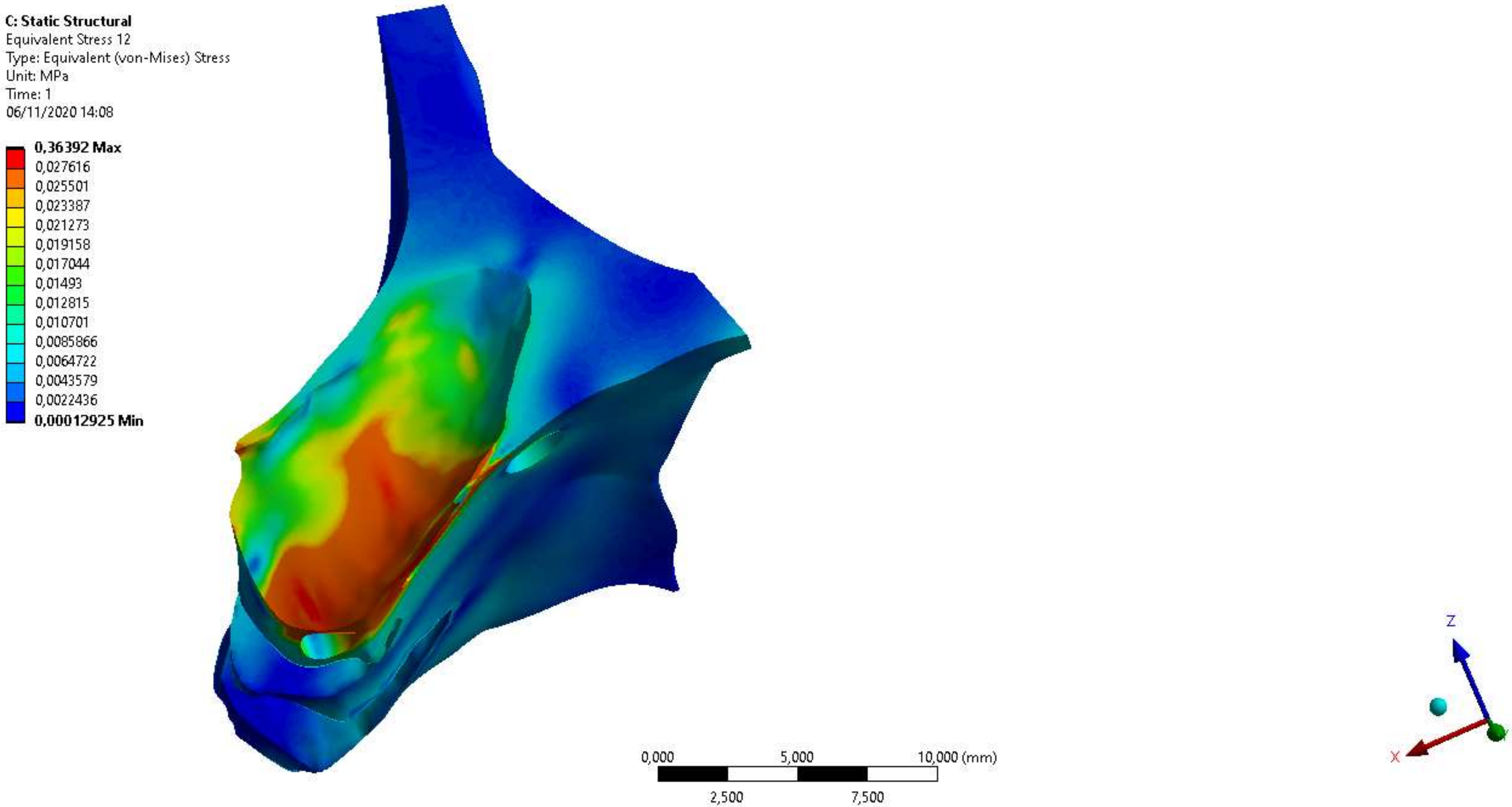
C: Static Structural
Equivalent Stress 12
Type: Equivalent (von-Mises) Stress
Unit: MPa
Time: 1
06/11/2020 14:08
0,36392 Max
0,027616
0,025501
0,023387
0,021273
0,019158
0,017044
0,01493
0,012815
0,010701
0,0085866
0,0064722
0,0043579
0,0022436
0,00012925 Min
0,000
5,000
10,000 (mm)
2,500
7,500
Z
X

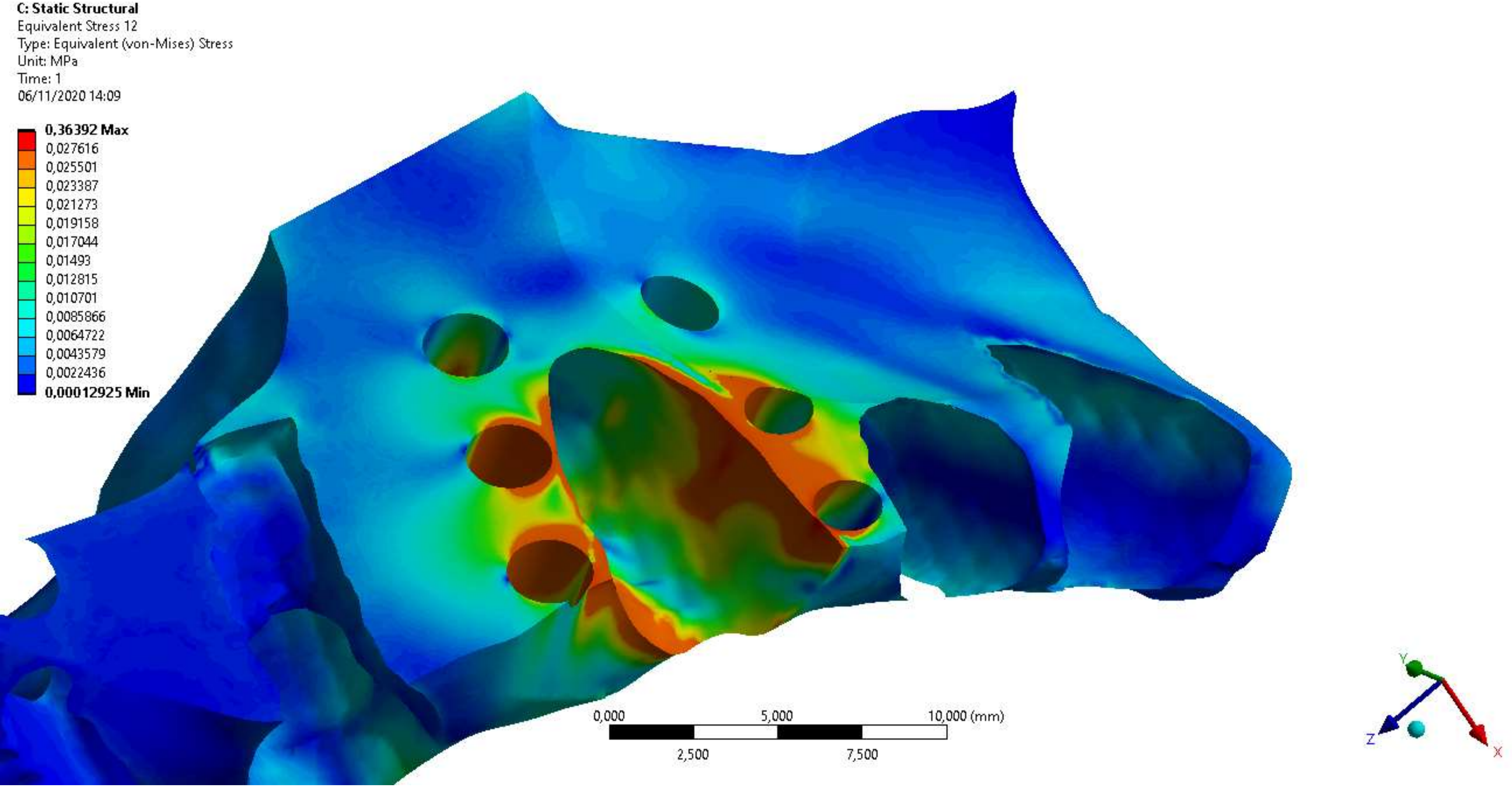
C: Static Structural
Equivalent Stress 12
Type: Equivalent (von-Mises) Stress
Unit: MPa
Time: 1
06/11/2020 14:09
0,36392 Max
0,027616
0,025501
0,023387
0,021273
0,019158
0,017044
0,01493
0,012815
0,010701
0,0085866
0,0064722
0,0043579
0,0022436
0,00012925 Min
0,000
5,000
10,000 (mm)
2,500
7,500
Y
Z
X

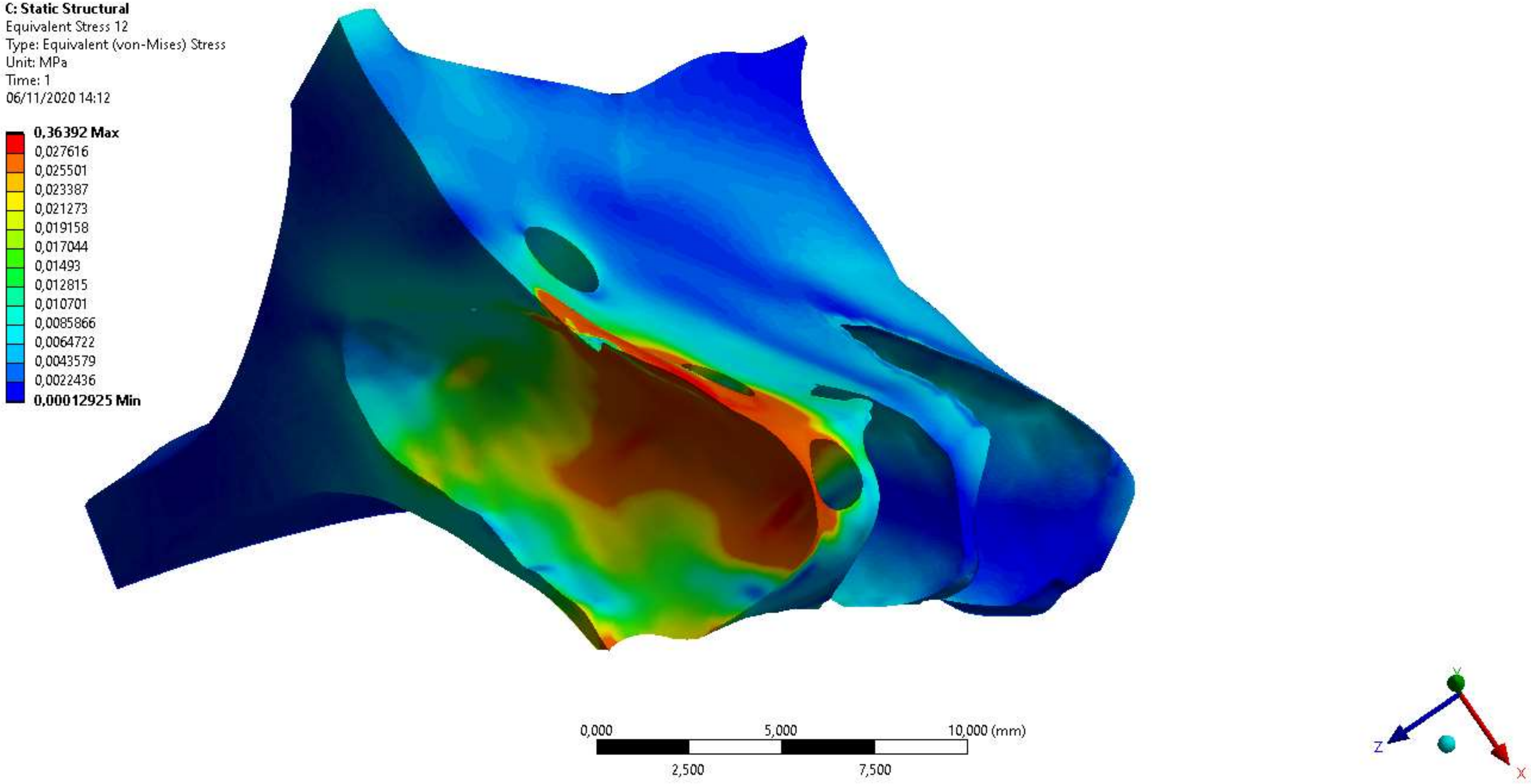
C: Static Structural
Equivalent Stress 12
Type: Equivalent (von-Mises) Stress
Unit: MPa
Time: 1
06/11/2020 14:12
0,36392 Max
0,027616
0,025501
0,023387
0,021273
0,019158
0,017044
0,01493
0,012815
0,010701
0,0085866
0,0064722
0,0043579
0,0022436
0,00012925 Min
0,000
5,000
10,000 (mm)
2,500
7,500
Y
Z
X

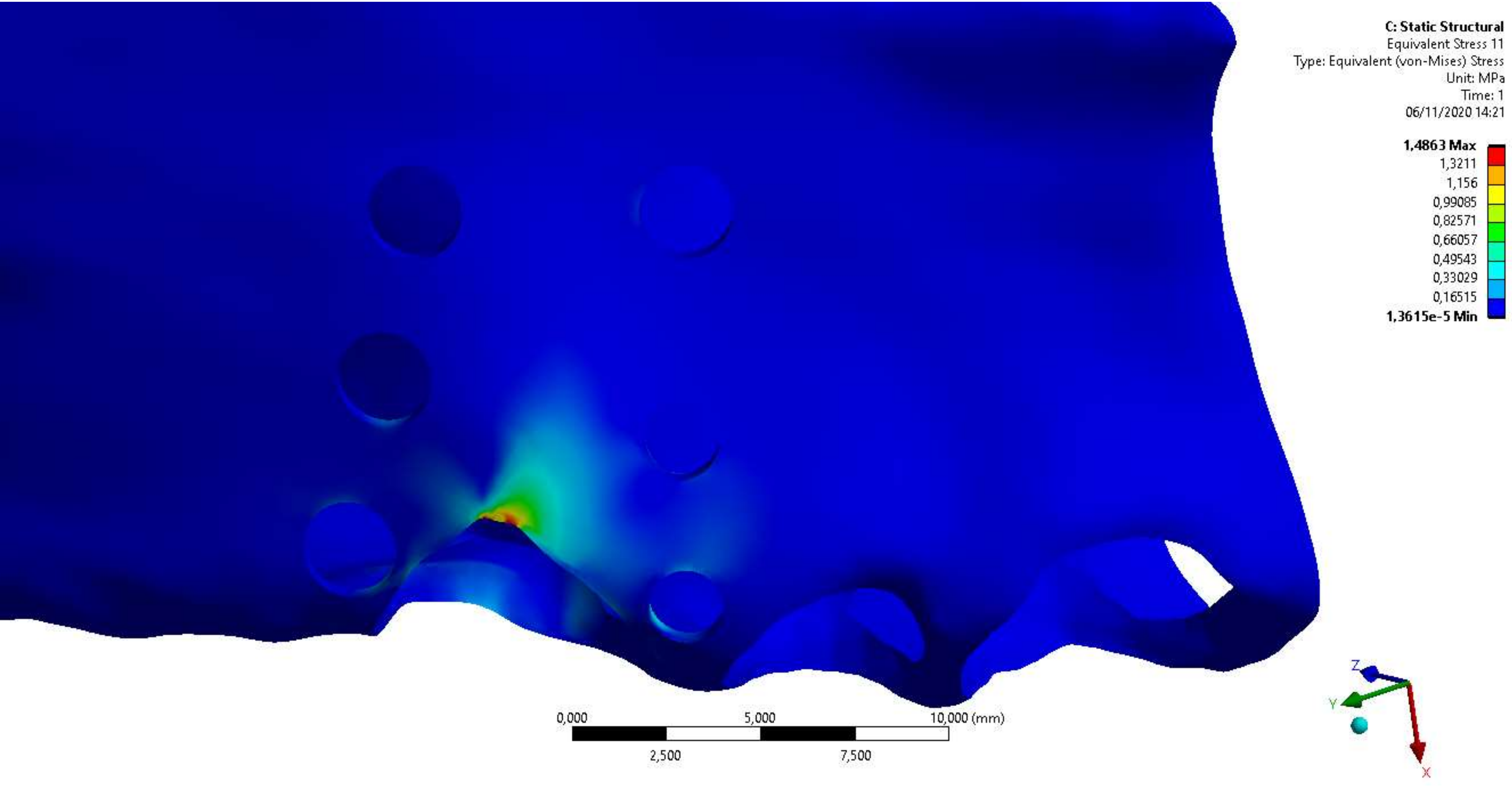
C: Static Structural
Equivalent Stress 11
Type: Equivalent (von-Mises) Stress
Unit: MPa
Time: 1
06/11/2020 14:21
1,4863 Max
1,3211
1,156
0,99085
0,82571
0,66057
0,49543
0,33029
0,16515
1,3615e-5 Min
0,000
5,000
10,000 (mm)
2,500
7,500
Z
Y
X

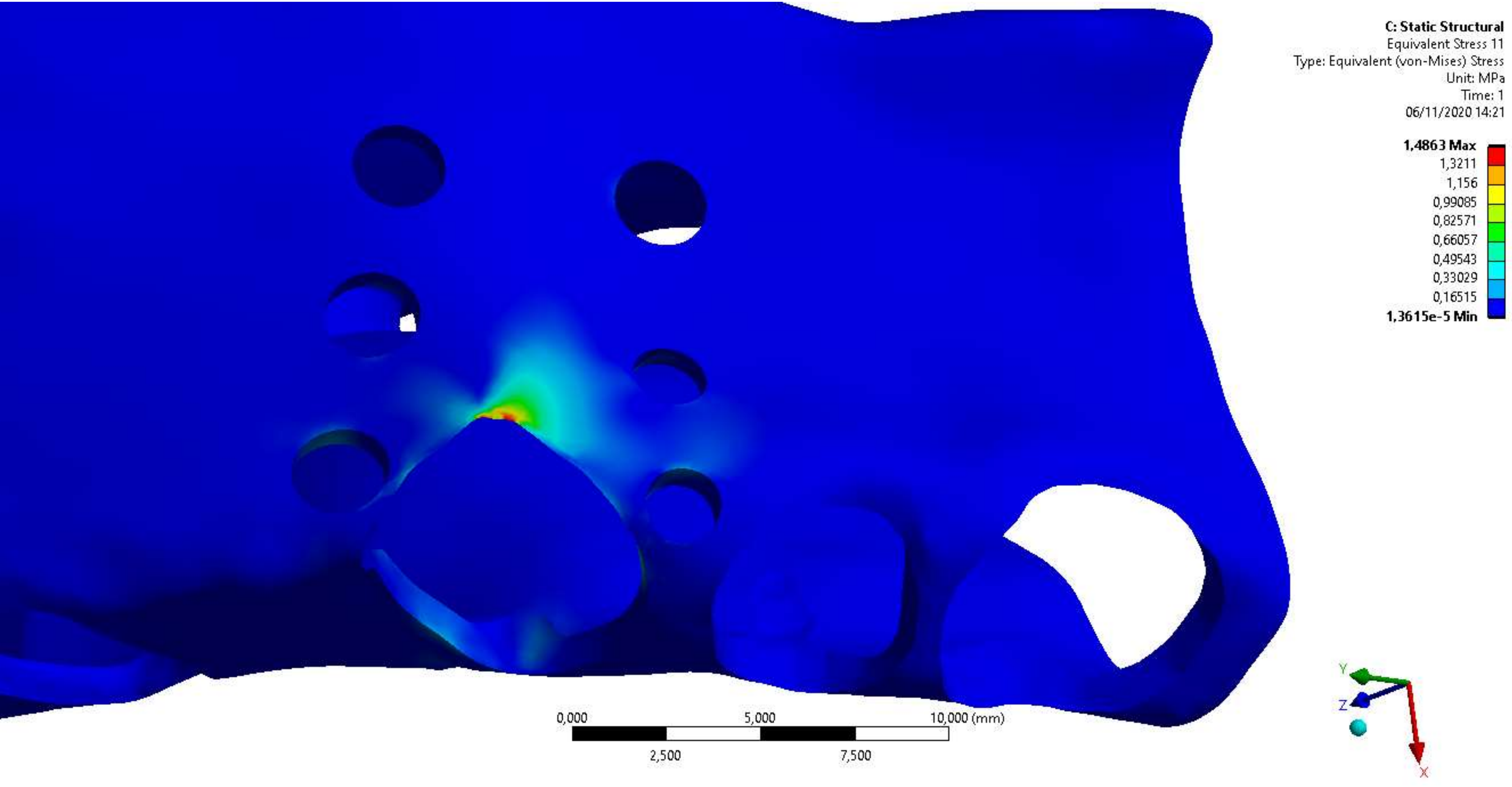
C: Static Structural
Equivalent Stress 11
Type: Equivalent (von-Mises) Stress
Unit: MPa
Time: 1
06/11/2020 14:21
1,4863 Max
1,3211
1,156
0,99085
0,82571
0,66057
0,49543
0,33029
0,16515
1,3615e-5 Min
0,000
5,000
10,000 (mm)
2,500
7,500
Y
Z
X

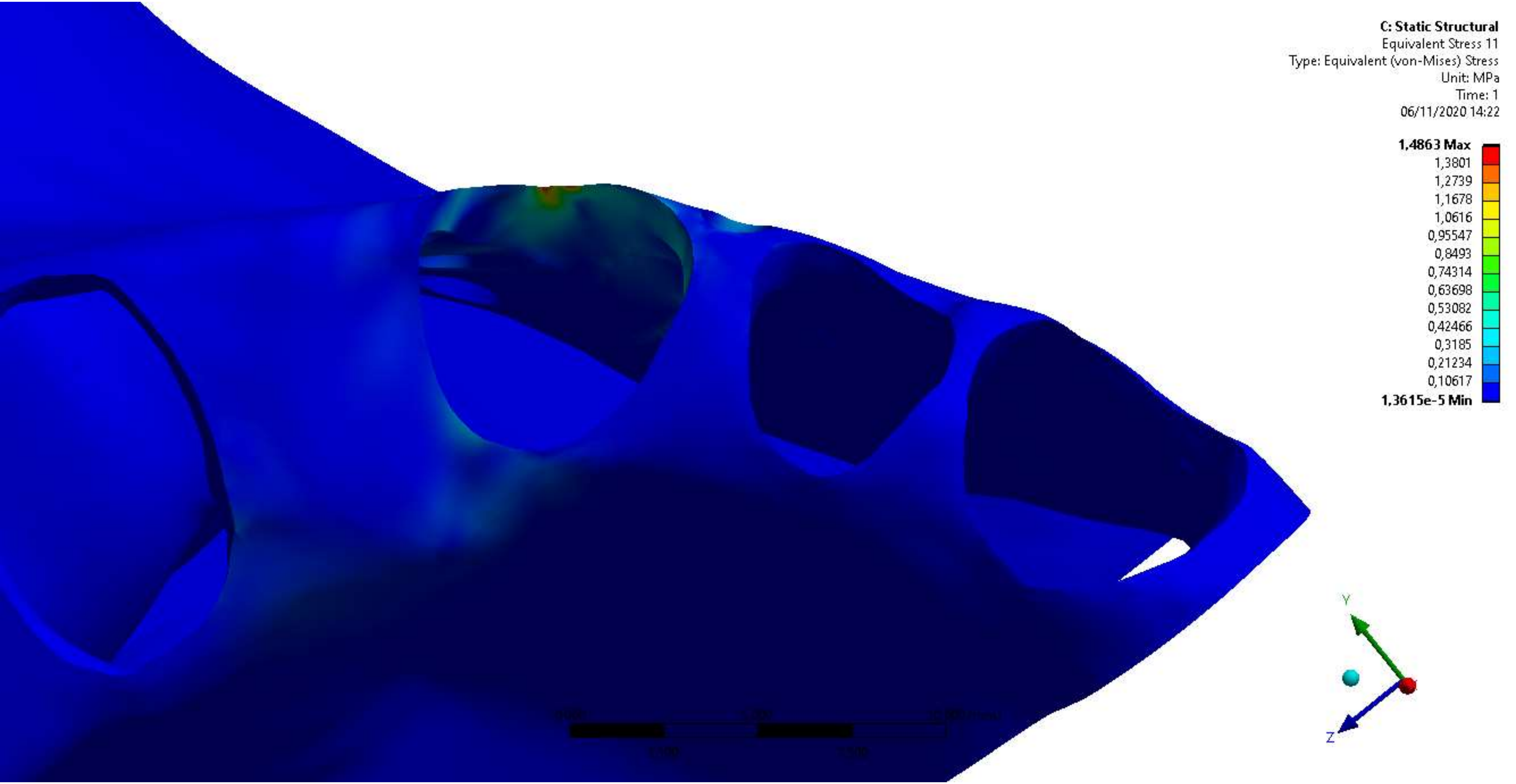
C: Static Structural
Equivalent Stress 11
Type: Equivalent (von-Mises) Stress
Unit: MPa
Time: 1
06/11/2020 14:22
1,4863 Max
1,3801
1,2739
1,1678
1,0616
0,95547
0,8493
0,74314
0,63698
0,53082
0,42466
0,3185
0,21234
0,10617
1,3615e-5 Min
Y
Z

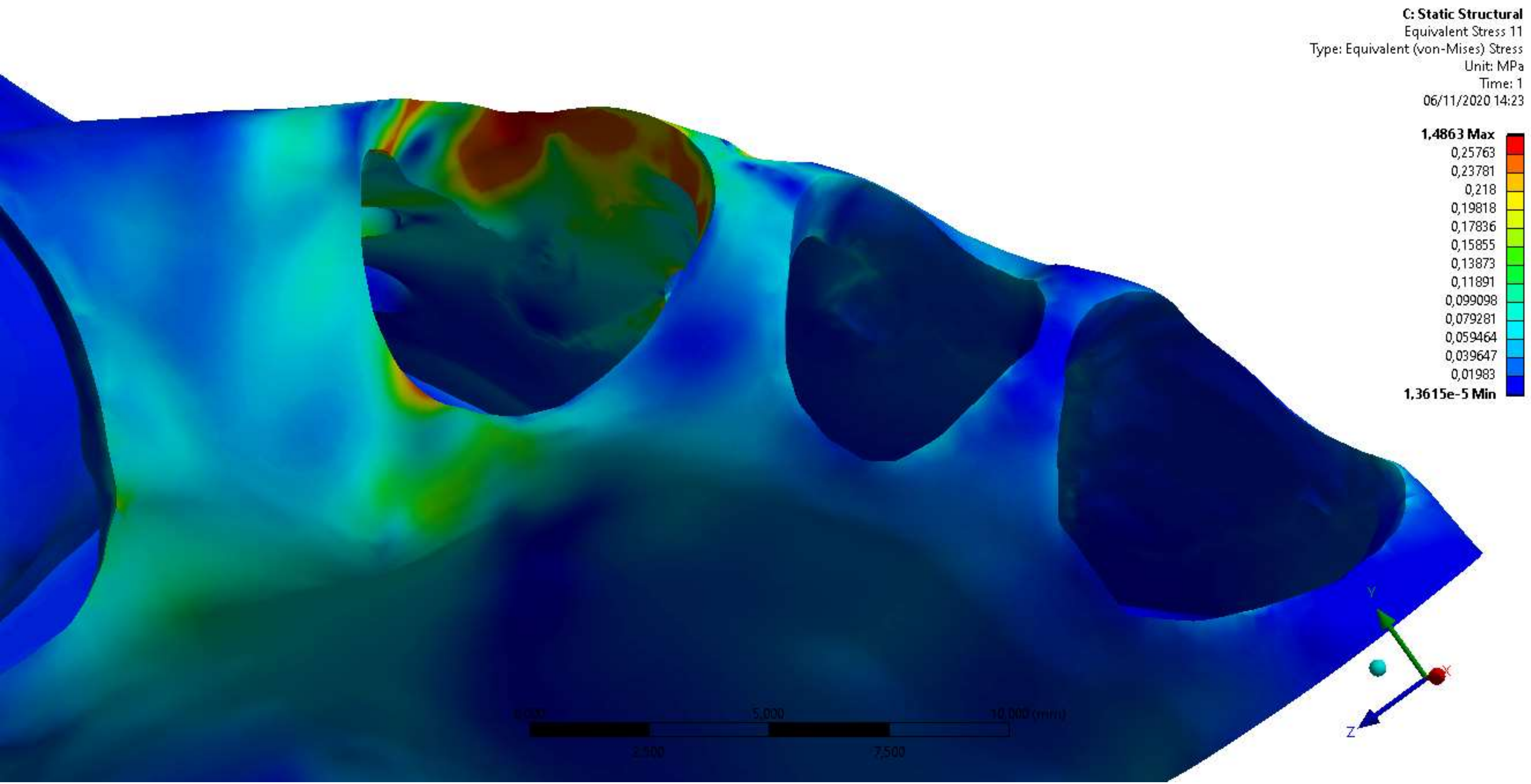
C: Static Structural
Equivalent Stress 11
Type: Equivalent (von-Mises) Stress
Unit: MPa
Time: 1
06/11/2020 14:23
1,4863 Max
0,25763
0,23781
0,218
0,19818
0,17836
0,15855
0,13873
0,11891
0,099098
0,079281
0,059464
0,039647
0,01983
1,3615e-5 Min
Y
X
Z

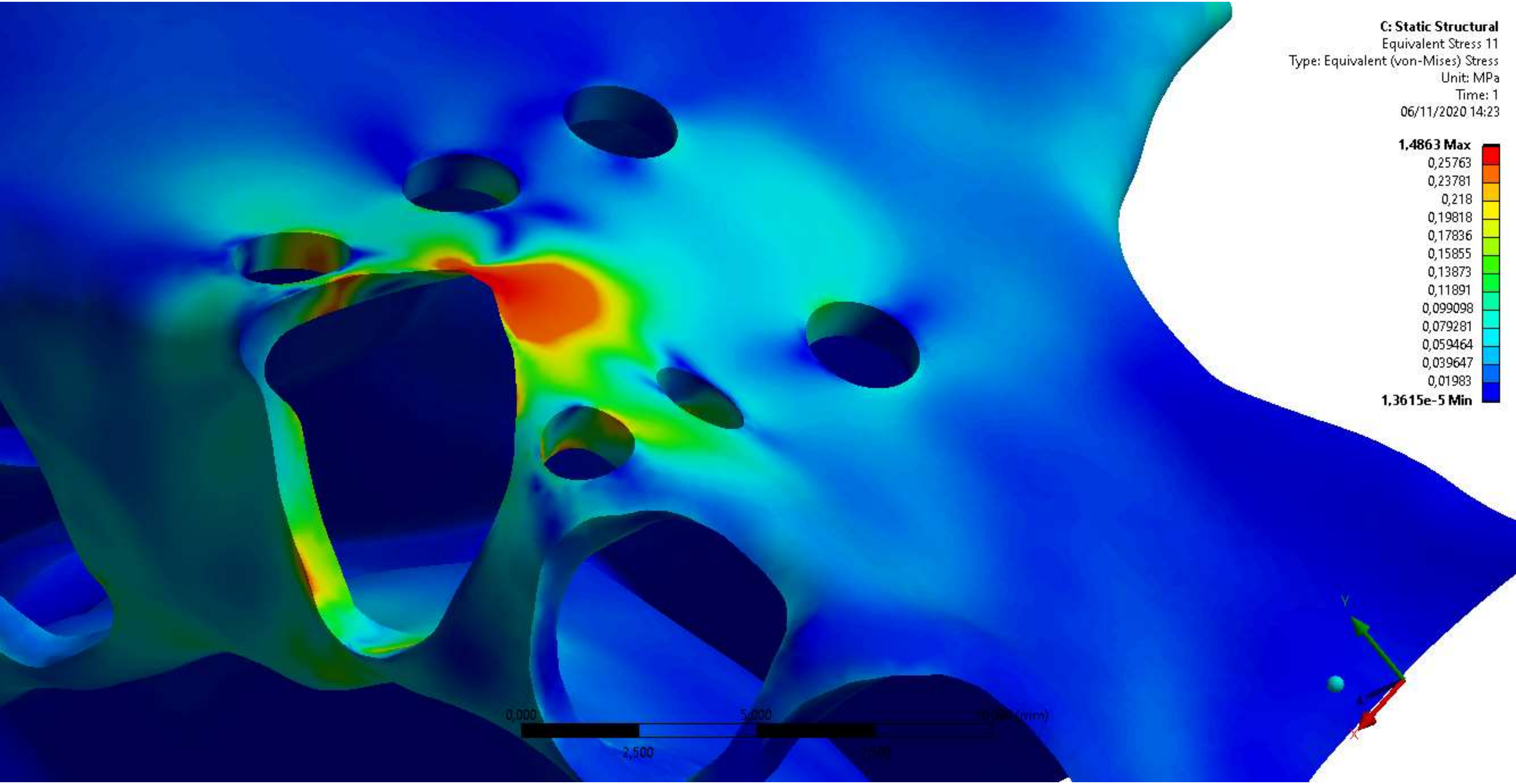
C: Static Structural
Equivalent Stress 11
Type: Equivalent (von-Mises) Stress
Unit: MPa
Time: 1
06/11/2020 14:23
1,4863 Max
0,25763
0,23781
0,218
0,19818
0,17836
0,15855
0,13873
0,11891
0,099098
0,079281
0,059464
0,039647
0,01983
1,3615e-5 Min
0,000
5,000
2,500
Y
X

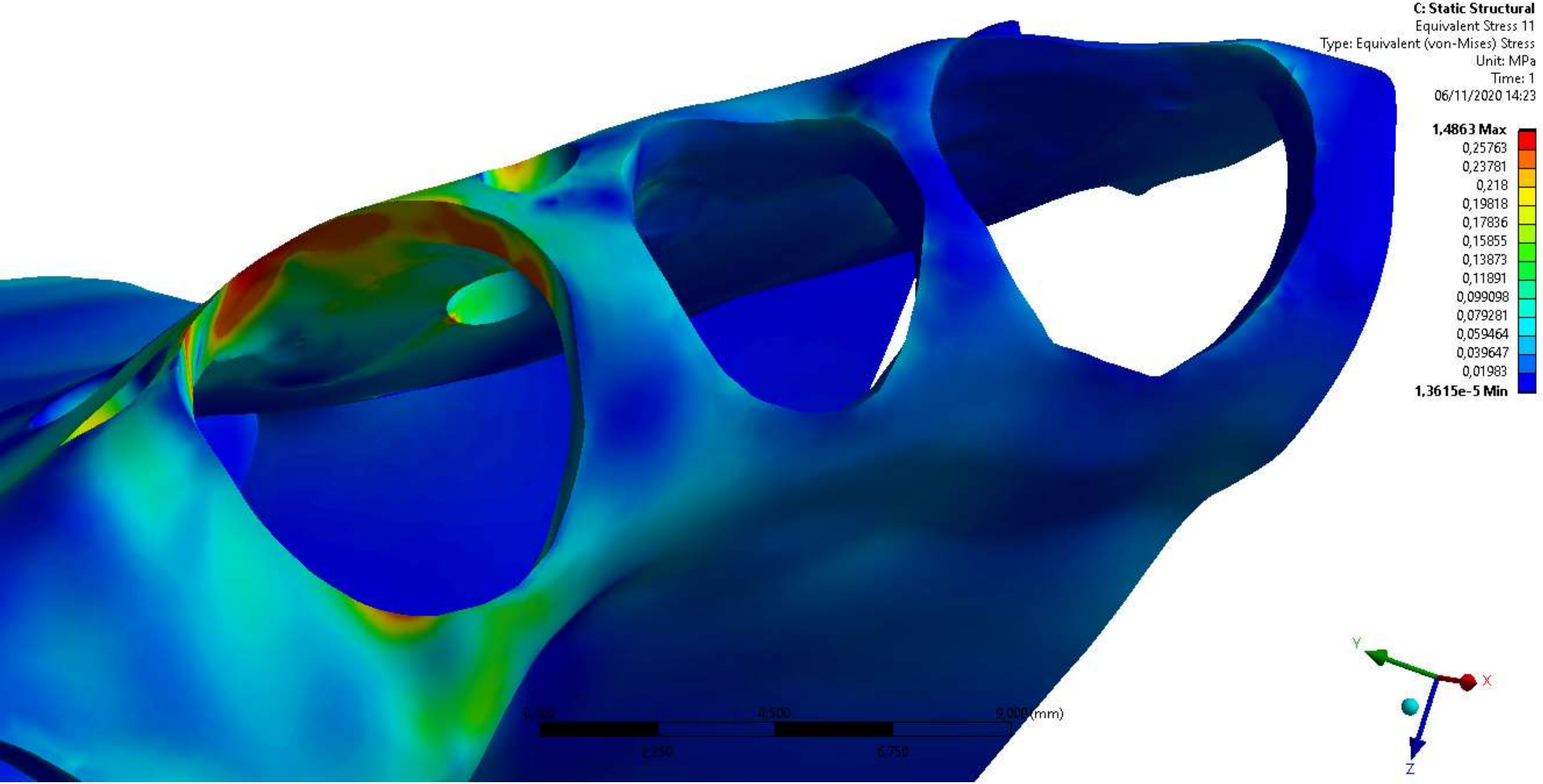
C: Static Structural
Equivalent Stress 11
Type: Equivalent (von-Mises) Stress
Unit: MPa
Time: 1
06/11/2020 14:23
1,4863 Max
0,25763
0,23781
0,218
0,19818
0,17836
0,15855
0,13873
0,11891
0,099098
0,079281
0,059464
0,039647
0,01983
1,3615e-5 Min
0,000
4,500
9,000 (mm)
2,250
6,750
Y
X
Z

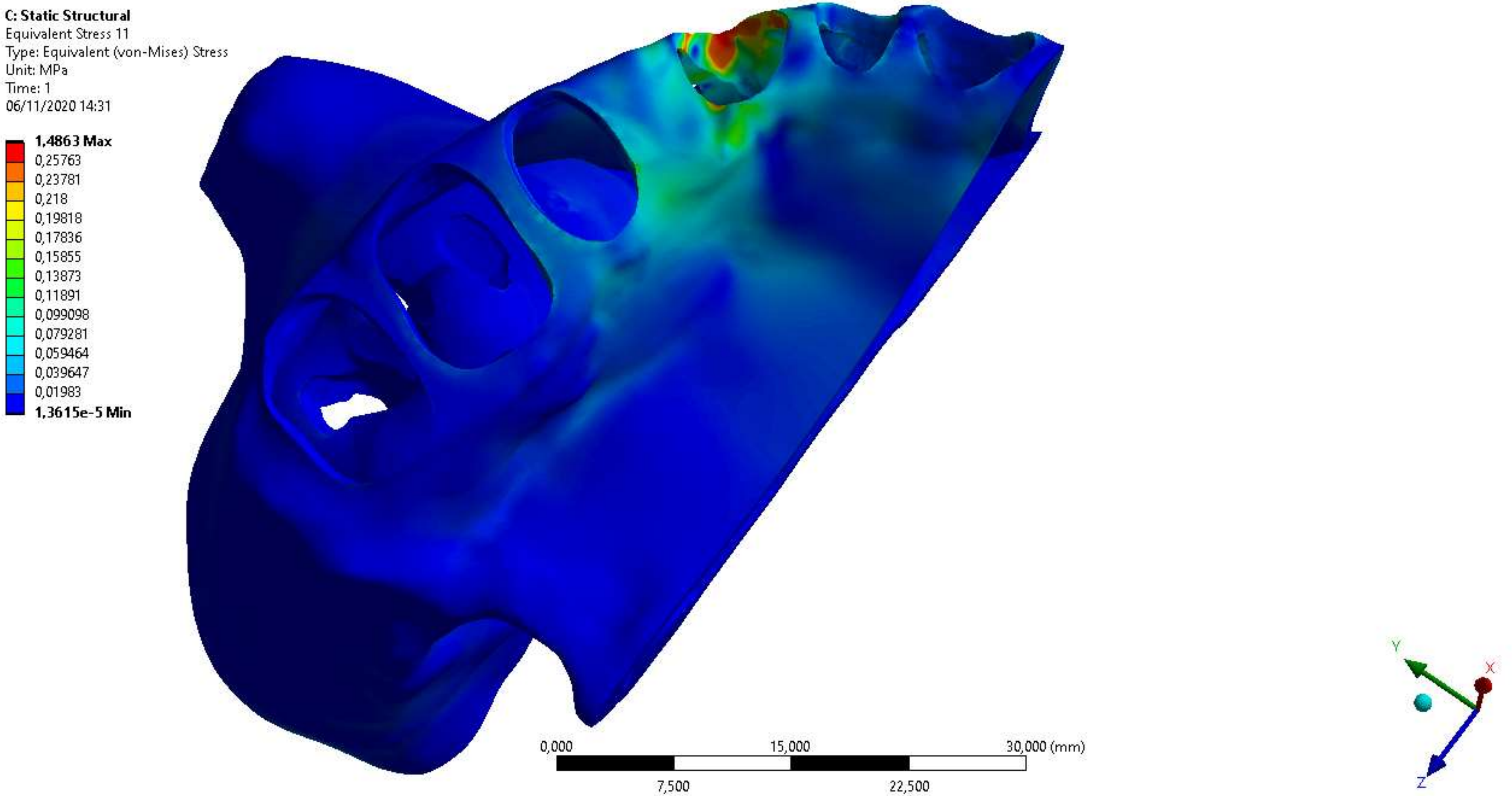
C: Static Structural
Equivalent Stress 11
Type: Equivalent (von-Mises) Stress
Unit: MPa
Time: 1
06/11/2020 14:31
1,4863 Max
0,25763
0,23781
0,218
0,19818
0,17836
0,15855
0,13873
0,11891
0,099098
0,079281
0,059464
0,039647
0,01983
1,3615e-5 Min
0,000
15,000
30,000 (mm)
7,500
22,500
Y
X
Z

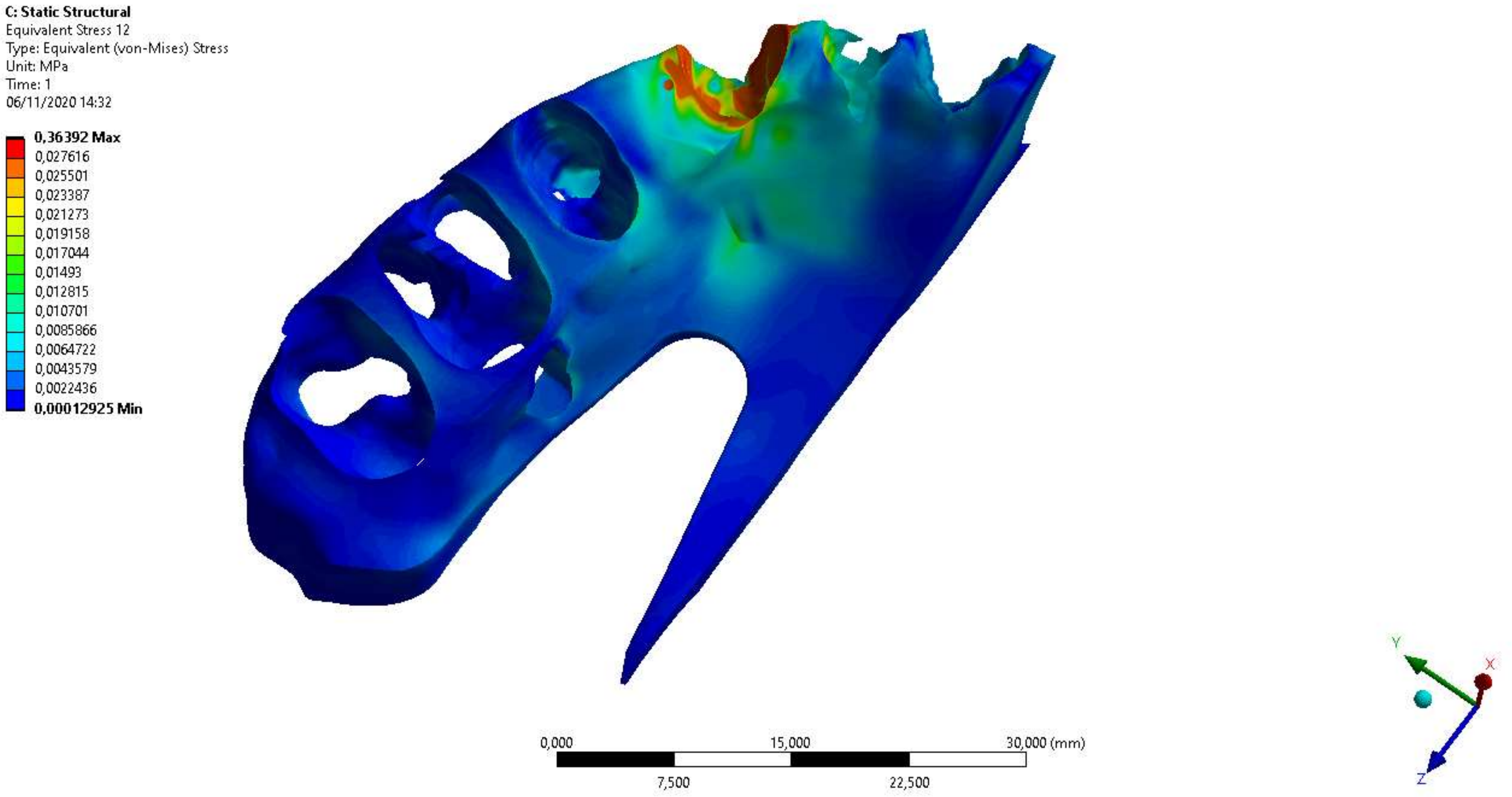
C: Static Structural
Equivalent Stress 12
Type: Equivalent (von-Mises) Stress
Unit: MPa
Time: 1
06/11/2020 14:32
0,36392 Max
0,027616
0,025501
0,023387
0,021273
0,019158
0,017044
0,01493
0,012815
0,010701
0,0085866
0,0064722
0,0043579
0,0022436
0,00012925 Min
0,000
15,000
30,000 (mm)
7,500
22,500
Y
X
Z

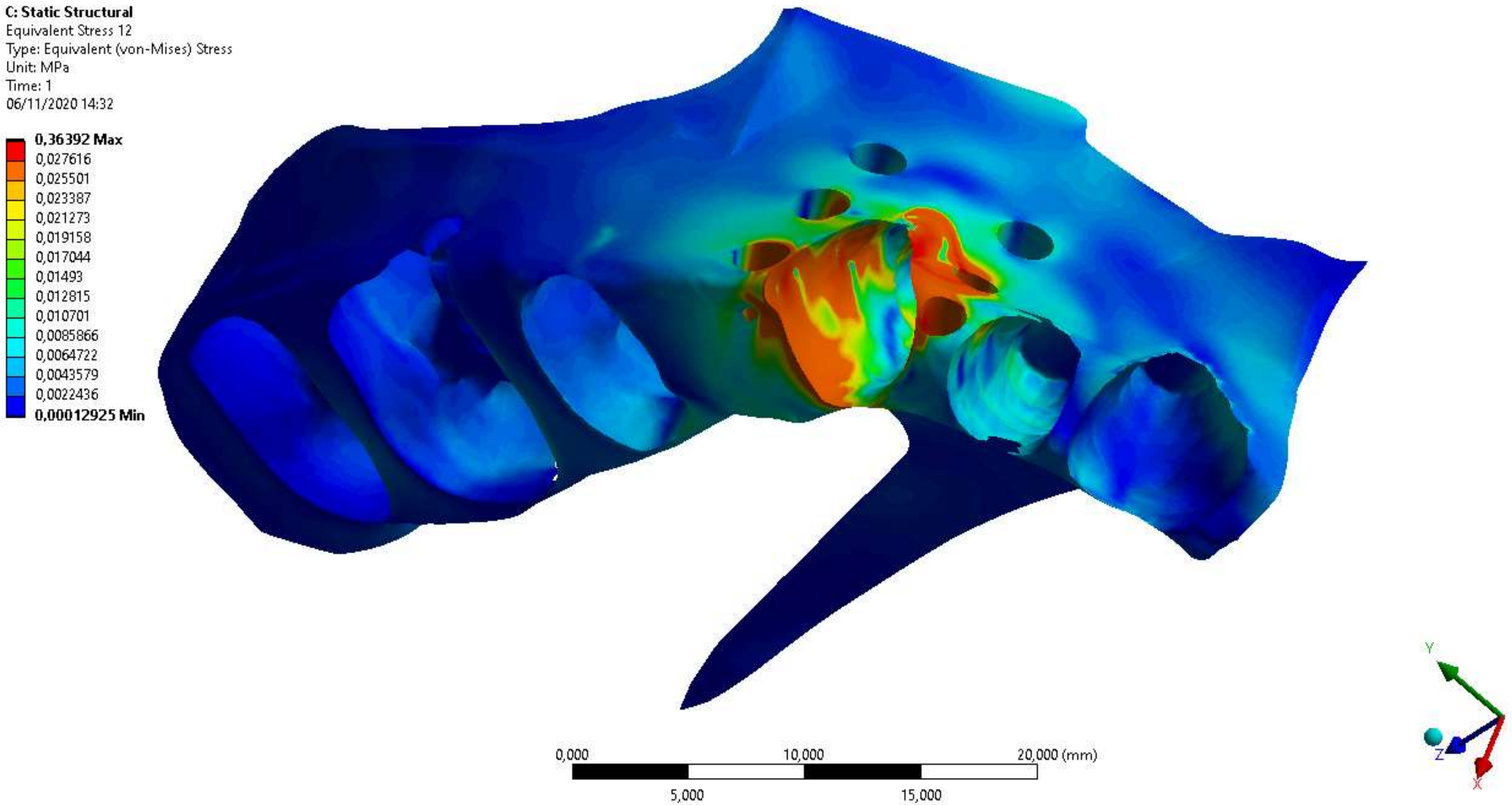
C: Static Structural
Equivalent Stress 12
Type: Equivalent (von-Mises) Stress
Unit: MPa
Time: 1
06/11/2020 14:32
0,36392 Max
0,027616
0,025501
0,023387
0,021273
0,019158
0,017044
0,01493
0,012815
0,010701
0,0085866
0,0064722
0,0043579
0,0022436
0,00012925 Min
0,000
10,000
20,000 (mm)
5,000
15,000
Y
Z
X

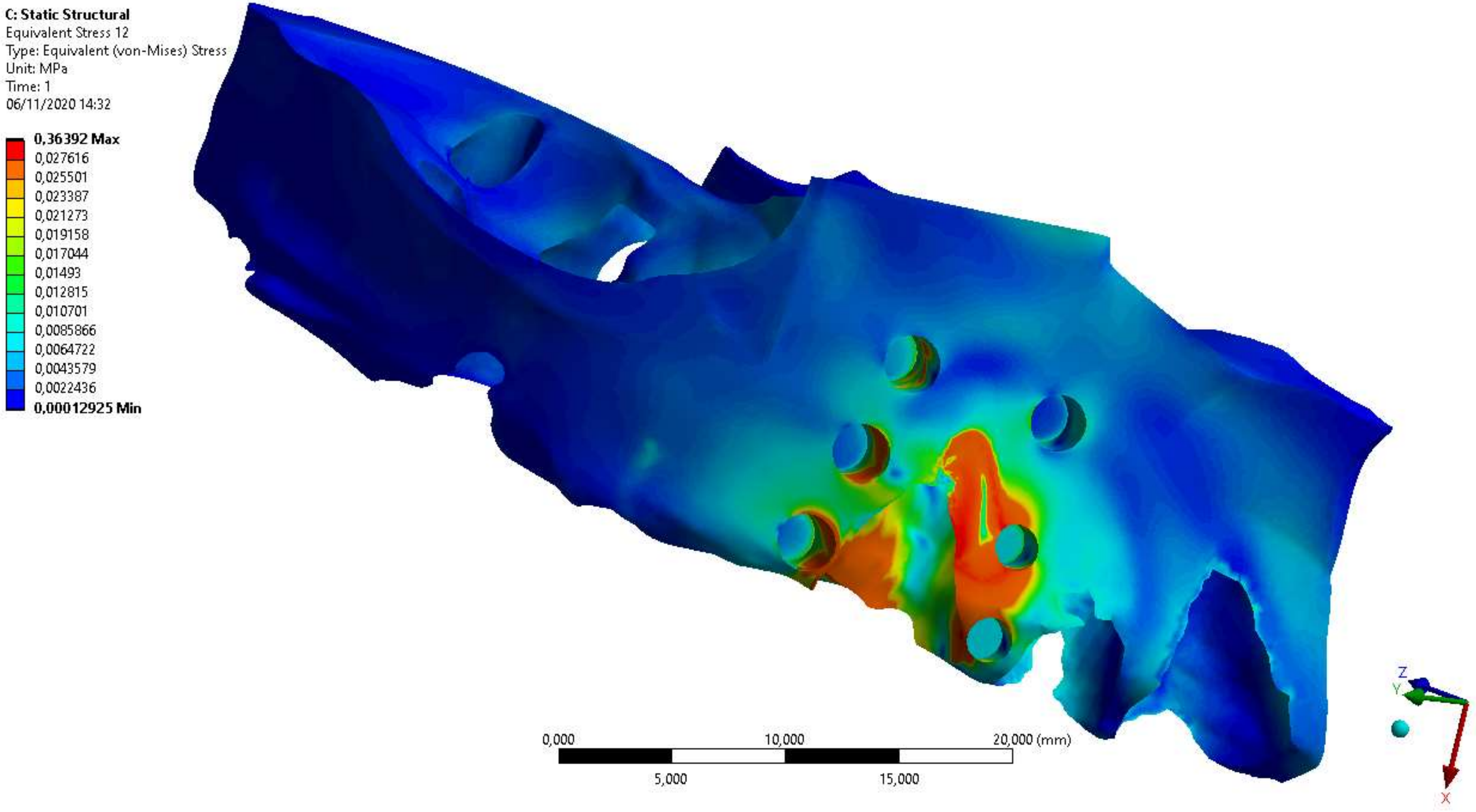
C: Static Structural
Equivalent Stress 12
Type: Equivalent (von-Mises) Stress
Unit: MPa
Time: 1
06/11/2020 14:32
0,36392 Max
0,027616
0,025501
0,023387
0,021273
0,019158
0,017044
0,01493
0,012815
0,010701
0,0085866
0,0064722
0,0043579
0,0022436
0,00012925 Min
0,000
10,000
20,000 (mm)
5,000
15,000
Z
Y
X

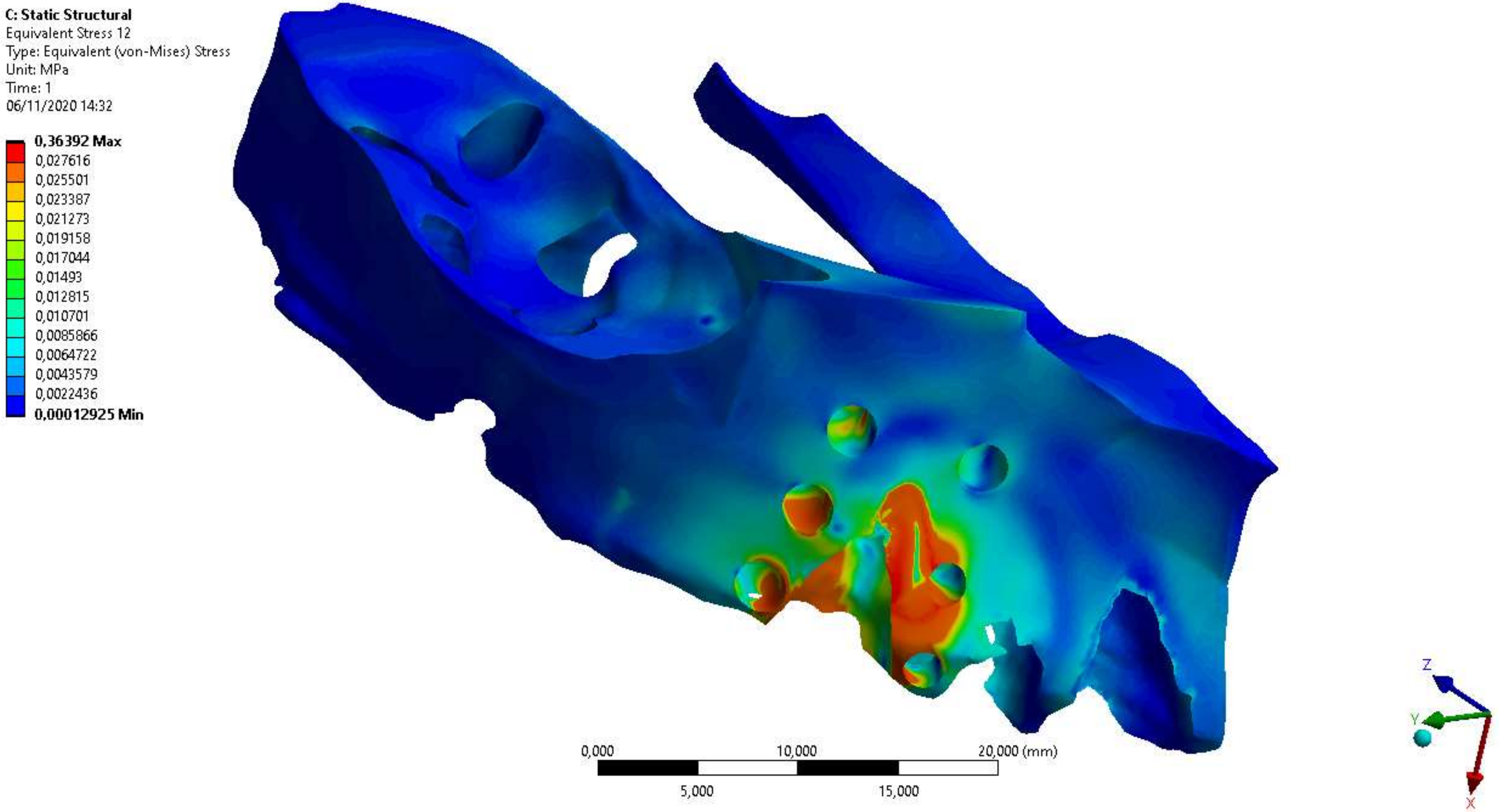
C: Static Structural
Equivalent Stress 12
Type: Equivalent (von-Mises) Stress
Unit: MPa
Time: 1
06/11/2020 14:32
0,36392 Max
0,027616
0,025501
0,023387
0,021273
0,019158
0,017044
0,01493
0,012815
0,010701
0,0085866
0,0064722
0,0043579
0,0022436
0,00012925 Min
0,000
10,000
20,000 (mm)
5,000
15,000
Z
Y
X

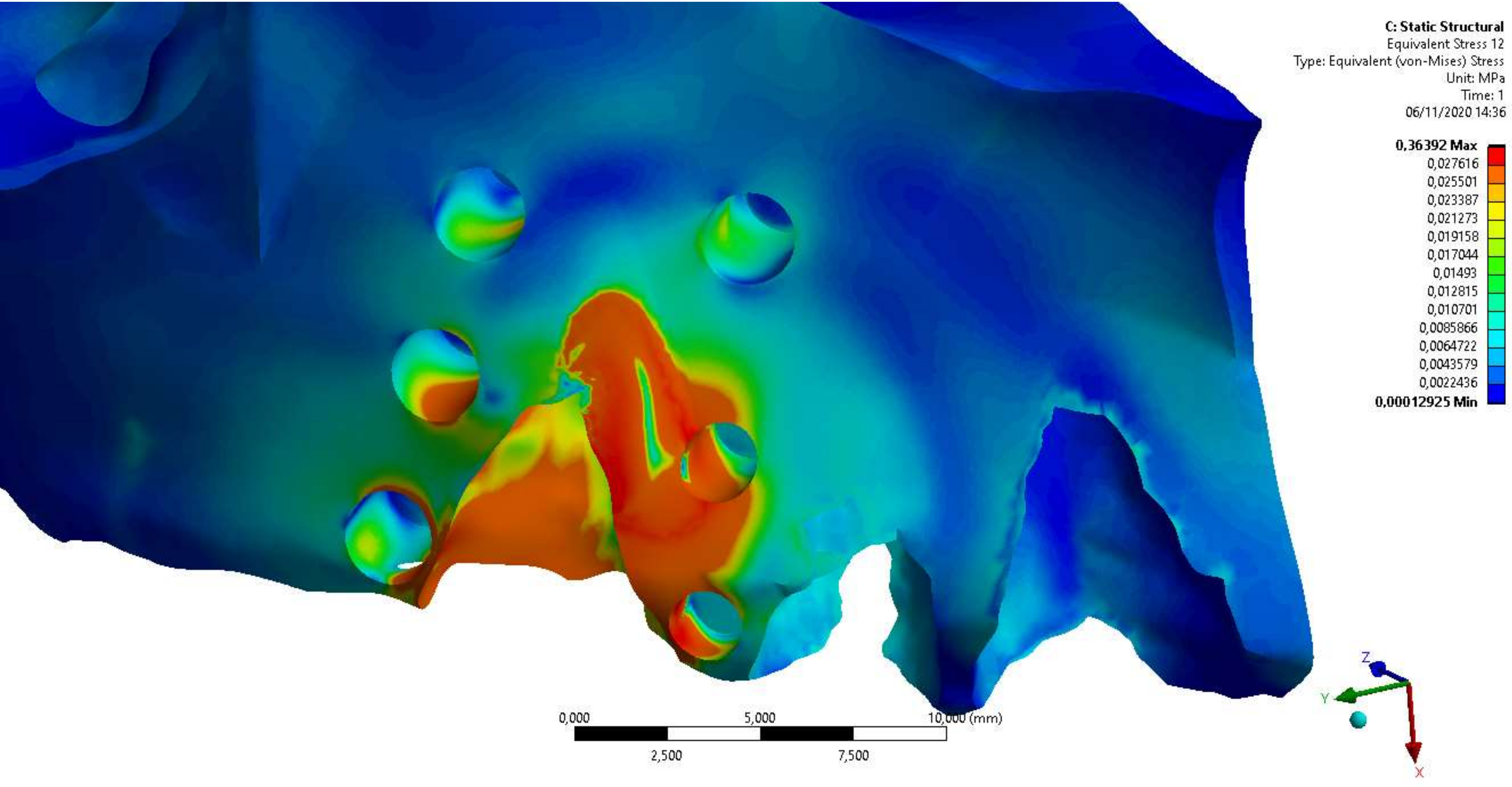
C: Static Structural
Equivalent Stress 12
Type: Equivalent (von-Mises) Stress
Unit: MPa
Time: 1
06/11/2020 14:36
0,36392 Max
0,027616
0,025501
0,023387
0,021273
0,019158
0,017044
0,01493
0,012815
0,010701
0,0085866
0,0064722
0,0043579
0,0022436
0,00012925 Min
0,000
5,000
10,000 (mm)
2,500
7,500
Z
Y
X

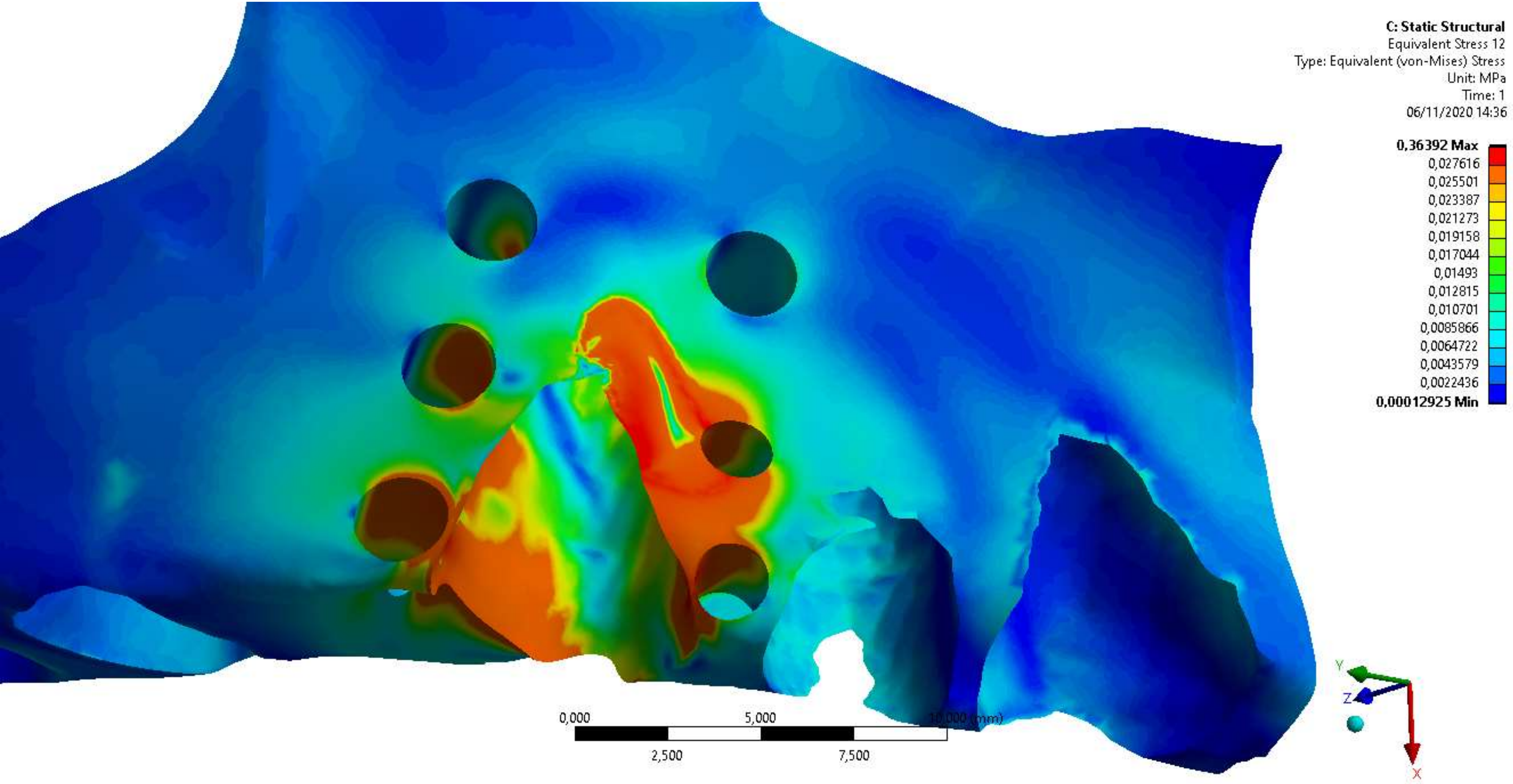
C: Static Structural
Equivalent Stress 12
Type: Equivalent (von-Mises) Stress
Unit: MPa
Time: 1
06/11/2020 14:36
0,36392 Max
0,027616
0,025501
0,023387
0,021273
0,019158
0,017044
0,01493
0,012815
0,010701
0,0085866
0,0064722
0,0043579
0,0022436
0,00012925 Min
0,000
5,000
10,000 (mm)
2,500
7,500
Y
Z
X

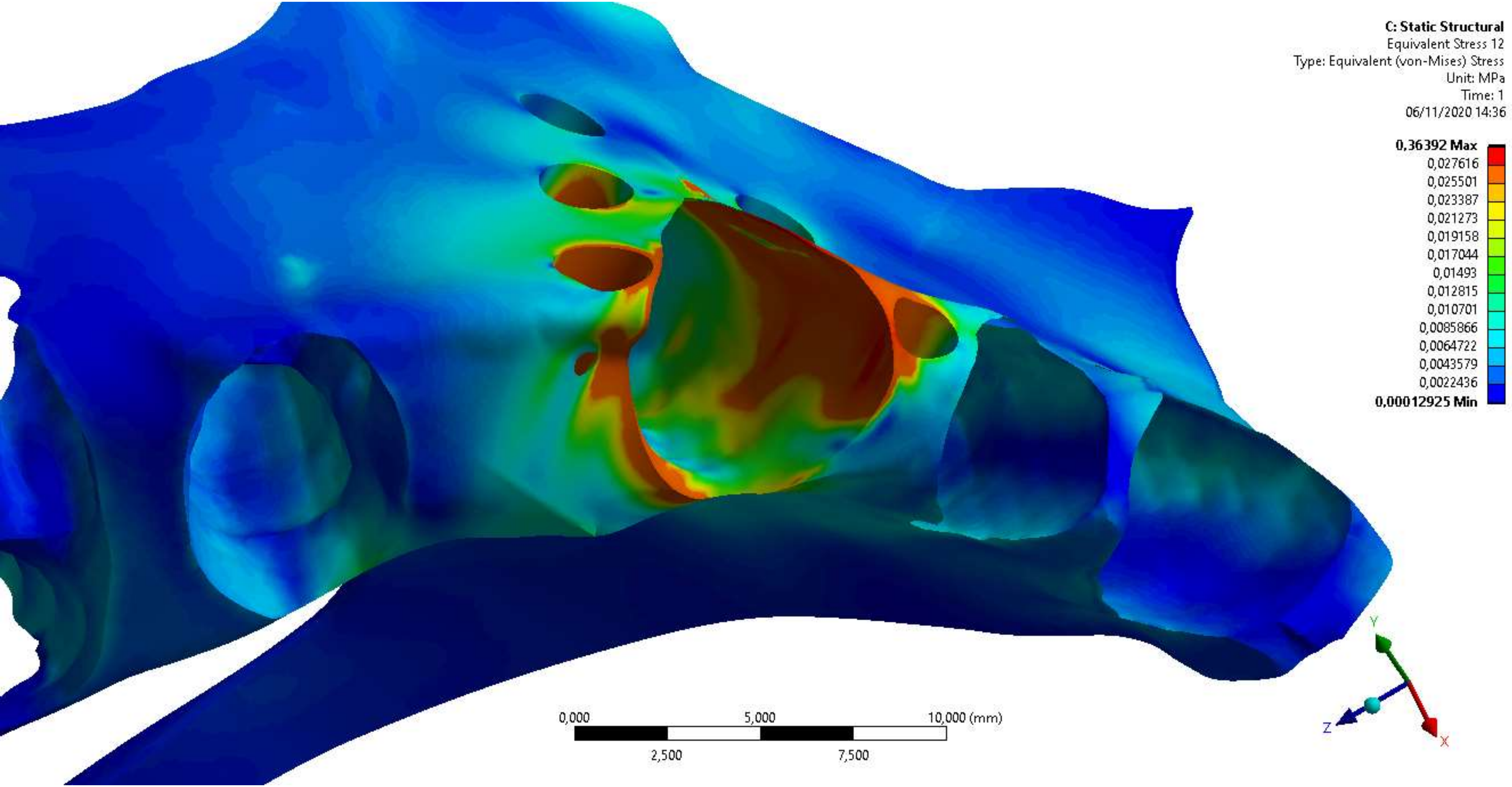

C: Static Structural
Equivalent Stress 12
Type: Equivalent (von-Mises) Stress
Unit: MPa
Time: 1
06/11/2020 14:36
0,36392 Max
0,027616
0,025501
0,023387
0,021273
0,019158
0,017044
0,01493
0,012815
0,010701
0,0085866
0,0064722
0,0043579
0,0022436
0,00012925 Min
0,000
5,000
10,000 (mm)
2,500
7,500
Y
Z
X

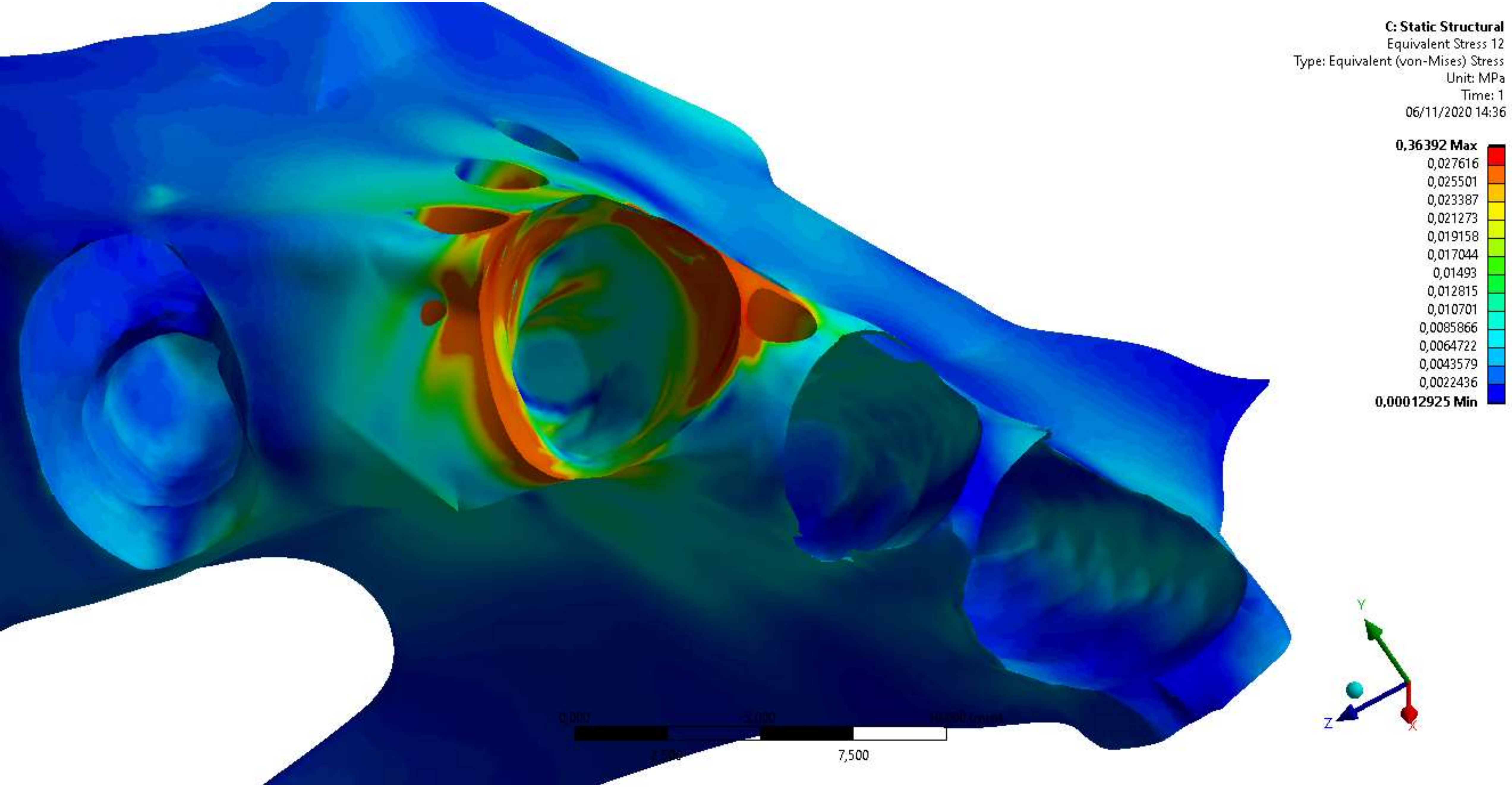
C: Static Structural
Equivalent Stress 12
Type: Equivalent (von-Mises) Stress
Unit: MPa
Time: 1
06/11/2020 14:36
0,36392 Max
0,027616
0,025501
0,023387
0,021273
0,019158
0,017044
0,01493
0,012815
0,010701
0,0085866
0,0064722
0,0043579
0,0022436
0,00012925 Min
7,500
Y
Z
X

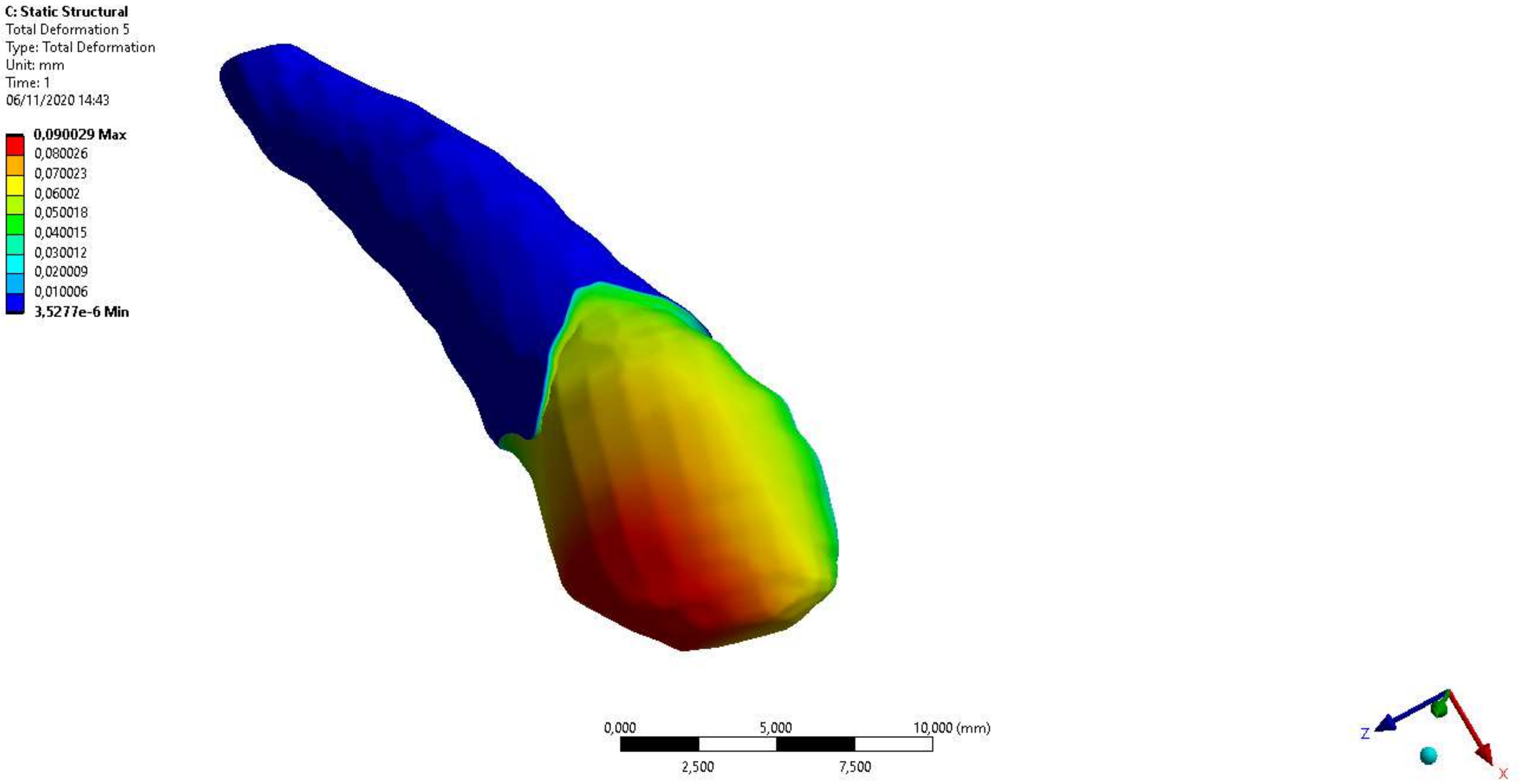
C: Static Structural
Total Deformation 5
Type: Total Deformation
Unit: mm
Time: 1
06/11/2020 14:43
0,090029 Max
0,080026
0,070023
0,06002
0,050018
0,040015
0,030012
0,020009
0,010006
3,5277e-6 Min
0,000
5,000
10,000 (mm)
2,500
7,500
Z
X

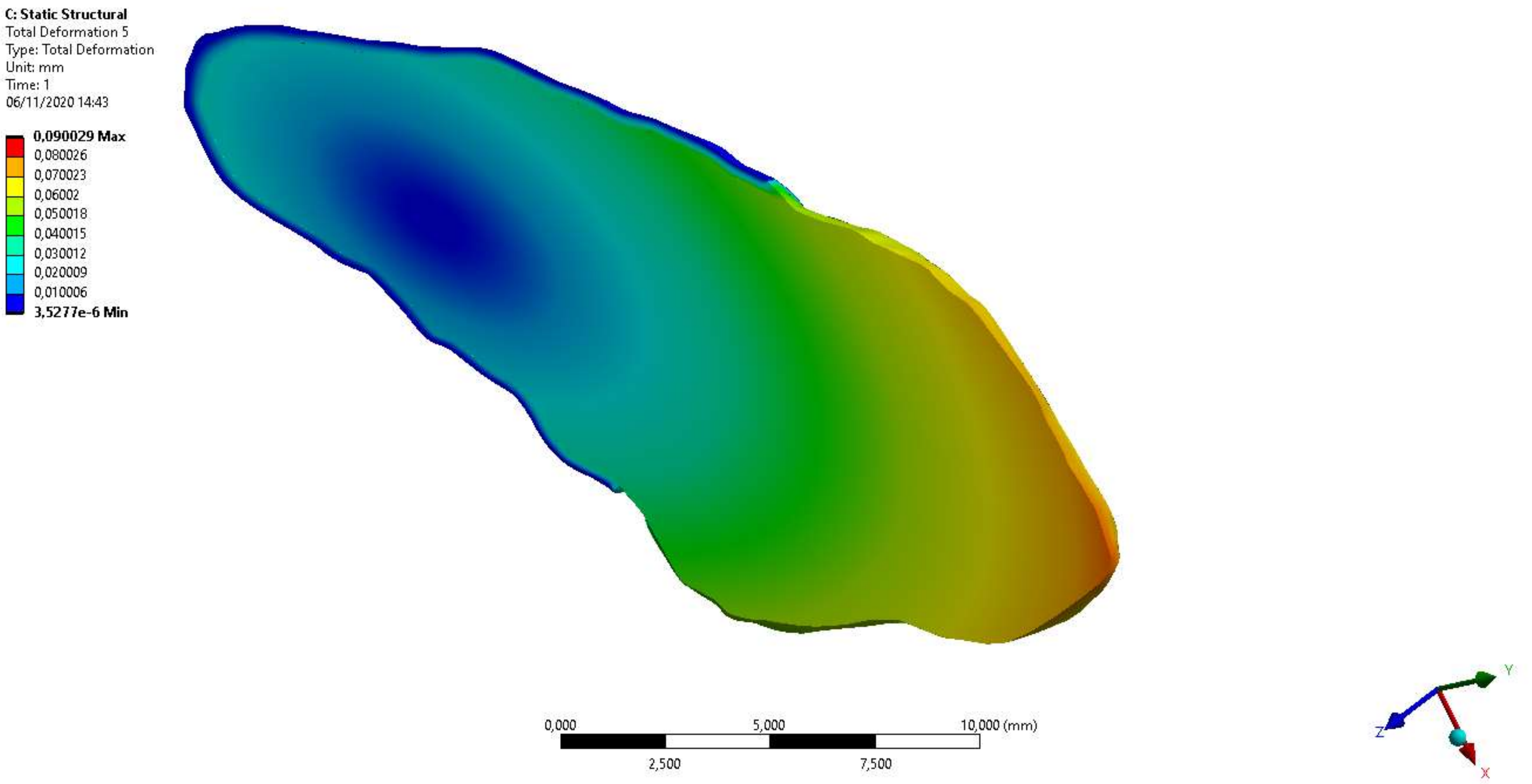

C: Static Structural
Total Deformation 5
Type: Total Deformation
Unit: mm
Time: 1
06/11/2020 14:43
0,090029 Max
0,080026
0,070023
0,06002
0,050018
0,040015
0,030012
0,020009
0,010006
3,5277e-6 Min
0,000
5,000
10,000 (mm)
2,500
7,500
Y
Z
X

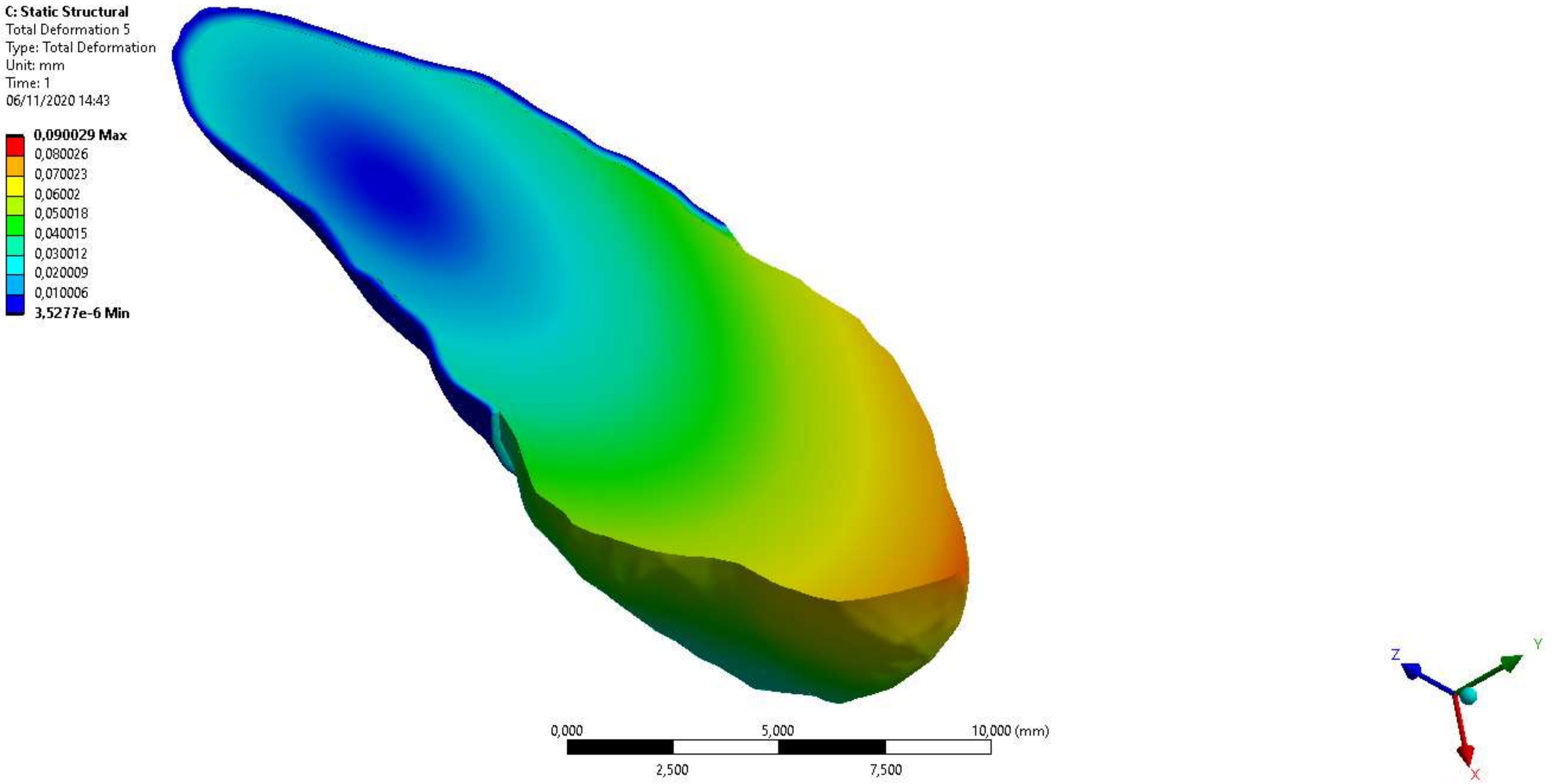
C: Static Structural
Total Deformation 5
Type: Total Deformation
Unit: mm
Time: 1
06/11/2020 14:43
0,090029 Max
0,080026
0,070023
0,06002
0,050018
0,040015
0,030012
0,020009
0,010006
3,5277e-6 Min
0,000
5,000
10,000 (mm)
2,500
7,500
Z
Y
X

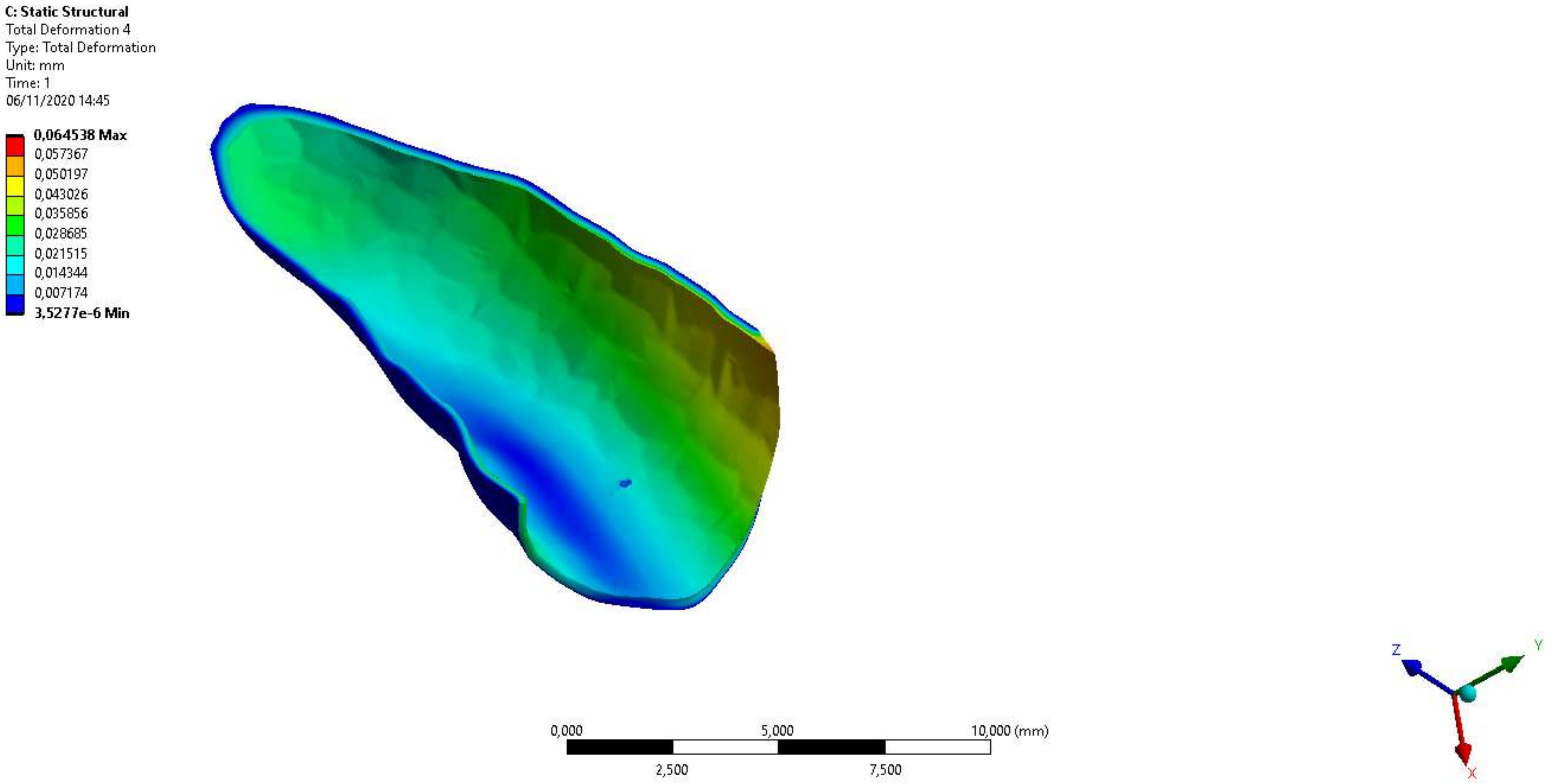
C: Static Structural
Total Deformation 4
Type: Total Deformation
Unit: mm
Time: 1
06/11/2020 14:45
0,064538 Max
0,057367
0,050197
0,043026
0,035856
0,028685
0,021515
0,014344
0,007174
3,5277e-6 Min
0,000
5,000
10,000 (mm)
2,500
7,500
Z
Y
X

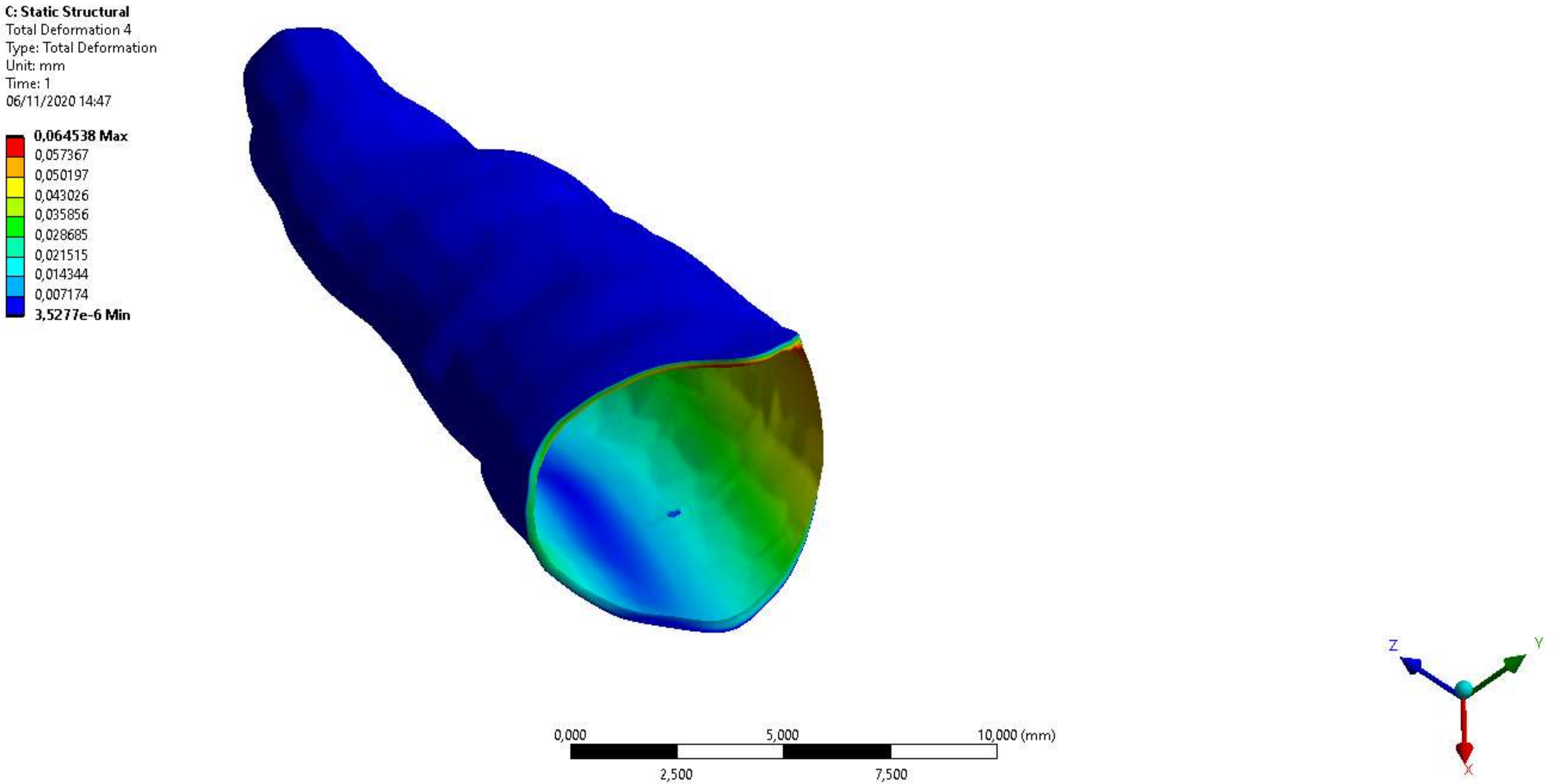
C: Static Structural
Total Deformation 4
Type: Total Deformation
Unit: mm
Time: 1
06/11/2020 14:47
0,064538 Max
0,057367
0,050197
0,043026
0,035856
0,028685
0,021515
0,014344
0,007174
3,5277e-6 Min
0,000
5,000
10,000 (mm)
2,500
7,500
Z
Y
X

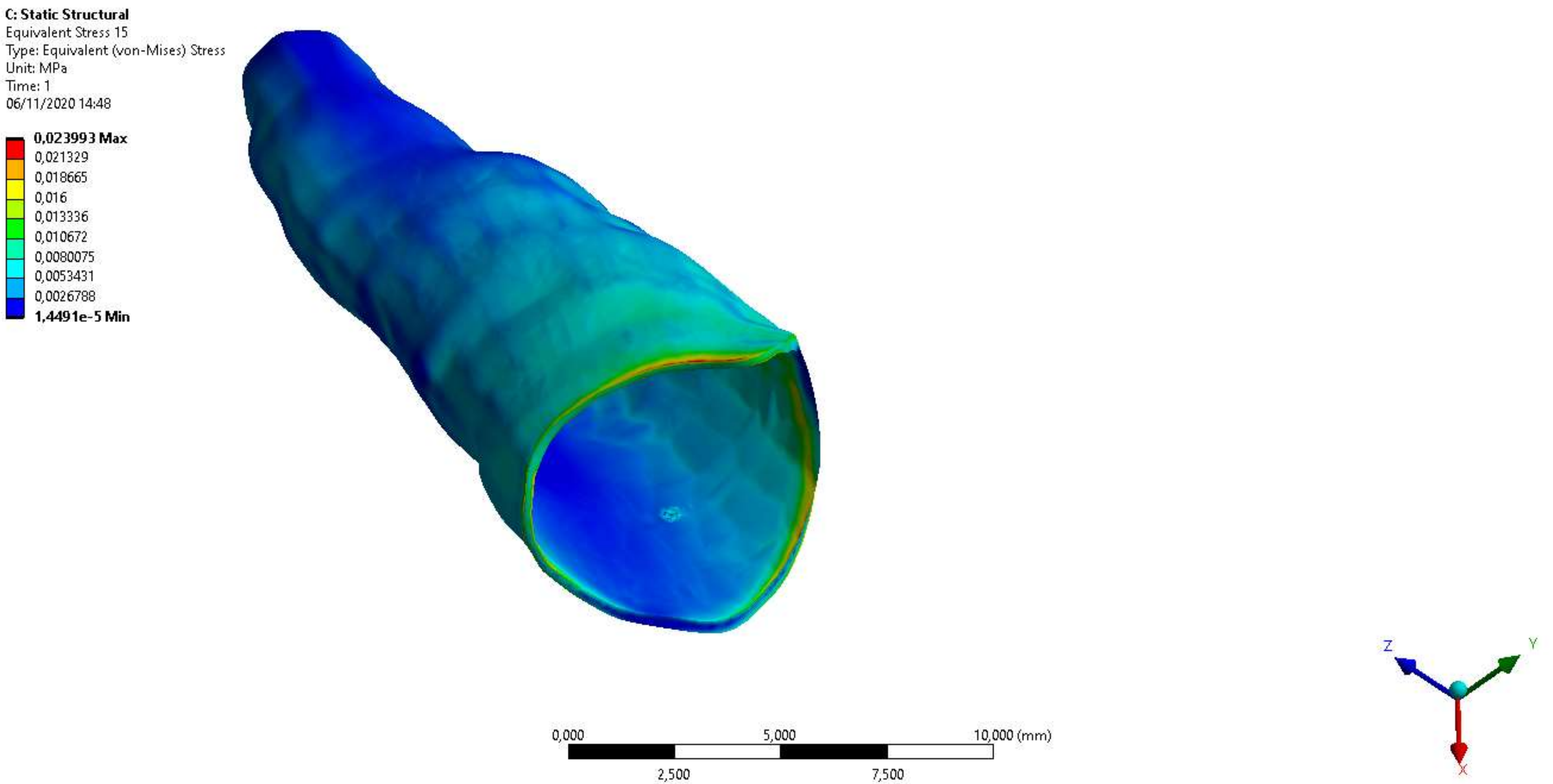
C: Static Structural
Equivalent Stress 15
Type: Equivalent (von-Mises) Stress
Unit: MPa
Time: 1
06/11/2020 14:48
0,023993 Max
0,021329
0,018665
0,016
0,013336
0,010672
0,0080075
0,0053431
0,0026788
1,4491e-5 Min
0,000
5,000
10,000 (mm)
2,500
7,500
Z
Y
X

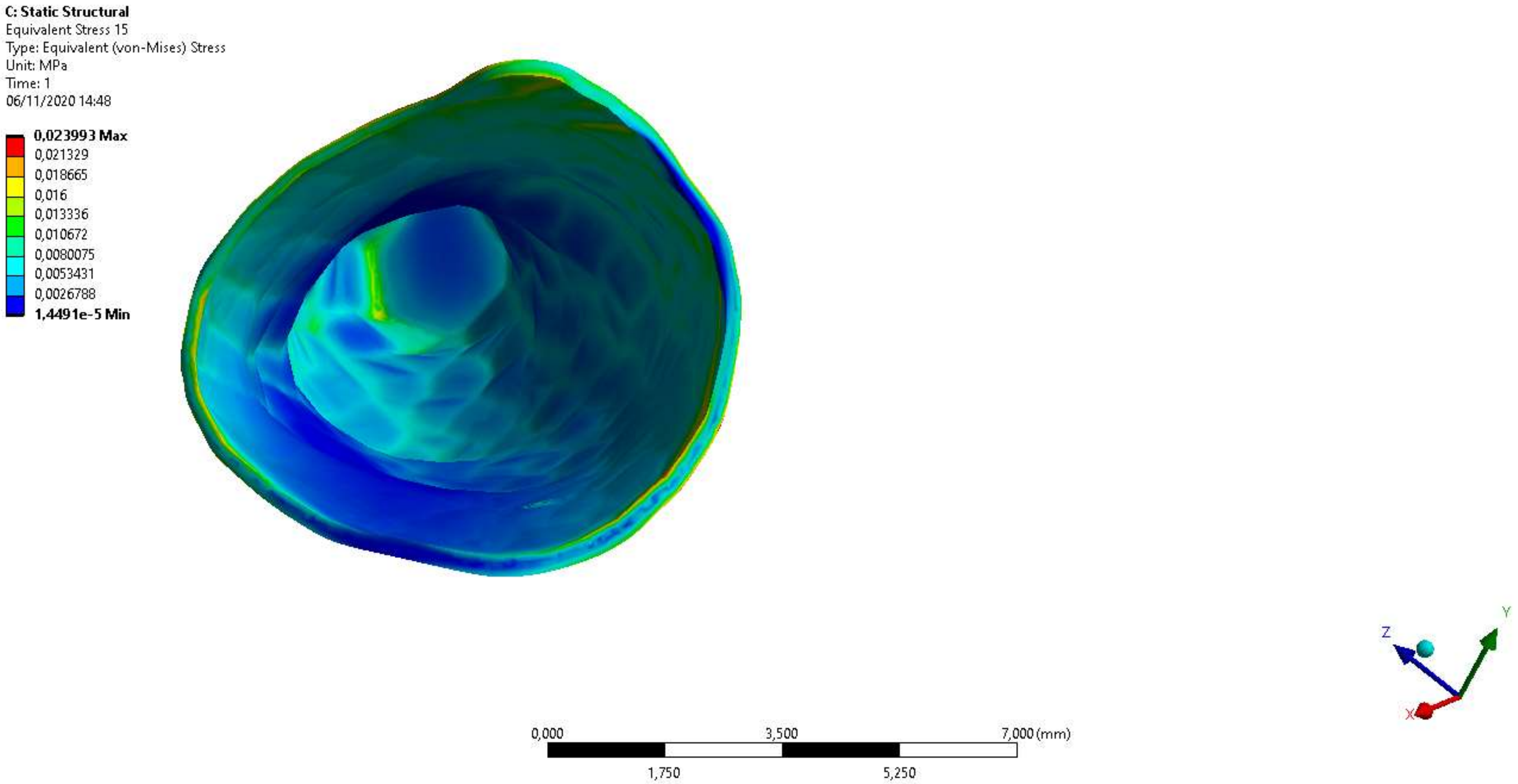
C: Static Structural
Equivalent Stress 15
Type: Equivalent (von-Mises) Stress
Unit: MPa
Time: 1
06/11/2020 14:48
0,023993 Max
0,021329
0,018665
0,016
0,013336
0,010672
0,0080075
0,0053431
0,0026788
1,4491e-5 Min
0,000
3,500
7,000 (mm)
1,750
5,250
Z
Y
X

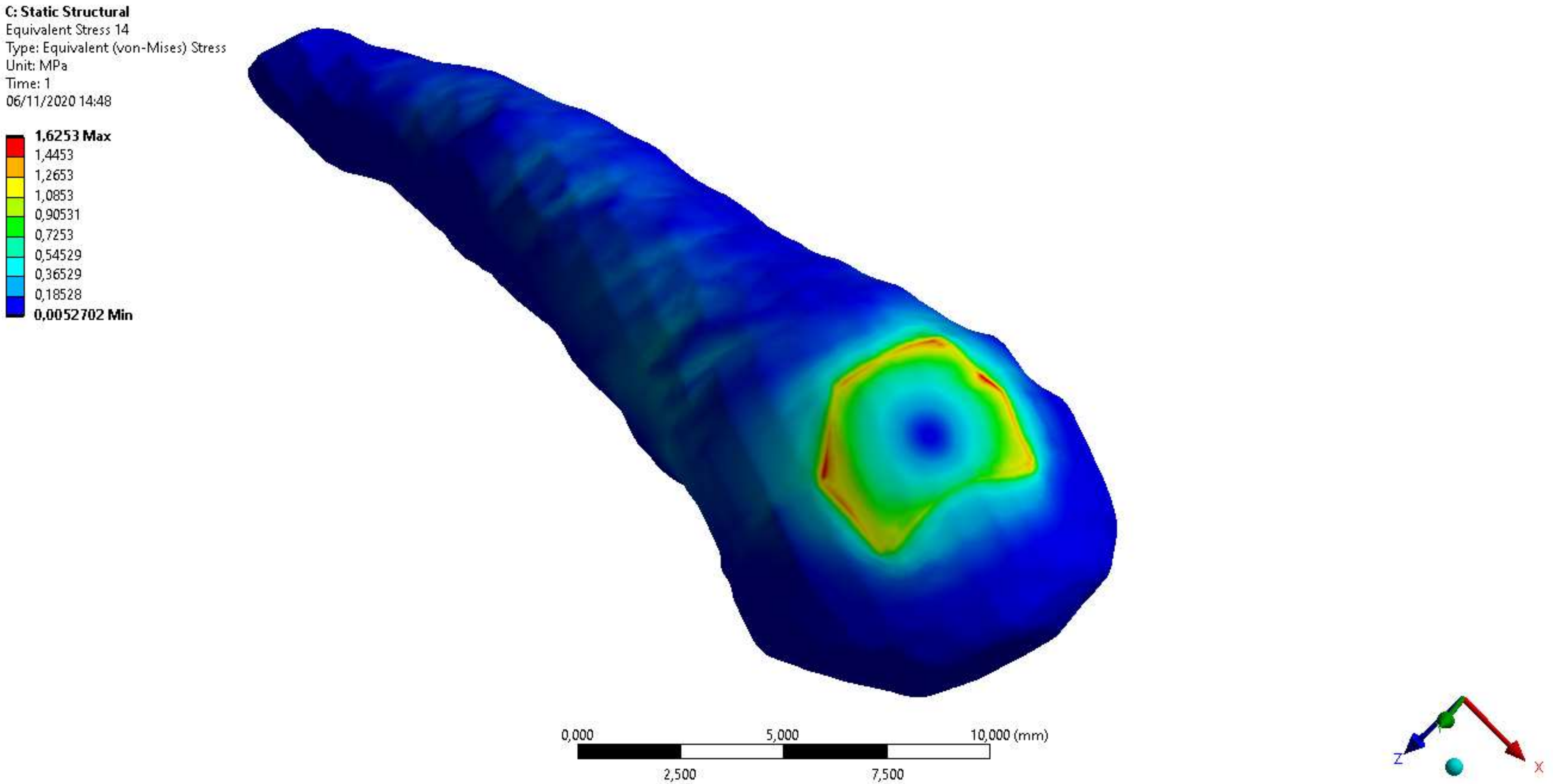
C: Static Structural
Equivalent Stress 14
Type: Equivalent (von-Mises) Stress
Unit: MPa
Time: 1
06/11/2020 14:48
1,6253 Max
1,4453
1,2653
1,0853
0,90531
0,7253
0,54529
0,36529
0,18528
0,0052702 Min
0,000
5,000
10,000 (mm)
2,500
7,500
Z
X

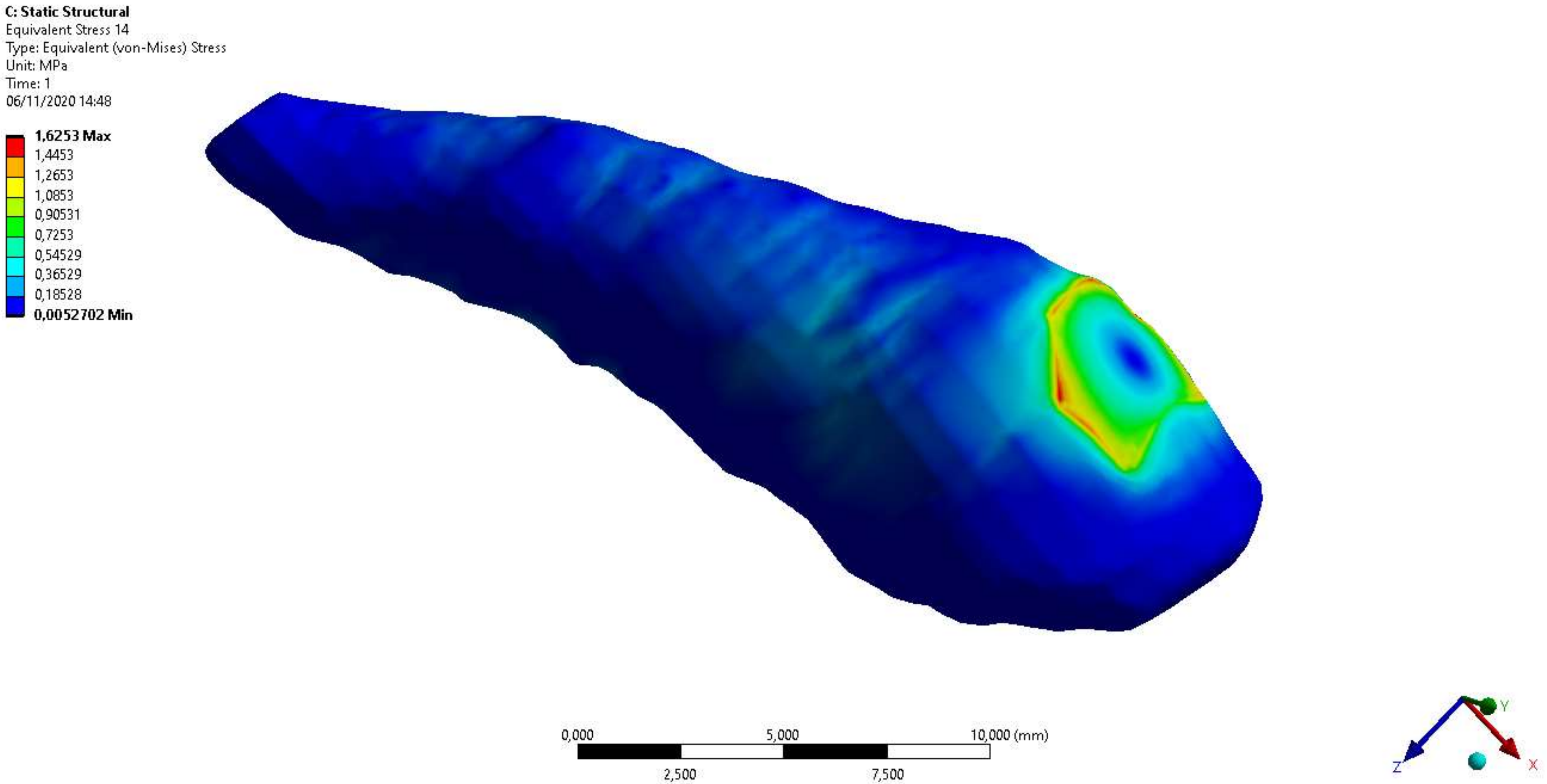
C: Static Structural
Equivalent Stress 14
Type: Equivalent (von-Mises) Stress
Unit: MPa
Time: 1
06/11/2020 14:48
1,6253 Max
1,4453
1,2653
1,0853
0,90531
0,7253
0,54529
0,36529
0,18528
0,0052702 Min
0,000
5,000
10,000 (mm)
2,500
7,500
Y
Z
X

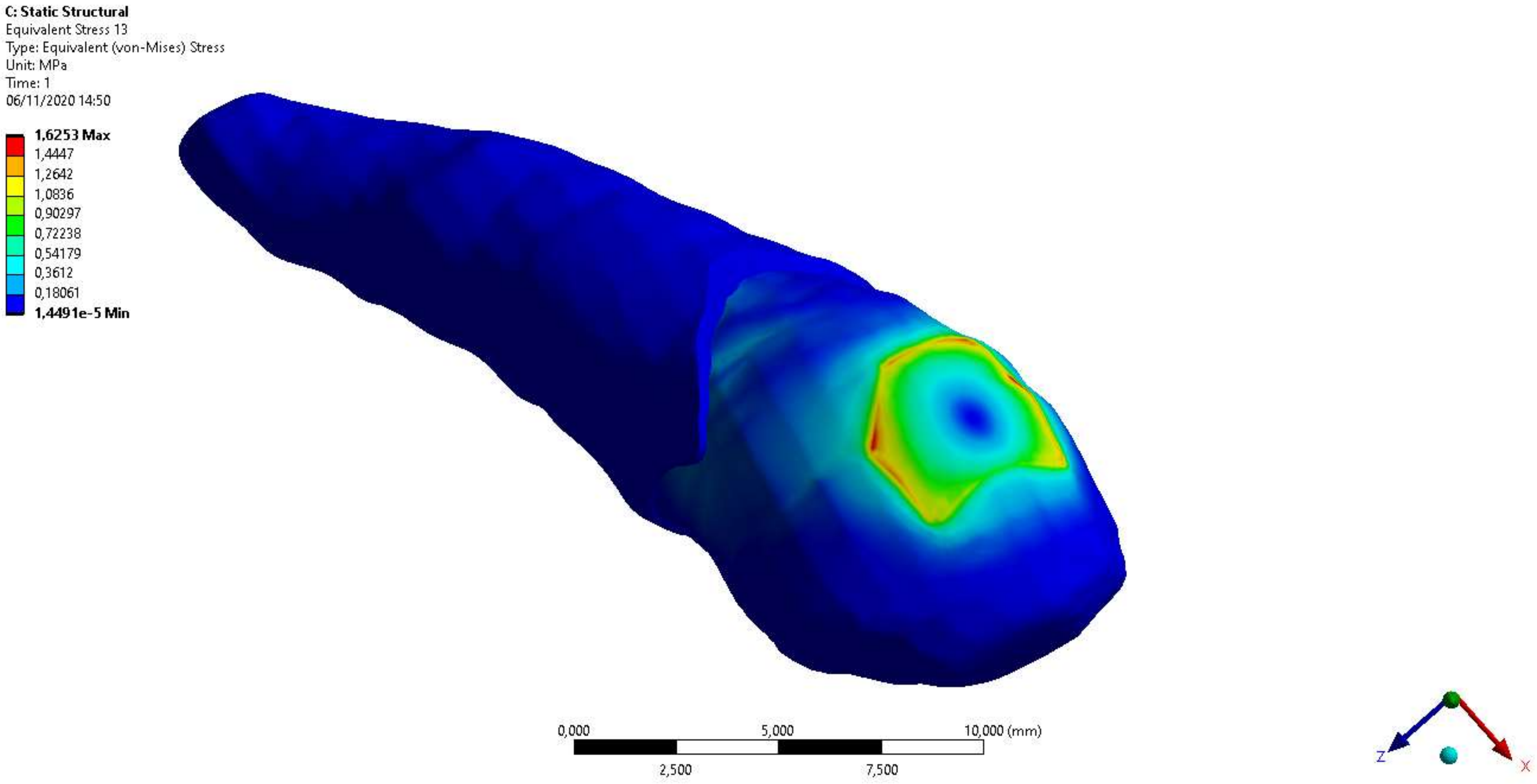
C: Static Structural
Equivalent Stress 13
Type: Equivalent (von-Mises) Stress
Unit: MPa
Time: 1
06/11/2020 14:50
1,6253 Max
1,4447
1,2642
1,0836
0,90297
0,72238
0,54179
0,3612
0,18061
1,4491e-5 Min
0,000
5,000
10,000 (mm)
2,500
7,500
Z
X

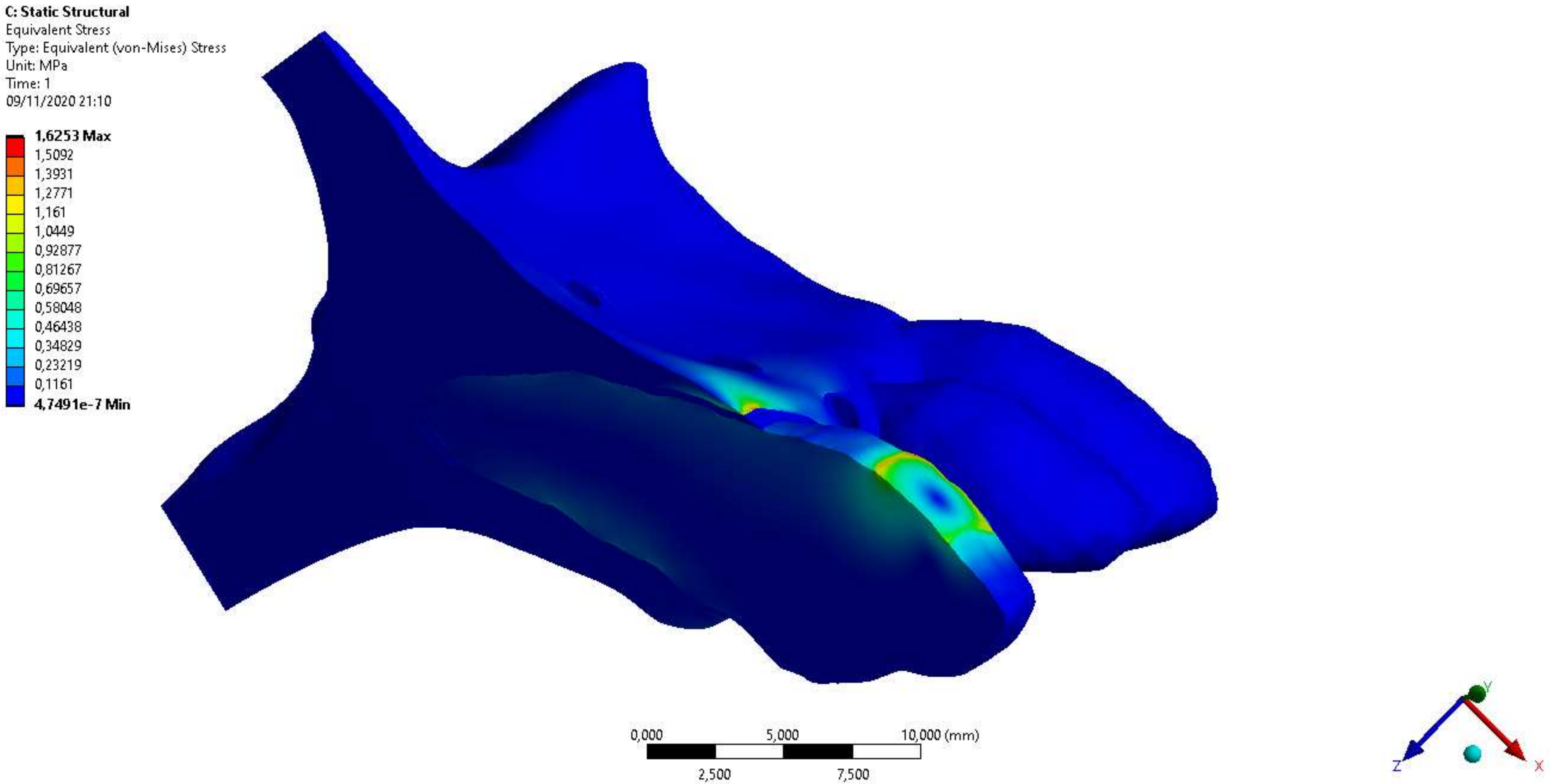
C: Static Structural
Equivalent Stress
Type: Equivalent (von-Mises) Stress
Unit: MPa
Time: 1
09/11/2020 21:10
1,6253 Max
1,5092
1,3931
1,2771
1,161
1,0449
0,92877
0,81267
0,69657
0,58048
0,46438
0,34829
0,23219
0,1161
4,7491e-7 Min
0,000
5,000
10,000 (mm)
2,500
7,500
Y
Z
X

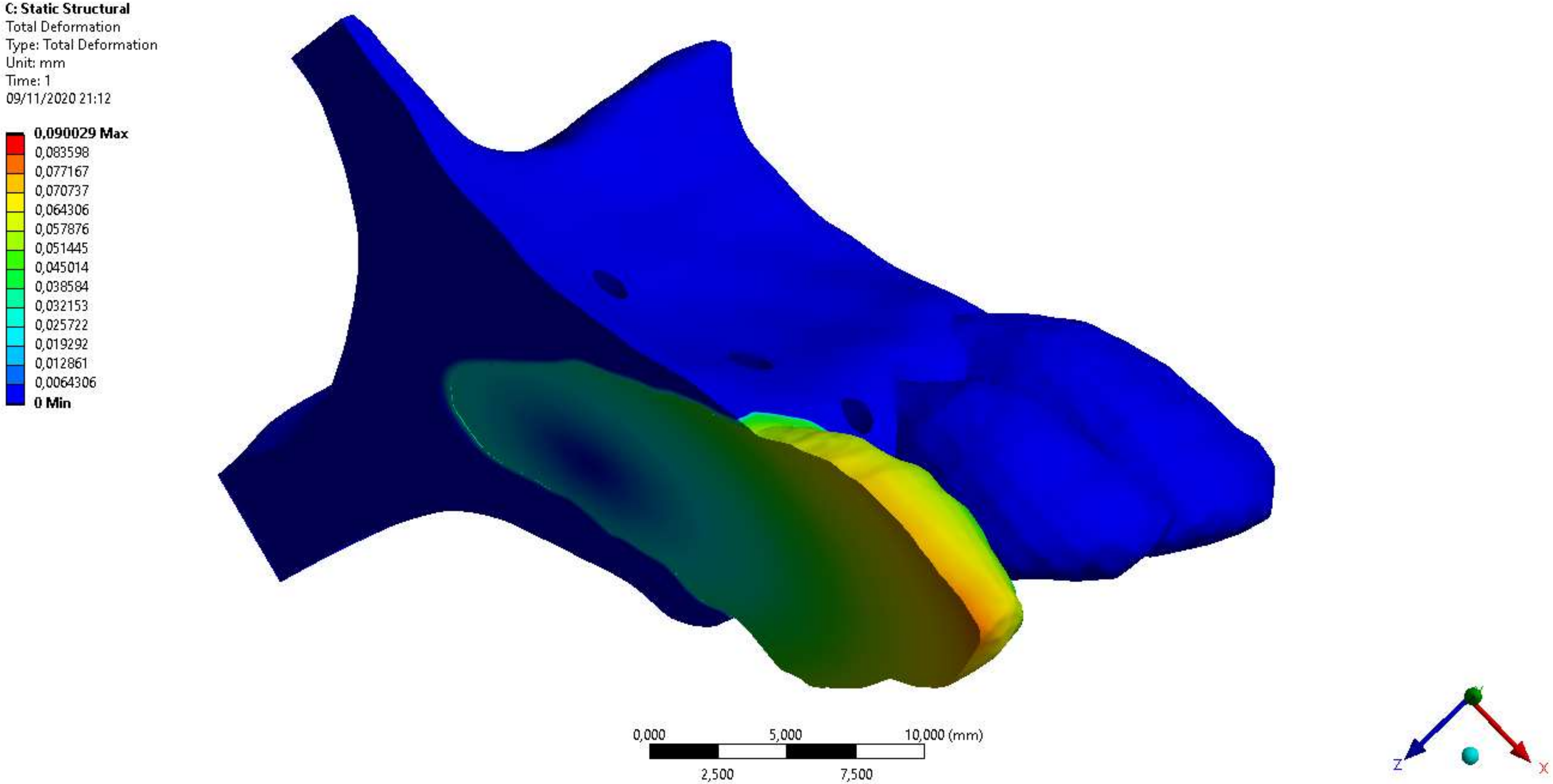
C: Static Structural
Total Deformation
Type: Total Deformation
Unit: mm
Time: 1
09/11/2020 21:12
0,090029 Max
0,083598
0,077167
0,070737
0,064306
0,057876
0,051445
0,045014
0,038584
0,032153
0,025722
0,019292
0,012861
0,0064306
0 Min
0,000
5,000
10,000 (mm)
2,500
7,500
Y
Z
X

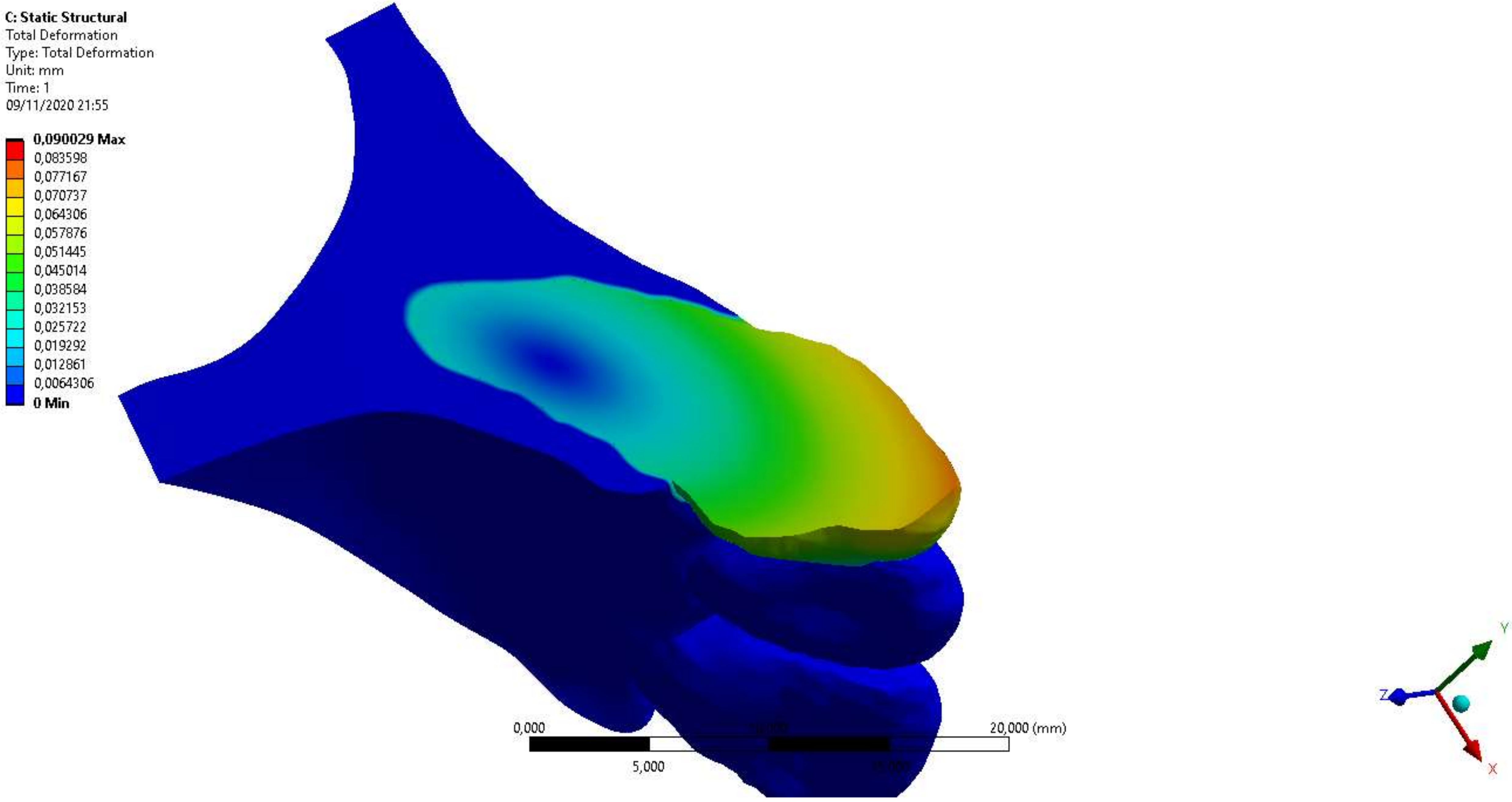
C: Static Structural
Total Deformation
Type: Total Deformation
Unit: mm
Time: 1
09/11/2020 21:55
0,090029 Max
0,083598
0,077167
0,070737
0,064306
0,057876
0,051445
0,045014
0,038584
0,032153
0,025722
0,019292
0,012861
0,0064306
0 Min
0,000
5,000
20,000 (mm)
Y
Z
X

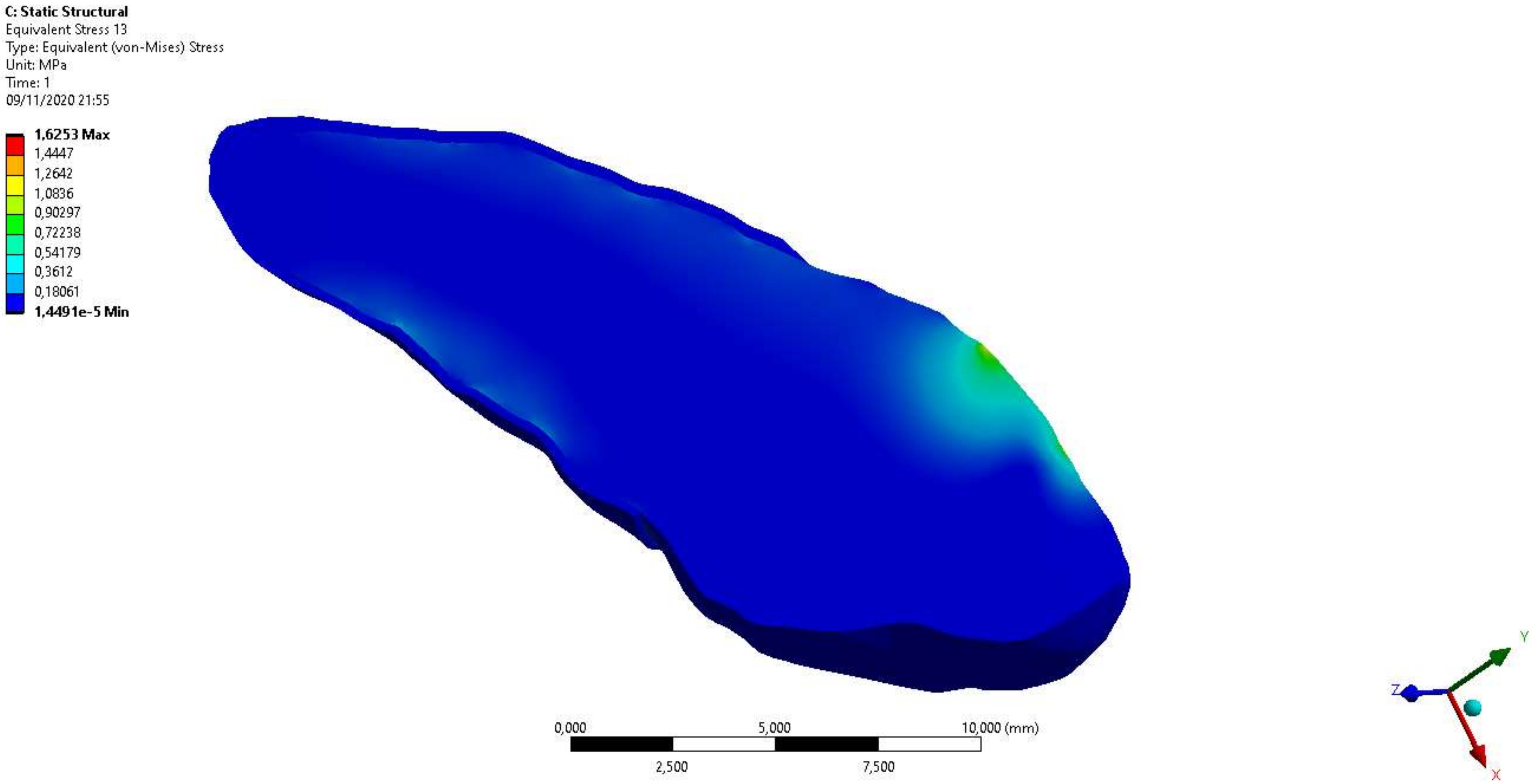
C: Static Structural
Equivalent Stress 13
Type: Equivalent (von-Mises) Stress
Unit: MPa
Time: 1
09/11/2020 21:55
1,6253 Max
1,4447
1,2642
1,0836
0,90297
0,72238
0,54179
0,3612
0,18061
1,4491e-5 Min
0,000
5,000
10,000 (mm)
2,500
7,500
Y
Z
X

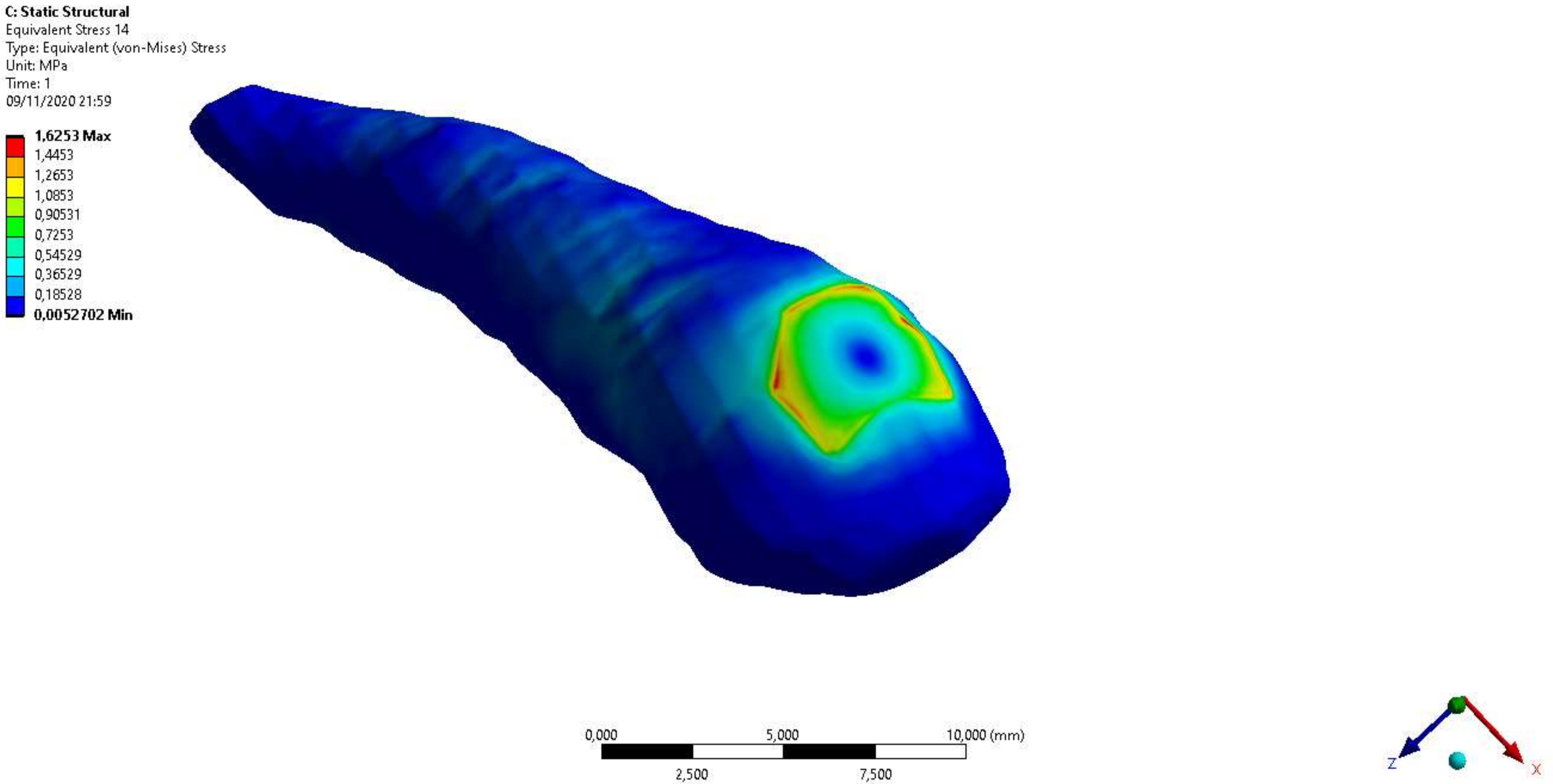
C: Static Structural
Equivalent Stress 14
Type: Equivalent (von-Mises) Stress
Unit: MPa
Time: 1
09/11/2020 21:59
1,6253 Max
1,4453
1,2653
1,0853
0,90531
0,7253
0,54529
0,36529
0,18528
0,0052702 Min
0,000
5,000
10,000 (mm)
2,500
7,500
Z
X

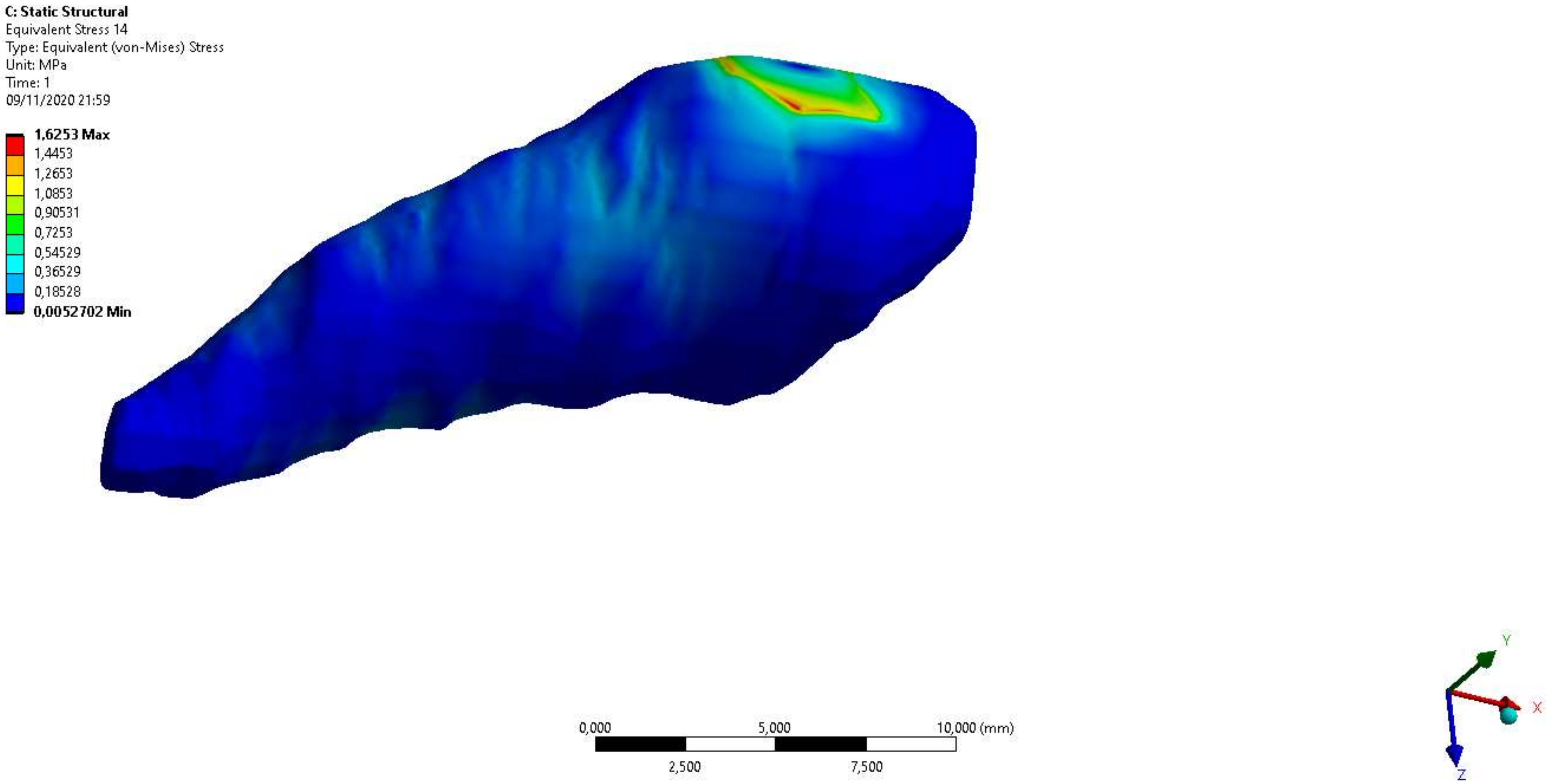
C: Static Structural
Equivalent Stress 14
Type: Equivalent (von-Mises) Stress
Unit: MPa
Time: 1
09/11/2020 21:59
1,6253 Max
1,4453
1,2653
1,0853
0,90531
0,7253
0,54529
0,36529
0,18528
0,0052702 Min
0,000
5,000
10,000 (mm)
2,500
7,500
Y
X
Z

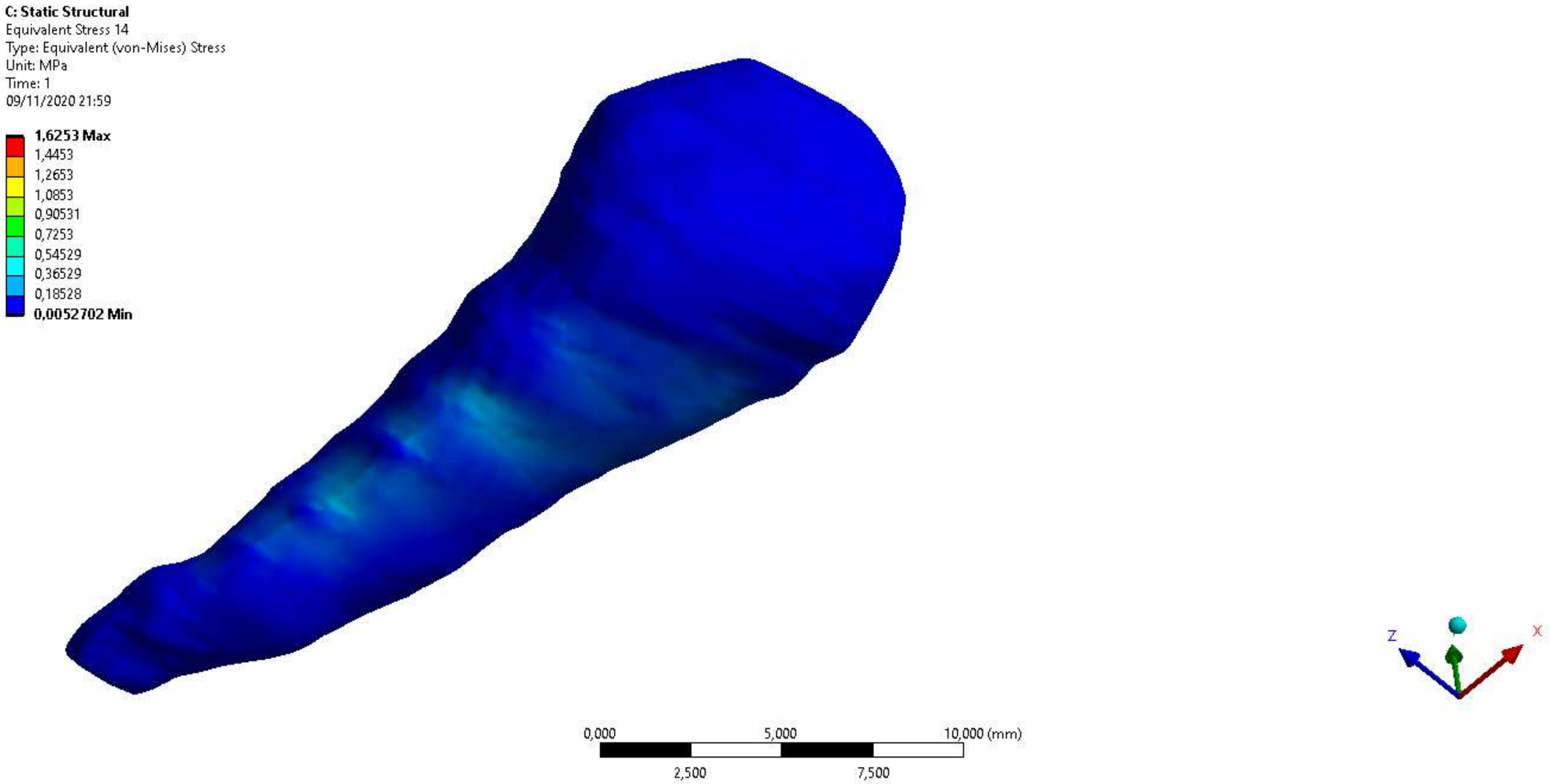
C: Static Structural
Equivalent Stress 14
Type: Equivalent (von-Mises) Stress
Unit: MPa
Time: 1
09/11/2020 21:59
1,6253 Max
1,4453
1,2653
1,0853
0,90531
0,7253
0,54529
0,36529
0,18528
0,0052702 Min
0,000
5,000
10,000 (mm)
2,500
7,500
Z
X
