# Supplementary material for: Micro-osteoperforation for enhancement of orthodontic movement: A mechanical analysis using the finite element method
Source: PLoS One. 2024 Aug 19;19(8):e0308739. doi: 10.1371/journal.pone.0308739 (PMC11332926; doi:10.1371/journal.pone.0308739)

S9. Comparison of images 1

Maxilla without perforations

Dente

Fatigue Data at zero mean stress comes from 1998 ASME BPV Code, Section 8, Div 2, Table 5-110.1

Density	1,96e-06 kg/mm³

Structural

Isotropic Elasticity

Derive from	Young's Modulus and Poisson's Ratio
Young's Modulus	14700 MPa
Poisson's Ratio	0,31000
Bulk Modulus	12895 MPa
Shear Modulus	5610,7 MPa
Isotropic Secant Coefficient of Thermal Expansion	1,2e-05 1/°C
Compressive Ultimate Strength	0 MPa
Compressive Yield Strength	250,00 MPa

Maxilla with perforations

Dente

Fatigue Data at zero mean stress comes from 1998 ASME BPV Code, Section 8, Div 2, Table 5-110.1

Density	1,96e-06 kg/mm³

Structural

Isotropic Elasticity

Derive from	Young's Modulus and Poisson's Ratio
Young's Modulus	14700 MPa
Poisson's Ratio	0,31000
Bulk Modulus	12895 MPa
Shear Modulus	5610,7 MPa
Isotropic Secant Coefficient of Thermal Expansion	1,2e-05 1/°C
Compressive Ultimate Strength	0 MPa
Compressive Yield Strength	250,00 MPa

Maxilla without perforations with moment

Dente

Fatigue Data at zero mean stress comes from 1998 ASME BPV Code, Section 8, Div 2, Table 5-110.1

Density	1,96e-06 kg/mm³

Structural

Isotropic Elasticity

Derive from	Young's Modulus and Poisson's Ratio
Young's Modulus	14700 MPa
Poisson's Ratio	0,31000
Bulk Modulus	12895 MPa
Shear Modulus	5610,7 MPa
Isotropic Secant Coefficient of Thermal Expansion	1,2e-05 1/°C
Compressive Ultimate Strength	0 MPa
Compressive Yield Strength	250,00 MPa

Maxilla with perforations with moment

Dente

Fatigue Data at zero mean stress comes from 1998 ASME BPV Code, Section 8, Div 2, Table 5-110.1

Density	1,96e-06 kg/mm³

Structural

Isotropic Elasticity

Derive from	Young's Modulus and Poisson's Ratio
Young's Modulus	14700 MPa
Poisson's Ratio	0,31000
Bulk Modulus	12895 MPa
Shear Modulus	5610,7 MPa
Isotropic Secant Coefficient of Thermal Expansion	1,2e-05 1/°C
Compressive Ultimate Strength	0 MPa
Compressive Yield Strength	250,00 MPa

Maxilla without perforations

LigamentoPeriodotal

Fatigue Data at zero mean stress comes from 1998 ASME BPV Code, Section 8, Div 2, Table 5-110.1

Density	1,2e-06 kg/mm³

Structural

Isotropic Elasticity

Derive from	Young's Modulus and Poisson's Ratio
Young's Modulus	0,068000 MPa
Poisson's Ratio	0,45000
Bulk Modulus	0,22667 MPa
Shear Modulus	0,023448 MPa

Maxilla with perforations

LigamentoPeriodotal

Fatigue Data at zero mean stress comes from 1998 ASME BPV Code, Section 8, Div 2, Table 5-110.1

Density	1,2e-06 kg/mm³

Structural

Isotropic Elasticity

Derive from	Young's Modulus and Poisson's Ratio
Young's Modulus	0,068000 MPa
Poisson's Ratio	0,45000
Bulk Modulus	0,22667 MPa
Shear Modulus	0,023448 MPa

Maxilla without perforations with moment

LigamentoPeriodotal

Fatigue Data at zero mean stress comes from 1998 ASME BPV Code, Section 8, Div 2, Table 5-110.1

Density	1,2e-06 kg/mm³

Structural

Isotropic Elasticity

Derive from	Young's Modulus and Poisson's Ratio
Young's Modulus	0,068000 MPa
Poisson's Ratio	0,45000
Bulk Modulus	0,22667 MPa
Shear Modulus	0,023448 MPa

Maxilla with perforations with moment

LigamentoPeriodotal

Fatigue Data at zero mean stress comes from 1998 ASME BPV Code, Section 8, Div 2, Table 5-110.1

Density	1,2e-06 kg/mm³

Structural

Isotropic Elasticity

Derive from	Young's Modulus and Poisson's Ratio
Young's Modulus	0,068000 MPa
Poisson's Ratio	0,45000
Bulk Modulus	0,22667 MPa
Shear Modulus	0,023448 MPa

Maxilla without perforations

Osso Medular

Density	4,1e-07 kg/mm³

Structural

Isotropic Elasticity

Derive from	Young's Modulus and Poisson's Ratio
Young's Modulus	1370,0 MPa
Poisson's Ratio	0,30000
Bulk Modulus	1141,7 MPa
Shear Modulus	526,92 MPa

Maxilla with perforations

Osso Medular

Density	4,1e-07 kg/mm³

Structural

Isotropic Elasticity

Derive from	Young's Modulus and Poisson's Ratio
Young's Modulus	1370,0 MPa
Poisson's Ratio	0,30000
Bulk Modulus	1141,7 MPa
Shear Modulus	526,92 MPa

Maxilla without perforations with moment

Osso Medular

Density	4,1e-07 kg/mm³

Structural

Isotropic Elasticity

Derive from	Young's Modulus and Poisson's Ratio
Young's Modulus	1370,0 MPa
Poisson's Ratio	0,30000
Bulk Modulus	1141,7 MPa
Shear Modulus	526,92 MPa

Maxilla with perforations with moment

Osso Medular

Density	4,1e-07 kg/mm³

Structural

Isotropic Elasticity

Derive from	Young's Modulus and Poisson's Ratio
Young's Modulus	1370,0 MPa
Poisson's Ratio	0,30000
Bulk Modulus	1141,7 MPa
Shear Modulus	526,92 MPa

Maxilla without perforations

Osso Cortical Isotropico

Density	1,99e-06 kg/mm³

Structural

Isotropic Elasticity

Derive from	Young's Modulus and Poisson's Ratio
Young's Modulus	13700 MPa
Poisson's Ratio	0,30000
Bulk Modulus	11417 MPa
Shear Modulus	5269,2 MPa

Maxilla with perforations

Osso Cortical Isotropico

Density	1,99e-06 kg/mm³

Structural

Isotropic Elasticity

Derive from	Young's Modulus and Poisson's Ratio
Young's Modulus	13700 MPa
Poisson's Ratio	0,30000
Bulk Modulus	11417 MPa
Shear Modulus	5269,2 MPa

Maxilla without perforations with moment

Osso Cortical Isotropico

Density	1,99e-06 kg/mm³

Structural

Isotropic Elasticity

Derive from	Young's Modulus and Poisson's Ratio
Young's Modulus	13700 MPa
Poisson's Ratio	0,30000
Bulk Modulus	11417 MPa
Shear Modulus	5269,2 MPa

Maxilla with perforations with moment

Osso Cortical Isotropico

Density	1,99e-06 kg/mm³

Structural

Isotropic Elasticity

Derive from	Young's Modulus and Poisson's Ratio
Young's Modulus	13700 MPa
Poisson's Ratio	0,30000
Bulk Modulus	11417 MPa
Shear Modulus	5269,2 MPa

Maxilla with perforations

Maxilla with perforations with moment

Tecido Granulomatoso

Density	4,1e-07 kg/mm³

Structural

Isotropic Elasticity

Derive from	Young's Modulus and Poisson's Ratio
Young's Modulus	1,0000 MPa
Poisson's Ratio	0,49000
Bulk Modulus	16,667 MPa
Shear Modulus	0,33557 MPa

Maxilla without perforations

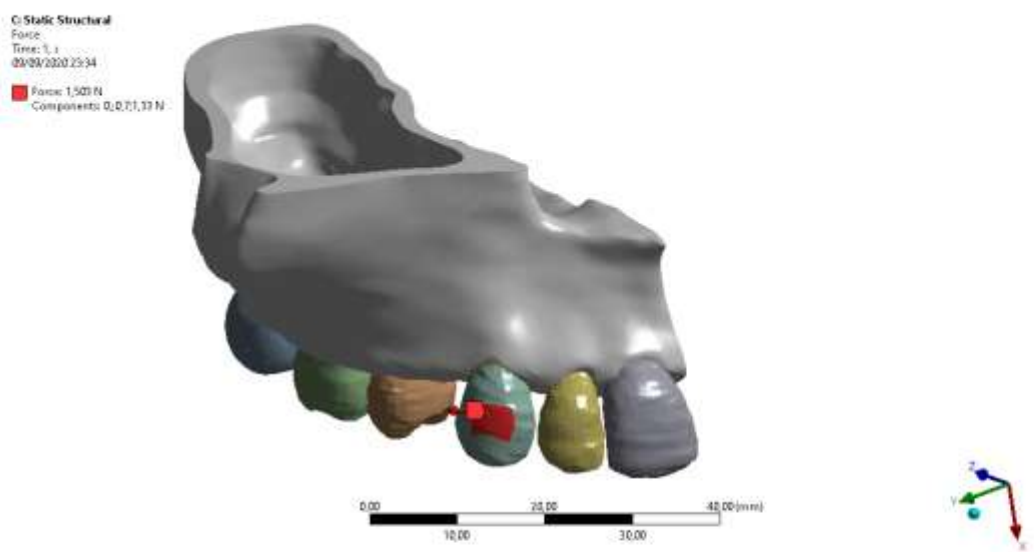

Maxilla with perforations

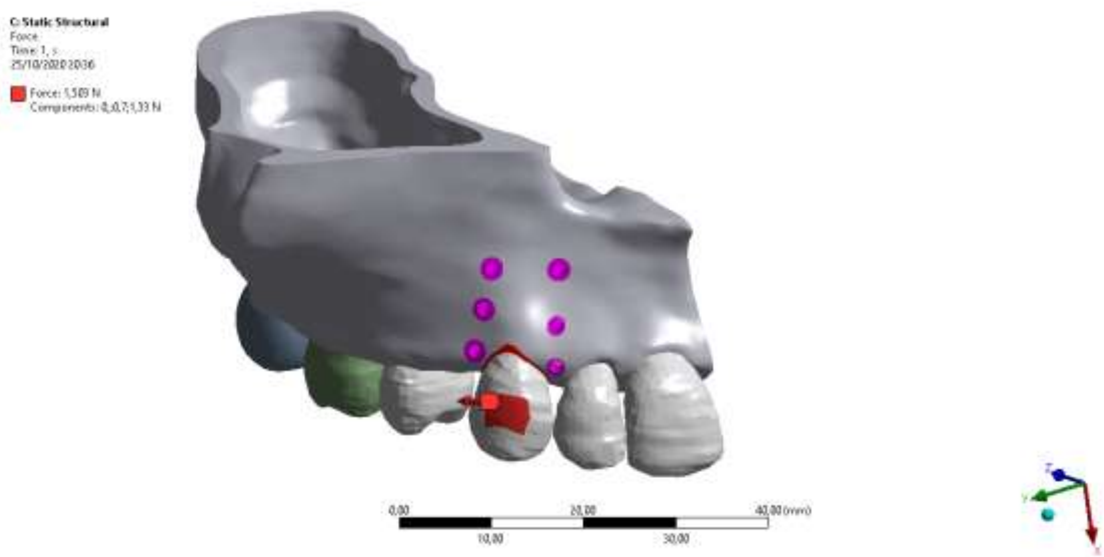

Maxilla without perforations with moment

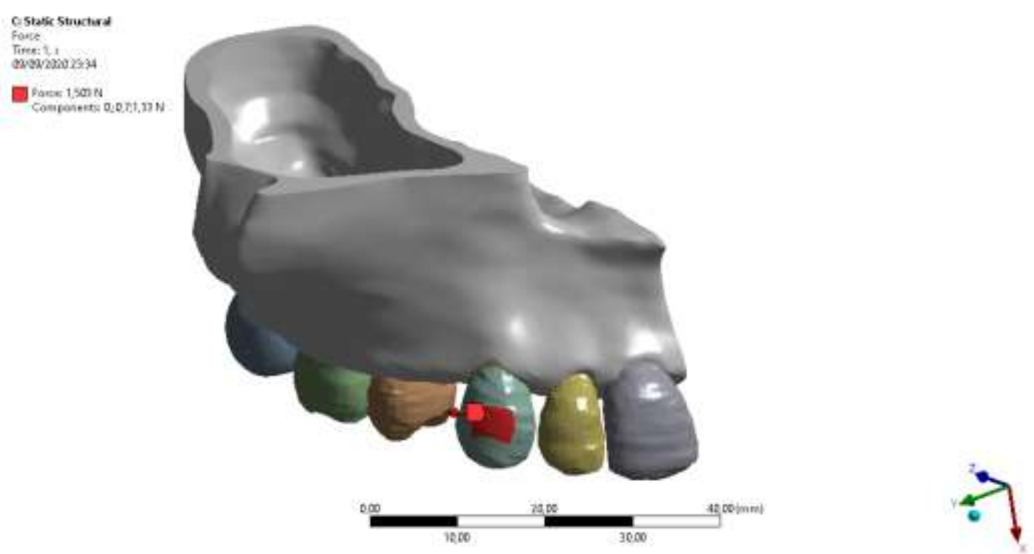

Maxilla with perforations with moment

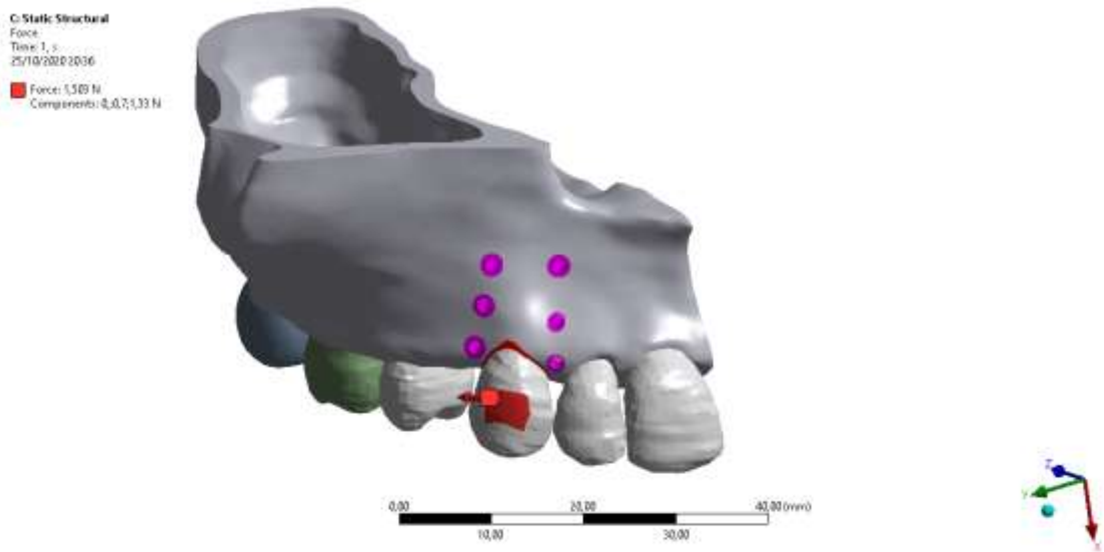

Maxilla with perforations with moment

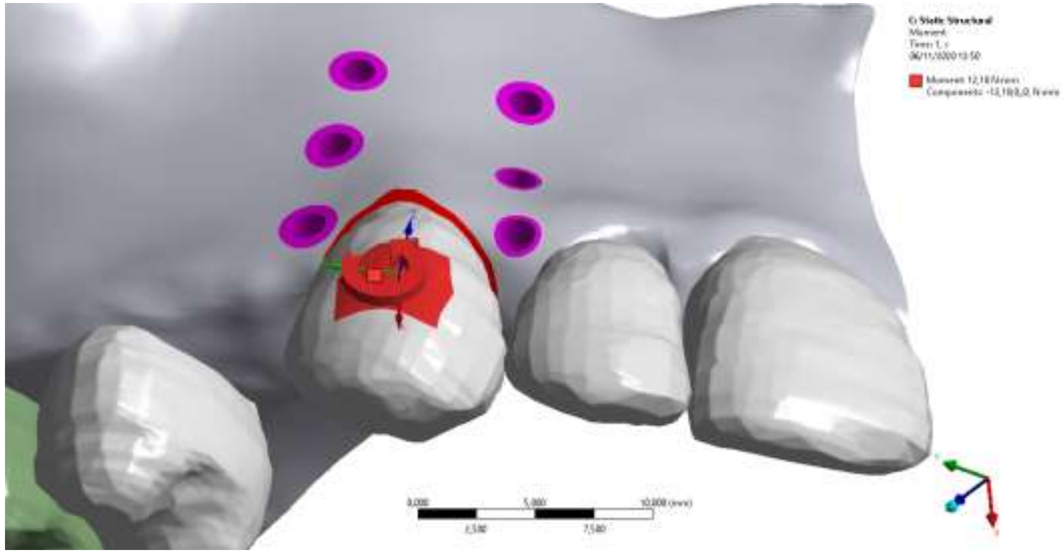

Maxilla with perforations

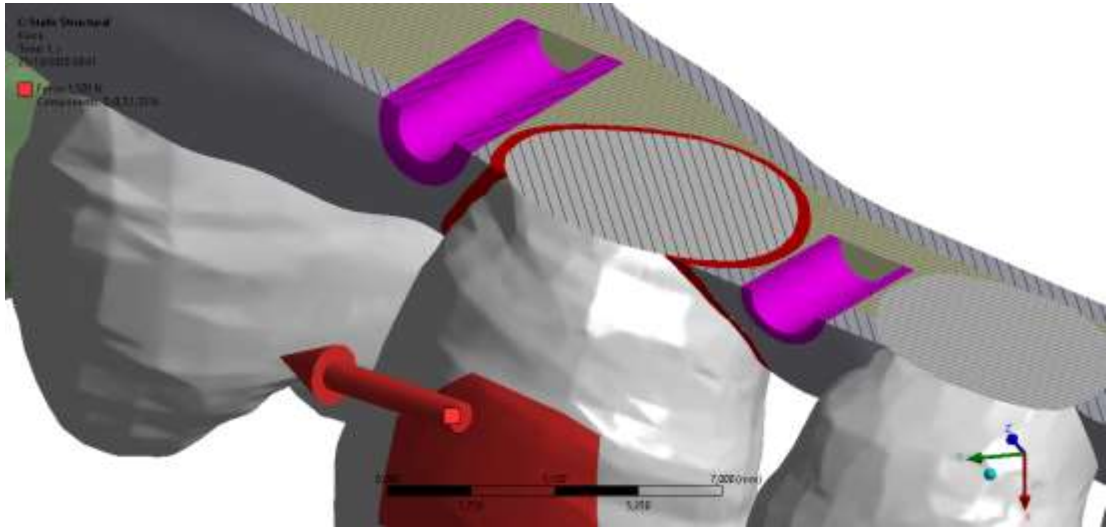

Maxilla with perforations with moment

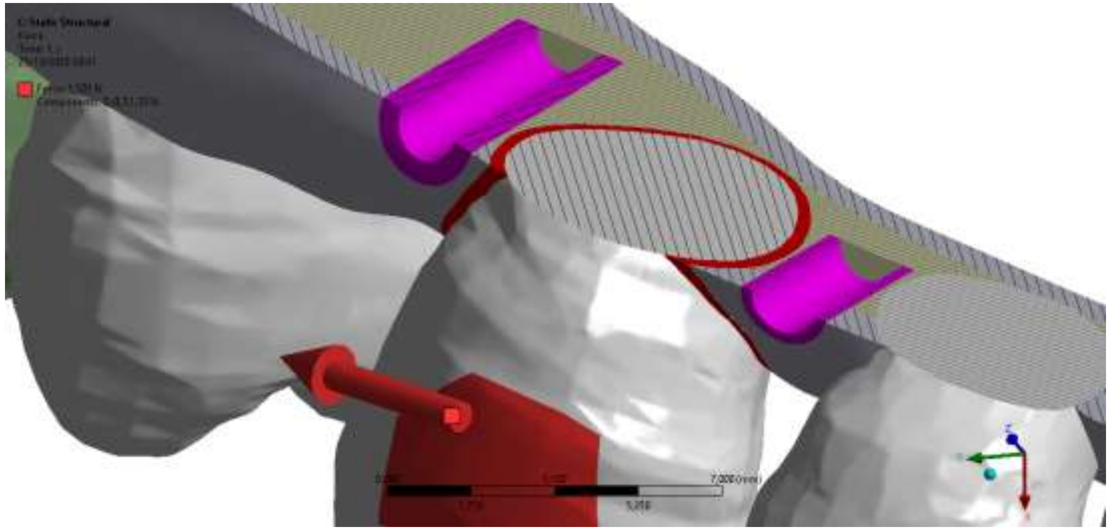

Maxilla without perforations with moment

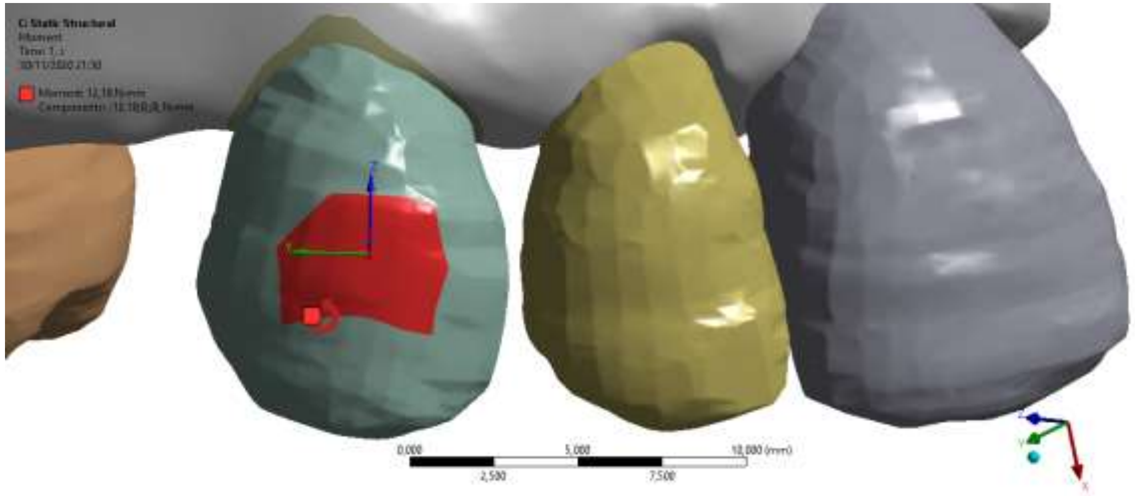

1-

Maxilla without perforations

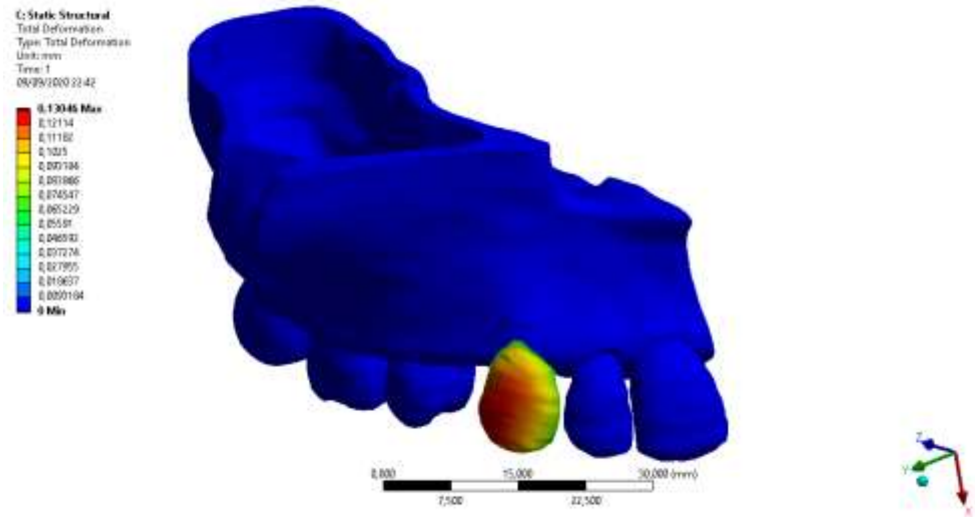

Maxilla with perforations

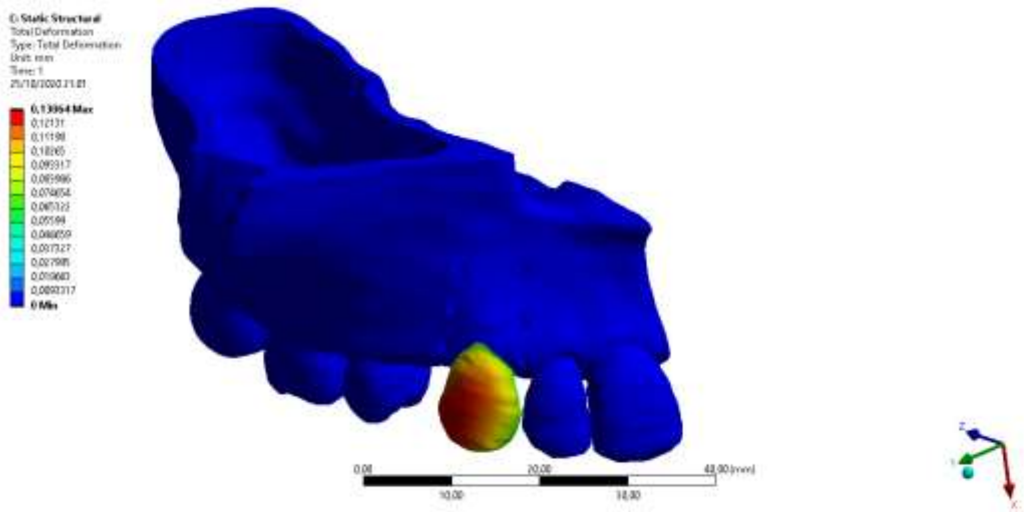

Maxilla without perforations with moment

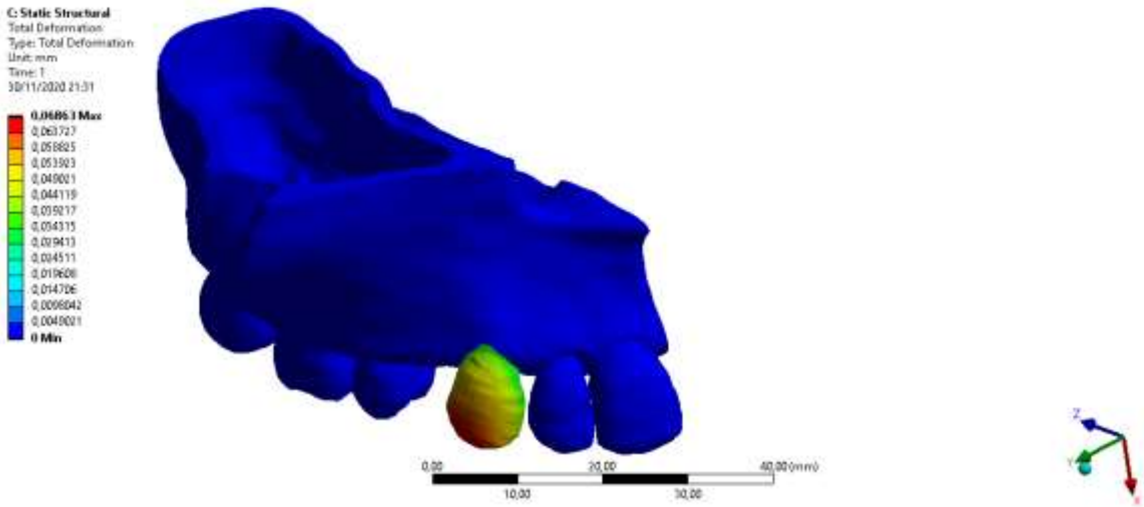

Maxilla with perforations with moment

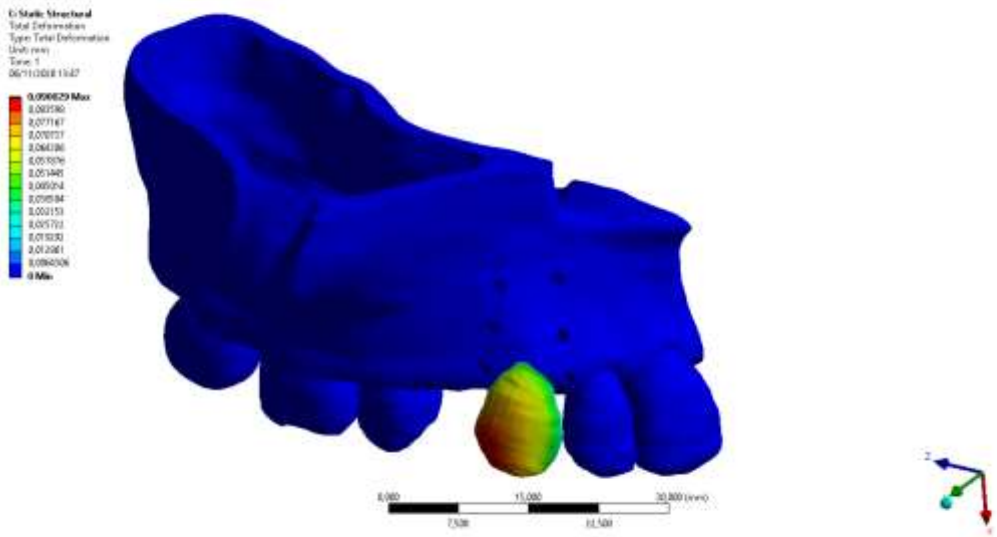

2-

Maxilla without perforations

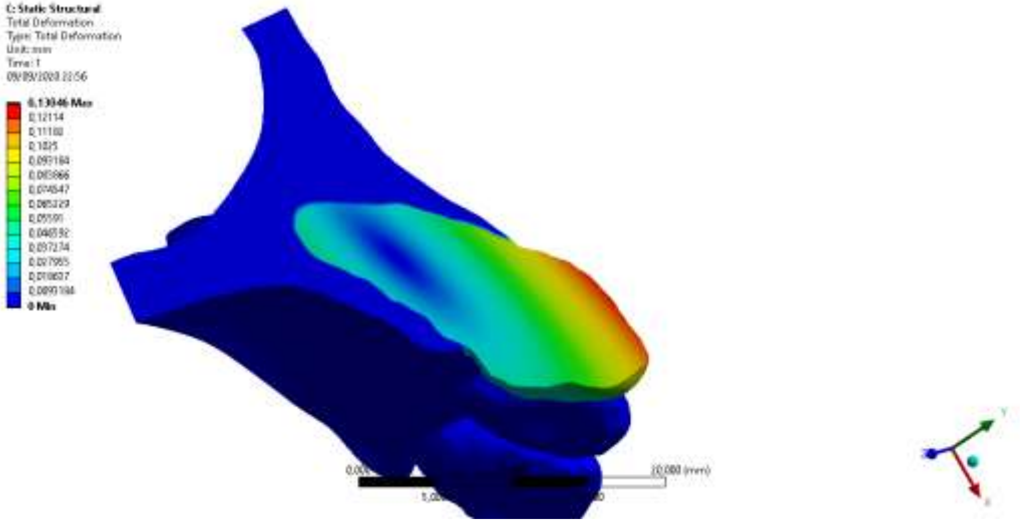

Maxilla with perforations

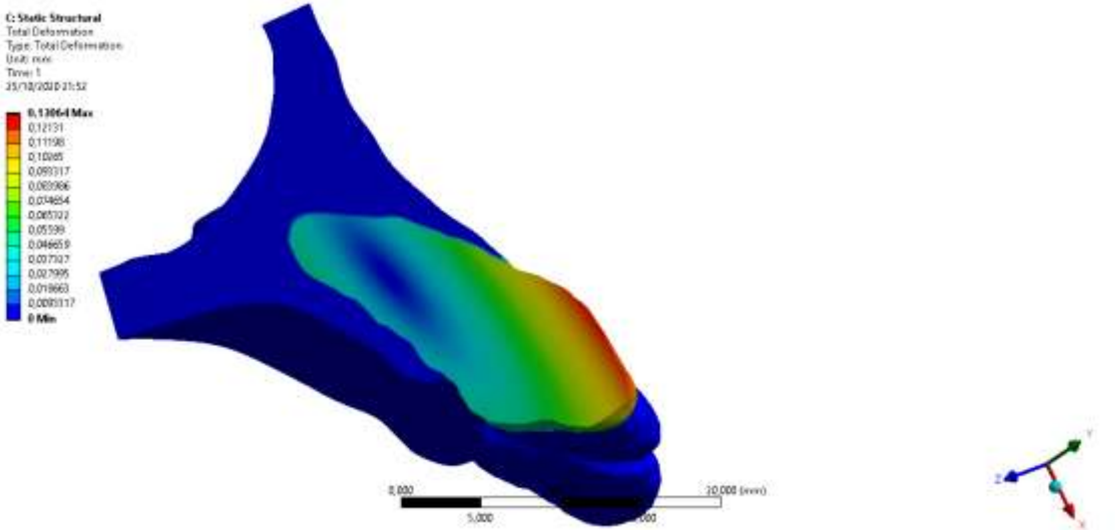

Maxilla without perforations with moment

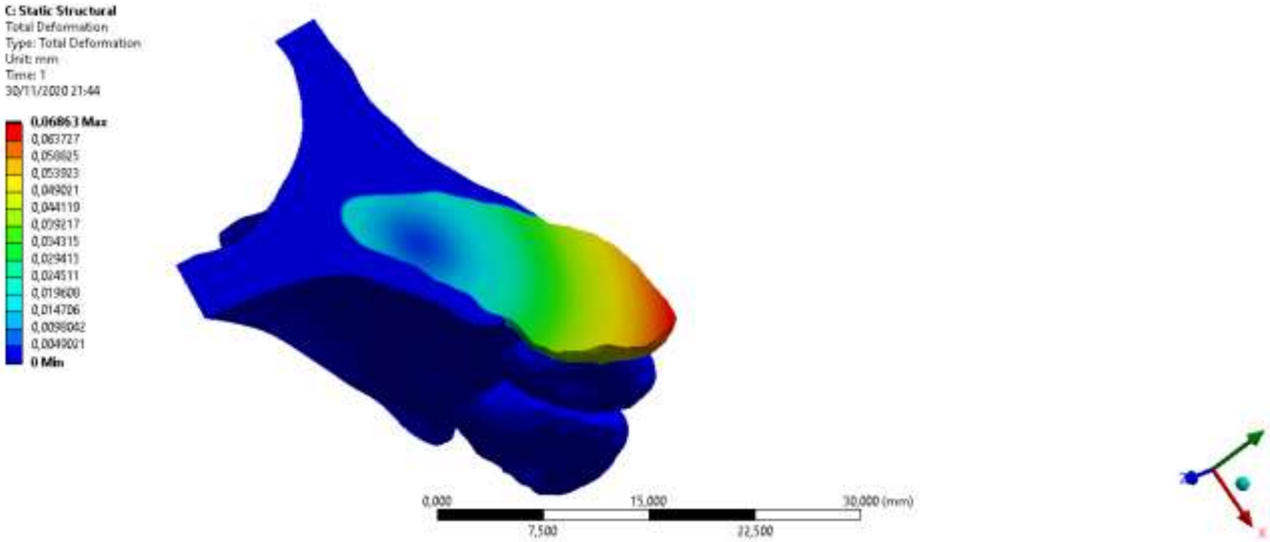

Maxilla with perforations with moment

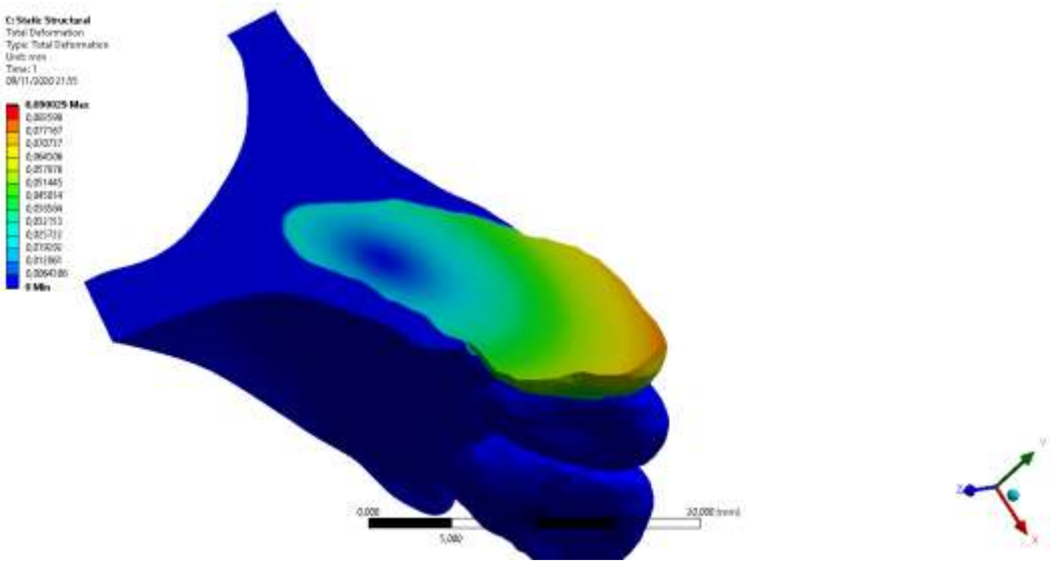

Maxilla without perforations

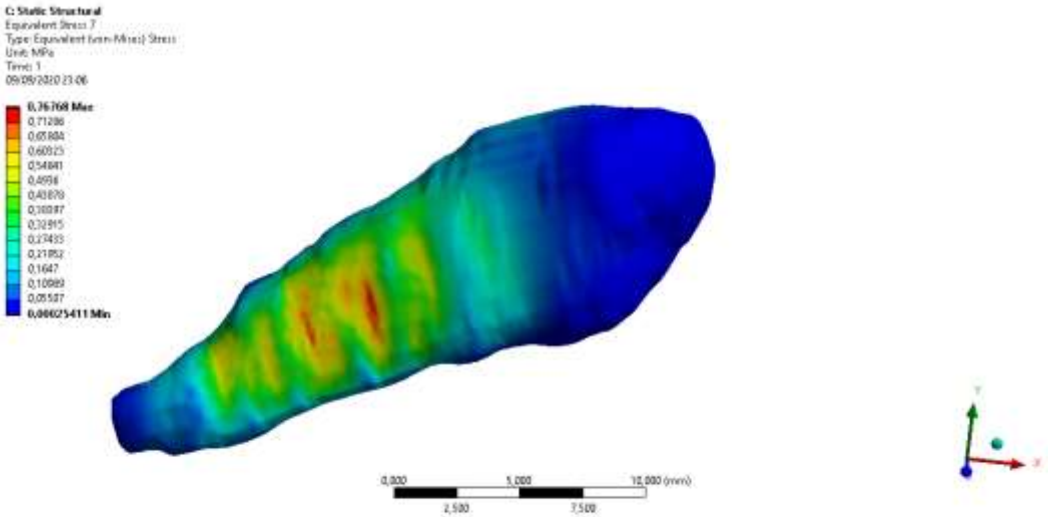

Maxilla with perforations

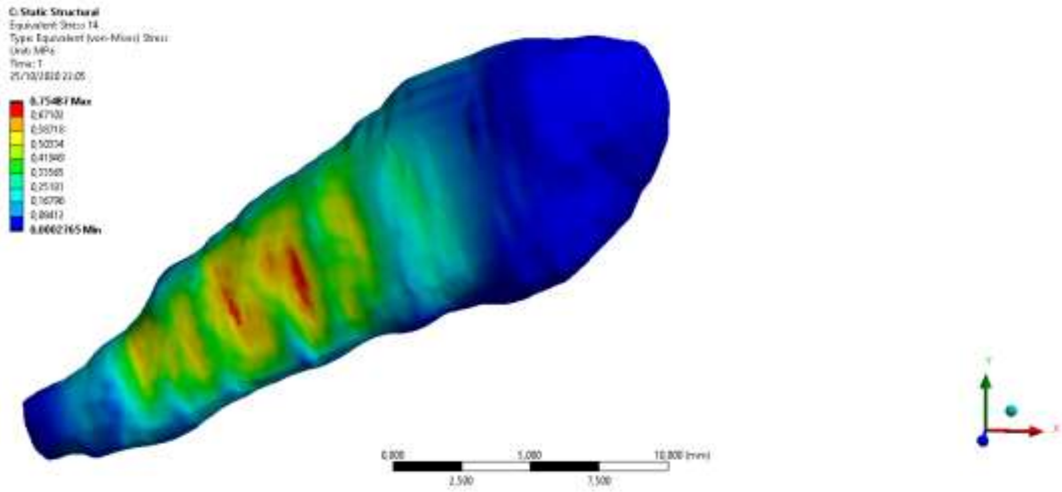

Maxilla without perforations with moment

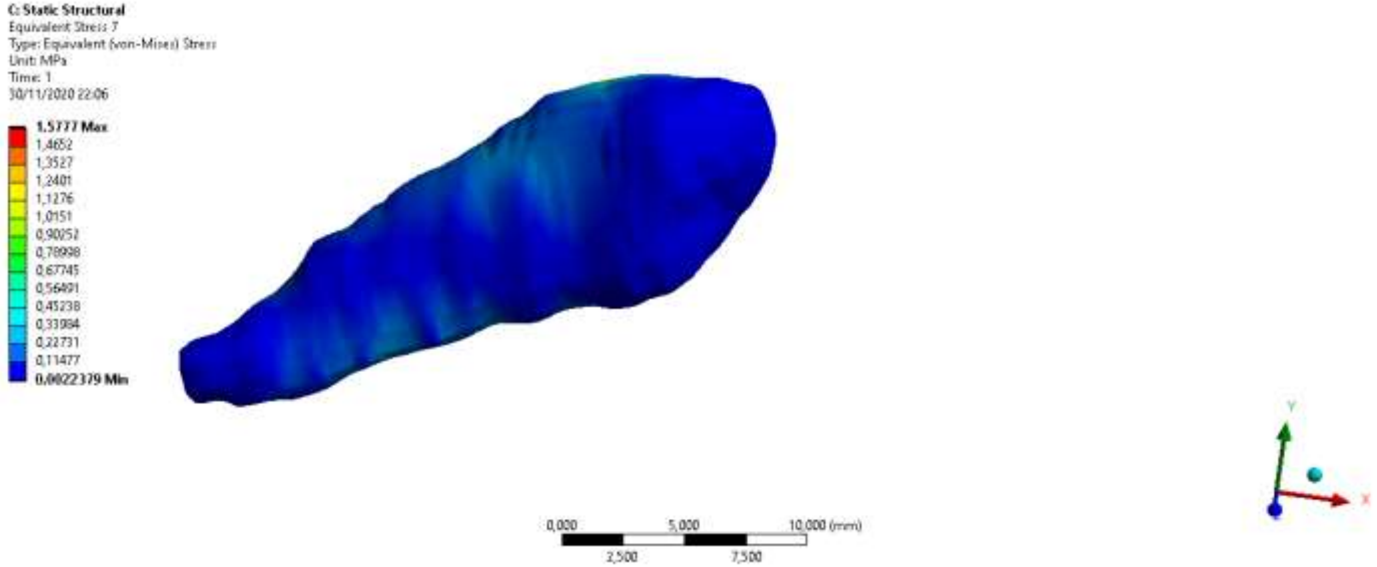

Maxilla with perforations with moment

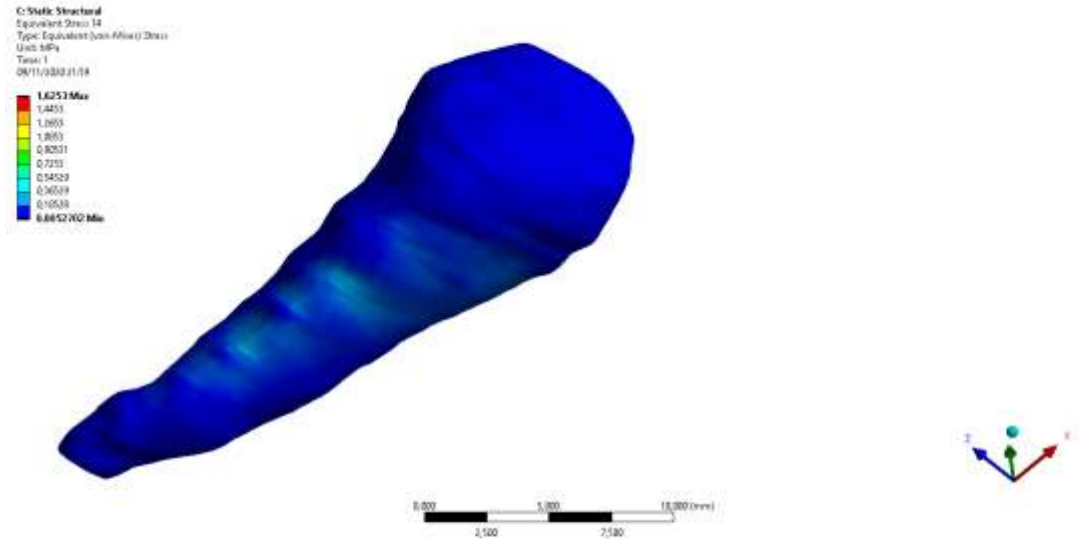

Maxilla without perforations

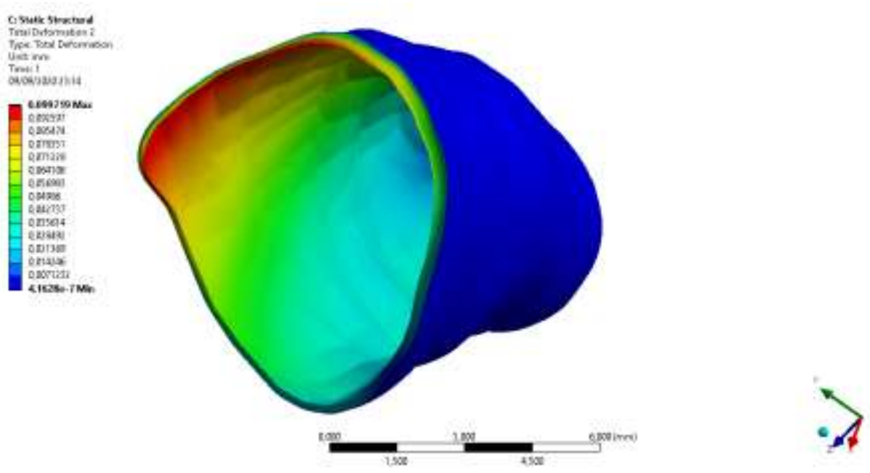

Maxilla with perforations

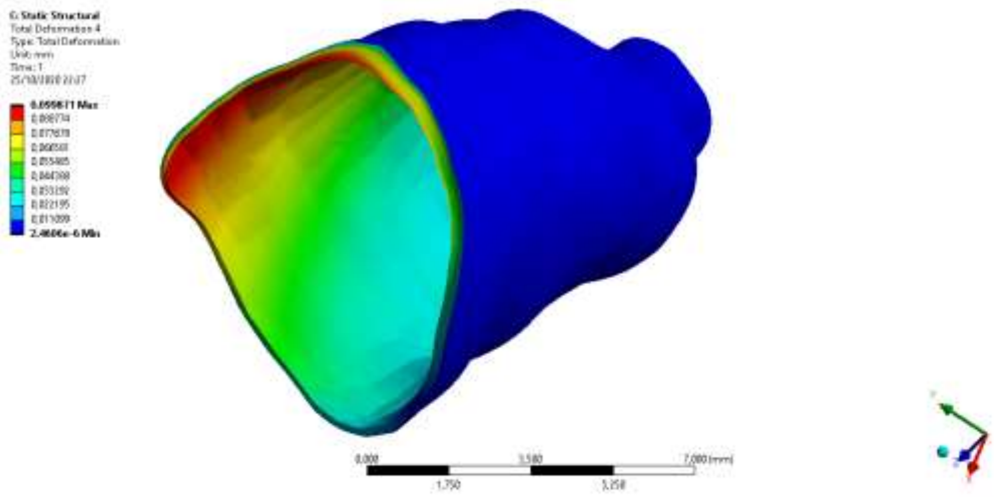

Maxilla without perforations with moment

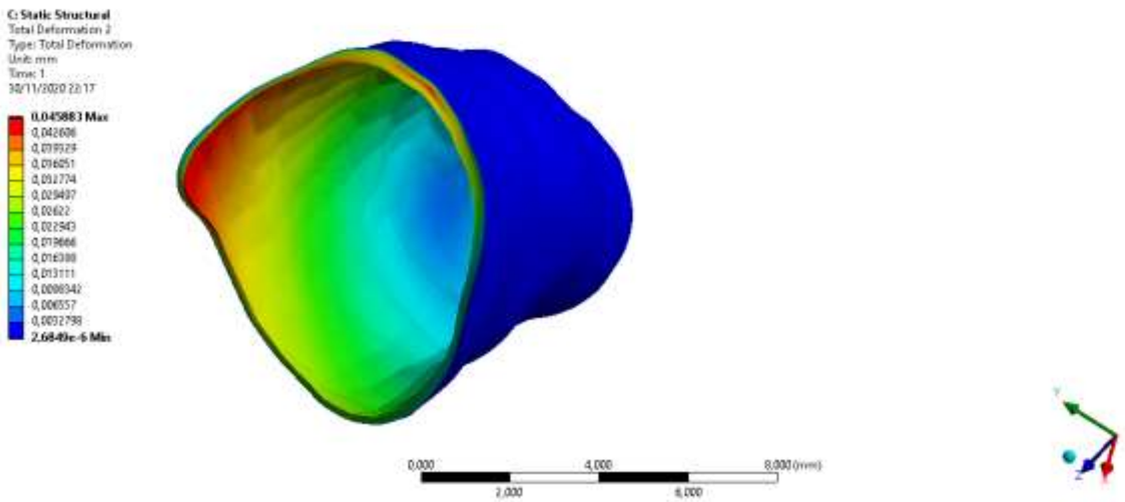

Maxilla with perforations with moment

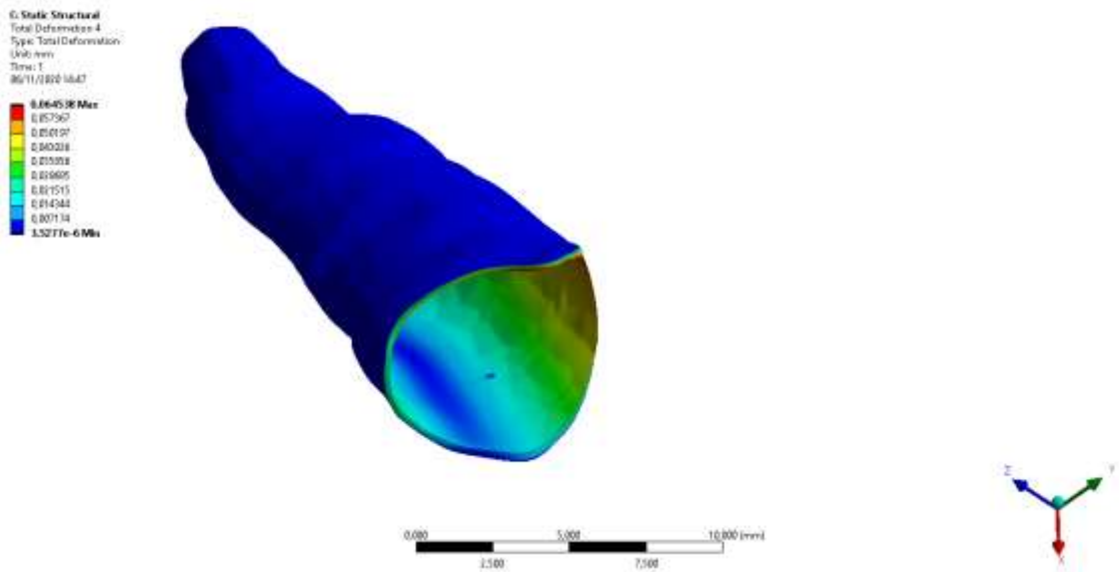

3-

Maxilla without perforations

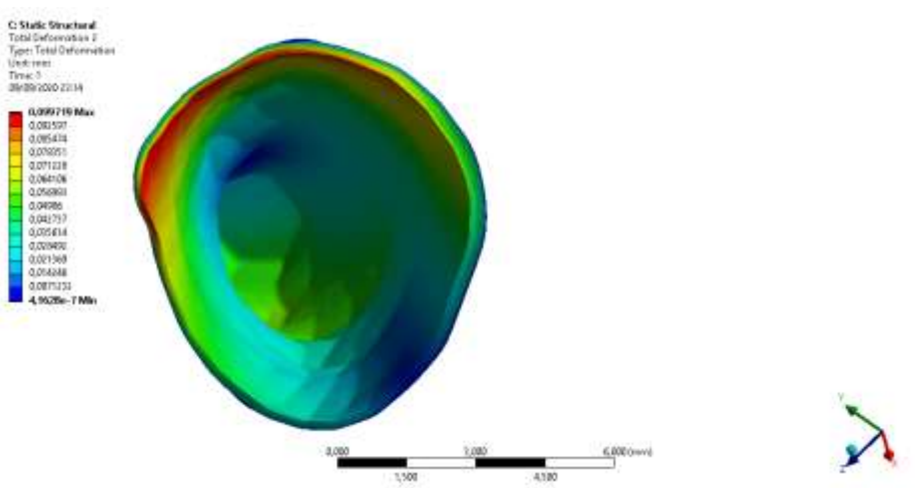

Maxilla with perforations

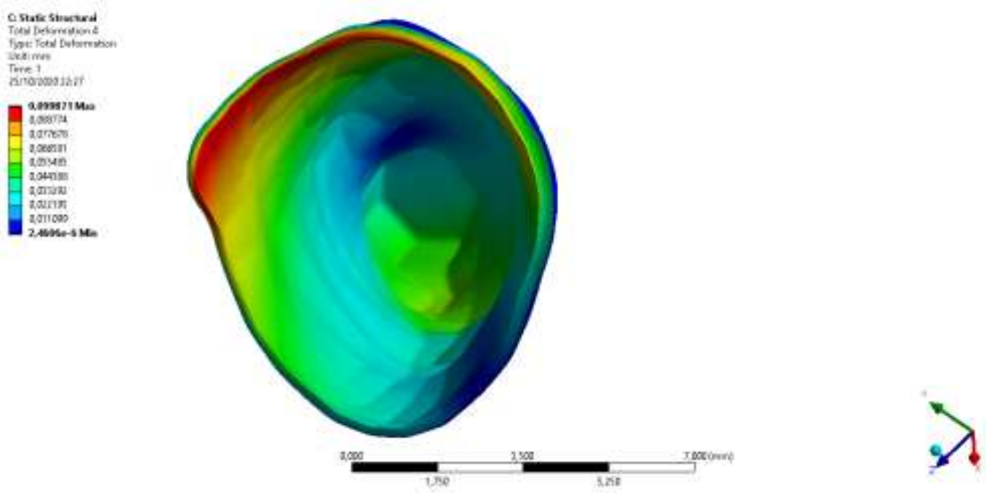

Maxilla without perforations with moment

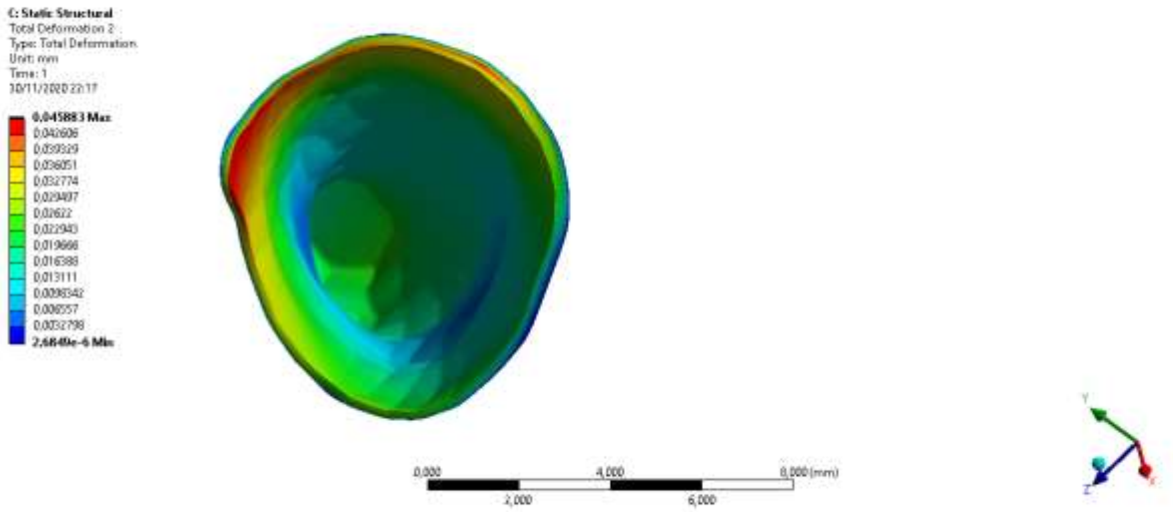

Maxilla with perforations with moment

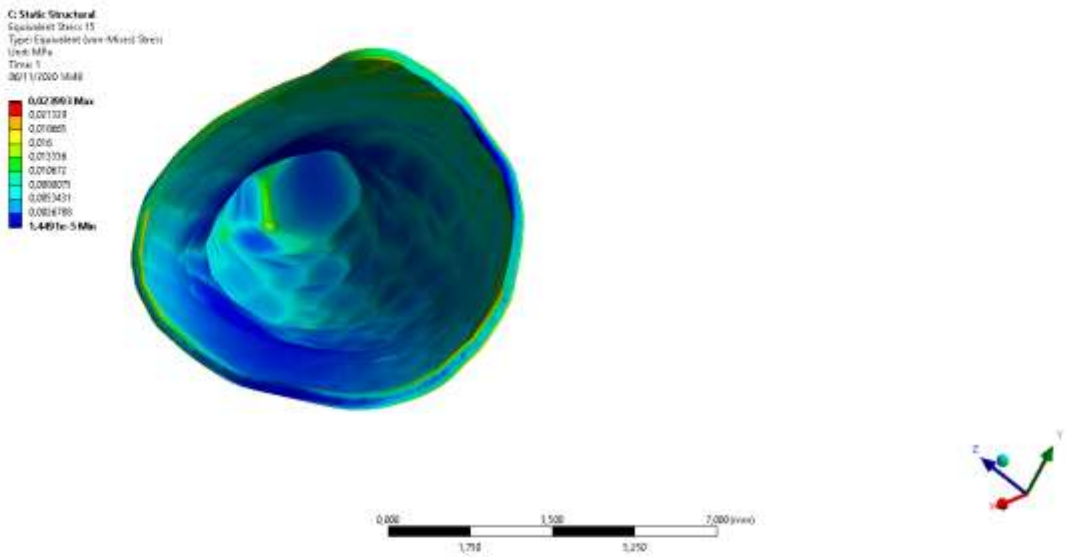

Maxilla without perforations

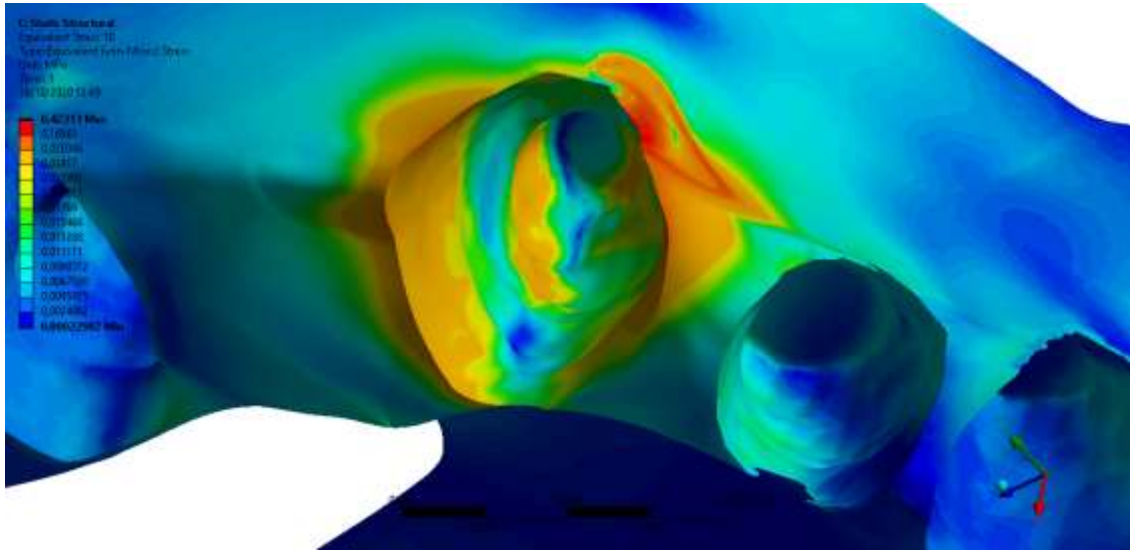

Maxilla without perforations with moment

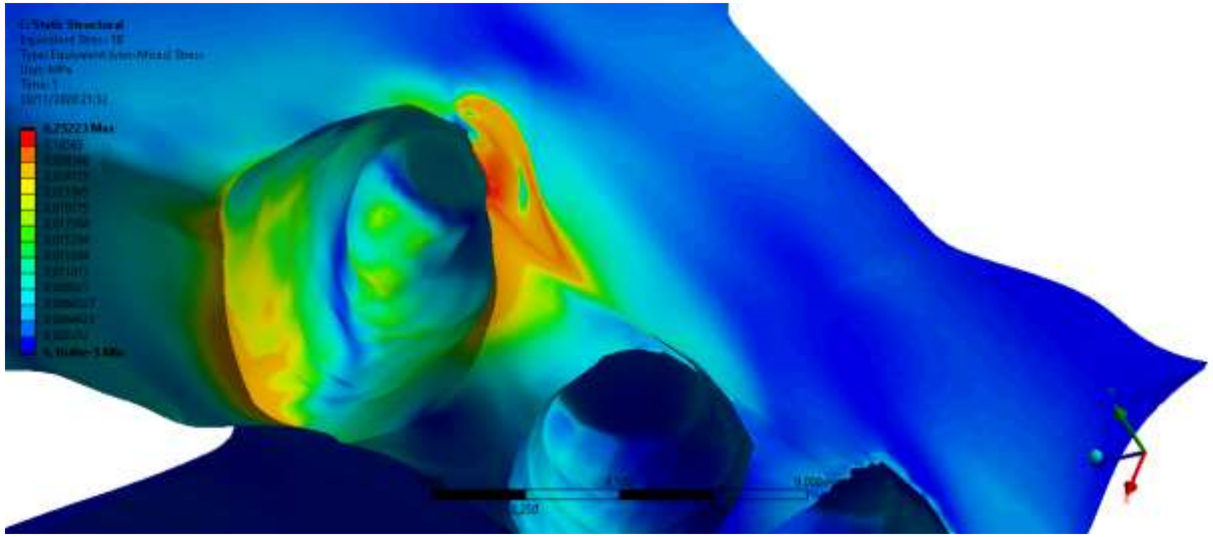

Maxilla with perforations with torque

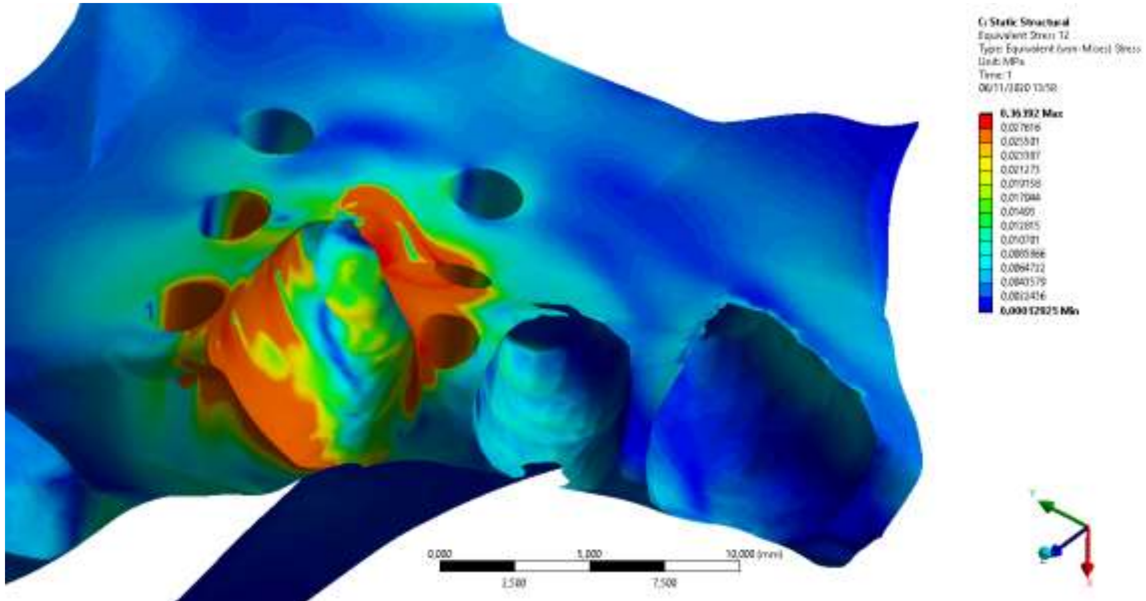

Maxilla without perforations

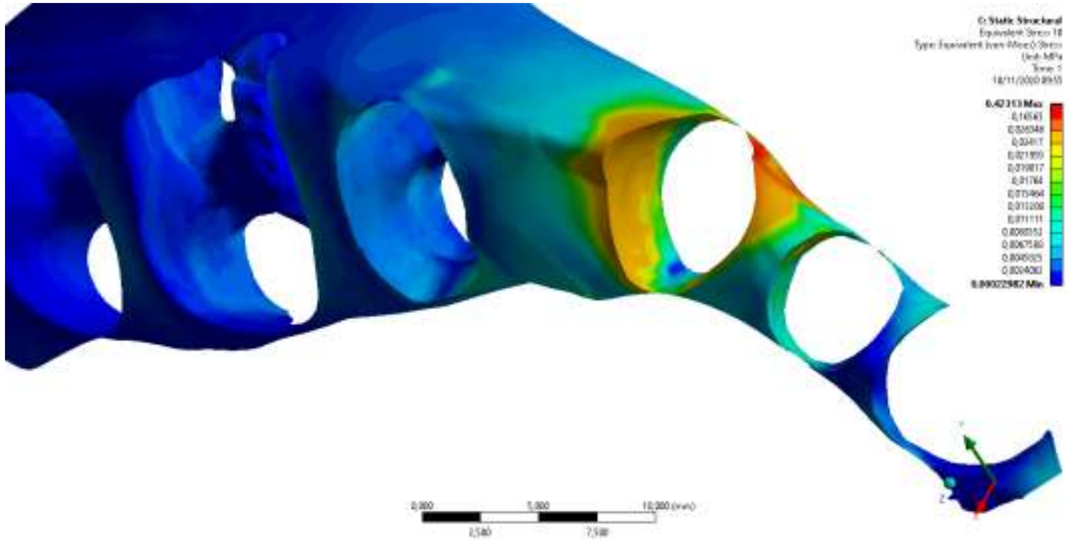

Maxilla without perforations with moment

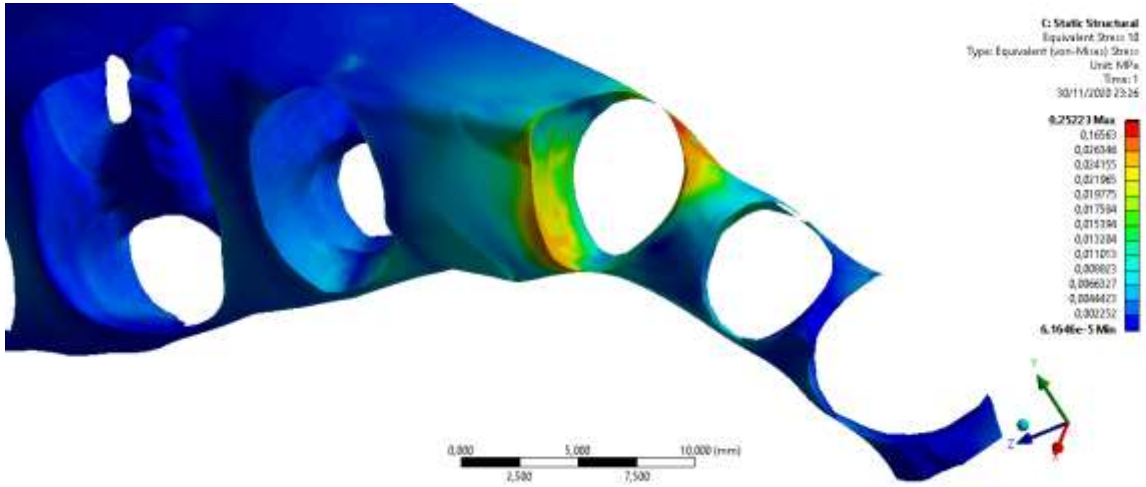

Maxilla without perforations

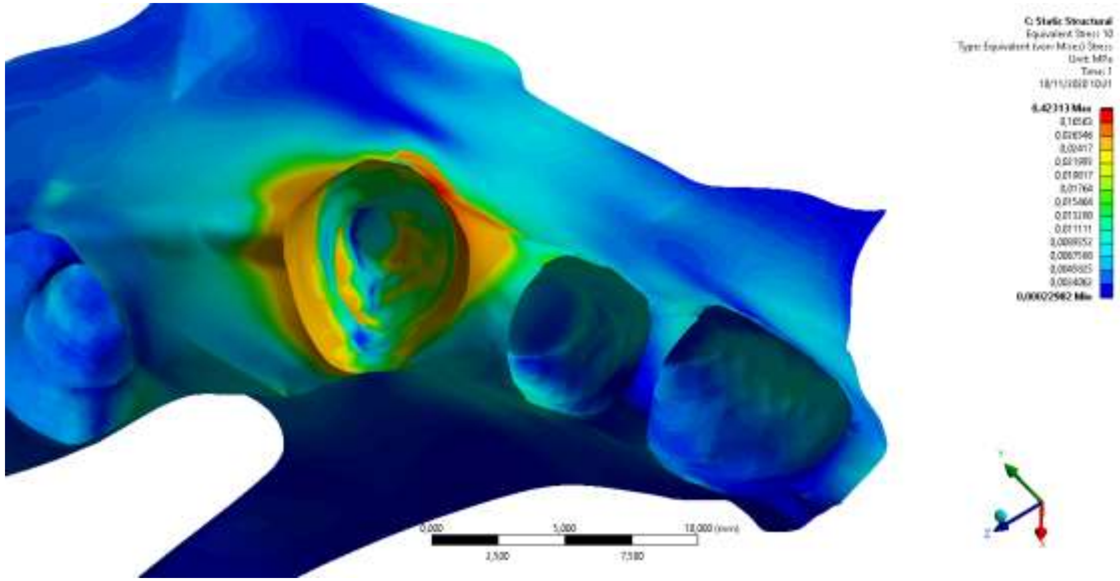

Maxilla with perforations

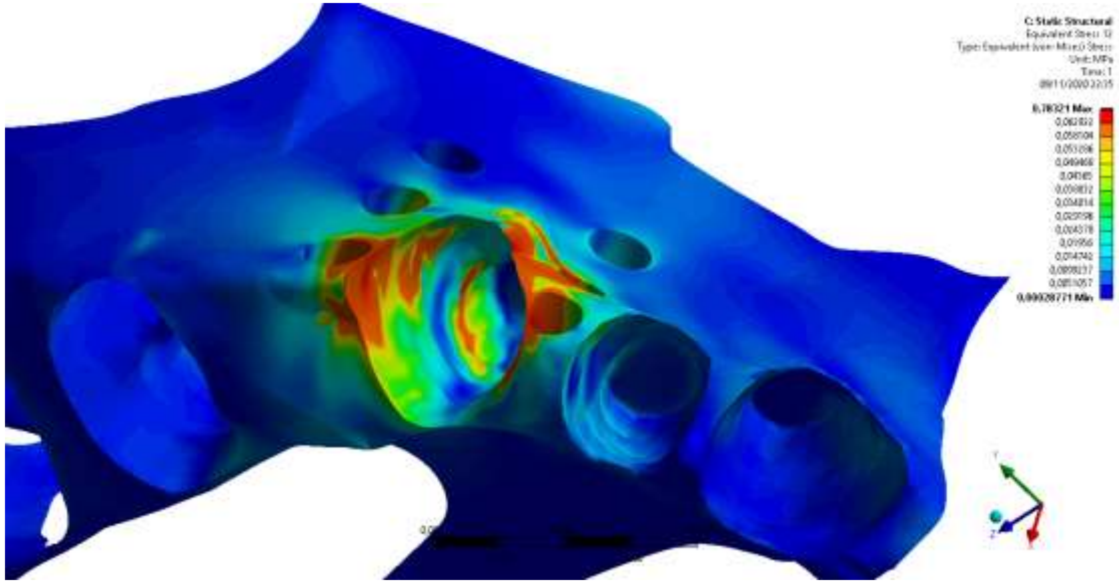

Maxilla without perforations with moment

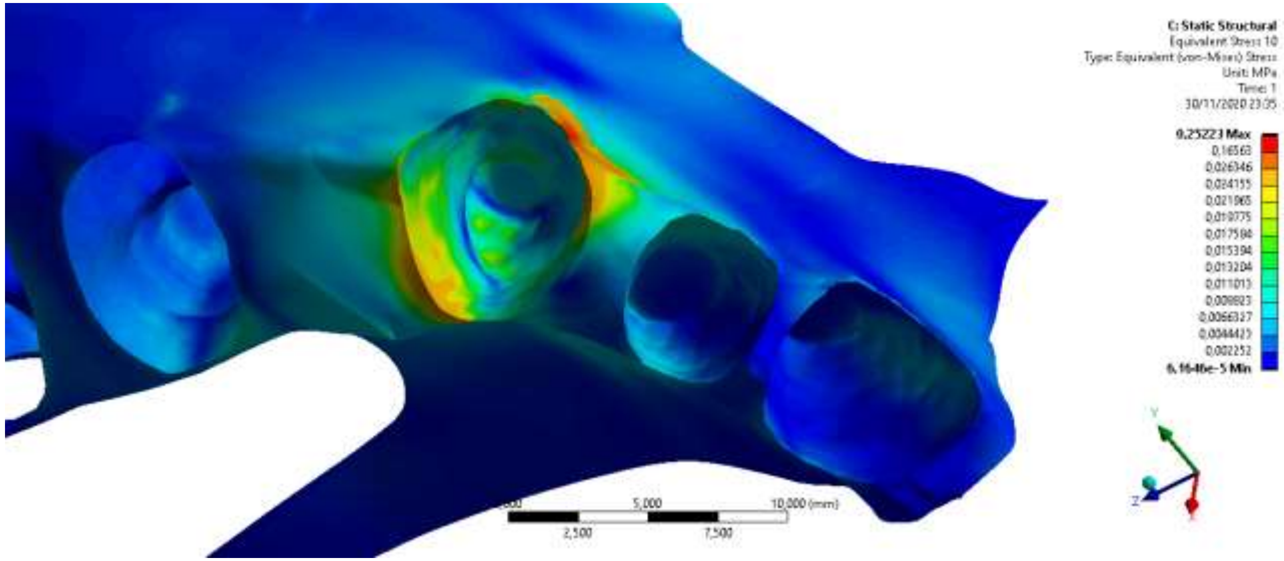

Maxilla with perforations with moment

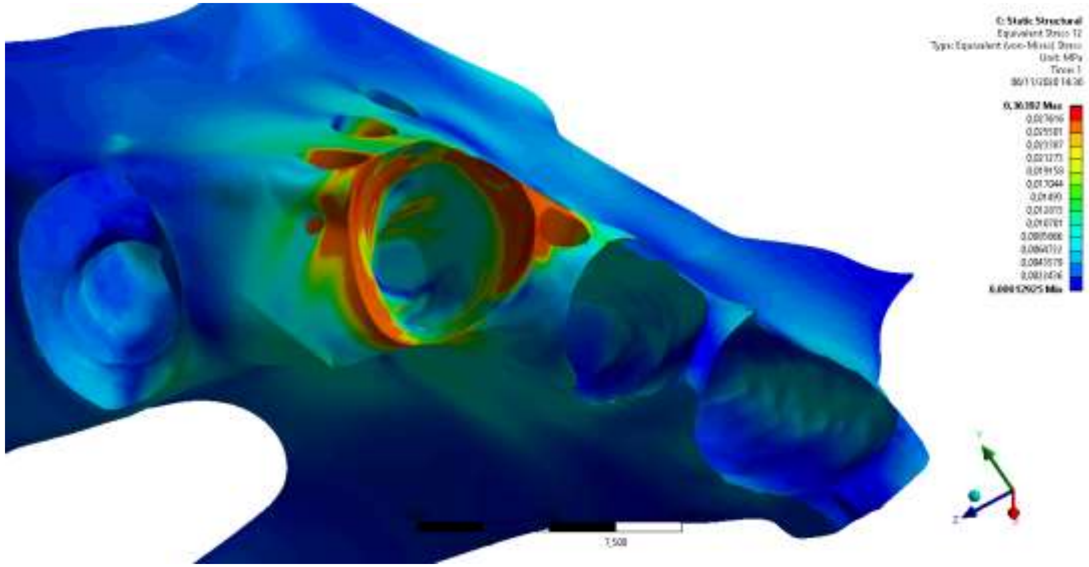

Maxilla with perforations

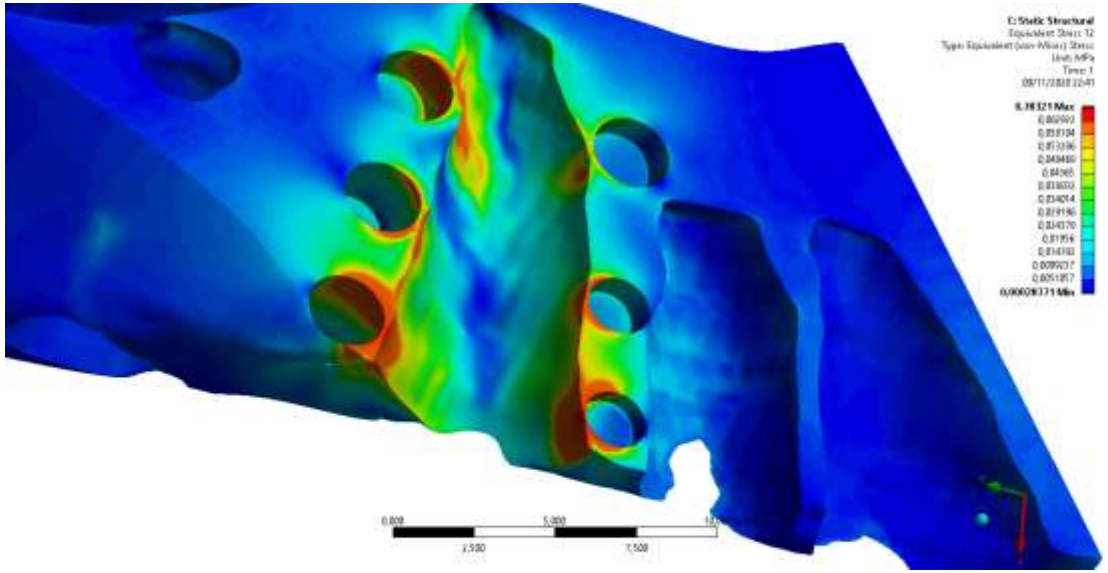

Maxilla with perforations with moment

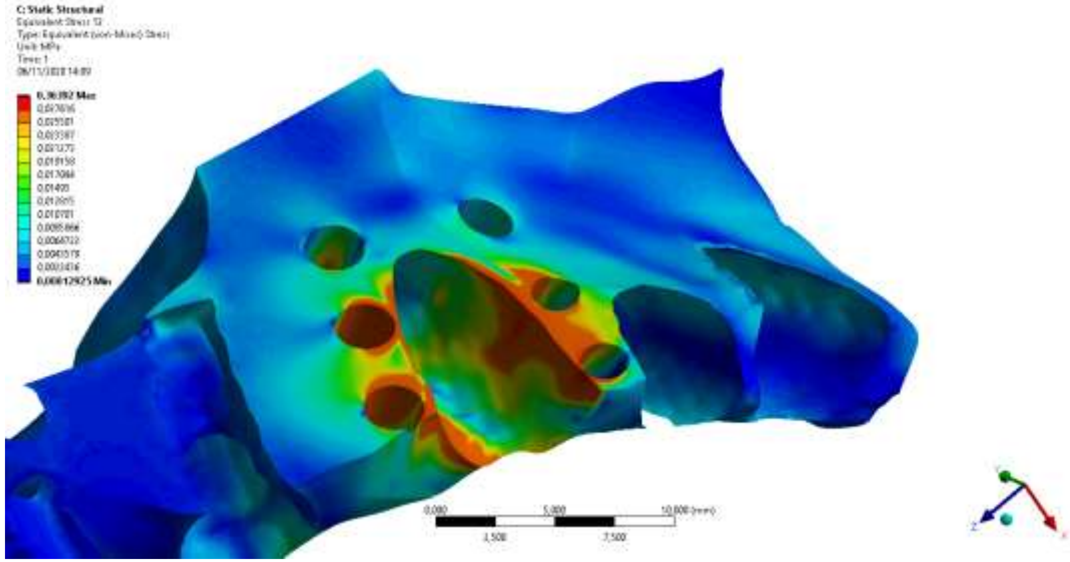

Supplement: S1 Fig — (PDF) [file pone.0308739.s009.pdf]
