# Supplementary material for: Micro-osteoperforation for enhancement of orthodontic movement: A mechanical analysis using the finite element method
Source: PLoS One. 2024 Aug 19;19(8):e0308739. doi: 10.1371/journal.pone.0308739 (PMC11332926; doi:10.1371/journal.pone.0308739)
Supplement: S3 Fig — (PDF) [file pone.0308739.s011.pdf]

S11. Comparison of images 3

Maxilla without perforations

Dente

Fatigue Data at zero mean stress comes from 1998 ASME BPV Code, Section 8, Div 2, Table 5-110.1

Density	1,96e-06 kg/mm³

Structural

Isotropic Elasticity

Derive from	Young's Modulus and Poisson's Ratio
Young's Modulus	14700 MPa
Poisson's Ratio	0,31000
Bulk Modulus	12895 MPa
Shear Modulus	5610,7 MPa
Isotropic Secant Coefficient of Thermal Expansion	1,2e-05 1/°C
Compressive Ultimate Strength	0 MPa
Compressive Yield Strength	250,00 MPa

Maxilla with perforations

Dente

Fatigue Data at zero mean stress comes from 1998 ASME BPV Code, Section 8, Div 2, Table 5-110.1

Density	1,96e-06 kg/mm³

Structural

Isotropic Elasticity

Derive from	Young's Modulus and Poisson's Ratio
Young's Modulus	14700 MPa
Poisson's Ratio	0,31000
Bulk Modulus	12895 MPa
Shear Modulus	5610,7 MPa
Isotropic Secant Coefficient of Thermal Expansion	1,2e-05 1/°C
Compressive Ultimate Strength	0 MPa
Compressive Yield Strength	250,00 MPa

Maxilla without perforations with moment

Dente

Fatigue Data at zero mean stress comes from 1998 ASME BPV Code, Section 8, Div 2, Table 5-110.1

Density	1,96e-06 kg/mm³

Structural

Isotropic Elasticity

Derive from	Young's Modulus and Poisson's Ratio
Young's Modulus	14700 MPa
Poisson's Ratio	0,31000
Bulk Modulus	12895 MPa
Shear Modulus	5610,7 MPa
Isotropic Secant Coefficient of Thermal Expansion	1,2e-05 1/°C
Compressive Ultimate Strength	0 MPa
Compressive Yield Strength	250,00 MPa

Maxilla with perforations with moment

Dente

Fatigue Data at zero mean stress comes from 1998 ASME BPV Code, Section 8, Div 2, Table 5-110.1

Density	1,96e-06 kg/mm³

Structural

Isotropic Elasticity

Derive from	Young's Modulus and Poisson's Ratio
Young's Modulus	14700 MPa
Poisson's Ratio	0,31000
Bulk Modulus	12895 MPa
Shear Modulus	5610,7 MPa
Isotropic Secant Coefficient of Thermal Expansion	1,2e-05 1/°C
Compressive Ultimate Strength	0 MPa
Compressive Yield Strength	250,00 MPa

Maxilla without perforations

LigamentoPeriodotal

Fatigue Data at zero mean stress comes from 1998 ASME BPV Code, Section 8, Div 2, Table 5-110.1

Density	1,2e-06 kg/mm³

Structural

Isotropic Elasticity

Derive from	Young's Modulus and Poisson's Ratio
Young's Modulus	0,068000 MPa
Poisson's Ratio	0,45000
Bulk Modulus	0,22667 MPa
Shear Modulus	0,023448 MPa

Maxilla with perforations

LigamentoPeriodotal

Fatigue Data at zero mean stress comes from 1998 ASME BPV Code, Section 8, Div 2, Table 5-110.1

Density	1,2e-06 kg/mm³

Structural

Isotropic Elasticity

Derive from	Young's Modulus and Poisson's Ratio
Young's Modulus	0,068000 MPa
Poisson's Ratio	0,45000
Bulk Modulus	0,22667 MPa
Shear Modulus	0,023448 MPa

Maxilla without perforations with moment

LigamentoPeriodotal

Fatigue Data at zero mean stress comes from 1998 ASME BPV Code, Section 8, Div 2, Table 5-110.1

Density	1,2e-06 kg/mm³

Structural

Isotropic Elasticity

Derive from	Young's Modulus and Poisson's Ratio
Young's Modulus	0,068000 MPa
Poisson's Ratio	0,45000
Bulk Modulus	0,22667 MPa
Shear Modulus	0,023448 MPa

Maxilla with perforations with moment

LigamentoPeriodotal

Fatigue Data at zero mean stress comes from 1998 ASME BPV Code, Section 8, Div 2, Table 5-110.1

Density	1,2e-06 kg/mm³

Structural

Isotropic Elasticity

Derive from	Young's Modulus and Poisson's Ratio
Young's Modulus	0,068000 MPa
Poisson's Ratio	0,45000
Bulk Modulus	0,22667 MPa
Shear Modulus	0,023448 MPa

Maxilla without perforations

Osso Medular

Density	4,1e-07 kg/mm³

Structural

Isotropic Elasticity

Derive from	Young's Modulus and Poisson's Ratio
Young's Modulus	1370,0 MPa
Poisson's Ratio	0,30000
Bulk Modulus	1141,7 MPa
Shear Modulus	526,92 MPa

Maxilla with perforations

Osso Medular

Density	4,1e-07 kg/mm³

Structural

Isotropic Elasticity

Derive from	Young's Modulus and Poisson's Ratio
Young's Modulus	1370,0 MPa
Poisson's Ratio	0,30000
Bulk Modulus	1141,7 MPa
Shear Modulus	526,92 MPa

Maxilla without perforations with moment

Osso Medular

Density	4,1e-07 kg/mm³

Structural

Isotropic Elasticity

Derive from	Young's Modulus and Poisson's Ratio
Young's Modulus	1370,0 MPa
Poisson's Ratio	0,30000
Bulk Modulus	1141,7 MPa
Shear Modulus	526,92 MPa

Maxilla with perforations with moment

Osso Medular

Density	4,1e-07 kg/mm³

Structural

Isotropic Elasticity

Derive from	Young's Modulus and Poisson's Ratio
Young's Modulus	1370,0 MPa
Poisson's Ratio	0,30000
Bulk Modulus	1141,7 MPa
Shear Modulus	526,92 MPa

Maxilla without perforations

Osso Cortical Isotropico

Density	1,99e-06 kg/mm³

Structural

Isotropic Elasticity

Derive from	Young's Modulus and Poisson's Ratio
Young's Modulus	13700 MPa
Poisson's Ratio	0,30000
Bulk Modulus	11417 MPa
Shear Modulus	5269,2 MPa

Maxilla with perforations

Osso Cortical Isotropico

Density	1,99e-06 kg/mm³

Structural

Isotropic Elasticity

Derive from	Young's Modulus and Poisson's Ratio
Young's Modulus	13700 MPa
Poisson's Ratio	0,30000
Bulk Modulus	11417 MPa
Shear Modulus	5269,2 MPa

Maxilla without perforations with moment

Osso Cortical Isotropico

Density	1,99e-06 kg/mm³

Structural

Isotropic Elasticity

Derive from	Young's Modulus and Poisson's Ratio
Young's Modulus	13700 MPa
Poisson's Ratio	0,30000
Bulk Modulus	11417 MPa
Shear Modulus	5269,2 MPa

Maxilla with perforations with moment

Osso Cortical Isotropico

Density	1,99e-06 kg/mm³

Structural

Isotropic Elasticity

Derive from	Young's Modulus and Poisson's Ratio
Young's Modulus	13700 MPa
Poisson's Ratio	0,30000
Bulk Modulus	11417 MPa
Shear Modulus	5269,2 MPa

Maxilla with perforations

Maxilla with perforations with moment

Tecido Granulomatoso

Density	4,1e-07 kg/mm³

Structural

Isotropic Elasticity

Derive from	Young's Modulus and Poisson's Ratio
Young's Modulus	1,0000 MPa
Poisson's Ratio	0,49000
Bulk Modulus	16,667 MPa
Shear Modulus	0,33557 MPa

Maxilla without perforations

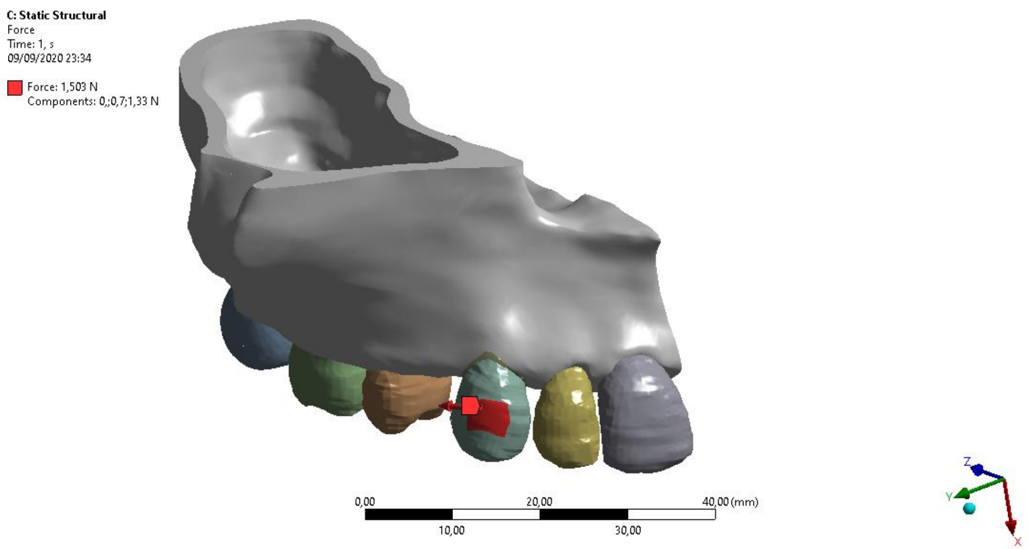

Maxilla with perforations

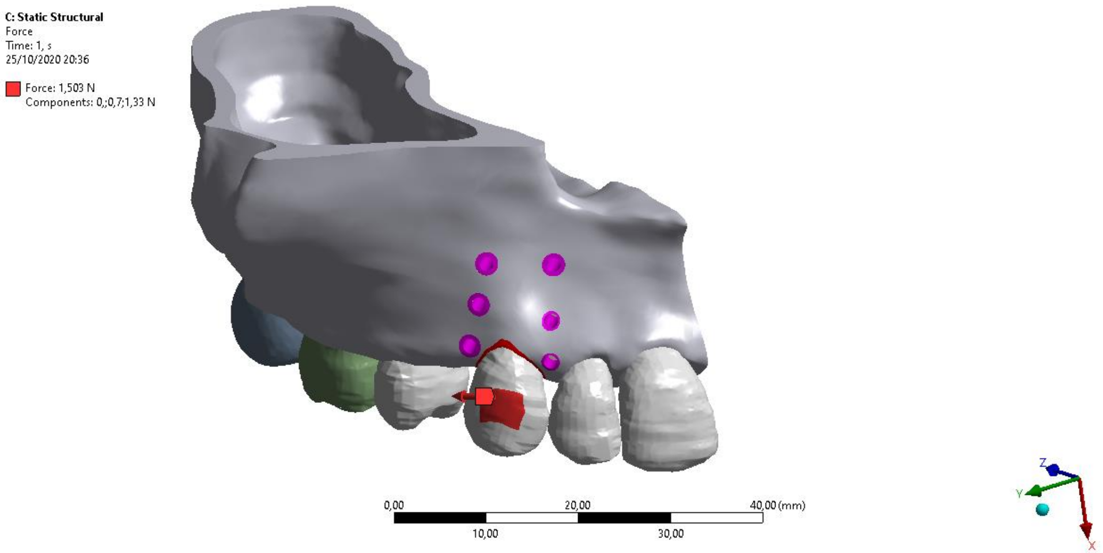

Maxilla without perforations with moment

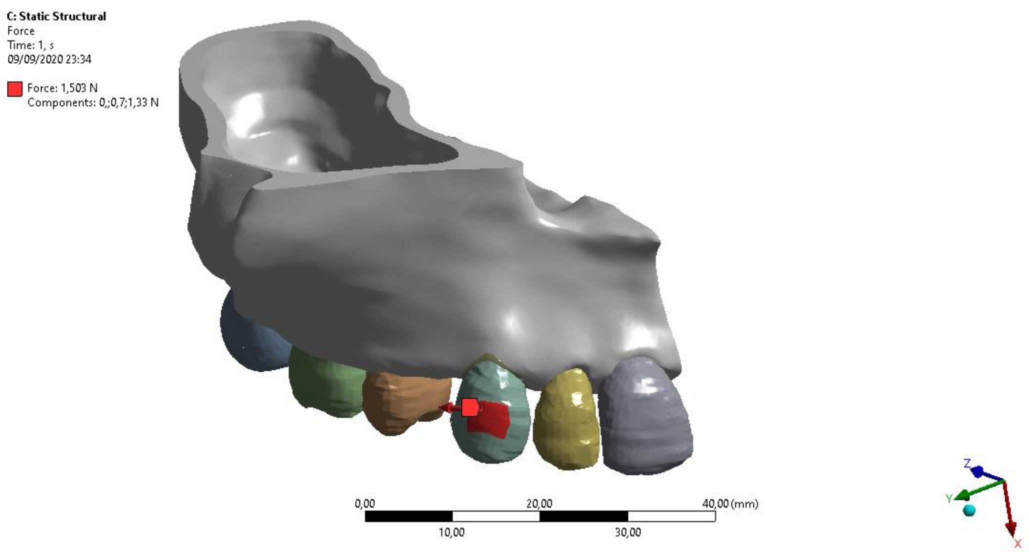

Maxilla with perforations with moment

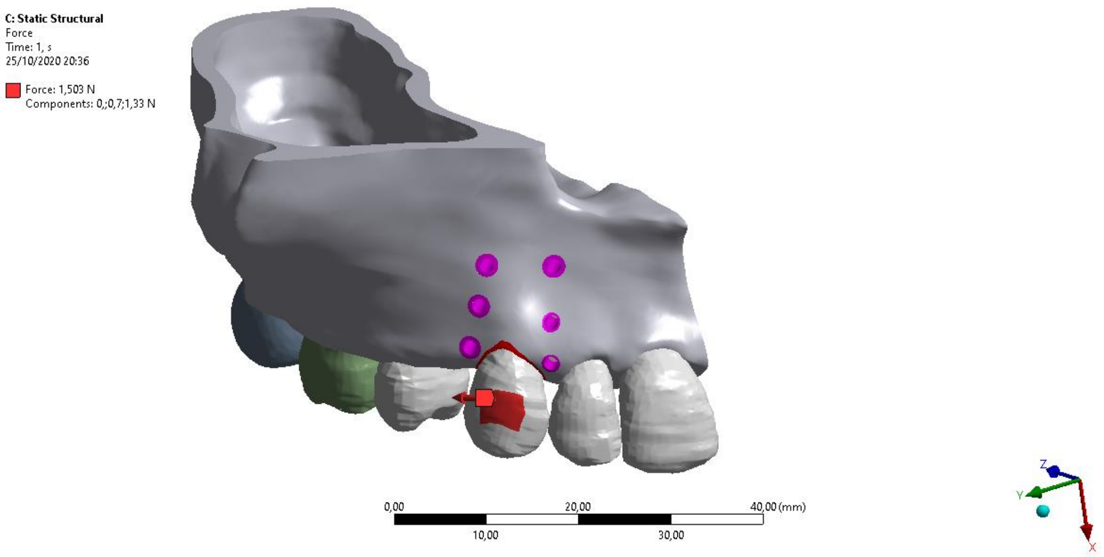

Maxilla with perforations with moment

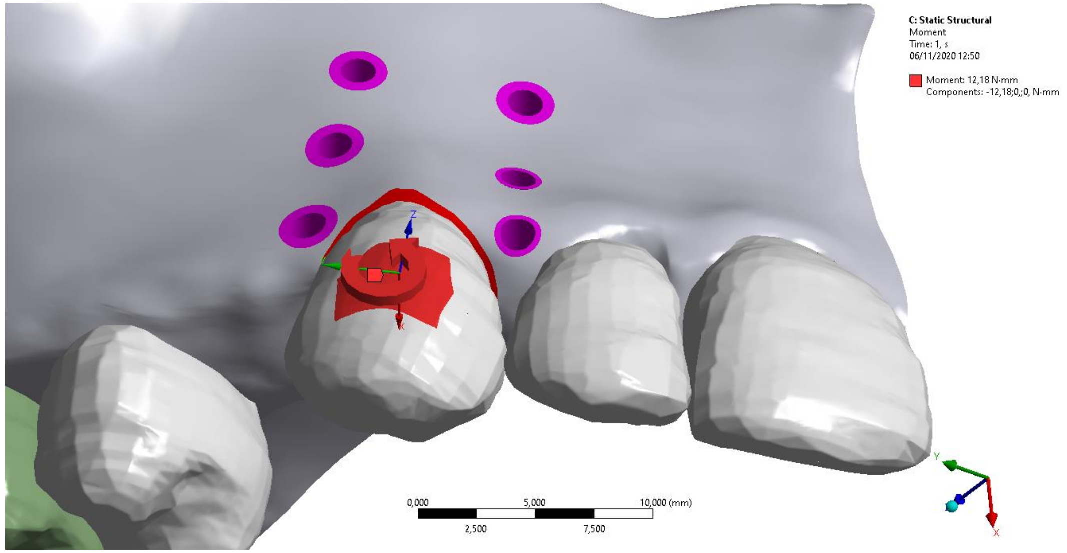

Maxilla with perforations

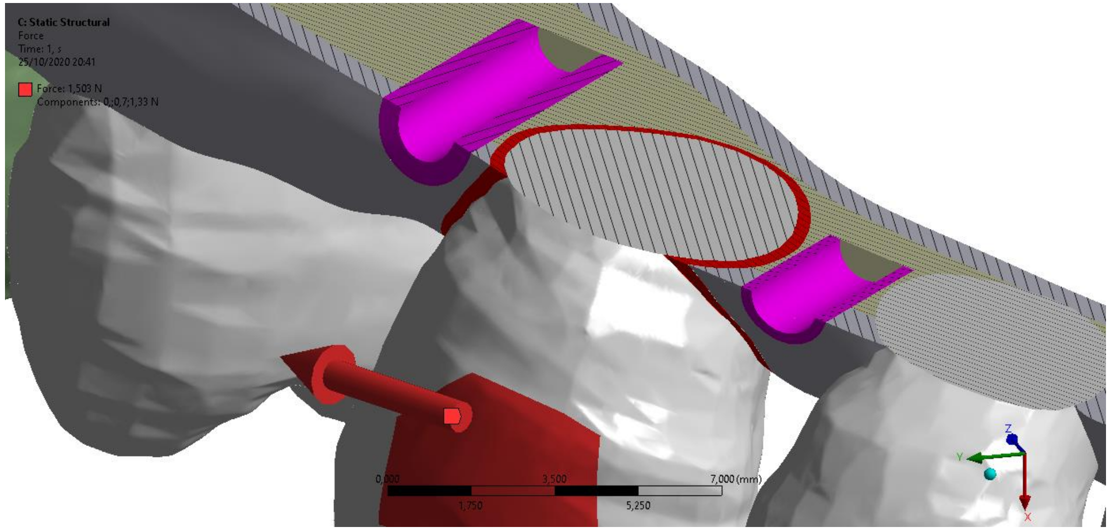

Maxilla with perforations with moment

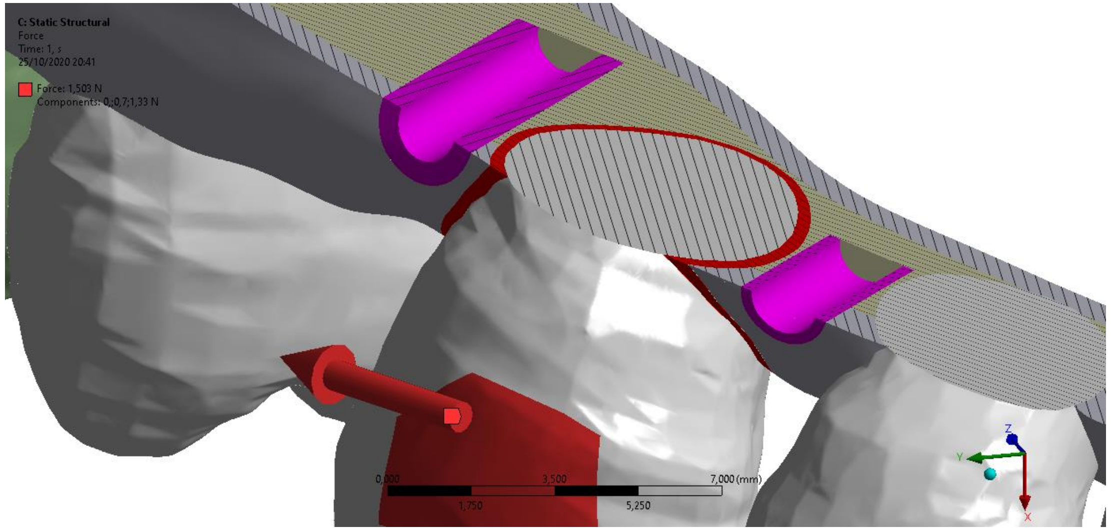

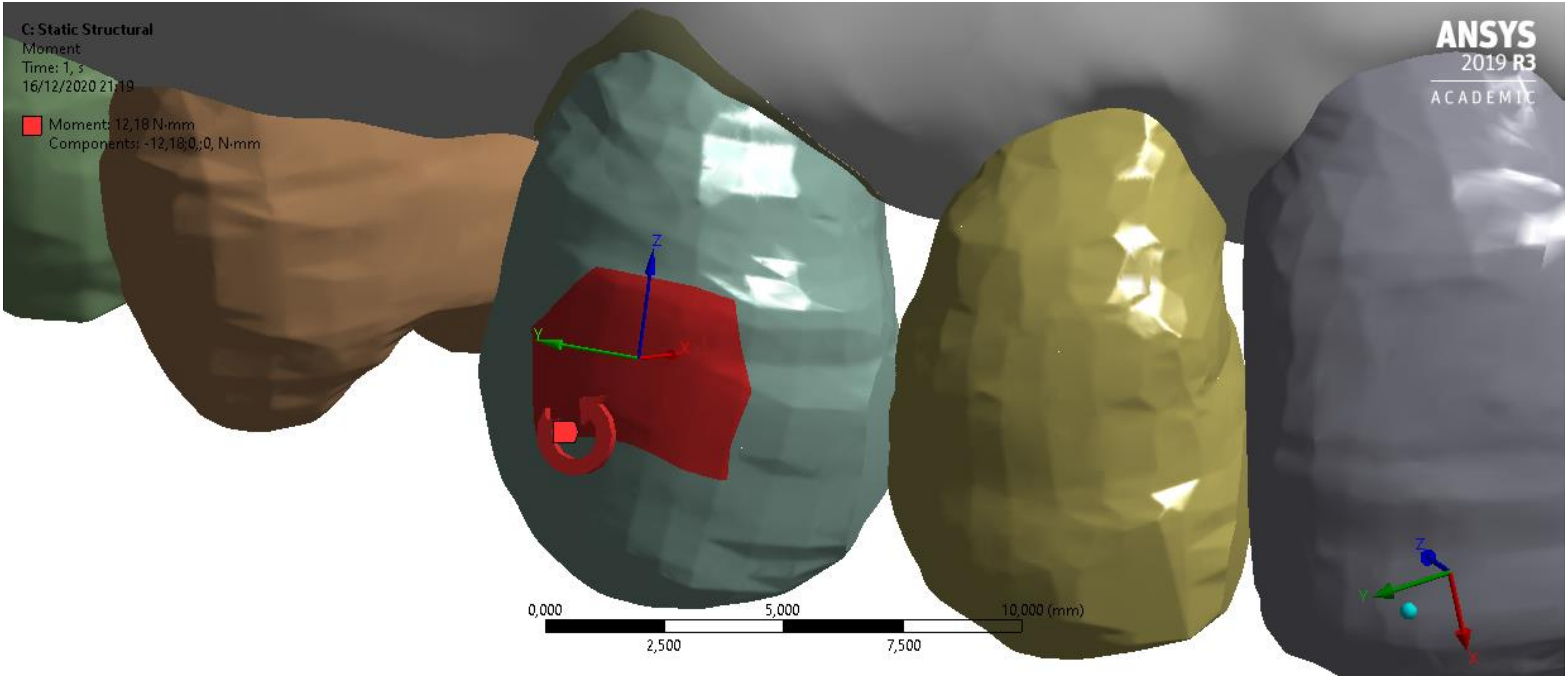
C: Static Structural
Moment
Time: 1, s
16/12/2020 21:19
Moment: 12,18 N·mm
Components: -12,18;0,;0, N·mm
ANSYS
2019 R3
ACADEMIC
0,000
5,000
10,000 (mm)
2,500
7,500

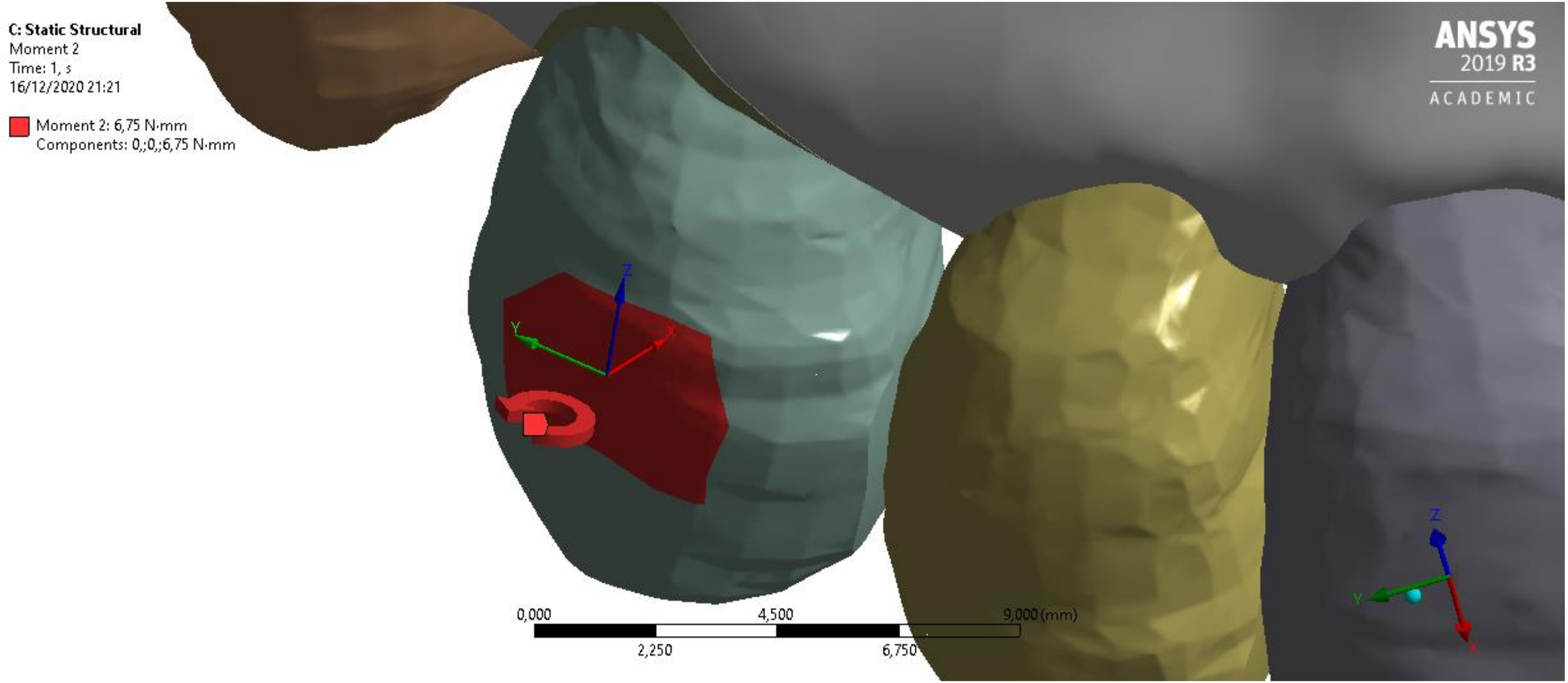
C: Static Structural
Moment 2
Time: 1, s
16/12/2020 21:21
Moment 2: 6,75 N·mm
Components: 0,;0,;6,75 N·mm
ANSYS
2019 R3
ACADEMIC
0,000
4,500
9,000 (mm)
2,250
6,750

1-

Maxilla without perforations

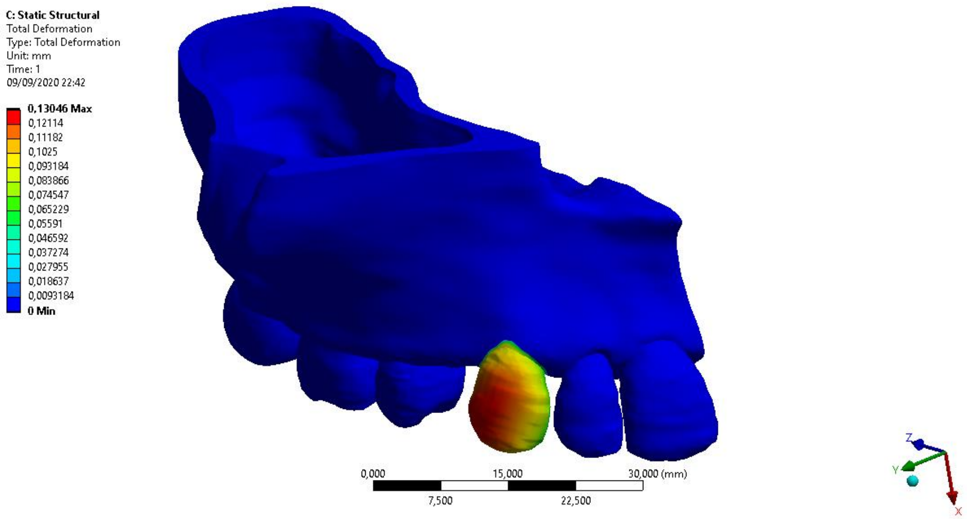

Maxilla with perforations

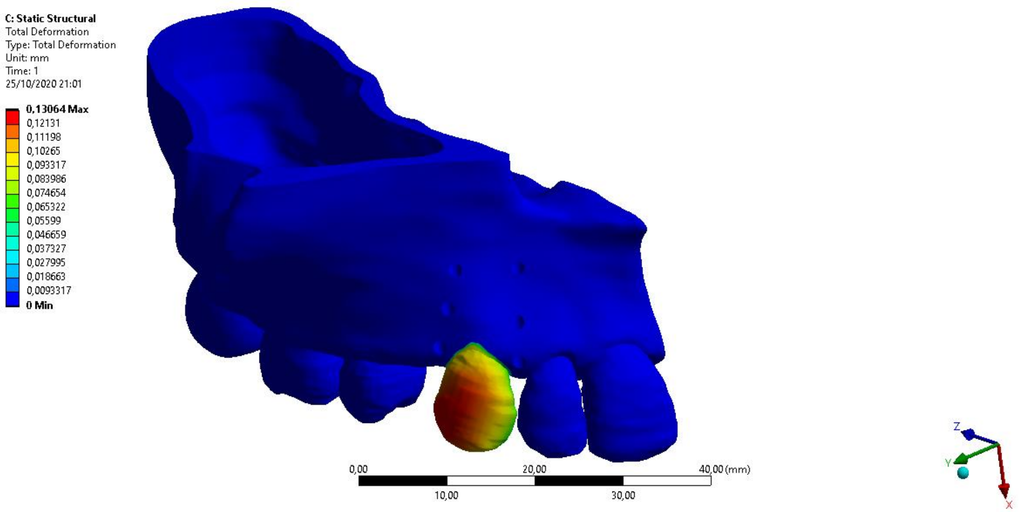

Maxilla without perforations with moment

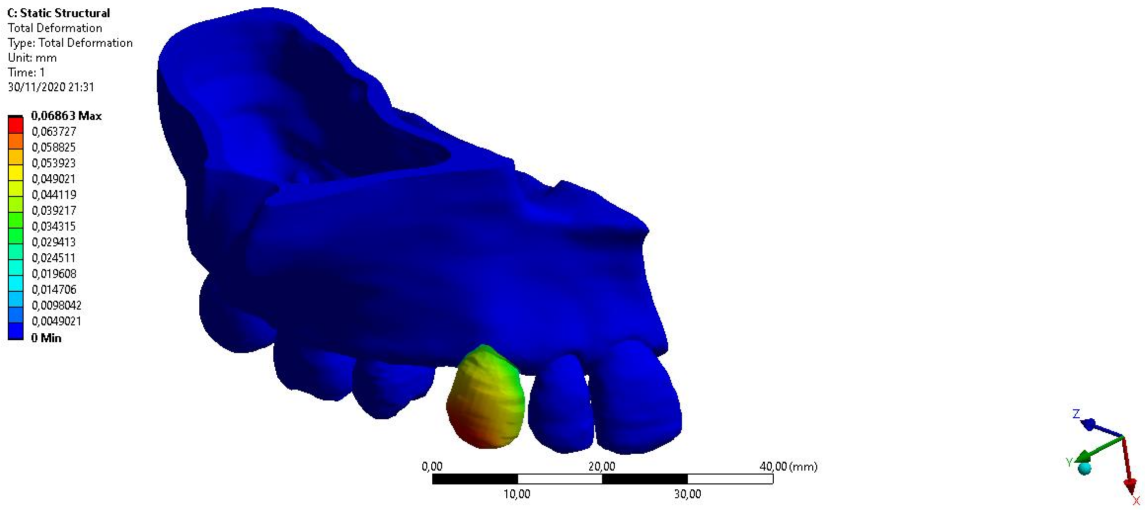

Maxilla with perforations with moment

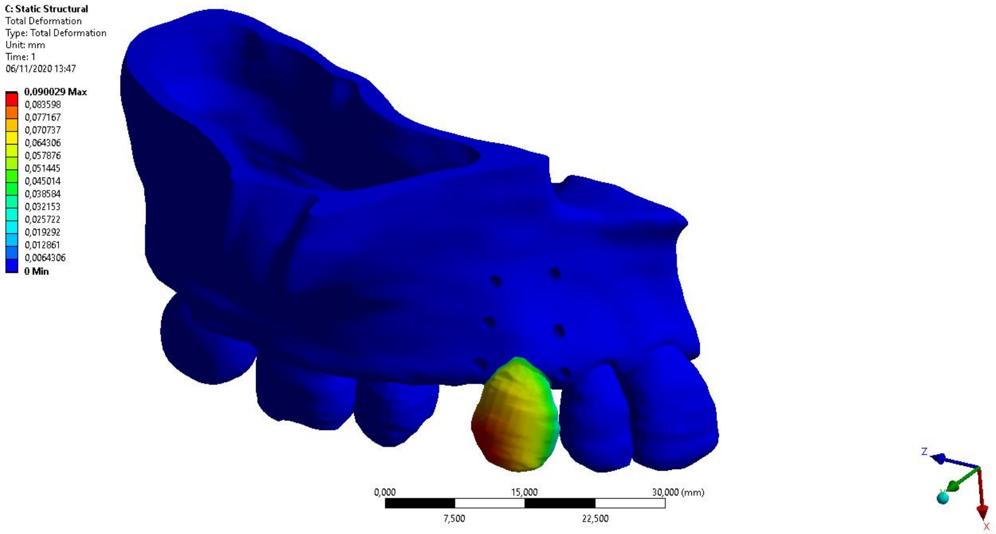

2-

Maxilla without perforations

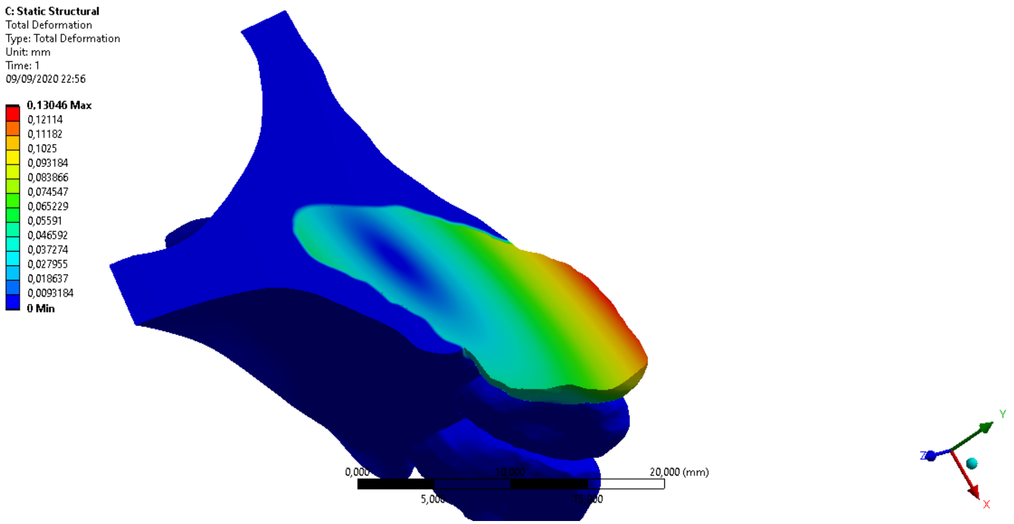

Maxilla with perforations

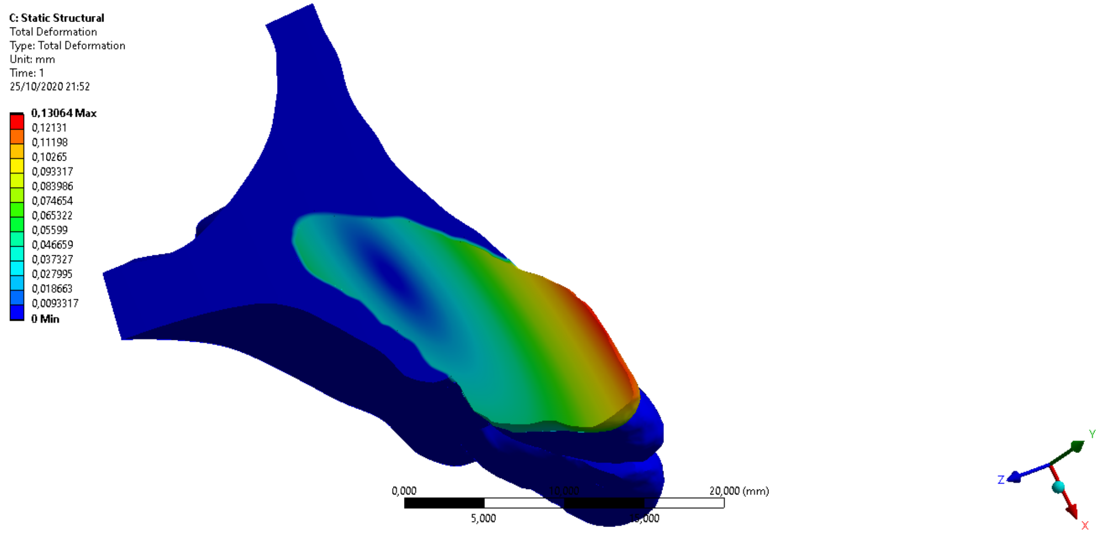

Maxilla without perforations with moment

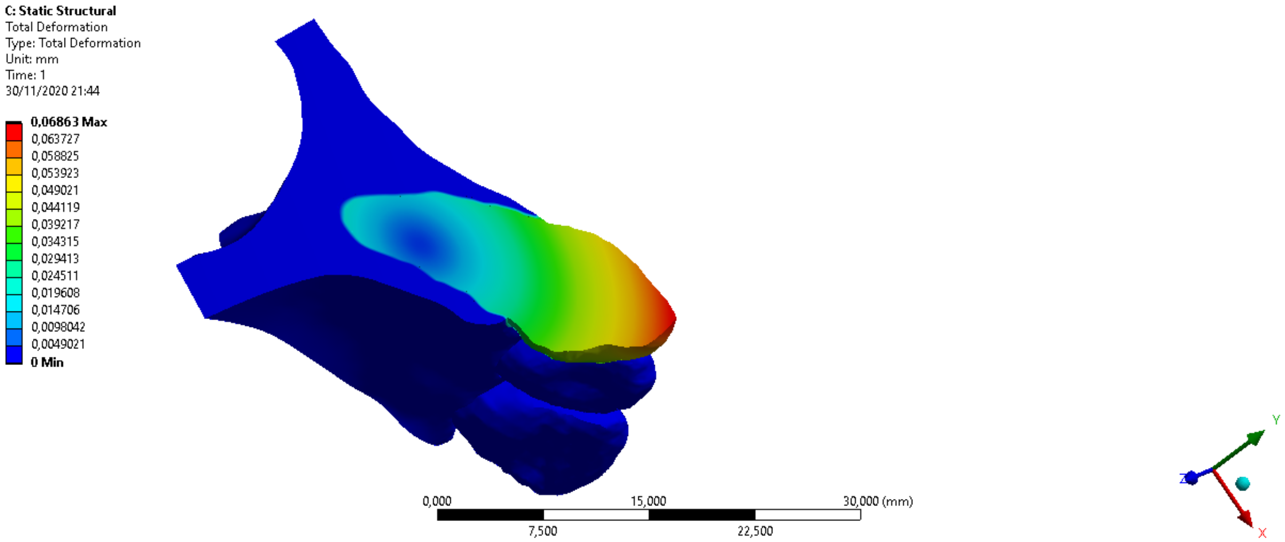

Maxilla with perforations with moment

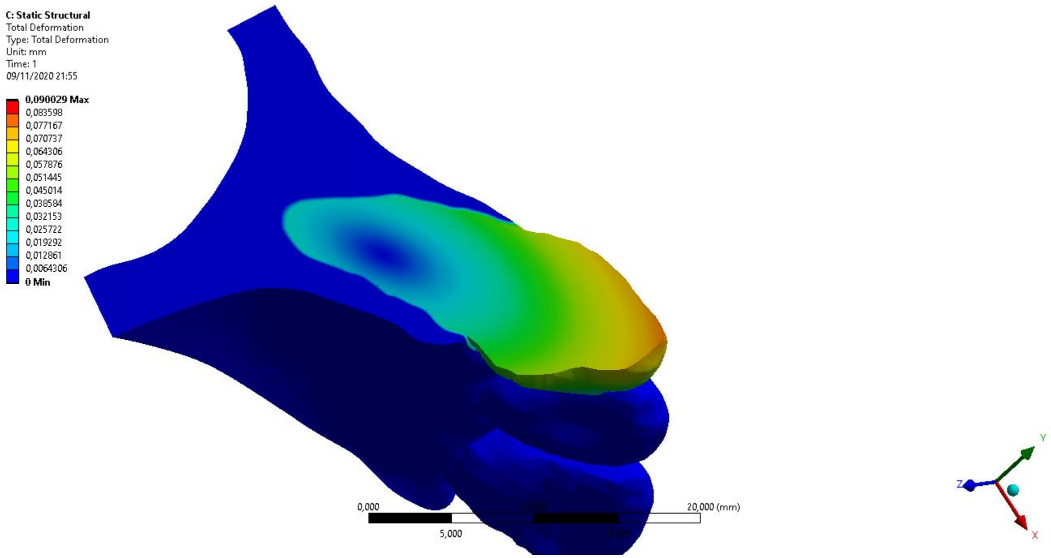

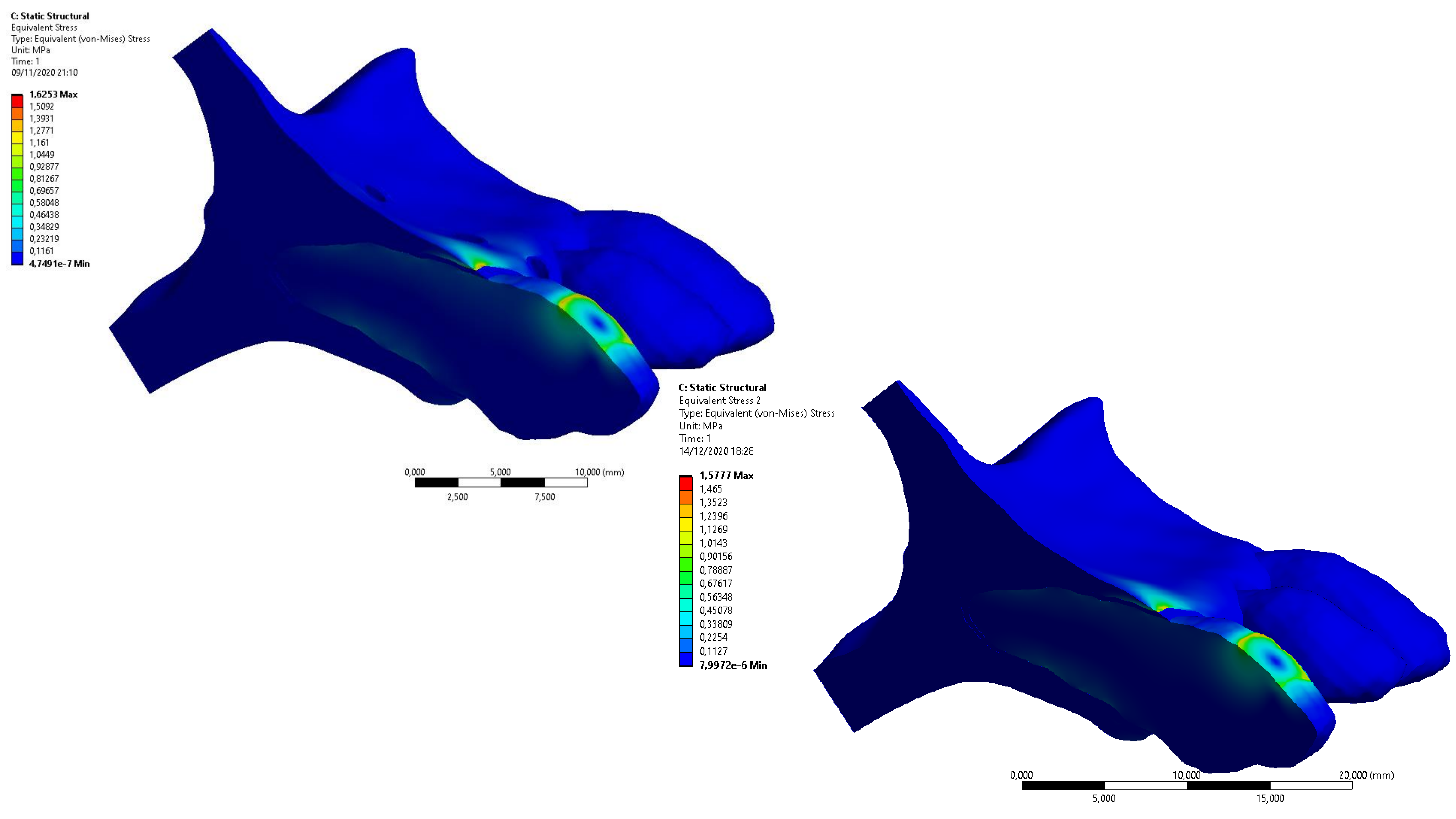
C: Static Structural
Equivalent Stress
Type: Equivalent (von-Mises) Stress
Unit: MPa
Time: 1
09/11/2020 21:10
1,6253 Max
1,5092
1,3931
1,2771
1,161
1,0449
0,92877
0,81267
0,69657
0,58048
0,46438
0,34829
0,23219
0,1161
4,7491e-7 Min
0,000
5,000
10,000 (mm)
2,500
7,500
C: Static Structural
Equivalent Stress 2
Type: Equivalent (von-Mises) Stress
Unit: MPa
Time: 1
14/12/2020 18:28
1,5777 Max
1,465
1,3523
1,2396
1,1269
1,0143
0,90156
0,78887
0,67617
0,56348
0,45078
0,33809
0,2254
0,1127
7,9972e-6 Min
0,000
10,000
20,000 (mm)
5,000
15,000

Maxilla without perforations

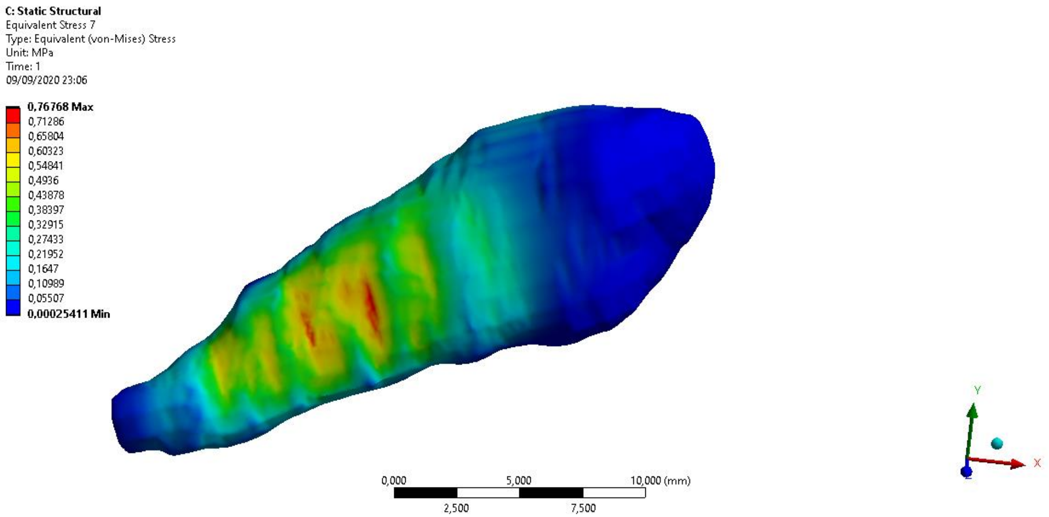

Maxilla with perforations

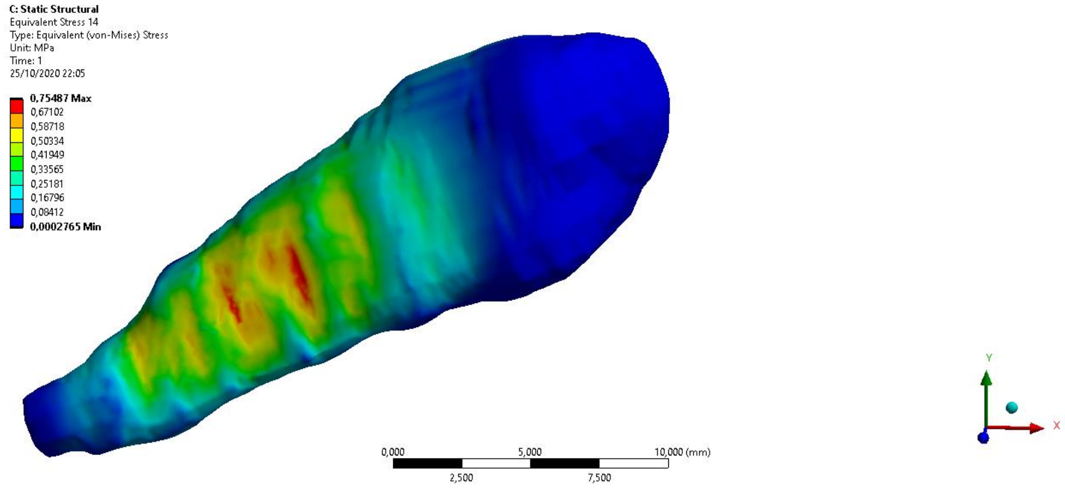

Maxilla without perforations with moment

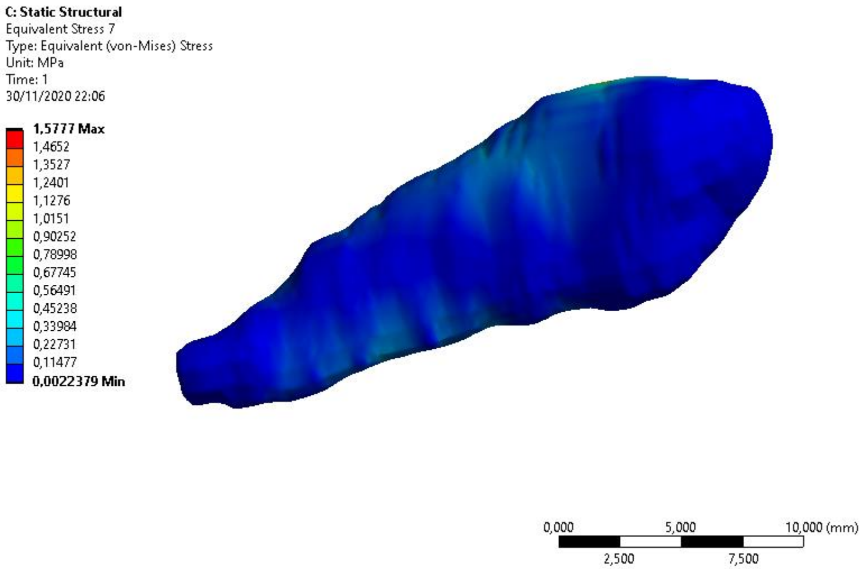

Maxilla with perforations with moment

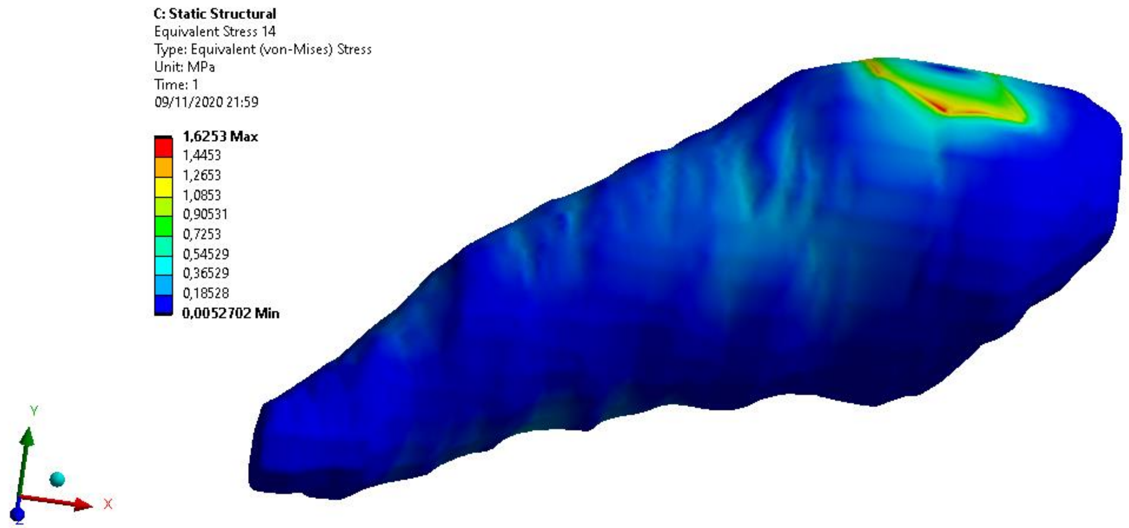

Maxilla without perforations with moment

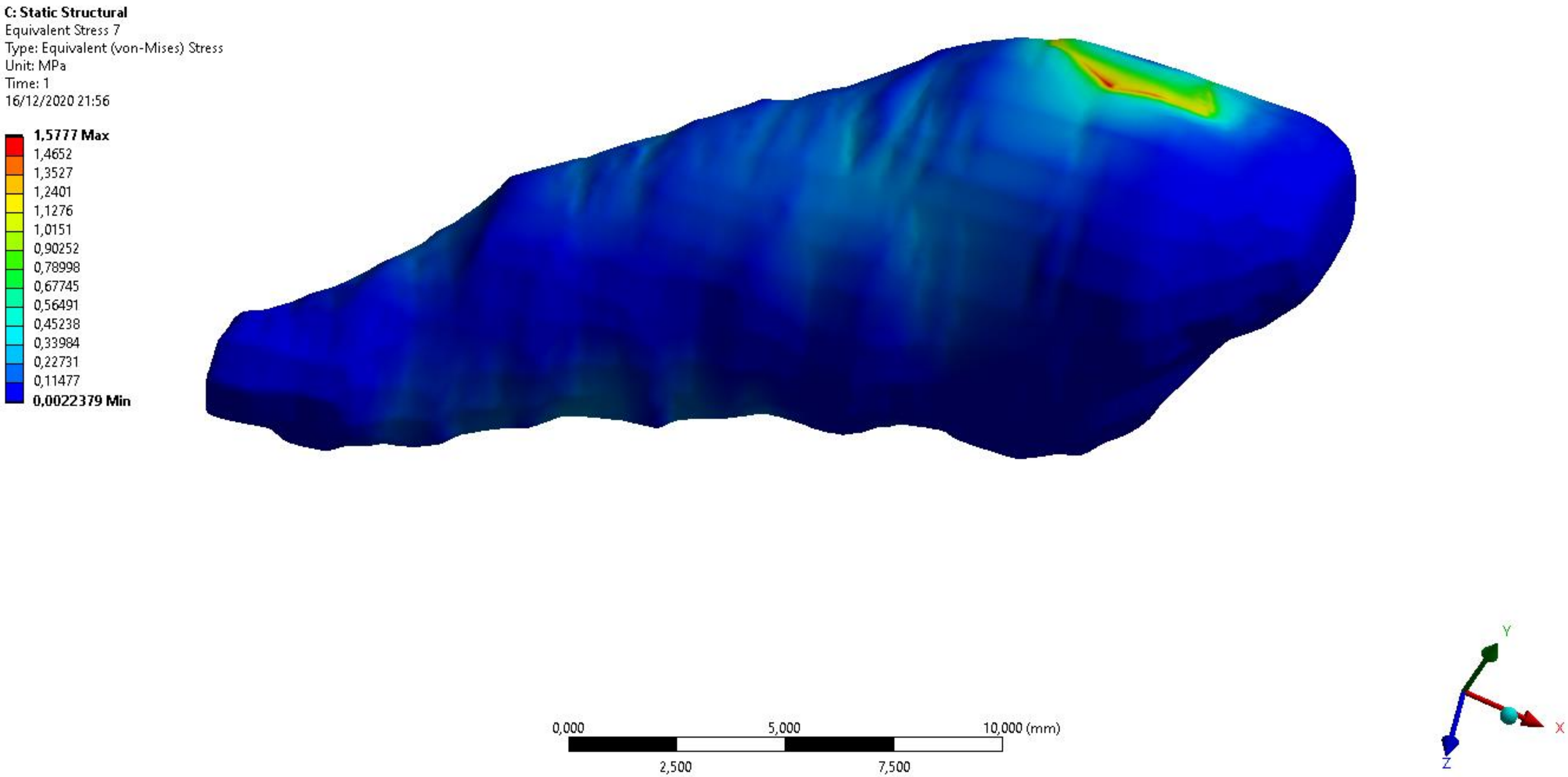

Maxilla with perforations with moment

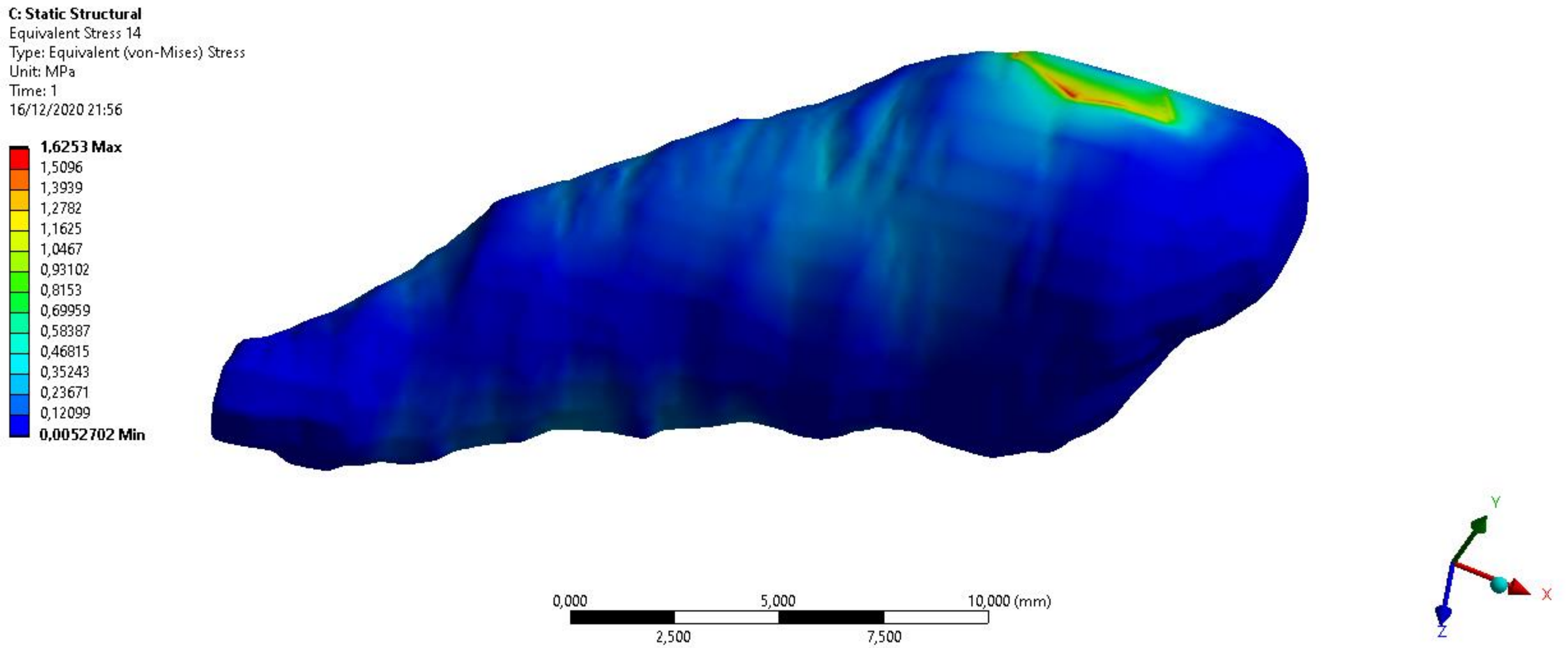

Maxilla without perforations with moment

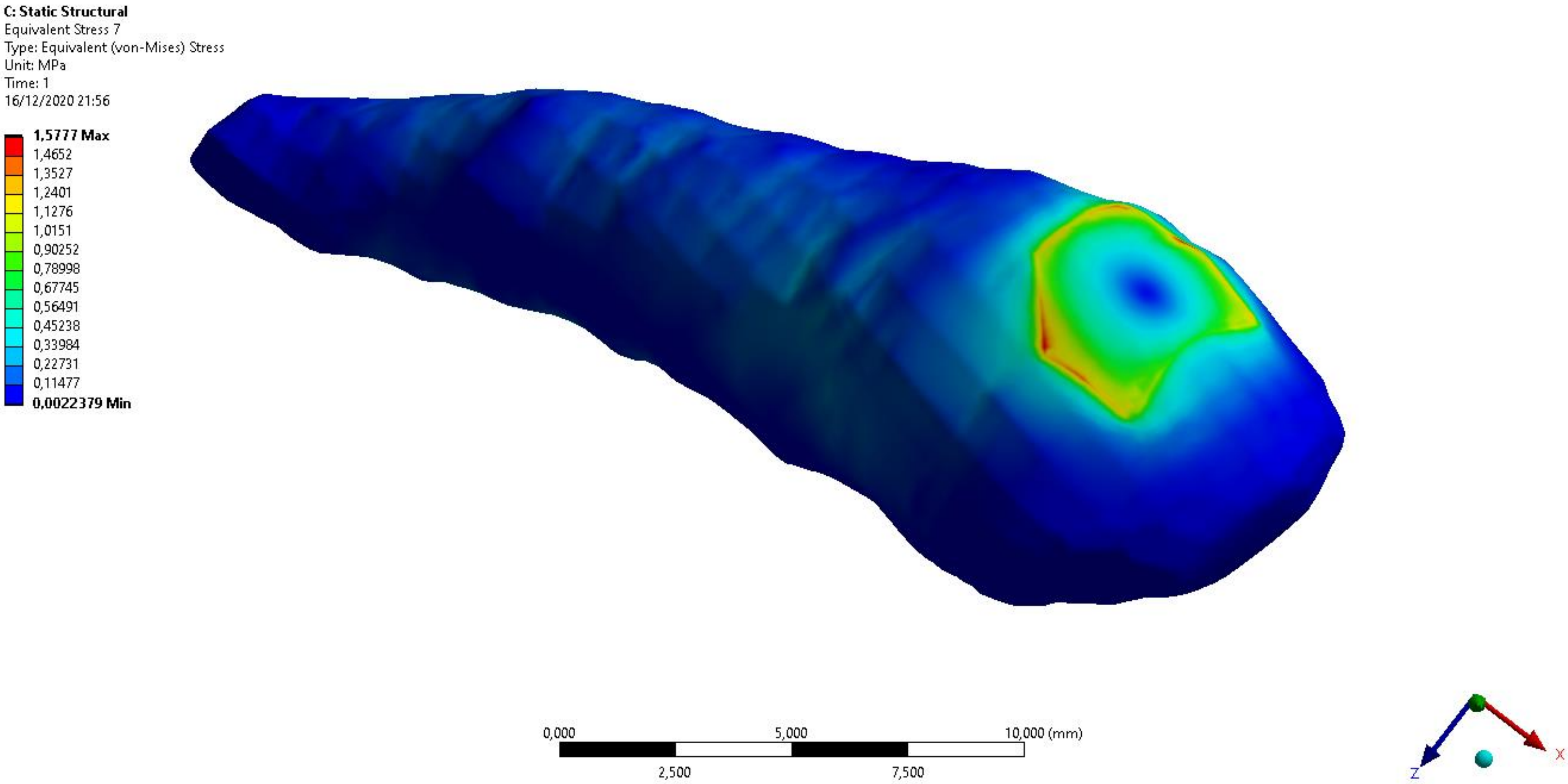

Maxilla with perforations with moment

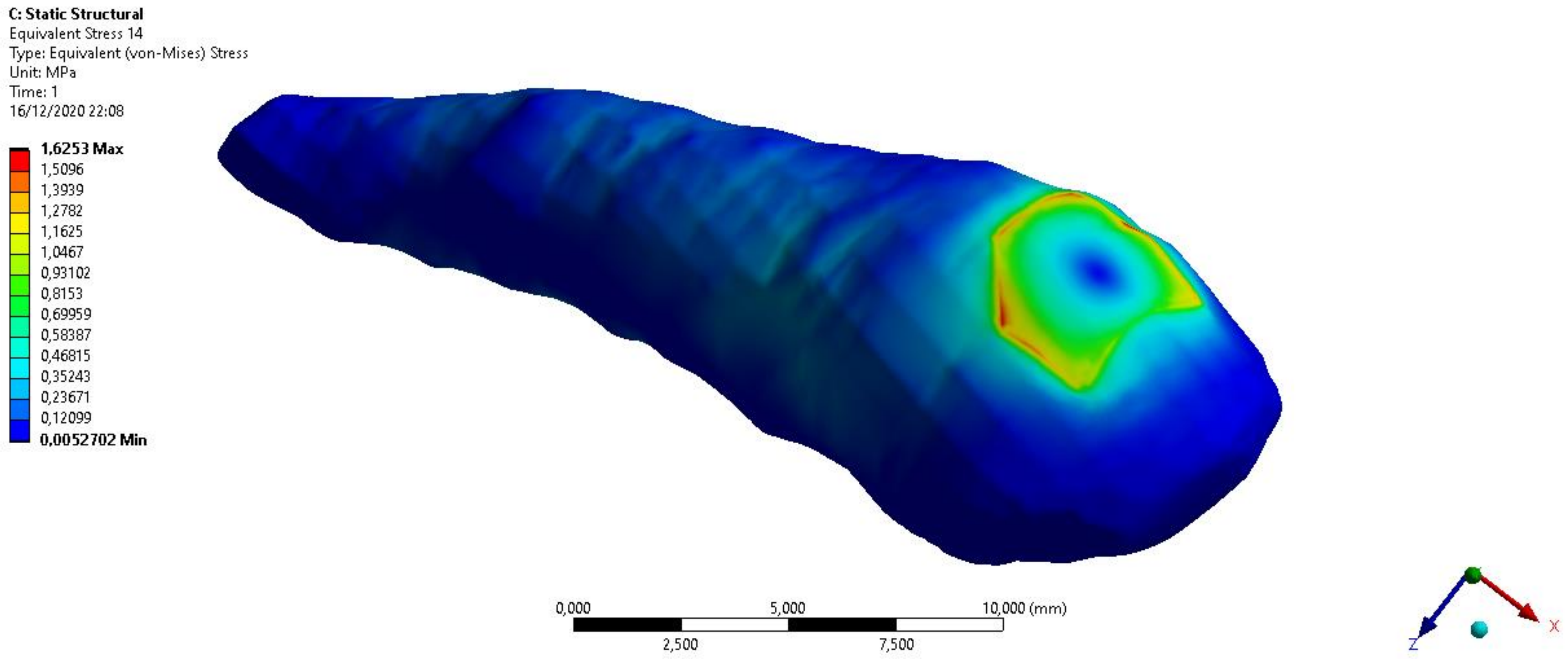

Maxilla without perforations

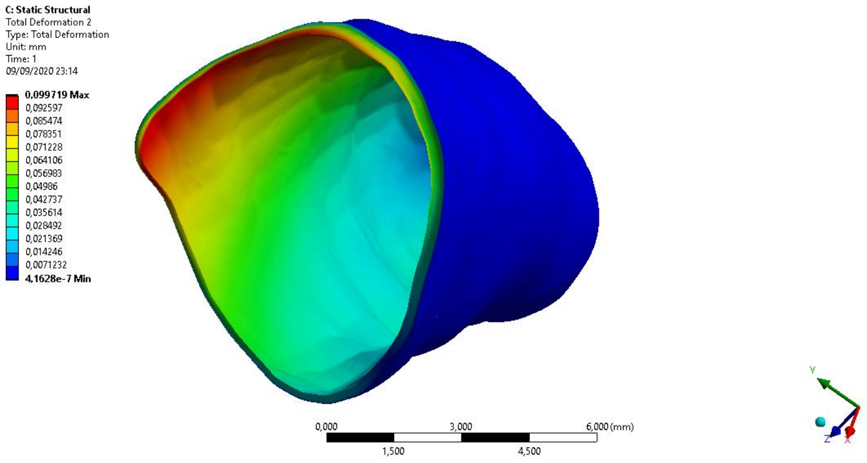

Maxilla with perforations

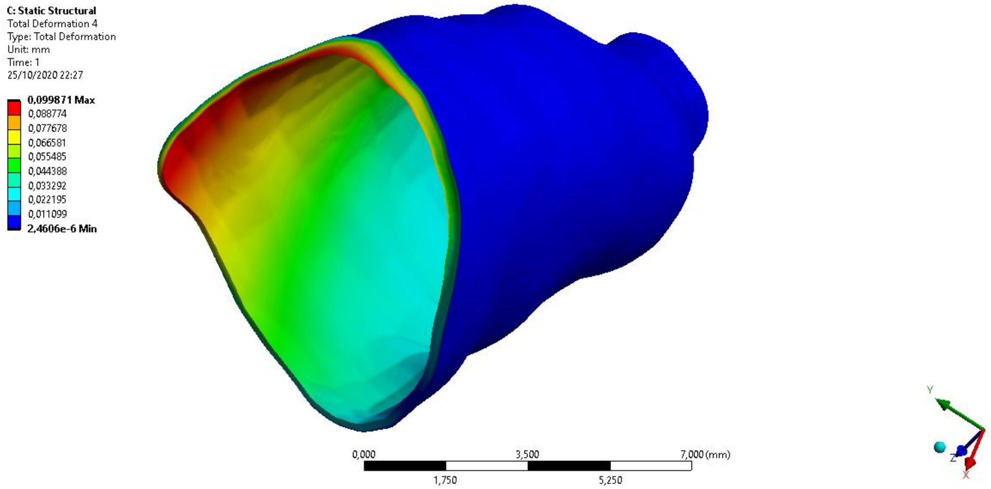

Maxilla without perforations with moment

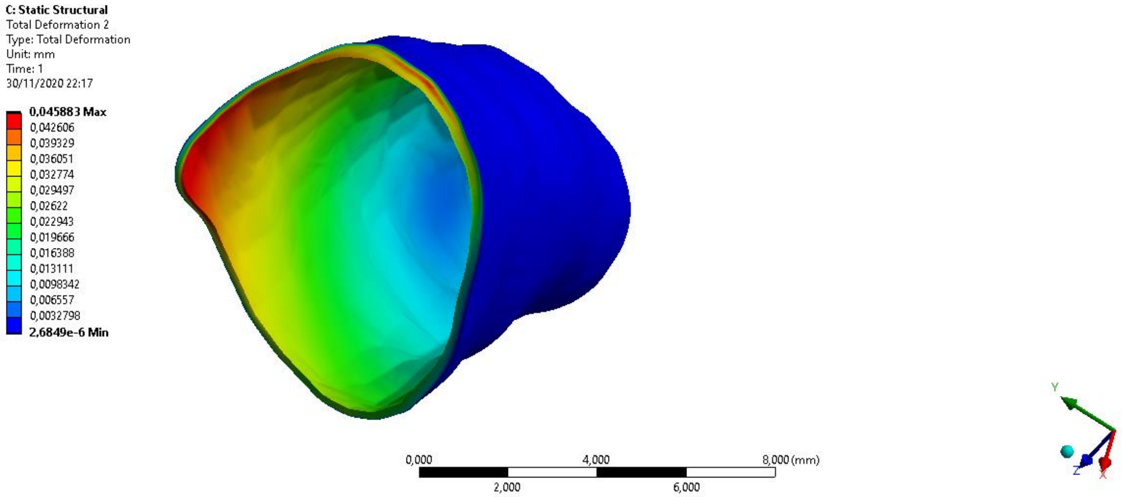

Maxilla with perforations with moment

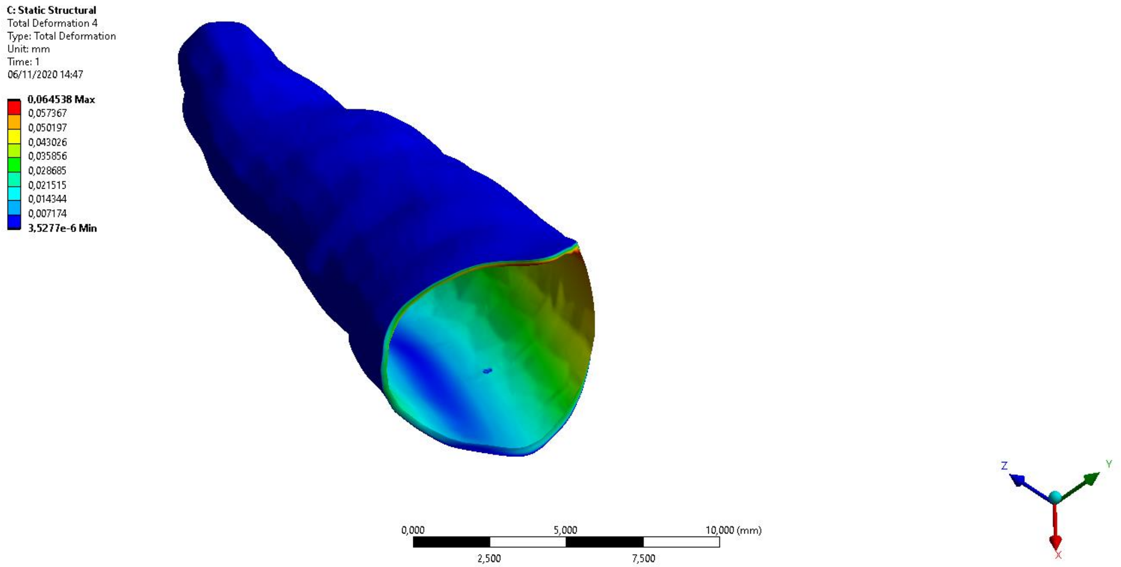

Maxilla without perforations with moment

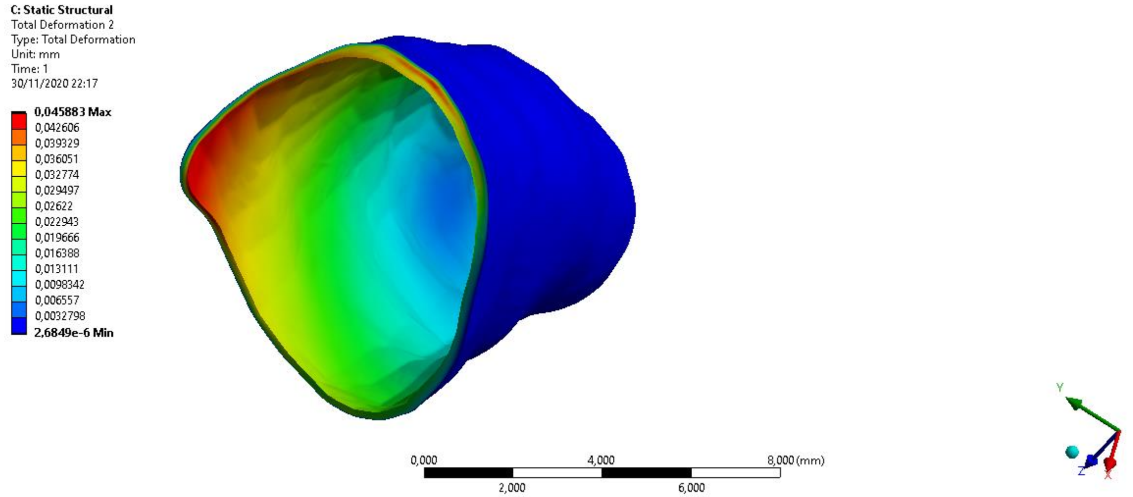

Maxilla with perforations with moment

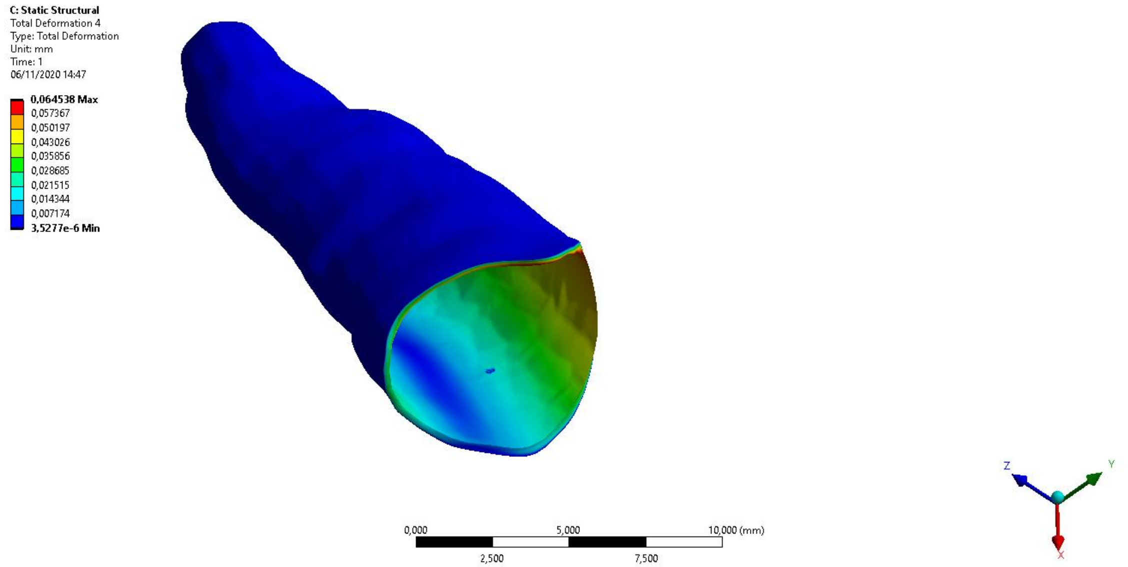

3-

Maxilla without perforations

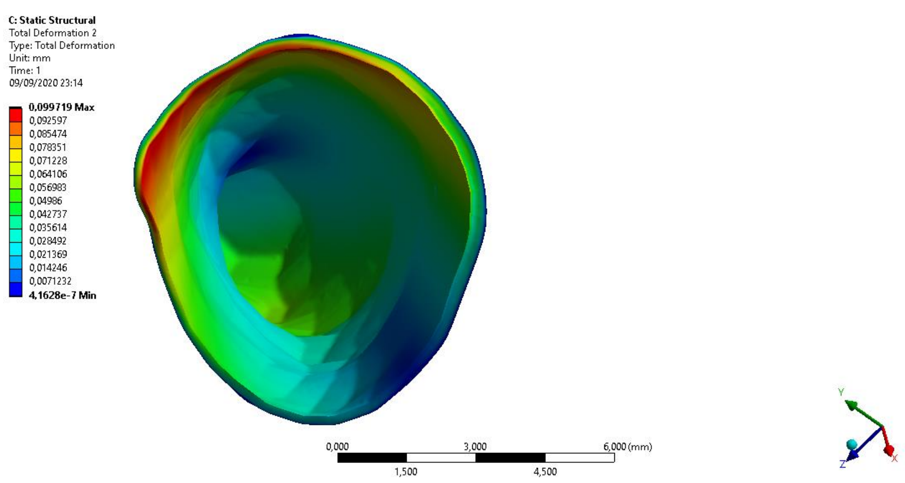

Maxilla with perforations

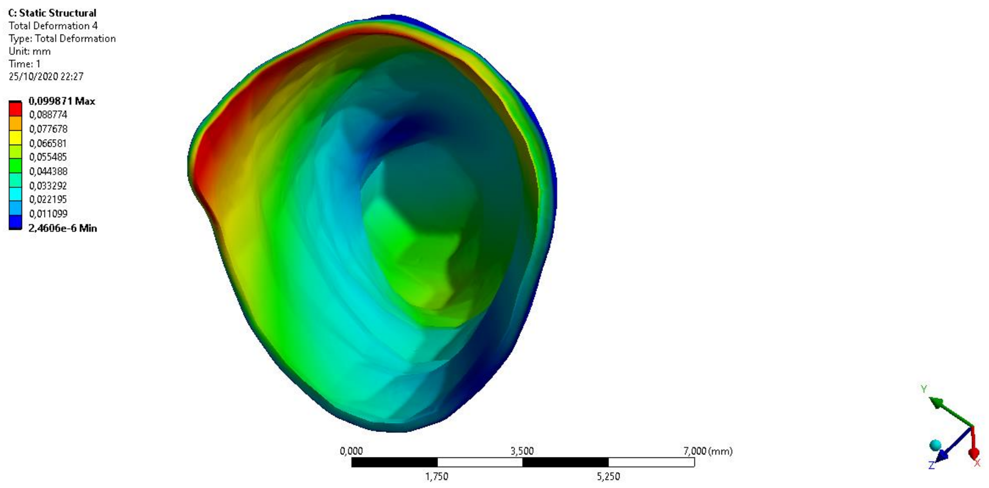

Maxilla without perforations with moment

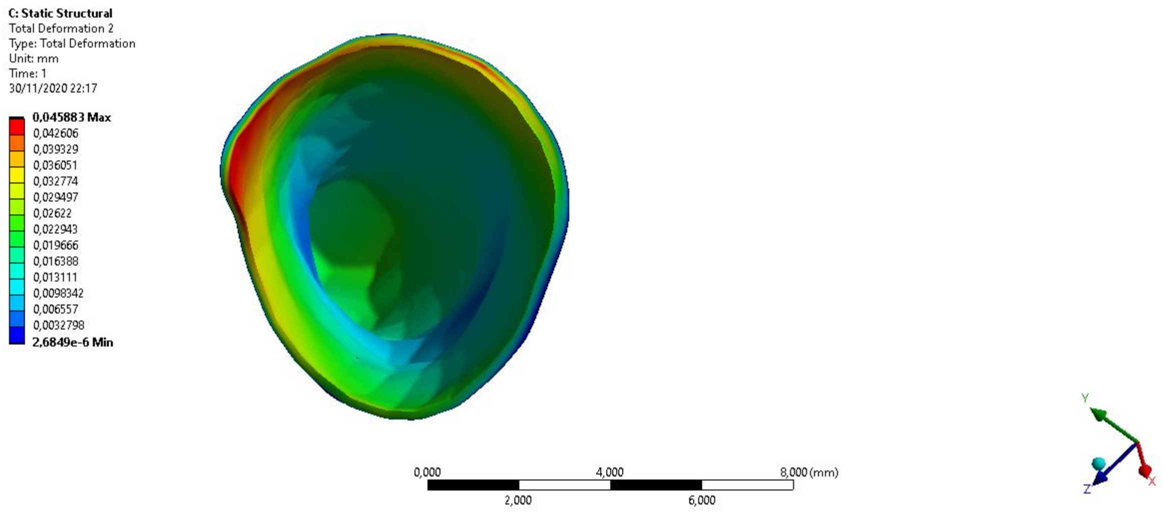

Maxilla with perforations with moment

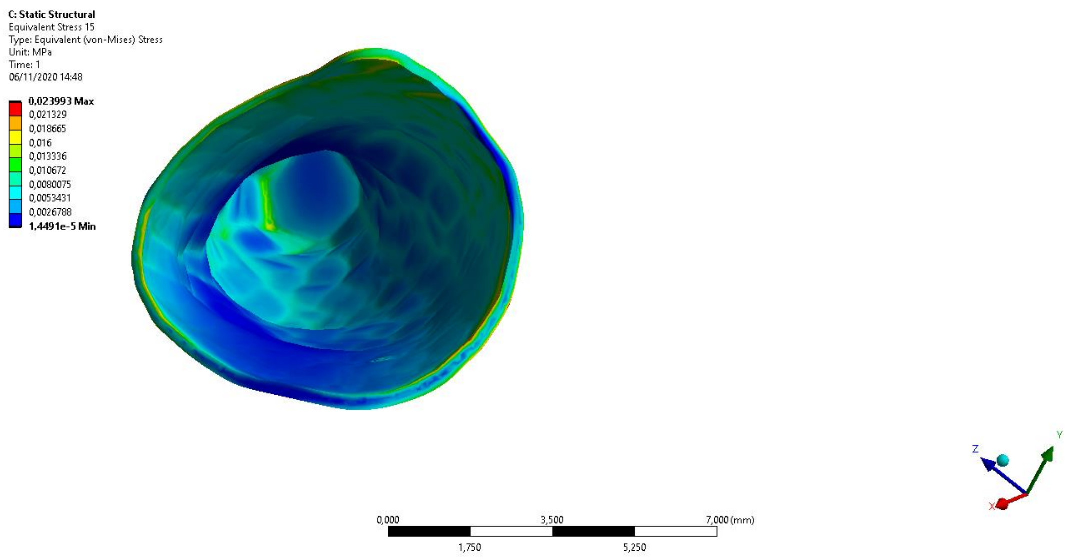

Maxilla with perforations with moment

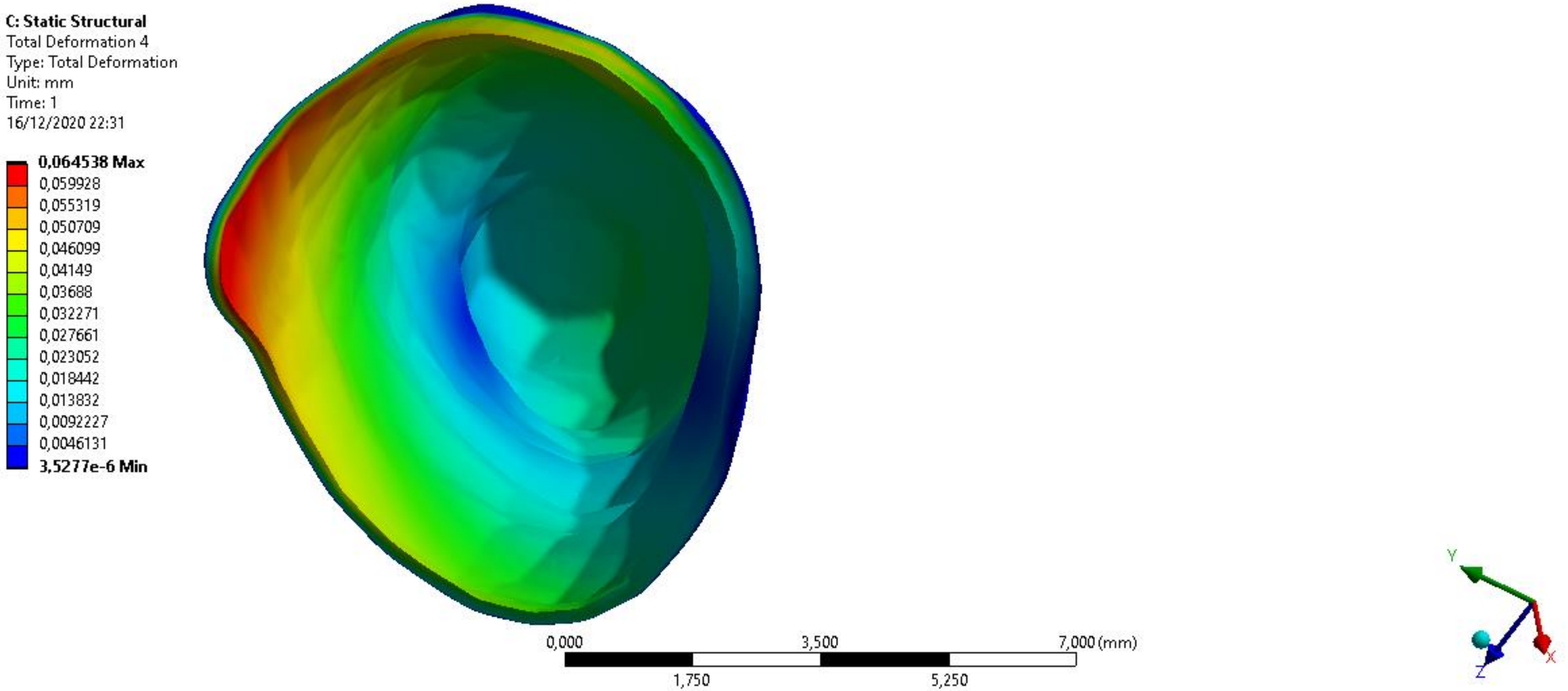

Maxilla without perforations with moment

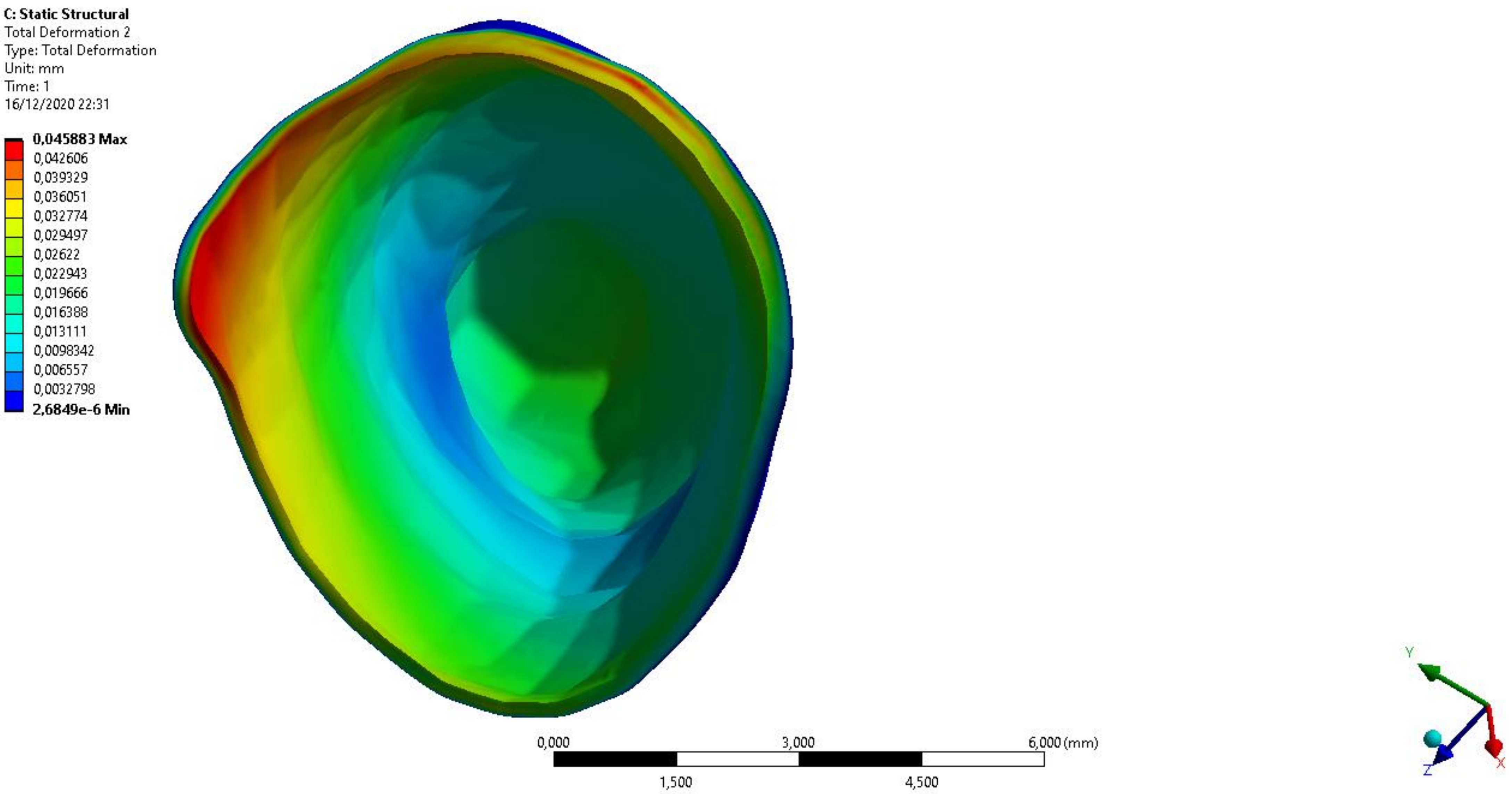

Maxilla without perforations

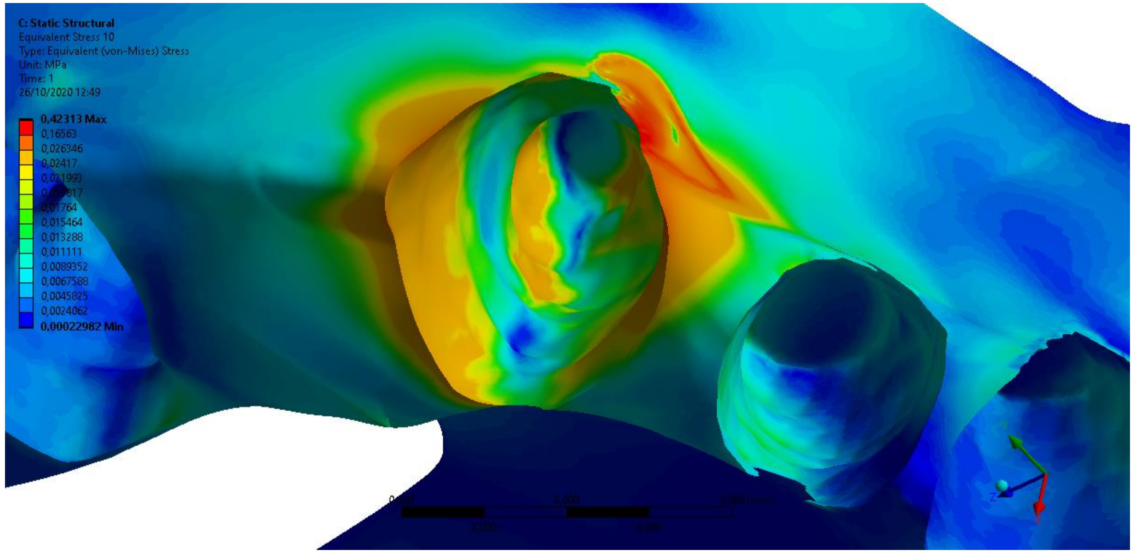

Maxilla without perforations with moment

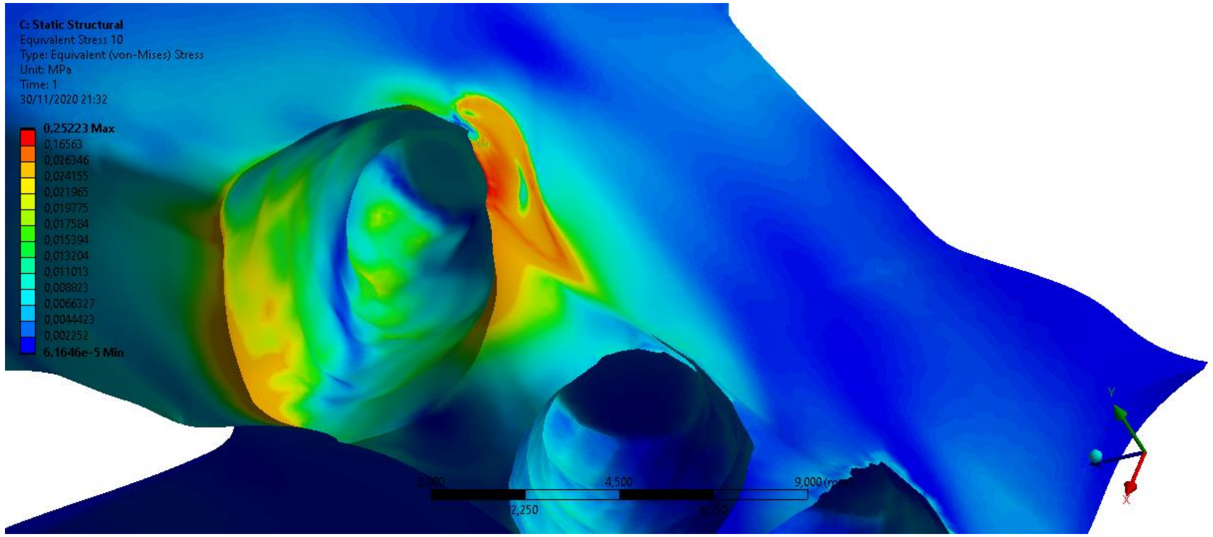

Maxilla with perforations with moment

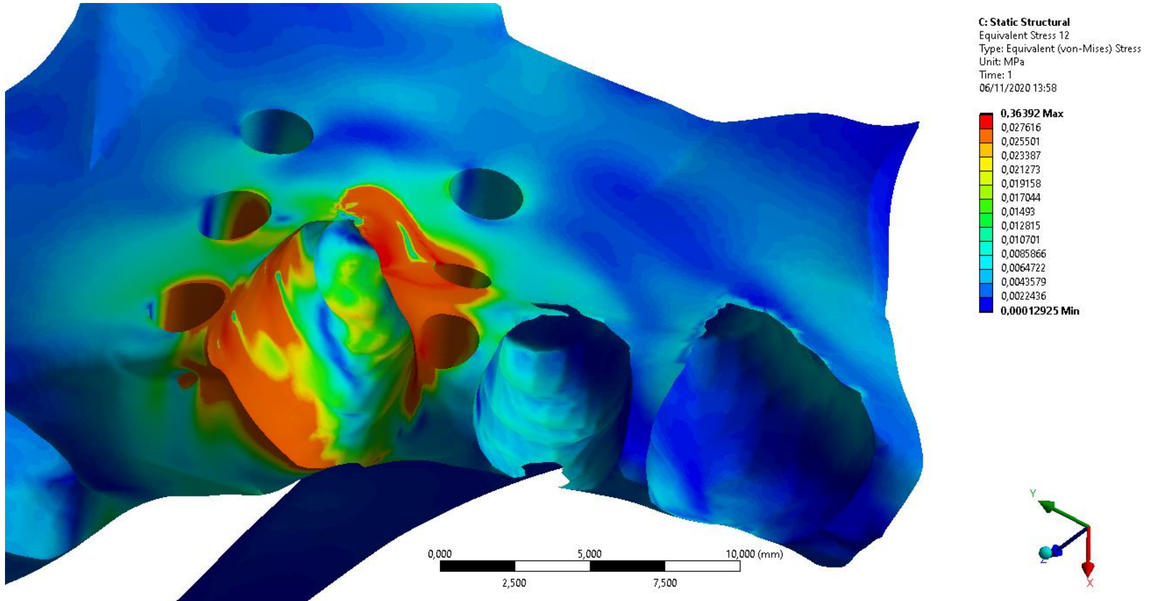

Maxilla without perforations

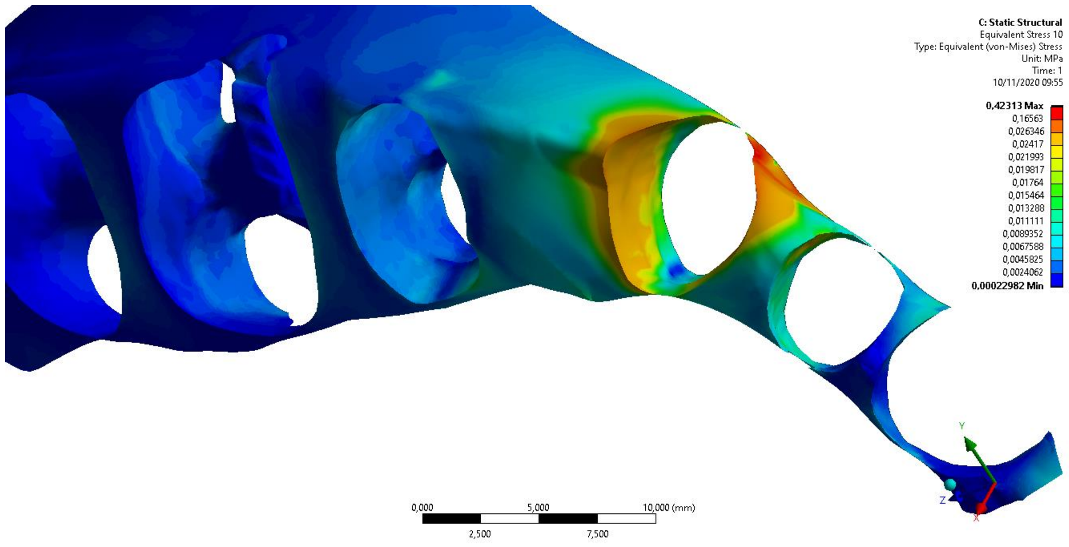

Maxilla without perforations with moment

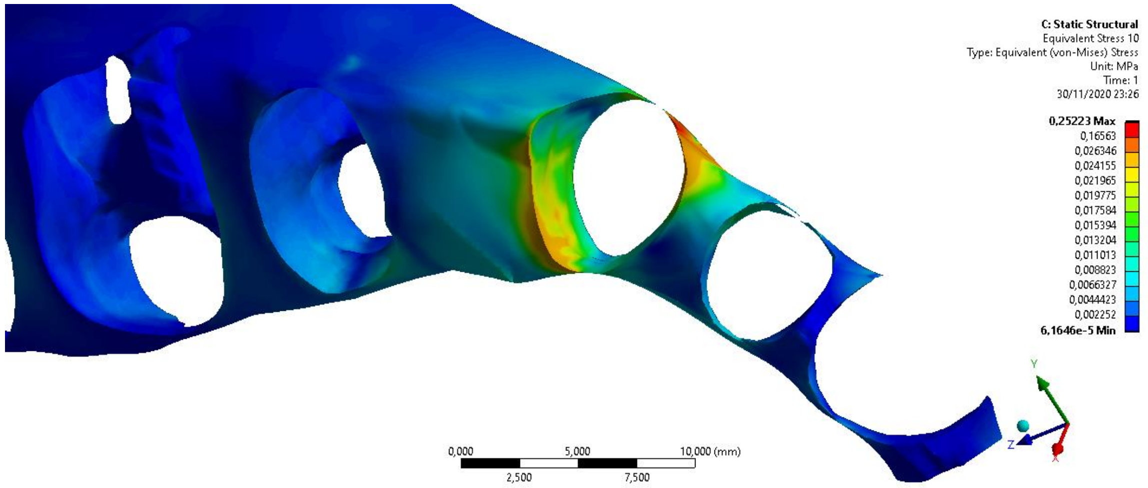

Maxilla without perforations

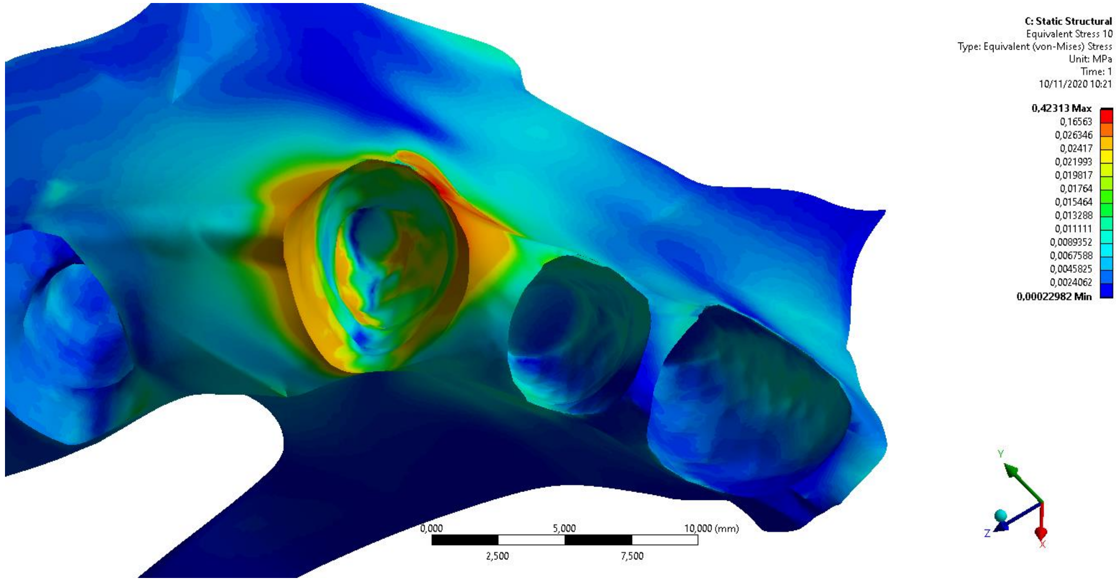

Maxilla with perforations

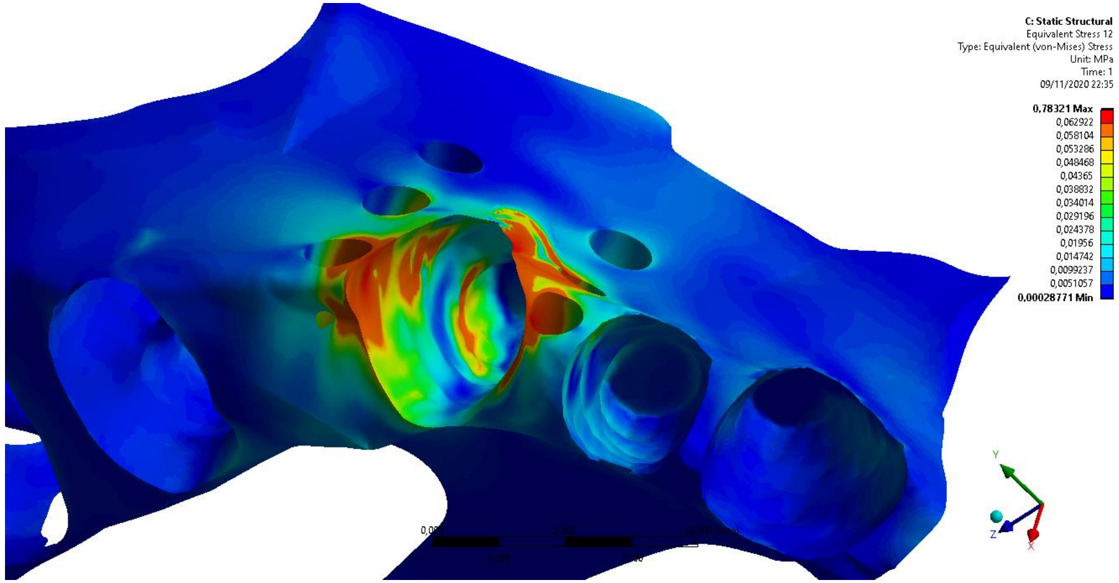

Maxilla without perforations with moment

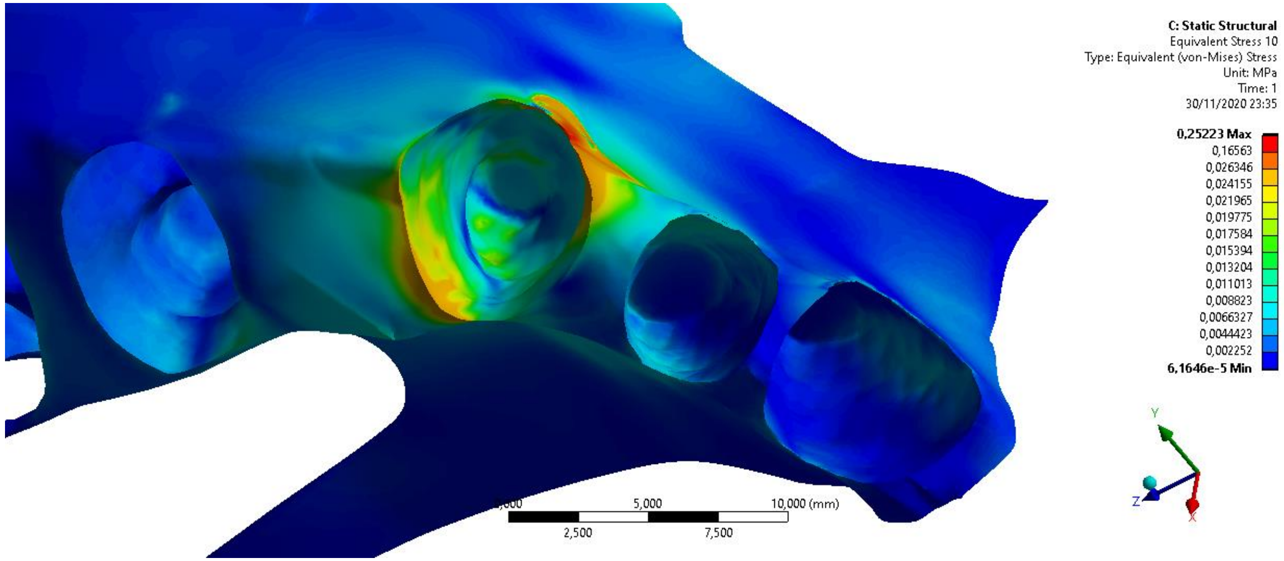

Maxilla with perforations with moment

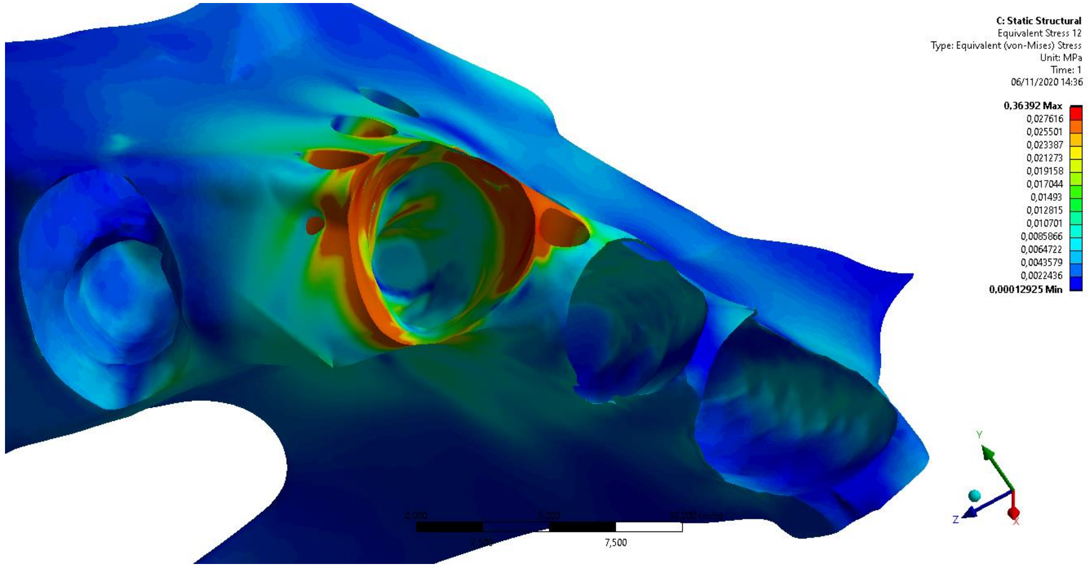

Maxilla with perforations

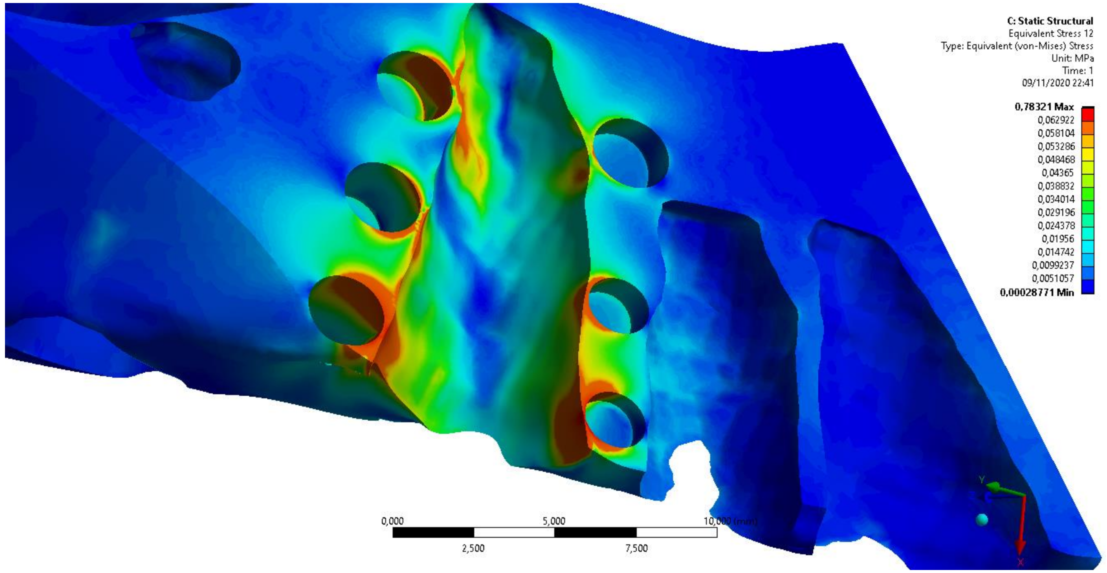

Maxilla with perforations with moment

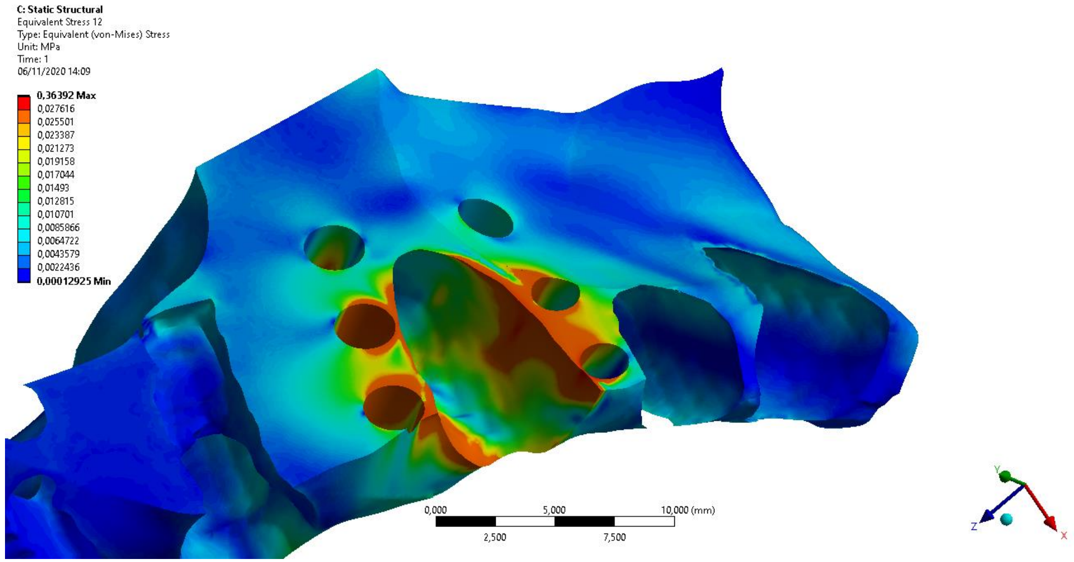

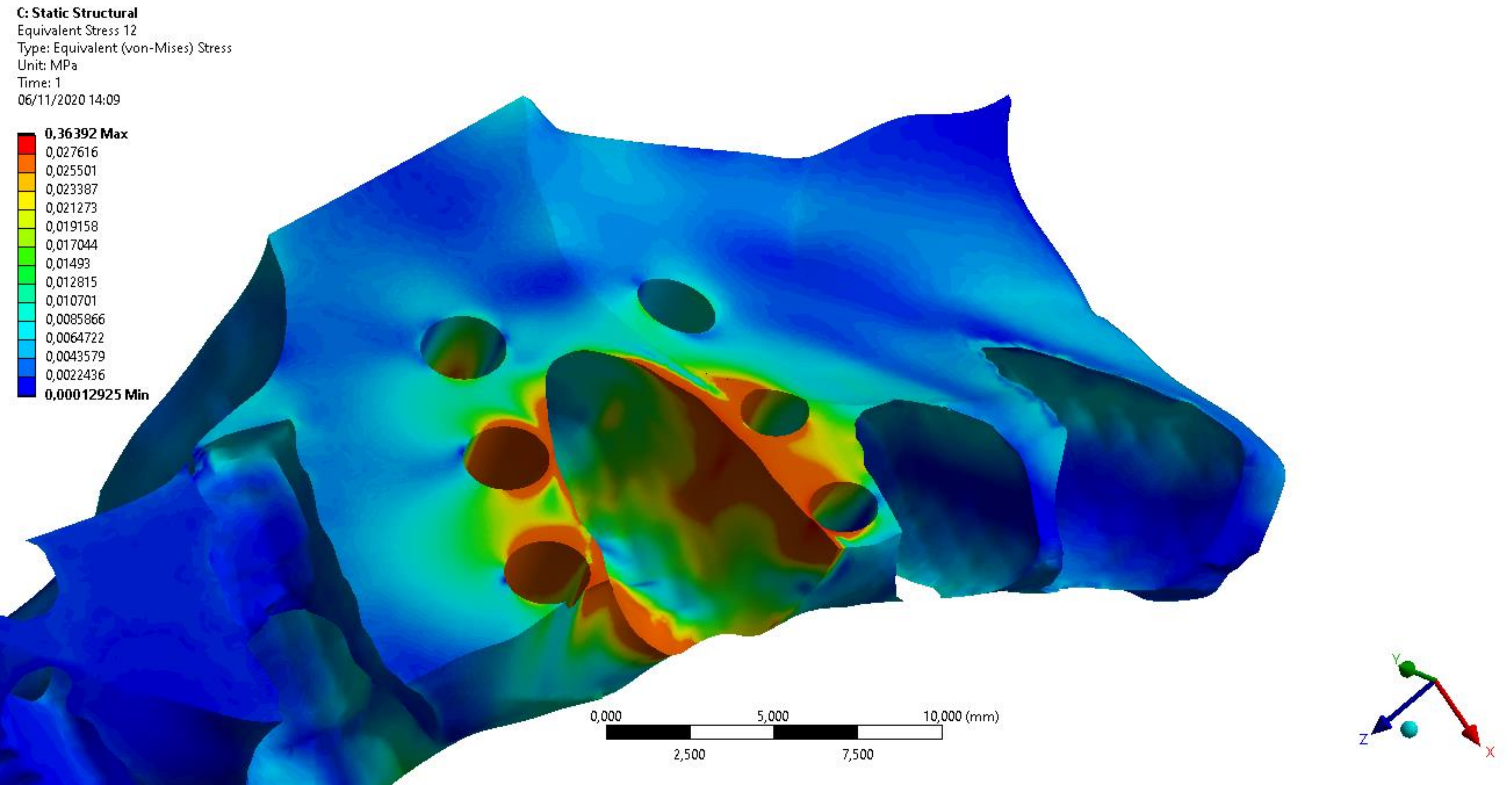
C: Static Structural
Equivalent Stress 12
Type: Equivalent (von-Mises) Stress
Unit: MPa
Time: 1
06/11/2020 14:09
0,36392 Max
0,027616
0,025501
0,023387
0,021273
0,019158
0,017044
0,01493
0,012815
0,010701
0,0085866
0,0064722
0,0043579
0,0022436
0,00012925 Min
0,000
5,000
10,000 (mm)
2,500
7,500
Y
Z
X

Maxilla without perforations with moment

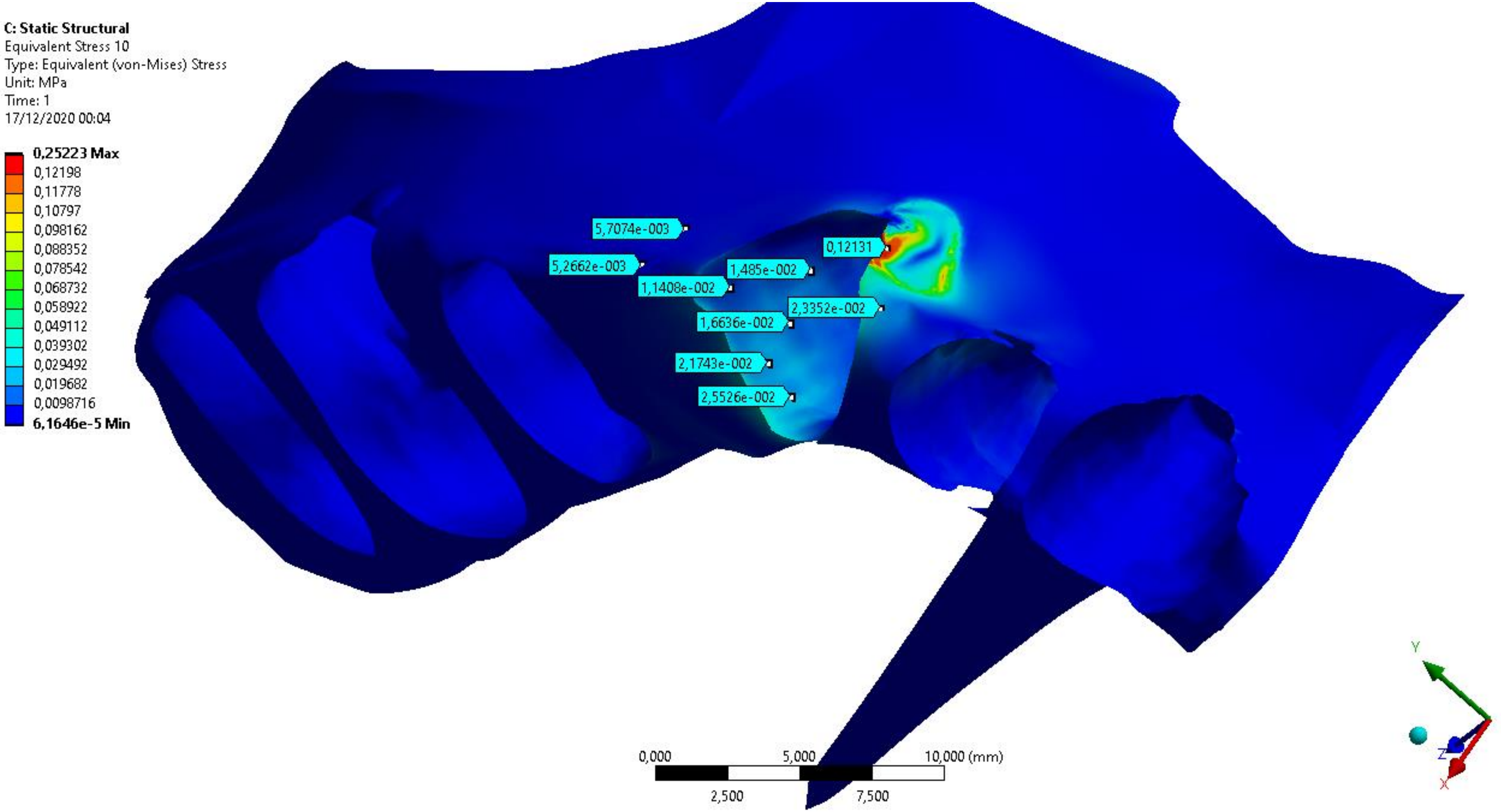

Maxilla with perforations with moment

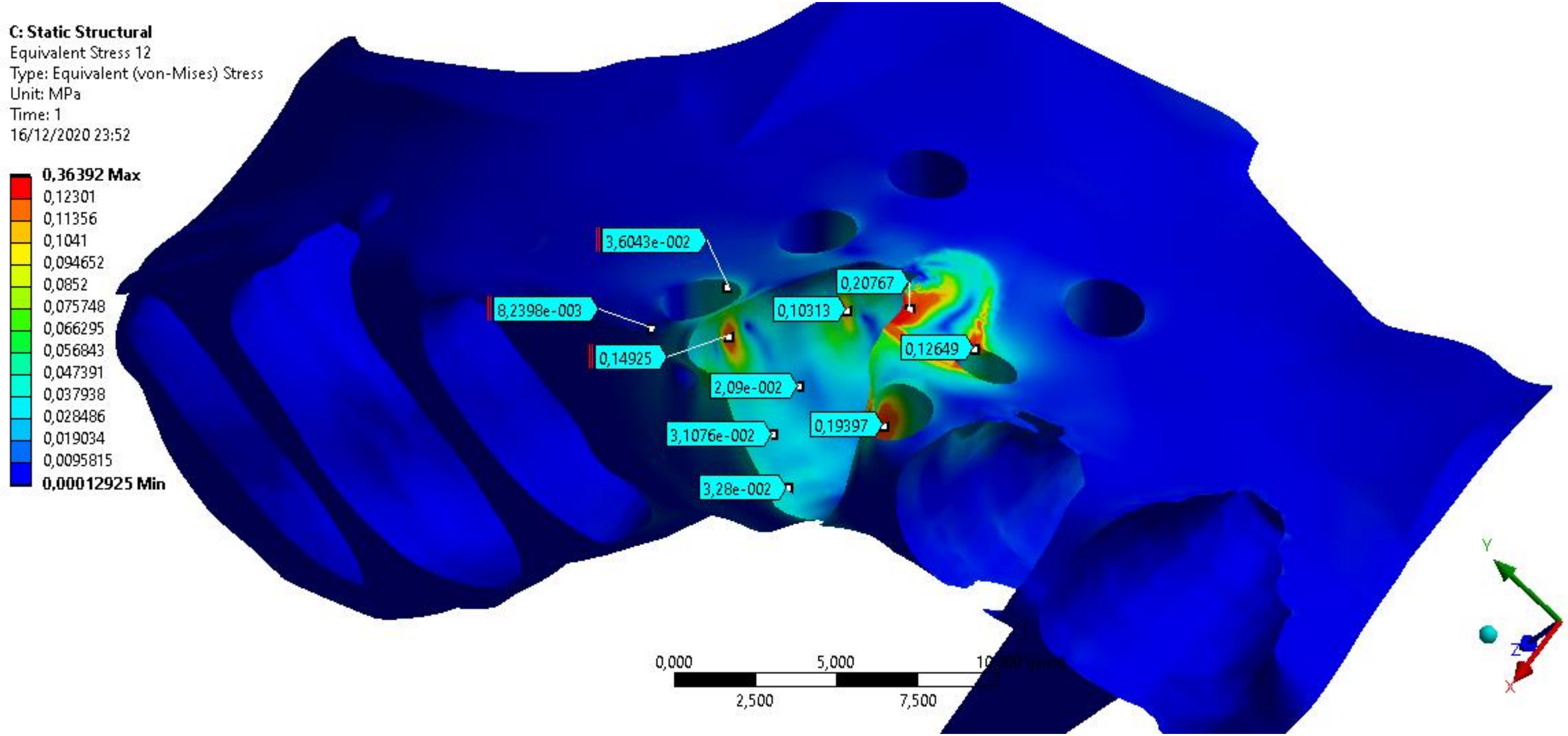

Maxilla without perforations with moment

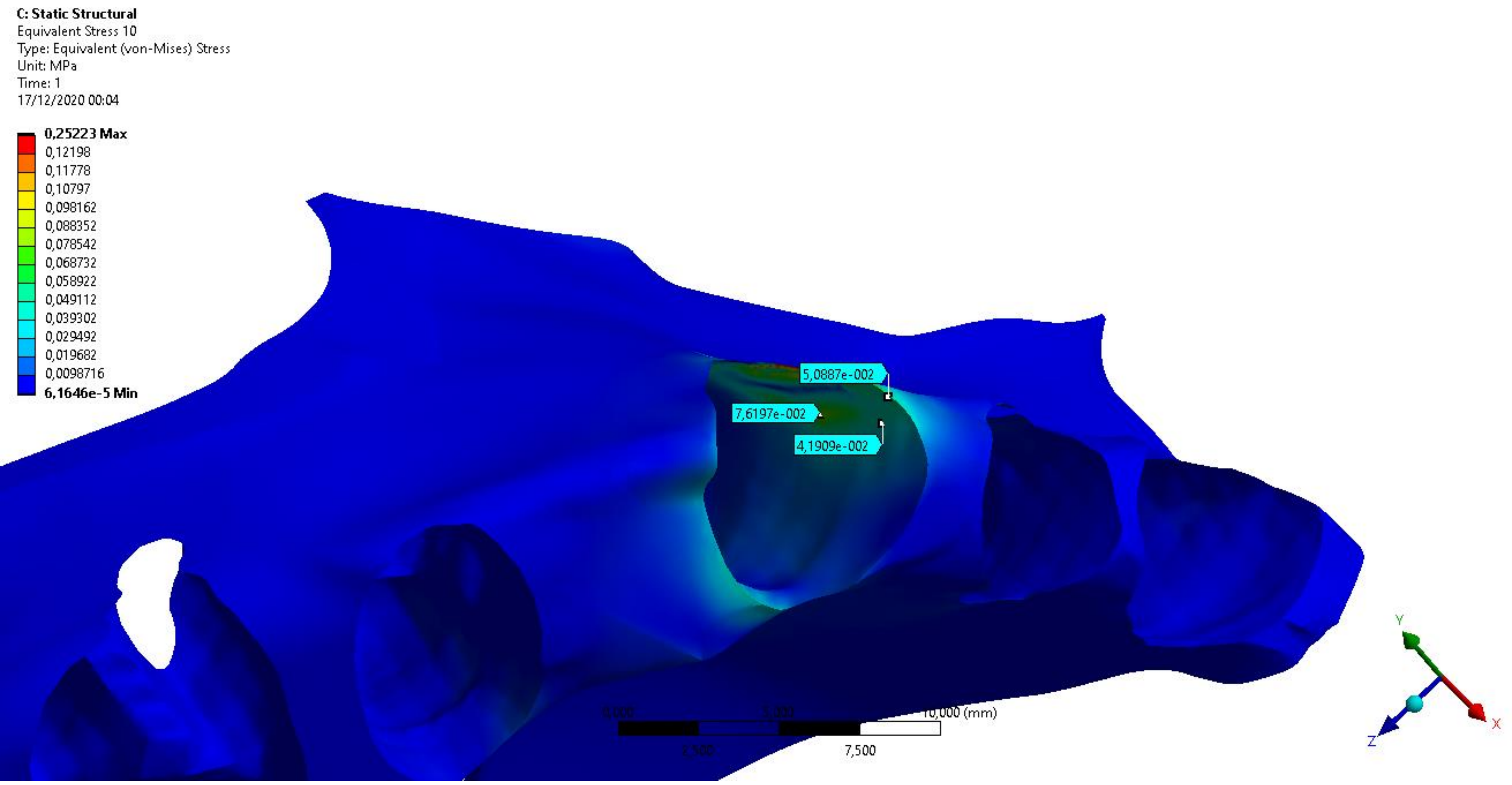

Maxilla with perforations with moment

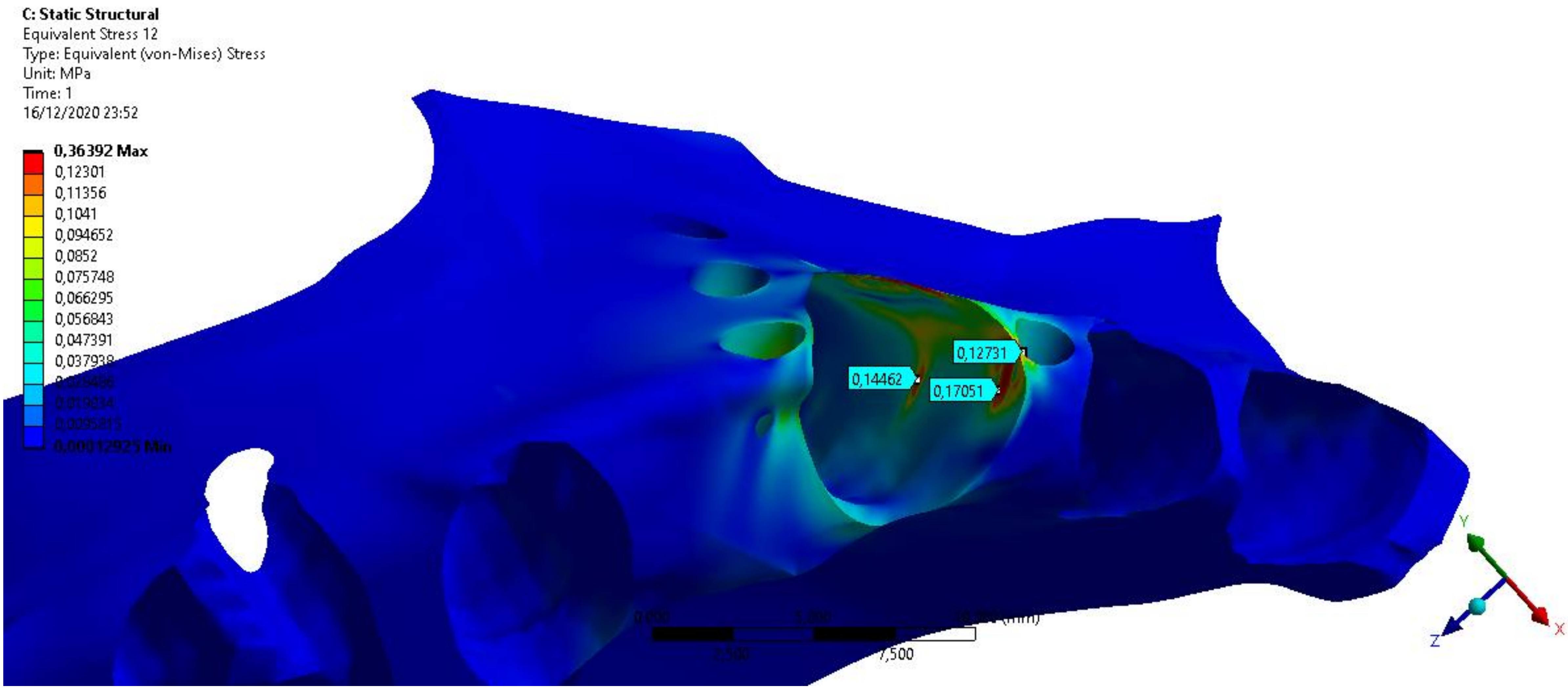

Maxilla without perforations with moment

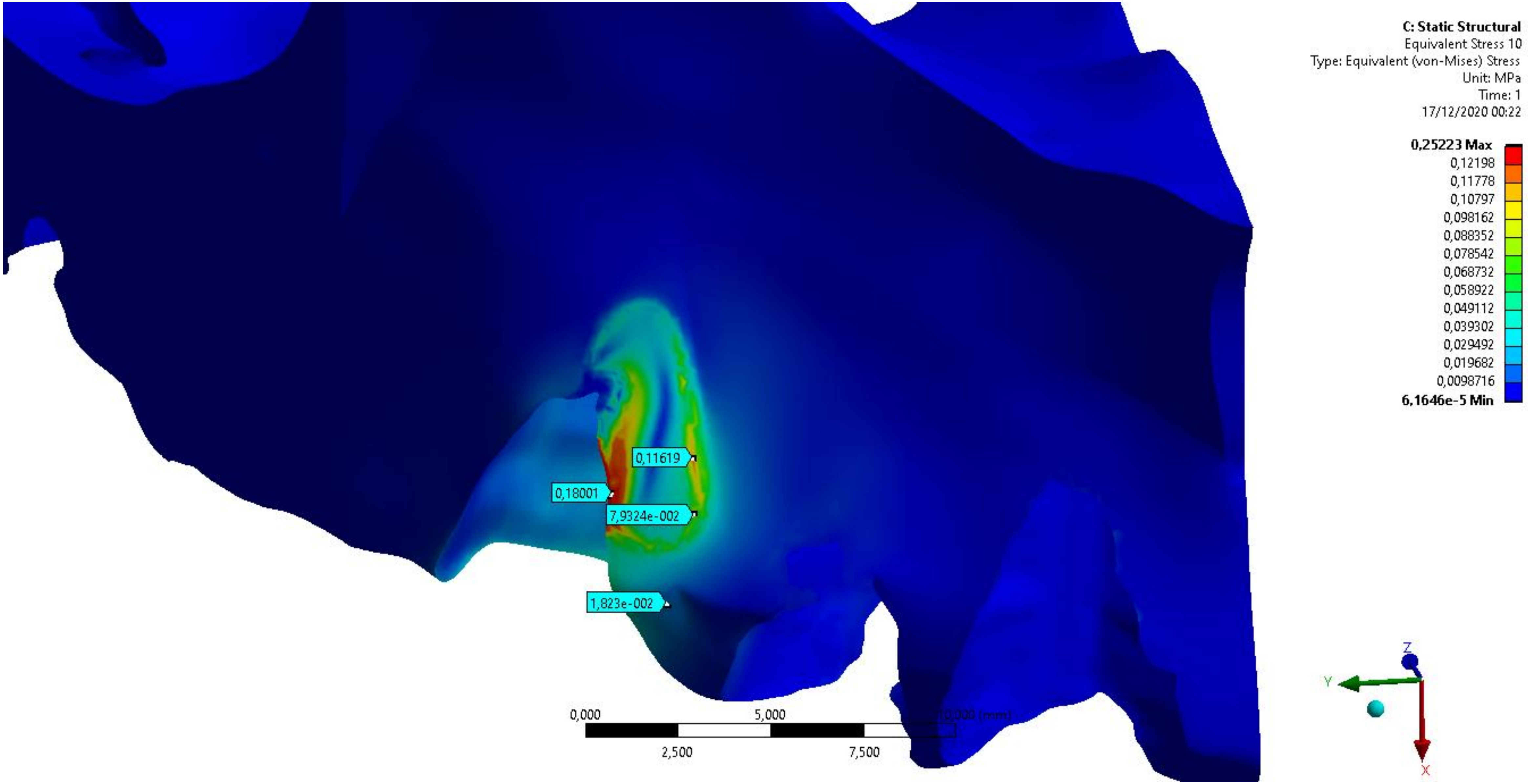

Maxilla with perforations with moment

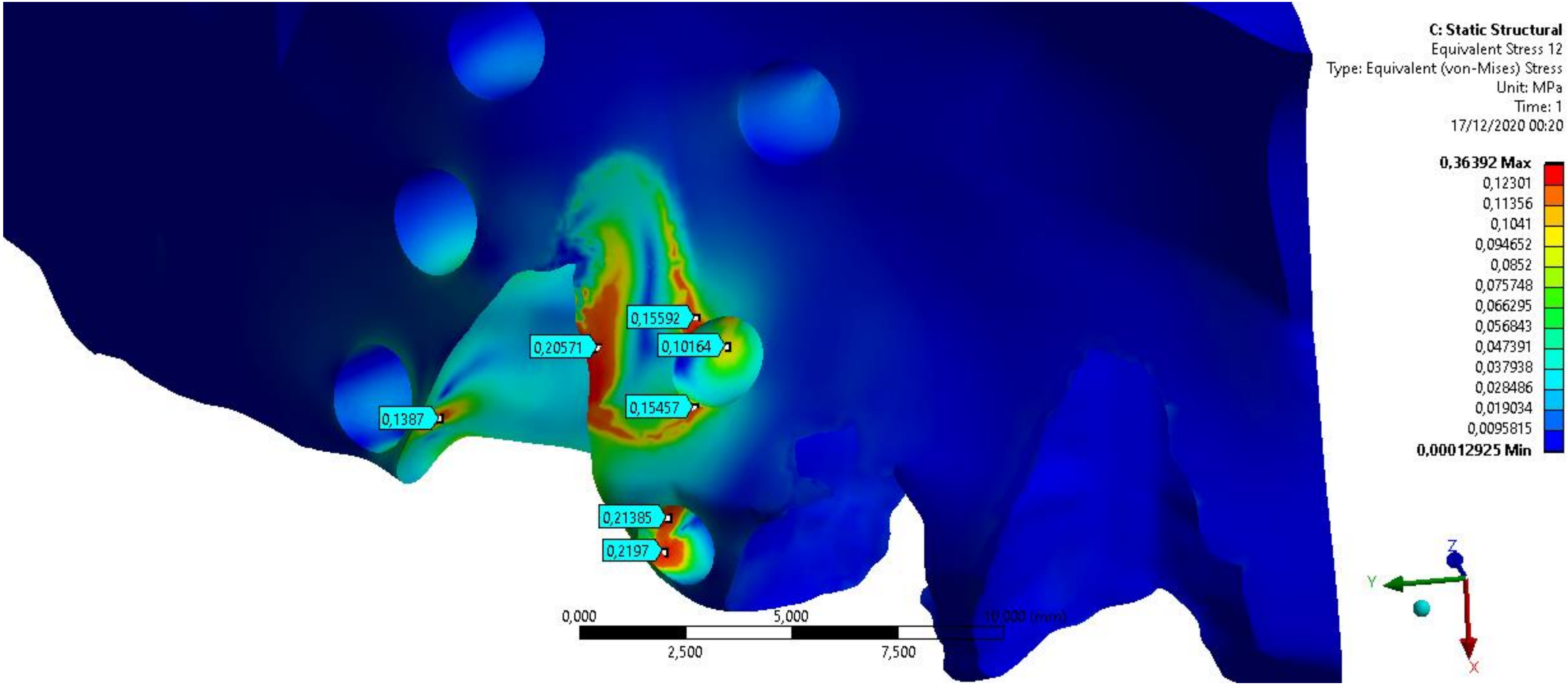

Maxilla without perforations with moment

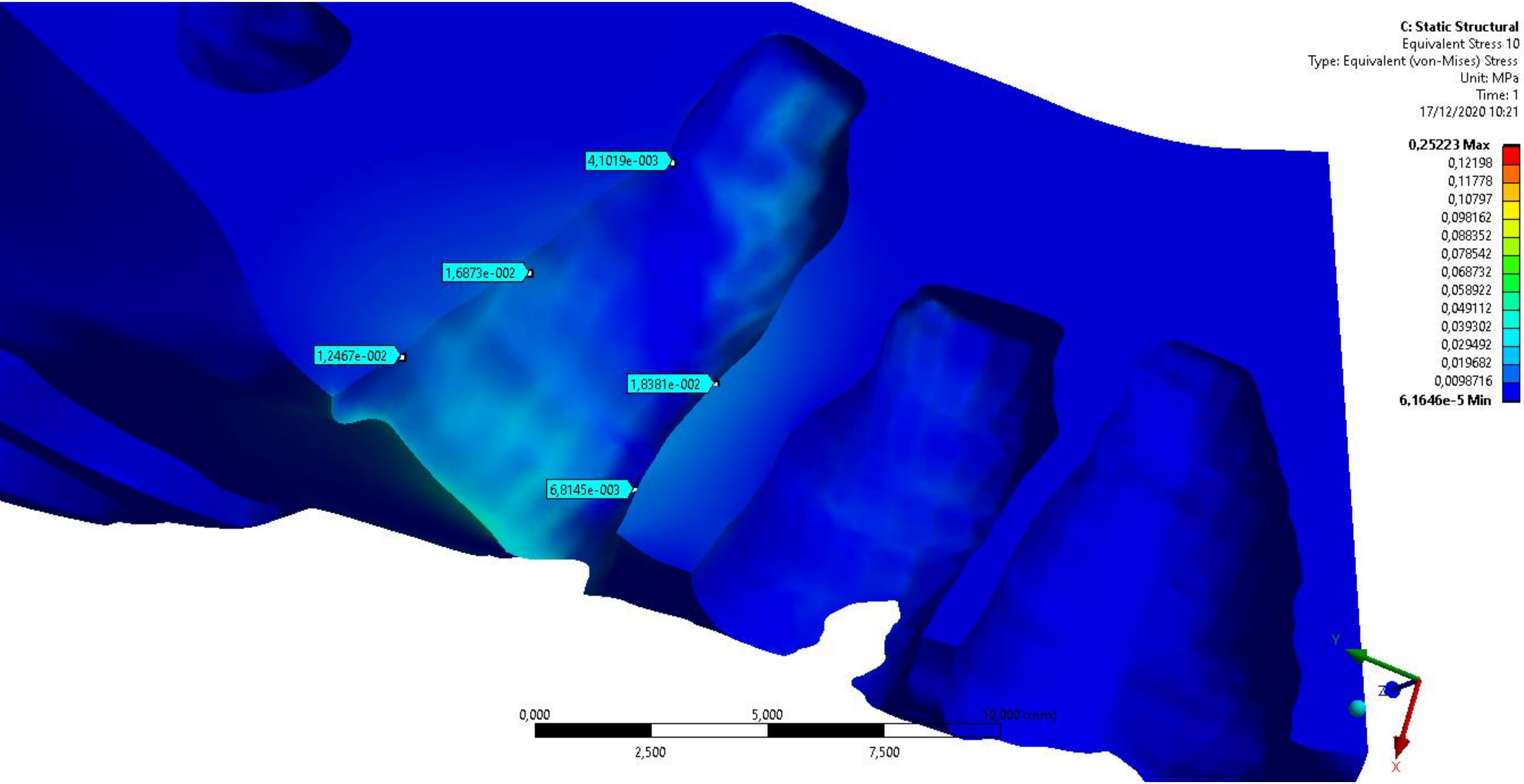

Maxilla with perforations with moment

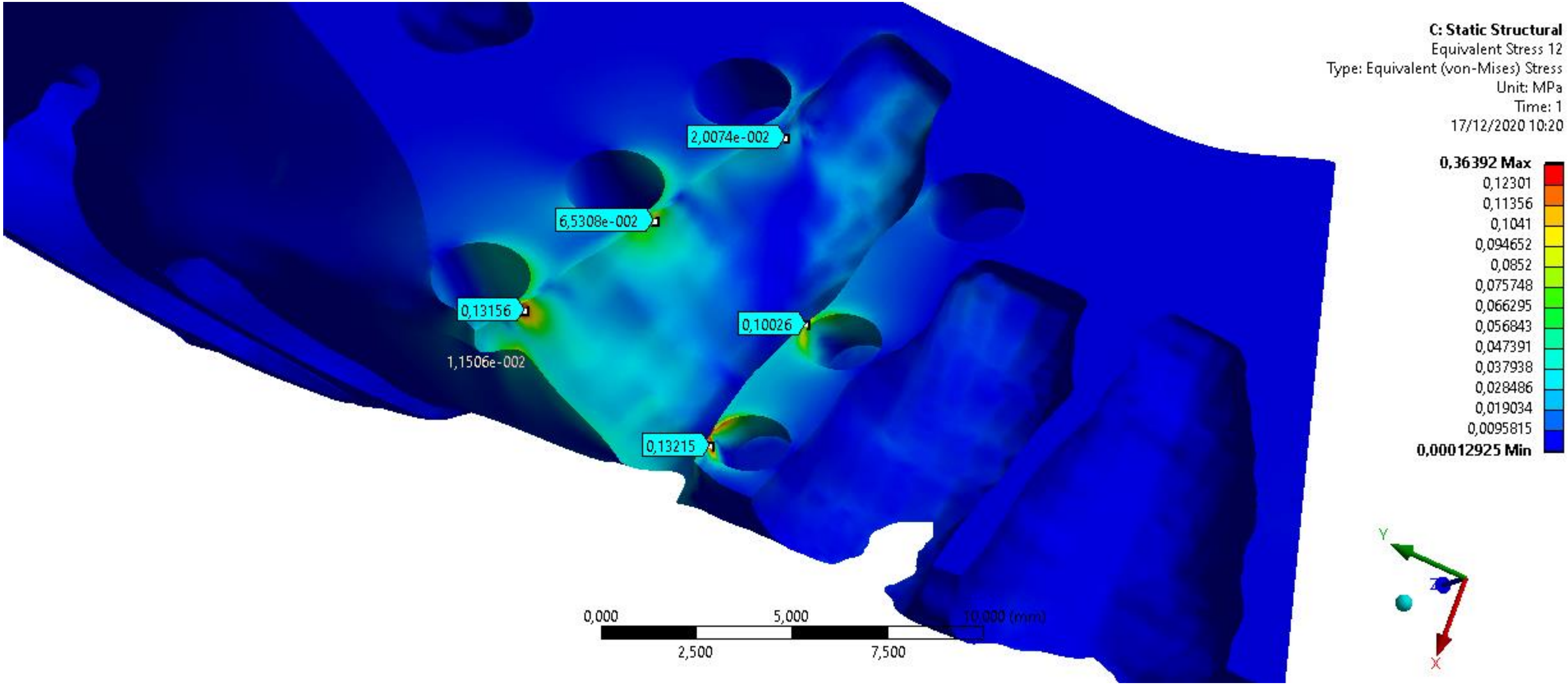

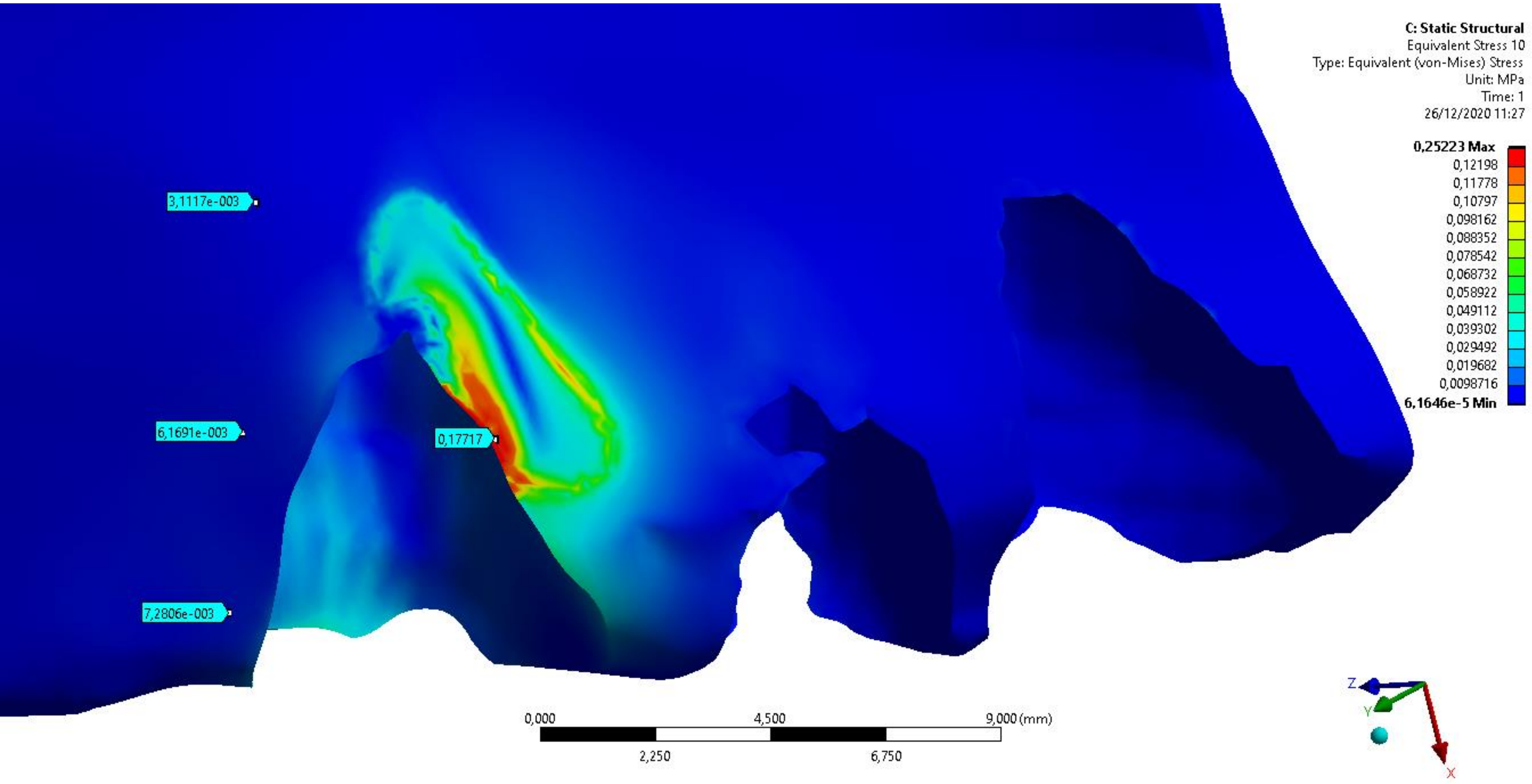
C: Static Structural
Equivalent Stress 10
Type: Equivalent (von-Mises) Stress
Unit: MPa
Time: 1
26/12/2020 11:27
0,25223 Max
0,12198
0,11778
0,10797
0,098162
0,088352
0,078542
0,068732
0,058922
0,049112
0,039302
0,029492
0,019682
0,0098716
6,1646e-5 Min
3,1117e-003
6,1691e-003
0,17717
7,2806e-003
0,000
4,500
9,000 (mm)
2,250
6,750
Z
Y
X

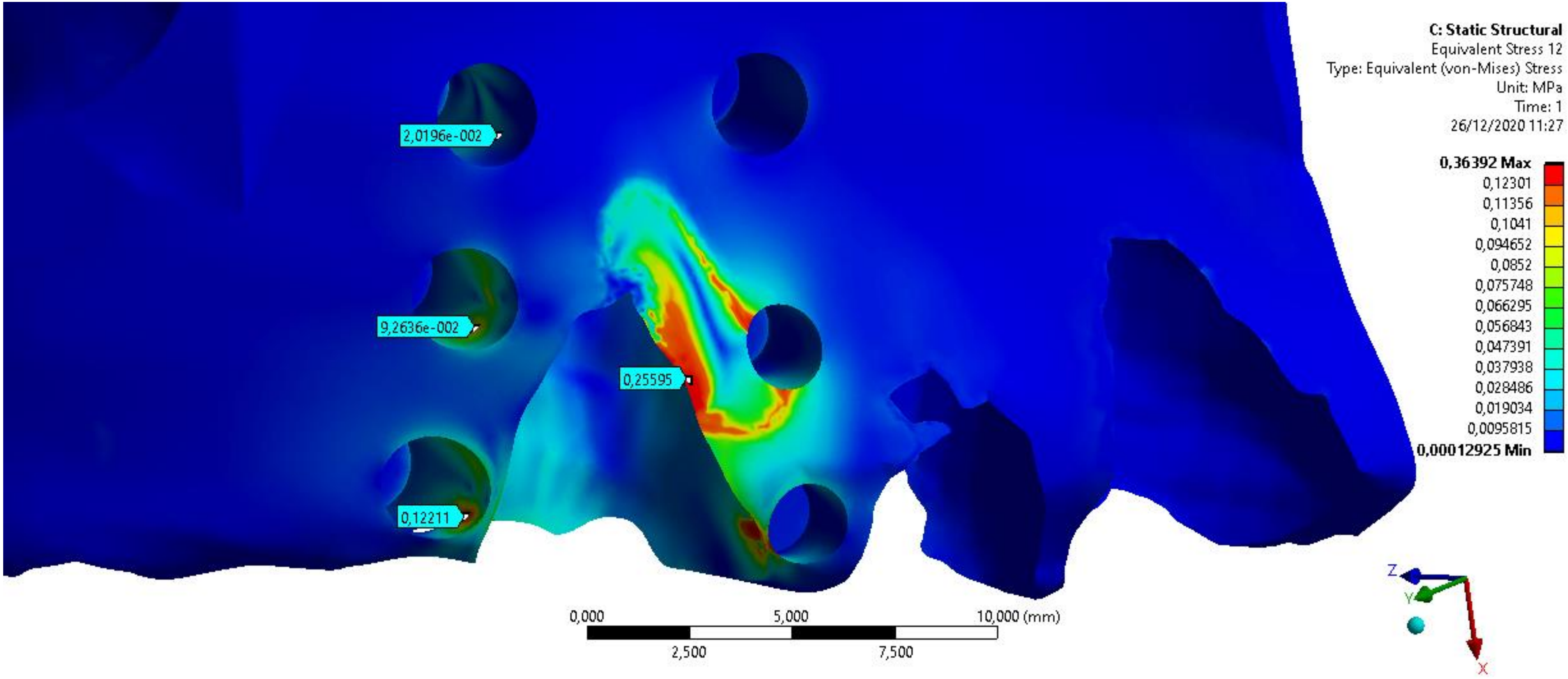
C: Static Structural
Equivalent Stress 12
Type: Equivalent (von-Mises) Stress
Unit: MPa
Time: 1
26/12/2020 11:27
0,36392 Max
0,12301
0,11356
0,1041
0,094652
0,0852
0,075748
0,066295
0,056843
0,047391
0,037938
0,028486
0,019034
0,0095815
0,00012925 Min
2,0196e-002
9,2636e-002
0,25595
0,12211
0,000
5,000
10,000 (mm)
2,500
7,500
Z
Y
X
