# Supplementary material for: Micro-osteoperforation for enhancement of orthodontic movement: A mechanical analysis using the finite element method
Source: PLoS One. 2024 Aug 19;19(8):e0308739. doi: 10.1371/journal.pone.0308739 (PMC11332926; doi:10.1371/journal.pone.0308739)
Supplement: S4 Fig — (PDF) [file pone.0308739.s012.pdf]

S12. Final images 1

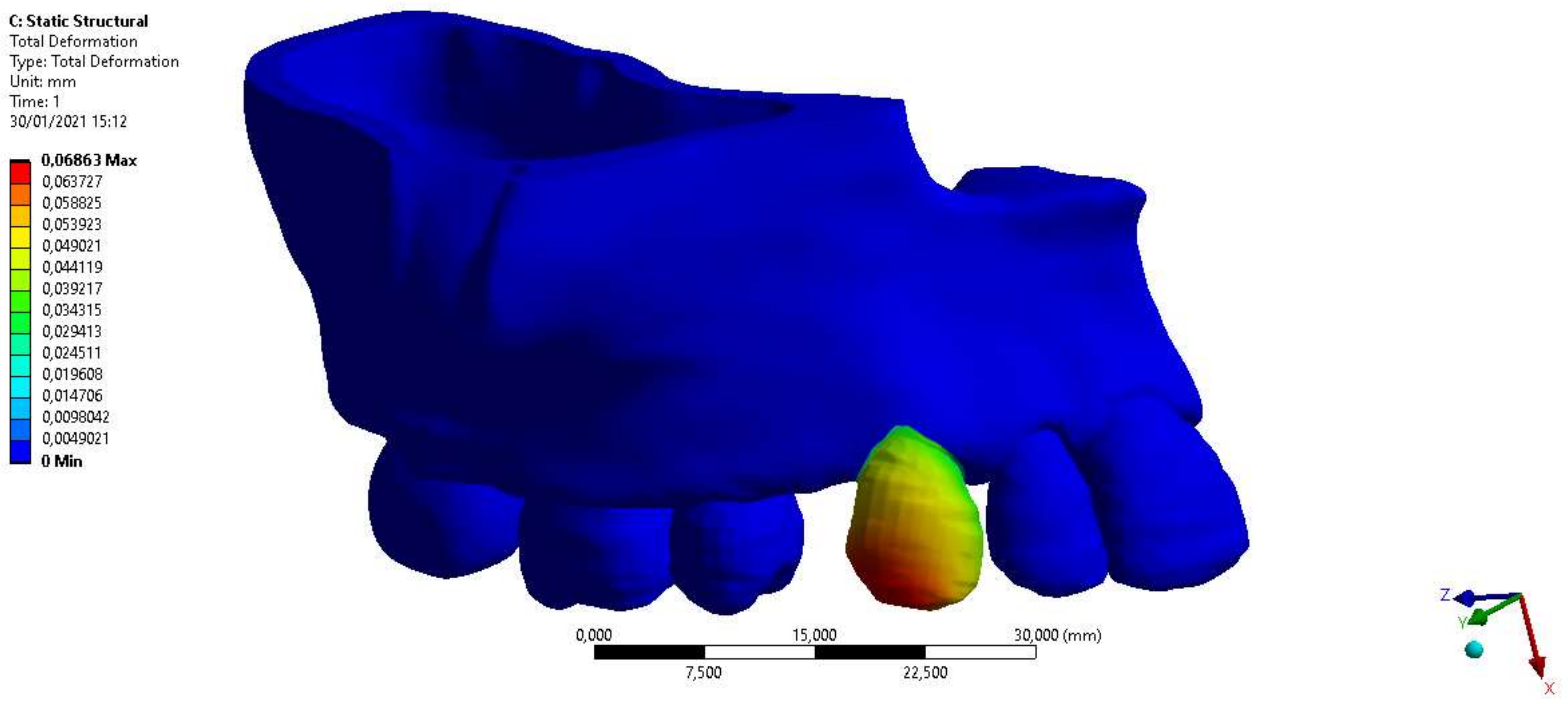

C: Static Structural
Total Deformation
Type: Total Deformation
Unit: mm
Time: 1
30/01/2021 15:12
0,06863 Max
0,063727
0,058825
0,053923
0,049021
0,044119
0,039217
0,034315
0,029413
0,024511
0,019608
0,014706
0,0098042
0,0049021
0 Min
0,000
15,000
30,000 (mm)
7,500
22,500
Z
Y
X

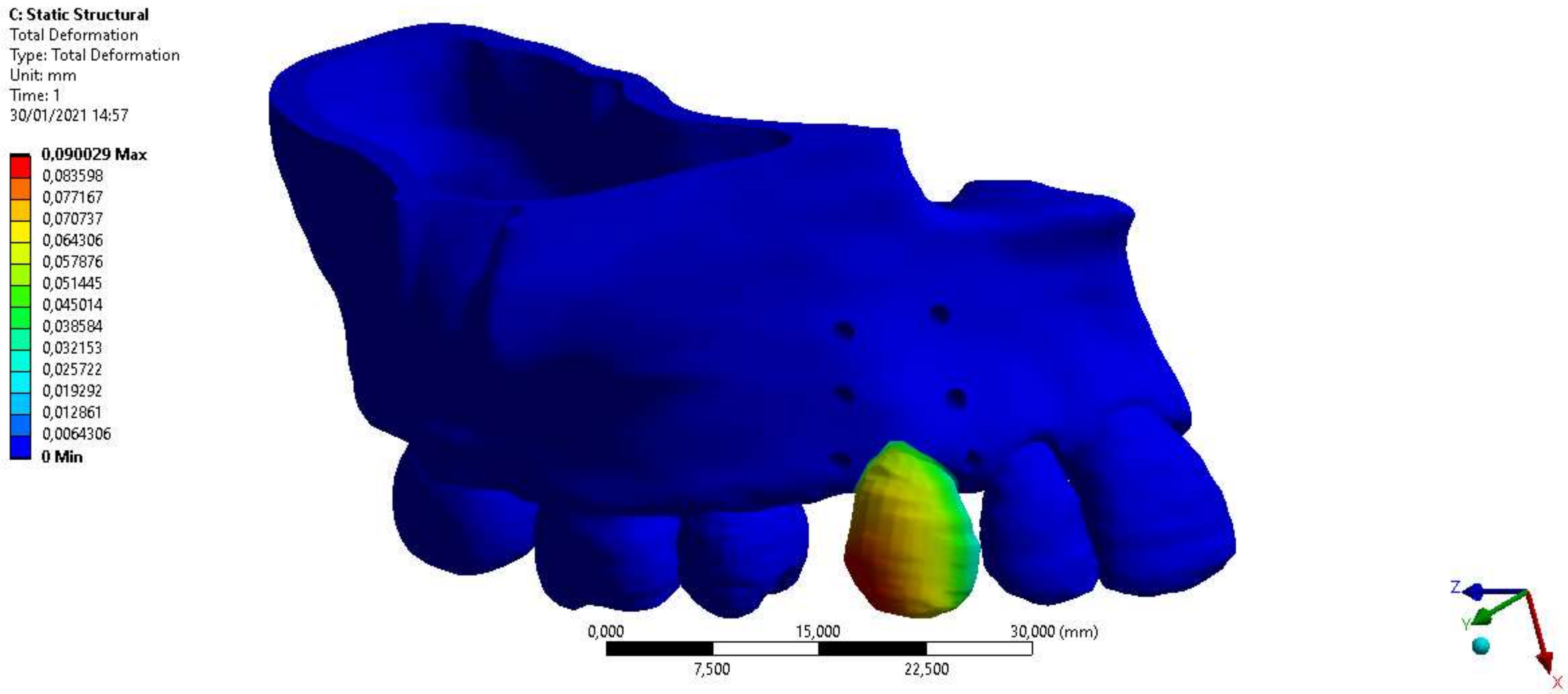
C: Static Structural
Total Deformation
Type: Total Deformation
Unit: mm
Time: 1
30/01/2021 14:57
0,090029 Max
0,083598
0,077167
0,070737
0,064306
0,057876
0,051445
0,045014
0,038584
0,032153
0,025722
0,019292
0,012861
0,0064306
0 Min
0,000
15,000
30,000 (mm)
7,500
22,500
Z
Y
X

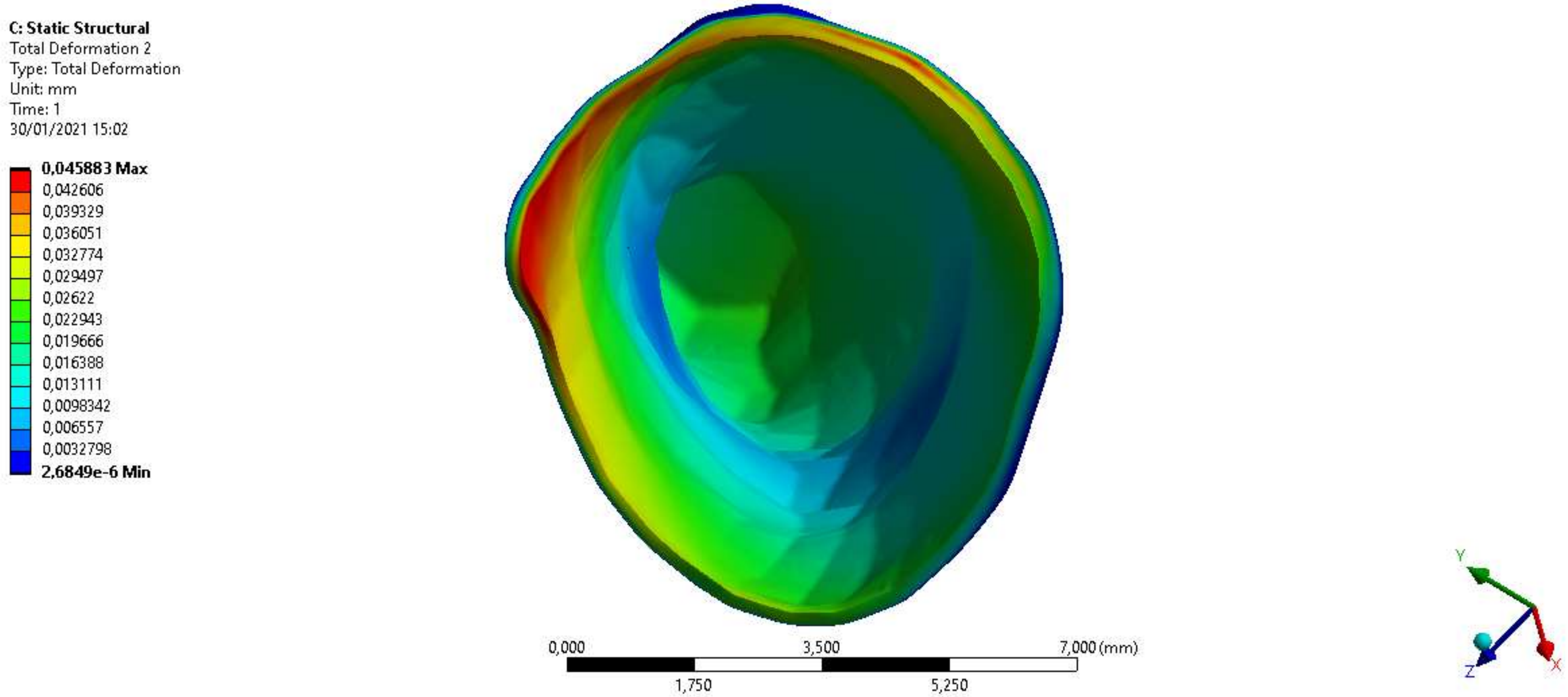
C: Static Structural
Total Deformation 2
Type: Total Deformation
Unit: mm
Time: 1
30/01/2021 15:02
0,045883 Max
0,042606
0,039329
0,036051
0,032774
0,029497
0,02622
0,022943
0,019666
0,016388
0,013111
0,0098342
0,006557
0,0032798
2,6849e-6 Min
0,000
3,500
7,000 (mm)
1,750
5,250
Y
Z
X

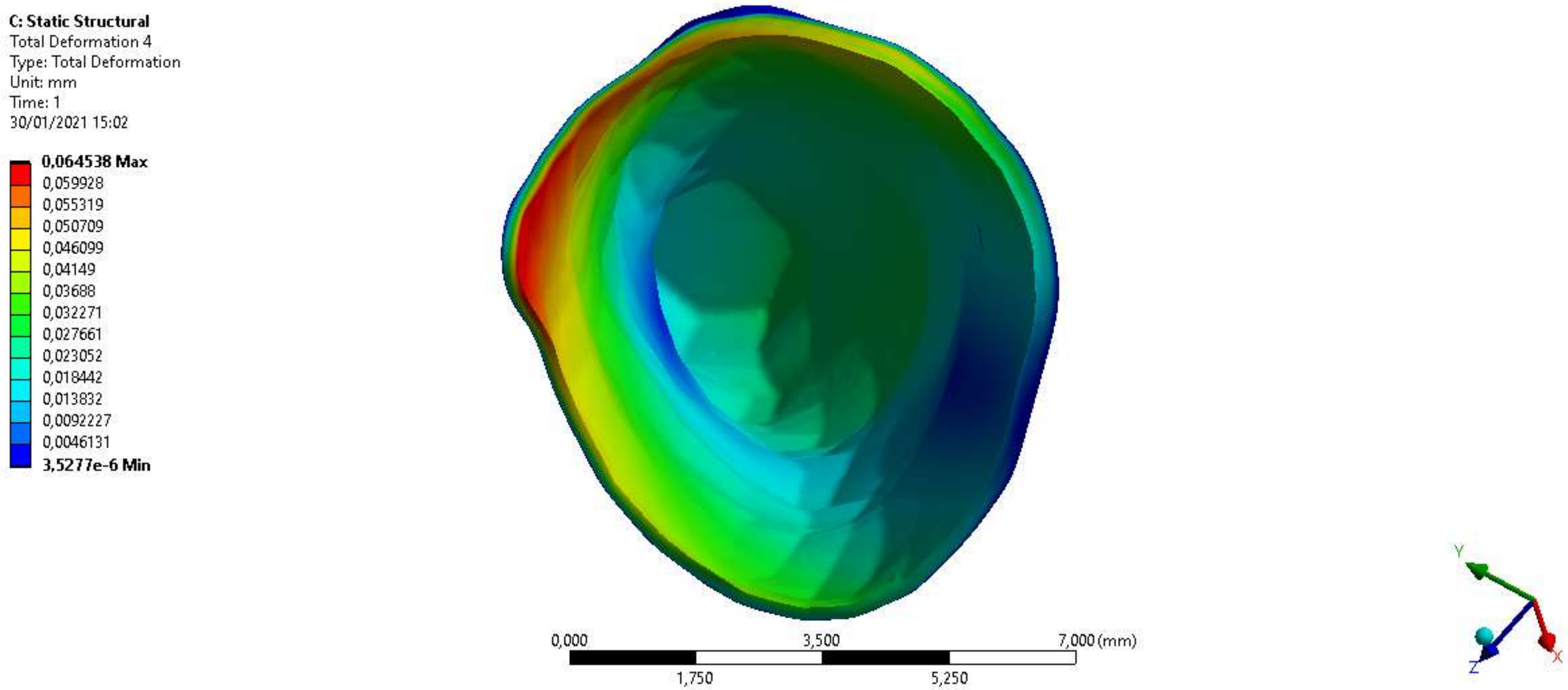
C: Static Structural
Total Deformation 4
Type: Total Deformation
Unit: mm
Time: 1
30/01/2021 15:02
0,064538 Max
0,059928
0,055319
0,050709
0,046099
0,04149
0,03688
0,032271
0,027661
0,023052
0,018442
0,013832
0,0092227
0,0046131
3,5277e-6 Min
0,000
3,500
7,000 (mm)
1,750
5,250
Y
Z
X

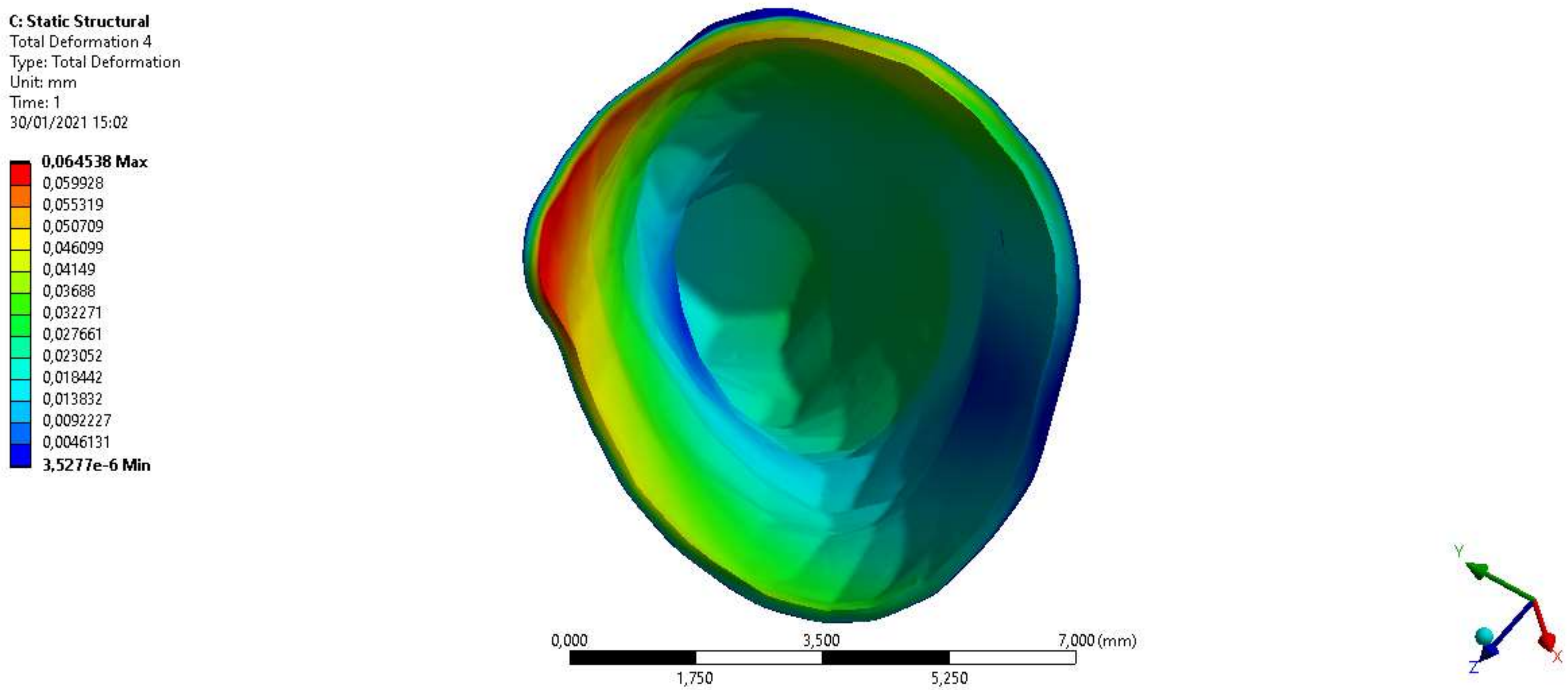
C: Static Structural
Total Deformation 4
Type: Total Deformation
Unit: mm
Time: 1
30/01/2021 15:02
0,064538 Max
0,059928
0,055319
0,050709
0,046099
0,04149
0,03688
0,032271
0,027661
0,023052
0,018442
0,013832
0,0092227
0,0046131
3,5277e-6 Min
0,000
3,500
7,000 (mm)
1,750
5,250
Y
Z
X

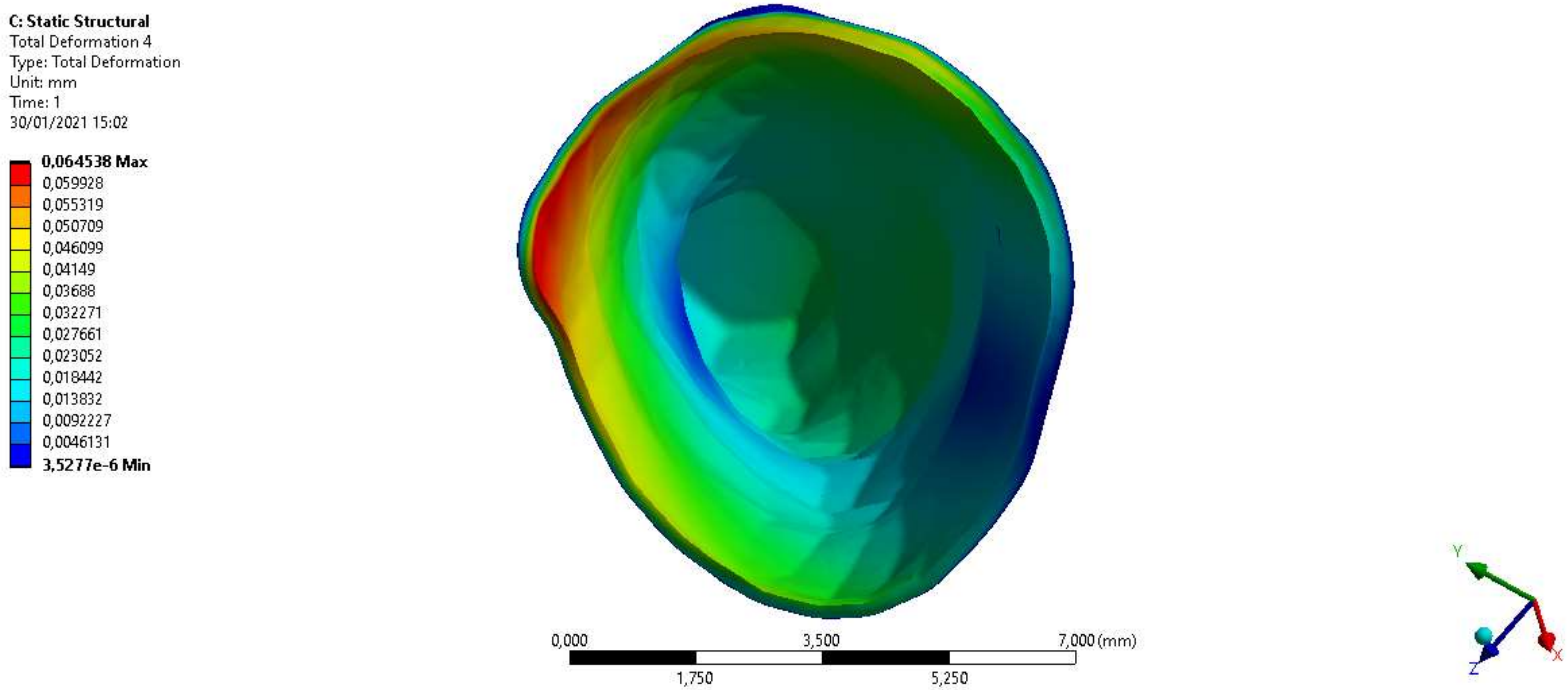
C: Static Structural
Total Deformation 4
Type: Total Deformation
Unit: mm
Time: 1
30/01/2021 15:02
0,064538 Max
0,059928
0,055319
0,050709
0,046099
0,04149
0,03688
0,032271
0,027661
0,023052
0,018442
0,013832
0,0092227
0,0046131
3,5277e-6 Min
0,000
3,500
7,000 (mm)
1,750
5,250
Y
X
Z

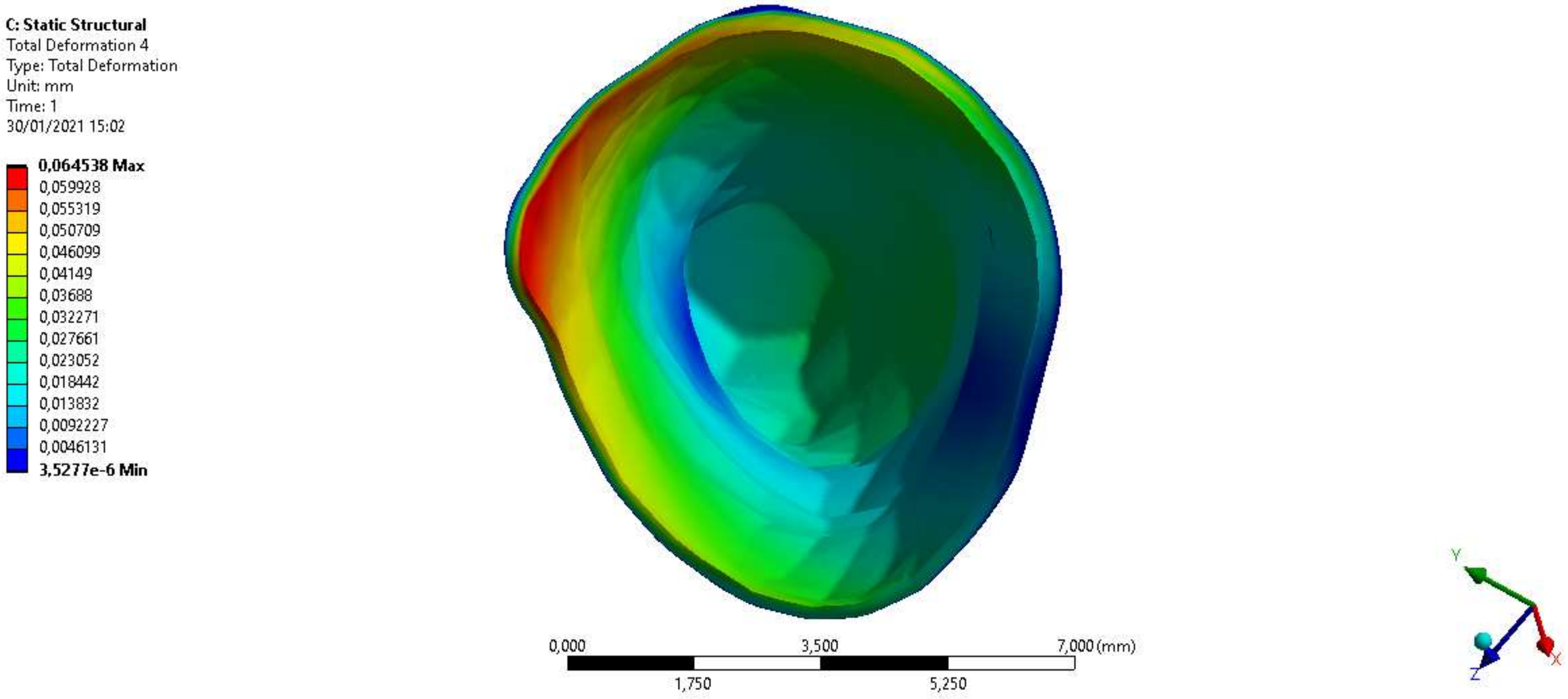
C: Static Structural
Total Deformation 4
Type: Total Deformation
Unit: mm
Time: 1
30/01/2021 15:02
0,064538 Max
0,059928
0,055319
0,050709
0,046099
0,04149
0,03688
0,032271
0,027661
0,023052
0,018442
0,013832
0,0092227
0,0046131
3,5277e-6 Min
0,000
3,500
7,000 (mm)
1,750
5,250
Y
Z
X

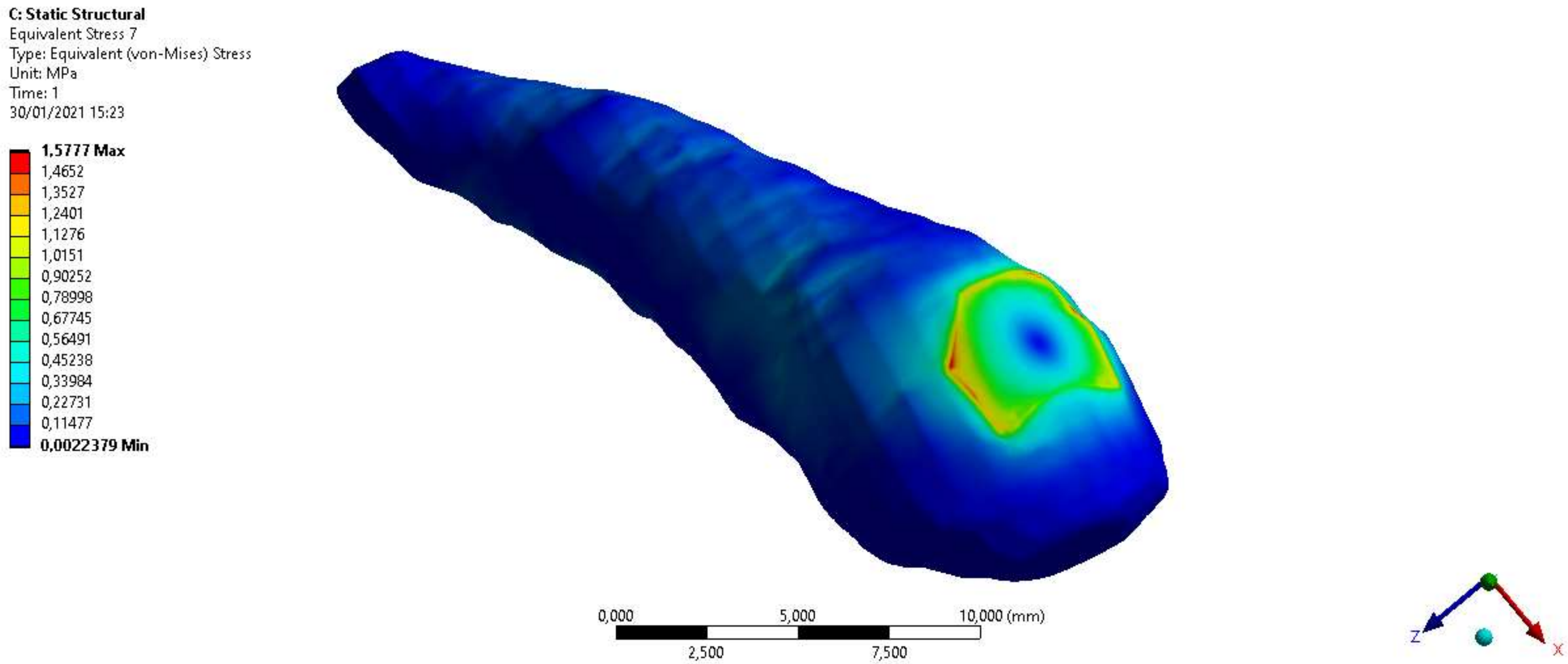
C: Static Structural
Equivalent Stress 7
Type: Equivalent (von-Mises) Stress
Unit: MPa
Time: 1
30/01/2021 15:23
1,5777 Max
1,4652
1,3527
1,2401
1,1276
1,0151
0,90252
0,78998
0,67745
0,56491
0,45238
0,33984
0,22731
0,11477
0,0022379 Min
0,000
5,000
10,000 (mm)
2,500
7,500
Z
X

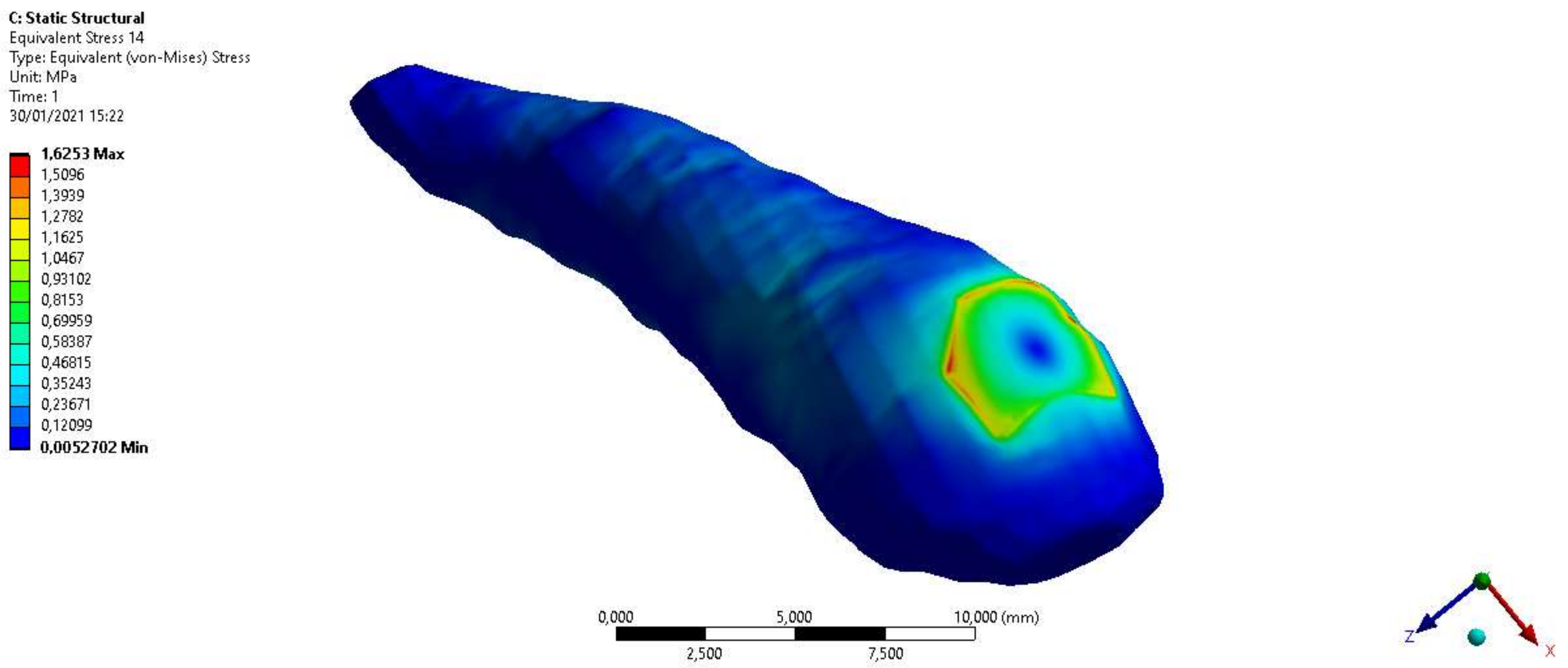
C: Static Structural
Equivalent Stress 14
Type: Equivalent (von-Mises) Stress
Unit: MPa
Time: 1
30/01/2021 15:22
1,6253 Max
1,5096
1,3939
1,2782
1,1625
1,0467
0,93102
0,8153
0,69959
0,58387
0,46815
0,35243
0,23671
0,12099
0,0052702 Min
0,000
5,000
10,000 (mm)
2,500
7,500
Z
X

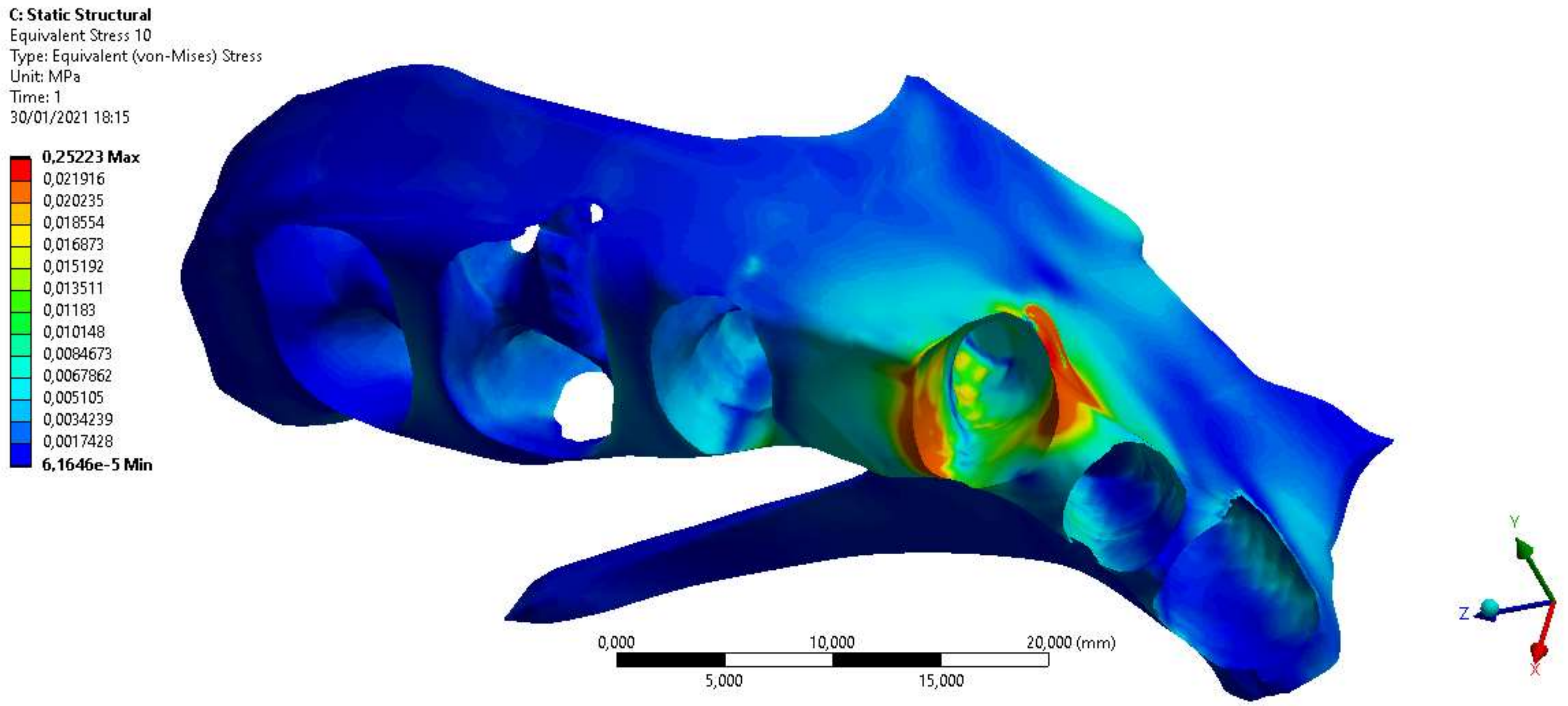
C: Static Structural
Equivalent Stress 10
Type: Equivalent (von-Mises) Stress
Unit: MPa
Time: 1
30/01/2021 18:15
0,25223 Max
0,021916
0,020235
0,018554
0,016873
0,015192
0,013511
0,01183
0,010148
0,0084673
0,0067862
0,005105
0,0034239
0,0017428
6,1646e-5 Min
0,000
10,000
20,000 (mm)
5,000
15,000
Y
Z
X

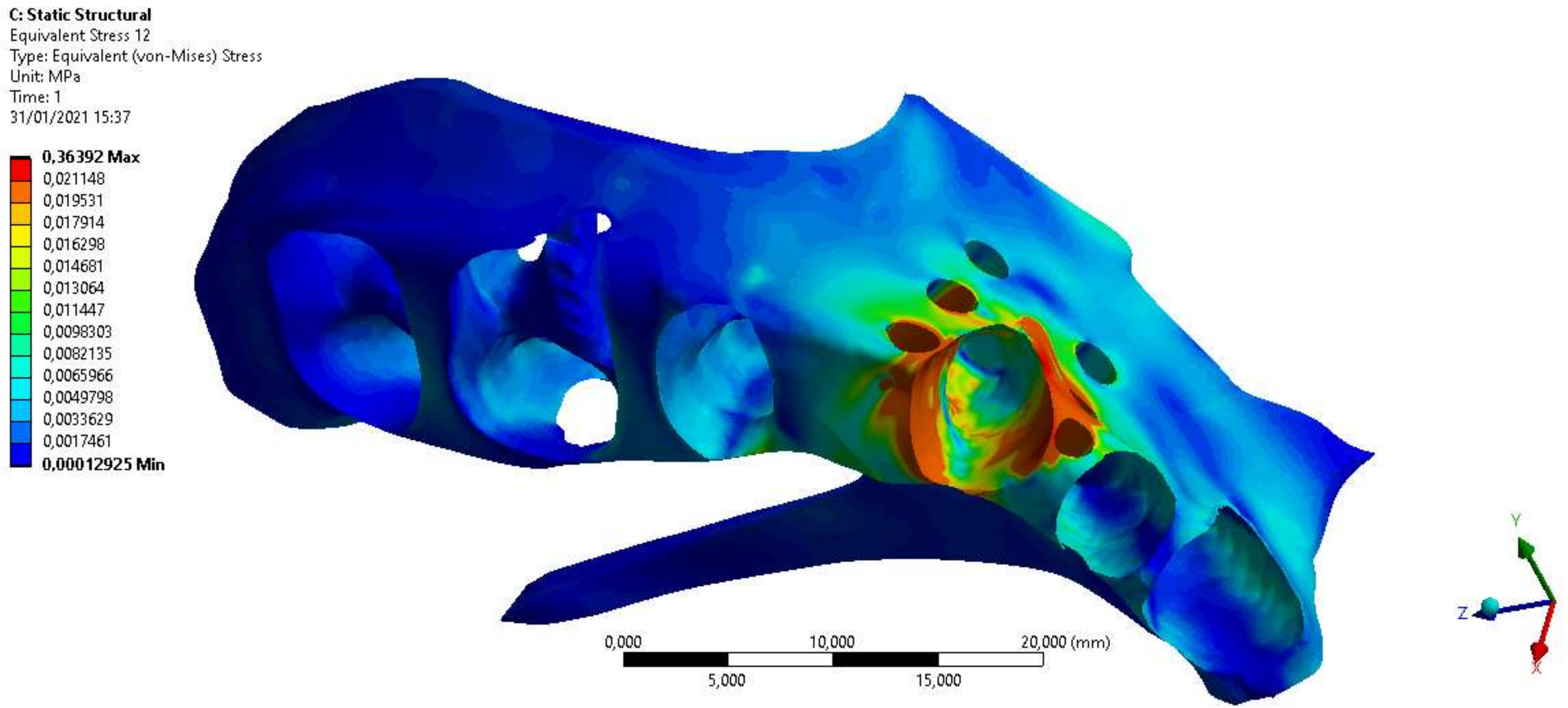

C: Static Structural
Equivalent Stress 12
Type: Equivalent (von-Mises) Stress
Unit: MPa
Time: 1
31/01/2021 15:37
0,36392 Max
0,021148
0,019531
0,017914
0,016298
0,014681
0,013064
0,011447
0,0098303
0,0082135
0,0065966
0,0049798
0,0033629
0,0017461
0,00012925 Min
0,000
10,000
20,000 (mm)
5,000
15,000
Y
Z
X

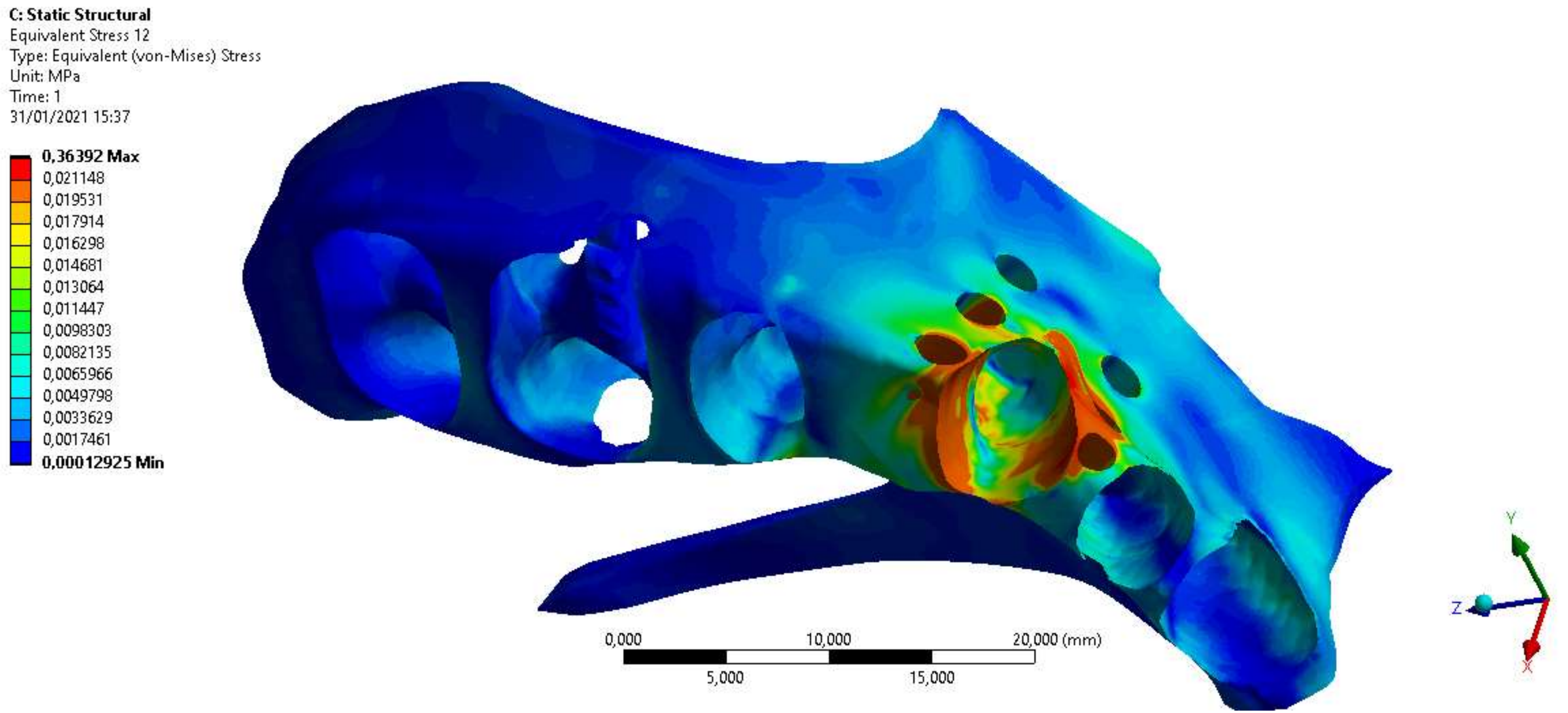
C: Static Structural
Equivalent Stress 12
Type: Equivalent (von-Mises) Stress
Unit: MPa
Time: 1
31/01/2021 15:37
0,36392 Max
0,021148
0,019531
0,017914
0,016298
0,014681
0,013064
0,011447
0,0098303
0,0082135
0,0065966
0,0049798
0,0033629
0,0017461
0,00012925 Min
0,000
10,000
20,000 (mm)
5,000
15,000
Y
Z
X

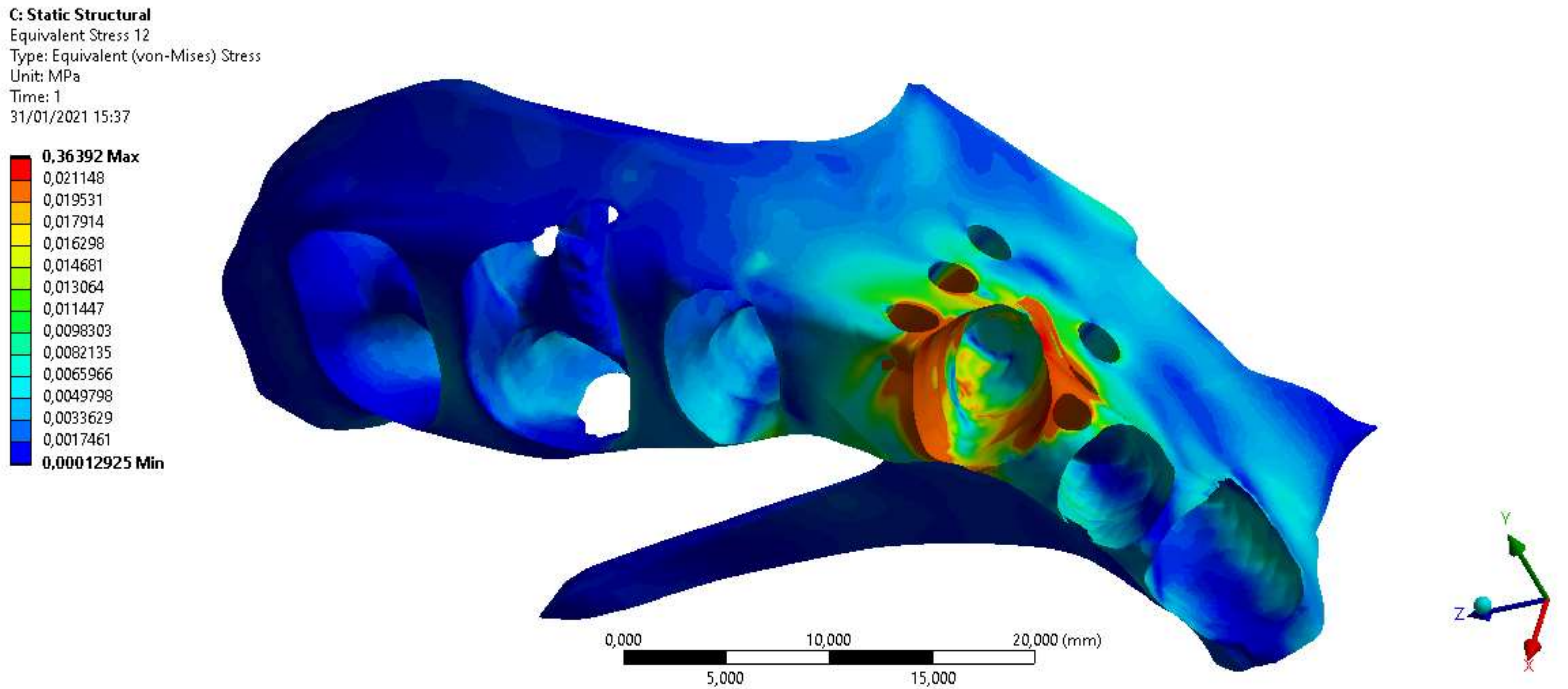
C: Static Structural
Equivalent Stress 12
Type: Equivalent (von-Mises) Stress
Unit: MPa
Time: 1
31/01/2021 15:37
0,36392 Max
0,021148
0,019531
0,017914
0,016298
0,014681
0,013064
0,011447
0,0098303
0,0082135
0,0065966
0,0049798
0,0033629
0,0017461
0,00012925 Min
0,000
10,000
20,000 (mm)
5,000
15,000
Y
Z
X

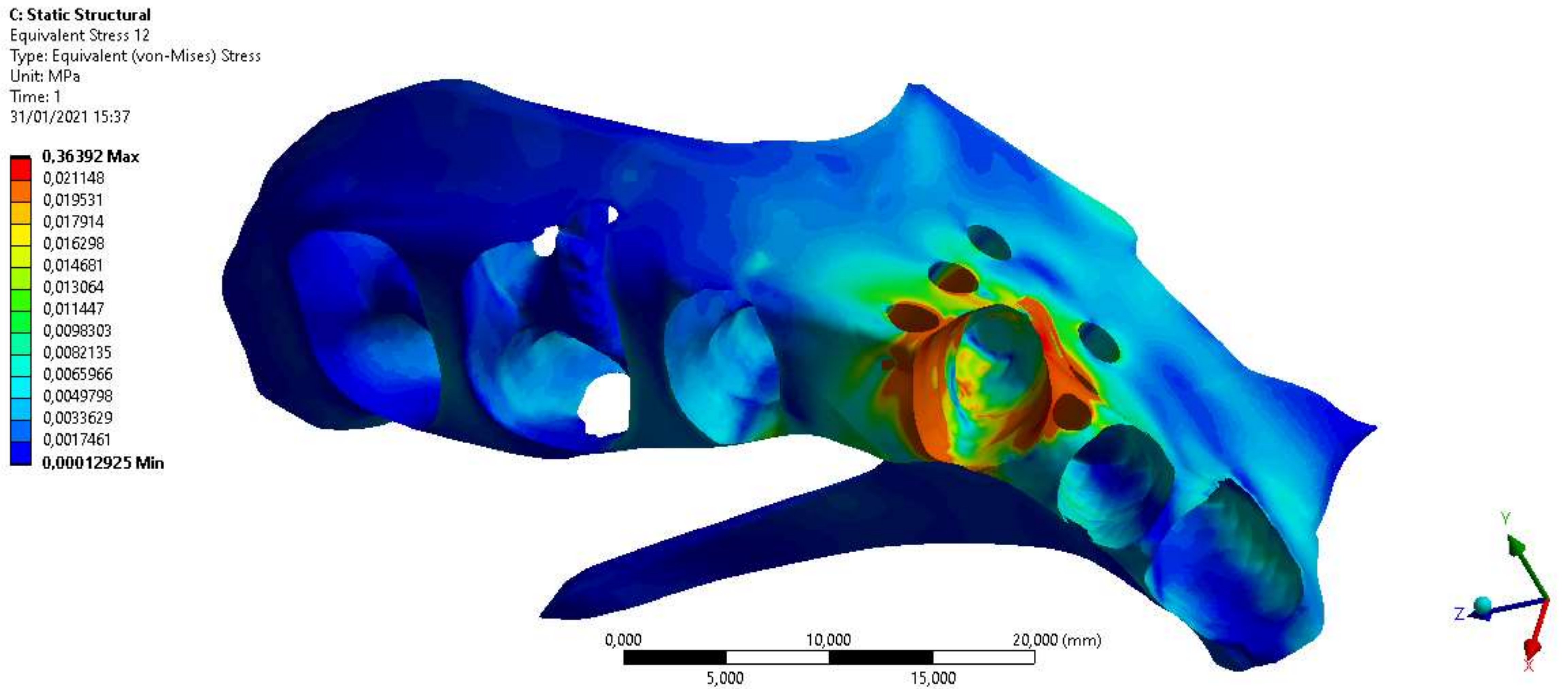
C: Static Structural
Equivalent Stress 12
Type: Equivalent (von-Mises) Stress
Unit: MPa
Time: 1
31/01/2021 15:37
0,36392 Max
0,021148
0,019531
0,017914
0,016298
0,014681
0,013064
0,011447
0,0098303
0,0082135
0,0065966
0,0049798
0,0033629
0,0017461
0,00012925 Min
0,000
10,000
20,000 (mm)
5,000
15,000
Y
Z
X

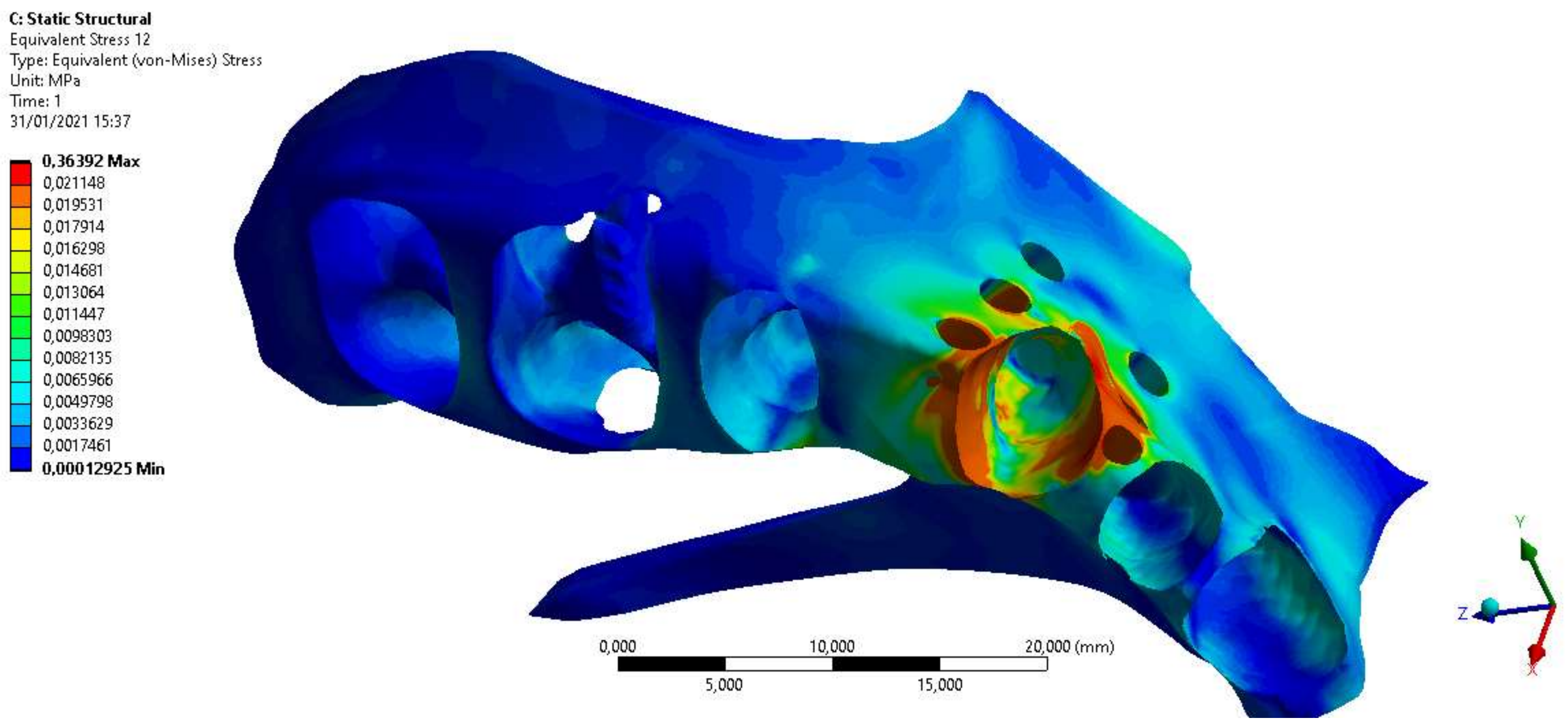

C: Static Structural
Equivalent Stress 12
Type: Equivalent (von-Mises) Stress
Unit: MPa
Time: 1
31/01/2021 15:37
0,36392 Max
0,021148
0,019531
0,017914
0,016298
0,014681
0,013064
0,011447
0,0098303
0,0082135
0,0065966
0,0049798
0,0033629
0,0017461
0,00012925 Min
0,000
10,000
20,000 (mm)
5,000
15,000
Y
Z
X

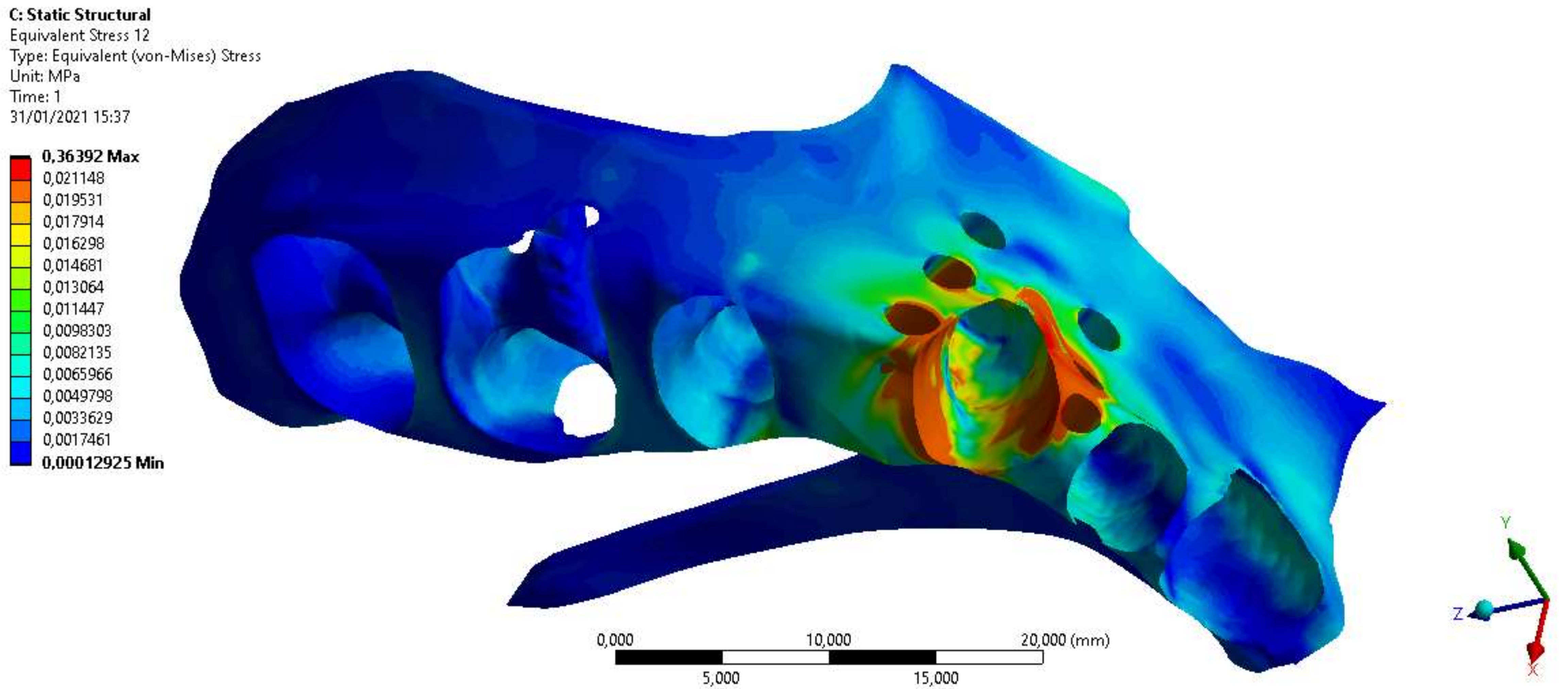
C: Static Structural
Equivalent Stress 12
Type: Equivalent (von-Mises) Stress
Unit: MPa
Time: 1
31/01/2021 15:37
0,36392 Max
0,021148
0,019531
0,017914
0,016298
0,014681
0,013064
0,011447
0,0098303
0,0082135
0,0065966
0,0049798
0,0033629
0,0017461
0,00012925 Min
0,000
10,000
20,000 (mm)
5,000
15,000
Y
Z
X

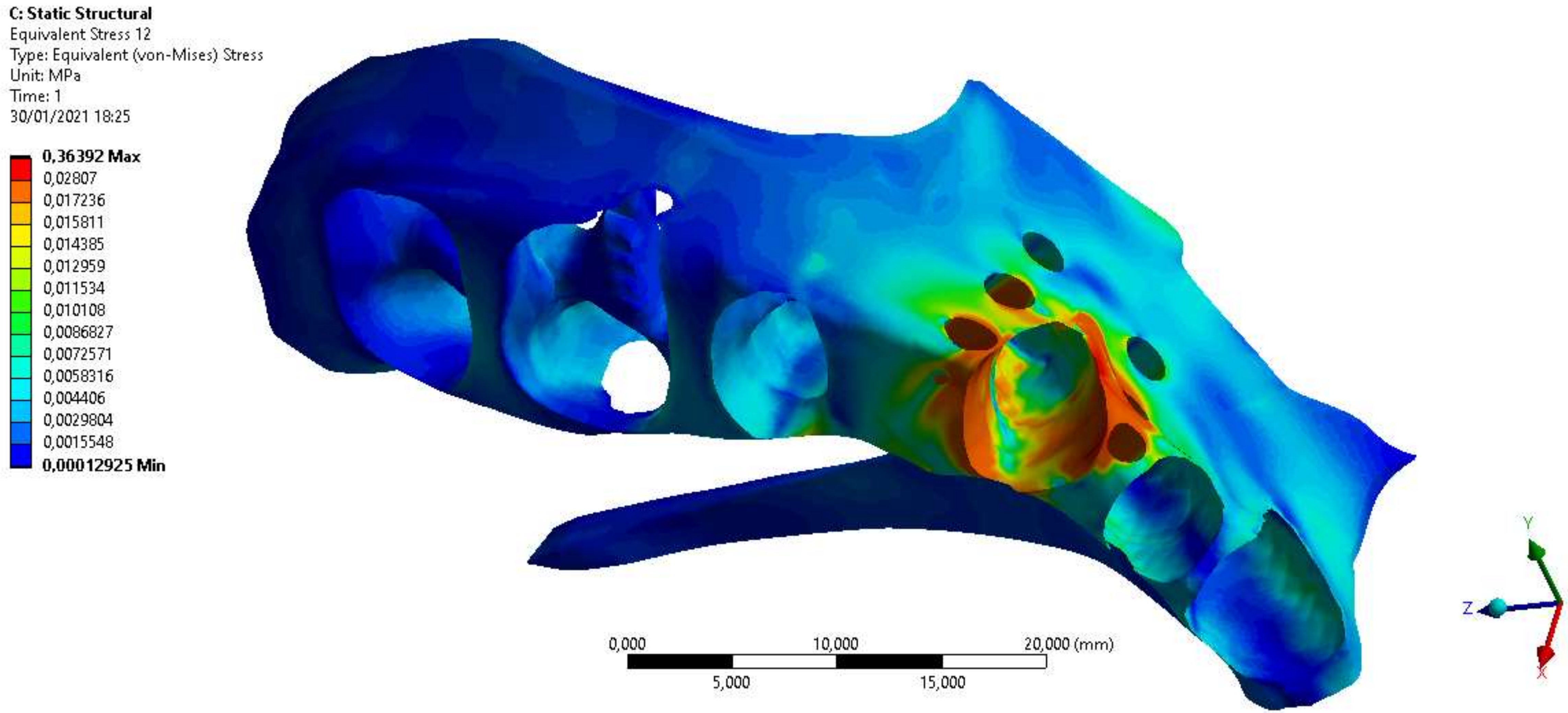
C: Static Structural
Equivalent Stress 12
Type: Equivalent (von-Mises) Stress
Unit: MPa
Time: 1
30/01/2021 18:25
0,36392 Max
0,02807
0,017236
0,015811
0,014385
0,012959
0,011534
0,010108
0,0086827
0,0072571
0,0058316
0,004406
0,0029804
0,0015548
0,00012925 Min
0,000
10,000
20,000 (mm)
5,000
15,000
Y
Z
X
