# Supplementary material for: Micro-osteoperforation for enhancement of orthodontic movement: A mechanical analysis using the finite element method
Source: PLoS One. 2024 Aug 19;19(8):e0308739. doi: 10.1371/journal.pone.0308739 (PMC11332926; doi:10.1371/journal.pone.0308739)
Supplement: S5 Fig — (PDF) [file pone.0308739.s013.pdf]

S13. Final images 2

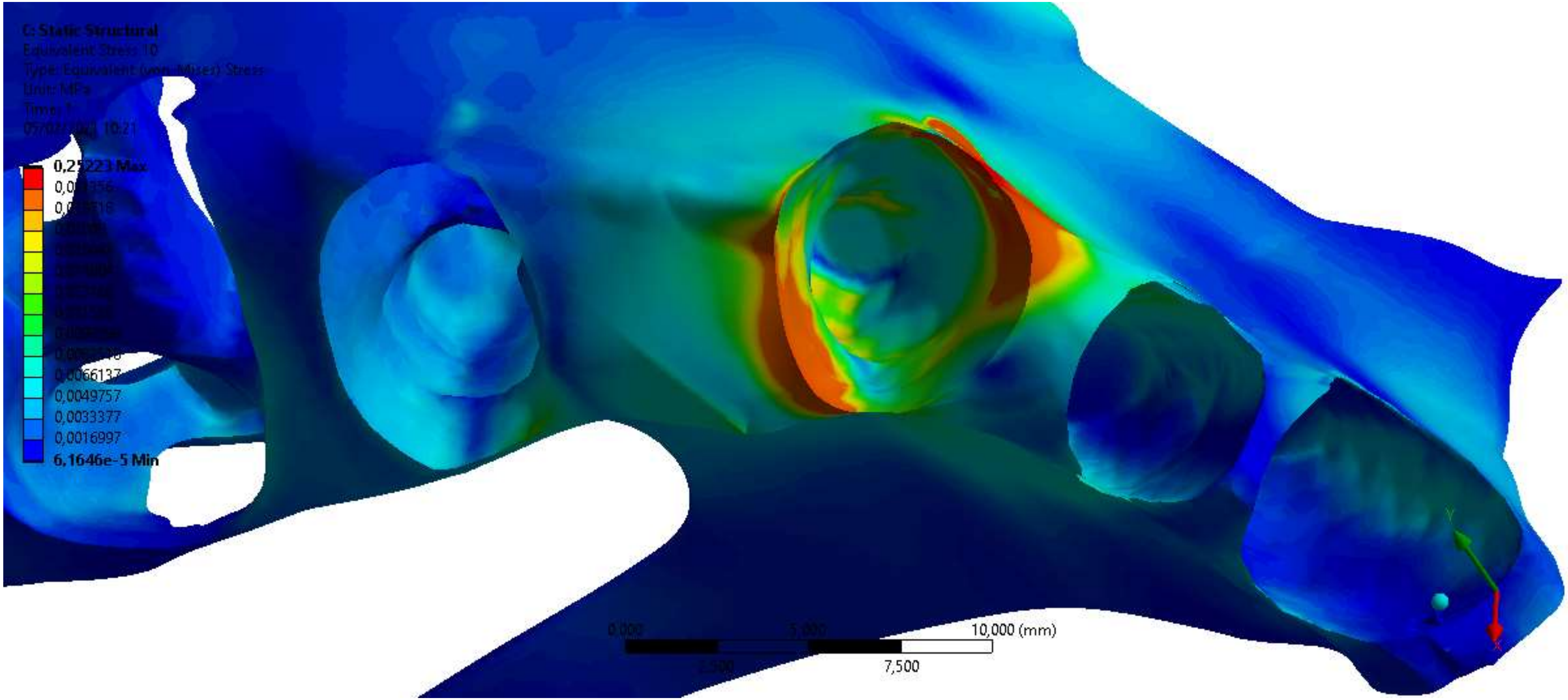
C: Static Structural
Equivalent Stress 10
Type: Equivalent (von-Mises) Stress
Unit: MPa
Time: 1
Max
0,0066137
0,0049757
0,0033377
0,0016997
6,1646e-5 Min
0,000
5,000
10,000 (mm)
2,500
7,500

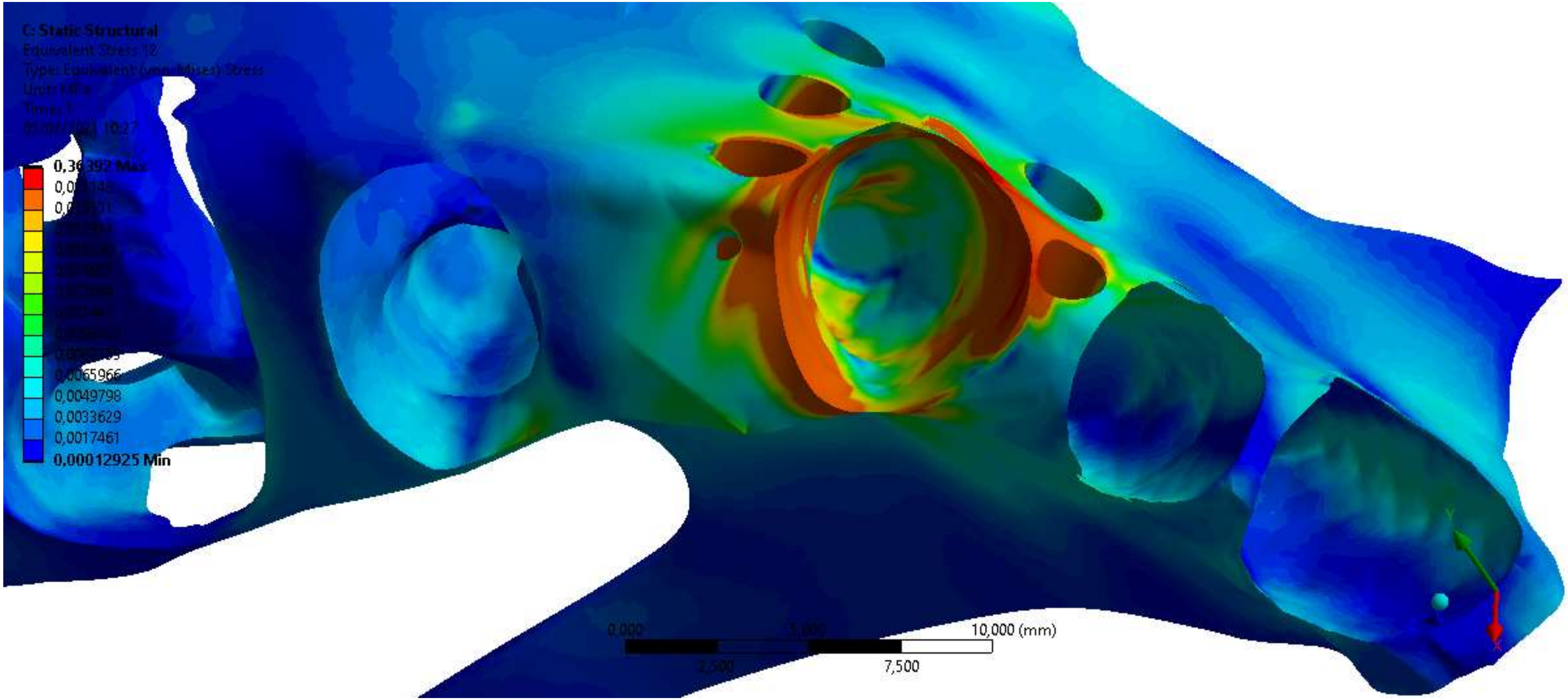
C: Static Structural
Equivalent Stress 12
Unit: MPa
Time: 1
0,36392 Max
0,0065966
0,0049798
0,0033629
0,0017461
0,00012925 Min
0,000
5,000
10,000 (mm)
2,500
7,500

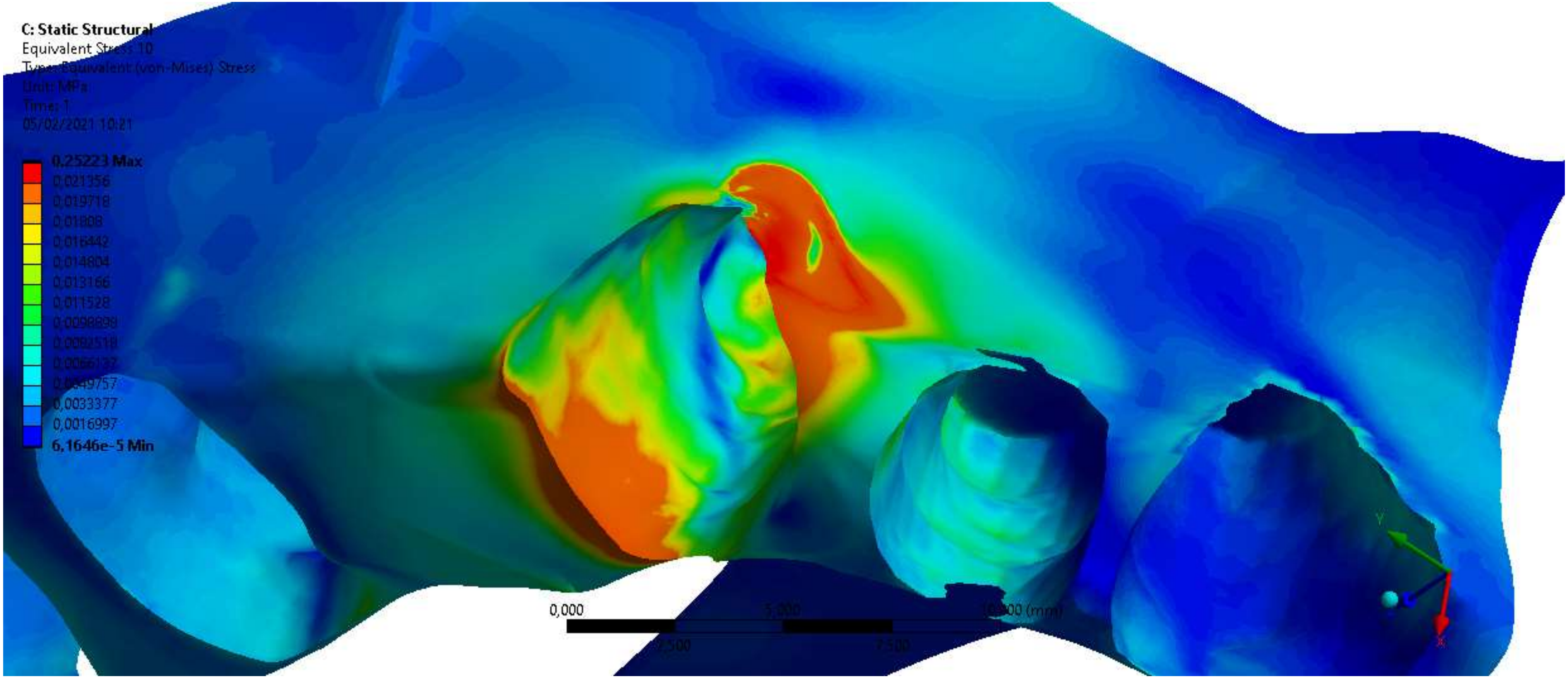
C: Static Structural
Equivalent Stress 10
Type: Equivalent (von-Mises) Stress
Unit: MPa
Time: 1
05/02/2021 10:21
0,25223 Max
0,021356
0,019718
0,01808
0,016442
0,014804
0,013166
0,011528
0,0098898
0,0082518
0,0066137
0,0049757
0,0033377
0,0016997
6,1646e-5 Min
0,000
5,000
10,000 (mm)
2,500
7,500

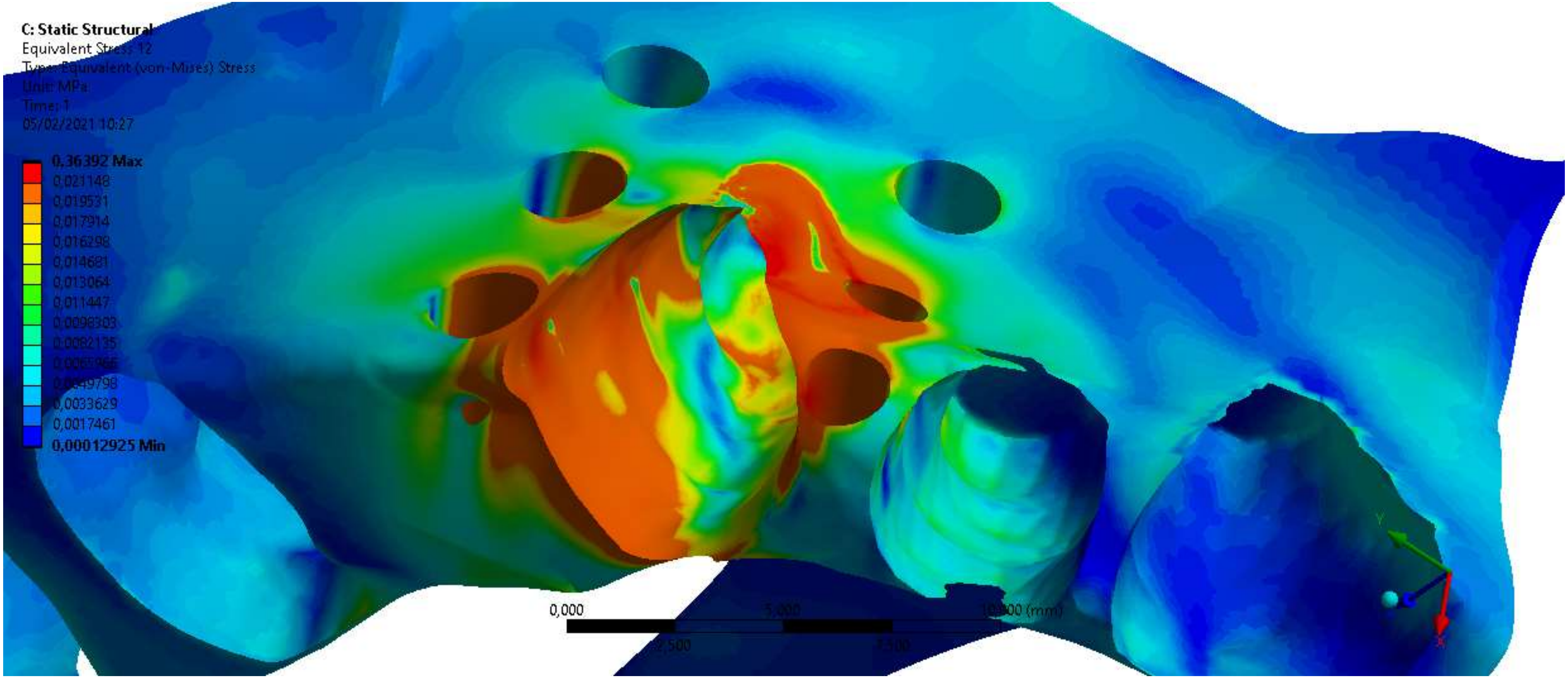
C: Static Structural
Equivalent Stress 12
Type: Equivalent (von-Mises) Stress
Unit: MPa
Time: 1
05/02/2021 10:27
0,36392 Max
0,021148
0,019531
0,017914
0,016298
0,014681
0,013064
0,011447
0,0098303
0,0082135
0,0065966
0,0049798
0,0033629
0,0017461
0,00012925 Min
0,000
5,000
10,000 (mm)
2,500
7,500

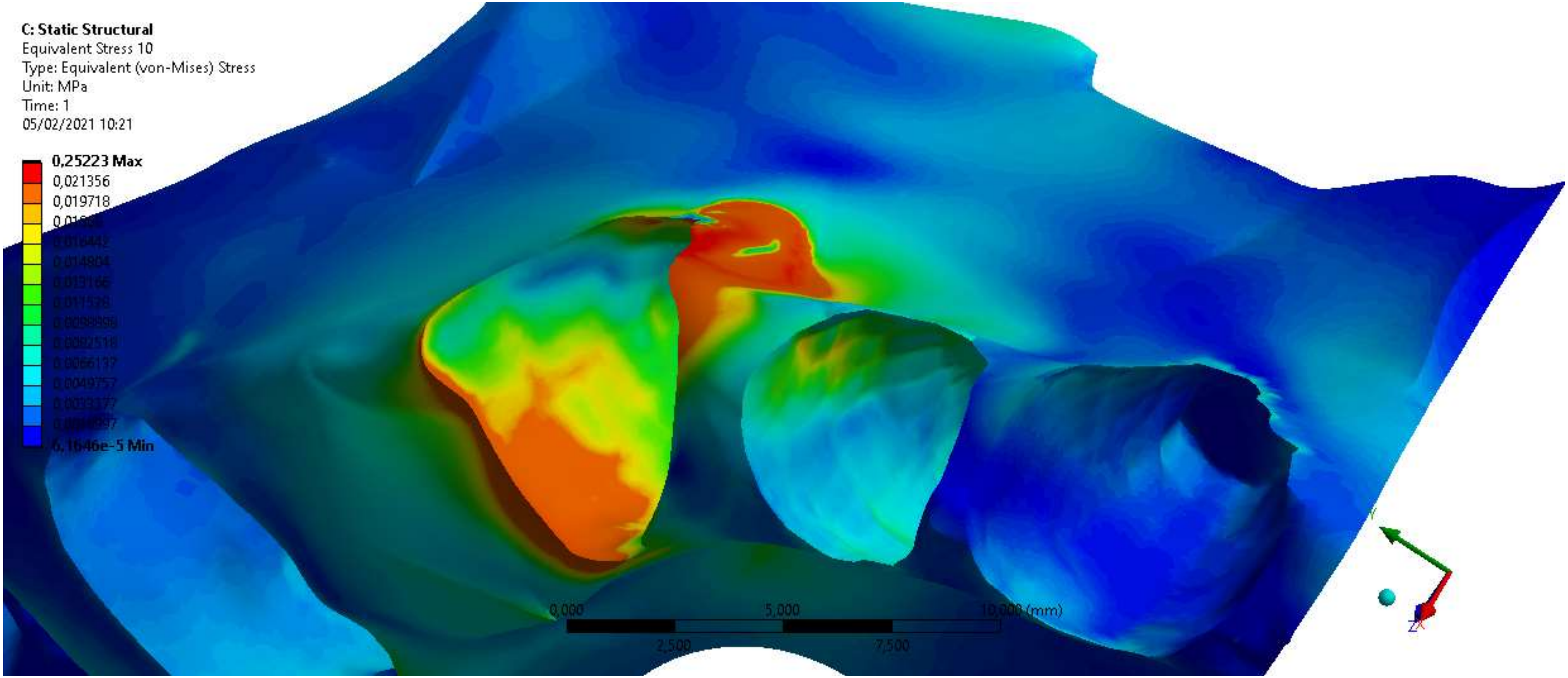
C: Static Structural
Equivalent Stress 10
Type: Equivalent (von-Mises) Stress
Unit: MPa
Time: 1
05/02/2021 10:21
0,25223 Max
0,021356
0,019718
6,1646e-5 Min
0,000
5,000
10,000 (mm)
2,500
7,500

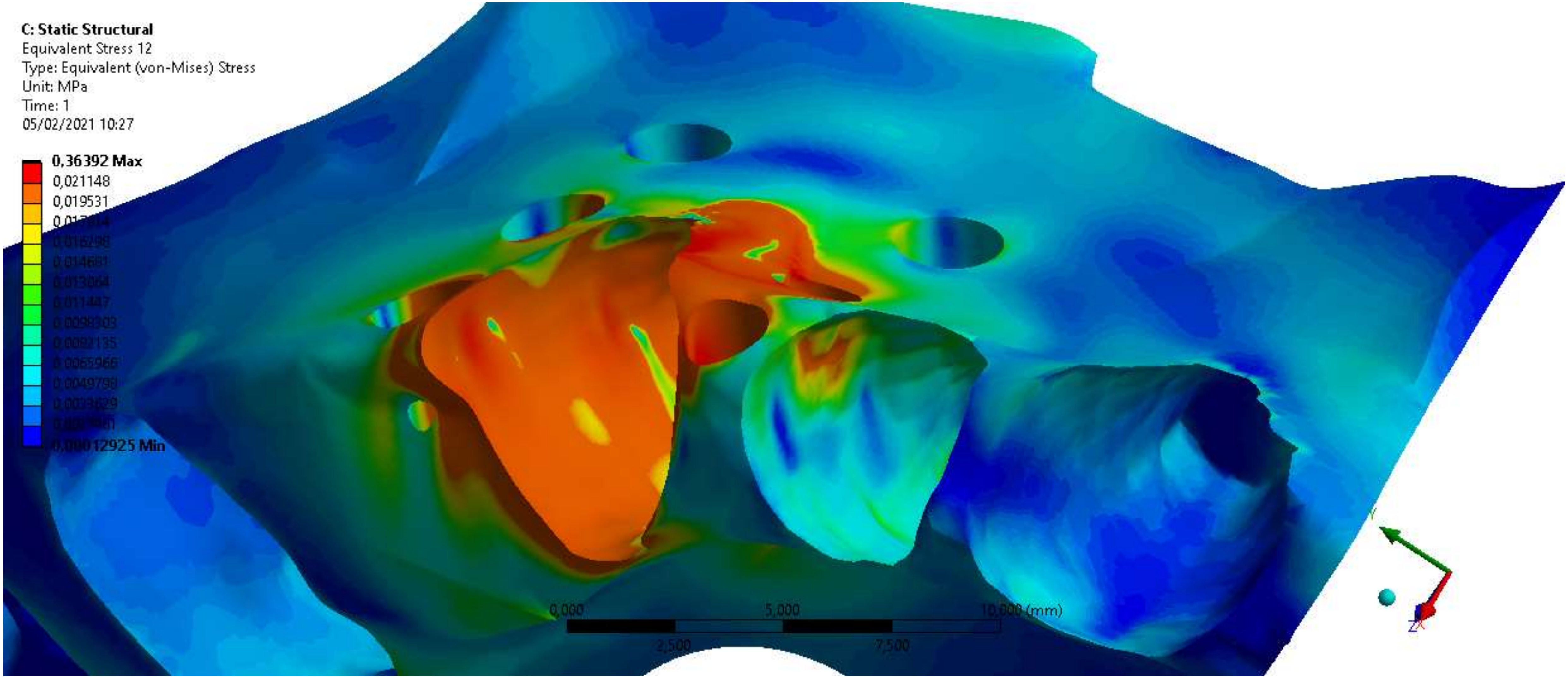
C: Static Structural
Equivalent Stress 12
Type: Equivalent (von-Mises) Stress
Unit: MPa
Time: 1
05/02/2021 10:27
0,36392 Max
0,021148
0,019531
0,00012925 Min
0,000
5,000
10,000 (mm)
2,500
7,500

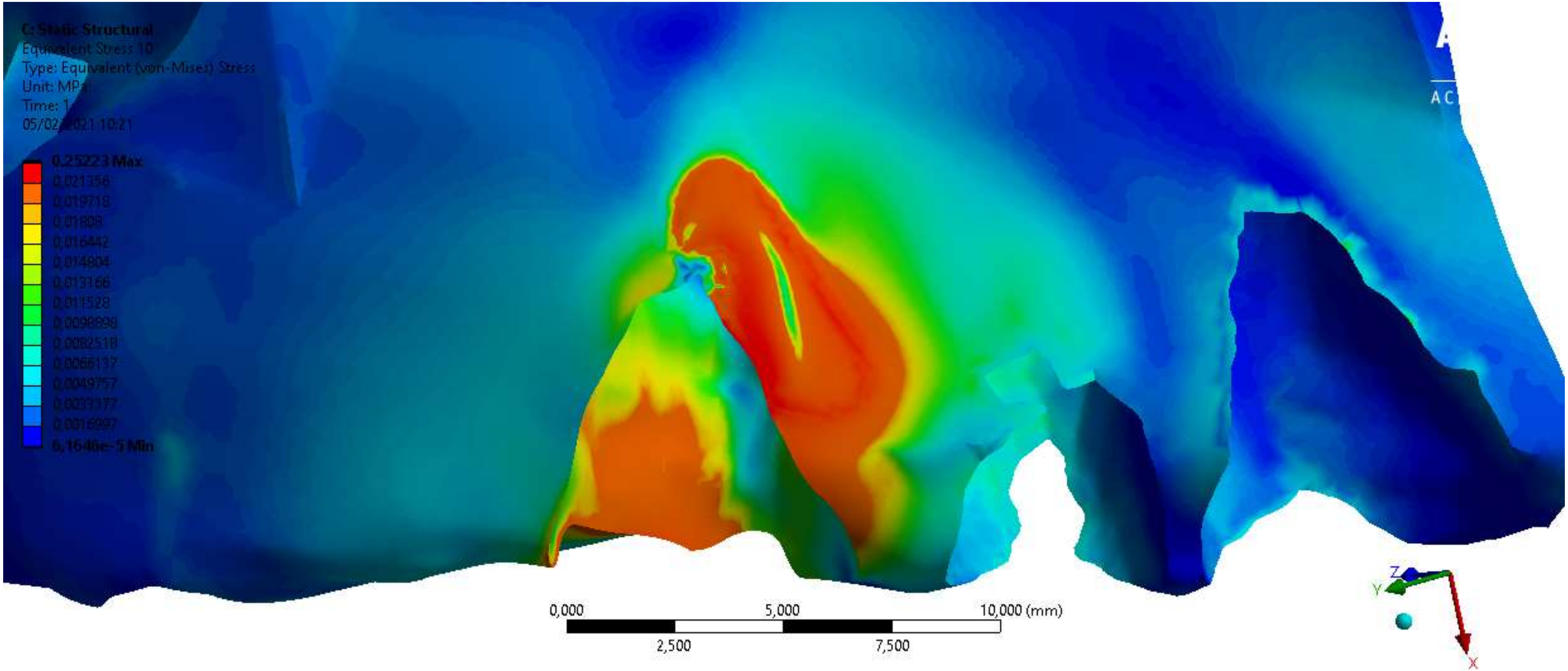
C: Static Structural
Equivalent Stress 10
Type: Equivalent (von-Mises) Stress
Unit: MPa
Time: 1
05/02/2021 10:21
0,25223 Max
0,021356
0,019718
0,01808
0,016442
0,014804
0,013166
0,011528
0,0098898
0,0082518
0,0066137
0,0049757
0,0033377
0,0016997
6,1646e-5 Min
0,000
5,000
10,000 (mm)
2,500
7,500
Z
Y
X

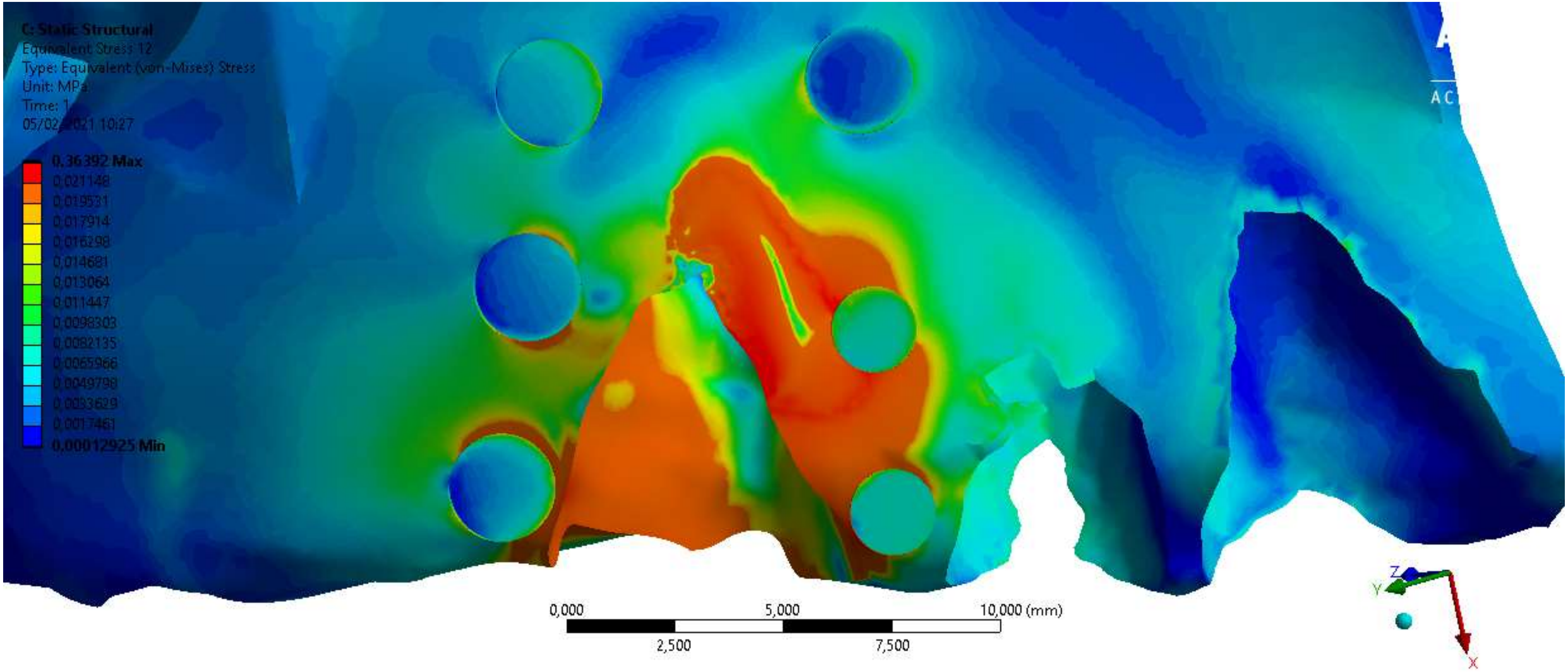
C: Static Structural
Equivalent Stress 12
Type: Equivalent (von-Mises) Stress
Unit: MPa
Time: 1
05/02/2021 10:27
0,36392 Max
0,021148
0,019531
0,017914
0,016298
0,014681
0,013064
0,011447
0,0098303
0,0082135
0,0065966
0,0049798
0,0033629
0,0017461
0,00012925 Min
0,000
5,000
10,000 (mm)
2,500
7,500
Z
Y
X

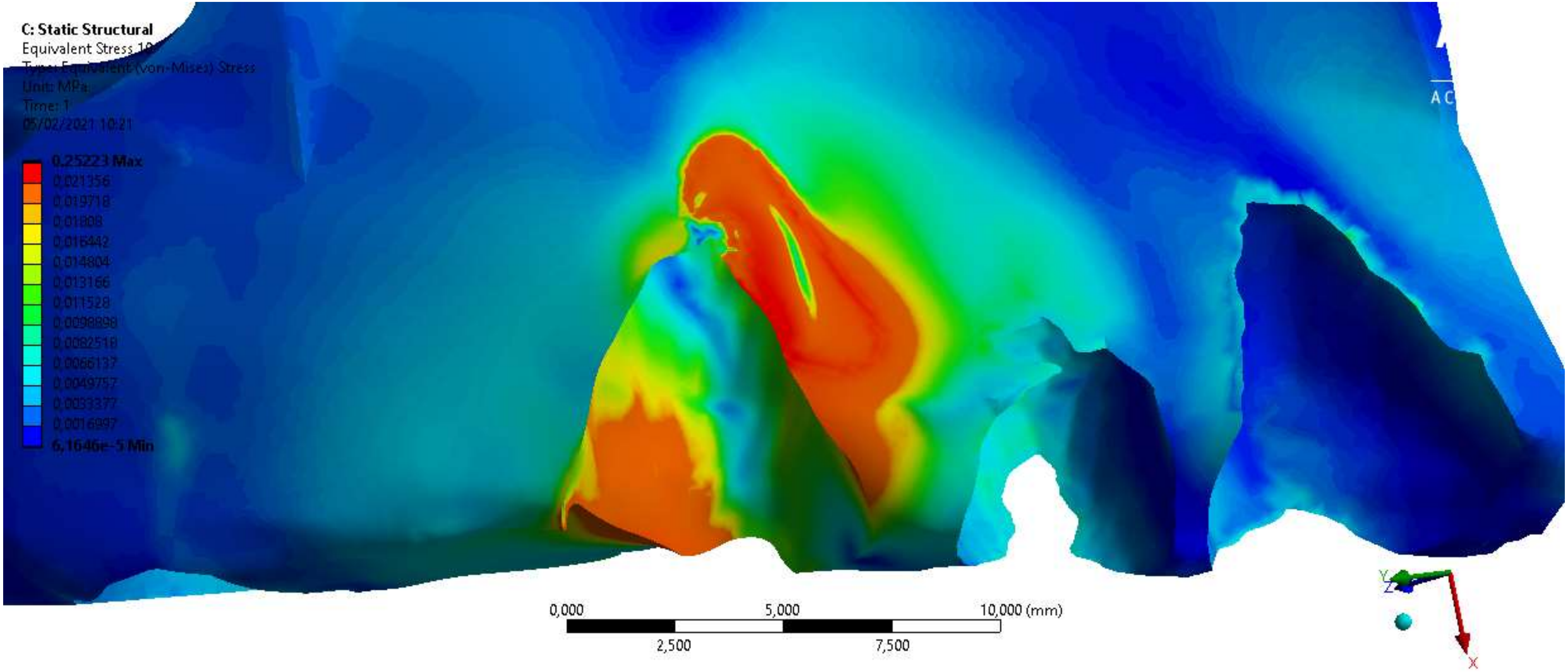
C: Static Structural
Equivalent Stress
Type: Equivalent (von-Mises) Stress
Unit: MPa
Time: 1
05/02/2021 10:21
0,25223 Max
0,021356
0,019718
0,01808
0,016442
0,014804
0,013166
0,011528
0,0098898
0,0082518
0,0066137
0,0049757
0,0033377
0,0016997
6,1646e-5 Min
0,000
5,000
10,000 (mm)
2,500
7,500
Y
Z
X

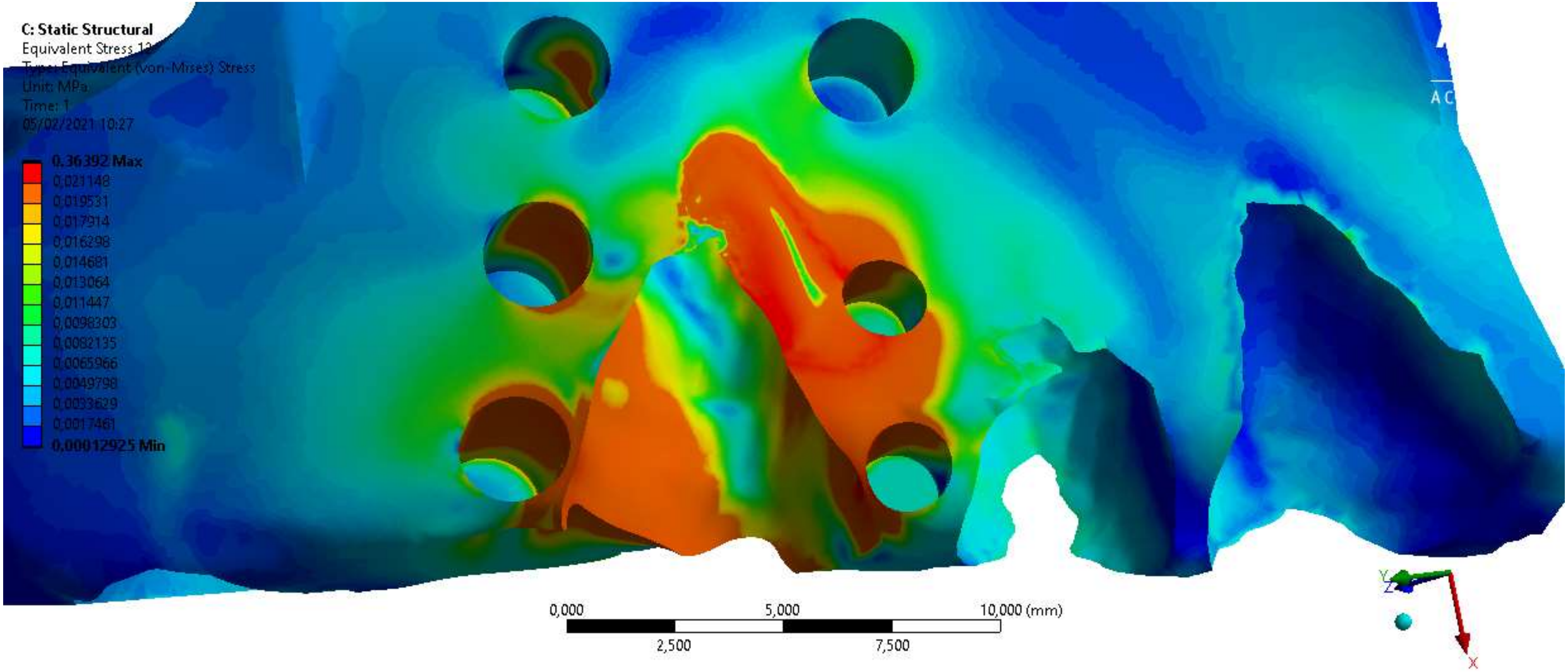
C: Static Structural
Equivalent Stress 12
Type: Equivalent (von-Mises) Stress
Unit: MPa
Time: 1
05/02/2021 10:27
0,36392 Max
0,021148
0,019531
0,017914
0,016298
0,014681
0,013064
0,011447
0,0098303
0,0082135
0,0065966
0,0049798
0,0033629
0,0017461
0,00012925 Min
0,000
5,000
10,000 (mm)
2,500
7,500
Y
Z
X

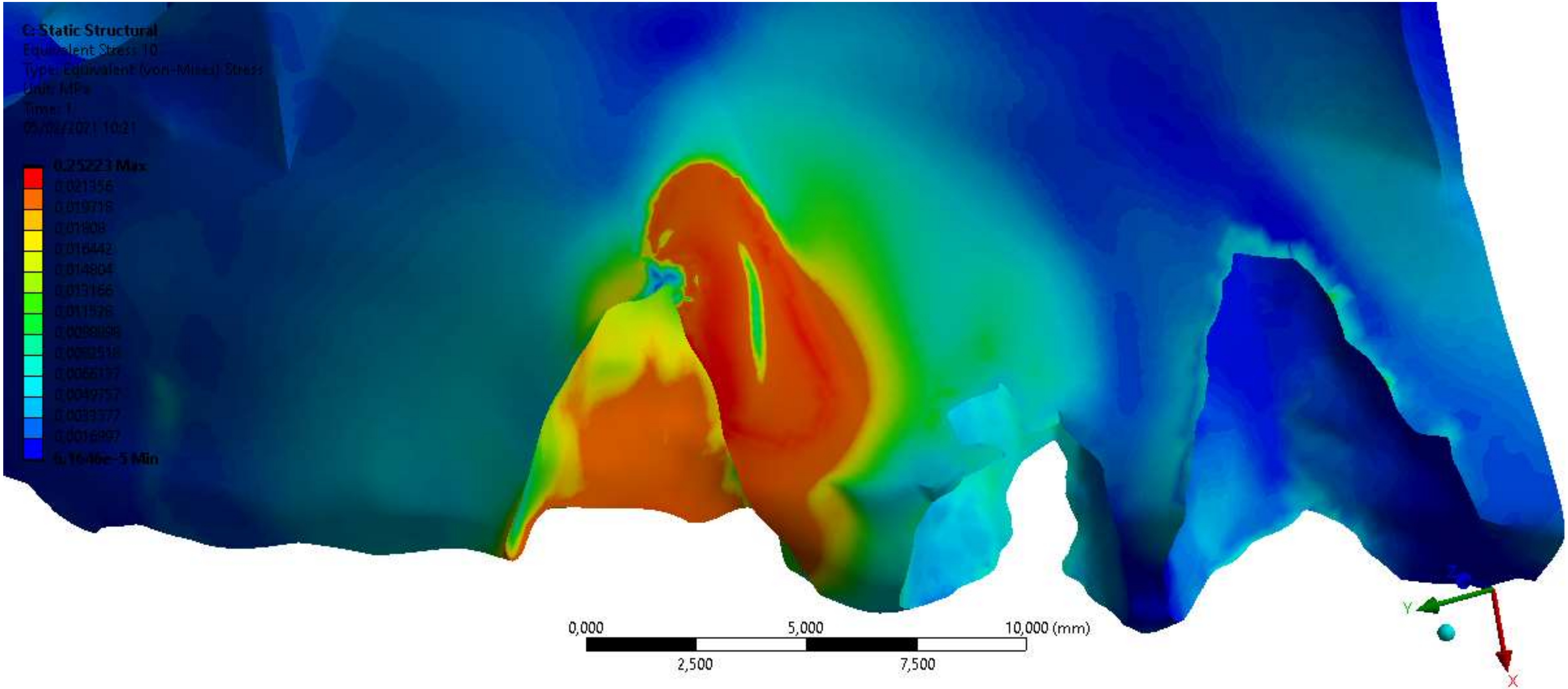
C: Static Structural
Equivalent Stress 10
Type: Equivalent (von-Mises) Stress
Unit: MPa
Time: 1
0,25223 Max
0,021356
0,019718
0,01808
0,016442
0,014804
0,013166
0,0098898
0,0082518
0,0066137
0,0049757
0,0033377
0,0016997
6,1646e-5 Min
0,000
5,000
10,000 (mm)
2,500
7,500
Y
X

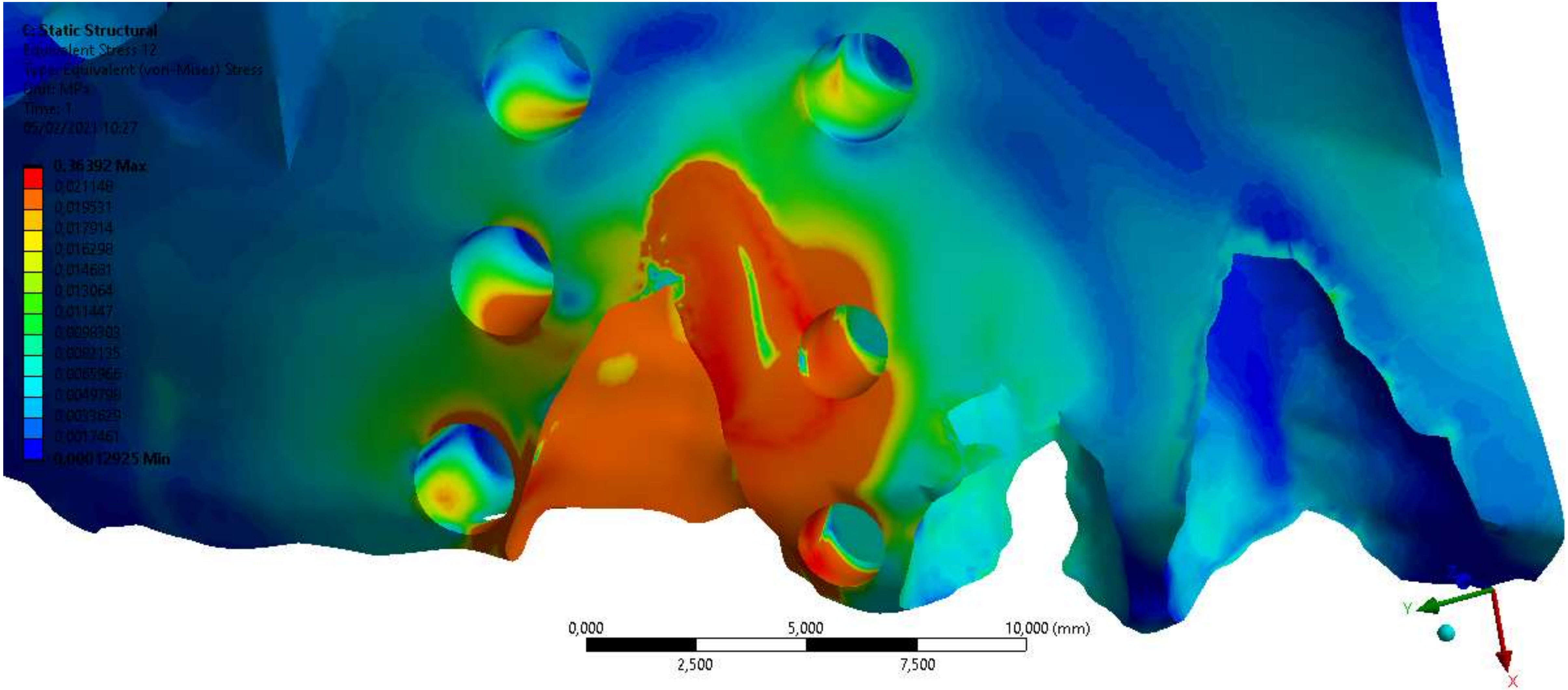

C: Static Structural
Equivalent Stress 12
Type: Equivalent (von-Mises) Stress
Unit: MPa
Time: 1
05/02/2021 10:27
0,36392 Max
0,021148
0,019531
0,017914
0,016298
0,014681
0,013064
0,011447
0,0098303
0,0082135
0,0065966
0,0049798
0,0033629
0,0017461
0,00012925 Min
0,000
5,000
10,000 (mm)
2,500
7,500
Y
X
