# Supplementary material for: Micro-osteoperforation for enhancement of orthodontic movement: A mechanical analysis using the finite element method
Source: PLoS One. 2024 Aug 19;19(8):e0308739. doi: 10.1371/journal.pone.0308739 (PMC11332926; doi:10.1371/journal.pone.0308739)
Supplement: S6 Fig — (PDF) [file pone.0308739.s014.pdf]

S14. Final images 3

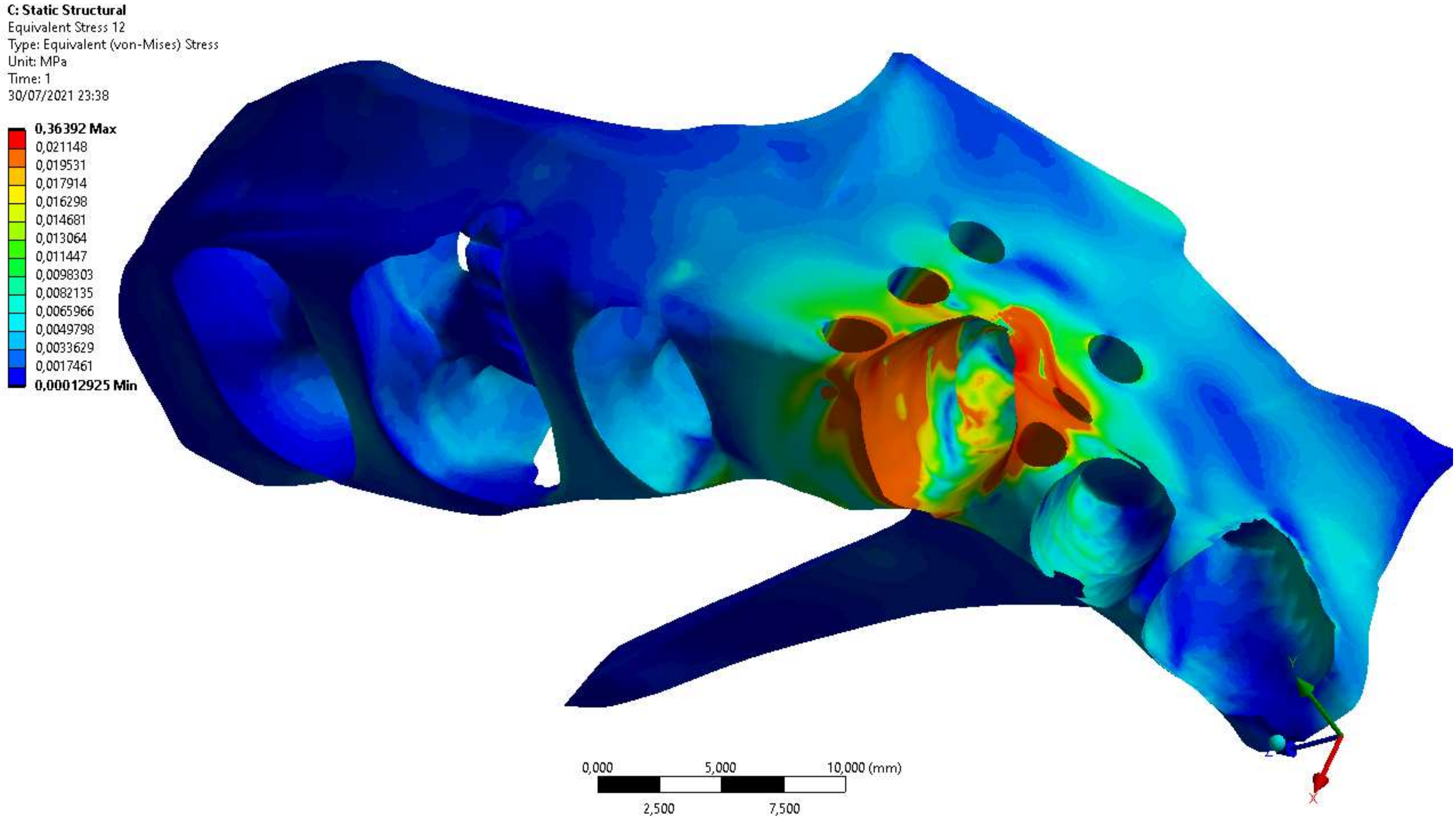
C: Static Structural
Equivalent Stress 12
Type: Equivalent (von-Mises) Stress
Unit: MPa
Time: 1
30/07/2021 23:38
0,36392 Max
0,021148
0,019531
0,017914
0,016298
0,014681
0,013064
0,011447
0,0098303
0,0082135
0,0065966
0,0049798
0,0033629
0,0017461
0,00012925 Min
0,000
5,000
10,000 (mm)
2,500
7,500
Y
Z
X

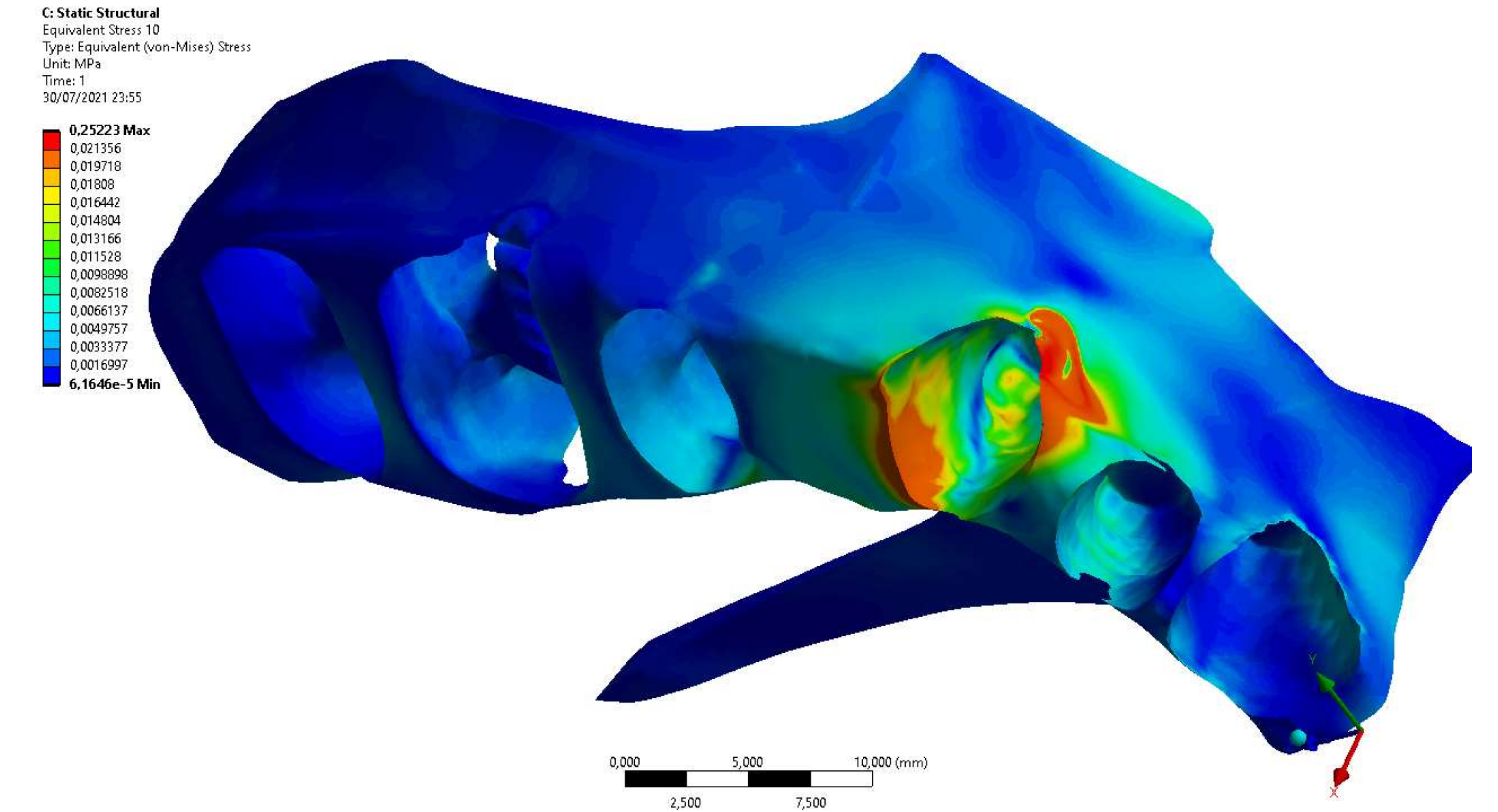
C: Static Structural
Equivalent Stress 10
Type: Equivalent (von-Mises) Stress
Unit: MPa
Time: 1
30/07/2021 23:55
0,25223 Max
0,021356
0,019718
0,01808
0,016442
0,014804
0,013166
0,011528
0,0098898
0,0082518
0,0066137
0,0049757
0,0033377
0,0016997
6,1646e-5 Min
0,000
5,000
10,000 (mm)
2,500
7,500
Y
X

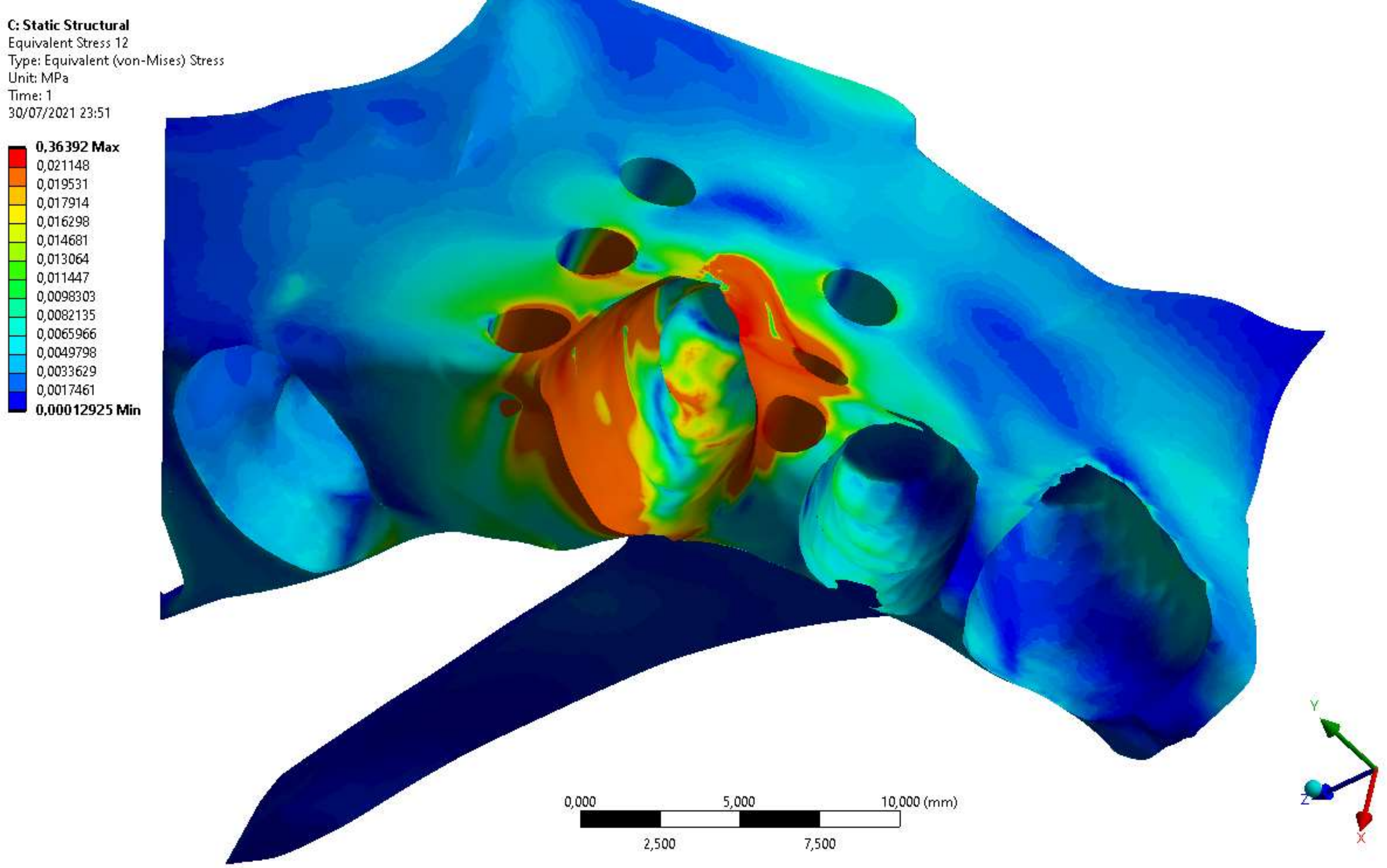
C: Static Structural
Equivalent Stress 12
Type: Equivalent (von-Mises) Stress
Unit: MPa
Time: 1
30/07/2021 23:51
0,36392 Max
0,021148
0,019531
0,017914
0,016298
0,014681
0,013064
0,011447
0,0098303
0,0082135
0,0065966
0,0049798
0,0033629
0,0017461
0,00012925 Min
0,000
5,000
10,000 (mm)
2,500
7,500
Y
Z
X

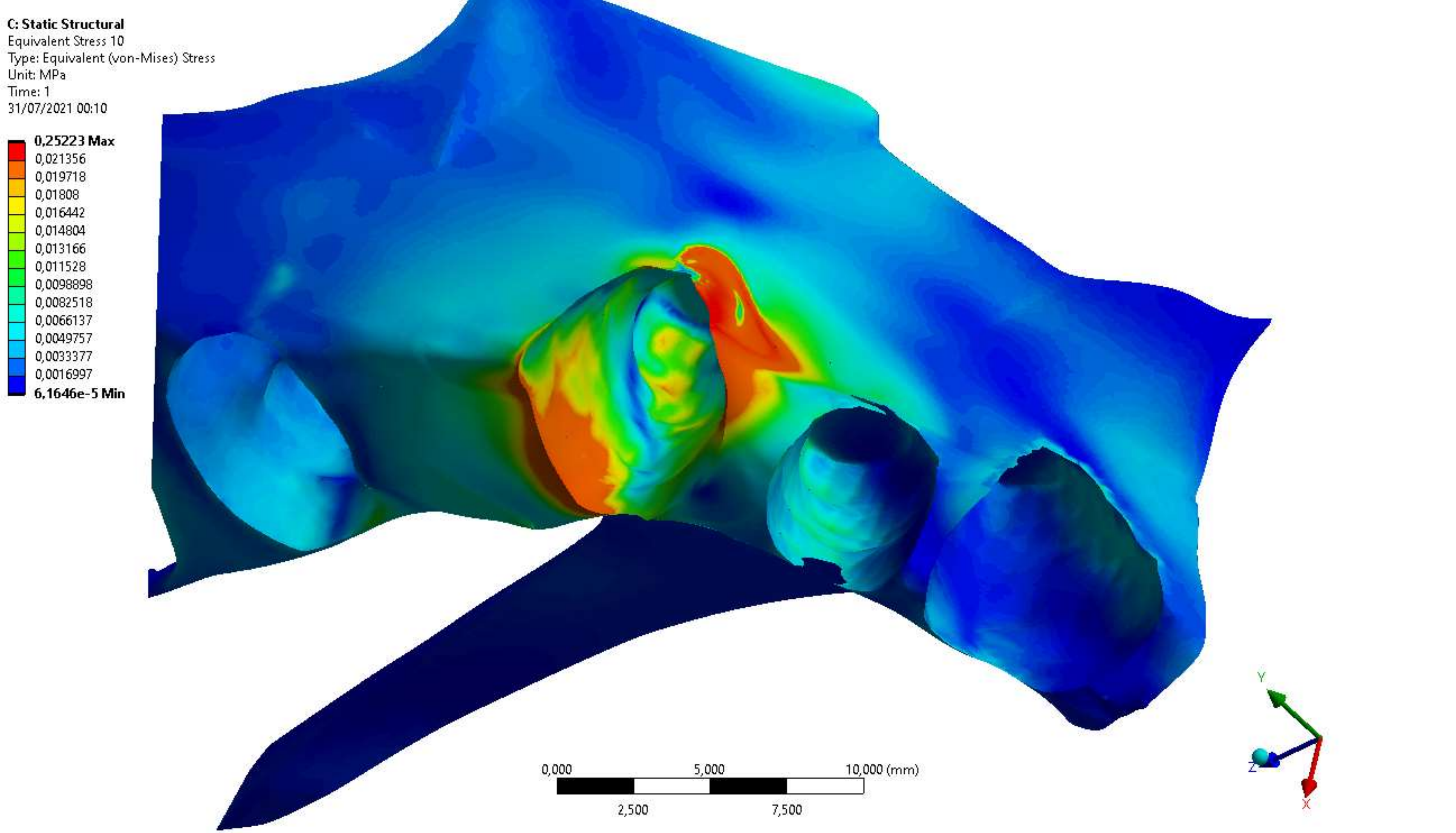
C: Static Structural
Equivalent Stress 10
Type: Equivalent (von-Mises) Stress
Unit: MPa
Time: 1
31/07/2021 00:10
0,25223 Max
0,021356
0,019718
0,01808
0,016442
0,014804
0,013166
0,011528
0,0098898
0,0082518
0,0066137
0,0049757
0,0033377
0,0016997
6,1646e-5 Min
0,000
5,000
10,000 (mm)
2,500
7,500
Y
Z
X

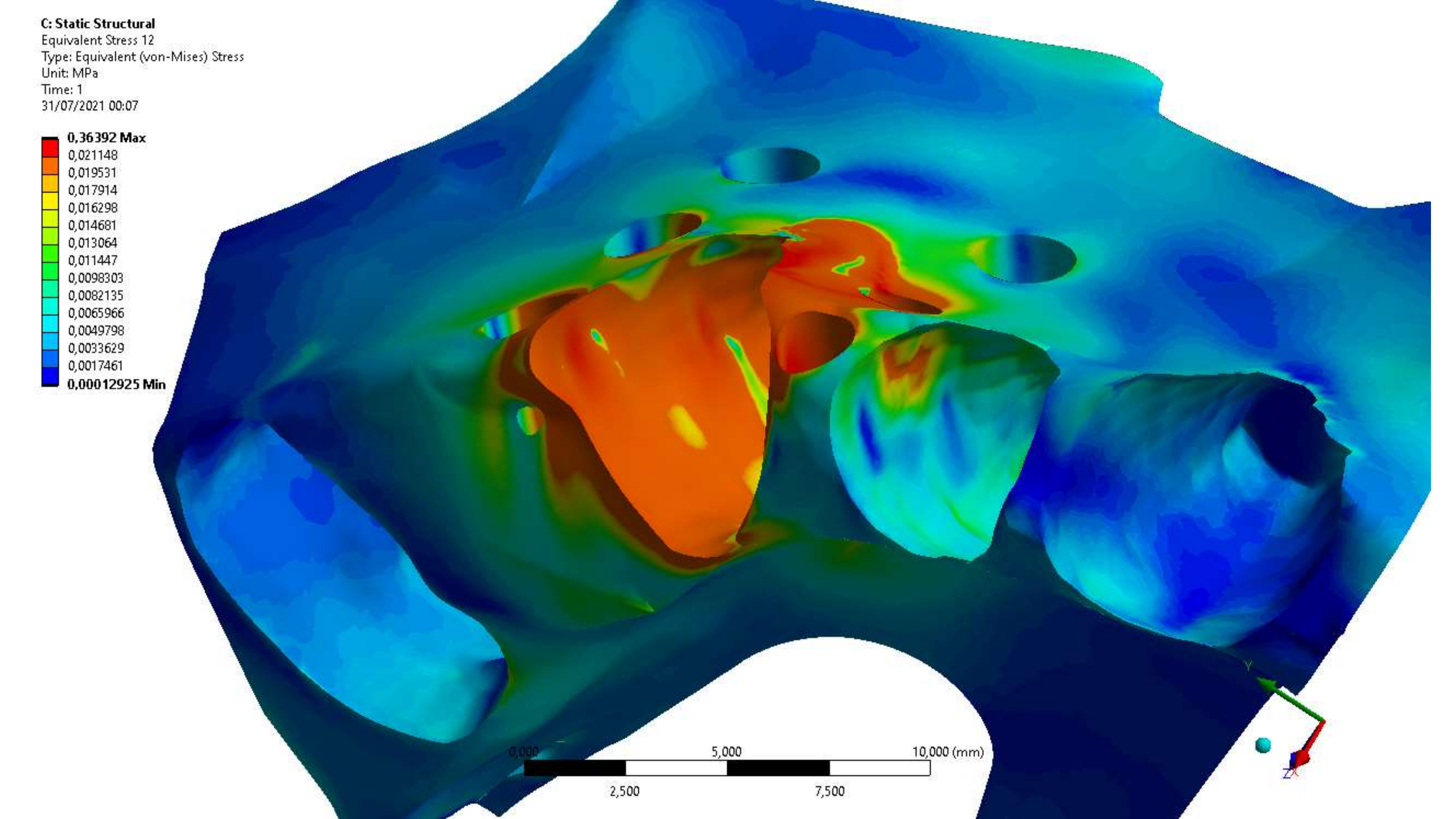
C: Static Structural
Equivalent Stress 12
Type: Equivalent (von-Mises) Stress
Unit: MPa
Time: 1
31/07/2021 00:07
0,36392 Max
0,021148
0,019531
0,017914
0,016298
0,014681
0,013064
0,011447
0,0098303
0,0082135
0,0065966
0,0049798
0,0033629
0,0017461
0,00012925 Min
0,000
5,000
10,000 (mm)
2,500
7,500

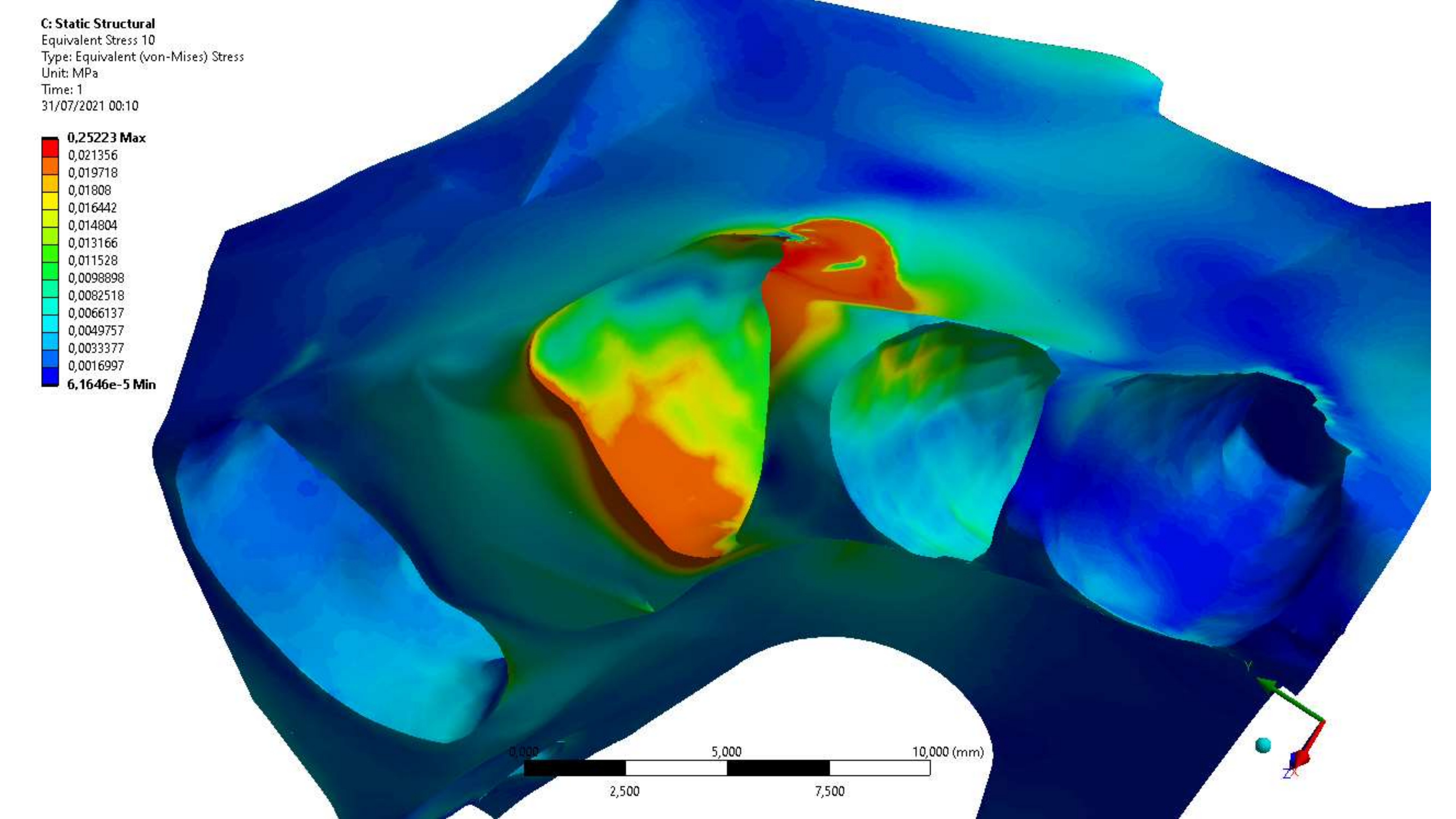

C: Static Structural
Equivalent Stress 10
Type: Equivalent (von-Mises) Stress
Unit: MPa
Time: 1
31/07/2021 00:10
0,25223 Max
0,021356
0,019718
0,01808
0,016442
0,014804
0,013166
0,011528
0,0098898
0,0082518
0,0066137
0,0049757
0,0033377
0,0016997
6,1646e-5 Min
0,000
5,000
10,000 (mm)
2,500
7,500

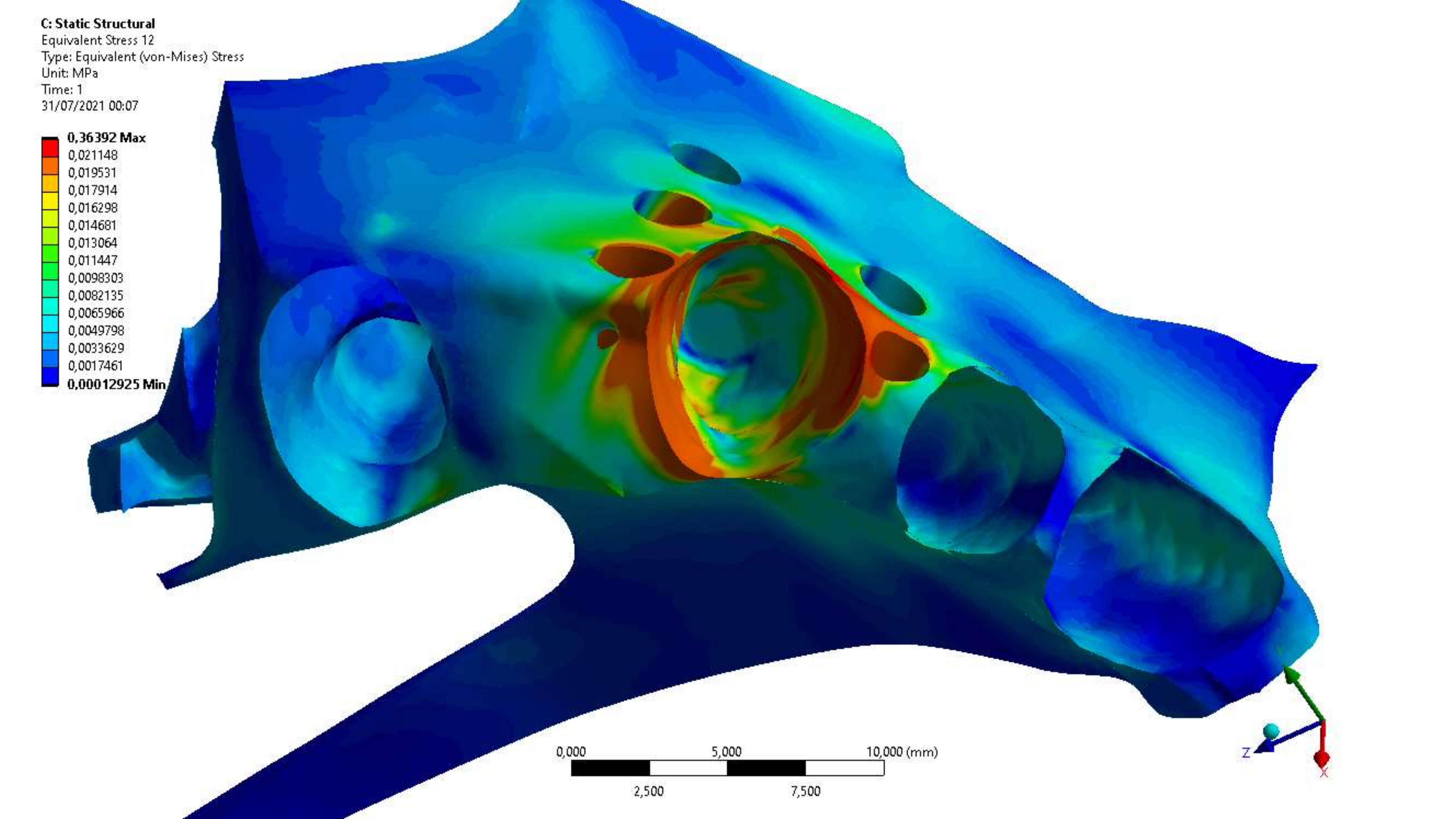
C: Static Structural
Equivalent Stress 12
Type: Equivalent (von-Mises) Stress
Unit: MPa
Time: 1
31/07/2021 00:07
0,36392 Max
0,021148
0,019531
0,017914
0,016298
0,014681
0,013064
0,011447
0,0098303
0,0082135
0,0065966
0,0049798
0,0033629
0,0017461
0,00012925 Min
0,000
5,000
10,000 (mm)
2,500
7,500
Z
X

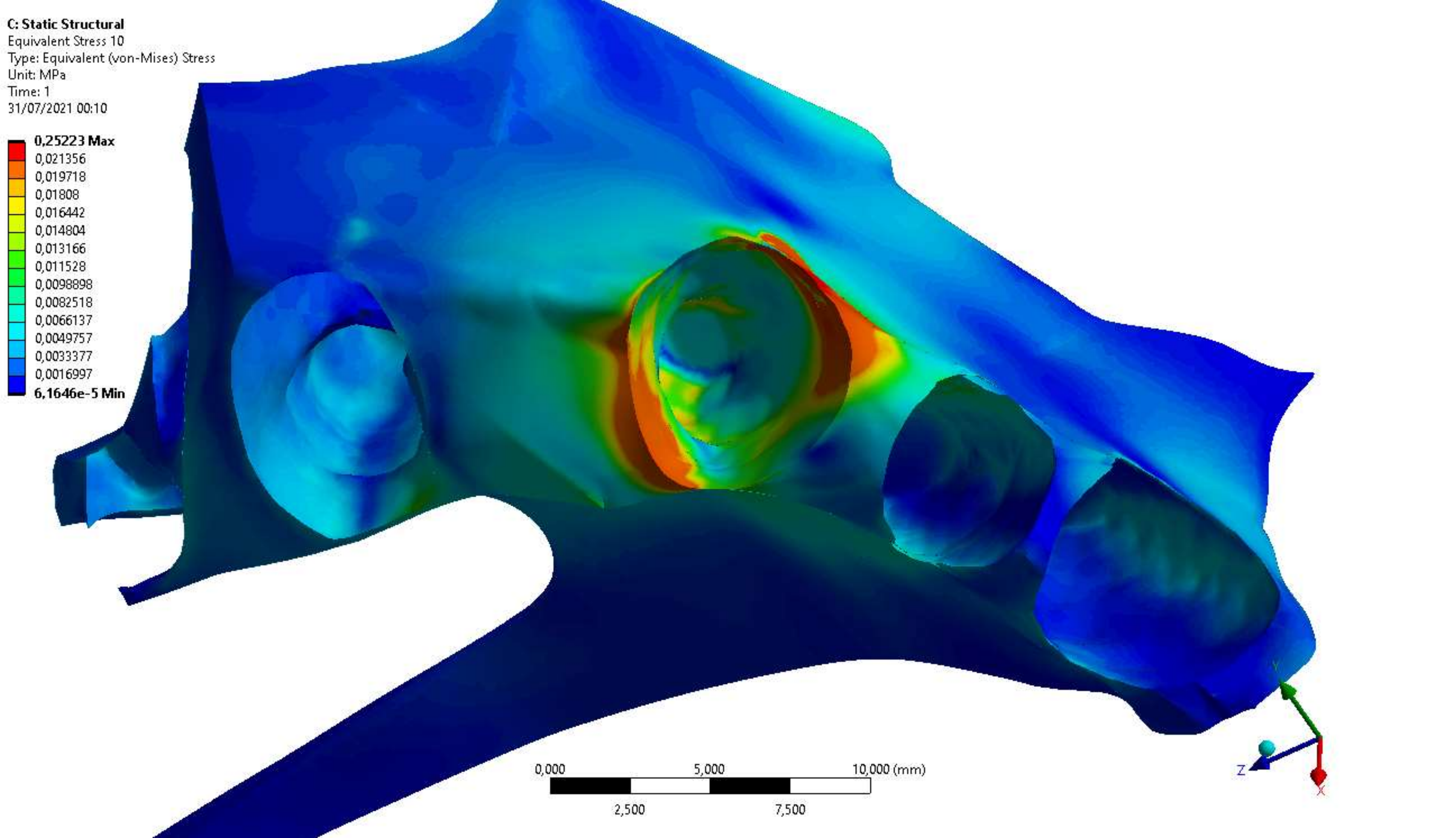
C: Static Structural
Equivalent Stress 10
Type: Equivalent (von-Mises) Stress
Unit: MPa
Time: 1
31/07/2021 00:10
0,25223 Max
0,021356
0,019718
0,01808
0,016442
0,014804
0,013166
0,011528
0,0098898
0,0082518
0,0066137
0,0049757
0,0033377
0,0016997
6,1646e-5 Min
0,000
5,000
10,000 (mm)
2,500
7,500

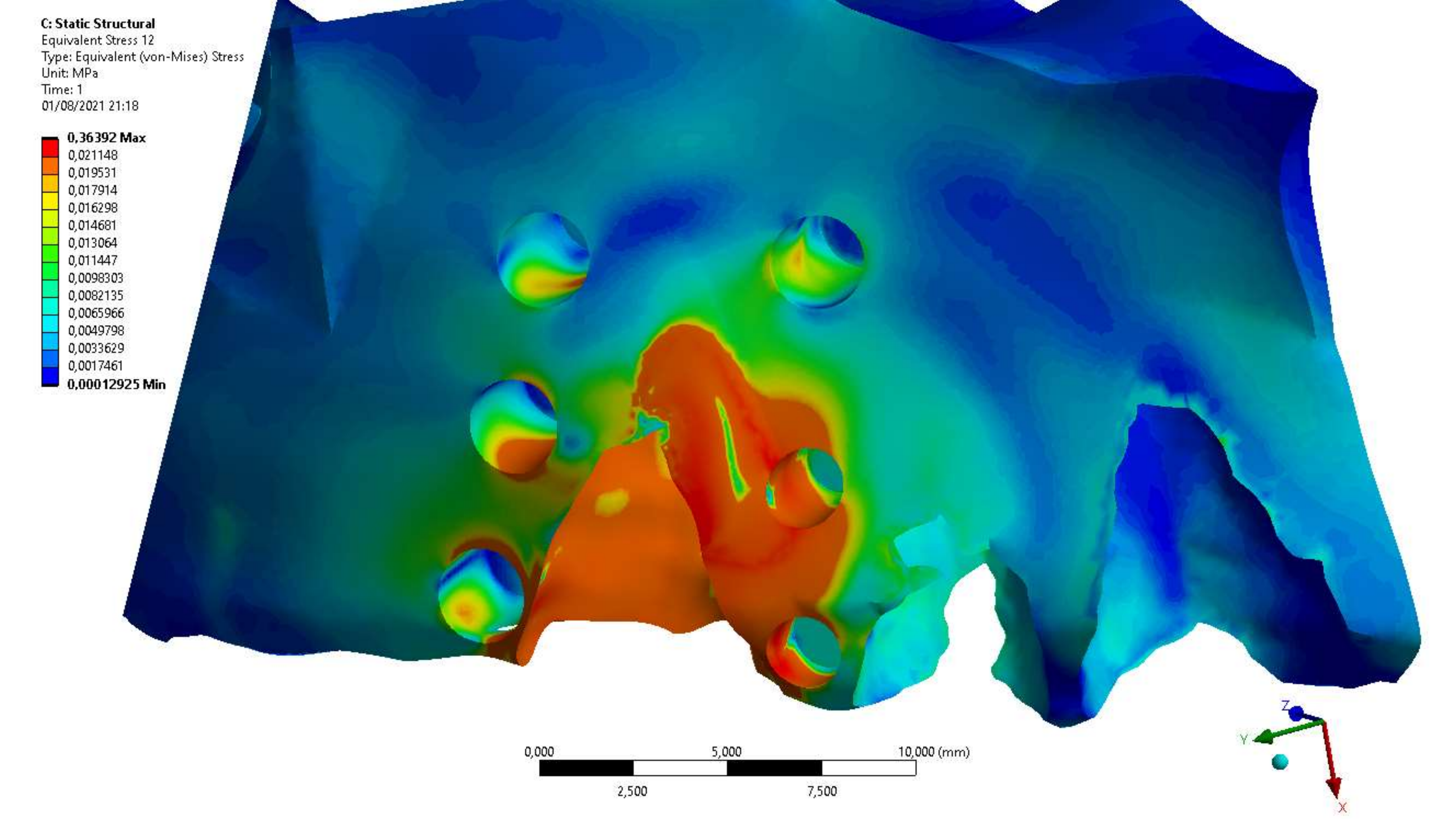
C: Static Structural
Equivalent Stress 12
Type: Equivalent (von-Mises) Stress
Unit: MPa
Time: 1
01/08/2021 21:18
0,36392 Max
0,021148
0,019531
0,017914
0,016298
0,014681
0,013064
0,011447
0,0098303
0,0082135
0,0065966
0,0049798
0,0033629
0,0017461
0,00012925 Min
0,000
5,000
10,000 (mm)
2,500
7,500
Z
Y
X

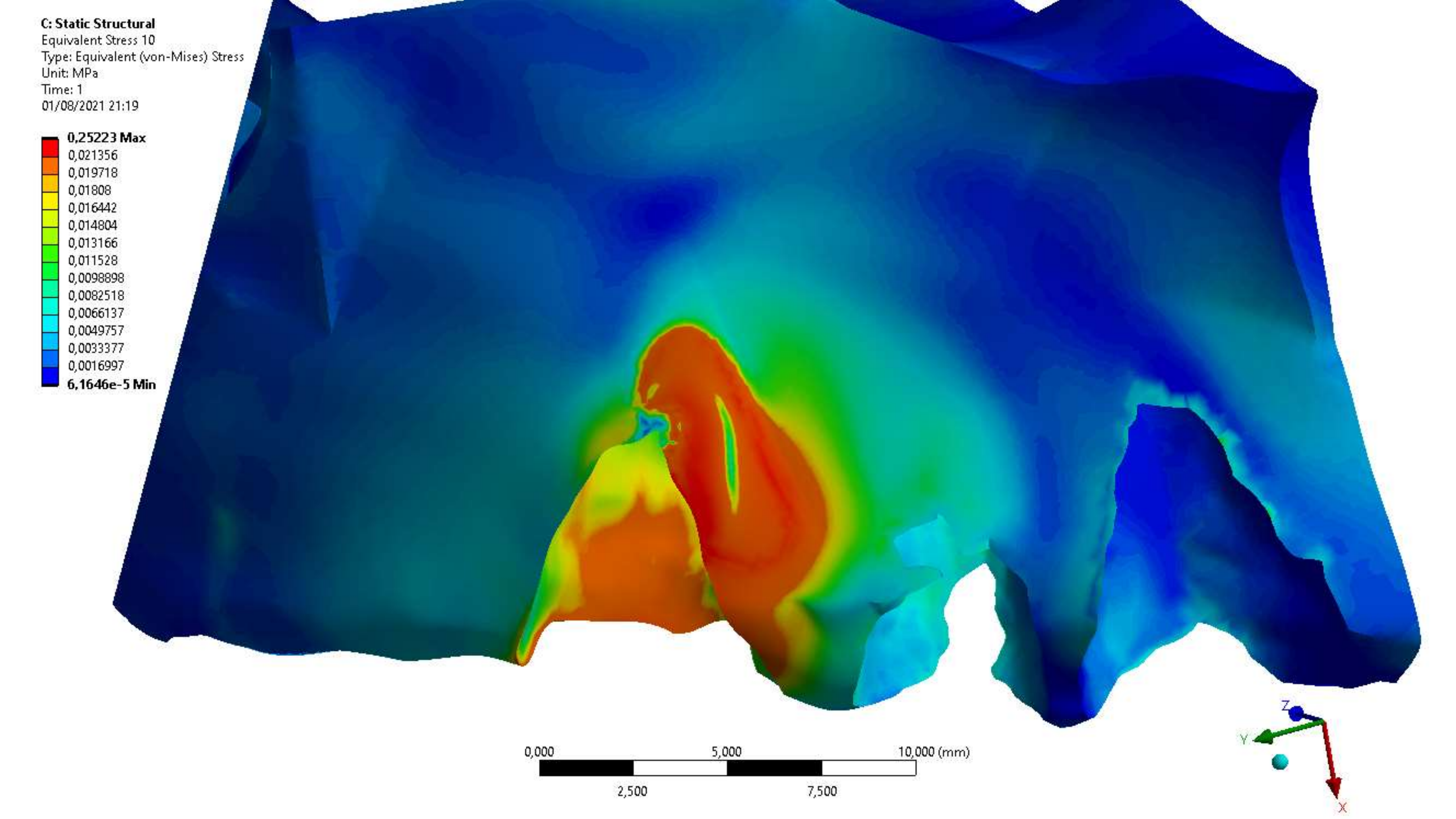
C: Static Structural
Equivalent Stress 10
Type: Equivalent (von-Mises) Stress
Unit: MPa
Time: 1
01/08/2021 21:19
0,25223 Max
0,021356
0,019718
0,01808
0,016442
0,014804
0,013166
0,011528
0,0098898
0,0082518
0,0066137
0,0049757
0,0033377
0,0016997
6,1646e-5 Min
0,000
5,000
10,000 (mm)
2,500
7,500
Z
Y
X

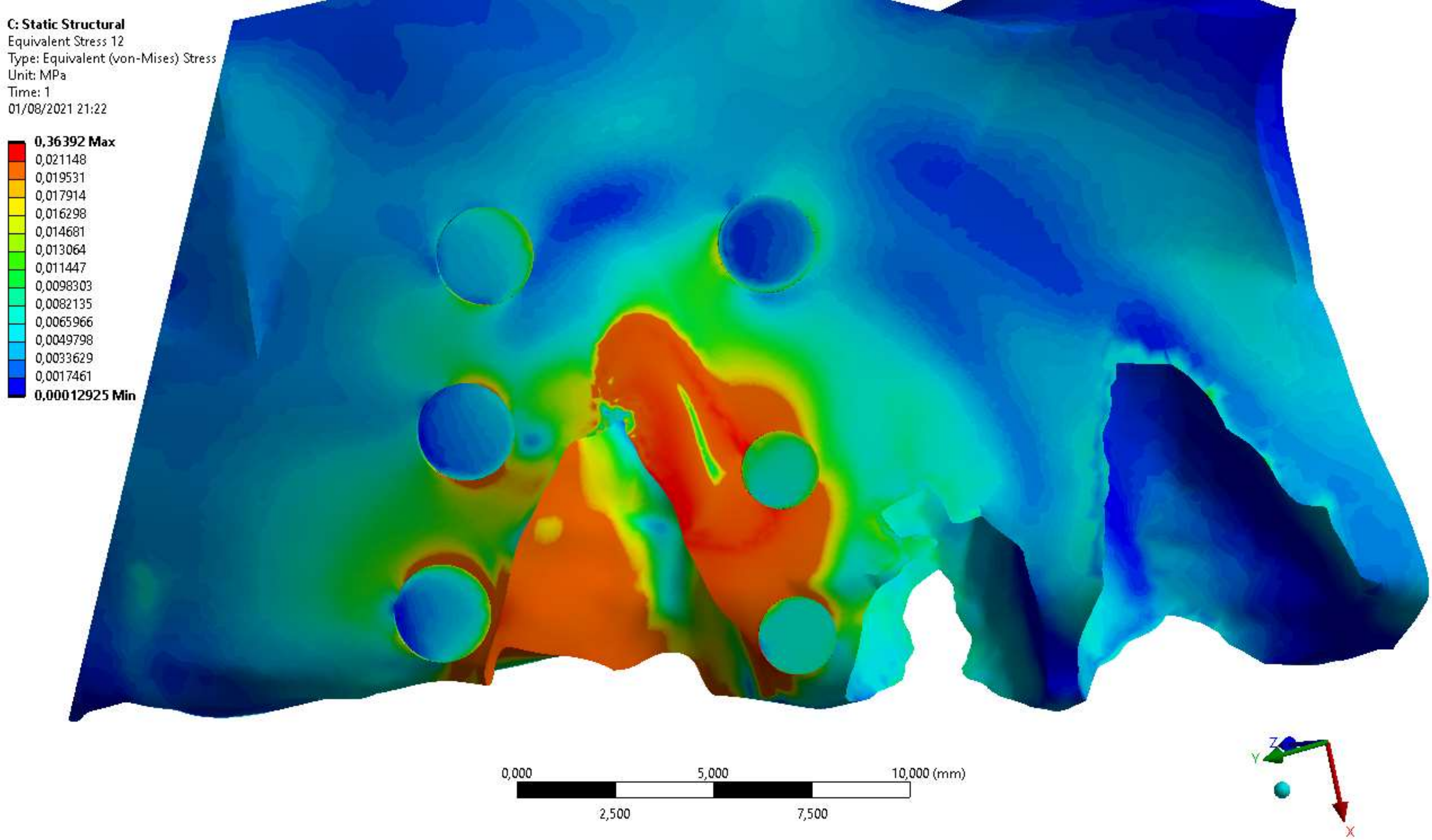
C: Static Structural
Equivalent Stress 12
Type: Equivalent (von-Mises) Stress
Unit: MPa
Time: 1
01/08/2021 21:22
0,36392 Max
0,021148
0,019531
0,017914
0,016298
0,014681
0,013064
0,011447
0,0098303
0,0082135
0,0065966
0,0049798
0,0033629
0,0017461
0,00012925 Min
0,000
5,000
10,000 (mm)
2,500
7,500
Z
Y
X

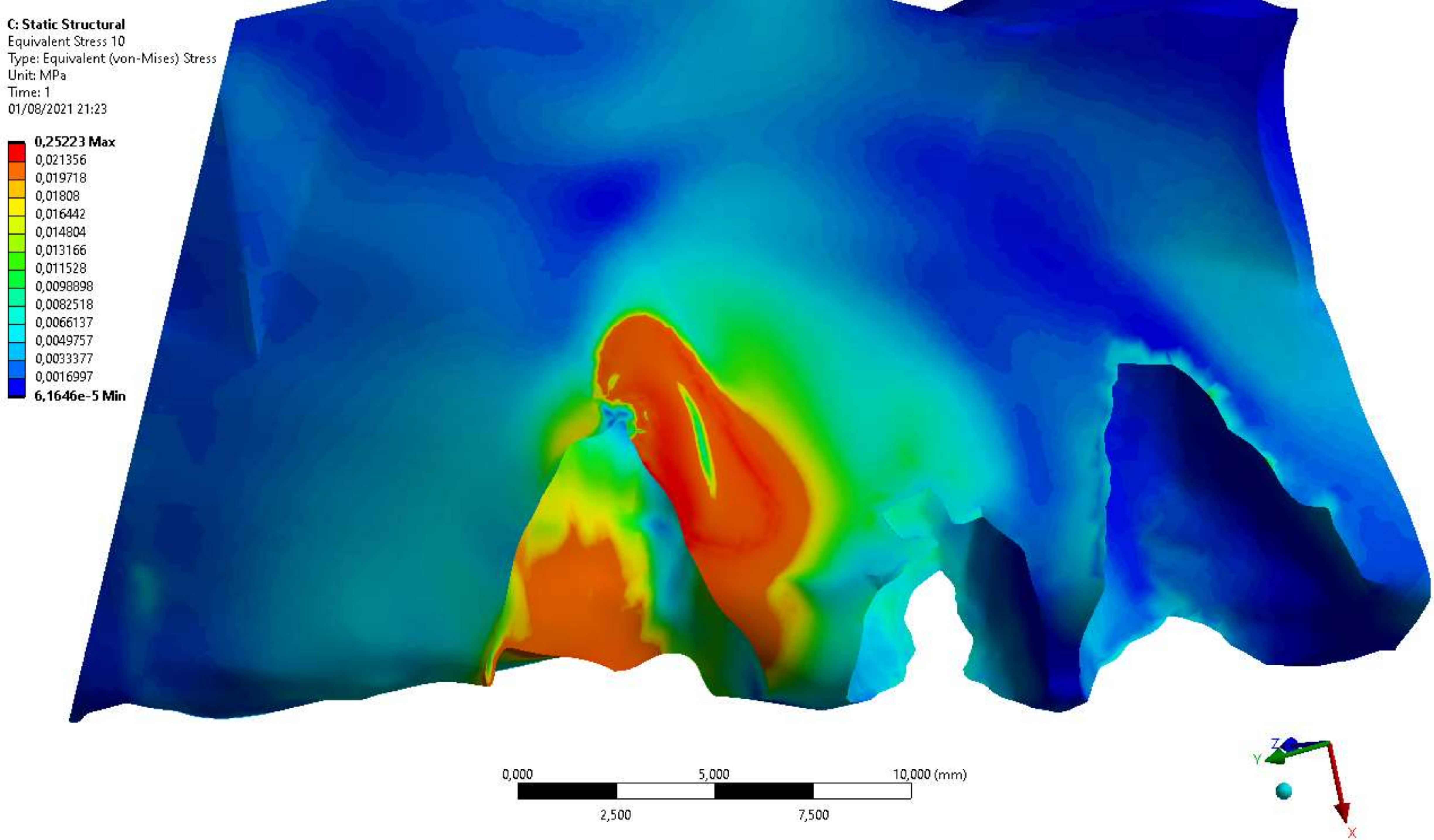
C: Static Structural
Equivalent Stress 10
Type: Equivalent (von-Mises) Stress
Unit: MPa
Time: 1
01/08/2021 21:23
0,25223 Max
0,021356
0,019718
0,01808
0,016442
0,014804
0,013166
0,011528
0,0098898
0,0082518
0,0066137
0,0049757
0,0033377
0,0016997
6,1646e-5 Min
0,000
5,000
10,000 (mm)
2,500
7,500
Z
Y
X
