# Supplementary material for: Micro-osteoperforation for enhancement of orthodontic movement: A mechanical analysis using the finite element method
Source: PLoS One. 2024 Aug 19;19(8):e0308739. doi: 10.1371/journal.pone.0308739 (PMC11332926; doi:10.1371/journal.pone.0308739)
Supplement: Attachment — Submitted filename: doc.docx [file pone.0308739.s015.docx]

What are the findings regarding the immediate tooth movement and stress distribution after the application of simulated orthodontic force with and without micro-osteoperforations?

How did the finite element method (FEM) demonstrate the impact of micro-osteoperforations on tooth movement and stress distribution on the dental crown, periodontal ligament, and bone structure?

What are the limitations of the study in terms of cellular and tissue response and how does the study contribute to filling a gap in the previously published clinical trials?

What are the potential implications of the study's findings on orthodontic treatment outcomes and the use of micro-osteoperforations in clinical practice?
